# Assessing ChatGPT as a Medical Consultation Assistant for Chronic Hepatitis B: Cross-Language Study of English and Chinese

**DOI:** 10.2196/56426

**Published:** 2024-08-08

**Authors:** Yijie Wang, Yining Chen, Jifang Sheng

**Affiliations:** 1 State Key Laboratory for Diagnosis and Treatment of Infectious Diseases Collaborative Innovation Center for Diagnosis and Treatment of Infectious Disease The First Affiliated Hospital, Zhejiang University School of Medicine Hangzhou China; 2 Department of Urology Sir Run Run Shaw Hospital Zhejiang University School of Medicine Hangzhou China

**Keywords:** chronic hepatitis B, artificial intelligence, large language models, chatbots, medical consultation, AI in health care, cross-linguistic study

## Abstract

**Background:**

Chronic hepatitis B (CHB) imposes substantial economic and social burdens globally. The management of CHB involves intricate monitoring and adherence challenges, particularly in regions like China, where a high prevalence of CHB intersects with health care resource limitations. This study explores the potential of ChatGPT-3.5, an emerging artificial intelligence (AI) assistant, to address these complexities. With notable capabilities in medical education and practice, ChatGPT-3.5’s role is examined in managing CHB, particularly in regions with distinct health care landscapes.

**Objective:**

This study aimed to uncover insights into ChatGPT-3.5’s potential and limitations in delivering personalized medical consultation assistance for CHB patients across diverse linguistic contexts.

**Methods:**

Questions sourced from published guidelines, online CHB communities, and search engines in English and Chinese were refined, translated, and compiled into 96 inquiries. Subsequently, these questions were presented to both ChatGPT-3.5 and ChatGPT-4.0 in independent dialogues. The responses were then evaluated by senior physicians, focusing on informativeness, emotional management, consistency across repeated inquiries, and cautionary statements regarding medical advice. Additionally, a true-or-false questionnaire was employed to further discern the variance in information accuracy for closed questions between ChatGPT-3.5 and ChatGPT-4.0.

**Results:**

Over half of the responses (228/370, 61.6%) from ChatGPT-3.5 were considered comprehensive. In contrast, ChatGPT-4.0 exhibited a higher percentage at 74.5% (172/222; *P*<.001). Notably, superior performance was evident in English, particularly in terms of informativeness and consistency across repeated queries. However, deficiencies were identified in emotional management guidance, with only 3.2% (6/186) in ChatGPT-3.5 and 8.1% (15/154) in ChatGPT-4.0 (*P*=.04). ChatGPT-3.5 included a disclaimer in 10.8% (24/222) of responses, while ChatGPT-4.0 included a disclaimer in 13.1% (29/222) of responses (*P*=.46). When responding to true-or-false questions, ChatGPT-4.0 achieved an accuracy rate of 93.3% (168/180), significantly surpassing ChatGPT-3.5’s accuracy rate of 65.0% (117/180) (*P*<.001).

**Conclusions:**

In this study, ChatGPT demonstrated basic capabilities as a medical consultation assistant for CHB management. The choice of working language for ChatGPT-3.5 was considered a potential factor influencing its performance, particularly in the use of terminology and colloquial language, and this potentially affects its applicability within specific target populations. However, as an updated model, ChatGPT-4.0 exhibits improved information processing capabilities, overcoming the language impact on information accuracy. This suggests that the implications of model advancement on applications need to be considered when selecting large language models as medical consultation assistants. Given that both models performed inadequately in emotional guidance management, this study highlights the importance of providing specific language training and emotional management strategies when deploying ChatGPT for medical purposes. Furthermore, the tendency of these models to use disclaimers in conversations should be further investigated to understand the impact on patients’ experiences in practical applications.

## Introduction

### Chronic Hepatitis B: A Dual Burden on Patients and Society

Chronic hepatitis B (CHB) imposes significant economic and social burdens. In 2019, approximately 296 million people were affected by CHB, resulting in an estimated 820 thousand deaths [1]. The World Health Organization (WHO) noted that among those chronically infected with hepatitis B and C, about 20% or more would develop end-stage chronic liver disease, such as cirrhosis and hepatocellular carcinoma [2].

Hepatitis B virus (HBV) primarily spreads through blood contact, unprotected sexual intercourse, and mother-to-infant transmission. Effective management of chronic infection necessitates daily monitoring and self-care [3]. Nevertheless, the intricacy of regular monitoring, encompassing multiple tests, such as hepatitis B surface antigen (HBsAg), hepatitis B e-antigen (HBeAg), HBV-DNA, alanine transaminase (ALT), and fibrosis assessment, as endorsed by authoritative bodies in hepatitis B diagnosis and treatment, including the European Association for the Study of the Liver (EASL), presents hurdles to patient compliance [4]. Additionally, the prolonged, often lifelong, administration of antiviral medications contributes to further adherence issues [4,5]. Unique considerations for pregnant individuals and children add another layer of complexity, demanding targeted counseling and specialized management. This intricate management landscape not only burdens patients with emotional stress but also jeopardizes adherence to treatment regimens [6,7]. The complexity of CHB management requires personalized health care strategies, easing individual and societal burdens and emphasizing the importance of diverse health approaches.

### ChatGPT as a Prospective Medical Assistant

Currently, artificial intelligence (AI) has become integral in the medical domain, particularly in medical research and clinical practice. Notably, according to Wani et al [8], traditional machine learning methodologies require the supervision of skilled individuals and structured input data, resulting in considerable resource-intensive processes. Recognizing the limitations of traditional approaches, Haug et al [9] proposed chatbots for capabilities in medical practice assistance.

ChatGPT-3.5, which was released in June 2020, underpins ChatGPT’s emergence in AI-assisted medical applications. As a large language model (LLM), it shows potential for medical assistance [10], though challenges and concerns persist in its application within the field [11]. The functioning of LLMs involves predicting and generating a coherent and contextually relevant response based on preinput materials, necessitating training on massive amounts of diverse textual data. Various studies have explored ChatGPT’s capacity to act as a virtual doctor or medical tutor for diagnosis or treatment [12].

The study by Gilson et al [13] revealed that ChatGPT performed well in medical knowledge assessments, demonstrating potential as a virtual medical tutor. Yeo et al [14] evaluated ChatGPT’s performance in answering questions regarding cirrhosis and hepatocellular carcinoma. Most studies have compared its performance to that of real doctors or medical students, aiming to determine whether AI assistants could surpass human medical service providers. However, there are challenges and risks of employing ChatGPT in clinical practice, including the potential generation of plausible yet inaccurate information and ethical considerations [15]. According to these studies, LLMs could potentially assist in medical consulting and auxiliary diagnosis, as well as traditional medical research, treatment, and education, but there are still unidentified risks and problems.

ChatGPT-4.0, which was released on March 14, 2023, is an updated version of the ChatGPT model. Many researchers have compared the applications of ChatGPT-3.5 and ChatGPT-4.0 in medical practice [16-18]. In this research, we included ChatGPT-4.0 as a comparative model to further assess the application problem of this model.

### Medical Assistance in Hepatitis B Management With Chinese as the Primary Language

Bearing the highest global burden of hepatitis B, China recorded over 90 million people living with CHB in 2017 [1,2]. Research has revealed troubling trends in treatment noncompliance for HBV in China, including challenges in preventing vertical transmission [19-21]. Beyond China, studies in various regions have highlighted the impact of factors like family income, employment, and patient gender on medical treatment compliance for CHB [22].

Physician encouragement is crucial for patient compliance with medication regimens [23]. Despite a rising number of medical doctors in China, there is a shortage of medical practitioners, including licensed physicians and physician assistants, who face high workloads and burnout rates [24-26]. While research has indicated that Chinese physicians generally adhere to hepatitis B guidelines [27], medical errors due to workload demands could undermine intended impacts on patient compliance [26]. Amidst these challenges, exploring medical assistance using Chinese as the primary working language is crucial. This inquiry is vital for enhancing patient compliance in hepatitis B management and alleviating strain on health care professionals amid work-related stress. However, a study involving ChatGPT’s performance in a medical examination in Chinese emphasized the significance of exploring the cross-language difference in ChatGPT’s performance in a future study [28].

In brief, a dialogue-based medical assistant is being increasingly recognized as essential in clinical practice. Exploring the application of ChatGPT in this domain shows promise for medical research and clinical usage. This study assessed ChatGPT-3.5 and ChatGPT-4.0 in tasks such as diagnosis, providing management advice, and addressing counseling needs among patients with CHB. Given that English data account for the largest proportion of data (approximately 92%) used for the original training of this model [29], it is reasonable to assume that among all the languages included in the pretraining resource, this model performs best in English. However, the Chinese language is used by the world’s largest group of CHB patients, underscoring the irreplaceable role of Chinese in studies regarding medical AI assistants. Therefore, this research includes both English and Chinese as working languages and compares ChatGPT’s performance in both languages. Additionally, the study compares ChatGPT-3.5 and ChatGPT-4.0 to investigate the improvements from the former version to the latter version. Through this investigation, we aim to uncover the potential of this application and its limitations in medical practice.

## Methods

### Questionnaire Development Process

**Figure 1:**
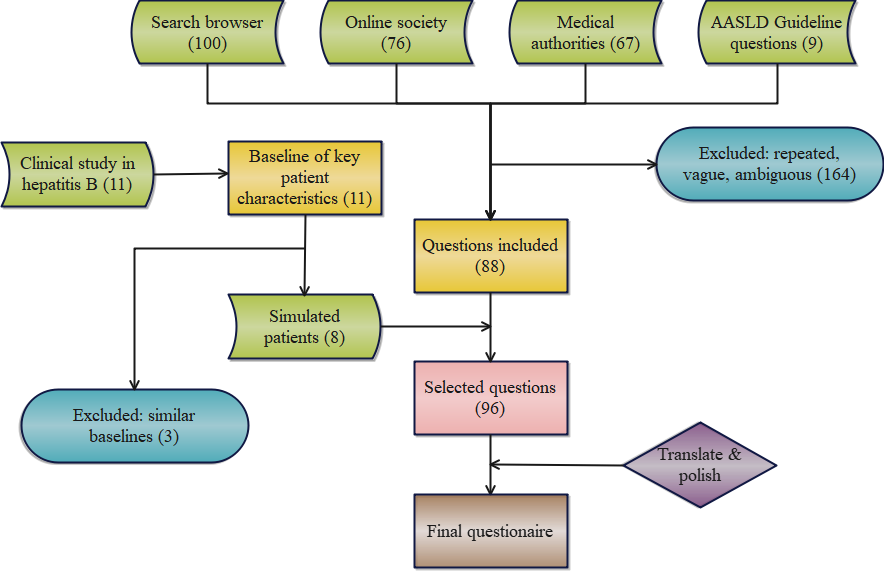
Workflow of the questionnaire design process. The specific information of each stage of the questionnaire compiling process is shown. AASLD: American Association for the Study of Liver Disease.

Following the workflow shown in Figure 1, we systematically compiled a set of questions relevant to both patients and physicians in clinical practice. This compilation process involved several processes. First, we sourced questions from esteemed professional associations and institutions, such as the WHO, American Centers for Disease Control and Prevention (CDC), American Association for the Study of Liver Disease (AASLD), EASL, and Asian Pacific Association for the Study of the Liver (APASL). Second, we identified queries about hepatitis B found on online social media platforms, particularly in patient support groups and disease-specific forums. The inclusion criteria prioritized relevance to diagnosis, treatment, daily monitoring, lifestyle, and other hepatitis B–related concerns. Questions with precise wording, minimal grammatical errors, and clarity were included, while nonmedical inquiries, ambiguously framed questions, and those related to nonmedical issues were excluded. Questions with significant updates after September 2021 were also omitted. Third, we conducted an exploration of associative keywords following the entry of “hepatitis B” or “HBV” into widely used search engines, such as Google, Bing, and Baidu, in both Chinese and English languages. Fourth, based on diverse published hepatitis B clinical studies, we systematically extracted key patient characteristics, including age, gender, hepatitis B serum markers, HBV-DNA levels, ALT levels, and concomitant diseases. We developed profiles for 8 simulated patients using these data with a random number function. ChatGPT was tasked with providing advice to these simulated individuals on various aspects, including treatment or examination recommendations, treatment strategies, daily monitoring practices, lifestyle adjustments, etc.

Among all the questions gathered, multiple questions were separated into single entities, while repeated questions were excluded. To avoid ambiguity and misunderstanding resulting from language vagueness, which could potentially impact the assessment of the model’s information accuracy, we carefully polished all the collected questions, refined their grammar and phrasing, and performed localized translations between Chinese and English. Examples of revised or excluded questions are provided in Multimedia Appendix 1. In total, we gathered 96 questions about hepatitis B. Among the questions, there were 2 with only an English version and 5 with only a Chinese version. These language-specific questions focused on issues specific to the country or region where the questioner was located.

### Section Allocation

We systematically categorized all questions into 5 distinct sections: Term Explanation Questions, Short Answer Questions, Clinical Problem Questions, AASLD Guideline Questions, and Simulated Patient Questions.

The “Term Explanation Questions” section featured 17 terms associated with hepatitis B, including 1 term exclusively for Chinese responses. In the “Short Answer Questions” section, there were 22 questions, with 1 specifically designed for Chinese responses. Questions within the “Clinical Problem Questions” section were primarily sourced from online hepatitis B societies, totaling 40 questions. Within this section, there was 1 question intended solely for English responses and 2 exclusively for Chinese responses. The questions in the “AASLD Guideline Questions” section were derived from the AASLD guidelines for hepatitis B in 2016 and 2018 (updated version) and included 9 questions that were all translated into Chinese. The “Simulated Patient Questions” section consisted of 8 questions related to simulated patient information, as previously constructed. These questions were provided in both English and Chinese versions.

### Gathering Responses

The questions were submitted to ChatGPT-3.5 from April 1 to April 30, 2023, with each question forming a separate dialogue. Each question was sent twice for Chinese and English separately to ensure a comprehensive evaluation, and responses were collected. In the case of a system error preventing ChatGPT-3.5 from responding, the question was resubmitted in a new dialogue. All responses were compiled into a table for further assessment.

### Assessment of Responses

Two senior physicians independently evaluated all responses. In the case of discrepancies in information accuracy, consistency of repeated responses, and emotional management guidance assessments, a third senior physician with over 30 years of experience in hepatitis B diagnosis and treatment conducted a final review for the ultimate assessment and provided the final scores. The criterion of assessment was discussed and voted on by a committee of 5 senior physicians in hepatitis B diagnosis and treatment. The assessment process referred to the research of Yeo et al [14].

#### Information Accuracy Assessment

The information accuracy assessment was mainly focused on correctness and comprehensiveness. Four assessment grades (1-4) were assigned: 1, correct and comprehensive; 2, correct but with missing information; 3, a mix of correct and incorrect details; and 4, wholly incorrect or irrelevant information.

#### Categorization of the Types of Mistakes

Mistakes in responses assessed as “a mix of correct and incorrect details” and “wholly incorrect or irrelevant information” were analyzed, and the types of mistakes were categorized. The mistakes were classified into 5 categories: A, misunderstanding of medical terms or jargon; B, incorrect usage of medical terms; C, mistakes in diagnosis/treatment/management without mistakes in terms or jargon; D, total irrelevant information; and E, a mixture of two or more kinds of mistakes among A-C.

#### Content Consistency of Repeated Response Assessment

A binary assessment (“Yes” or “No”) was employed to indicate the consistency of the 2 responses for each question. This evaluation was independent of the information accuracy assessment and solely focused on the consistency of the response content.

#### Emotional Management Guidance Assessment

For all responses in the “Clinical Problem Questions” and “Simulated Patient Questions” sections (48 in total; 1 with only an English version and 2 with only a Chinese version), an emotional management guidance assessment was conducted. The assessment comprised three levels: (1) sufficient emotional and psychological management guidance, (2) respectful but lacking or inadequate emotional or psychological management guidance, and (3) disrespectful or negative emotional guidance.

#### Analysis of ChatGPT's Cautionary Statements Regarding Medical Advice

We quantified the instances where ChatGPT recommended consulting a genuine health care provider or doctor. Meanwhile, we counted the frequency of ChatGPT explicitly stating disclaimers, such as “I am not a doctor” and “I cannot give diagnosis or treatment,” among all questions involving clinical practice (including the sections of Clinical Problem Questions, AASLD Guideline Questions, and Simulated Patient Questions).

#### Parallel Assessment of ChatGPT-4.0’s Performance

We replicated the above assessment process for ChatGPT-4.0. Considering that ChatGPT-4.0 is the updated version of the model, we omitted sections involving only basic medical knowledge in the questionnaire. As a more intuitive alternative, we chose closed questions to evaluate the fundamental knowledge differences between the 2 model versions. The assessment of ChatGPT-4.0 included questions from the “Clinical Problem Questions,” “AASLD Guideline Questions,” and “Simulated Patient Questions” sections of the questionnaires used in the previous assessment. However, mistake analysis was omitted as there were no responses from ChatGPT-4.0 that were assessed as incorrect.

#### Comparison of ChatGPT-3.5 and ChatGPT-4.0 Using Closed Questions (True-or-False Statements)

In this assessment, we formulated 30 statements based on AASLD guidelines for the treatment of CHB, including all its updates up to September 2021. These statements were input into the models in separate dialogues. We used prompts to ask the models to judge whether the statements were correct and to provide a judgment with “Yes” or “No.” The prompts are detailed in Table 1. Each statement was input into the model 3 times, and the response for each iteration was recorded. All responses of the models were collected, and their accuracy and stability (the consistency of 3 responses to a repeated statement) were assessed.

**Table 1:** An example of the prompts used in closed questions.

Language	Prompts for ChatGPT-4.0	Prompts for ChatGPT-3.5
English	Now, I would like you to act as a hepatologist in the upcoming conversation and determine whether the statements are true and answer with only “Yes” or “No”. Here are the statements: []^a^	Now, I would like you to act as a hepatologist in the upcoming conversation and determine whether the statements are true and answer with only “Yes” or “No”, and do not add any explanation. Here are the statements: []
Chinese	现在，我希望你在接下来的对话中扮演一名肝病学医师，判断以下陈述是否正确，并用“是”或“否”来回答: []^a^	现在，我希望你在接下来的对话中扮演一名肝病学医师，判断以下陈述是否正确，并用“是”或“否”来回答，不要增加任何解释或说明: []

^a^The statements were added in square brackets.

### Statistical Analysis

All statistical analyses were performed with the SPSS 26.0 statistical package (IBM Corp). Cohen kappa coefficients were used to determine interobserver reliabilities. Assessment grades were calculated and reported as percentages. Comparative analysis of ranked data employed the Mann-Whitney test. Categorical data were compared using chi-square tests. The Wilcoxon signed rank test was applied to compare the grades of response 1 and response 2 to each question. Statistical significance was set at *P*<.05.

### Ethical Considerations

Our study did not use any information of real-world humans. Questions obtained from the internet were all polished and revised, and were included in the model with no personal information. Files of simulated patients were composed based on baseline data of clinical trials of hepatitis B, but the numbers were modified using a random number function to avoid possible leakage of real information.

## Results

### Information Accuracy Assessment of ChatGPT-3.5

**Figure 2:**
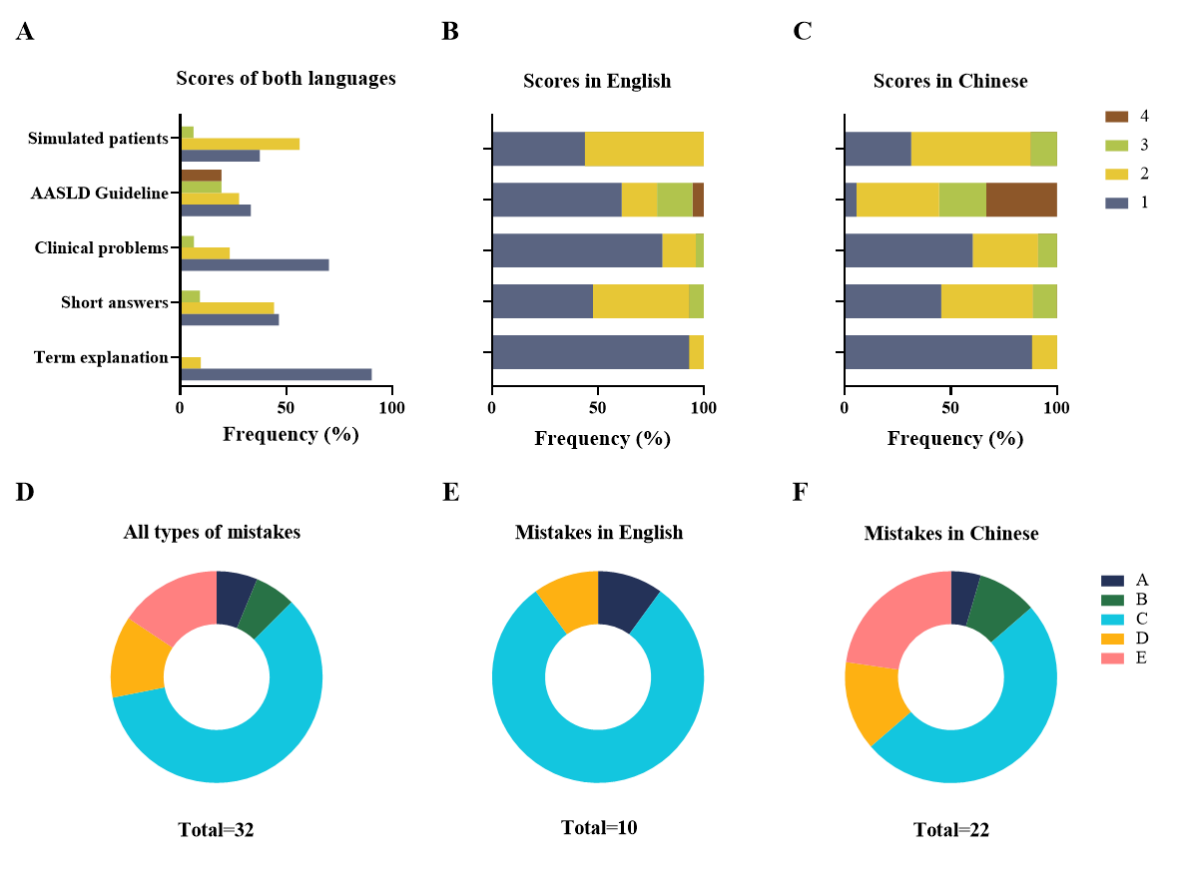
Results of the information accuracy assessment and mistake analysis of ChatGPT-3.5. (A) Comparison of the percentage for each grade across all responses in distinct question sections. (B) Percentage of each grade of responses in English in separated question sections. (C) Percentage of each grade of responses in Chinese in separated question sections. (D) Overview of mistake types across all responses. (E) Breakdown of mistake types specifically among responses in English. (F) Breakdown of mistake types specifically among responses in Chinese. In parts D-F, grade A indicates misunderstanding of medical terms or jargon, grade B indicates incorrect usage of medical terms, grade C indicates mistakes in diagnosis/treatment/management without mistakes in terms or jargon, grade D indicates total irrelevant information, and grade E indicates a mixture of two or more kinds of mistakes among grades A-C. AASLD: American Association for the Study of Liver Disease.

The interobserver reliability κ was 0.6020 (*P*<.001) for the information accuracy assessment. The results of this assessment are shown in Figure 2. Across all the questions, 90.8% (336/370) of responses from ChatGPT-3.5 contained no incorrect information (including comprehensive responses and correct but incomprehensive responses). The likelihood of ChatGPT giving correct and comprehensive responses was 61.6% (228/370), while there was a 29.2% (108/370) probability of responses being correct with missing information (Multimedia Appendix 2). Responses with a mix of correct and incorrect information accounted for 7.3% (27/370) of responses. There were 1.9% (7/370) of responses wholly incorrect or irrelevant to the questions.

The performance of ChatGPT-3.5 varied across the sections, and the differences were statistically significant (*P*<.0001; Multimedia Appendix 2). The section “Term Explanation Questions” had the highest percentage of responses assessed as complete and comprehensive (26/28, 93% in English and 30/34, 88% in Chinese; Figure 2A), while the section “AASLD Guideline Questions” had the highest percentage of responses totally wrong or irrelevant, or mixed with incorrect information (4/18, 22% in English and 10/18, 56% in Chinese).

The language environment in which ChatGPT-3.5 operated also influenced its performance (Figure 2B). ChatGPT demonstrated poorer performance in Chinese than in English (*P*=.001), particularly in the sections “Clinical Problem Questions” (*P*=.03) and “AASLD Guideline Questions” (*P*=.002). However, performance in the sections “Term Explanation Questions” (*P*=.54), “Short Answer Questions” (*P*=.62), and “Simulated Patient Questions” (*P*=.33) showed no significant difference between the 2 working languages. The evaluation table is presented in Multimedia Appendix 3.

### Categorization of the Types of Mistakes of ChatGPT-3.5

Figures 2D-F summarize the types of mistakes in the responses. In both languages, the most common error pertained to diagnosis, treatment, or disease management (Figure 2D). Notably, in Chinese, 10 out of 32 mistakes (31%) involved incorrect usage or misunderstanding of technical terms (Figure 2F), while in English, there were no such mistakes (Figure 2E). The evaluation table is provided in Multimedia Appendix 4.

### Content Consistency of the Repeated Response Assessment of ChatGPT-3.5

**Figure 3:**
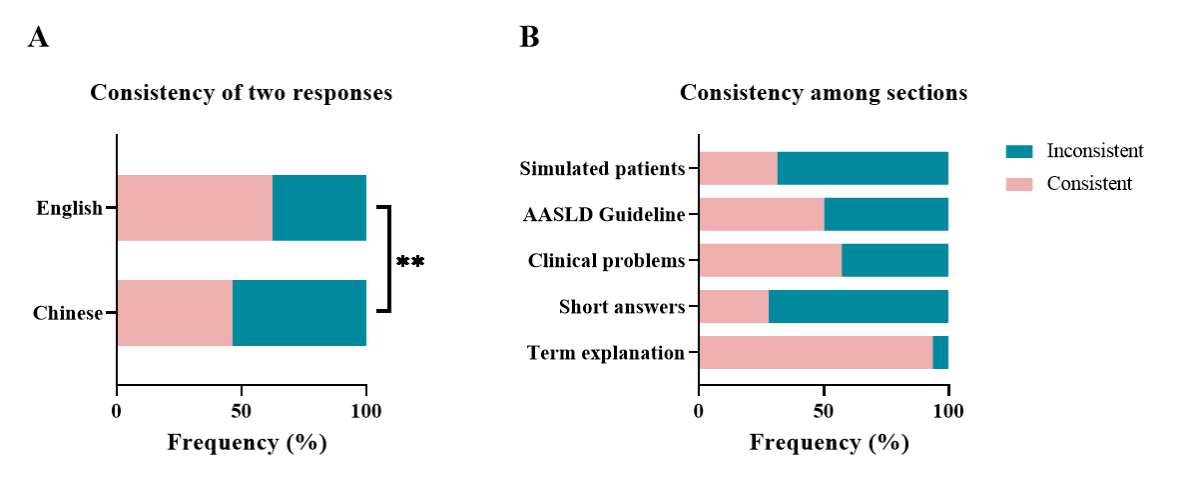
Assessment of the content consistency of responses to repeated questions. (A) Comparison of content consistency between responses in different working languages. (B) Examination of content consistency in different sections of questions. AASLD: American Association for the Study of Liver Disease. **P<.01.

The interobserver reliability κ was 0.6532 (*P*<.001) for content consistency of the repeated response assessment of ChatGPT-3.5. Figure 3 shows the content consistency of repeated responses. For all questions, the probability of content consistency between 2 responses was 54.1% (100/185 pairs of responses). In English, the consistency was 62% (56/90 pairs of responses), while in Chinese, it was 46% (44/95 pairs of responses), showing a significant difference (*P*=.04; Figure 3A and Multimedia Appendix 2). This disparity was also significant in the section “Clinical Problem Questions” (*P*=.04; Figure 3B and Multimedia Appendix 2). The “Term Explanation Questions” section had the highest consistency at 94% (29/31 pairs of responses), while the “Short Answer Questions” section had the lowest consistency at 30% (13/43 pairs of responses). Despite poor content consistency, the responses exhibited similarity in grades (*P*=.65). The evaluation table is provided in Multimedia Appendix 5.

### Emotional Management Guidance Assessment of ChatGPT-3.5

Among responses to questions within the “Clinical Problem Questions” and “Simulated Patient Questions” sections, only 3.2% (6/186) were deemed to provide sufficient emotional management support (Table 2). Related responses are listed in Multimedia Appendix 6. Most responses (180/186, 96.8%) were assessed as “respectful but lacking or inadequate emotional or psychological management guidance.” No response was assessed as “disrespectful or negative emotional guidance.” ChatGPT-3.5 exhibited comparable performance in both languages (*P*=.39).

**Table 2:** Results of the emotional management guidance assessment.

Language	Clinical Problem Questions (n=154), n				Simulated Patient Questions (n=32), n				Total (N=186), n				*P* value
	Grade 1	Grade 2	Grade 3	Grade 1		Grade 2	Grade 3	Grade 1		Grade 2	Grade 3		
Chinese	1	77	0	1		15	0	2		92	0	.48^a^	
English	4	72	0	0		16	0	4		88	0		
Total	5	149	0	1		31	0	6		180	0	.39^b^	

^a^*P* value across the grades of each section.

^b^*P* value between the grades of different working languages.

### Analysis of the Cautionary Statements of ChatGPT-3.5 Regarding Medical Advice

**Figure 4:**
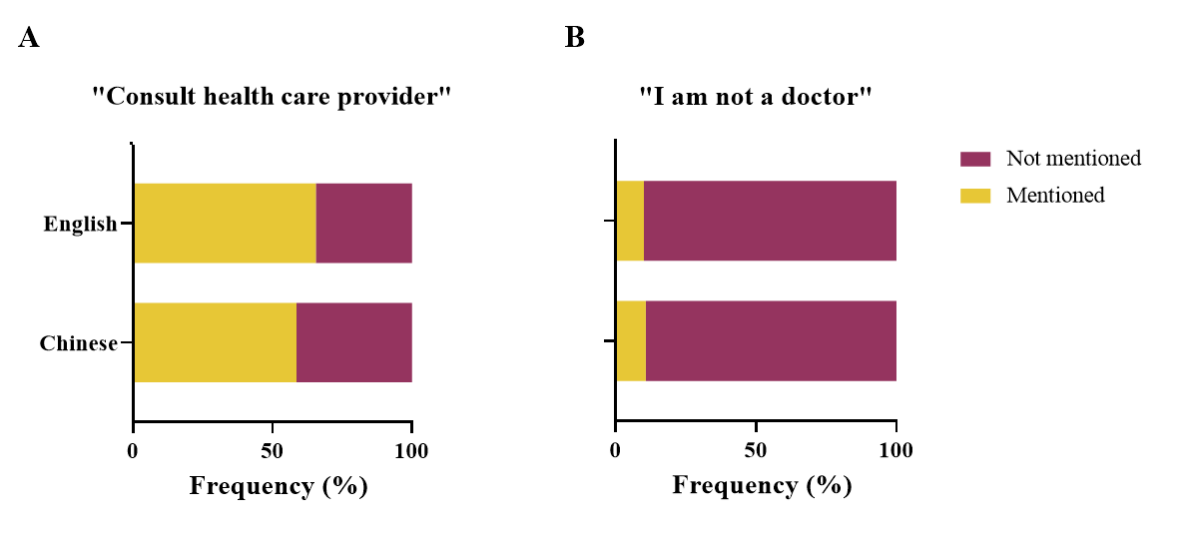
Percentage of ChatGPT cautionary statements regarding medical advice and disclaimers. (A) Percentage of responses that include the recommendation to “consult health care providers or doctors.” (B) Percentage of responses containing the disclaimer phrase “I am not a doctor” or “I cannot give diagnosis or treatment”.

Figure 4 shows the results of this assessment. ChatGPT-3.5 exhibited distinct characteristics as a medical assistant. In most responses, ChatGPT-3.5 tended to remind patients to consult a health care provider or a physician (227/370, 61.4%). This percentage was consistent in both English (118/180, 65.6%; Figure 4A) and Chinese (109/190, 57.4%), with no significant difference (*P*=.20). These responses are listed in Multimedia Appendix 7.

Among all questions involving clinical practice, the probability of ChatGPT-3.5 using the phrase “I am not a doctor” or “As a language model, I cannot give diagnosis or treatment…” was 10.8% (24/222). The probability was 11.6% (13/112) in Chinese and 10.0% (11/110) in English (Figure 4B). No significant difference was observed between the 2 languages (*P*>.99). These responses are listed in Multimedia Appendix 8.

### Parallel Assessment of ChatGPT-4.0 in Sections Involving Clinical Practice

**Figure 5:**
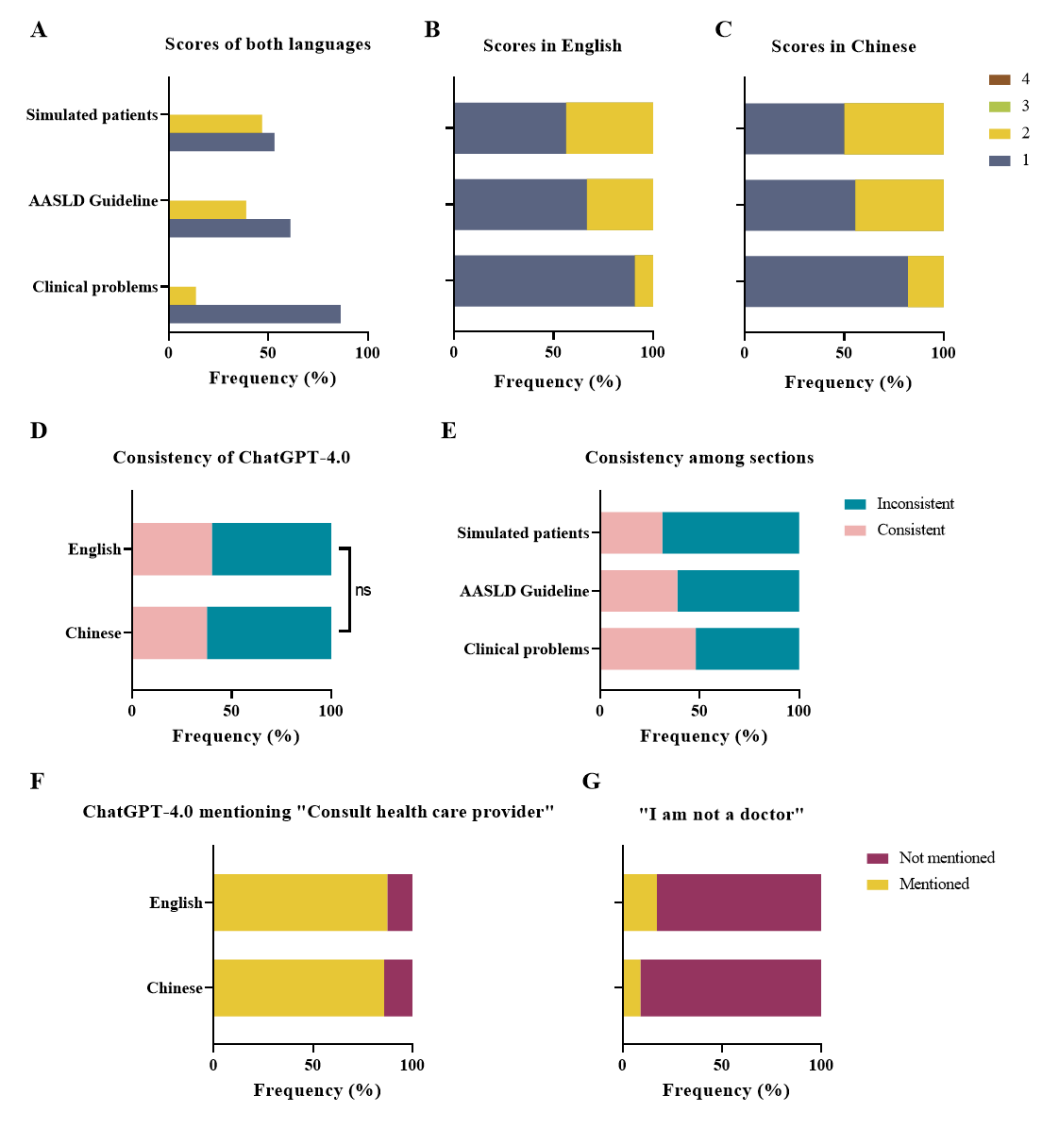
Results of the parallel assessment of ChatGPT-4.0 in sections involving clinical practice. (A) Comparison of the percentage of each grade across all responses in distinct question sections. (B) Percentage of each grade of responses in English in separated question sections. (C) Percentage of each grade of responses in Chinese in separated question sections. (D) Comparison of content consistency between responses in different working languages. (E) Examination of content consistency in different sections of questions. (F) Percentage of responses that include the recommendation to “consult health care providers or doctors.” (G) Percentage of responses containing the disclaimer phrase “I am not a doctor” or “I cannot give diagnosis or treatment.” AASLD: American Association for the Study of Liver Disease; ns: not significant.

The interobserver reliability κ was 0.6896 (*P*<.001) for information accuracy assessment. Notably, ChatGPT-4.0 demonstrated distinct performance compared to ChatGPT-3.5. The scores of ChatGPT-4.0 are presented in Figures 5A-C. Across the 3 sections of “Clinical Problem Questions,” “AASLD Guideline Questions,” and “Simulated Patient Questions,” the percentage of responses assessed as complete and comprehensive, as well as “grade 1,” was higher for ChatGPT-4.0 than for ChatGPT-3.5 (172/222, 77.5% vs 132/222, 59.5%), with a significant difference (*P*<.001). Furthermore, variations in grades were observed across the sections (*P*<.001). The “Clinical Problem Questions” section exhibited the highest percentage of responses assessed as complete and comprehensive for ChatGPT-4.0 (133/154, 86.4%), surpassing that for ChatGPT-3.5 (108/154, 70.1%; *P*<.001). Importantly, no responses from ChatGPT-4.0 were assessed as “a mix of correct and incorrect details” and “wholly incorrect or irrelevant information.” In general, ChatGPT-4.0 demonstrated superior information accuracy compared to ChatGPT-3.5. Moreover, ChatGPT-4.0 showed improved performance in responding to Chinese questions. Although there was a slightly lower percentage of responses assessed as “grade 1” for Chinese (82/112, 73.2%) than for English (90/110, 81.8%), the difference in performance between the languages was not significant (*P*=.13). The evaluation tables are presented in Multimedia Appendix 9 and 10.

The interobserver reliability κ was 0.6052 (*P*<.001) for content consistency of the repeated response assessment. ChatGPT-4.0 showed poor consistency in responses to repeated questions. Across all questions, ChatGPT-4.0 provided 44.1% (49/111 pairs of responses) stable repeated responses, and this proportion was lower than that for ChatGPT-3.5 (58/111, 52.3%). However, the difference was not significant (*P*=.23). Specifically, ChatGPT-4.0’s stability percentage was 38% (21/56 pairs of responses) in Chinese and 51% (28/55 pairs of responses) in English (Figure 5D), with no significant difference (*P*=.16). Among all the sections, responses in the “Clinical Problem Questions” section exhibited the highest rate of consistency at 48% (37/77 pairs of responses; Figure 5E). The difference in consistency across sections was not significant (*P*=.42). Detailed evaluation tables are provided in Multimedia Appendix 9 and 11.

Regarding responses to questions within the “Clinical Problem Questions” and “Simulated Patient Questions” sections, ChatGPT-4.0’s responses were assessed to provide sufficient emotional management support 8.1% (15/186) of the time (Table 3). This performance differed significantly from that of ChatGPT-3.5 (*P*=.04). The percentage was similar between Chinese and English (7/94, 7% and 8/92, 9%, respectively; *P*=.76). No response was assessed as “disrespectful or negative emotional guidance.” ChatGPT-4.0 showed similar performance between the 2 sections (10/154, 6.5% for Clinical Problem Questions and 5/32, 15.6% for Simulated Patient Questions assessed as grade 1; *P*=.08). However, among all responses assessed as “unstable,” there was no significant difference between the scores of response 1 and response 2 (*P*=.06). All the responses assessed as “sufficient emotional and psychological management guidance” are listed in Multimedia Appendix 12.

**Table 3:** Results of the emotional management guidance assessment of ChatGPT-4.0.

Language	Clinical Problem Questions (n=154), n				Simulated Patient Questions (n=32), n				Total (N=186), n				*P* value
	Grade 1	Grade 2	Grade 3	Grade 1		Grade 2	Grade 3	Grade 1		Grade 2	Grade 3		
										
Chinese	5	73	0	2		14	0	7		87	0	.08^a^	
English	5	71	0	3		13	0	8		84	0		
Total	10	144	0	5		27	0	15		171	0	.75^b^	

^a^*P* value across the grades of each section.

^b^*P* value between the grades of different working languages.

As shown in Figure 5F, ChatGPT-4.0 demonstrated comparable performance to ChatGPT-3.5 across all responses, with 86.5% (192/222) of responses emphasizing the importance of seeking medical assistance. In Chinese, 96 out of 112 responses (85.7%) stressed this need, while in English, 96 out of 110 responses (87.3%) did the same. Notably, no significant difference was observed between the languages (*P*=.73). In responses from ChatGPT-3.5 in the sections “Clinical Problem Questions,” “AASLD Guideline Questions,” and “Simulated Patient Questions,” the total percentage of responses with a medical service recommendation was 81.5% (181/222), which was not different from that for ChatGPT-4.0 (*P*=.15). All responses emphasizing the necessity of seeking medical services are listed in Multimedia Appendix 13.

Figure 5G illustrates that among all responses to questions involving clinical practice, ChatGPT-4.0 used the phrase “I am not a doctor” or “As a language model, I cannot give diagnosis or treatment…” with a probability of 13.1% (29/222). This percentage did not significantly differ from that of ChatGPT-3.5 (*P*=.46). In Chinese, the probability was 8.9% (10/112), while in English, the probability was 17.3% (19/110; Figure 5B). No significant difference was observed between the 2 languages (*P*=.07). These responses are detailed in Multimedia Appendix 14.

### Assessment of Responses to Closed Questions Across ChatGPT-4.0 and ChatGPT-3.5

**Figure 6:**
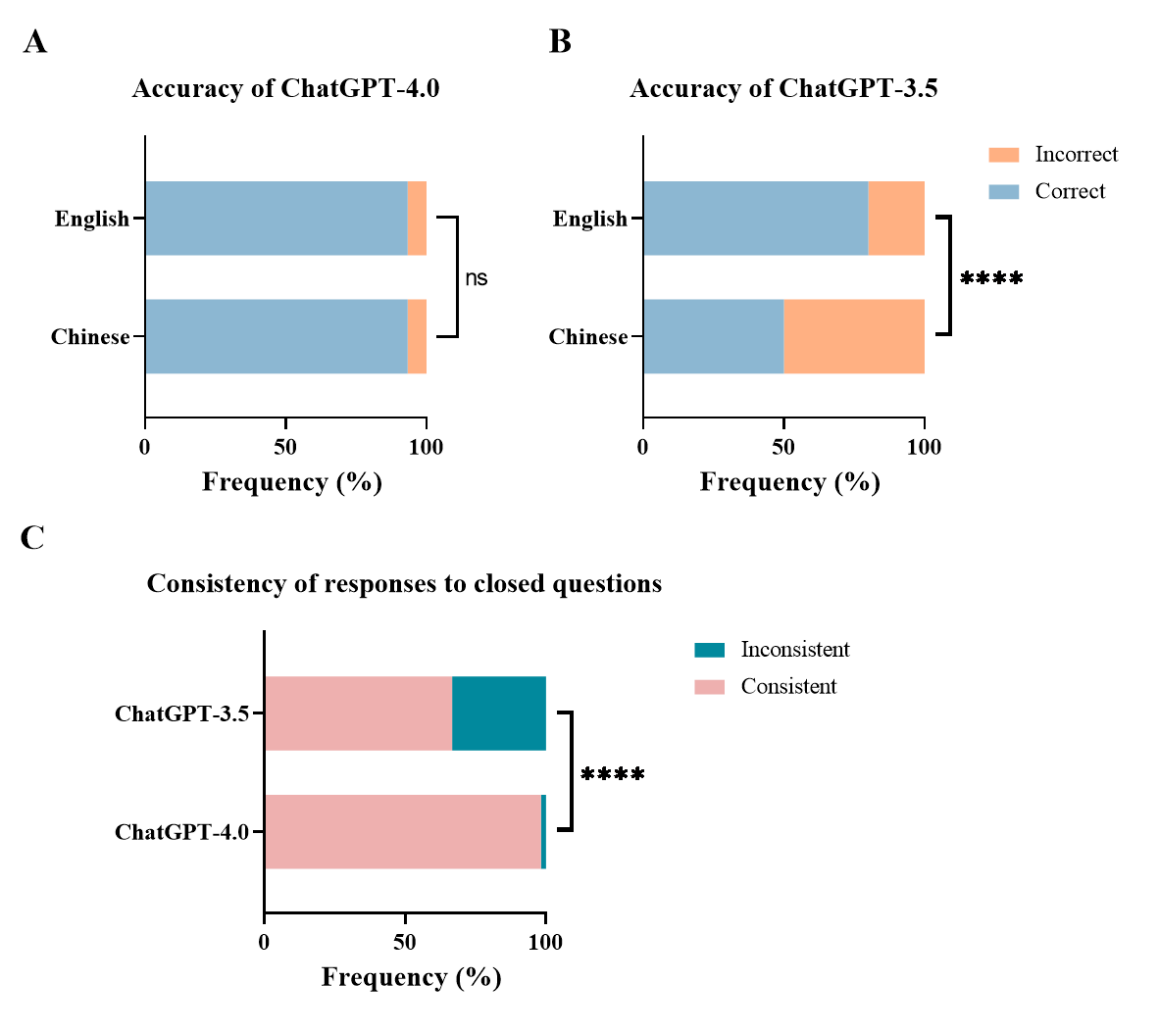
Results of responses to true-or-false questions. (A) Rate of accuracy of ChatGPT-4.0. (B) Rate of accuracy of ChatGPT-3.5. (C) Comparison of the consistency of responses to true-or-false questions between ChatGPT-4.0 and ChatGPT-3.5. ns: not significant. ****P<.0001.

When assessing the accuracy of statements derived from the AASLD guidelines for the treatment of CHB, ChatGPT-4.0 exhibited significantly superior performance compared to ChatGPT-3.5 (Figures 6A and B). ChatGPT-4.0 achieved a correctness percentage of 93.3% (168/180), with the same percentage accuracy in both Chinese and English (93.3% for each language). Conversely, ChatGPT-3.5 yielded an overall accuracy of 65.0% (117/180), with a split of 50.0% (45/90) in Chinese and 80.0% (72/90) in English.

Furthermore, ChatGPT-4.0 displayed enhanced consistency in repeated responses (Figure 6C). Stable responses accounted for 98.3% (59/60) of responses in ChatGPT-4.0, whereas ChatGPT-3.5 provided only 66.7% (40/60) stable responses. The difference in response stability between the models was statistically significant (*P*<.001). Details are provided in Multimedia Appendix 15.

## Discussion

### ChatGPT-3.5 Working as a Medical Consulting Assistant

Our evaluation highlighted the proficiency of ChatGPT-3.5 as a medical consultation assistant. ChatGPT-3.5 provided predominantly accurate information, but there was a notable limitation in the comprehensiveness of the responses, indicating a need for targeted medical professional input. Continuous enhancement of LLMs may contribute to more specific and reliable guidance. Despite its strengths, ChatGPT-3.5 displayed limitations in emotional management support, a crucial aspect of chronic disease management [30]. Facilitating emotional modulation is integral to fostering patient willingness for self-management and treatment compliance [7,30].

Therefore, it is imperative to consider emotional cognition and regulation in medical diagnosis and treatment. Our study suggested that the potential for ChatGPT to serve as an emotional management assistant for chronic patients warrants further study, with related localized training considered if LLMs are to be employed in clinical practice as health consultation assistants.

### Impact of Working Language on Performance

By revealing ChatGPT’s inferior performance in Chinese compared to English, the study emphasized the influence of the choice of working language on stability and correctness. ChatGPT-3.5 showed worse performance on information accuracy in Chinese, implying the insufficient input of knowledgeable materials in Chinese. Both ChatGPT-3.5 and ChatGPT-4.0 showed less stability in Chinese, which was reflected in a lower consistency rate of responses to the same questions. This challenge stemmed from variations in language resources during the model’s original training, primarily centered around English-based medical guidelines. Though there are Chinese versions of these guidelines, the timeliness and accuracy of Chinese materials are limited. To enhance ChatGPT’s efficacy in diverse language environments, the model should undergo additional training based on data sourced from specific language resources. This targeted training should focus on potential misunderstandings related to terms and phrasings in local languages, thereby addressing language-specific nuances and enhancing overall performance. Notably, ChatGPT-3.5 exhibited language-specific mistakes, with Chinese responses showing errors related to misunderstanding or incorrect usage of terms. This underscores the importance of targeted language training for LLMs to minimize inaccuracies, especially in medical contexts.

### Cautionary Statements and Patient-Oriented Usage

In discussions related to diagnosis and therapy, ChatGPT-3.5 consistently emphasized the importance of consulting a health care provider, indicating a cautious approach. Owing to constraints in both timeliness and accuracy inherent in language models, ChatGPT-3.5 occasionally emphasized its nondoctor status, thus refraining from providing direct diagnosis or therapy in the conversation. However, such statements may imply the unreliability of the medical judgment, especially in a Chinese cultural context. Thus, further inquiries are warranted to evaluate the potential risks and benefits of this response mode, considering its impact on patient trust and compliance challenges.

### Implications for Future Development in Clinical Medicine

As AI, including LLMs, is being progressively integrated into clinical medicine, understanding the advantages and disadvantages is paramount. While ChatGPT demonstrates promise as a medical consulting assistant for CHB patients, future research and development should prioritize targeted language input and emotional management training. Besides, establishing and updating prompts, which are in a specific order, and templates based on which LLMs could provide responses in a standardized format would significantly enhance ChatGPT’s performance. Overcoming language barriers and addressing emotional support deficiencies will be crucial for maximizing the potential benefits of LLMs in medical assistance.

### Comparison of ChatGPT-4.0 to ChatGPT-3.5

ChatGPT-4.0 demonstrated superior performance compared with ChatGPT-3.5 in terms of information accuracy. This improvement aligns with the expected advancements in ChatGPT-4.0 as a more advanced iteration. However, ChatGPT-4.0 did not exhibit better response stability in open-ended questions. This could be attributed to a reduced ability to follow chain-of-thought prompting [31]. Despite this inconsistency, it did not affect the accuracy of information, suggesting that LLMs tend to employ diverse language patterns and content combinations.

In responses to closed questions (30 true-or-false statements based on the AASLD guidelines for the treatment of hepatitis B), ChatGPT-4.0 demonstrated a higher rate of accuracy and stability, indicating substantial improvement in the model’s understanding of hepatitis B medical knowledge as the model progressed.

The improvement of ChatGPT-4.0 in terms of information accuracy suggested the tremendous benefit of the model update, but the deficiency in emotional management remained. Therefore, additional training related to emotional management guidance and humanistic care is essential for the preparation of the model before application.

Notably, in responses to open questions, ChatGPT-4.0 displayed interesting changes compared to ChatGPT-3.5. ChatGPT-4.0 included reference information in 5 of the responses, all of which were verified to be accurate. This suggests an enhancement in the format and reliability of ChatGPT-4.0. However, the impact of such changes on the patient experience warrants further exploration. Additionally, ChatGPT-4.0 was more likely to use a direct disclaimer like “I am an AI model...” or “I’m not a doctor...” and even “Disclaimer: I’m not a doctor...,” indicating a more stringent approach. However, the increase in possibility was too subtle to be considered significant.

### Comparison to Prior Work

Numerous studies have explored the potential application of ChatGPT in clinical practice. Ayers et al [32] observed that ChatGPT tends to deliver longer and more empathetic responses of higher quality compared to real doctors. In a study by Cascella et al [12], ChatGPT demonstrated proficiency in composing medical notes for intensive care unit patients and scientific writing, despite lacking medical expertise. The researchers highlighted the model’s effectiveness in providing medical advice and its potential in patient communication [12]. Several studies have evaluated ChatGPT’s responses in various medical specialties [14,33-35]. In contrast, our study uniquely focused on ChatGPT’s cross-language performance in clinical counseling, revealing that language choice impacts accuracy and answer stability. This emphasizes the importance of language selection for the practical application of LLMs.

### Limitations

It is important to acknowledge certain limitations in our study. The evaluation did not comprehensively assess ChatGPT’s knowledge and ability in guiding emotional management for patients due to questionnaire resource constraints. The lack of a standardized questionnaire also limited the reliability of the questionnaire used owing to the lack of a related interrater reliability measure. Meanwhile, as the first work in the assessment of medical consulting AI systems for hepatitis B, it was difficult to estimate the possible effects of vagueness, ambiguity, and misunderstanding associated with grammatical mistakes or vagueness of the questions. The researchers revised the questions to address such concerns, which created new concerns about discrepancies between these “standard” questions and practical application scenarios. These problems should be addressed in future research. Additionally, while cautionary statements promote responsible usage, the potential risks and benefits of this approach require further exploration. Future studies should address these limitations for a more comprehensive understanding of ChatGPT’s application in medical assistance.

### Conclusion

ChatGPT-3.5 exhibits promising capabilities as a medical consultation assistant, providing accurate yet occasionally less comprehensive information. ChatGPT-4.0, which is an improved version of the model, showed stronger application potential than ChatGPT-3.5. Recognizing their limitations in emotional support and language-specific performance, future developments should prioritize targeted language training and enhanced emotional management features. While cautionary statements underscore responsible usage, the model’s potential in aiding patients with CHB is evident. As AI continues to shape the medical practice, refining LLMs for nuanced health care contexts is imperative. Striking a balance among linguistic accuracy, emotional sensitivity, and ethical patient engagement remains important for successful integration into clinical settings.

## Appendix

Multimedia Appendix 1 Examples of questions revised or eliminated.

Multimedia Appendix 2 Summary of the information accuracy grades and consistency of ChatGPT-3.5.

Multimedia Appendix 3 Assessment of the information accuracy of ChatGPT-3.5.

Multimedia Appendix 4 Results of mistake type evaluation of ChatGPT-3.5.

Multimedia Appendix 5 Content consistency assessment of ChatGPT-3.5.

Multimedia Appendix 6 Responses provided with sufficient emotional management guidance in ChatGPT-3.5.

Multimedia Appendix 7 Evaluation of whether responses mentioned consulting health care providers or doctors in ChatGPT-3.5.

Multimedia Appendix 8 Responses mentioning “I am not a doctor” or “I cannot give diagnosis or treatment” in ChatGPT-3.5.

Multimedia Appendix 9 Summary of the information accuracy grades and consistency of ChatGPT-4.0.

Multimedia Appendix 10 Assessment of the information accuracy of ChatGPT-4.0.

Multimedia Appendix 11 Content consistency assessment of ChatGPT-4.0.

Multimedia Appendix 12 Responses provided with sufficient emotional management guidance in ChatGPT-4.0.

Multimedia Appendix 13 Evaluation of whether responses mentioned consulting health care providers or doctors in ChatGPT-4.0.

Multimedia Appendix 14 Responses mentioning “I am not a doctor” or “I cannot give diagnosis or treatment” in ChatGPT-4.0.

Multimedia Appendix 15 Results of responses to closed questions (true-or-false) in ChatGPT-3.5 and ChatGPT-4.0.

## Abbreviations

AASLD: American Association for the Study of Liver Disease
AI: artificial intelligence
ALT: alanine transaminase
CHB: chronic hepatitis B
EASL: European Association for the Study of the Liver
HBV: hepatitis B virus
LLM: large language model
WHO: World Health Organization

## References

[1] Hepatitis B. World Health Organization.

[2] Global hepatitis report, 2017. World Health Organization.

[3] Han S, Tran TT (2015). Management of Chronic Hepatitis B: An Overview of Practice Guidelines for Primary Care Providers. J Am Board Fam Med.

[4] European Association for the Study of the Liver. Electronic address: easloffice@easloffice.eu, European Association for the Study of the Liver (2017). EASL 2017 Clinical Practice Guidelines on the management of hepatitis B virus infection. J Hepatol.

[5] Degasperi E, Anolli MP, Lampertico P (2022). Towards a Functional Cure for Hepatitis B Virus: A 2022 Update on New Antiviral Strategies. Viruses.

[6] Appleton AA, Buka SL, Loucks EB, Gilman SE, Kubzansky LD (2013). Divergent associations of adaptive and maladaptive emotion regulation strategies with inflammation. Health Psychol.

[7] de Ridder D, Geenen R, Kuijer R, van Middendorp H (2008). Psychological adjustment to chronic disease. The Lancet.

[8] Wani SUD, Khan NA, Thakur G, Gautam SP, Ali M, Alam P, Alshehri S, Ghoneim MM, Shakeel F (2022). Utilization of Artificial Intelligence in Disease Prevention: Diagnosis, Treatment, and Implications for the Healthcare Workforce. Healthcare (Basel).

[9] Haug CJ, Drazen JM (2023). Artificial Intelligence and Machine Learning in Clinical Medicine, 2023. N Engl J Med.

[10] Au K, Yang W (2023). Auxiliary use of ChatGPT in surgical diagnosis and treatment. Int J Surg.

[11] van Dis EAM, Bollen J, Zuidema W, van Rooij R, Bockting CL (2023). ChatGPT: five priorities for research. Nature.

[12] Cascella M, Montomoli J, Bellini V, Bignami E (2023). Evaluating the Feasibility of ChatGPT in Healthcare: An Analysis of Multiple Clinical and Research Scenarios. J Med Syst.

[13] Gilson A, Safranek CW, Huang T, Socrates V, Chi L, Taylor RA, Chartash D (2023). How Does ChatGPT Perform on the United States Medical Licensing Examination (USMLE)? The Implications of Large Language Models for Medical Education and Knowledge Assessment. JMIR Med Educ.

[14] Yeo YH, Samaan JS, Ng WH, Ting P, Trivedi H, Vipani A, Ayoub W, Yang JD, Liran O, Spiegel B, Kuo A (2023). Assessing the performance of ChatGPT in answering questions regarding cirrhosis and hepatocellular carcinoma. Clin Mol Hepatol.

[15] Liu J, Wang C, Liu S (2023). Utility of ChatGPT in Clinical Practice. J Med Internet Res.

[16] Deng L, Wang T, Zhai Z, Tao W, Li J, Zhao Y, Luo S, Xu J (2024). Evaluation of large language models in breast cancer clinical scenarios: a comparative analysis based on ChatGPT-3.5, ChatGPT-4.0, and Claude2. Int J Surg.

[17] Frosolini A, Franz L, Benedetti S, Vaira LA, de Filippis C, Gennaro P, Marioni G, Gabriele G (2023). Assessing the accuracy of ChatGPT references in head and neck and ENT disciplines. Eur Arch Otorhinolaryngol.

[18] Lee T, Rao AK, Campbell DJ, Radfar N, Dayal M, Khrais A (2024). Evaluating ChatGPT-3.5 and ChatGPT-4.0 Responses on Hyperlipidemia for Patient Education. Cureus.

[19] Zheng H, Zhang G, Chan P, Wang F, Rodewald LE, Miao N, Sun X, Yin Z, Edwards J, Wang H (2019). Compliance among infants exposed to hepatitis B virus in a post-vaccination serological testing program in four provinces in China. Infect Dis Poverty.

[20] Wang M, Chen EQ (2022). [Impact of treatment compliance in chronic hepatitis B]. Zhonghua Gan Zang Bing Za Zhi.

[21] Zhou X, Zhang F, Ao Y, Lu C, Li T, Xu X, Zeng H (2021). Diagnosis experiences from 50 hepatitis B patients in Chongqing, China: a qualitative study. BMC Public Health.

[22] Tütüncü EE, Güner R, Gürbüz Y, Kaya Kalem A, Öztürk B, Hasanoğlu İ, Şencan İ, Taşyaran MA (2017). Adherence to Nucleoside/Nucleotide Analogue Treatment in Patients with Chronic Hepatitis B. Balkan Med J.

[23] Ozyigitoglu D, Sevgi DY, Tahtasakal CA, Oncul A, Gunduz A, Dokmetas I (2022). Adherence to Treatment with Oral Nucleoside/Nucleotide Analogs in Patients with Chronic Hepatitis B. Sisli Etfal Hastan Tip Bul.

[24] Jiang H, Zhao Q, Chen K, Yang J, Li Q (2022). The main features of physician assistants/associates and insights for the development of similar professions in China. J Evid Based Med.

[25] Yu Q, Yin W, Huang D, Sun K, Chen Z, Guo H, Wu D (2021). Trend and equity of general practitioners' allocation in China based on the data from 2012-2017. Hum Resour Health.

[26] Wen J, Cheng Y, Hu X, Yuan P, Hao T, Shi Y (2016). Workload, burnout, and medical mistakes among physicians in China: A cross-sectional study. Biosci Trends.

[27] Wei L, Jia J, Weng X, Dou X, Jiang J, Tang H, Ning Q, Dai Q, Li R, Liu J (2016). Treating chronic hepatitis B virus: Chinese physicians' awareness of the 2010 guidelines. World J Hepatol.

[28] Yu P, Fang C, Liu X, Fu W, Ling J, Yan Z, Jiang Y, Cao Z, Wu M, Chen Z, Zhu W, Zhang Y, Abudukeremu A, Wang Y, Liu X, Wang J (2024). Performance of ChatGPT on the Chinese Postgraduate Examination for Clinical Medicine: Survey Study. JMIR Med Educ.

[29] Brown TB, Mann B, Ryder N, Subbiah M, Kaplan J, Dhariwal P, Neelakantan A Language Models are Few-Shot Learners. arXiv.

[30] Wierenga KL, Lehto RH, Given B (2017). Emotion Regulation in Chronic Disease Populations: An Integrative Review. Res Theory Nurs Pract.

[31] Chen L, Zaharia M, Zou J How is ChatGPT's behavior changing over time?. arXiv.

[32] Ayers JW, Poliak A, Dredze M, Leas EC, Zhu Z, Kelley JB, Faix DJ, Goodman AM, Longhurst CA, Hogarth M, Smith DM (2023). Comparing Physician and Artificial Intelligence Chatbot Responses to Patient Questions Posted to a Public Social Media Forum. JAMA Intern Med.

[33] Grünebaum A, Chervenak J, Pollet SL, Katz A, Chervenak FA (2023). The exciting potential for ChatGPT in obstetrics and gynecology. Am J Obstet Gynecol.

[34] Fournier A, Fallet C, Sadeghipour F, Perrottet N (2024). Assessing the applicability and appropriateness of ChatGPT in answering clinical pharmacy questions. Ann Pharm Fr.

[35] Antaki F, Touma S, Milad D, El-Khoury J, Duval R (2023). Evaluating the Performance of ChatGPT in Ophthalmology: An Analysis of Its Successes and Shortcomings. Ophthalmol Sci.

