# Supplementary material for: Assessing ChatGPT as a Medical Consultation Assistant for Chronic Hepatitis B: Cross-Language Study of English and Chinese
Source: JMIR Med Inform. 2024 Aug 8;12:e56426. doi: 10.2196/56426 (PMC11342014; doi:10.2196/56426)
Supplement: Multimedia Appendix 1 [file medinform_v12i1e56426_app1.docx]

**Multimedia Appendix 1.** Examples of questions revised or eliminated.

| Language | Questions | Classification and Polishing |
| --- | --- | --- |
| English | HbSag and antiHCV negative enough for Hep B and C elimination? | Vague questions  (Eliminated) |
| English | Just this year when i went to doctor for bloodtest, the result was Hepatitis B Reactive. If it become severe? What will be the symptoms?Can i still live longer even though i have Hepa B? Can i still have babies? | Mispunctuation /  Multiple questions  (Separated) |
| English | Hepatits B reactive, what i do stop drugs? doctors? | Grammar mistake (Mended) |
| English | recently got the second dose of Covid-19 vaccine and i am going on a vacation to Thailand in 1,5 weeks. I have not had the hepatitis vaccine before and i was wondering if it is safe for me to take the hepatits vaccine 7 days after my second dose of covid-19 vaccine. | Repeated questions (Only one kept) |
| English | Can I get Hepatitis B vaccine a week after COVID-19 vaccine? |  |
| Chinese | 打完新冠疫苗还能打乙肝疫苗吗？ |  |
| Chinese | 能治好吗，乙肝几年了 | Grammar mistake/  Mispunctuation /  Ambiguity (Mended) |
| Chinese | 乙肝大三阳，在吃药，相亲被嫌弃怎么办？ | Non-medical Issues |
