# Supplementary material for: Assessing ChatGPT as a Medical Consultation Assistant for Chronic Hepatitis B: Cross-Language Study of English and Chinese
Source: JMIR Med Inform. 2024 Aug 8;12:e56426. doi: 10.2196/56426 (PMC11342014; doi:10.2196/56426)
Supplement: Multimedia Appendix 2 [file medinform_v12i1e56426_app2.docx]

**Multimedia Appendix 2.** Summary of the information accuracy grades and consistency of ChatGPT-3.5.

|  |  | Chinese | | | | | | English | | | | | | Total | | | | | |
| --- | --- | --- | --- | --- | --- | --- | --- | --- | --- | --- | --- | --- | --- | --- | --- | --- | --- | --- | --- |
| Assessment Content | Grades | Term Explanation | Short Answer | Clinical Problems | AASLD Guideline | Simulated Patients | Total | Term Explanation | Short Answer | Clinical Problems | AASLD Guideline | Simulated Patients | Total | Term Explanation | Short Answer | Clinical Problems | AASLD Guideline | Simulated Patients | Total |
|  |  |  |  |  |  |  |  |  |  |  |  |  |  |  |  |  |  |  |  |
| Information Accuracy | 1 | 30 (88.24%) | 20 (45.45%) | 47 (60.26%) | 1 (5.56%) | 5 (31.25%) | 103 (54.21%) | 26 (92.86%) | 20 (47.62%) | 61 (80.26%) | 11 (61.11%) | 7 (43.75%) | 125 (69.44%) | 56 (90.32%) | 40 (46.51%) | 108 (70.13%) | 12 (33.33%) | 12 (37.50%) | 228 (61.62%) |
|  | 2 | 4 (11.76%) | 19 (43.18%) | 24 (30.77%) | 7 (38.89%) | 9 (56.25%) | 63 (33.16%) | 2 (7.14%) | 19 (45.24%) | 12 (15.79%) | 3 (16.67%) | 9 (56.25%) | 45 (25.00%) | 6 (9.68%) | 38 (44.19%) | 36 (23.38%) | 10 (27.78%) | 18 (56.25%) | 108 (29.19%) |
|  | 3 | 0 (0.00%) | 5 (11.36%) | 7 (8.97%) | 4 (22.22%) | 2 (12.50%) | 18 (9.47%) | 0 (0.00%) | 3 (7.14%) | 3 (3.95%) | 3 (16.67%) | 0 (0.00%) | 9 (5.00%) | 0 (0.00%) | 8 (9.30%) | 10 (6.49%) | 7 (19.44%) | 2 (6.25%) | 27 (7.30%) |
|  | 4 | 0 (0.00%) | 0 (0.00%) | 0 (0.00%) | 6 (33.33%) | 0 (0.00%) | 6 (3.16%) | 0 (0.00%) | 0 (0.00%) | 0 (0.00%) | 1 (5.56%) | 0 (0.00%) | 1 (0.56%) | 0 (0.00%) | 0 (0.00%) | 0 (0.00%) | 7 (19.44%) | 0 (0.00%) | 7 (1.89%) |
|  | Total of Grades | 34 | 44 | 78 | 18 | 16 | 190 | 28 | 42 | 76 | 18 | 16 | 180 | 62 | 86 | 154 | 36 | 32 | 370 |
|  | P | 0.0013^a^ | | | | | | | | | | | | 0.5434^b^ | 0.6235 | 0.0337 | 0.0022 | 0.3268 | <0.0001^c^ |
| Consistency | Consistent  responses | 16 (94.12%) | 5 (22.73%) | 18 (46.15%) | 3 (33.33%) | 2 (25.00.00%) | 44 (46.32%) | 13 (94.12%) | 8 (94.12%) | 27 (71.05%) | 5 (55.56%) | 3 (37.50.00%) | 56 (62.22%) | 29 (93.55%) | 13 (30.23%) | 45 (58.44%) | 8 (44.44%) | 5 (31.25%) | 100 (54.05%) |
|  | Inconsistent  responses | 1 | 17 | 21 | 6 | 6 | 51 | 1 | 13 | 11 | 4 | 5 | 34 | 2 | 30 | 32 | 10 | 11 | 85 |
|  | Total responses | 17 | 22 | 39 | 9 | 8 | 95 | 14 | 21 | 38 | 9 | 8 | 90 | 31 | 43 | 77 | 18 | 16 | 185 |
|  | P | 0.0387^d^ | | | | | | | | | | | | >0.9999^e^ | 0.3319 | 0.0375 | 0.6372 | >0.9999 | <0.0001^f^ |

^a^Indicating the P value of the difference between grades in the two languages.

^b^Indicating the P value of the different grades between the two languages in the section Term Explanation.

^c^Indicating the P value of the difference in grades across the sections.

^d^Indicating the P value of the difference between consistency in the two languages.

^e^Indicating the P value of the different consistency between the two languages in the section Term Explanation.

^f^Indicating the P value of the difference in consistency across the sections.
