# Supplementary material for: Assessing ChatGPT as a Medical Consultation Assistant for Chronic Hepatitis B: Cross-Language Study of English and Chinese
Source: JMIR Med Inform. 2024 Aug 8;12:e56426. doi: 10.2196/56426 (PMC11342014; doi:10.2196/56426)
Supplement: Multimedia Appendix 4 [file medinform_v12i1e56426_app4.docx]

**Multimedia Appendix 4.** Results of mistake type evaluation of ChatGPT-3.5.

| Number | Classicfication | Question | Response1 | Response2 | Grade | Type |
| --- | --- | --- | --- | --- | --- | --- |
| 21 | Short Answer Questions | List treatment methods for hepatitis B | The treatment for hepatitis B depends on whether the infection is acute or chronic, and the severity of the disease. Here are some common methods for treating hepatitis B: 1.Antiviral medications: Antiviral medications can help reduce the amount of virus in the body, slow down the progression of the disease, and prevent liver damage. These medications include entecavir, tenofovir, lamivudine, and adefovir. 2.Interferon therapy: Interferon therapy is a type of treatment that involves injecting interferon into the body to boost the immune system and fight the virus. It is usually used to treat chronic hepatitis B and has some side effects. 3.Liver transplant: In severe cases of hepatitis B, liver transplantation may be necessary if the liver is severely damaged. 4.Lifestyle changes: Patients with hepatitis B should avoid alcohol, maintain a healthy diet, and get regular exercise. They should also avoid certain medications and supplements that may be harmful to the liver. 5.Vaccination: Vaccination is the best way to prevent hepatitis B infection, and it is recommended for everyone, especially those at high risk of contracting the virus. It is important to consult a healthcare provider for the appropriate treatment plan for hepatitis B as they can provide tailored recommendations based on the patient's individual needs and medical history. | / | 3,1 | C |
| 34 | Short Answer Questions | Who should not receive the hepatitis B vaccine? | The hepatitis B vaccine is generally safe and well-tolerated, and most people can receive the vaccine without any problems. However, there are some situations where the vaccine may not be recommended or should be delayed. Here are some groups of people who may not be able to receive the hepatitis B vaccine: 1.People who have had a severe allergic reaction to a previous dose of the hepatitis B vaccine or any component of the vaccine. 2.People who are currently ill or have a fever. In general, vaccination should be delayed until the person has recovered. 3.Infants who are born weighing less than 2,000 grams. These infants may receive a different schedule of the hepatitis B vaccine. 4.People who are allergic to yeast, as the hepatitis B vaccine is made using yeast. 5.Pregnant women. While the hepatitis B vaccine is generally considered safe during pregnancy, it is not routinely recommended unless the woman is at high risk of HBV infection. It is important to consult a healthcare provider before receiving the hepatitis B vaccine to discuss any concerns or medical conditions that may affect vaccine eligibility. In general, the hepatitis B vaccine is safe and highly effective at preventing HBV infection and its complications, including liver cancer and cirrhosis. | / | 3,2 | C |
| 39 | Short Answer Questions | Symptoms of early cirrhosis | Cirrhosis is a late-stage liver disease that can develop over several years in people with chronic hepatitis B infection. In the early stages of cirrhosis, there may not be any noticeable symptoms. However, as the disease progresses, the liver becomes increasingly damaged and scarred, which can lead to the following symptoms: 1.Fatigue: Feeling tired or weak, even after getting enough rest, is a common symptom of early cirrhosis. 2.Loss of appetite: People with early cirrhosis may experience a loss of appetite, nausea, and vomiting. 3.Abdominal pain and bloating: As the liver becomes more damaged, it can cause abdominal pain and bloating. 4.Swelling: Swelling in the legs, ankles, and feet, or swelling in the abdomen, may occur due to a buildup of fluids in the body. 5.Jaundice: Jaundice is a condition where the skin and eyes become yellowish due to a buildup of bilirubin in the blood. It can be a sign of liver damage. 6.Itching: Itching, or pruritus, can be a symptom of liver damage and may be caused by a buildup of toxins in the blood. 7.Mental confusion: As cirrhosis progresses, it can lead to mental confusion, forgetfulness, and difficulty concentrating. It is important to consult a healthcare provider if you experience any of these symptoms, as early diagnosis and treatment can help slow or even reverse the progression of cirrhosis. People with chronic hepatitis B infection should undergo regular monitoring of liver function and virus levels to detect any signs of cirrhosis early. | / | 3,2 | A |
| 40 | Clinical Problems | How are HBV infectious, cirrhosis and hepatocellular carcinoma related? | / | HBV (Hepatitis B virus) is a viral infection that attacks the liver and can cause both acute and chronic hepatitis. The virus is transmitted through contact with infected blood, semen, and other body fluids. Chronic HBV infection can lead to liver cirrhosis, which is the scarring of the liver tissue that occurs over time due to ongoing liver damage. Cirrhosis can cause liver failure and can increase the risk of developing hepatocellular carcinoma (HCC), which is a type of liver cancer. HBV is a major risk factor for developing HCC. About 80% of HCC cases are associated with chronic HBV infection. Chronic infection causes damage to the liver, which increases the risk of HCC. The risk of developing HCC is highest in people with both HBV infection and cirrhosis. Overall, HBV infection can lead to liver damage, cirrhosis, and HCC. It is important to get vaccinated against HBV and to take precautions to prevent its transmission, such as practicing safe sex, not sharing needles or other injection equipment, and getting screened for HBV if you are at risk. Early detection and treatment of HBV infection can help reduce the risk of developing cirrhosis and HCC. | 1,3 | C |
| 47 | Clinical Problems | Give advice for a patient who's HBeAb is positive | HBeAb (hepatitis B e antibody) positivity indicates that a person has been exposed to hepatitis B virus (HBV) in the past and has developed an immune response to the e antigen of the virus. This may indicate a resolved infection, or it could be a result of vaccination against hepatitis B. If a patient's HBeAb test is positive, but their hepatitis B surface antigen (HBsAg) and HBV DNA tests are negative, then it suggests that the patient has been previously infected with HBV but has cleared the virus and is no longer infectious. In this case, no specific treatment is necessary, but the patient should be monitored periodically to ensure that the virus does not reactivate. However, if a patient is positive for both HBeAb and HBsAg, it suggests that they have chronic hepatitis B infection. In this case, the patient should be evaluated by a hepatologist or infectious disease specialist to determine the appropriate treatment regimen. The goal of treatment for chronic hepatitis B is to suppress viral replication and prevent progression of liver disease. The treatment options may include antiviral medications such as tenofovir or entecavir, which are effective at suppressing viral replication and reducing the risk of liver damage. These medications are usually prescribed for a prolonged period, and the patient will need to be monitored regularly with blood tests to assess their response to treatment and liver function. In addition to medication, lifestyle modifications such as avoiding alcohol and maintaining a healthy diet may also be recommended to help improve liver health. Vaccination against hepatitis A and B may also be recommended for patients with chronic hepatitis B to protect against co-infection with other types of viral hepatitis. It is important for patients with chronic hepatitis B to follow their treatment plan and attend regular follow-up appointments to monitor their condition and ensure that they are responding to treatment. | / | 3,1 | C |
| 68 | Clinical Problems | I was just told to be Hepatitis B Reactive. If it become severe, what will be the symptoms? | / | Hepatitis B is a viral infection that can cause inflammation of the liver, and it is spread through contact with blood or other body fluids of an infected person. The severity of symptoms can vary depending on the individual and the stage of the infection. In some cases, individuals with Hepatitis B may not experience any symptoms at all, while in others, symptoms may range from mild to severe. Some common symptoms of Hepatitis B include: 1. Fatigue 2. Loss of appetite 3. Nausea and vomiting 4. Abdominal pain 5. Jaundice (yellowing of the skin and eyes) 6. Dark urine 7. Clay-colored stools 8. In severe cases of Hepatitis B, individuals may experience symptoms such as: 9. Bleeding and bruising easily 10. Swollen abdomen due to fluid accumulation 11. Confusion or cognitive impairment 12. Severe liver damage (cirrhosis) 13. Liver failure If you have been diagnosed with Hepatitis B, it is important to speak with your healthcare provider about your condition and any potential symptoms or complications that may arise. Treatment options are available that can help manage symptoms and reduce the risk of liver damage. | 1,3 | C |
| 82 | AASLD Guideline questions | Should antiviral therapy be discontinued in hepatitis B e antigen (HBeAg)-positive persons who have developed HBeAg seroconversion on therapy? | The decision to discontinue antiviral therapy in hepatitis B e antigen (HBeAg)-positive individuals who have achieved HBeAg seroconversion on therapy should be carefully evaluated on a case-by-case basis by a healthcare professional experienced in managing hepatitis B. HBeAg seroconversion is the loss of HBeAg with or without the appearance of hepatitis B e antibody (HBeAb) and is considered an important treatment endpoint in HBeAg-positive chronic hepatitis B. However, the decision to discontinue antiviral therapy after achieving HBeAg seroconversion depends on several factors, including the patient's age, liver function tests, HBV DNA levels, and the degree of fibrosis or cirrhosis. In general, the current guidelines recommend that antiviral therapy should be continued for at least 12 months after HBeAg seroconversion, and ideally, until HBsAg loss or seroconversion occurs. After discontinuing therapy, close monitoring with regular liver function tests, HBV DNA levels, and ultrasound is required to detect any potential reactivation of the virus or disease progression. If HBV DNA levels remain undetectable and liver function tests remain stable for an extended period, then it may be possible to consider stopping antiviral therapy. However, it is important to note that there is a high risk of disease reactivation and progression if antiviral therapy is stopped too early or without appropriate monitoring. Therefore, the decision to discontinue therapy should be made with caution, taking into account the individual patient's clinical and virological characteristics, as well as the potential risks and benefits of stopping therapy. | / | 3,2 | C |
| 85 | AASLD Guideline questions | Is there a benefit to adding a second antiviral agent in persons with persistent low levels of viremia while being treated with either tenofovir or entecavir? | The addition of a second antiviral agent may be considered in persons with persistent low levels of viremia while being treated with either tenofovir or entecavir, but the decision should be made on a case-by-case basis after careful consideration of the potential risks and benefits. In general, the goal of antiviral therapy for chronic hepatitis B is to achieve sustained suppression of viral replication, as this is associated with reduced risk of disease progression, including liver cirrhosis and hepatocellular carcinoma. However, some patients may have persistent low levels of viremia despite treatment with a first-line antiviral agent, such as tenofovir or entecavir. In this situation, the addition of a second antiviral agent may be considered to further reduce viral replication and potentially improve clinical outcomes. However, it is important to note that the use of multiple antiviral agents may increase the risk of adverse effects and drug interactions, and may also lead to the development of drug resistance. Therefore, the decision to add a second antiviral agent should be made after careful consideration of the individual patient's clinical status, viral load, and liver function, as well as potential drug interactions and the risk of developing drug resistance. Additionally, close monitoring is required to assess treatment response and potential adverse effects. | The use of combination therapy with two antiviral agents is a common strategy in the treatment of chronic viral infections, such as HIV and hepatitis B and C. However, the benefit of adding a second antiviral agent in persons with persistent low levels of viremia while being treated with either tenofovir or entecavir may depend on the specific context and individual case. For example, in the case of hepatitis B, guidelines from the American Association for the Study of Liver Diseases (AASLD) suggest that combination therapy with entecavir or tenofovir plus another antiviral agent (such as interferon-alpha or adefovir) may be considered in certain circumstances, such as when a patient has evidence of drug resistance or persistent low-level viremia despite treatment. However, the decision to add a second agent should be made on a case-by-case basis and should take into account a variety of factors, including the patient's overall health, the severity of their infection, and the potential risks and benefits of combination therapy. In addition, close monitoring of viral load and liver function is necessary to assess the effectiveness of treatment and to detect any potential side effects. Overall, the addition of a second antiviral agent may be beneficial in some cases, but it is important to consult with a healthcare provider and follow their guidance on the best course of treatment. | 4,3 | D,C |
| 86 | AASLD Guideline questions | Should persons with compensated cirrhosis and low levels of viremia be treated with antiviral agents? | The decision to treat individuals with compensated cirrhosis and low levels of viremia with antiviral agents depends on various factors and should be made by a qualified healthcare professional after considering the individual's medical history, current condition, and other relevant factors. In general, the goal of antiviral therapy in individuals with compensated cirrhosis and low levels of viremia is to reduce the risk of disease progression and prevent complications such as liver failure and hepatocellular carcinoma. The decision to initiate treatment is often based on the severity of liver disease, the degree of viremia, and the presence of other risk factors. Antiviral therapy can effectively suppress hepatitis B viral replication and reduce the risk of liver-related complications in individuals with compensated cirrhosis. Treatment with antiviral agents such as nucleoside analogs (e.g., entecavir or tenofovir) may be recommended for individuals with compensated cirrhosis and detectable viremia. However, the decision to initiate antiviral therapy in individuals with compensated cirrhosis and low levels of viremia may depend on various factors, including the individual's overall health status, the presence of other medical conditions, the risk of disease progression, and the potential side effects of antiviral therapy. Therefore, it is important for individuals with compensated cirrhosis to consult with their healthcare provider to determine the most appropriate course of treatment based on their individual medical history and current condition. | / | 3,2 | C |
| 19 | Short Answer Questions | 慢性乙肝常见症状有哪些 | 慢性乙肝是乙型肝炎病毒长期感染肝脏而导致的慢性肝炎病变。其常见症状包括： 1. 肝区不适感或疼痛，尤其是右上腹部疼痛。 2. 乏力、疲劳、身体不适感。 3. 食欲不振、恶心、呕吐、腹泻、便秘等胃肠道症状。 4. 黄疸，即皮肤、白眼球和黏膜等部位发黄。 5. 腹水，即腹部积液，导致腹部胀大和不适。 6. 体重下降，食欲减退等症状。 7. 血小板计数减少，可能导致出血倾向。 8. 肝功能异常，如肝酶升高、肝功能减退等。 9. 肝硬化，表现为腹水、黄疸、肝功能不全等症状。 10. 肝癌，长期慢性乙肝感染是导致肝癌的重要危险因素之一。 需要注意的是，有些患者可能没有任何症状，因此需要定期检查以及及时进行病毒检测。如果您出现这些症状，请尽快就医。 | / | 3,1 | C |
| 22 | Short Answer Questions | 简述乙肝病毒结构 | / | 乙肝病毒是一种双链DNA病毒，其结构由外壳层和核心层两部分组成。 1. 外壳层：外壳层主要由乙肝表面抗原（HBsAg）组成，其形状为球形或椭圆形，直径约为22纳米。外壳层的主要作用是保护病毒、促进病毒入侵宿主细胞，同时也是乙肝疫苗的主要成分。 2. 核心层：核心层主要由乙肝核心抗原（HBcAg）和乙肝e抗原（HBeAg）组成，形状为圆形或半圆形，直径约为30纳米。核心层包含了乙肝病毒的DNA，是病毒的遗传物质，也是乙肝病毒复制和繁殖的关键。 乙肝病毒的外壳层和核心层之间还存在一层膜状物质，称为基质（matrix），其作用是将外壳层和核心层粘在一起，维持乙肝病毒的整体结构。 了解乙肝病毒的结构可以帮助人们更好地了解乙肝感染的传播和治疗机制，同时也为研究乙肝疫苗和抗病毒药物提供了重要的基础知识。 | 2,3 | C |
| 32 | Short Answer Questions | 简述乙肝的预防策略 | / | 乙肝的预防策略主要包括以下几个方面： 1. 接种乙肝疫苗：乙肝疫苗是预防乙肝病毒感染最有效的方法之一，推荐所有人群进行接种。根据接种情况，乙肝疫苗可以分为基础免疫和加强免疫，推荐的接种时间和剂量也有所不同。 2. 保持个人卫生：乙肝病毒可以通过血液、性行为、母婴传播等途径传播，因此保持个人卫生非常重要。建议使用安全套、避免共用个人卫生用品等。 3. 避免与乙肝感染者接触：乙肝感染者的血液、体液等都可能携带乙肝病毒，接触这些物质可能会感染病毒。因此，避免与乙肝感染者接触，尤其是避免共用个人卫生用品等。 4. 食品安全：乙肝病毒可以通过污染的食品、饮水等途径传播，因此保障食品安全也是预防乙肝感染的重要措施。 5. 定期体检：对于有乙肝感染风险的人群，建议定期进行乙肝相关检测，及早发现和处理乙肝感染，预防病情恶化和传染给他人。 综上所述，乙肝的预防策略需要从个人、家庭和社会多个层面入手，建议大家加强宣传和教育，共同致力于预防和控制乙肝的发生和传播。 | 1,3 | C |
| 34 | Short Answer Questions | 简述乙肝疫苗禁忌人群 | / | 乙肝疫苗是一种安全有效的预防乙肝病毒感染的疫苗，但是对于某些人群来说，接种乙肝疫苗可能会出现禁忌证，包括以下几个方面： 1. 对乙肝疫苗过敏的人：对乙肝疫苗或其任何成分过敏的人不适宜接种乙肝疫苗。 2. 发热或急性疾病的人：对于正在发热或急性疾病的人，建议延迟接种乙肝疫苗，直到身体状况稳定后再接种。 3. 免疫系统受损的人：对于免疫系统受损的人，如患有某些疾病、正在接受免疫抑制治疗或放疗等，应在医生的指导下决定是否接种乙肝疫苗。 4. 妊娠期的女性：妊娠期的女性可以接种乙肝疫苗，但最好在怀孕前进行接种。如果在怀孕期间接种乙肝疫苗，应在医生的建议下进行决定。 5. 癫痫患者：有癫痫病史的人，接种乙肝疫苗时需要谨慎，应在医生的建议下进行决定。 需要注意的是，以上禁忌证并非绝对，对于一些特殊情况，需要根据医生的具体建议来决定是否接种乙肝疫苗。同时，接种乙肝疫苗的剂量和接种方案也应根据个人情况来制定。 | 2,3 | A |
| 35 | Short Answer Questions | 简述所有婴幼儿推荐接种乙肝疫苗的原因 | / | 以下是所有婴幼儿推荐接种乙肝疫苗的原因： 1. 预防乙肝病毒感染：乙肝病毒可以通过母婴传播、医疗器械传播等多种途径感染人体，而接种乙肝疫苗可以预防婴幼儿感染乙肝病毒，从而减少乙肝的发病率和传播风险。 2. 免疫保护期长：乙肝疫苗接种后，能够产生长期的免疫保护作用，即使是在婴幼儿期接种，也能够维持长达十几年的免疫力，有效降低儿童和成人感染乙肝病毒的风险。 3. 提高疫苗接种率：婴幼儿是接种疫苗的优先对象，他们的免疫系统比成人更为脆弱，而且在婴幼儿时期接种乙肝疫苗可以有效提高疫苗接种率，为后续的疫苗接种打下坚实的基础。 4. 降低治疗成本和治疗难度：乙肝是一种慢性肝病，需要长期的治疗和护理，而接种乙肝疫苗可以有效预防乙肝病毒感染，避免病情恶化，从而降低治疗成本和治疗难度。 因此，根据世界卫生组织和各国卫生部门的建议，所有婴幼儿都应该接种乙肝疫苗，以保护他们的健康和生命安全。 | 1,3 | E |
| 46 | Clinical Problems | 乙型肝炎核心抗体阳性怎么办？ | 如果您的乙型肝炎核心抗体（anti-HBc）检测结果为阳性，这意味着您曾经感染过乙型肝炎病毒（HBV），但目前可能已经康复或是成为了携带者。 建议您进行以下措施： 建议您去医院进行进一步检查，包括HBV DNA、乙肝表面抗原（HBsAg）等指标的检测，以了解您的肝炎病毒感染情况和病情严重程度。 如果您是慢性乙型肝炎病毒携带者，则需要接受医生的治疗和监护，并遵循医生的建议进行规范治疗，以控制和预防疾病的恶化。 如果您还没有接种过乙型肝炎疫苗，建议您尽快接种乙型肝炎疫苗，以预防乙型肝炎病毒的感染。 建议您遵守卫生习惯，避免与血液和体液直接接触，避免高危行为，如注射毒品、性行为等。 请注意，以上建议仅供参考，具体诊治和治疗方案应根据医生的诊断和建议进行。 | / | 3,1 | B |
| 50 | Clinical Problems | 乙肝可以治愈吗 | 是的，乙肝可以治愈，但是这取决于许多因素，包括感染乙肝病毒的时间、病毒的血清学标志物、肝脏损伤程度和是否存在肝硬化或肝癌等并发症。 对于许多乙肝患者来说，特别是那些处于慢性感染早期的人，通过药物治疗可以控制病毒复制并使肝脏恢复正常功能。对于其他患者，可能需要更长时间的治疗以达到完全恢复的目标。 对于那些患有肝硬化或肝癌的人，治疗的目标是控制疾病的进展并预防并发症的发生。在这些情况下，治愈可能是不可能的，但可以通过治疗延长生命并提高生活质量。 总的来说，治疗乙肝需要一个综合的方法，并且应根据每个人的独特情况进行个性化治疗。如果您认为自己可能感染了乙肝病毒，请尽快咨询医生以获取诊断和治疗建议。 | 是的，乙肝是一种可以治愈的疾病，但治愈的难度和可能性因患者的情况而异。 对于急性乙肝，多数患者能够在数周内自行恢复，这种情况下治愈率很高。而对于慢性乙肝，治愈的难度会增加，但仍有一定的可能性。目前，乙肝的主要治疗方法是使用抗病毒药物来抑制病毒复制和减轻症状。在长期治疗的过程中，一些患者可能会出现乙肝病毒表面抗原（HBsAg）阴转的情况，即病毒被完全清除，患者已经治愈。 需要注意的是，乙肝治愈不仅仅是指病毒被清除，还包括患者肝功能恢复正常、肝脏组织病变逐渐消失等多个方面。同时，对于已经成为慢性乙肝患者的人来说，尽早发现、积极治疗是非常重要的，可以减少肝功能受损和肝癌的风险。 | 3,3 | E,E |
| 56 | Clinical Problems | 乙肝疫苗加强针是什么，我需要打吗 | 乙肝疫苗加强针是一种用于加强人体对乙肝病毒免疫力的疫苗。一般来说，接种完初步的乙肝疫苗后，医生会建议在一定时间后进行加强针的接种，以增强对乙肝病毒的免疫力。加强针的接种次数和时间间隔会根据不同的情况而有所不同，具体应该咨询医生。 在一些地区，乙肝病毒较为常见，接种乙肝疫苗是保护自身健康的重要措施。如果你没有接种过乙肝疫苗，或者没有接种过加强针，那么建议咨询医生并考虑接种疫苗，以保护自己免受乙肝病毒的感染。 | 乙肝疫苗加强针是指在完成基础免疫接种（通常是在出生后的24小时内和后续几个月内完成的）之后，再次接种乙肝疫苗以增强身体对乙肝病毒的免疫力。加强针的接种时间通常在基础免疫接种完成后的6个月到1年之间。 对于需要打乙肝疫苗加强针的人群，通常包括以下几类人： 1.慢性乙肝病毒感染者：慢性乙肝病毒感染者的免疫系统可能无法有效清除病毒，因此需要加强针来提高免疫力。 2.具有乙肝感染风险的人群：例如医务工作者、卫生救援人员、实验室工作人员、有性接触风险的人群等。 3.具有乙肝疫苗接种失败风险的人群：例如免疫力低下、肝功能异常、重症疾病患者等。 如果您属于以上人群中的任何一类，建议您接种乙肝疫苗加强针以增强免疫力。如果您不确定是否需要接种，请咨询医生或前往当地的疾病预防控制中心咨询。 | 3,3 | D,C |
| 72 | Clinical Problems | 打完新冠疫苗第二针之后1周可以打乙肝疫苗吗 | / | 一般情况下，接种新冠疫苗和乙肝疫苗的时间间隔应该至少为4周，以确保身体有足够的时间产生免疫反应。因此，如果您刚刚接种完新冠疫苗第二针，建议您在至少4周之后再接种乙肝疫苗。但是，具体的接种时间还应该根据医生的建议和您的个人情况进行决定。如果您有任何疑问或担忧，建议您咨询医生或其他医疗专业人士的意见。 | 1,3 | C |
| 73 | Clinical Problems | 想去亚洲旅行，查到推荐打甲肝和乙肝疫苗，但时间只够打一针，打甲肝、乙肝、还是混合针最好？ | 如果您只有时间打一针疫苗，我建议您首先考虑接种乙肝疫苗。以下是一些原因： 1.乙肝病毒在亚洲地区比甲肝更为普遍，因此感染乙肝的风险更高。 2.乙肝是一种慢性病毒感染，会导致肝硬化和肝癌等严重并发症。甲肝通常是一种自限性疾病，大多数人可以自愈。 3.接种乙肝疫苗的效果比接种甲肝疫苗更好。根据疾病控制和预防中心的建议，接种乙肝疫苗需要完成三剂次，而接种甲肝疫苗只需要完成两剂次。 4.如果您有足够的时间，可以考虑接种混合疫苗，其中包括甲肝、乙肝和其他一些疫苗。但是，如果您只有时间打一针，那么我建议您优先考虑接种乙肝疫苗。 请注意，我是一名语言模型，不能替代医生或疫苗专家的建议。建议您咨询医生或专业人士的意见，以确定最适合您的疫苗接种方案。 | / | 3,1 | E |
| 81 | AASLD Guideline questions | 免疫耐受感染的成人是否应接受抗病毒治疗以减少肝脏相关并发症？ | 我不是医生，也不能提供具体的医学建议。但是，免疫耐受感染的成人是否应接受抗病毒治疗通常取决于多种因素，包括感染的类型、严重程度、个体的健康状况以及其他潜在风险因素。 免疫耐受感染通常指人体对某些病毒（如乙型肝炎病毒）产生了免疫反应，但病毒仍然存在于体内。在这种情况下，医生可能会考虑多种因素，以确定是否需要进行抗病毒治疗。 乙型肝炎是一种常见的免疫耐受感染情况，有时可能需要抗病毒治疗以防止肝脏相关并发症，如肝硬化和肝癌的发生。然而，治疗决策通常需要个体化，根据患者的具体情况进行。 如果你或有人对乙型肝炎或其他病毒感染有疑虑，最好咨询专业医生。医生将能够评估你的健康状况，进行必要的检查，并为你制定最合适的治疗方案。 | 我不是医生，不能提供具体的医疗建议。然而，如果成年人具有免疫耐受感染，特别是涉及肝脏的感染，例如乙型肝炎病毒（HBV）或丙型肝炎病毒（HCV），医生可能会考虑是否需要抗病毒治疗。这通常基于多种因素，包括病毒载量、肝功能、肝纤维化程度、患者的一般健康状况以及其他个体因素。 抗病毒治疗的目标可能是减缓疾病的进展、预防肝硬化和肝癌的发生，以及改善生活质量。对于HBV感染，抗病毒治疗可以帮助降低病毒载量，减少肝脏损伤。对于HCV感染，抗病毒治疗通常能够实现病毒持续性清除，从而预防肝炎的长期并发症。 最终的决定应由专业医疗团队根据患者的具体情况做出。如果您或您认识的人面临这种情况，请咨询医生以获取详细的诊断和治疗建议。 | 4,4 | C,D |
| 84 | AASLD Guideline questions | 在HBV单一感染者中，恩替卡韦治疗与替诺福韦治疗对肾脏和骨骼健康的影响是否不同？ | / | 截至我知识截止日期（2021年9月），我没有找到关于恩替卡韦（Entecavir）和替诺福韦（Tenofovir）在HBV（乙型肝炎病毒）单一感染者中对肾脏和骨骼健康影响的具体比较的最新研究。 这两种药物是用于治疗慢性HBV感染的核苷酸类似物，它们通过抑制病毒的复制来减缓疾病的进展。然而，不同药物可能会在患者体内产生不同的影响，包括对肾脏和骨骼的影响。 如果你对这个问题有具体的兴趣，我建议你查阅最新的医学文献、临床研究或专业医学指南，以获取关于这两种药物对肾脏和骨骼的影响的最新信息。此外，最好的做法是咨询专业的医疗保健提供者，以获取针对个体情况的建议和治疗方案。 | 1,4 | D |
| 86 | AASLD Guideline questions | 有低病毒血症的代偿期肝硬化患者是否应接受抗病毒药物治疗？ | 我不是医生，但我可以提供一些一般性的信息。对于乙肝患者是否应该接受抗病毒药物治疗，最好的建议是咨询专业医生，因为医生能够根据患者的具体情况做出最合适的决定。 一般来说，治疗决策取决于多个因素，包括患者的病毒载量、肝功能、肝脏纤维化或硬化的程度，以及是否存在其他健康问题。对于代偿期乙肝患者，即肝脏功能在正常范围内的患者，医生可能会根据病毒载量和其他指标来评估是否需要抗病毒治疗。 一些专家组织和指南建议，对于代偿期患者，如果存在高病毒载量、肝功能异常或肝脏纤维化程度较高的情况，可能需要考虑抗病毒治疗，以减缓疾病进展和降低肝硬化、肝癌等并发症的风险。 总之，乙肝治疗的决策是一个复杂的过程，应该由专业医生根据患者的具体情况进行评估。如果您或您认识的人患有乙肝，建议咨询医生以获取专业建议。 | 我不是医生，但我可以提供一些一般性的信息供您参考。对于乙肝肝硬化患者是否应接受抗病毒药物治疗，最好的建议是咨询专业医生，因为医疗决策应该基于个体病情和医生的专业意见。 一般来说，治疗乙肝的决定取决于多个因素，包括病毒活动水平、肝脏炎症和纤维化程度、患者的年龄、性别、肝功能以及患者的整体健康状况。 对于乙肝患者，低病毒血症并不一定表示没有病毒活动。即使在低病毒血症的情况下，患者的肝脏仍然可能受到损害，导致肝硬化的发展。因此，医生可能会考虑抗病毒药物治疗以抑制病毒复制，减缓或阻止肝脏的病变。 再次强调，最终的决策应由医生根据患者的具体情况做出。如果您或您认识的人有乙肝相关问题，建议寻求专业医生的建议和治疗。 | 3,3 | C,C |
| 87 | AASLD Guideline questions | 高病毒载量的乙型肝炎表面抗原阳性孕妇是否应在妊娠晚期接受抗病毒治疗以预防HBV的围产期传播？ | 我不是医生，因此不能提供具体的医学建议。然而，我可以向您提供一些一般性的信息。 乙型肝炎病毒（HBV）是一种可以通过血液和其他体液传播的病毒，而孕妇如果感染了HBV，有可能将病毒传给新生儿。高病毒载量的乙型肝炎表面抗原（HBsAg）阳性是一种孕妇可能会传播给胎儿的风险因素。 抗病毒治疗是否适合孕妇需要根据具体情况来判断，包括孕妇的病毒载量、肝功能状况、孕期、医学历史等因素。对于乙肝携带者的孕妇，医生可能会根据孕妇的具体情况来评估是否需要进行抗病毒治疗，以降低围产期传播的风险。 如果您或您认识的人是乙型肝炎表面抗原阳性的孕妇，建议咨询专业医生的意见。医生将能够根据具体情况制定适当的治疗计划，并权衡治疗的利弊，以确保母婴的健康。 | 我不是医生，但我可以提供一些一般性的信息。乙型肝炎（HBV）是一种由乙型肝炎病毒引起的病毒性感染。对于怀孕的女性，特别是在妊娠晚期，如果她们是高病毒载量的乙型肝炎表面抗原（HBsAg）阳性，医生可能会考虑采取一些预防措施，以减少新生儿HBV感染的风险。 一般而言，垂直传播是新生儿感染HBV的主要途径。在这种情况下，医生可能会考虑采取以下预防措施： 1.乙肝免疫球蛋白（HBIG）注射： 在新生儿出生后的12小时内，可以给予HBIG注射，以提供即时 passively 获得的被动免疫，减少HBV传播的风险。 2.乙肝疫苗接种： 新生儿应该在出生后尽早接种乙肝疫苗，这样可以提高他们对乙型肝炎的主动免疫水平。 此外，医生还可能根据具体情况考虑给予抗病毒药物，以降低母体病毒载量，减少传播的风险。然而，对于是否使用抗病毒药物以及具体使用哪种药物，应该由专业的医疗团队评估患者的个体情况，并根据权衡利弊作出决策。 如果你或者你认识的人有类似的情况，强烈建议咨询专业医生的建议，他们能够提供个性化的医疗建议和治疗方案。 | 4,3 | D,B |
| 88 | AASLD Guideline questions | 阳性慢性乙型病毒性肝炎患儿是否应接受抗病毒治疗以减少肝脏相关并发症？ | / | 我不是医生，因此不能提供专业医学建议。但一般来说，对于慢性乙型病毒性肝炎的患者，是否需要接受抗病毒治疗应该由医生根据患者的具体情况来决定。以下是一些通常考虑的因素： 1.病毒活动性： 医生通常会评估乙型肝炎病毒的活动程度。如果病毒活动性较高，可能会倾向于开始抗病毒治疗，以减少病毒复制并防止肝脏进一步受损。 2.肝功能： 医生会评估患者的肝功能，包括肝酶水平和其他指标。如果肝功能受损，可能需要考虑抗病毒治疗以减轻肝脏负担。 3.肝纤维化和肝硬化： 如果患者已经出现了肝纤维化或肝硬化的迹象，可能需要更积极地干预，以防止疾病进展。 4.患者的整体健康状况： 医生还会考虑患者的整体健康状况，包括其他慢性病、免疫状态等因素。 请务必咨询专业医生，以获取针对个体情况的建议。他们可以评估患者的病历、实验室结果和其他相关信息，并制定最合适的治疗方案。 | 2,3 | C |
| 93 | Simulated Patients | 一个44岁的女性患者，HBV-DNA 10^8 IU/ml，HBeAg阳性，没有肝硬化，eGFR 105 ml/min，ALT 110 U/L，BMI 22.5kg/m^2，有高血压病史，有吸烟和饮酒习惯，未进行抗病毒治疗。请给出用药方案和生活方式建议。 | 首先，需要说明我不是医生，不能提供专业医疗建议。针对HBV感染的治疗，最好咨询专业医生以获取个性化的建议。以下是一般性的信息，仅供参考。 对于这位患者，考虑到她的HBeAg阳性，高血压，吸烟和饮酒习惯，以及ALT水平升高，可能需要综合治疗的方法，包括抗病毒治疗、生活方式干预和相关药物治疗高血压。 1.抗病毒治疗： 针对患者的HBV-DNA水平，可能需要考虑抗病毒治疗。抗病毒药物的选择应由专业医生决定，通常会考虑核苷(tide)或核酸酸(tide)类药物，如阿德福韦酯或替比夫定。治疗的目标是抑制病毒复制，减缓疾病的进展。 2.生活方式建议： 戒烟戒酒：吸烟和饮酒都会对肝脏健康产生不良影响，对于HBV感染的患者来说更是如此。尽量戒烟和限制饮酒。 饮食和运动：保持健康的饮食习惯，避免高脂肪、高糖分的食物。适量的锻炼对于整体健康有益，但在开始运动之前最好咨询医生的建议。 控制体重：保持适当的体重对于控制高血压和改善肝脏健康都很重要。 3.管理高血压： 考虑高血压病史，可能需要药物治疗。抗高血压药物的选择应该由医生根据患者的具体情况来确定。 4.监测： 定期进行检查，包括HBV病毒载量、肝功能、肾功能和血压监测等，以便及时调整治疗方案。 请记住，以上建议是一般性的，具体治疗方案需要根据患者的详细病史和体检结果由专业医生来制定。患者应该密切关注医生的建议，定期进行复查，并积极参与治疗过程。 | 请注意，我不是医生，以下只是一些建议，具体的治疗方案应该由专业医生根据详细的临床情况进行制定。对于慢性乙型肝炎（HBV）的治疗，建议您咨询专业医生以获取最准确的建议。 根据提供的信息，您的患者属于慢性HBV感染，目前HBV-DNA水平较高，HBeAg阳性，ALT升高，但无肝硬化。以下是可能的治疗方案和生活方式建议： 1. 抗病毒治疗方案： 抗病毒药物： 对于HBV患者，抗病毒治疗是关键。一些常用的抗病毒药物包括： Nucleos(t)ide analogs (NAs)： 如恩替卡韦（Entecavir）、替比夫定（Tenofovir）等。这些药物可以有效抑制病毒复制，减少炎症和改善肝功能。 2. 生活方式建议： 戒烟和限制饮酒： 戒烟和限制饮酒是非常重要的。吸烟和过量饮酒都会对肝脏健康产生负面影响，尤其是在慢性乙型肝炎的情况下。 控制体重： 维持健康的体重对于肝脏健康至关重要。尽量保持适当的体重，避免肥胖。 健康饮食： 采用均衡的饮食，多摄取新鲜水果、蔬菜、全谷物和低脂蛋白质，有助于支持免疫系统和维持肝脏功能。 控制高血压： 由于患者有高血压病史，需要积极控制血压，可能需要药物治疗，同时也可以通过生活方式改变，如控制钠盐摄入、增加锻炼等。 3. 定期监测： 定期检查肝功能： 包括ALT、AST等指标，以监测肝脏炎症的程度。 HBV-DNA检测： 定期检测HBV-DNA水平，以评估病毒复制的情况。 血压监测： 定期监测血压，确保血压在合适范围内。 请务必在制定治疗方案之前与专业医生咨询。这些建议仅供参考，具体治疗方案需要根据患者的具体病情和体征由医生来确定。 | 3,3 | C,E |
