# Supplementary material for: Assessing ChatGPT as a Medical Consultation Assistant for Chronic Hepatitis B: Cross-Language Study of English and Chinese
Source: JMIR Med Inform. 2024 Aug 8;12:e56426. doi: 10.2196/56426 (PMC11342014; doi:10.2196/56426)
Supplement: Multimedia Appendix 5 [file medinform_v12i1e56426_app5.docx]

**Multimedia Appendix 5.** Content consistency assessment of ChatGPT-3.5.

| Number | Classicfication | Question | Response1 | Response2 | Reviewer1 | Reviewer2 | Reviewer3 | Consistency |
| --- | --- | --- | --- | --- | --- | --- | --- | --- |
| 1 | Term Explanation | What is hepatitis B | Hepatitis B is a viral infection that affects the liver. It is caused by the hepatitis B virus (HBV) and can cause both acute and chronic illness.  Acute hepatitis B occurs when a person is first infected with the virus. It can cause symptoms such as fever, fatigue, loss of appetite, nausea, vomiting, abdominal pain, dark urine, and jaundice (yellowing of the skin and eyes). Most people with acute hepatitis B recover fully within a few months, but in some cases, the infection can lead to severe liver damage, liver failure, or death.  Chronic hepatitis B occurs when the virus remains in the body for more than six months. It can cause ongoing inflammation in the liver, which can lead to cirrhosis (scarring of the liver) and liver cancer. People with chronic hepatitis B may not have symptoms initially but can develop serious liver complications over time.  Hepatitis B is spread through contact with the blood or bodily fluids of an infected person. It can be spread through sexual contact, sharing needles or other equipment used for injecting drugs, from mother to baby during childbirth, or through contact with open wounds or sores of an infected person.  Hepatitis B can be prevented through vaccination, practicing safe sex, not sharing needles or other equipment used for injecting drugs, and avoiding contact with blood and bodily fluids from infected individuals. Treatment for hepatitis B may include antiviral medications, regular monitoring of liver function, and in some cases, liver transplant. | Hepatitis B is a viral infection caused by the hepatitis B virus (HBV) that can cause inflammation and damage to the liver. HBV is a blood-borne virus that can be transmitted through contact with infected blood, semen, vaginal fluids, or other bodily fluids. HBV can also be transmitted from an infected mother to her baby during childbirth.  The symptoms of hepatitis B can vary from mild to severe and can include fatigue, fever, abdominal pain, nausea, vomiting, and jaundice. In some cases, people infected with HBV may not have any symptoms at all, but they can still transmit the virus to others.  HBV infection can be acute or chronic. Acute infection is a short-term illness that usually lasts a few weeks, and the body is able to clear the virus on its own. Chronic infection, however, is a long-term illness that can last a lifetime and can lead to serious health problems such as liver damage, cirrhosis, and liver cancer.  Prevention of HBV infection includes getting vaccinated against hepatitis B, avoiding sharing needles or other equipment that could spread the virus, practicing safe sex to prevent sexual transmission of the virus, and getting tested for HBV if you are at risk of infection. Treatment for chronic HBV infection may include antiviral medications to suppress the replication of the virus and to reduce the risk of liver damage. | Yes | Yes |  | Yes |
| 1 | Term Explanation | 乙肝是什么 | 乙肝是指乙型肝炎病毒（HBV）感染引起的肝炎，是一种严重的传染病。乙型肝炎病毒是一种DNA病毒，可以在人体内长期存活并繁殖，引起肝细胞的破坏和肝功能异常。乙型肝炎病毒感染可以分为急性感染和慢性感染两种类型。 急性乙肝通常在感染后数周至数月内发病，患者可能会出现发热、疲劳、食欲不振、肝区疼痛、黄疸等症状，但多数患者可以自愈，少数患者可能转化为慢性感染。 慢性乙肝是指乙型肝炎病毒在人体内持续存在超过六个月，大多数患者无明显症状，但长期潜伏可导致肝硬化、肝癌等严重并发症。乙肝的传播途径包括血液、性接触、婴儿母亲传播、共用针头等。预防乙肝的有效措施包括接种疫苗、避免接触感染途径等。治疗方法包括抗病毒药物、免疫调节剂、肝保护药等。 | 乙肝，又称乙型肝炎，是由乙型肝炎病毒（HBV）引起的一种肝炎。乙肝病毒主要通过血液和其他体液传播，包括性传播、母婴传播、注射毒品、血液透析等途径。乙肝病毒感染后，可能会引起急性肝炎，也可能在体内长期潜伏而不发作，成为慢性乙肝。  乙肝病毒感染后，病毒会进入肝细胞内进行复制和繁殖，导致肝细胞受损和炎症，进而出现肝炎、肝硬化、肝癌等疾病。乙肝病毒感染的症状包括疲劳、食欲不振、恶心、呕吐、肝区疼痛、黄疸等，但部分感染者可能没有明显症状。  乙肝是一种可预防和可治疗的疾病。乙肝疫苗是预防乙肝的最有效方法，接种乙肝疫苗可以有效降低乙肝的感染风险。对于已经感染乙肝病毒的患者，抗病毒治疗是常用的治疗方法。抗病毒治疗可以减少病毒载量，预防病情进展和并发症的发生。同时，保持良好的生活方式和饮食习惯，避免高危行为，可以降低乙肝病毒感染的风险。 | Yes | Yes |  | Yes |
| 2 | Term Explanation | What is hepatitis B vaccine | The hepatitis B vaccine is a vaccine that helps to protect against infection with the hepatitis B virus (HBV). It contains a part of the HBV called the surface antigen, which triggers the body's immune system to produce protective antibodies against the virus.  The hepatitis B vaccine is given in a series of three or four doses, depending on the vaccine used. The doses are typically given over a six-month period, with the second dose given one month after the first dose and the third dose given six months after the first dose. For some high-risk individuals, such as healthcare workers or people living with someone with chronic hepatitis B, an accelerated schedule of the vaccine may be used.  The hepatitis B vaccine is safe and effective and has been in use since the 1980s. It is recommended for all infants and children, as well as for adults who are at increased risk for infection with HBV, such as healthcare workers, people with multiple sexual partners, people who inject drugs, and people with chronic liver disease.  The hepatitis B vaccine is considered one of the most effective vaccines available, with more than 95% of people who receive the full vaccine series developing protective antibodies against HBV. It is an important tool in the prevention of hepatitis B and the serious liver diseases that can result from infection with the virus. | The hepatitis B vaccine is a vaccine that protects against the hepatitis B virus (HBV). The vaccine contains a small part of the hepatitis B virus, known as the hepatitis B surface antigen (HBsAg), which triggers the immune system to produce antibodies against the virus.  The hepatitis B vaccine is given in a series of three or four shots over a period of six months. The vaccine is recommended for all infants, children, and adults who have not been vaccinated and who are at risk of HBV infection, including healthcare workers, people with multiple sex partners, people with chronic liver disease, and people who inject drugs.  The hepatitis B vaccine is highly effective and provides long-lasting protection against HBV infection. It is estimated that more than 95% of people who receive the complete hepatitis B vaccine series develop protective antibodies.  Side effects of the hepatitis B vaccine are usually mild and include soreness, redness, or swelling at the injection site, fever, and fatigue. Serious side effects are rare.  Getting vaccinated against hepatitis B is the most effective way to prevent HBV infection and its complications, including liver damage, cirrhosis, and liver cancer. It is recommended that everyone receive the hepatitis B vaccine to protect themselves and to prevent the spread of the virus to others. | Yes | Yes |  | Yes |
| 2 | Term Explanation | 乙肝疫苗是什么 | 乙肝疫苗是一种预防乙型肝炎病毒（HBV）感染的疫苗。乙肝疫苗主要由乙肝表面抗原（HBsAg）制成，可以刺激人体免疫系统产生针对HBsAg的抗体，从而预防乙肝病毒的感染。 乙肝疫苗可以通过肌肉注射或皮下注射的方式接种，通常分为三剂接种，第一剂和第二剂之间间隔4周，第二剂和第三剂之间间隔5-6个月。在完成三剂疫苗接种后，大多数人可以产生免疫保护，并具有长期的保护效果。 乙肝疫苗是目前预防乙肝病毒感染的最有效措施之一，被认为是预防乙肝的最佳方法。除此之外，还可以通过避免接触乙肝病毒的途径（如避免共用针头、避免性接触等）等措施来预防乙肝病毒的传播。 | 乙肝疫苗是一种可以预防乙型肝炎病毒感染的疫苗。该疫苗由乙型肝炎表面抗原（HBsAg）制成，通过注射疫苗可以让人体产生针对HBsAg的免疫反应，从而形成免疫保护，预防乙型肝炎病毒感染。  乙肝疫苗分为重组乙肝疫苗和减毒乙肝疫苗两种。重组乙肝疫苗是通过基因工程技术将HBsAg基因克隆到酵母细胞或其他宿主细胞中，制备成的疫苗。减毒乙肝疫苗是以减毒乙肝病毒为载体制备的疫苗。两种疫苗的接种程序和接种剂量略有不同，但均能有效预防乙型肝炎病毒感染。  乙肝疫苗是一种安全有效的疫苗，已被广泛应用于全球范围内的乙型肝炎疫苗接种计划中。根据世界卫生组织的推荐，乙肝疫苗应该在出生后24小时内接种，接种周期为3剂次，一般在出生后的第0、1和6个月接种，也可以根据不同接种程序和剂量的要求进行接种。 | Yes | Yes |  | Yes |
| 3 | Term Explanation | What is hepatitis B virus | Hepatitis B virus (HBV) is a blood-borne virus that primarily infects the liver and can cause serious liver disease. It is a member of the Hepadnaviridae family of viruses, which are small, partially double-stranded DNA viruses. The HBV genome consists of four overlapping open reading frames that encode several viral proteins, including the surface antigen (HBsAg), core antigen (HBcAg), and polymerase. These viral proteins are important targets for the body's immune system in response to HBV infection. HBV is transmitted through contact with infected blood or other bodily fluids, such as semen and vaginal secretions. It can be spread through sexual contact, sharing needles or other equipment used for injecting drugs, from mother to baby during childbirth, or through contact with open wounds or sores of an infected person. Hepatitis B can cause acute or chronic infection. Acute infection can range in severity from a mild illness with few or no symptoms to a severe illness that can lead to liver failure and death. Chronic infection occurs when the virus remains in the body for more than six months and can lead to serious liver conditions such as cirrhosis, liver cancer, and liver failure. Hepatitis B is preventable with a safe and effective vaccine that has been available since 1982. Treatment for chronic hepatitis B includes antiviral medications and regular monitoring of liver function. It is important for people at high risk for hepatitis B infection, such as healthcare workers and those with multiple sexual partners or who inject drugs, to get vaccinated and to take other precautions to prevent transmission of the virus. | The hepatitis B virus (HBV) is a virus that can cause inflammation and damage to the liver. It is a blood-borne virus that is transmitted through contact with infected blood, semen, vaginal fluids, or other bodily fluids. HBV can also be transmitted from an infected mother to her baby during childbirth. Symptoms of hepatitis B can include fatigue, fever, abdominal pain, nausea, vomiting, and jaundice. In some cases, people infected with HBV may not have any symptoms at all, but they can still transmit the virus to others. HBV infection can be acute or chronic. Acute infection is a short-term illness that usually lasts a few weeks, and the body is able to clear the virus on its own. Chronic infection, however, is a long-term illness that can last a lifetime and can lead to serious health problems such as liver damage, cirrhosis, and liver cancer. Prevention of HBV infection includes getting vaccinated against hepatitis B, avoiding sharing needles or other equipment that could spread the virus, practicing safe sex to prevent sexual transmission of the virus, and getting tested for HBV if you are at risk of infection. Treatment for chronic HBV infection may include antiviral medications to suppress the replication of the virus and to reduce the risk of liver damage. | Yes | Yes |  | Yes |
| 3 | Term Explanation | 乙肝病毒是什么 | 乙肝病毒指的是乙型肝炎病毒（HBV），是一种DNA病毒。乙型肝炎病毒是引起乙型肝炎的主要病因，可通过血液、性行为等途径传播，引起肝细胞的破坏和肝功能异常。 乙肝病毒具有强的传染性，可以通过接触感染途径（如血液、体液、母婴传播、共用针头等）传播。乙肝病毒感染可以分为急性感染和慢性感染两种类型。急性感染在感染后短期内出现症状，如发热、疲劳、食欲不振、肝区疼痛、黄疸等，随后可能会自行恢复，但也可能会转化为慢性感染。慢性感染则是指乙型肝炎病毒在人体内持续存在六个月或以上，大多数患者无明显症状，但长期潜伏可导致肝硬化、肝癌等严重并发症。 乙肝病毒的诊断可以通过检测血液中的乙肝表面抗原（HBsAg）和抗体（如乙肝表面抗体、乙肝e抗原和抗体等）来确定。预防乙肝病毒感染的有效措施包括接种疫苗、避免接触感染途径等。治疗方法包括抗病毒药物、免疫调节剂、肝保护药等。 | 乙肝病毒（Hepatitis B virus，HBV）是一种DNA病毒，是导致肝炎、肝硬化和肝癌的主要病因之一。乙肝病毒主要通过血液、性接触、母婴传播等途径传播。感染乙肝病毒后，有些人会出现急性乙型肝炎症状，如疲劳、发热、恶心、呕吐、腹泻、腹痛等；而另一些人可能无任何症状，但仍然可以成为慢性乙型肝炎的患者。  乙肝病毒是一种非常坚韧的病毒，可以在外界环境中存活长达7天，因此在预防乙肝感染方面，应该注意避免血液、性接触、母婴传播等途径的风险，同时接种乙肝疫苗也是一种有效的预防措施。如果已经感染了乙肝病毒，应该及时就医，遵循医生的治疗方案进行治疗。 | Yes | Yes |  | Yes |
| 4 | Term Explanation | What is five indicator test for Hepatitis B | The five indicator tests for hepatitis B are a set of blood tests used to evaluate a person's current or previous hepatitis B infection status. These tests are often ordered together and include: 1.HBsAg (Hepatitis B surface antigen) test: This test checks for the presence of HBsAg, which is a protein on the surface of the hepatitis B virus. If this antigen is detected in the blood, it indicates a current hepatitis B infection. 2.HBsAb (Hepatitis B surface antibody) test: This test checks for the presence of antibodies to HBsAg, which are produced by the immune system in response to a past hepatitis B infection or vaccination. 3.HBcAb (Hepatitis B core antibody) test: This test checks for the presence of antibodies to the hepatitis B core antigen, which is a protein that makes up the inner core of the hepatitis B virus. The presence of these antibodies indicates a past or current hepatitis B infection. 4.HBeAg (Hepatitis B e antigen) test: This test checks for the presence of HBeAg, which is a protein produced by the hepatitis B virus. If this antigen is detected in the blood, it indicates a higher risk of transmitting the virus to others. 5.HBeAb (Hepatitis B e antibody) test: This test checks for the presence of antibodies to HBeAg, which are produced by the immune system in response to the presence of HBeAg. The presence of these antibodies indicates a lower risk of transmitting the virus to others. These tests are important for the diagnosis and management of hepatitis B infection. They can help determine if a person is currently infected, has been infected in the past, or has immunity due to vaccination or previous infection. The results of these tests can also guide treatment decisions for people with chronic hepatitis B infection. | The five indicator tests for hepatitis B are a set of blood tests that are used to diagnose and monitor hepatitis B infection. These tests are: 1.Hepatitis B surface antigen (HBsAg) test: This test detects the presence of the hepatitis B surface antigen, which is a protein on the surface of the hepatitis B virus. A positive result indicates that the person is infected with HBV. 2.Hepatitis B surface antibody (HBsAb) test: This test detects the presence of antibodies against the hepatitis B surface antigen, which can be produced naturally after a person recovers from acute HBV infection or after vaccination against the virus. A positive result indicates that the person has immunity to HBV. 3.Hepatitis B core antibody (HBcAb) test: This test detects the presence of antibodies against the hepatitis B core antigen, which is a protein inside the hepatitis B virus. A positive result indicates that the person has been exposed to HBV at some point in the past. 4.Hepatitis B e antigen (HBeAg) test: This test detects the presence of the hepatitis B e antigen, which is a protein produced by the hepatitis B virus. A positive result indicates that the person is highly infectious and the virus is actively replicating in the body. 5.Hepatitis B e antibody (HBeAb) test: This test detects the presence of antibodies against the hepatitis B e antigen. A positive result indicates that the person's immune system has responded to the virus and the virus is no longer actively replicating. These five indicator tests are used in combination to diagnose and monitor hepatitis B infection. The results of these tests can help healthcare providers determine the stage of the infection, the risk of transmission, and the need for treatment. | Yes | Yes |  | Yes |
| 4 | Term Explanation | 乙肝五项是什么 | 乙肝五项是指针对乙型肝炎病毒感染的五项常规检查，包括乙肝表面抗原（HBsAg）、乙肝表面抗体（HBsAb）、乙肝e抗原（HBeAg）、乙肝e抗体（HBeAb）和乙肝核心抗体（HBcAb）。这些指标可以反映一个人是否感染了乙型肝炎病毒、是否具有乙肝病毒的免疫保护等信息。 • HBsAg：是乙肝病毒的表面抗原，它存在于病毒颗粒的外表面。HBsAg检测可以确定是否感染了乙肝病毒。 • HBsAb：是乙肝病毒表面抗体，也称为乙肝疫苗抗体，是人体免疫系统产生的一种抗体，可以预防乙肝病毒的感染。 • HBeAg：是乙肝病毒e抗原，它表示病毒正在活跃地复制和感染肝细胞。 • HBeAb：是乙肝病毒e抗体，也称为乙肝e抗体，是人体免疫系统产生的一种抗体，可以反映乙肝病毒的复制程度。 • HBcAb：是乙肝病毒核心抗体，可以反映是否曾经感染过乙肝病毒。 通过检测这五项指标，可以对乙肝病毒感染的状态进行评估，并采取相应的预防和治疗措施。 乙肝五项是指针对乙型肝炎病毒（HBV）进行的五项检测指标，包括HBsAg、HBsAb、HBcAb、HBeAg、HBeAb。这五项检测指标可以全面评估HBV感染的情况，帮助医生做出正确的诊断和治疗方案。 | 具体来说，这五项检测指标的含义如下：  1.HBsAg：乙型肝炎表面抗原，阳性表示病毒已经进入人体，可能存在HBV感染。  2.HBsAb：乙型肝炎表面抗体，阳性表示人体免疫系统已经对病毒产生了免疫应答，具有一定的免疫保护力。  3.HBcAb：乙型肝炎核心抗体，阳性表示人体曾经感染过HBV，或者正在感染。  4.HBeAg：乙型肝炎e抗原，阳性表示病毒在肝细胞内大量复制和繁殖，并且患者是具有高传染性的状态。  5.HBeAb：乙型肝炎e抗体，阳性表示病毒已经被免疫系统清除，不再具有传染性。  乙肝五项检测是诊断乙型肝炎的重要方法之一，可以全面了解感染者的乙肝病毒感染情况，并指导医生制定合理的治疗方案和预防措施。 | Yes | Yes |  | Yes |
| 5 | Term Explanation | What is hepatitis B surface antibody (HBsAb) | Hepatitis B surface antibody (HBsAb), also known as anti-HBs or HBsAg antibody, is a type of antibody that is produced by the body's immune system in response to exposure to the hepatitis B virus (HBV). HBsAb is specific to the surface antigen of the hepatitis B virus (HBsAg), which is a protein that is found on the outer surface of the virus. When a person is exposed to HBV, their immune system recognizes the HBsAg as a foreign invader and produces antibodies to neutralize it. The presence of HBsAb in the blood indicates that a person has either been vaccinated against hepatitis B or has recovered from a past infection with the virus. People who have HBsAb are considered to be immune to hepatitis B and are protected against future infection. HBsAb is often measured in combination with other hepatitis B-related blood tests, such as HBsAg and HBcAb, to determine a person's hepatitis B infection status. If a person tests positive for HBsAb but negative for HBsAg and HBcAb, it indicates that they have been vaccinated against hepatitis B. If a person tests positive for both HBsAb and HBcAb, it indicates that they have recovered from a past hepatitis B infection. Testing for HBsAb is an important part of hepatitis B screening and management. It can help determine a person's immunity status and guide decisions about vaccination or further testing. | The hepatitis B surface antibody (HBsAb), also known as anti-HBs, is a protein produced by the body's immune system in response to the hepatitis B virus (HBV) vaccine or a previous HBV infection. The presence of HBsAb in the blood indicates that a person has developed immunity to the hepatitis B virus. This immunity can be natural, as a result of a past HBV infection that has been resolved, or acquired, as a result of vaccination against the virus. The HBsAb test is used to determine if a person has been previously infected with HBV or if they have developed immunity to the virus through vaccination. The test is also used to monitor the effectiveness of the vaccine and to determine if a booster shot is needed. A positive HBsAb test result indicates that the person has immunity to HBV, while a negative result indicates that the person does not have immunity and may be at risk of infection. In some cases, the HBsAb test may be performed along with other tests, such as the hepatitis B surface antigen (HBsAg) test, to help diagnose or monitor hepatitis B infection. | Yes | Yes |  | Yes |
| 5 | Term Explanation | 乙肝表面抗体是什么 | 乙肝表面抗体（HBsAb）是一种人体免疫系统产生的抗体，主要作用是与乙肝病毒表面抗原（HBsAg）结合，从而中和病毒，防止乙肝病毒侵入人体细胞并引起感染。HBsAb也是乙肝疫苗接种后形成的一种抗体，是预防乙肝病毒感染的重要免疫指标之一。 在乙肝病毒感染和恢复过程中，人体免疫系统会产生HBsAb。对于未感染过乙肝病毒的人，接种乙肝疫苗后，一般会在接种后1-2个月内产生充足的HBsAb，形成有效的免疫保护。对于已感染乙肝病毒或患有慢性乙肝的患者，HBsAb水平可能较低或不存在，因此需要根据具体情况进行个体化治疗。 检测HBsAb水平可以帮助评估乙肝病毒感染的免疫状态，判断是否具有乙肝病毒的免疫保护。在乙肝疫苗接种后，检测HBsAb水平也可以评估疫苗接种效果和免疫保护状态。 | 乙肝表面抗体（HBsAb）是人体针对乙型肝炎表面抗原（HBsAg）产生的抗体。当人体免疫系统暴露于乙型肝炎病毒（HBV）时，会产生抗体来抵抗这种病毒。在HBV感染后，人体免疫系统会产生不同类型的抗体，其中HBsAb是一种能够中和HBV的表面抗原的抗体。在乙肝疫苗接种后，人体也会产生HBsAb，形成免疫保护，预防乙型肝炎病毒感染。 乙肝表面抗体检测是检测人体是否曾经感染过HBV或者接种过乙肝疫苗的重要方法之一。在HBsAb检测结果为阳性时，表示人体免疫系统已经产生了对HBsAg的免疫应答，即曾经感染过HBV或接种过乙肝疫苗，具备一定的免疫保护力。因此，HBsAb检测常常用于乙肝疫苗接种后的免疫效果检测、慢性乙肝病毒感染的评估和治疗等方面。 需要注意的是，HBsAb阴性并不能完全排除乙肝病毒感染的可能，因为不同的人体免疫系统对乙肝病毒的应答不同。因此，如果怀疑自己存在乙肝病毒感染风险，还需要进行其他乙肝病毒相关检测，如HBsAg、HBcAb、HBeAg等检测。 | Yes | Yes |  | Yes |
| 6 | Term Explanation | NA |  |  |  |  |  |  |
| 6 | Term Explanation | 乙肝大三阳是什么 | 乙肝大三阳是指乙肝病毒感染时，乙肝表面抗原（HBsAg）、乙肝e抗原（HBeAg）和乙肝核心抗体（HBcAb）三项指标同时呈阳性的检测结果，也被称为“HBsAg、HBeAg、HBcAb三合一”或“三阳性”。 乙肝大三阳的检测结果通常表示患者处于急性乙肝病毒感染的活动期，病毒正在活跃地复制和感染肝细胞。此时，患者具有高传染性，可以通过血液、性行为等途径传播病毒。同时，乙肝大三阳的检测结果也可能意味着患者病情较为严重，需要及时接受治疗。 对于乙肝大三阳的患者，需要及时进行肝功能检查、血清病毒学指标检测和肝脏超声等检查，以确定病情和制定治疗方案。治疗方法包括抗病毒治疗、免疫调节剂、肝保护药物等，以及控制乙肝病毒传播的有效措施，如避免接触感染途径、使用无菌注射器等。 | 乙肝大三阳是指血液中乙型肝炎病毒（HBV）三项检测指标中的三项均阳性，即乙肝表面抗原（HBsAg）、乙肝表面抗体（HBsAb）和乙肝e抗原（HBeAg）均为阳性。乙肝大三阳通常是急性乙肝病毒感染的表现，也可以是慢性乙肝病毒感染的表现。 HBsAg阳性表示病毒已经进入人体，而HBsAb阳性表示免疫系统已经对病毒产生了免疫应答。HBeAg阳性则表示病毒在肝细胞内大量复制和繁殖，并且患者是具有高传染性的状态，即患者可以通过血液、性接触、母婴传播等途径将病毒传染给他人。 对于乙肝大三阳的患者，应该及时就医，接受医生的治疗方案进行治疗，并且采取措施避免乙肝病毒传播。在治疗过程中，需要密切监测病情变化和治疗效果，及时调整治疗方案。此外，乙肝大三阳还需要注意饮食、休息等方面的调整，增强身体免疫力，促进康复。 | Yes | Yes |  | Yes |
| 7 | Term Explanation | NA |  |  |  |  |  |  |
| 7 | Term Explanation | 乙肝小三阳是什么 | 乙肝小三阳是指乙肝表面抗原（HBsAg）和乙肝核心抗体（HBcAb）呈阳性，而乙肝e抗原（HBeAg）呈阴性的检测结果。因为只有两项指标呈阳性，所以被称为“小三阳”。 乙肝小三阳通常表示患者已经感染了乙肝病毒，但病毒处于较为稳定的状态，不活跃地复制和感染肝细胞。与乙肝大三阳不同，乙肝小三阳的患者传染性较低，不会像大三阳那样轻易地传播病毒。 对于乙肝小三阳的患者，需要密切关注病情变化，并定期进行肝功能检查、血清病毒学指标检测和肝脏超声等检查，以及采取必要的措施控制病情发展和传播病毒。在具体治疗方案上，视患者的具体情况而定，可以采用抗病毒治疗、免疫调节剂等方法来治疗和控制病情。此外，避免接触感染途径、使用无菌注射器等措施也可以有效预防乙肝病毒传播。 | 乙肝小三阳是指血液中乙型肝炎病毒（HBV）三项检测指标中的两项阳性，即乙肝表面抗原（HBsAg）和乙肝表面抗体（HBsAb）阴性，乙肝核心抗体（HBcAb）阳性。因为乙肝小三阳中的HBsAg阴性，因此也被称为“乙肝表面抗原缺失型乙肝”、“乙肝表面抗原阴性乙肝”等。 乙肝小三阳是乙型肝炎病毒感染的一种表现形式，通常是慢性乙肝病毒感染的早期或稳定期的表现，不同于慢性乙肝病毒感染的其他形式，如HBsAg阳性、HBsAg阴性/HBcAb阳性和HBsAg阳性/HBeAg阳性等。尽管乙肝小三阳中的HBsAg阴性，但仍然存在HBV感染和传播的风险，因此需要加强自我防护，避免血液、性接触、母婴传播等途径的风险。此外，对于患有乙肝小三阳的患者，应该进行定期检查和随访，以便及时发现和处理可能的问题。 | Yes | Yes |  | Yes |
| 8 | Term Explanation | What is HBV-DNA | HBV-DNA refers to the genetic material (DNA) of the hepatitis B virus (HBV) that is present in the blood or other bodily fluids of people infected with HBV. HBV-DNA testing is a laboratory test used to detect and quantify the amount of HBV-DNA in a person's blood. This test is used to monitor the progression of HBV infection and the effectiveness of antiviral therapy in people with chronic HBV infection. The amount of HBV-DNA in a person's blood is known as the viral load. A higher viral load generally indicates a greater risk of liver damage and disease progression. Antiviral therapy can reduce the viral load and slow down the progression of liver disease. HBV-DNA testing is also used in the diagnosis of acute and chronic HBV infections. A positive HBV-DNA test result indicates the presence of HBV in the blood and confirms a diagnosis of HBV infection. Overall, HBV-DNA testing is an important tool in the management of HBV infection. It helps to determine a person's viral load and guide decisions about antiviral therapy and monitoring of liver function. | HBV-DNA refers to the genetic material (DNA) of the hepatitis B virus (HBV) that is found in the blood of infected individuals. The HBV-DNA test is used to measure the amount of HBV-DNA in the blood, which is an important indicator of the severity and activity of HBV infection. The test is typically used to monitor the effectiveness of treatment and to determine if the virus is active and replicating in the body. The HBV-DNA test is also used to diagnose HBV infection, especially in cases where the person has no symptoms or has a mild case of the infection. The test is often performed along with other tests, such as the hepatitis B surface antigen (HBsAg) test, to confirm a diagnosis of HBV infection. HBV-DNA levels can vary widely in people with HBV infection, from very low levels to very high levels. High levels of HBV-DNA indicate that the virus is actively replicating in the body and can increase the risk of liver damage and other complications. Antiviral medications can be used to suppress the replication of the virus and reduce the amount of HBV-DNA in the blood. Regular monitoring of HBV-DNA levels is important for people with chronic HBV infection to determine the effectiveness of treatment and to prevent long-term liver damage. | Yes | Yes |  | Yes |
| 8 | Term Explanation | HBV-DNA是什么 | HBV-DNA是指乙型肝炎病毒（HBV）的DNA分子，是乙肝病毒复制和感染的关键物质。HBV-DNA可以通过核酸检测等方法进行检测，可以反映乙肝病毒在人体内的活跃程度和繁殖水平，是评估乙肝病毒感染状态和病情严重程度的重要指标之一。 乙肝病毒在人体内的复制过程中，先经过RNA病毒的阶段，然后再通过病毒酶反转录转化为DNA分子。乙肝病毒的DNA分子可以存在于血液、肝组织、淋巴结等部位，并随时释放入血液循环系统中。乙肝病毒在人体内的繁殖水平和活跃程度越高，HBV-DNA的含量也就越高。 检测HBV-DNA的水平可以用来判断乙肝病毒感染的活跃程度和病情严重程度，是指导乙肝病毒治疗和预后判断的重要依据。在治疗乙肝病毒感染的过程中，检测HBV-DNA的水平可以帮助评估治疗效果和指导药物治疗的调整。 | HBV-DNA是指乙型肝炎病毒（HBV）的DNA分子。在HBV感染者体内，病毒会进入肝细胞内部进行复制，产生新的病毒颗粒。这些病毒颗粒可以进入血液循环，并在血液中存在多个形态，其中包括HBV-DNA。HBV-DNA是血液中HBV病毒的一种特定形式，可以通过检测血液中的HBV-DNA来确定一个人是否患有乙型肝炎病毒感染。 检测HBV-DNA的方法主要有PCR（聚合酶链式反应）法、荧光定量PCR法、DNA杂交法等。HBV-DNA检测可以用于诊断乙肝病毒感染、监测乙肝病毒感染患者的病情和治疗效果，以及指导乙肝病毒相关疾病的治疗方案。对于慢性乙肝病毒感染者，定期检测HBV-DNA可以帮助及早发现病情变化，及时调整治疗方案，控制病情进展。 | Yes | Yes |  | Yes |
| 9 | Term Explanation | NA |  |  |  |  |  |  |
| 9 | Term Explanation | HBV病毒是什么 | HBV病毒是乙型肝炎病毒（Hepatitis B Virus，简称HBV）的简称。乙肝病毒是一种双链DNA病毒，属于乙肝病毒科，是一种会引起肝脏感染和炎症的病毒。乙肝病毒可以通过血液、性接触、共用针头等途径传播，是全球范围内公共卫生领域的重大问题。 乙肝病毒具有很强的耐受性和传染性，可以在极端环境下存活多年。感染乙肝病毒的人会产生抗体和细胞免疫反应，导致肝脏炎症、肝损伤和肝硬化等疾病。严重的情况下，可能导致肝癌等恶性肿瘤的发生。 目前，预防和控制乙肝病毒的方法主要包括加强宣传教育、控制传染途径、乙肝疫苗接种等。对于已经感染乙肝病毒的人，还可以采用抗病毒治疗等方法来控制病情发展。 | HBV病毒是乙型肝炎病毒（Hepatitis B virus）的简称，是一种DNA病毒，属于肝炎病毒科。HBV病毒通过血液、性接触、母婴传播等途径感染人类，引起急性或慢性乙型肝炎。乙型肝炎是一种常见的肝脏疾病，全球有超过2亿人感染乙型肝炎病毒，其中约有3500万人患有慢性乙型肝炎。 HBV病毒是一种非常坚韧的病毒，可以在外界环境中存活长达7天，因此在预防乙型肝炎感染方面，应该注意避免血液、性接触、母婴传播等途径的风险，同时接种乙肝疫苗也是一种有效的预防措施。如果已经感染了HBV病毒，应该及时就医，遵循医生的治疗方案进行治疗，以预防疾病的进展和并发症的发生。 | Yes | Yes |  | Yes |
| 10 | Term Explanation | What is HBV cirrhosis | HBV cirrhosis is a type of liver disease caused by chronic infection with the hepatitis B virus (HBV) that results in scarring of the liver tissue. Cirrhosis is a late stage of liver disease that occurs when liver tissue is damaged and replaced by scar tissue, which can interfere with the liver's ability to function properly. In chronic HBV infection, the immune system's response to the virus can cause ongoing inflammation in the liver, which over time can lead to fibrosis, or the accumulation of scar tissue. As the fibrosis progresses, it can lead to cirrhosis. Cirrhosis can cause a range of symptoms, including fatigue, weakness, weight loss, loss of appetite, nausea, abdominal pain, jaundice, itching, and swelling of the legs and abdomen. In advanced cases, cirrhosis can lead to liver failure, portal hypertension, and an increased risk of liver cancer. Treatment for HBV cirrhosis focuses on managing the complications of the disease and preventing further liver damage. This may include antiviral medications to suppress the HBV infection, medications to reduce inflammation in the liver, and lifestyle changes such as avoiding alcohol and maintaining a healthy weight. In some cases, liver transplantation may be necessary. Prevention is key to reducing the risk of developing HBV cirrhosis. This includes getting vaccinated against HBV, practicing safe sex, not sharing needles or other equipment used for injecting drugs, and avoiding contact with blood and bodily fluids from infected individuals. | HBV cirrhosis refers to a condition in which the hepatitis B virus (HBV) causes scarring and damage to the liver, leading to the development of cirrhosis. Cirrhosis is a late-stage liver disease in which the liver becomes scarred and hardened, making it difficult for the liver to function properly. In HBV cirrhosis, the virus causes inflammation and damage to the liver cells, leading to the accumulation of scar tissue over time. As the amount of scar tissue increases, the liver becomes less able to function properly, which can lead to a range of complications such as jaundice, ascites (fluid buildup in the abdomen), and portal hypertension (high blood pressure in the veins that carry blood to the liver). People with HBV cirrhosis may not have any symptoms at first, but as the disease progresses, they may develop symptoms such as fatigue, weakness, abdominal pain, and weight loss. The diagnosis of HBV cirrhosis is typically made through blood tests, imaging studies, and sometimes a liver biopsy. Treatment for HBV cirrhosis may include antiviral medications to suppress the replication of the virus and to reduce the risk of liver damage, as well as medications to manage complications such as ascites and portal hypertension. In some cases, a liver transplant may be necessary to replace the damaged liver with a healthy one. Prevention of HBV cirrhosis includes getting vaccinated against hepatitis B, avoiding sharing needles or other equipment that could spread the virus, practicing safe sex to prevent sexual transmission of the virus, and getting tested for HBV if you are at risk of infection. | Yes | Yes |  | Yes |
| 10 | Term Explanation | 乙肝肝硬化是什么 | 乙肝肝硬化是乙型肝炎病毒（HBV）感染引起的慢性肝炎逐渐发展至肝纤维化和肝脏组织结构严重破坏，最终导致肝功能衰竭的疾病状态。在乙肝慢性感染的人群中，肝硬化是常见并发症之一。 肝硬化的发生过程是由于慢性肝炎导致肝脏组织损伤、炎症和纤维化，导致肝脏结构和功能丧失，最终形成瘢痕和结节状的肝组织。肝硬化发生后，肝脏功能逐渐减退，血液中的毒素无法被肝脏代谢清除，进一步损害身体其他器官的功能。严重时，可能会导致肝功能衰竭、肝癌等严重后果。 乙肝肝硬化的治疗需要根据患者的具体情况进行个体化治疗。治疗措施包括抗病毒治疗、肝保护治疗、营养支持等，并需要采取相应的措施预防肝功能进一步恶化和相关并发症的发生。同时，控制乙肝病毒的传播和加强健康管理也是预防和控制乙肝肝硬化的重要措施。 | 乙肝肝硬化（Hepatitis B-related cirrhosis）是由乙型肝炎病毒（HBV）感染引起的肝硬化。肝硬化是一种严重的肝脏疾病，其特征是肝脏组织的瘢痕化和纤维化，从而导致肝脏功能的逐渐丧失。 乙肝病毒感染后，部分患者会出现急性肝炎症状，而另一些患者可能没有明显症状，但仍然可能成为慢性乙型肝炎病毒感染者。长期的乙肝病毒感染会导致肝脏组织不断受到损害，细胞死亡和再生的过程导致肝脏的纤维化，最终导致肝硬化。乙肝肝硬化的主要症状包括腹胀、腹水、黄疸、肝性脑病、肝功能异常等。肝硬化是不可逆转的，但如果尽早诊断并控制疾病进程，可以延缓病情进展，减轻症状，提高生活质量。在治疗方面，包括药物治疗、营养支持、干预肝硬化的并发症、肝移植等。 | Yes | Yes |  | Yes |
| 11 | Term Explanation | What is hepatitis B virus carrier | A hepatitis B virus carrier is a person who has chronic hepatitis B infection, meaning that the virus remains in their body for an extended period, often for life. Some people who are carriers of the hepatitis B virus may not have any symptoms, while others may have ongoing symptoms such as fatigue, abdominal pain, and loss of appetite. Over time, chronic hepatitis B infection can lead to serious liver complications such as cirrhosis, liver cancer, and liver failure. The term "carrier" is sometimes used to describe people who are infected with HBV but do not have any symptoms and do not have evidence of liver damage on tests such as liver function tests or liver biopsy. These people may have the virus in their blood and be capable of transmitting it to others. Chronic carriers of the hepatitis B virus are at increased risk of transmitting the virus to others, especially through blood-to-blood contact or unprotected sexual activity. People who are carriers of HBV are also at increased risk of developing serious liver disease and require ongoing monitoring and medical care. The most effective way to prevent hepatitis B virus carrier status is to get vaccinated against HBV. Vaccination is recommended for all infants and children, as well as for adults who are at increased risk for infection with HBV, such as healthcare workers, people with multiple sexual partners, people who inject drugs, and people with chronic liver disease. | A hepatitis B virus (HBV) carrier is a person who is infected with the hepatitis B virus but does not show any symptoms of the disease. This means that the person can spread the virus to others without knowing it. In some cases, the virus can remain in the carrier's body for many years without causing any problems, but in other cases, it can lead to liver damage, liver cancer, or other serious health problems. HBV carriers can be divided into two categories: chronic carriers and inactive carriers. Chronic carriers have the virus in their bloodstream for at least six months and are at risk of developing liver damage or other complications. Inactive carriers, on the other hand, have the virus in their bloodstream, but their immune system is able to keep it under control, and they are not at risk of developing liver damage or other complications. It is important for HBV carriers to get regular medical checkups and to take steps to prevent the spread of the virus to others. This includes getting vaccinated against hepatitis B, avoiding sharing needles or other equipment that could spread the virus, and using condoms during sex to prevent sexual transmission of the virus. | Yes | Yes |  | Yes |
| 11 | Term Explanation | 乙肝病毒携带者是什么 | 乙肝病毒携带者是指血清HBsAg（乙肝表面抗原）阳性，但其他乙肝病毒血清学指标阴性，肝功能正常，无临床症状和肝脏病变的人群。也就是说，乙肝病毒携带者是一种具有潜在传染性的人群，但不一定发生肝炎和肝硬化等病变，也没有任何症状。 乙肝病毒携带者是乙肝病毒感染的一种状态，其感染途径和传播方式与其他乙肝病毒感染者相同。乙肝病毒携带者具有慢性乙肝病毒感染的风险，可能会发展为慢性乙肝病毒感染、肝炎、肝硬化和肝癌等严重疾病。此外，乙肝病毒携带者在接受血液透析、器官移植、手术和血液制品等治疗时也可能成为感染源。 对于乙肝病毒携带者，需要进行定期的肝功能检查和血清病毒学指标检测，以及采取必要的措施控制乙肝病毒传播和病情发展。乙肝病毒携带者还应该注重个人卫生和生活方式，避免接触乙肝病毒的传播途径，如避免过度饮酒、避免性行为和共用针头等。对于未感染乙肝病毒的人群，应该接种乙肝疫苗，以预防乙肝病毒感染。 | 乙肝病毒携带者指的是被乙型肝炎病毒（HBV）感染，但没有出现明显乙肝症状和异常肝功能的人。这些人的体内可能会存在HBV病毒和病毒载量，但是由于免疫系统的作用，病毒复制和繁殖的速度较慢，导致肝功能正常，也没有明显的症状表现。 乙肝病毒携带者可以分为两类：一类是急性乙肝病毒携带者，指的是HBsAg阳性持续不足6个月的人群；另一类是慢性乙肝病毒携带者，指的是HBsAg阳性持续6个月以上的人群。 乙肝病毒携带者虽然没有明显的症状，但是仍然有可能传播病毒给他人。尤其是在性行为和注射毒品等高风险行为中，乙肝病毒携带者需要特别注意，避免传播病毒给他人。同时，乙肝病毒携带者需要定期接受检查，以便及时发现和治疗乙肝病毒感染，防止病情进展。 对于乙肝病毒携带者，一般没有特殊治疗需要，但需要定期进行乙肝病毒相关指标的检查，以监测病毒水平和肝功能状况。对于病毒复制水平较高的患者，可能需要进行抗病毒治疗，以减少病毒载量和预防疾病的进展。同时，生活中也需要注意饮食和生活方式，避免过度饮酒、暴饮暴食等不良习惯，保持肝脏的健康。 | No | No |  | No |
| 12 | Term Explanation | What is occult HBV infectionn | Occult HBV infection (OBI) is a term used to describe a condition in which a person has a chronic hepatitis B virus (HBV) infection but the virus is not detectable by standard blood tests for HBV. In OBI, the HBV DNA is present in the liver cells but is not detectable in the blood using standard blood tests. People with OBI may have normal liver function tests and no symptoms of liver disease, but they are still at risk of developing liver disease over time. OBI can occur in people who have been previously infected with HBV and have cleared the virus but still have small amounts of HBV DNA in their liver cells. It can also occur in people who have chronic HBV infection but have very low levels of HBV DNA in their blood. OBI is more common in people who are immunocompromised, such as people living with HIV or those who have undergone organ transplantation. It is also more common in people who have received blood transfusions or have a history of injecting drug use. OBI is a concern because people with OBI can still transmit the virus to others, even though it is not detectable in their blood. They are also at risk of developing liver disease over time, even though their liver function tests may be normal. Treatment for OBI may involve antiviral therapy to suppress the virus and prevent the development of liver disease. Prevention measures, such as vaccination against HBV, practicing safe sex, and not sharing needles or other equipment used for injecting drugs, can help reduce the risk of OBI. | Occult HBV infection (OBI) is a condition where a person has a hidden or low-level hepatitis B virus (HBV) infection, which cannot be detected by standard blood tests for HBV. In people with OBI, HBV DNA is present in the liver or peripheral blood mononuclear cells, but the amount of virus in the bloodstream is too low to be detected by conventional HBV tests, such as the HBsAg test. People with OBI may not have any symptoms, but they can still transmit the virus to others through blood transfusions, organ transplantation, or sexual contact. OBI can also reactivate in people who are immunosuppressed, such as those with HIV or undergoing chemotherapy, leading to acute or chronic HBV infection. OBI can occur in people who have recovered from acute HBV infection, people with chronic HBV infection who have received treatment, or people who have never been infected with HBV. The exact mechanisms of OBI are not fully understood, but it is believed to be caused by mutations in the HBV DNA that allow the virus to evade the immune system and persist in the body at low levels. Diagnosis of OBI requires specialized testing to detect HBV DNA in the liver or peripheral blood mononuclear cells. Treatment for OBI is generally not recommended unless there is evidence of liver disease or evidence of HBV reactivation. Prevention of OBI includes getting vaccinated against hepatitis B, avoiding sharing needles or other equipment that could spread the virus, practicing safe sex to prevent sexual transmission of the virus, and getting tested for HBV if you are at risk of infection. | Yes | Yes |  | Yes |
| 12 | Term Explanation | 隐匿性乙肝感染是什么 | 隐匿性乙肝感染是指患者感染乙肝病毒后，虽然HBsAg（乙肝表面抗原）呈阴性，但乙肝病毒DNA在肝脏或血液中仍然存在的一种状态。与乙肝病毒携带者不同的是，隐匿性乙肝感染者同时存在乙肝病毒DNA和抗体，而病毒表面抗原（HBsAg）呈阴性。 隐匿性乙肝感染患者的肝功能常常正常，而肝组织检查也显示轻微的肝炎，但这些病变不会引起任何临床症状，也不会引起慢性肝炎、肝硬化、肝癌等并发症。然而，隐匿性乙肝感染者在一些情况下，如免疫抑制状态下、临床检查中等，可能会激活乙肝病毒的复制和感染，从而导致乙肝病毒传播和肝炎等疾病的发生。 对于隐匿性乙肝感染者，应该定期进行肝功能检查、血清病毒学指标检测和肝脏超声等检查，以评估病情和制定相应的治疗方案。尽管隐匿性乙肝感染不会引起肝炎等临床症状，但这类人群仍具有一定的传染性，应该避免通过血液、性行为、共用针头等途径传播乙肝病毒。同时，隐匿性乙肝感染者也需要加强个人卫生管理，避免过度饮酒和其他可能导致肝病发生的不良习惯。 | 隐匿性乙肝感染是指人体携带乙型肝炎病毒（HBV），但血液中HBsAg阴性、HBV-DNA阳性，且没有肝功能异常和肝炎病症状的一种乙型肝炎感染状态。也被称为“乙肝表面抗原阴性乙肝”或“乙肝非A-E型肝炎”。 隐匿性乙肝感染通常在常规体检或疾病检测中意外发现，由于HBsAg阴性，所以隐匿性乙肝感染者很难被发现。虽然患者的HBsAg阴性，但是仍然可以通过HBV-DNA的检测来确定患者是否感染了乙肝病毒。 隐匿性乙肝感染的人群通常是一些HBV携带者，他们虽然没有发生急性肝炎或慢性肝炎，但仍然具有传染性，可以通过血液、性接触、母婴传播等途径将乙肝病毒传染给他人。因此，对于发现隐匿性乙肝感染者，应该加强监测和随访，以及采取必要的措施，避免乙肝病毒的传播。 需要注意的是，隐匿性乙肝感染可能存在一定的风险，因为隐匿性乙肝感染也有可能发展成为慢性乙肝病毒感染，导致肝硬化、肝癌等严重后果。因此，隐匿性乙肝感染者需要定期进行乙肝病毒检测，以及肝功能和肝脏影像学检查等，及时发现并处理可能的问题。 | Yes | Yes |  | Yes |
| 13 | Term Explanation | What is HBV infection recovery period | The recovery period for HBV infection can vary depending on several factors, including the individual's age, overall health, and the severity of the infection. In general, the acute phase of HBV infection lasts for several weeks to several months, during which time the virus is actively replicating in the body. Symptoms of acute HBV infection may include fatigue, nausea, abdominal pain, jaundice, and dark urine. Most people with acute HBV infection recover fully within 3 to 4 months, although some may take longer. For people with chronic HBV infection, the recovery period may be much longer or may not occur at all. Chronic HBV infection is defined as the persistence of HBV DNA in the blood for more than six months. People with chronic HBV infection may not have symptoms initially, but over time, the virus can lead to serious liver disease such as cirrhosis, liver cancer, and liver failure. Treatment for HBV infection may involve antiviral therapy, which can help to suppress the virus and reduce the risk of liver damage. In some cases, people with chronic HBV infection may require ongoing treatment and monitoring to prevent complications. Prevention is key to avoiding the recovery period of HBV infection. This includes getting vaccinated against HBV, practicing safe sex, not sharing needles or other equipment used for injecting drugs, and avoiding contact with blood and bodily fluids from infected individuals. | The recovery period for HBV infection can vary depending on whether the infection is acute or chronic. Acute HBV infection is a short-term illness that usually lasts a few weeks to several months. During this time, the body's immune system works to clear the virus from the body, and most people with acute HBV infection will recover fully without any long-term effects. However, in some cases, acute HBV infection can lead to severe illness and even liver failure, which can be life-threatening. Chronic HBV infection, on the other hand, is a long-term illness that can last a lifetime. People with chronic HBV infection may not have any symptoms, but the virus can cause liver damage, cirrhosis, and liver cancer over time. There is no cure for chronic HBV infection, but antiviral medications can be used to suppress the replication of the virus and reduce the risk of liver damage. The recovery period for acute HBV infection can vary depending on the individual and the severity of the infection. In general, most people with acute HBV infection will begin to feel better within a few weeks and will recover fully within a few months. However, it can take several months for the virus to be completely cleared from the body. It is important for people who have been infected with HBV to get regular medical checkups and to follow up with their healthcare provider to monitor their liver health and to receive appropriate treatment if necessary. | Yes | Yes |  | Yes |
| 13 | Term Explanation | 乙肝感染恢复期是什么 | 乙肝感染恢复期是指患者感染乙肝病毒后，通过免疫反应逐渐清除病毒，HBsAg（乙肝表面抗原）和HBV-DNA（乙肝病毒DNA）逐渐降低至阴性，同时产生抗HBs（乙肝表面抗体）和抗HBc（乙肝核心抗体），进而恢复了正常肝功能，从而达到对乙肝病毒的免疫保护状态。 乙肝感染恢复期的时长和治疗效果与患者的年龄、病毒感染水平、免疫系统状态等因素有关。一般来说，乙肝感染恢复期的时间在数月到数年不等，个体差异较大。在恢复期期间，患者需要遵医嘱定期进行肝功能检查、血清学指标检测和肝脏超声等检查，以评估病情和制定相应的治疗方案。 对于乙肝感染恢复期的患者，需要遵循良好的生活习惯，注意肝保护，避免饮酒、过度劳累、高脂饮食等不良习惯，减轻肝脏负担。同时，定期接受医生的监测和随访，及时发现和处理乙肝病毒再活动等情况，以防止病情复发和并发症的发生。 | 乙肝感染恢复期是指患者在急性乙肝病毒感染后，体内的乙型肝炎病毒被免疫系统清除，并且肝功能恢复正常的过程。在恢复期间，患者的体温、黄疸、肝功能等指标均会逐渐恢复正常，一般需要数周或数个月的时间。  恢复期的乙肝感染患者可以通过血液检测，检测到HBsAg和HBeAg阴性，同时HBsAb和HBeAb阳性，表示体内已经没有活跃的乙肝病毒，同时产生了对乙肝病毒的抗体，具有一定的免疫保护力。恢复期的患者通常无需特殊治疗，但需要遵守医生的建议，保持良好的饮食和生活习惯，同时注意避免乙肝病毒的传播。  需要注意的是，乙肝感染的恢复期并不代表病毒被完全清除，有一定概率转变为慢性乙肝感染。因此，恢复期的患者仍需定期进行肝功能和乙肝病毒相关检测，以及接受医生的指导和治疗，以预防疾病的进展和并发症的发生。 | Yes | Yes |  | Yes |
| 14 | Term Explanation | What is acute (short-term) hepatitis B? | Acute hepatitis B is a short-term infection with the hepatitis B virus (HBV) that typically lasts for several weeks to several months. Acute hepatitis B can cause inflammation of the liver and a range of symptoms, which can vary from mild to severe. During the acute phase of HBV infection, the virus is actively replicating in the body and can be detected in the blood using standard blood tests. Symptoms of acute hepatitis B may include fatigue, nausea, abdominal pain, jaundice (yellowing of the skin and eyes), and dark urine. Most people with acute hepatitis B recover fully within 3 to 4 months, with no lasting liver damage. However, in some cases, acute hepatitis B can lead to serious complications such as liver failure, which can be life-threatening. Acute hepatitis B is typically spread through contact with infected blood or bodily fluids, such as semen and vaginal secretions. It can be spread through sexual contact, sharing needles or other equipment used for injecting drugs, from mother to baby during childbirth, or through contact with open wounds or sores of an infected person. Prevention is key to avoiding acute hepatitis B. This includes getting vaccinated against HBV, practicing safe sex, not sharing needles or other equipment used for injecting drugs, and avoiding contact with blood and bodily fluids from infected individuals. Early detection and treatment of acute hepatitis B can help prevent complications and promote a full recovery. | Acute hepatitis B is a short-term illness caused by the hepatitis B virus (HBV) that typically lasts for several weeks to several months. During this time, the body's immune system works to clear the virus from the body. Acute hepatitis B can be asymptomatic, meaning that the person infected with the virus may not experience any symptoms. However, some people with acute HBV infection may develop symptoms such as fatigue, fever, abdominal pain, nausea, vomiting, and jaundice (yellowing of the skin and eyes). Most people with acute HBV infection will recover fully without any long-term effects, although in some cases, acute HBV infection can lead to severe illness and even liver failure, which can be life-threatening. Acute hepatitis B is diagnosed through blood tests, including the hepatitis B surface antigen (HBsAg) test, which detects the presence of the virus in the blood, and the hepatitis B surface antibody (HBsAb) test, which detects the presence of antibodies against the virus. There is no specific treatment for acute hepatitis B, but supportive care can be provided to manage symptoms. Rest, adequate hydration, and a healthy diet can help the body to recover. In most cases, the body's immune system will clear the virus on its own, and people with acute hepatitis B will develop immunity to the virus. Prevention of acute hepatitis B includes getting vaccinated against hepatitis B, avoiding sharing needles or other equipment that could spread the virus, practicing safe sex to prevent sexual transmission of the virus, and getting tested for HBV if you are at risk of infection. | Yes | Yes |  | Yes |
| 14 | Term Explanation | 急性乙肝是什么 | 急性乙肝是由乙型肝炎病毒（HBV）感染引起的一种肝脏疾病。急性乙肝感染一般分为四个不同的阶段：潜伏期、前症期、黄疸期和恢复期。 在潜伏期，乙肝病毒已经进入人体，但尚未引起任何明显的症状或体征。在前症期，一些患者可能会出现轻微的症状，如乏力、食欲不振、恶心、呕吐、上腹部不适、关节疼痛等。在黄疸期，患者的黄疸症状逐渐加重，皮肤和眼睛可能变黄，尿液变暗，粪便变白等。此外，一些患者还可能会出现发热、疲劳、肝区疼痛等症状。在恢复期，患者的黄疸症状逐渐减轻，体温和肝功能逐渐恢复正常。 乙肝病毒主要通过血液、性行为和母婴传播途径感染人体，因此，预防乙肝病毒感染的关键措施包括接种乙肝疫苗、避免与乙肝病毒感染者进行性接触、避免共用针头、注意个人卫生等。对于已经感染乙肝病毒的人，及时诊断、积极治疗和科学管理，是控制乙肝疾病的关键。 | 急性乙肝是由乙型肝炎病毒（HBV）引起的一种急性肝炎，通常在感染后4至12周内出现临床症状。急性乙肝的症状包括疲劳、食欲不振、恶心、呕吐、腹泻、发热、关节疼痛、黄疸等。其中，黄疸是急性乙肝的典型症状，是由于肝细胞损伤导致的胆红素沉积在皮肤和黏膜上所致。 急性乙肝感染的传播途径主要是血液、性接触、母婴传播等途径。急性乙肝感染通常在症状出现后自行恢复，不需要特殊治疗，但在恢复期间需要注意休息，多喝水，注意饮食，避免饮酒等刺激性食物。 对于某些高危人群，如医务人员、输血受血者、有多性伴侣、注射吸毒者等，可以接种乙肝疫苗预防急性乙肝的发生。如果出现乙肝症状，应及时就医，进行相关检查和治疗。及时发现和治疗急性乙肝可以有效预防乙肝病毒引起的严重后果，如慢性乙肝、肝硬化、肝癌等。 | Yes | Yes |  | Yes |
| 15 | Term Explanation | What is chronic (long-term) hepatitis B? | Chronic hepatitis B is a long-term infection with the hepatitis B virus (HBV) that persists for more than six months. Unlike acute hepatitis B, which typically resolves within a few months, chronic hepatitis B can last for many years and can cause ongoing liver damage. During chronic hepatitis B, the immune system's response to the virus can cause ongoing inflammation in the liver, which over time can lead to fibrosis, or the accumulation of scar tissue. As the fibrosis progresses, it can lead to cirrhosis, which is a late stage of liver disease characterized by significant scarring and damage to the liver tissue. Chronic hepatitis B can also increase the risk of liver cancer. Most people with chronic hepatitis B do not have symptoms initially, but over time, they may experience fatigue, abdominal pain, loss of appetite, and other symptoms of liver disease. Chronic hepatitis B can be diagnosed through blood tests that detect the presence of HBV DNA in the blood. Treatment for chronic hepatitis B may involve antiviral medications, which can suppress the virus and reduce the risk of liver damage. Regular monitoring of liver function and imaging studies may be necessary to monitor for complications such as cirrhosis or liver cancer. In some cases, liver transplantation may be necessary. Prevention is key to avoiding chronic hepatitis B. This includes getting vaccinated against HBV, practicing safe sex, not sharing needles or other equipment used for injecting drugs, and avoiding contact with blood and bodily fluids from infected individuals. Early detection and treatment of acute hepatitis B can also help prevent the development of chronic hepatitis B. | Chronic hepatitis B is a long-term illness caused by the hepatitis B virus (HBV) that can lead to serious liver damage over time. Chronic hepatitis B occurs when the virus remains in the body for more than six months after the initial infection. Chronic hepatitis B can be asymptomatic, meaning that the person infected with the virus may not experience any symptoms. However, over time, chronic hepatitis B can cause liver damage, cirrhosis (scarring of the liver), and even liver cancer. Symptoms of chronic hepatitis B may include fatigue, abdominal pain, loss of appetite, nausea and vomiting, dark urine, pale stools, and yellowing of the skin and eyes (jaundice). Chronic hepatitis B can also lead to liver failure, which is a life-threatening condition. Treatment for chronic hepatitis B aims to reduce the risk of liver damage and slow down the progression of the disease. Antiviral medications can be used to suppress the replication of the virus and reduce inflammation in the liver. Regular monitoring of liver function and regular screenings for liver cancer are also important for people with chronic hepatitis B. Prevention of chronic hepatitis B includes getting vaccinated against hepatitis B, avoiding sharing needles or other equipment that could spread the virus, practicing safe sex to prevent sexual transmission of the virus, and getting tested for HBV if you are at risk of infection. | Yes | Yes |  | Yes |
| 15 | Term Explanation | 慢性乙肝是什么 | 慢性乙肝是由乙型肝炎病毒（HBV）感染引起的一种肝脏疾病，病程较长，可能持续数十年。与急性乙肝不同，慢性乙肝病毒持续存在于患者体内，并且可能引起肝炎、肝硬化和肝癌等严重并发症。 慢性乙肝的发生和发展与个体的免疫反应和乙肝病毒的感染水平有关。在乙肝病毒感染后，患者的免疫系统会产生免疫反应，以清除病毒，但在一些患者中，免疫反应可能不足或存在免疫耐受现象，使得乙肝病毒得以长期存在于肝细胞中，逐渐引起肝脏组织损伤和炎症反应。慢性乙肝的病情程度可以因患者的年龄、乙肝病毒感染水平、免疫系统状态等因素而异。 慢性乙肝的临床症状和体征不太明显，可能包括乏力、食欲不振、恶心、呕吐、上腹部不适、肝区疼痛、黄疸等。因此，慢性乙肝病毒的诊断需要通过肝功能检查、血清学指标检测、肝脏超声等多种方法来进行评估。 对于慢性乙肝患者，需要根据具体情况进行个体化治疗。抗病毒治疗是治疗慢性乙肝的主要方法，可以有效地抑制乙肝病毒的复制和繁殖，减少肝脏炎症和纤维化，降低肝硬化和肝癌的发生风险。同时，患者也需要遵循良好的生活习惯和饮食习惯，注意肝保护，避免饮酒、过度劳累等不良习惯，减轻肝脏负担。对于慢性乙肝病毒感染者，还需要接受定期的监测和随访，及时发现和处理病情变化，预防并发症的发生。对于慢性乙肝病毒感染者，还应该遵循个人防护措施，避免通过血液、性行为、共用针头等途径传播乙肝病毒，以防止乙肝病毒的传播。 | 慢性乙肝是由乙型肝炎病毒（HBV）引起的一种肝炎，通常是指HBsAg阳性持续6个月以上的肝炎状态。与急性乙肝不同，慢性乙肝病毒可以在人体内长期存在而不被清除，持续对肝脏造成损害和炎症，导致肝脏逐渐受损。 慢性乙肝的症状通常不明显，患者可能会出现疲劳、食欲不振、肝区不适、黄疸等轻度症状，也可能没有症状表现。慢性乙肝的病程较长，可能持续数十年，而在这段时间内，肝脏可能逐渐受损，甚至发展成肝硬化和肝癌等严重疾病。 慢性乙肝的治疗需要依据患者的肝功能状况、病毒水平、肝组织病理学改变等因素来确定治疗方案。一般治疗方法包括抗病毒治疗、免疫调节治疗、肝保护治疗等，对于肝硬化和肝癌等并发症，也需要进行相应的治疗。 预防是预防慢性乙肝的最好方法，乙肝疫苗是预防乙肝的有效途径。尤其是在新生儿期接种乙肝疫苗，可以大大降低慢性乙肝的发生率。同时，避免接触血液和其他体液、使用安全套等也是预防乙肝感染的重要措施。 | Yes | Yes |  | Yes |
| 16 | Term Explanation | What is hepatitis B immune globulin (HBIG) | Hepatitis B immune globulin (HBIG) is a medication made from the blood of people who have been vaccinated against hepatitis B or who have recovered from a past infection with the hepatitis B virus (HBV). HBIG contains antibodies to the hepatitis B virus, which can help prevent or reduce the severity of HBV infection. It is used in several situations, including: Prevention of mother-to-child transmission of HBV: Infants born to mothers who are infected with HBV may be given HBIG within 12 hours of birth, along with the hepatitis B vaccine, to help prevent transmission of the virus from the mother to the baby. Post-exposure prophylaxis: People who have been exposed to HBV, such as through sexual contact or needlestick injuries, may be given HBIG as a preventive measure, along with the hepatitis B vaccine. Treatment of acute HBV infection: In rare cases, HBIG may be used to treat acute HBV infection, although it is not always effective in this situation. HBIG is given as an injection into a muscle or vein. The dose and frequency of HBIG administration depends on the specific situation and the individual's age and weight. HBIG is considered safe and effective when used appropriately. However, like all medications, it can cause side effects, such as pain at the injection site, fever, headache, and nausea. Rarely, more serious side effects such as an allergic reaction may occur. | Hepatitis B immune globulin (HBIG) is a blood product that contains antibodies against the hepatitis B virus (HBV). HBIG is used to provide passive immunity to people who have been exposed to HBV, particularly those at high risk of developing acute hepatitis B infection. HBIG works by providing a temporary supply of HBV-specific antibodies to the body, which can help to prevent the virus from infecting liver cells and causing further damage. HBIG is typically given as an injection into a muscle or vein, either alone or in combination with the hepatitis B vaccine. HBIG is used in a variety of settings, including: 1.Post-exposure prophylaxis: HBIG is given to people who have been exposed to HBV through a needlestick injury, sexual contact with an infected person, or other high-risk activities to prevent or reduce the severity of acute HBV infection. 2.Perinatal prophylaxis: HBIG is given to infants born to mothers with HBV to prevent mother-to-child transmission of the virus. 3.Liver transplantation: HBIG is given to people who have received a liver transplant from an HBV-infected donor to prevent the transmission of the virus from the donor organ. Side effects of HBIG are generally mild and can include soreness, redness, or swelling at the injection site, fever, and headache. Rarely, more serious allergic reactions can occur. HBIG is an important tool in the prevention and management of HBV infection, particularly in high-risk populations. It is typically used in combination with the hepatitis B vaccine to provide both passive and active immunity to HBV. | No | No |  | No |
| 16 | Term Explanation | 乙肝免疫球蛋白是什么 | 乙肝免疫球蛋白（HBIG）是一种由乙型肝炎病毒（HBV）表面抗原（HBsAg）制备的免疫制剂，主要用于预防和治疗乙肝病毒感染。HBIG含有高滴度的抗体，可以在接触乙肝病毒时提供即刻的免疫保护，防止病毒侵入人体，从而减少乙肝病毒的感染风险。 HBIG主要用于以下几个方面： 1.预防乙肝病毒感染：在高风险人群中接种乙肝免疫球蛋白可以提供即刻免疫保护，减少乙肝病毒感染的风险，如接触乙肝病毒患者、接受输血和器官移植等手术前后等情况。 2.预防母婴乙肝传播：对于乙肝病毒携带者的孕妇，产前接种乙肝免疫球蛋白可以有效预防母婴乙肝传播。 3.治疗乙肝病毒感染：对于急性乙肝感染和一些特定的慢性乙肝感染患者，使用乙肝免疫球蛋白可以提供即刻的被动免疫保护，降低病毒复制水平，减轻肝脏炎症反应，促进肝功能恢复。 值得注意的是，乙肝免疫球蛋白并不能永久性预防乙肝病毒感染，其提供的免疫保护只有暂时性的作用，通常只能维持数周至数个月的时间。对于需要长期保护的人群，建议接种乙肝疫苗来建立持久性免疫保护。 | 乙肝免疫球蛋白是一种通过采集献血者血浆并进行纯化、加工后制成的免疫球蛋白制剂，含有高浓度的乙肝表面抗体（HBsAb），可以用于预防和治疗乙型肝炎病毒（HBV）感染。 乙肝免疫球蛋白通过静脉注射的方式，将高浓度的HBsAb直接注入到接触过HBV的人体内，从而起到立即的被动免疫保护作用。乙肝免疫球蛋白可以在短时间内提供较高的抗体水平，预防HBV感染或减轻病情，对于免疫系统功能低下或无法接种乙肝疫苗的人群特别有效。 乙肝免疫球蛋白可以应用于下列情况： 1.非免疫人群接触HBV感染源后需要快速预防，例如：新生儿、医疗保健工作者等。 2.对于患有慢性乙肝病的人群，可以作为辅助治疗，提高患者的免疫水平。 3.对于乙肝病毒携带者的亲属、同住人员等，可以应用乙肝免疫球蛋白预防感染。 需要注意的是，乙肝免疫球蛋白并不能完全替代乙肝疫苗，乙肝免疫球蛋白的保护作用是短暂的，一般持续时间在3-6个月之间，因此需要定期注射或接种乙肝疫苗。同时，对于已经患有乙型肝炎的患者，乙肝免疫球蛋白并不能治愈乙肝，需要进行其他治疗措施。 | Yes | Yes |  | Yes |
| 17 | Term Explanation | What is hepatitis B/hepatitis D coinfection | Hepatitis B/hepatitis D coinfection refers to a condition in which a person is infected with both the hepatitis B virus (HBV) and the hepatitis D virus (HDV) at the same time. Hepatitis D, also known as delta hepatitis, is a type of liver disease caused by the hepatitis D virus. HDV can only infect people who are already infected with HBV, as it requires HBV to replicate and cause disease. HDV can either infect people who are already chronically infected with HBV or can cause acute hepatitis in people who are acutely infected with HBV. Hepatitis B/hepatitis D coinfection can cause more severe liver disease and a higher risk of liver complications than HBV infection alone. Coinfection with HDV can also increase the risk of liver cancer. The symptoms of hepatitis B/hepatitis D coinfection are similar to those of HBV infection alone and can include fatigue, abdominal pain, nausea, jaundice, and dark urine. Diagnosis of hepatitis B/hepatitis D coinfection requires specific blood tests for both HBV and HDV. Treatment for hepatitis B/hepatitis D coinfection may include antiviral medications to suppress both viruses, as well as management of any liver complications. Vaccination against HBV is also recommended for people with hepatitis B/hepatitis D coinfection who have not already been vaccinated. Prevention is key to avoiding hepatitis B/hepatitis D coinfection. This includes getting vaccinated against HBV, practicing safe sex, not sharing needles or other equipment used for injecting drugs, and avoiding contact with blood and bodily fluids from infected individuals. | Hepatitis B/hepatitis D coinfection occurs when a person is infected with both the hepatitis B virus (HBV) and the hepatitis D virus (HDV) at the same time. HDV is a small RNA virus that requires HBV to replicate and cause an infection. Therefore, HDV can only infect individuals who are already infected with HBV. Coinfection with HDV can make HBV infection more severe and increase the risk of liver damage, cirrhosis, and liver cancer. The symptoms of hepatitis B/hepatitis D coinfection can vary from mild to severe and can include fatigue, abdominal pain, jaundice, and fever. However, some people with coinfection may not have any symptoms at all. The diagnosis of hepatitis B/hepatitis D coinfection is usually made by testing for both viruses in the blood. Treatment for coinfection may include antiviral medications to suppress the replication of HBV and/or HDV, and in some cases, pegylated interferon may also be used. Vaccination against hepatitis A and B is also recommended for people with coinfection to prevent further liver damage. Prevention of hepatitis B/hepatitis D coinfection includes getting vaccinated against hepatitis B, avoiding sharing needles or other equipment that could spread the virus, and practicing safe sex to prevent sexual transmission of the virus. People who have chronic hepatitis B should also be screened for hepatitis D to determine if they have a co-infection. | Yes | Yes |  | Yes |
| 17 | Term Explanation | 乙肝和丁肝共感染是什么 | 乙肝和丁肝共感染是指同时感染乙型肝炎病毒（HBV）和丁型肝炎病毒（HDV）的情况。丁型肝炎病毒是一种由RNA病毒引起的肝炎病毒，需要HBV作为宿主才能生存和繁殖。因此，只有乙肝病毒感染者才有可能发生丁肝病毒感染。 乙肝和丁肝共感染可能会加重肝脏损伤和炎症反应，导致肝炎、肝硬化、肝衰竭和肝癌等严重并发症的发生。与单纯乙肝病毒感染相比，乙肝和丁肝共感染患者的肝病进展更快，预后也更差。因此，乙肝和丁肝共感染的诊断和治疗十分重要。 乙肝和丁肝共感染的诊断可以通过检测血清中的丁肝抗体和核酸，以及乙肝表面抗原、抗体和核酸等指标来确定。对于乙肝和丁肝共感染的患者，应该采取综合治疗措施，包括使用抗病毒药物控制病毒复制、维持免疫系统稳定、控制肝炎炎症、减轻肝硬化和肝癌等并发症的发生。此外，患者还需要注意饮食和生活习惯，积极预防和处理感染相关的并发症和合并症。 | 乙肝和丁肝共感染是指一个人同时感染了乙型肝炎病毒（HBV）和丁型肝炎病毒（HDV）的情况。HDV病毒需要依附于HBV病毒才能感染人体，因此只有在HBV感染的基础上才会发生HDV感染，HDV感染只会发生在HBsAg阳性的人群中。 乙肝和丁肝共感染相对较少见，但是危害较大，因为丁肝病毒通常会加重乙肝病情，加速肝病的进展，同时丁肝病毒的治疗相对乙肝病毒更为困难。 乙肝和丁肝共感染的症状和表现与单纯的乙肝或丁肝感染有所不同，可能包括发热、黄疸、恶心、呕吐、腹泻、肝功能异常、肝硬化等。如果怀疑自己存在乙肝和丁肝共感染的风险，应该及时就医，进行相关检查和治疗。 治疗乙肝和丁肝共感染通常需要综合治疗，包括抗病毒治疗、免疫调节治疗、肝保护治疗等。治疗方案需要根据患者的具体情况制定，包括肝病的程度、肝功能的状态、病毒载量、免疫状态等等。及时有效的治疗可以改善患者的预后和生活质量，降低并发症和肝炎相关死亡的风险。 | Yes | Yes |  | Yes |
| 18 | Short Answer Questions | List common symptoms of acute (short-term) hepatitis B | Acute hepatitis B is a viral infection that affects the liver. The symptoms of acute hepatitis B can vary in severity and may include: 1.Jaundice (yellowing of the skin and whites of the eyes) 2.Fatigue 3.Abdominal pain 4.Loss of appetite 5.Nausea and vomiting 6.Joint pain 7.Dark urine 8.Pale stool 9.Fever 10.Headache 11.Muscle aches Not everyone with acute hepatitis B will experience all of these symptoms. In some cases, people may not have any symptoms at all. It is important to get tested if you think you may have been exposed to the hepatitis B virus, as early diagnosis and treatment can help prevent serious liver damage. | Acute hepatitis B is a short-term infection of the liver caused by the hepatitis B virus (HBV). The symptoms of acute hepatitis B can vary from person to person, and some people may not experience any symptoms at all. However, some common symptoms of acute hepatitis B include: 1.Fatigue and weakness 2.Loss of appetite 3.Nausea and vomiting 4.Abdominal pain, especially in the area of the liver 5.Jaundice (yellowing of the skin and eyes) 6.Dark urine 7.Pale-colored stools 8.Joint pain 9.Low-grade fever These symptoms typically appear one to six months after exposure to the virus and can last for several weeks to months. In some cases, acute hepatitis B can lead to chronic hepatitis B if the virus is not cleared from the body within six months. If you experience any of these symptoms or think you may have been exposed to hepatitis B, it's important to speak with a healthcare provider and get tested for the virus. Early diagnosis and treatment can help prevent liver damage and the progression of the infection to chronic hepatitis B. | No | Yes | No | No |
| 18 | Term Explanation | 急性乙肝常见症状有哪些 | 急性乙肝是由乙型肝炎病毒引起的肝炎病毒性感染，其常见症状包括： 1. 乏力、疲劳感和身体不适。 2. 肝区疼痛或不适感，尤其是右上腹疼痛。 3. 食欲不振、恶心、呕吐、腹泻、便秘等胃肠道症状。 4. 黄疸，即皮肤、白眼球和黏膜等部位发黄。 5. 尿液变深、便便颜色变浅。 6. 体温升高，出现发热、寒战等症状。 7. 头痛、头晕、嗜睡等神经系统症状。 8. 关节痛和肌肉疼痛。 需要注意的是，有些患者可能没有任何症状，或者症状非常轻微，因此需要定期检查以及及时进行病毒检测。如果您出现这些症状，请尽快就医。 | 急性乙肝的症状因个体差异而有所不同，一些人可能无症状或症状非常轻微，而其他人则可能出现以下一些常见症状： 1. 乏力和疲劳感：这是急性乙肝最常见的症状之一，患者可能会感到非常疲倦和虚弱。 2. 黄疸：患者可能会出现皮肤和眼白发黄的症状，这是由于肝脏受损造成胆汁淤积的结果。 3. 恶心和呕吐：患者可能会感到恶心和呕吐，尤其在进食时可能会感到不适。 4. 腹部不适：患者可能会出现上腹部不适和疼痛，可能伴随着肝区不适感。 5. 发热和头痛：患者可能会出现发热和头痛等症状，尤其在病情严重时。 6. 关节疼痛：少数患者可能会出现关节疼痛和肌肉疼痛等症状。 需要指出的是，急性乙肝的症状通常会在感染后数天到数周内出现，严重程度和持续时间因人而异。如果有疑似急性乙肝的情况，应及早就医，接受专业的检查和诊断，以便尽早治疗和控制病情。 | No | Yes | No | No |
| 19 | Short Answer Questions | List common symptoms of chronic (long-term) hepatitis B | Chronic hepatitis B is a long-term viral infection of the liver. Many people with chronic hepatitis B may not experience any symptoms, particularly in the early stages of the infection. However, as the infection progresses, some people may develop symptoms such as: 1.Fatigue and weakness 2.Loss of appetite 3.Nausea and vomiting 4.Abdominal pain and discomfort 5.Jaundice (yellowing of the skin and whites of the eyes) 6.Itching 7.Dark urine 8.Pale stool 9.Joint pain 10.Muscle aches 11.Fluid accumulation in the abdomen (ascites) 12.Swelling of the legs and ankles (edema) 13.Spider-like blood vessels on the skin (spider angiomas) 14.Easy bruising and bleeding 15.Confusion and forgetfulness (hepatic encephalopathy) It is important to note that some people with chronic hepatitis B may not experience any symptoms at all, and may only find out they have the infection through routine blood tests. Regular monitoring and treatment can help prevent serious liver damage in people with chronic hepatitis B. | Chronic hepatitis B (CHB) is a viral infection of the liver that can cause inflammation and damage to the liver over time. Many people with CHB have no symptoms in the early stages of the disease, but as the disease progresses, symptoms may develop. Here are some common symptoms of chronic hepatitis B: 1. Fatigue: Fatigue is a common symptom of CHB, and it may be the first symptom that people notice. As the liver becomes more damaged, it may not be able to produce enough energy to support the body's needs, leading to fatigue and weakness. 2. Abdominal pain: In more advanced stages of CHB, you may experience pain or discomfort in the upper right side of the abdomen, where the liver is located. 3. Loss of appetite: As the liver becomes more damaged, you may lose your appetite and have trouble eating. This can lead to weight loss and malnutrition. 4. Nausea and vomiting: Nausea and vomiting are common symptoms of CHB, especially in more advanced stages of the disease. These symptoms can be caused by the buildup of toxins in the body due to liver dysfunction. 5. Jaundice: Jaundice is a yellowing of the skin and eyes that can occur when the liver is not functioning properly. It is caused by the buildup of bilirubin in the body. 6. Swelling in the legs and feet: Swelling in the legs and feet (edema) can occur in more advanced stages of CHB, as the liver is unable to produce enough protein to maintain fluid balance in the body. 7. Itchy skin: Itchy skin is a common symptom of CHB, which is caused by the buildup of bile salts in the body. It's important to speak with a healthcare provider if you experience any symptoms of liver disease, including chronic hepatitis B symptoms. Early diagnosis and treatment can help prevent liver damage and the progression of the disease. | No | No | No | No |
| 19 | Short Answer Questions | 慢性乙肝常见症状有哪些 | 慢性乙肝是乙型肝炎病毒长期感染肝脏而导致的慢性肝炎病变。其常见症状包括： 1. 肝区不适感或疼痛，尤其是右上腹部疼痛。 2. 乏力、疲劳、身体不适感。 3. 食欲不振、恶心、呕吐、腹泻、便秘等胃肠道症状。 4. 黄疸，即皮肤、白眼球和黏膜等部位发黄。 5. 腹水，即腹部积液，导致腹部胀大和不适。 6. 体重下降，食欲减退等症状。 7. 血小板计数减少，可能导致出血倾向。 8. 肝功能异常，如肝酶升高、肝功能减退等。 9. 肝硬化，表现为腹水、黄疸、肝功能不全等症状。 10. 肝癌，长期慢性乙肝感染是导致肝癌的重要危险因素之一。 需要注意的是，有些患者可能没有任何症状，因此需要定期检查以及及时进行病毒检测。如果您出现这些症状，请尽快就医。 | 慢性乙肝的症状因患者个体差异而有所不同，有些患者可能没有症状或仅表现为轻微的不适，但大多数患者会表现出以下一些症状： 1. 乏力和疲劳感：这是慢性乙肝最常见的症状之一，患者可能会感觉非常疲倦和乏力。 2. 食欲不振和消化问题：患者可能会感到恶心、呕吐、上腹部不适、食欲减退等症状。 3. 黄疸：患者可能会出现皮肤和眼白发黄的症状，这是由于肝脏受损造成胆汁淤积的结果。 4. 腹水：在肝硬化晚期，患者可能会出现腹部肿胀和腹水的症状。 5. 肝区疼痛：患者可能会感到右上腹部或肝区不适或疼痛。 6. 肝功能异常：慢性乙肝会导致肝功能异常，例如肝酶升高、肝功能失常等，这些可能在检查中被发现。 需要指出的是，慢性乙肝的症状往往不典型，不同患者的症状表现可能存在差异，因此及早进行检查和诊断是非常重要的。如果有疑似慢性乙肝的情况，应及早就医，接受专业的检查和诊断。 | Yes | No | No | No |
| 20 | Short Answer Questions | List transmission routes of hepatitis B | Hepatitis B is a viral infection that spreads through contact with blood or other bodily fluids of an infected person. The most common routes of transmission of hepatitis B are: 1.Sexual contact: The virus can be transmitted through unprotected sexual contact with an infected person, including vaginal, anal, and oral sex. 2.Sharing of needles and other injection equipment: The virus can be transmitted through the sharing of needles, syringes, or other injection equipment contaminated with the blood of an infected person. 3.Mother-to-child transmission: The virus can be transmitted from an infected mother to her baby during childbirth or through breastfeeding. 4.Exposure to infected blood: The virus can be transmitted through exposure to infected blood or other bodily fluids, such as during medical procedures, tattooing, body piercing, or sharing personal items like razors or toothbrushes. 5.Occupational exposure: Healthcare workers and others who come into contact with blood or bodily fluids in their work are at risk of contracting the virus. It is important to take preventive measures to avoid the transmission of hepatitis B, such as getting vaccinated, practicing safe sex, avoiding sharing needles and other injection equipment, and using precautions to avoid exposure to infected blood and bodily fluids. | Hepatitis B is a viral infection that affects the liver and is transmitted through contact with infected blood, semen, or other bodily fluids. The most common routes of transmission for hepatitis B are: 1.Unprotected sex: Hepatitis B can be spread through unprotected sex with an infected person. This includes vaginal, anal, and oral sex. 2.Sharing of needles: Hepatitis B can be spread through the sharing of needles or other drug injection equipment. 3.Mother-to-child transmission: Pregnant women infected with hepatitis B can pass the virus to their babies during delivery or shortly after birth. Infants born to mothers with hepatitis B are at high risk of developing chronic hepatitis B. 4.Blood transfusions: Hepatitis B can be spread through blood transfusions from an infected donor. However, the risk of transmission through blood transfusions is very low in most countries because donated blood is routinely screened for hepatitis B. 5.Accidental needlesticks: Healthcare workers and others who come into contact with infected blood or bodily fluids can be at risk of accidental needlesticks or other sharps injuries that can lead to hepatitis B infection. 6.Sharing personal hygiene items: Sharing personal hygiene items, such as razors or toothbrushes, can also lead to hepatitis B transmission if the items are contaminated with infected blood. It's important to take precautions to prevent hepatitis B transmission, such as practicing safe sex, not sharing needles or other drug injection equipment, and getting vaccinated against hepatitis B. Vaccination is the best way to prevent hepatitis B infection. | Yes | Yes |  | Yes |
| 20 | Short Answer Questions | 乙肝传播途径有哪些 | 乙肝的传播途径主要包括以下几种： 1. 血液传播：接触感染者的血液、血制品、注射器、手术器械等被污染的物品，或性行为、母婴传播等途径都可能引起感染。 2. 母婴传播：乙肝病毒可以通过母体传给胎儿，或在分娩时从母体感染新生儿。 3. 采血、输血及器官移植传播：在采血、输血或器官移植过程中，如果使用了感染乙肝病毒的血液或器官，则易被感染。 4. 医疗器械传播：由于乙肝病毒较为稳定，所以在医疗过程中的注射器、手术器械等可能存在污染和传播的风险。 5. 性传播：性接触是乙肝病毒传播的重要途径之一，尤其是高危人群。 6. 个人卫生不良：包括共用刮胡刀、牙刷等个人物品，或者使用其他人的注射器、刺青工具等容易感染病毒。 预防乙肝病毒的传播，可以注重个人卫生、规范医疗操作、提高自我防范意识等措施。同时，定期进行乙肝病毒筛查，及时发现和治疗感染，也是非常重要的。 | 乙肝病毒的传播途径主要有以下几种： 1. 血液传播：乙肝病毒可以通过血液传播途径感染人体，如输血、血制品、血透、血浆、器官移植、注射毒品等。 2. 性传播：乙肝病毒也可以通过性行为传播，如多个性伴侣、同性恋、不安全性行为等。 3. 母婴传播：乙肝病毒可以通过母婴传播途径感染胎儿或新生儿，如孕妇携带乙肝病毒、分娩时感染、哺乳期间传播等。 4. 医疗器械感染：未经消毒的医疗器械如注射器、刀具、针头等可能成为乙肝病毒的携带者，从而感染其他人。 5. 个人卫生习惯不良：如共用牙刷、剃须刀、针头等个人卫生用品，以及共用餐具、杯子、毛巾等等，也可能成为乙肝病毒的传播途径。 需要注意的是，乙肝病毒可以在潜伏期和急性期均可感染他人，即使感染者没有任何症状，也能够传播病毒。因此，人们需要加强个人卫生习惯，避免不必要的接触，如避免使用未消毒的医疗器械、使用安全套等，同时还需要接种乙肝疫苗，以有效预防乙肝病毒的感染和传播。 | No | Yes | Yes | Yes |
| 21 | Short Answer Questions | List treatment methods for hepatitis B | The treatment for hepatitis B depends on whether the infection is acute or chronic, and the severity of the disease. Here are some common methods for treating hepatitis B: 1.Antiviral medications: Antiviral medications can help reduce the amount of virus in the body, slow down the progression of the disease, and prevent liver damage. These medications include entecavir, tenofovir, lamivudine, and adefovir. 2.Interferon therapy: Interferon therapy is a type of treatment that involves injecting interferon into the body to boost the immune system and fight the virus. It is usually used to treat chronic hepatitis B and has some side effects. 3.Liver transplant: In severe cases of hepatitis B, liver transplantation may be necessary if the liver is severely damaged. 4.Lifestyle changes: Patients with hepatitis B should avoid alcohol, maintain a healthy diet, and get regular exercise. They should also avoid certain medications and supplements that may be harmful to the liver. 5.Vaccination: Vaccination is the best way to prevent hepatitis B infection, and it is recommended for everyone, especially those at high risk of contracting the virus. It is important to consult a healthcare provider for the appropriate treatment plan for hepatitis B as they can provide tailored recommendations based on the patient's individual needs and medical history. | The treatment for hepatitis B depends on whether the infection is acute or chronic. Acute hepatitis B infection usually does not require treatment and resolves on its own in a few weeks to months. However, chronic hepatitis B infection may require long-term treatment to manage the virus and prevent liver damage. The following are some of the methods for treating chronic hepatitis B: 1.Antiviral medication: There are several antiviral medications available that can help suppress the hepatitis B virus and prevent liver damage. These medications include entecavir, tenofovir, lamivudine, and adefovir. 2.Interferon injections: Interferon injections can help boost the immune system to fight the virus. However, this treatment is typically reserved for people with more severe cases of chronic hepatitis B. 3.Liver transplant: In rare cases where chronic hepatitis B has caused severe liver damage, a liver transplant may be necessary. 4.Lifestyle changes: Maintaining a healthy lifestyle can help manage chronic hepatitis B. This includes eating a healthy diet, avoiding alcohol, and getting regular exercise. It's essential to speak with a healthcare provider about the most appropriate treatment options for your specific case of chronic hepatitis B. Treatment may be long-term, and it's important to follow the treatment plan recommended by your healthcare provider to manage the virus and prevent liver damage. | No | Yes | No | No |
| 21 | Short Answer Questions | 乙肝治疗方法有哪些 | 乙肝的治疗方法根据病情和病程的不同而有所差异。以下是常见的乙肝治疗方法： 1. 抗病毒治疗：抗病毒治疗是乙肝治疗的主要方法，通过使用抗病毒药物（如恩替卡韦、阿德福韦等）来抑制乙肝病毒复制，减少肝脏病变，避免疾病的进展。 2. 免疫调节治疗：免疫调节治疗是利用免疫调节药物来调节机体免疫功能，提高机体对乙肝病毒的免疫力，从而减少病毒复制和肝脏损伤。常用药物包括干扰素和鸟苷酸等。 3. 对症支持治疗：包括补液、补充维生素、营养支持、对症处理肝脏病变所引起的症状，如黄疸、腹水等。 4. 肝移植：对于极少数病情严重、药物治疗无效的患者，可能需要进行肝移植手术来解决病情。 需要注意的是，不同类型和不同病程的乙肝需要不同的治疗方法，因此，患者应根据医生的建议制定个人化的治疗方案，并定期复查、监测病情。此外，积极预防乙肝病毒的感染，也是预防和治疗乙肝的重要措施之一。 | 乙肝的治疗方法包括药物治疗和手术治疗两种。 药物治疗：目前可以用于乙肝治疗的药物主要有抗病毒药物和免疫调节剂。抗病毒药物可以直接作用于乙肝病毒，抑制病毒复制，达到抑制病毒复制和减轻肝损伤的效果。常用的抗病毒药物包括阿德福韦、恩替卡韦、替比夫定等。免疫调节剂主要是通过调节免疫系统来达到控制乙肝病毒复制的效果，常用的免疫调节剂包括干扰素和核苷类似物。 手术治疗：主要是针对乙肝肝硬化、肝癌等并发症而进行的手术治疗。如肝移植、肝切除、肝动脉栓塞术等。 需要指出的是，不同乙肝患者的病情和身体情况不同，治疗方法应该根据医生的建议和具体情况选择，同时需要坚持治疗，遵守医嘱，定期复查以评估疗效。 | No | No |  | No |
| 22 | Short Answer Questions | Briefly introduce structure of the hepatitis B virus | Hepatitis B virus (HBV) is a small, enveloped virus that belongs to the family Hepadnaviridae. The virus is spherical in shape and measures about 42 nanometers (nm) in diameter. The structure of HBV consists of three main parts: 1.Outer envelope: The outer envelope of HBV is made up of a lipid bilayer, which is studded with surface proteins called hepatitis B surface antigens (HBsAg). These surface antigens play a crucial role in the attachment of the virus to host cells and in the immune response to the virus. 2.Core: The core of HBV is composed of protein subunits called core antigens (HBcAg). The core encapsidates the viral genome, which consists of a circular, partially double-stranded DNA molecule. 3.Enzymes: HBV also contains enzymes that are required for viral replication, including reverse transcriptase, which converts the viral RNA genome into DNA, and a DNA polymerase, which synthesizes new DNA strands. The structure of HBV is important in understanding how the virus infects and replicates in host cells, and in the development of vaccines and antiviral therapies. | The hepatitis B virus (HBV) is a small, enveloped DNA virus that belongs to the Hepadnaviridae family. The HBV virion consists of several components, including: 1.Capsid: The capsid is a protein shell that surrounds the viral genome. It is composed of 180 or more copies of the core antigen (HBcAg) and encloses the viral DNA. 2.Envelope: The envelope is a lipid membrane that surrounds the capsid and is derived from the host cell membrane. The envelope contains the surface antigen (HBsAg), which is the major antigenic determinant of the virus and is used for serological testing and vaccination. 3.DNA genome: The viral genome is a partially double-stranded circular DNA molecule that is about 3.2 kilobases in size. The viral DNA encodes four major proteins: the surface antigen (HBsAg), the core antigen (HBcAg), the polymerase, and the X protein. 4.Polymerase: The polymerase is an enzyme that is responsible for the replication of the viral genome. It is encoded by the viral DNA and is located within the capsid. 5.X protein: The X protein is a regulatory protein that is encoded by the viral DNA. It has multiple functions, including regulation of viral replication, modulation of host cell signaling pathways, and interference with host cell DNA repair mechanisms. The HBV virion is about 42 nanometers in diameter and is non-enveloped in its mature form. However, during viral replication, the immature virion acquires an envelope by budding through the host cell membrane. The HBV virion is highly infectious and can be transmitted through contact with infected blood or other bodily fluids. | Yes | Yes |  | Yes |
| 22 | Short Answer Questions | 简述乙肝病毒结构 | 乙肝病毒是一种双链DNA病毒，属于乙型肝炎病毒科。其结构分为外壳层、内核层和病毒DNA组成三个部分。 1. 外壳层：由表面抗原（HBsAg）组成，是乙肝病毒最外层的结构。HBsAg分为3种不同的抗原型，分别为adw、ayw和adr。 2. 内核层：由核心抗原（HBcAg）组成，是乙肝病毒的中间层结构。HBcAg包括两种类型：IgM型和IgG型。 3. 病毒DNA：乙肝病毒的遗传物质，位于病毒的内核层内。乙肝病毒的DNA分为两种形式：rcDNA和cccDNA。其中rcDNA是病毒复制过程中的中间产物，而cccDNA是乙肝病毒的主要遗传物质，嵌入宿主细胞的染色体中，也是乙肝病毒慢性感染的重要原因之一。 乙肝病毒的结构与其他病毒相比较简单，但其外壳层的表面抗原变异性较大，因此导致不同地区和不同人群的乙肝病毒株有很大的差异，这也是乙肝疫苗研发和预防乙肝病毒传播的一大难题。 | 乙肝病毒是一种双链DNA病毒，其结构由外壳层和核心层两部分组成。 1. 外壳层：外壳层主要由乙肝表面抗原（HBsAg）组成，其形状为球形或椭圆形，直径约为22纳米。外壳层的主要作用是保护病毒、促进病毒入侵宿主细胞，同时也是乙肝疫苗的主要成分。 2. 核心层：核心层主要由乙肝核心抗原（HBcAg）和乙肝e抗原（HBeAg）组成，形状为圆形或半圆形，直径约为30纳米。核心层包含了乙肝病毒的DNA，是病毒的遗传物质，也是乙肝病毒复制和繁殖的关键。 乙肝病毒的外壳层和核心层之间还存在一层膜状物质，称为基质（matrix），其作用是将外壳层和核心层粘在一起，维持乙肝病毒的整体结构。 了解乙肝病毒的结构可以帮助人们更好地了解乙肝感染的传播和治疗机制，同时也为研究乙肝疫苗和抗病毒药物提供了重要的基础知识。 | No | No |  | No |
| 23 | Short Answer Questions | Briefly introduce diagnostic criteria for hepatitis B | The diagnosis of hepatitis B is based on a combination of clinical features, laboratory tests, and imaging studies. Here are some of the diagnostic criteria used for hepatitis B: 1.Clinical features: Patients with acute hepatitis B may have symptoms such as jaundice, fatigue, nausea, vomiting, and abdominal pain. However, many patients with chronic hepatitis B may not have any symptoms. 2.Blood tests: Blood tests can be used to detect the presence of HBV antigens and antibodies. These include hepatitis B surface antigen (HBsAg), hepatitis B e antigen (HBeAg), hepatitis B core antibody (anti-HBc), and hepatitis B surface antibody (anti-HBs). 3.Liver function tests: Liver function tests can measure the levels of enzymes and proteins in the blood that are produced by the liver. Abnormal liver function tests can indicate liver damage or inflammation. 4.Imaging studies: Imaging studies such as ultrasound or CT scan can be used to evaluate the liver for signs of inflammation or scarring. 5.Liver biopsy: In some cases, a liver biopsy may be performed to assess the degree of liver damage or to determine the type of hepatitis B infection (acute or chronic). It is important to consult a healthcare provider for proper diagnosis and treatment of hepatitis B. Early diagnosis and treatment can help prevent serious liver damage and complications. | The diagnostic criteria for hepatitis B (HBV) infection may vary depending on the stage of the infection and the testing method used. However, some common diagnostic criteria for HBV include: Detection of HBsAg: The hepatitis B surface antigen (HBsAg) is the first and most reliable marker of HBV infection. It can be detected in the blood within a few weeks to months of infection and is used to diagnose acute and chronic HBV infection. Detection of antibodies: Antibodies against HBV can be detected in the blood during or after an acute infection. The presence of these antibodies indicates that the person has been infected with HBV and has developed an immune response to the virus. Detection of viral DNA: The amount of viral DNA in the blood can be measured using a test called a viral load assay. This test is used to monitor the progression of the infection and the response to treatment. Liver function tests: Liver function tests can be used to assess liver damage caused by HBV infection. These tests measure levels of enzymes and other substances in the blood that indicate liver damage. Imaging studies: Imaging studies, such as ultrasound, computed tomography (CT), or magnetic resonance imaging (MRI), may be used to assess liver damage and the progression of the disease. Liver biopsy: In some cases, a liver biopsy may be necessary to confirm the diagnosis of HBV and assess the extent of liver damage. A small sample of liver tissue is removed and examined under a microscope to look for signs of inflammation, fibrosis, or cirrhosis. It's important to speak with a healthcare provider if you think you may be at risk of HBV infection or if you experience any symptoms of liver disease. Early diagnosis and treatment can help prevent liver damage and the progression of the infection to chronic hepatitis B. | Yes | Yes |  | Yes |
| 23 | Short Answer Questions | 简述乙型肝炎的诊断标准 | 乙型肝炎的诊断标准主要根据以下几个方面： 1. 临床症状：乙肝病毒感染后，患者可能会出现乏力、疲劳、黄疸、恶心、呕吐、肝区疼痛等症状，这些症状有助于临床医生初步判断是否存在乙肝感染。 2. 乙肝病毒标志物检测：乙肝病毒的检测是诊断乙肝病的重要手段，包括HBsAg、HBeAg、HBcAb、HBsAb等标志物的检测。其中HBsAg阳性、HBcAb阳性、HBsAb阴性为急性乙肝感染的标志；HBeAg阳性、HBV DNA高水平为慢性乙肝感染和高度传染期的标志。 3. 肝功能检测：肝功能检测可以反映肝脏损伤和炎症程度，包括血清丙氨酸氨基转移酶（ALT）、天门冬氨酸转移酶（AST）、总胆红素、碱性磷酸酶（ALP）等指标。 4. 影像学检查：如肝脏B超、CT、MRI等检查可以评估肝脏的大小、形态、结构和功能，判断肝脏是否存在肝硬化等病变。 根据上述诊断标准，可初步诊断急性或慢性乙肝感染。对于疑似乙肝病患者，应及时进行乙肝病毒的检测和诊断，并制定个性化的治疗方案。此外，定期检测和监测乙肝病毒标志物、肝功能等指标，也是乙肝病患者管理的重要手段。 | 乙型肝炎的诊断主要基于患者的临床症状、肝功能检查、病毒学指标等多个方面进行综合分析。具体诊断标准如下： 1. 临床表现：发热、乏力、食欲不振、肝区疼痛、恶心、呕吐、黄疸等症状。 2. 体征：肝大、肝区压痛、脾大等体征。 3. 实验室检查： • 乙肝病毒表面抗原（HBsAg）：阳性表明患者有乙肝病毒感染； • 抗乙肝病毒表面抗原（anti-HBs）：阴性表明患者未曾接种乙肝疫苗或曾接种但未产生免疫力； • 抗乙肝病毒核心抗体（anti-HBc）：阳性表明患者曾经感染过乙肝病毒； • 乙肝病毒e抗原（HBeAg）：阳性表明病毒繁殖活跃，是慢性感染的危险因素； • 抗乙肝病毒e抗体（anti-HBe）：阴性表明病毒繁殖活跃； • 血清乙肝病毒DNA：可以检测病毒的数量，判断病情严重程度。 4. 肝穿刺活检：对于疑似乙肝患者进行肝穿刺活检，有助于明确肝炎的类型、病情的程度以及是否存在肝损伤等情况。 综合以上检查结果，结合患者的临床表现和病史，可以明确诊断乙型肝炎，进而制定合适的治疗方案和预后评估。 | No | No |  | No |
| 24 | Short Answer Questions | Briefly introduce hepatitis B virus family | Hepatitis B virus (HBV) belongs to the family Hepadnaviridae, which is a small family of enveloped, partially double-stranded DNA viruses. The family Hepadnaviridae includes two genera, Orthohepadnavirus and Avihepadnavirus. Orthohepadnavirus includes viruses that infect mammals, including humans, and is further divided into eight species. These include: 1.Hepatitis B virus (HBV) 2.Woolly monkey hepatitis B virus (WMHBV) 3.Ground squirrel hepatitis B virus (GSHBV) 4.Arctic squirrel hepatitis B virus (ASHBV) 5.Woodchuck hepatitis virus (WHV) 6.Eastern woodchuck hepatitis B virus (EWHBV) 7.Groundhog hepatitis virus (GHHV) 8.Bat hepatitis B virus (BtHBV) Avihepadnavirus includes viruses that infect birds and is divided into two species, duck hepatitis B virus (DHBV) and heron hepatitis B virus (HHBV). | HBV is the most well-known virus in the Hepadnaviridae family and is a major cause of acute and chronic hepatitis in humans. Understanding the family Hepadnaviridae and the properties of its viruses is important for the development of effective treatments and vaccines for hepatitis B and other related diseases. The hepatitis B virus (HBV) belongs to the Hepadnaviridae family of viruses, which are small DNA viruses that primarily infect the liver. The family Hepadnaviridae includes two genera: Orthohepadnavirus, which infects mammals, and Avihepadnavirus, which infects birds. HBV is a partially double-stranded DNA virus that replicates via an RNA intermediate. It has a circular genome of about 3.2 kilobases and encodes four major proteins: the surface antigen (HBsAg), the core antigen (HBcAg), the polymerase, and the X protein. The HBsAg is the major surface protein of the virus and is used as a target for HBV testing and vaccination. HBV is highly infectious and can be transmitted through contact with infected blood or other body fluids, such as semen and vaginal secretions. Chronic hepatitis B is a significant health problem worldwide and can lead to liver damage, liver failure, and liver cancer. There are several antiviral medications available that can help suppress the hepatitis B virus and prevent liver damage. Vaccination is the best way to prevent hepatitis B infection, and it is recommended for all infants and children, as well as for adults who are at high risk of infection. | No | No |  | No |
| 24 | Short Answer Questions | 简述乙肝病毒家族 | 乙肝病毒属于病毒界中的双链DNA病毒，乙肝病毒家族包括5个不同的种： 1. 乙型肝炎病毒（HBV）：是乙肝病毒家族中最为常见的种类，也是导致人类乙肝感染的主要病原体。 2. 旧世界鸽肝炎病毒（OWPHEV）：属于禽肝炎病毒，主要感染鸽类动物，对人类无致病性。 3. 新世界鸽肝炎病毒（NWPHEV）：也属于禽肝炎病毒，主要感染美洲洲鸽，同样对人类无致病性。 4. 圆盘鱼肝炎病毒（DHBV）：主要感染某些种类的水生动物，如鲤鱼、鲫鱼等。 5. 非洲爪蟾肝炎病毒（GHBV）：属于两栖动物肝炎病毒，主要感染非洲爪蟾。 总的来说，乙肝病毒家族的其他成员对人类的健康影响较小，而乙型肝炎病毒是其中最为重要的一种，对全球范围内的人类健康和经济社会发展都产生了严重的影响。 | 乙肝病毒属于病毒领域中的一种DNA病毒，属于瘤病毒科（Hepadnaviridae）家族。该家族的病毒一般都具有以下特征： 1. 非包膜病毒：Hepadnaviridae家族病毒没有外包膜，它们的结构比较简单，由内核和外壳两部分组成。 2. 双链DNA病毒：Hepadnaviridae家族病毒的基因组为双链DNA，相比之下，其他DNA病毒如疱疹病毒只有单链DNA。 3. 反转录酶：Hepadnaviridae家族病毒的基因组具有反转录酶活性，可以将其RNA转录成DNA，使得病毒基因组在细胞内复制。 除了乙肝病毒外，Hepadnaviridae家族还包括其他一些病毒，例如鸭病毒B（DHBV）和鹅病毒B（GHBV）。这些病毒通常只会感染特定的宿主，例如DHBV主要感染鸭子，GHBV主要感染鹅。不同种类的Hepadnaviridae家族病毒在结构和生命周期上都具有相似之处，但它们在病原性和传染性上可能存在差异，需要进一步研究和探索。 | No | No |  | No |
| 25 | Short Answer Questions | Briefly introduce HBV infection mechanism | Hepatitis B virus (HBV) infection occurs when the virus enters the bloodstream and infects liver cells (hepatocytes). The infection mechanism of HBV can be divided into several stages: 1.Attachment: HBV attaches to specific receptors on the surface of liver cells using its surface proteins, called hepatitis B surface antigens (HBsAg). 2.Entry: Once attached, HBV enters the liver cell and releases its genetic material, which consists of partially double-stranded DNA. 3.Reverse transcription: The HBV DNA is then converted into a single-stranded RNA molecule by the enzyme reverse transcriptase. 4.DNA synthesis: The viral RNA is then used as a template to synthesize new viral DNA, using the viral DNA polymerase. 5.Assembly: The new viral DNA is packaged into the viral core, which is composed of protein subunits called core antigens (HBcAg). 6.Enveloping: The new viral particles are enveloped with the lipid bilayer and surface proteins to form complete infectious virions. 7.Release: The new virions are released from the infected liver cell and can infect other liver cells or be transmitted to other individuals. HBV infection can lead to acute or chronic hepatitis, liver cirrhosis, and liver cancer. The infection mechanism of HBV is important in understanding how the virus causes disease and in the development of treatments and vaccines to prevent or control the infection. | The hepatitis B virus (HBV) infects liver cells called hepatocytes by a multi-step mechanism that involves several interactions between the virus and host cells. Here is a general overview of the HBV infection mechanism: Attachment: The virus attaches to specific receptors on the surface of the host liver cells. The major receptor for HBV is the sodium taurocholate co-transporting polypeptide (NTCP) receptor. Entry: Once the virus attaches to the host cell, it enters the cell through a process called endocytosis. During endocytosis, the cell membrane engulfs the virus and forms a vesicle called an endosome. Uncoating: Inside the endosome, the virus undergoes a conformational change that triggers the release of its genetic material, a partially double-stranded DNA molecule. Transport to the nucleus: The viral DNA is transported to the host cell nucleus, where it is converted into a covalently closed circular DNA (cccDNA) molecule. The cccDNA molecule serves as the template for the synthesis of new viral particles. Replication: The cccDNA molecule is transcribed into messenger RNA (mRNA), which is translated into viral proteins by the host cell machinery. The viral proteins include the capsid protein, envelope protein, and polymerase. Assembly: The viral proteins and viral DNA are assembled into new virions, which are released from the host cell through budding. Spread: The released virions can infect new host cells, perpetuating the infection. The HBV infection mechanism is complex and involves multiple interactions between the virus and host cells. The virus uses various strategies to evade the host immune system, leading to chronic infection in some individuals. Understanding the HBV infection mechanism is important for the development of new treatments and vaccines. | Yes | Yes |  | Yes |
| 25 | Short Answer Questions | 简述乙肝感染机制 | 乙肝病毒感染的机制涉及多个步骤： 1. 乙肝病毒入侵：乙肝病毒可以通过血液、性接触、母婴传播等途径进入人体，然后进入肝脏细胞。 2. 病毒释放：乙肝病毒释放到细胞质中，病毒DNA在细胞核内通过一系列复制和转录过程产生新的病毒颗粒，这些颗粒会在细胞内部聚集形成病毒核心颗粒。 3. 病毒膜形成：在细胞质中，新的病毒颗粒通过自组装形成病毒膜，即外壳层，包裹着内核层和病毒DNA。 4. 病毒复制：乙肝病毒的DNA可以被嵌入到宿主细胞的染色体中，形成稳定的cccDNA形式，这个过程是乙肝病毒慢性感染的关键环节。病毒的复制需要一系列的酶和蛋白质的参与，包括反转录酶、DNA聚合酶、HBsAg、HBeAg等。 5. 免疫反应：乙肝病毒感染后，机体的免疫系统会产生抗病毒抗体，包括HBsAb、HBeAb等。免疫反应的过程中，肝脏细胞受到损伤，可能出现炎症和纤维化反应，导致肝脏疾病的发展。 总的来说，乙肝病毒感染机制比较复杂，涉及多个步骤和环节。理解乙肝病毒的感染机制对于乙肝的治疗和预防具有重要意义。 | 乙肝病毒感染机制主要包括以下几个步骤： 1. 病毒进入宿主细胞：乙肝病毒首先依靠其表面的HBsAg蛋白结构，与宿主细胞的受体结合，进入宿主细胞内部。 2. 病毒基因组释放：进入宿主细胞后，乙肝病毒的基因组被释放，随后由宿主细胞的核酸合成机制产生新的病毒颗粒。 3. 病毒复制：乙肝病毒的基因组在宿主细胞内进行复制，产生新的病毒颗粒。 4. 病毒蛋白质合成：乙肝病毒的其他蛋白质也在宿主细胞中合成，包括HBcAg、HBeAg等。 5. 病毒颗粒释放：新的病毒颗粒在宿主细胞内积累，最终被释放出来，感染其他宿主细胞，或被传播至其他人体内。 值得注意的是，乙肝病毒感染并不一定会导致患者出现明显的症状，有些患者可能会成为乙肝病毒携带者，持续很长时间，同时也会成为传染源，继续传播乙肝病毒。因此，加强对乙肝病毒的认识和预防非常重要，包括接种乙肝疫苗、避免共用注射器和刺青用具、使用安全套等。 | Yes | Yes |  | Yes |
| 26 | Short Answer Questions | Briefly introduce infectivity of hepatitis B | Hepatitis B virus (HBV) is highly infectious, and can be transmitted from person to person through contact with infected blood or other bodily fluids. HBV is much more infectious than HIV, with a much lower threshold for transmission. The infectivity of HBV depends on several factors, including the amount of virus present in the blood or bodily fluids, the duration of exposure, and the mode of transmission. HBV is most infectious during the acute phase of infection when the viral load is high and symptoms are present. However, people with chronic hepatitis B infection can also transmit the virus, even if they do not have symptoms. The risk of transmission is highest through unprotected sexual contact with an infected person, sharing of needles or other injection equipment, and from an infected mother to her baby during childbirth or breastfeeding. The risk of transmission is also increased in healthcare workers who are exposed to blood or other bodily fluids in their work. Preventive measures such as vaccination, practicing safe sex, avoiding sharing of needles or other injection equipment, and using precautions to avoid exposure to infected blood and bodily fluids can help reduce the risk of HBV transmission. It is important to get tested if you think you may have been exposed to HBV, as early diagnosis and treatment can help prevent serious liver damage and complications. | Hepatitis B virus (HBV) is highly infectious and can be transmitted through contact with infected blood or other body fluids. HBV is considered to be more infectious than HIV and is 50-100 times more infectious than hepatitis C virus (HCV). Here are some factors that affect the infectivity of HBV: 1.Viral load: The amount of virus in the blood is a key factor in the infectivity of HBV. People with high viral loads are more infectious than those with low viral loads. 2.Route of transmission: HBV can be transmitted through contact with infected blood or other body fluids, such as semen and vaginal secretions. The route of transmission can affect the likelihood of infection. For example, the risk of transmission through sexual contact is higher than through casual contact. 3.Age at infection: The age at which a person is infected with HBV can affect the likelihood of chronic infection. Infants and young children are at higher risk of chronic infection than adults. 4.Immune status: People with weakened immune systems, such as those with HIV/AIDS or who are taking immunosuppressive medications, are at higher risk of HBV infection and may have more severe disease. 5.Vaccination status: Vaccination is the most effective way to prevent HBV infection. People who are not vaccinated are at higher risk of infection. It's important to take precautions to prevent HBV transmission, such as practicing safe sex, not sharing needles or other drug injection equipment, and getting vaccinated against HBV. Vaccination is the best way to prevent HBV infection. If you think you may have been exposed to HBV, it's important to get tested and speak with a healthcare provider about appropriate treatment and management options. | No | No |  | No |
| 26 | Short Answer Questions | 简述乙肝的传染性 | 乙肝是一种高传染性的疾病，其传染性来源于乙肝病毒的存在和病毒量的高低。乙肝病毒的传染性比较强，主要通过以下途径进行传播： 1. 血液传播：包括输血、手术、注射、吸毒等途径，是乙肝病毒传播最主要的途径之一。 2. 母婴传播：母体HBsAg阳性时，胎儿在出生时易受到感染。 3. 性传播：性接触时，如果乙肝病毒存在于血液或其他体液中，就会传播给对方。 4. 皮肤和黏膜接触：破损皮肤或黏膜接触乙肝患者的血液或其他体液，也可能造成乙肝病毒的传播。 除此之外，乙肝病毒的传染性还可能受到宿主个体的年龄、性别、免疫状况、感染时间、感染方式等因素的影响。相比较其他病毒，乙肝病毒的传染性比较高，但可以通过疫苗接种、个人防护措施、早期诊断和治疗等手段来减少乙肝的传播。 | 乙肝病毒具有很强的传染性，主要途径有以下几种： 1. 血液途径：感染者的血液和血制品是乙肝病毒传播的主要途径。比如通过共用注射器、输血、手术、牙医操作、血液透析等方式，都可能导致病毒的传播。 2. 性接触途径：乙肝病毒可以通过性接触传播。包括不使用安全套进行性行为、多性伴侣、与HBsAg阳性的人发生性关系等都可能导致病毒传播。 3. 垂直传播途径：感染乙肝病毒的孕妇可以将病毒传染给胎儿，或在分娩时将病毒传染给新生儿。 4. 日常接触途径：乙肝病毒在日常接触中也有一定的传播风险，包括共用个人卫生用品（如牙刷、剃须刀）、使用未经消毒的注射器和刺青用具、共用耳钉和耳环等。 需要注意的是，乙肝病毒的传播途径有很多种，而且乙肝病毒携带者有时不会出现任何症状，因此加强对乙肝病毒的防控和预防非常重要，包括接种乙肝疫苗、避免共用注射器和刺青用具、使用安全套等。如果存在可能感染乙肝病毒的情况，应及时进行乙肝病毒筛查和相关检查，以便早期发现和治疗。 | Yes | Yes |  | Yes |
| 27 | Short Answer Questions | NA |  |  |  |  |  |  |
| 27 | Short Answer Questions | 简述乙肝大三阳和小三阳的区别 | 乙肝大三阳和小三阳是乙肝病毒检测中常用的两个术语，它们之间的区别主要表现在以下方面： 1. 包含的病毒标志物不同：乙肝大三阳是指HBsAg、HBcAb和HBeAg三个标志物同时呈阳性，而乙肝小三阳是指HBsAg、HBcAb和HBsAb三个标志物中，HBsAg和HBcAb呈阳性，而HBsAb呈阴性。 2. 感染程度不同：乙肝大三阳通常是急性感染的标志，同时也是高度传染期，病毒载量高；而乙肝小三阳一般是慢性感染的表现，病毒载量相对较低，不易传染。 3. 预后和治疗方案不同：乙肝大三阳的预后不一定较乙肝小三阳差，但需要密切关注和积极治疗；而乙肝小三阳一般需要长期随访和监测，有些患者可能需要长期的抗病毒治疗，以减缓病情的进展。 总的来说，乙肝大三阳和小三阳反映了乙肝病毒感染和肝病发展的不同阶段和程度，对于患者的诊断和治疗具有重要意义。乙肝感染者应及时进行病毒标志物的检测和诊断，选择适当的治疗方案，以避免疾病的进展和传染。 | 乙肝大三阳和小三阳是乙肝病毒表面抗原（HBsAg）、乙肝病毒e抗原（HBeAg）和乙肝病毒核心抗体（anti-HBc）的不同组合状态。 1. 乙肝大三阳：指血液中同时检测到HBsAg、HBeAg和anti-HBc三种指标阳性。这种状态通常表示正在患有急性乙肝或慢性乙肝活动期，病毒载量较高，具有较强的传染性。 2. 乙肝小三阳：指血液中检测到HBsAg和anti-HBc阳性，HBeAg阴性的状态。这种状态通常表示已经感染了乙肝病毒，但病情处于隐性感染期或慢性乙肝不活动期，病毒载量较低，传染性较弱。 需要注意的是，虽然乙肝小三阳的传染性较低，但仍然存在感染他人的风险，因此乙肝小三阳的患者仍需要注意保护他人，如使用安全套、避免共用个人卫生用品等。对于患有乙肝大三阳或小三阳的患者，应及时到正规医院进行治疗和管理，以避免病情恶化，减少病毒传播。 | No | Yes | No | No |
| 28 | Short Answer Questions | Briefly introduce main indicators of HBV infection | There are several indicators of hepatitis B virus (HBV) infection that healthcare providers may use to diagnose and monitor the infection. Here are the main indicators of HBV infection: 1.Hepatitis B surface antigen (HBsAg): HBsAg is a protein on the surface of the HBV virus that can be detected in the blood of infected individuals. The presence of HBsAg indicates an active HBV infection. 2.Hepatitis B core antibody (anti-HBc): Anti-HBc is an antibody that is produced in response to the core antigen of the HBV virus. The presence of anti-HBc indicates that the individual has been infected with HBV at some point, but it does not distinguish between acute and chronic infection. 3.Hepatitis B e antigen (HBeAg): HBeAg is a protein produced by the HBV virus during active viral replication. The presence of HBeAg indicates a high level of viral replication and a higher risk of transmission. 4.Hepatitis B surface antibody (anti-HBs): Anti-HBs is an antibody produced by the immune system in response to HBV vaccination or previous infection. The presence of anti-HBs indicates immunity to HBV. 5.Liver function tests: Liver function tests measure the levels of enzymes and proteins in the blood that are produced by the liver. Abnormal liver function tests can indicate liver damage or inflammation, which may be caused by HBV infection. These indicators are used to diagnose and monitor HBV infection, determine the stage of the infection (acute or chronic), assess the level of viral replication, and guide treatment decisions. It is important to consult a healthcare provider for proper diagnosis and treatment of HBV infection. | There are several indicators of hepatitis B virus (HBV) infection that healthcare providers use to diagnose and manage the disease. These indicators include: 1.HBsAg: The hepatitis B surface antigen (HBsAg) is the first and most reliable indicator of HBV infection. It is a protein on the surface of the virus that can be detected in the blood within a few weeks to months of infection. The presence of HBsAg indicates that a person is infected with HBV. 2.Anti-HBc: The hepatitis B core antibody (anti-HBc) is a marker of previous or ongoing HBV infection. It is a protein produced by the immune system in response to the core antigen of the virus. Anti-HBc can be detected in the blood during acute or chronic HBV infection and can remain in the blood for years after infection. 3.HBV DNA: Hepatitis B viral DNA (HBV DNA) is a measure of the amount of virus in the blood. It is used to monitor the progression of the infection and the response to treatment. HBV DNA levels can be high during active infection and low or undetectable during inactive or resolved infection. 4.Liver function tests: Hepatitis B infection can cause inflammation and damage to the liver, which can be detected by liver function tests. These tests measure levels of enzymes and other substances in the blood that indicate liver damage. It's important to get tested for hepatitis B if you think you may have been exposed to the virus or if you experience any symptoms of liver disease, such as fatigue, abdominal pain, or yellowing of the skin and eyes (jaundice). Early diagnosis and treatment can help prevent liver damage and the progression of the infection to chronic hepatitis B. | No | No |  | No |
| 28 | Short Answer Questions | 简述乙肝感染的主要指标 | 乙肝感染的主要指标包括： 1. HBsAg：乙肝表面抗原，是乙肝病毒感染最重要的标志物，其检测结果通常是判断是否存在乙肝病毒感染的主要依据。 2. HBcAb：乙肝核心抗体，可以表明是否曾经感染过乙肝病毒。 3. HBeAg：乙肝e抗原，是乙肝病毒活动的指标，它的阳性通常意味着乙肝病毒感染者的病毒载量高，是乙肝传染性最强的阶段。 4. HBV DNA：乙肝病毒DNA，可以反映乙肝病毒的活动和复制情况，也是判断乙肝病毒感染的重要标志物之一。 5. ALT、AST等肝功能指标：肝功能指标的升高通常可以反映肝细胞的受损程度，也是判断乙肝病毒感染及肝炎活动性的重要依据之一。 此外，针对不同的乙肝感染阶段，还需要进行其他的相关检测，例如乙肝前兆期需要进行IgM抗体的检测，慢性乙肝需要进行肝功能、肝硬化、肝癌等方面的检查。在进行乙肝病毒检测时，应选择合适的指标组合，并结合临床症状和其他检查结果进行综合分析，以更准确地判断患者是否感染了乙肝病毒。 | 乙肝感染的主要指标包括以下几个方面： 1. HBsAg：乙肝病毒表面抗原，是乙肝感染最常用的检测指标，阳性表示感染者已经携带了乙肝病毒。 2. HBeAg：乙肝病毒e抗原，是乙肝病毒复制活跃的标志，通常与HBV DNA水平高度相关，阳性表示感染者病情较重。 3. anti-HBc：乙肝病毒核心抗体，是乙肝病毒感染后早期产生的抗体，阳性表示感染者曾经接触过乙肝病毒。 4. HBV DNA：乙肝病毒DNA，是评估乙肝感染病情的重要指标，也是评估治疗效果的重要指标。 5. ALT：谷丙转氨酶，是肝脏细胞损伤的标志，通常可以反映肝脏炎症程度和乙肝感染的活跃程度。 以上指标可以帮助医生判断乙肝感染者的感染状态和病情严重程度，同时也可以用来评估治疗效果。建议有乙肝感染风险的人定期检查乙肝相关指标，及时发现和处理乙肝感染，预防病情恶化和传染给他人。 | No | No |  | No |
| 29 | Short Answer Questions | Briefly introduce the difference between hepatitis A, hepatitis B, and hepatitis C? | Hepatitis A, hepatitis B, and hepatitis C are three different viruses that can cause inflammation of the liver, known as hepatitis. While all three viruses affect the liver, there are significant differences between them in terms of transmission, symptoms, and treatment. Here are the main differences: 1.Transmission: Hepatitis A is mainly spread through contaminated food or water, while hepatitis B and C are primarily transmitted through exposure to infected blood or bodily fluids, such as during unprotected sex or sharing of needles. Hepatitis B can also be transmitted from mother to child during childbirth. 2.Symptoms: The symptoms of hepatitis A, B, and C can be similar, and may include fatigue, nausea, vomiting, abdominal pain, and jaundice. However, hepatitis A usually causes a milder illness, with symptoms lasting a few weeks, while hepatitis B and C can cause chronic infection that may lead to long-term liver damage. 3.Treatment: Hepatitis A usually resolves on its own without specific treatment, while hepatitis B and C may require antiviral medications to reduce the amount of virus in the body and prevent liver damage. A vaccine is available to prevent hepatitis A and B, but there is no vaccine for hepatitis C. 4.Prognosis: The prognosis for hepatitis A is generally good, with most people recovering completely within a few weeks. Hepatitis B and C can cause chronic infection, which may lead to cirrhosis or liver cancer in some cases. 1.It is important to consult a healthcare provider for proper diagnosis and treatment of hepatitis A, B, or C. Preventive measures such as vaccination, practicing safe sex, avoiding sharing of needles or other injection equipment, and using precautions to avoid exposure to infected blood and bodily fluids can help reduce the risk of hepatitis infection. | Hepatitis A, hepatitis B, and hepatitis C are all viral infections that affect the liver. However, they are caused by different viruses and have different modes of transmission, symptoms, and treatment options. Here are the main differences between the three types of hepatitis: 1.Hepatitis A: Hepatitis A is caused by the hepatitis A virus (HAV) and is typically spread through contaminated food or water. The symptoms of hepatitis A are similar to those of the flu and can include fever, fatigue, loss of appetite, nausea, vomiting, abdominal pain, and yellowing of the skin and eyes (jaundice). However, most people with hepatitis A recover without any long-term liver damage, and there is a vaccine available to prevent infection. 2.Hepatitis B: Hepatitis B is caused by the hepatitis B virus (HBV) and is typically spread through contact with infected blood, semen, or other bodily fluids. The symptoms of acute hepatitis B are similar to those of hepatitis A, but the infection can also lead to chronic hepatitis B, which can cause liver damage, liver failure, or liver cancer. There is a vaccine available to prevent hepatitis B, and antiviral medications can help manage the infection in people with chronic hepatitis B. 3.Hepatitis C: Hepatitis C is caused by the hepatitis C virus (HCV) and is typically spread through contact with infected blood. Many people with hepatitis C do not experience any symptoms, but the infection can lead to chronic hepatitis C, which can cause liver damage, liver failure, or liver cancer. There is no vaccine for hepatitis C, but antiviral medications can cure the infection in most cases. In summary, hepatitis A, hepatitis B, and hepatitis C are all viral infections that affect the liver, but they are caused by different viruses, spread through different modes of transmission, and have different symptoms and treatment options. It's important to speak with a healthcare provider if you think you may be at risk for any type of hepatitis or if you experience any symptoms of liver disease. | Yes | Yes |  | Yes |
| 29 | Short Answer Questions | 简述甲肝，乙肝，丙肝的区别 | 甲肝、乙肝、丙肝是三种不同类型的肝炎病毒引起的肝炎，它们之间的区别如下： 1. 病毒类型不同：甲肝病毒为RNA病毒，乙肝病毒为DNA病毒，丙肝病毒也是RNA病毒。 2. 传播途径不同：甲肝病毒主要通过食物、水源等途径传播；乙肝病毒主要通过血液、性接触等途径传播；丙肝病毒主要通过血液传播。 3. 感染后症状不同：甲肝一般表现为急性肝炎，症状包括发热、乏力、黄疸等；乙肝感染者可能出现急性肝炎、慢性肝炎、肝硬化和肝癌等症状；丙肝感染者多数为慢性感染，部分患者可能进展为肝硬化和肝癌。 4. 预防措施不同：甲肝可以通过卫生措施和甲肝疫苗预防；乙肝可以通过乙肝疫苗接种和避免血液、性接触等途径的传播预防；丙肝目前尚无有效的疫苗，可以通过避免血液传播和使用无菌注射器等方式预防。 需要注意的是，三种肝炎病毒都可以引起肝脏病变，对人体健康造成严重危害。因此，在预防和治疗肝炎方面，不同类型的肝炎病毒都应该受到足够的重视和关注。 | 甲肝、乙肝和丙肝是三种不同的肝炎疾病，由不同的病毒引起，具有不同的传播途径、临床表现和治疗方法。 1. 甲肝（HAV）：甲肝病毒是一种单股RNA病毒，主要通过食物或饮用水中含有被感染人排泄的病毒传播。通常属于急性感染，患者通常不会变为慢性感染者。其临床症状包括发热、恶心、呕吐、腹泻、黄疸等，多数患者症状会在数周内缓解，预后通常良好。甲肝疫苗和改善个人卫生习惯是预防甲肝感染的重要手段。 2. 乙肝（HBV）：乙肝病毒是一种双股DNA病毒，主要通过血液和体液传播途径感染人体。乙肝可分为急性和慢性两种类型，约有5%至10%的患者可能成为慢性感染者。其临床症状包括发热、乏力、食欲不振、黄疸、肝肿大等，可引起肝硬化、肝癌等严重后果。乙肝疫苗和抗病毒治疗是预防和治疗乙肝的主要手段。 3. 丙肝（HCV）：丙肝病毒是一种单股RNA病毒，主要通过血液传播途径感染人体。丙肝感染后，约70%至85%的患者可能成为慢性感染者。其临床症状与乙肝类似，包括乏力、肝肿大、黄疸等，也可能引起肝硬化、肝癌等后果。目前，丙肝尚无有效的疫苗，但抗病毒治疗可以显著提高治愈率和缓解病情。 需要指出的是，由于甲肝、乙肝和丙肝的病原体、传播途径和治疗方法等存在差异，因此预防和治疗这些肝病需要有针对性和个性化。 | No | Yes | No | No |
| 30 | Short Answer Questions | Who is most likely to get chronic (long-term) hepatitis B? | Chronic (long-term) hepatitis B is a condition in which the hepatitis B virus (HBV) remains in the body for more than six months, leading to ongoing liver inflammation and potential liver damage. While anyone can get chronic hepatitis B, some people are at higher risk than others. Here are some of the groups that are more likely to develop chronic hepatitis B: 1.Infants and children: Infants and children who are infected with HBV are more likely to develop chronic hepatitis B than adults. In fact, up to 90% of infants infected with HBV will develop chronic infection. 2.People with weakened immune systems: People with weakened immune systems, such as those with HIV or undergoing immunosuppressive therapy, are more likely to develop chronic hepatitis B. 3.People with certain genetic factors: Some genetic factors may increase the risk of chronic hepatitis B, such as having certain variations in the human leukocyte antigen (HLA) genes. 4.People who were infected at a young age: People who were infected with HBV at a young age, especially before the age of 5, are more likely to develop chronic hepatitis B. 5.People who are male: Men are slightly more likely than women to develop chronic hepatitis B. 1.It is important to get tested for hepatitis B if you are at risk, as early diagnosis and treatment can help prevent serious liver damage and complications. Preventive measures such as vaccination, practicing safe sex, avoiding sharing of needles or other injection equipment, and using precautions to avoid exposure to infected blood and bodily fluids can help reduce the risk of HBV infection and chronic hepatitis B. | Chronic hepatitis B is a liver disease caused by the hepatitis B virus (HBV) that lasts longer than six months. While anyone can contract HBV, some individuals are more likely than others to develop chronic hepatitis B. The people who are at the highest risk of chronic hepatitis B infection are: 1.Infants born to mothers who are infected with HBV: Babies can be infected with HBV during delivery or shortly after birth, and if they don't receive appropriate treatment, they have a higher risk of developing chronic hepatitis B. 2.People who were infected with HBV at a young age: Children who contract HBV before the age of five have a much higher risk of developing chronic hepatitis B than adults who are infected with the virus. 3.People with weakened immune systems: Those with weakened immune systems due to medical conditions, such as HIV/AIDS, or medications that suppress the immune system, such as chemotherapy, are more likely to develop chronic hepatitis B. 4.Those who have close household or sexual contact with someone who has chronic hepatitis B: HBV is highly infectious and can spread through contact with an infected person's blood, semen, or other body fluids. People who live with or have sexual contact with someone who has chronic hepatitis B are at higher risk of contracting the virus and developing chronic hepatitis B. 5.People from regions with a high prevalence of hepatitis B: Chronic hepatitis B is more common in regions with high rates of HBV, including parts of Asia, Africa, and the Pacific Islands. If you believe you may be at risk of chronic hepatitis B, it's essential to get tested and speak with a healthcare provider about appropriate treatment and management options. | No | No |  | No |
| 30 | Short Answer Questions | 简述慢性乙肝的易感人群 | 乙肝的易感人群主要包括以下几类人群： 1. 新生儿和儿童：因为乙肝病毒的传播方式包括母婴传播、密切接触等途径，所以新生儿和儿童是乙肝的高危人群。同时，儿童对乙肝疫苗的免疫效果较好，所以推广疫苗接种是预防乙肝的重要措施。 2. 医务人员和实验室工作者：因为医务人员和实验室工作者可能接触到患者的血液和其他体液，所以他们是乙肝感染的高危人群。 3. 性接触者：乙肝病毒可以通过性接触途径传播，所以性接触者也是乙肝的易感人群之一。 4. 吸毒者和注射药物者：因为注射药物和吸毒可能使用共用注射器和吸毒器具，这些器具可能被污染，从而导致乙肝病毒的传播。 5. 慢性肝病患者：慢性肝病患者由于肝脏病变，其肝功能可能受到影响，从而导致对乙肝病毒的免疫力下降，易于感染乙肝病毒。 除了以上人群，其他人群如多次输血者、肝癌患者、透析患者等也是乙肝的易感人群之一。针对这些人群，应当采取积极的预防和控制措施，例如接种疫苗、避免不必要的手术和输血等操作，同时定期进行乙肝病毒的检测和监测。 | 乙肝病毒的易感人群包括以下几类： 1. 新生儿和婴幼儿：从乙肝病毒感染的母亲身上传染给胎儿或婴儿，尤其是在母亲乙肝病毒表面抗原（HBsAg）阳性的情况下易感。 2. 家庭成员和性伴侣：乙肝病毒通过血液、唾液、精液等途径传播，密切接触的家庭成员和性伴侣易感染乙肝病毒。 3. 医务工作者：乙肝病毒感染的患者可能通过血液、呼吸道分泌物等途径传染给医务工作者，因此医务工作者易感染乙肝病毒。 4. 注射毒品人群：共用注射器和针头是乙肝病毒传播的重要途径，注射毒品人群易感染乙肝病毒。 5. 血液透析患者：血液透析治疗需要经常进行血液置换，如果血液或器具受到乙肝病毒污染，透析患者易感染乙肝病毒。 6. 移民或旅居海外人士：一些地区的乙肝病毒感染率很高，而且一些国家或地区的乙肝病毒感染情况没有得到有效控制，因此移民或旅居海外人士易感染乙肝病毒。 7. 其他易感人群：如需要经常输血或血制品治疗的患者、器官移植受体、普通人群中存在免疫缺陷等人群也易感染乙肝病毒。 | Yes | No | No | No |
| 31 | Short Answer Questions | Briefly introduce population at risk for hepatitis B | Certain populations are at higher risk for hepatitis B virus (HBV) infection than others. Here are some groups that are at increased risk for HBV infection: 1.Infants born to infected mothers: Infants born to mothers who are infected with HBV are at high risk of developing chronic hepatitis B. 2.Sexual partners of infected individuals: People who have unprotected sex with someone who is infected with HBV are at risk of contracting the virus. 3.People who inject drugs: People who inject drugs are at higher risk of HBV infection if they share needles or other drug equipment with someone who is infected. 4.Healthcare workers: Healthcare workers who are exposed to infected blood or bodily fluids are at increased risk of HBV infection. 5.People with multiple sexual partners: People who have multiple sexual partners, especially if they engage in unprotected sex, are at increased risk of HBV infection. 6.Men who have sex with men: Men who have sex with men are at increased risk of HBV infection, particularly if they have multiple sexual partners. 7.People who travel to areas with high rates of HBV infection: People who travel to areas with high rates of HBV infection, such as parts of Asia, Africa, and the Pacific Islands, are at increased risk of HBV infection. It is important to get tested for HBV if you are at risk, as early diagnosis and treatment can help prevent serious liver damage and complications. Preventive measures such as vaccination, practicing safe sex, avoiding sharing of needles or other injection equipment, and using precautions to avoid exposure to infected blood and bodily fluids can help reduce the risk of HBV infection. | Any person can contract hepatitis B, but certain populations are at a higher risk of getting infected. The following groups of people are at a greater risk of contracting hepatitis B: 1.Infants and children born to mothers with hepatitis B: Infants can contract hepatitis B during delivery or shortly after birth if the mother is infected with the virus. Without treatment, these infants have a high risk of developing chronic hepatitis B. 2.People who have unprotected sex: Hepatitis B is a sexually transmitted infection and can spread through unprotected sex with an infected person. 3.People who inject drugs: Hepatitis B can spread through the sharing of needles or other drug injection equipment. 4.Healthcare workers: Healthcare workers who come in contact with infected blood or body fluids are at an increased risk of contracting hepatitis B. 5.Men who have sex with men: Men who have sex with men are at a higher risk of contracting hepatitis B due to sexual activity that can lead to exposure to infected blood or body fluids. 6.People from regions with high prevalence rates: Hepatitis B is more common in certain regions of the world, including parts of Asia, Africa, and the Pacific Islands. People who were born in or travel to these regions are at a higher risk of contracting the virus. 7.People with weakened immune systems: People with weakened immune systems due to medical conditions, such as HIV/AIDS or medications that suppress the immune system, are at a higher risk of contracting hepatitis B. It's important to get vaccinated against hepatitis B if you are in a high-risk group or if you may be exposed to the virus. Vaccination is the best way to prevent hepatitis B infection. | No | No |  | Yes |
| 31 | Short Answer Questions | 简述乙肝的高危人群 | 乙肝的高危人群包括以下几类人群： 1. 乙肝病毒携带者：指携带HBsAg阳性的人群，虽然这些人不一定出现肝炎症状，但是却是乙肝病毒的携带者，具有传染性。 2. 慢性乙肝患者：慢性乙肝是指HBsAg阳性并且超过6个月以上的感染者，这些人常常没有症状，但其病毒持续存在于体内，可导致肝硬化和肝癌等严重疾病。 3. 与乙肝病毒有密切接触的人群：例如患有乙肝病毒的家庭成员、性伴侣等，这些人的感染风险较高。 4. 肝病患者：例如慢性肝炎、肝硬化、肝癌等患者，由于其肝脏病变、免疫力下降等原因，易受到乙肝病毒的侵袭。 5. 注射毒品和共用注射器的人群：注射毒品和共用注射器是传播乙肝病毒的主要途径之一。 6. 透析患者：透析患者需要进行频繁的血液透析治疗，这可能会导致感染乙肝病毒。 总的来说，以上人群是乙肝感染和传播的高危人群，需要加强宣传教育，提高其对乙肝感染的认识和防范意识。同时，政府和医疗机构也应加强对这些人群的管理和照顾，提高预防和治疗乙肝的能力。 | 以下是乙肝的高危人群： 1. 新生儿和婴幼儿：如果母亲是乙肝病毒携带者或感染者，则新生儿会在出生时受到乙肝病毒的感染风险，因此应尽早接种乙肝疫苗并注射乙肝免疫球蛋白。 2. 医务人员和实验室人员：由于乙肝病毒的传播途径主要是血液和体液等，所以医务人员、实验室人员等高风险职业人群容易受到乙肝病毒感染。 3. 性接触史的人群：性接触是乙肝病毒的一种常见传播途径，因此有多个性伴侣、同性恋、经常进行不安全性行为等人群容易感染乙肝病毒。 4. 具有病毒感染史的人群：如果曾经感染过乙肝病毒，但未接受过及时、有效的治疗，那么就容易成为慢性感染者，并可能对身体产生严重危害。 5. 乙肝病毒携带者的家属：乙肝病毒携带者的配偶和其他家庭成员是易感染乙肝病毒的高风险人群，应接种乙肝疫苗，并注意采取必要的防护措施。 6. 吸毒者：共用注射器等不良行为容易感染乙肝病毒，因此吸毒者也是乙肝高危人群之一。 需要注意的是，以上列出的高危人群只是常见的人群类型，而并不代表其他人群不容易感染乙肝病毒。因此，接种乙肝疫苗、保持卫生健康习惯、避免过度劳累和注意安全性行为等，对于预防乙肝感染具有重要的作用。 | No | No |  | No |
| 32 | Short Answer Questions | Briefly introduce hepatitis B prevention strategy | Hepatitis B virus (HBV) is highly infectious and can cause serious liver damage and other health complications. Fortunately, there are several strategies that can help prevent HBV infection. Here are some key prevention strategies: 1.Vaccination: The hepatitis B vaccine is highly effective at preventing HBV infection and is recommended for all infants, children, and adults who have not been previously vaccinated. 2.Safe sex: Practicing safe sex, such as using condoms, can help reduce the risk of HBV transmission during sexual activity. 3.Avoid sharing needles or other injection equipment: Using clean needles and other injection equipment, or not injecting drugs, can help reduce the risk of HBV transmission among people who inject drugs. 4.Screen blood and organ donors: Screening blood and organ donors for HBV can help prevent transmission of the virus through transfusions or organ transplants. 5.Use precautions in healthcare settings: Healthcare workers should use precautions, such as wearing gloves and other protective gear, to avoid exposure to infected blood and bodily fluids. 6.Prevent mother-to-child transmission: Infants born to mothers who are infected with HBV should receive hepatitis B immune globulin and the hepatitis B vaccine to prevent mother-to-child transmission. 7.Increase public education and awareness: Educating the public about the risks and prevention of HBV infection can help reduce the incidence of the disease. It is important to consult a healthcare provider for proper diagnosis and treatment of HBV infection. Preventive measures such as vaccination, practicing safe sex, avoiding sharing of needles or other injection equipment, and using precautions to avoid exposure to infected blood and bodily fluids can help reduce the risk of HBV infection. | Preventing hepatitis B infection involves several strategies, including: 1.Vaccination: Vaccination is the best way to prevent hepatitis B infection. The hepatitis B vaccine is safe and highly effective, and it is recommended for all infants, children, and adults who are at risk of infection. The vaccine is given in three doses over a six-month period. 2.Safe sex practices: Hepatitis B can be spread through unprotected sex with an infected person. Practicing safe sex, such as using condoms, can reduce the risk of infection. 3.Not sharing needles: Hepatitis B can be spread through sharing needles or other drug injection equipment. Avoiding drug use or using sterile needles can help prevent infection. 4.Safe medical procedures: Healthcare workers and others who come into contact with infected blood or bodily fluids should follow standard precautions, such as wearing gloves and using sterile equipment, to prevent transmission of the virus. 5.Screening of blood and organ donors: In most countries, blood and organ donors are screened for hepatitis B to prevent transmission of the virus. 6.Screening of pregnant women: Pregnant women should be screened for hepatitis B to prevent mother-to-child transmission of the virus. 7.Post-exposure prophylaxis: If you are exposed to hepatitis B, such as through an accidental needlestick, post-exposure prophylaxis (PEP) can help prevent infection. PEP involves receiving the hepatitis B vaccine and/or hepatitis B immune globulin within 24 hours of exposure. It's important to speak with a healthcare provider if you think you may be at risk of hepatitis B infection or if you experience any symptoms of liver disease. Early diagnosis and treatment can help prevent liver damage and the progression of the infection to chronic hepatitis B. | No | No |  | No |
| 32 | Short Answer Questions | 简述乙肝的预防策略 | 乙肝的预防策略主要包括以下几个方面： 1. 接种乙肝疫苗：接种乙肝疫苗是预防乙肝的最有效方法之一，可以大大降低感染乙肝病毒的风险。乙肝疫苗需要按照规定的剂量和时间接种，建议婴儿出生后尽早接种疫苗，成年人也可以通过接种疫苗来增强免疫力。 2. 避免血液和体液的接触：乙肝病毒可以通过血液和体液途径传播，因此要避免接触不洁血液、不洁性器官分泌物等，避免共用注射器和吸毒器等。 3. 保持个人卫生：个人卫生是预防乙肝的重要措施之一。要保持身体清洁，勤洗手、勤洗澡，不吃生的或未煮熟的食物，不饮用不洁水源。 4. 促进公众健康教育：加强公众健康教育，提高公众对乙肝病毒的认识和防范意识，推广乙肝疫苗接种等措施，降低乙肝感染的风险。 5. 定期检测和治疗：定期检测HBsAg和其他乙肝病毒标志物，及时发现和治疗乙肝病毒感染，以减少乙肝病毒的传播和疾病的发生。 6 加强医疗管理：对医务人员和其他高危人群进行乙肝病毒的检测和管理，提高医疗机构和医务人员对乙肝的防范和诊治能力，防止乙肝的交叉感染。 综上所述，预防乙肝需要采取多种措施，从个人、家庭到社区和国家层面都需要加强预防和控制乙肝病毒的工作，以保障人民健康和社会稳定。 | 乙肝的预防策略主要包括以下几个方面： 1. 接种乙肝疫苗：乙肝疫苗是预防乙肝病毒感染最有效的方法之一，推荐所有人群进行接种。根据接种情况，乙肝疫苗可以分为基础免疫和加强免疫，推荐的接种时间和剂量也有所不同。 2. 保持个人卫生：乙肝病毒可以通过血液、性行为、母婴传播等途径传播，因此保持个人卫生非常重要。建议使用安全套、避免共用个人卫生用品等。 3. 避免与乙肝感染者接触：乙肝感染者的血液、体液等都可能携带乙肝病毒，接触这些物质可能会感染病毒。因此，避免与乙肝感染者接触，尤其是避免共用个人卫生用品等。 4. 食品安全：乙肝病毒可以通过污染的食品、饮水等途径传播，因此保障食品安全也是预防乙肝感染的重要措施。 5. 定期体检：对于有乙肝感染风险的人群，建议定期进行乙肝相关检测，及早发现和处理乙肝感染，预防病情恶化和传染给他人。 综上所述，乙肝的预防策略需要从个人、家庭和社会多个层面入手，建议大家加强宣传和教育，共同致力于预防和控制乙肝的发生和传播。 | No | No |  | No |
| 33 | Short Answer Questions | Who should get vaccinated against hepatitis B | The hepatitis B vaccine is recommended for everyone, but there are certain groups of people who are at higher risk of hepatitis B virus (HBV) infection and should receive the vaccine. Here are some groups of people who should get vaccinated against HBV: 1.Infants, children, and adolescents: The hepatitis B vaccine is recommended for all infants, starting at birth, and for children and adolescents who were not vaccinated as infants. 2.Healthcare workers: Healthcare workers who are at risk of exposure to blood or other bodily fluids should receive the hepatitis B vaccine. 3.People with certain medical conditions: People with certain medical conditions, such as chronic liver disease, kidney disease, or HIV, should receive the hepatitis B vaccine. 4.People who inject drugs: People who inject drugs are at increased risk of HBV infection and should receive the hepatitis B vaccine. 5.People with multiple sexual partners: People who have multiple sexual partners, especially if they engage in unprotected sex, are at increased risk of HBV infection and should receive the hepatitis B vaccine. 6.Men who have sex with men: Men who have sex with men are at increased risk of HBV infection and should receive the hepatitis B vaccine. 7.Travelers to areas with high rates of HBV infection: People who travel to areas with high rates of HBV infection, such as parts of Asia, Africa, and the Pacific Islands, should receive the hepatitis B vaccine. It is important to consult a healthcare provider for proper diagnosis and treatment of HBV infection. The hepatitis B vaccine is safe and highly effective at preventing HBV infection and its complications, including liver cancer and cirrhosis. | The hepatitis B vaccine is recommended for all infants, children, and adults who are at risk of HBV infection. Here are some groups of people who should receive the hepatitis B vaccine: Infants: All infants should receive the hepatitis B vaccine as part of their routine childhood immunization schedule. The vaccine is given in a series of three doses, with the first dose given at birth, the second dose given at 1-2 months of age, and the third dose given at 6-18 months of age. Healthcare workers: Healthcare workers who may be exposed to blood or bodily fluids should be vaccinated against hepatitis B to reduce their risk of infection. People with multiple sexual partners: People who have multiple sexual partners are at increased risk of HBV infection and should receive the vaccine. People who inject drugs: People who inject drugs are at increased risk of HBV infection and should receive the vaccine. People with chronic liver disease: People with chronic liver disease, such as cirrhosis or hepatitis C, are at increased risk of complications from HBV infection and should receive the vaccine. Travelers to high-risk areas: People who are traveling to areas where HBV infection is common, such as parts of Africa, Asia, and the Pacific Islands, should receive the vaccine. Household contacts of people with HBV infection: People who live with someone who has chronic HBV infection are at increased risk of infection and should receive the vaccine. It's important to speak with a healthcare provider if you think you may be at risk of HBV infection or if you have questions about the hepatitis B vaccine. They can provide more information and guidance based on your individual situation. | No | No |  | No |
| 33 | Short Answer Questions | 简述乙肝疫苗适用人群 | 乙肝疫苗适用人群主要包括以下几类人群： 1. 新生儿和儿童：新生儿和儿童是乙肝疫苗的主要接种人群，接种乙肝疫苗可以有效预防乙肝病毒的感染和传播，保障儿童健康成长。 2. 未感染乙肝病毒的成人：未感染乙肝病毒的成人包括高危人群和一般人群，例如医务人员、实验室工作者、性接触者、注射毒品者、透析患者等。接种乙肝疫苗可以有效预防乙肝病毒的感染和传播。 3. 未完成疫苗接种的人群：未完成乙肝疫苗接种计划的人群，包括未完成基础免疫和未完成加强免疫的人群。这些人需要及时补充疫苗接种，以达到乙肝疫苗的保护效果。 4. 有乙肝病毒感染风险的人群：例如曾经输过血或者有过手术、注射药物等操作的人群，接种乙肝疫苗可以降低感染乙肝病毒的风险。 需要注意的是，有些人群存在禁忌接种的情况，例如对疫苗成分过敏、急性发热等情况。在接种乙肝疫苗前，需要咨询医生，了解自身适宜接种的情况。同时，接种乙肝疫苗也不是百分之百有效，因此在日常生活中仍需要注意预防乙肝病毒的感染和传播。 | 乙肝疫苗适用于以下人群： 1. 新生儿：中国已经将乙肝疫苗列为国家免疫规划的必选疫苗之一，所有新生儿都应接种乙肝疫苗。 2. 没有乙肝病毒感染的人群：乙肝疫苗适用于没有乙肝病毒感染的人群，以提高他们的免疫力，预防乙肝病毒感染。 3. 有乙肝病毒感染风险的人群：包括医疗工作者、注射毒品人群、性传播高危人群等有乙肝病毒感染风险的人群。 4. 未接种或接种不全的人群：未接种或接种不全的人群应该尽早接种乙肝疫苗，以提高免疫力，预防乙肝病毒感染。 需要注意的是，乙肝疫苗适用于大多数人群，但在一些特殊情况下，例如存在过敏史、急性发热或疾病时，可能需要咨询医生是否适合接种乙肝疫苗。同时，乙肝疫苗接种的具体方案和剂量应该根据不同的年龄、病史和乙肝感染风险等因素进行调整，建议在医生的指导下接种。 | Yes | Yes |  | Yes |
| 34 | Short Answer Questions | Who should not receive the hepatitis B vaccine? | The hepatitis B vaccine is generally safe and well-tolerated, and most people can receive the vaccine without any problems. However, there are some situations where the vaccine may not be recommended or should be delayed. Here are some groups of people who may not be able to receive the hepatitis B vaccine: 1.People who have had a severe allergic reaction to a previous dose of the hepatitis B vaccine or any component of the vaccine. 2.People who are currently ill or have a fever. In general, vaccination should be delayed until the person has recovered. 3.Infants who are born weighing less than 2,000 grams. These infants may receive a different schedule of the hepatitis B vaccine. 4.People who are allergic to yeast, as the hepatitis B vaccine is made using yeast. 5.Pregnant women. While the hepatitis B vaccine is generally considered safe during pregnancy, it is not routinely recommended unless the woman is at high risk of HBV infection. It is important to consult a healthcare provider before receiving the hepatitis B vaccine to discuss any concerns or medical conditions that may affect vaccine eligibility. In general, the hepatitis B vaccine is safe and highly effective at preventing HBV infection and its complications, including liver cancer and cirrhosis. | In general, the hepatitis B vaccine is safe and effective for most people, and it is recommended for all infants, children, and adults who are at risk of infection. However, there are some situations where the vaccine may not be recommended or may need to be delayed. These include: Severe allergic reaction to a previous dose of the vaccine: If a person has had a severe allergic reaction (such as anaphylaxis) to a previous dose of the hepatitis B vaccine or any component of the vaccine, they should not receive further doses. Severe allergic reaction to yeast: The hepatitis B vaccine contains yeast as a component, and people who have had a severe allergic reaction to yeast should not receive the vaccine. Acute illness with fever: If a person has an acute illness with fever, the hepatitis B vaccine may be delayed until they have recovered. Pregnancy: The hepatitis B vaccine is generally considered safe during pregnancy, but it is usually only given to pregnant women who are at high risk of infection, such as healthcare workers or women with multiple sexual partners. Infants with certain medical conditions: Infants who have certain medical conditions, such as a severe allergy to baker's yeast or a history of seizures, may need to delay or avoid the hepatitis B vaccine. It's important to speak with a healthcare provider if you have concerns about receiving the hepatitis B vaccine or if you think you may be at risk of HBV infection. They can provide more information and guidance based on your individual situation. | No | No |  | No |
| 34 | Short Answer Questions | 简述乙肝疫苗禁忌人群 | 乙肝疫苗的禁忌证主要包括以下几类： 1. 对疫苗成分过敏：乙肝疫苗中可能含有酵母蛋白等成分，对这些成分过敏的人不适宜接种乙肝疫苗。 2. 急性发热和急性感染：由于急性发热和急性感染会影响人体免疫系统，接种疫苗可能会导致不良反应，因此这些人在发热或者急性感染期间不适宜接种疫苗。 3. 免疫缺陷患者：例如感染HIV病毒、患有白血病等免疫缺陷疾病的患者，其免疫系统功能较低，接种疫苗可能会导致不良反应或者疫苗效果不佳。 4. 孕妇：由于乙肝疫苗的安全性在孕妇身上尚未经过充分的研究，孕妇不建议接种乙肝疫苗。 5. 严重的过敏史：曾经出现过严重的过敏反应（例如过敏性休克、呼吸困难等）的人群，不适宜接种乙肝疫苗。 需要注意的是，接种疫苗时需要告知医生自己是否存在上述禁忌证，避免因为疫苗接种导致不良反应和并发症的发生。同时，即使没有上述禁忌证，也可能出现轻微的不适和不良反应，这是正常的反应，不需要过度担心。如果症状严重或者持续时间较长，应及时就医。 | 乙肝疫苗是一种安全有效的预防乙肝病毒感染的疫苗，但是对于某些人群来说，接种乙肝疫苗可能会出现禁忌证，包括以下几个方面： 1. 对乙肝疫苗过敏的人：对乙肝疫苗或其任何成分过敏的人不适宜接种乙肝疫苗。 2. 发热或急性疾病的人：对于正在发热或急性疾病的人，建议延迟接种乙肝疫苗，直到身体状况稳定后再接种。 3. 免疫系统受损的人：对于免疫系统受损的人，如患有某些疾病、正在接受免疫抑制治疗或放疗等，应在医生的指导下决定是否接种乙肝疫苗。 4. 妊娠期的女性：妊娠期的女性可以接种乙肝疫苗，但最好在怀孕前进行接种。如果在怀孕期间接种乙肝疫苗，应在医生的建议下进行决定。 5. 癫痫患者：有癫痫病史的人，接种乙肝疫苗时需要谨慎，应在医生的建议下进行决定。 需要注意的是，以上禁忌证并非绝对，对于一些特殊情况，需要根据医生的具体建议来决定是否接种乙肝疫苗。同时，接种乙肝疫苗的剂量和接种方案也应根据个人情况来制定。 | No | No |  | No |
| 35 | Short Answer Questions | Why is the hepatitis B vaccine recommended for all babies? | The hepatitis B vaccine is recommended for all babies because infants and young children are at high risk of hepatitis B virus (HBV) infection and are more likely to develop chronic infection, which can lead to serious liver damage and other health complications. Here are some reasons why the hepatitis B vaccine is recommended for all babies: 1.Most cases of chronic hepatitis B infection occur in people who were infected during childhood: Infants and young children who are infected with HBV are more likely to develop chronic infection than adults, with up to 90% of infected infants developing chronic infection. Chronic hepatitis B infection can lead to serious liver damage, liver cancer, and other health complications later in life. 2.HBV can be transmitted from mother to child during childbirth: Infants born to mothers who are infected with HBV are at high risk of developing chronic hepatitis B infection if they are not vaccinated at birth. The hepatitis B vaccine, along with hepatitis B immune globulin, can help prevent mother-to-child transmission of HBV. 3.The vaccine is safe and effective: The hepatitis B vaccine is safe and highly effective at preventing HBV infection and its complications, including liver cancer and cirrhosis. The vaccine is given in a series of three doses, with the first dose given at birth and the subsequent doses given at 1-2 months and 6-18 months of age. 4.Vaccination can help prevent the spread of HBV: Vaccination of all infants and young children can help prevent the spread of HBV in the community and reduce the overall incidence of HBV infection. In summary, the hepatitis B vaccine is recommended for all babies because it is safe and highly effective at preventing HBV infection and its complications, and infants and young children are at high risk of HBV infection and chronic infection. | The hepatitis B vaccine is recommended for all babies because hepatitis B virus (HBV) infection can cause serious liver disease, including chronic hepatitis B, cirrhosis, and liver cancer. The virus is highly infectious and can be transmitted from an infected mother to her baby during childbirth, or through contact with infected blood or other bodily fluids. Babies who become infected with HBV are at high risk of developing chronic infection, which can lead to liver damage and other complications later in life. In contrast, babies who receive the hepatitis B vaccine are protected from infection and the serious health consequences that can result from HBV. The hepatitis B vaccine is given in a series of three doses, with the first dose given at birth, the second dose given at 1-2 months of age, and the third dose given at 6-18 months of age. The vaccine is safe and effective, and studies have shown that it can prevent up to 95% of cases of chronic hepatitis B infection when given to infants. In addition to protecting individual babies from HBV infection, universal vaccination with the hepatitis B vaccine can also help reduce the overall prevalence of the virus in the population and prevent transmission to others, including family members, sexual partners, and healthcare workers. Overall, the hepatitis B vaccine is a safe and effective way to protect babies from the serious health consequences of HBV infection and to help prevent the spread of the virus in the community. | Yes | Yes |  | Yes |
| 35 | Short Answer Questions | 简述所有婴幼儿推荐接种乙肝疫苗的原因 | 所有婴幼儿推荐接种乙肝疫苗的原因主要有以下几点： 1. 婴幼儿易受到乙肝病毒感染：乙肝病毒主要通过血液和体液途径传播，而婴幼儿往往无法控制自己的行为和环境，容易接触不洁血液和体液，从而易受到乙肝病毒感染。 2. 乙肝病毒感染后易导致慢性肝炎和肝癌：乙肝病毒感染后，一部分人可能会出现乙肝慢性肝炎，长期慢性感染可能导致肝硬化、肝癌等严重疾病。 3. 婴幼儿对乙肝疫苗有较好的免疫效果：乙肝疫苗在婴幼儿接种后，可以刺激其免疫系统产生足够的抗体，建立起有效的免疫保护，有效预防乙肝病毒感染和疾病发展。 4. 早期接种能够有效避免乙肝病毒的传播：由于乙肝病毒可以经过母婴传播，通过早期接种乙肝疫苗，可以有效地避免母婴传播，减少乙肝病毒在儿童之间的传播和流行。 综上所述，婴幼儿推荐接种乙肝疫苗是为了预防乙肝病毒感染和疾病发展，有效保护儿童的健康和生命安全。此外，接种乙肝疫苗也是预防乙肝病毒在儿童之间的传播和流行的重要措施，对于维护社会公共卫生和稳定也具有重要的意义。 | 以下是所有婴幼儿推荐接种乙肝疫苗的原因： 1. 预防乙肝病毒感染：乙肝病毒可以通过母婴传播、医疗器械传播等多种途径感染人体，而接种乙肝疫苗可以预防婴幼儿感染乙肝病毒，从而减少乙肝的发病率和传播风险。 2. 免疫保护期长：乙肝疫苗接种后，能够产生长期的免疫保护作用，即使是在婴幼儿期接种，也能够维持长达十几年的免疫力，有效降低儿童和成人感染乙肝病毒的风险。 3. 提高疫苗接种率：婴幼儿是接种疫苗的优先对象，他们的免疫系统比成人更为脆弱，而且在婴幼儿时期接种乙肝疫苗可以有效提高疫苗接种率，为后续的疫苗接种打下坚实的基础。 4. 降低治疗成本和治疗难度：乙肝是一种慢性肝病，需要长期的治疗和护理，而接种乙肝疫苗可以有效预防乙肝病毒感染，避免病情恶化，从而降低治疗成本和治疗难度。 因此，根据世界卫生组织和各国卫生部门的建议，所有婴幼儿都应该接种乙肝疫苗，以保护他们的健康和生命安全。 | Yes | No | No | No |
| 36 | Short Answer Questions | Can a person spread hepatitis B without having symptoms? | Yes, a person infected with hepatitis B virus (HBV) can spread the virus to others even if they do not have any symptoms. This is because people with HBV can be asymptomatic, meaning they do not have any noticeable symptoms of the infection, but still have the virus in their blood and can transmit it to others. In fact, many people with chronic hepatitis B infection may not have any symptoms for years or even decades, but can still spread the virus to others through blood or other bodily fluids such as semen and vaginal secretions. In addition, people with acute hepatitis B infection may also be asymptomatic or have mild symptoms that are easily mistaken for other illnesses. This is why it is important for people who are at risk of HBV infection, such as healthcare workers, people who inject drugs, and those who have unprotected sex or share needles or other injection equipment, to get tested for HBV infection and take precautions to prevent transmission of the virus to others. Preventive measures such as vaccination, practicing safe sex, avoiding sharing of needles or other injection equipment, and using precautions to avoid exposure to infected blood and bodily fluids can help reduce the risk of HBV infection and transmission. | Yes, a person with hepatitis B (HBV) can spread the virus even if they have no symptoms. This is because HBV is highly infectious and can be transmitted through contact with infected blood or other bodily fluids, such as semen and vaginal secretions. In fact, many people with HBV have no symptoms at all, or they may have mild symptoms that go unnoticed or are mistaken for other illnesses. As a result, they may unknowingly transmit the virus to others, including family members, sexual partners, or healthcare workers. People with chronic hepatitis B are particularly at risk of spreading the virus, as they may have high levels of the virus in their blood even if they have no symptoms. It's important to practice safe sex, not share needles or other drug injection equipment, and get vaccinated against HBV to prevent transmission of the virus. If you think you may have been exposed to HBV, it's important to get tested and speak with a healthcare provider about appropriate treatment and management options. Early diagnosis and treatment can help prevent liver damage and the progression of the disease. | Yes | Yes |  | Yes |
| 36 | Short Answer Questions | 简述无症状者能否传播乙肝 | 是的，无症状者也能够传播乙肝。乙肝病毒携带者分为两类：有症状者和无症状者。有症状者指的是出现明显乙肝病毒感染症状的人，例如黄疸、乏力、食欲不振等。而无症状者指的是没有出现乙肝病毒感染症状的人，但是他们身体内仍然携带乙肝病毒，并且能够通过血液和其他体液途径传播乙肝病毒。 虽然无症状者不会出现乙肝病毒感染症状，但是他们的体内仍然存在乙肝病毒，因此可以通过血液、性接触、母婴传播等途径感染其他人。尤其是在血液透析、手术、注射等操作中，无症状者传播乙肝病毒的风险更高。因此，无症状者同样需要接受乙肝病毒检测和治疗，以防止乙肝病毒的传播和疾病的发生。 | 是的，无症状者也可以传播乙肝病毒。事实上，许多乙肝病毒感染者一开始可能并没有任何明显的症状，这种情况下被称为潜伏感染。在这种情况下，病毒仍然存在于患者体内并且可以通过血液、性行为或母婴传播途径传播给其他人。 此外，即使无症状感染者后来出现了症状，他们在感染之初的数周或数月内也有可能传染病毒给其他人，因为在这个时间段内乙肝病毒的病毒载量可能很高，而且体液中含有高浓度的病毒。因此，即使没有明显症状的患者也应该采取必要的预防措施，如使用安全套、避免共用牙刷、剃须刀、针头等个人卫生用品，以及避免共用注射器等医疗设备。同时，接种乙肝疫苗也是预防乙肝感染的重要措施。 | Yes | Yes |  | Yes |
| 37 | Short Answer Questions | How is acute (short-term) hepatitis B treated? | Acute (short-term) hepatitis B is a self-limiting infection, which means that in most cases, the body's immune system can clear the virus on its own without specific treatment. However, supportive care can help manage symptoms and prevent complications. Here are some ways to manage acute hepatitis B: 1.Rest: Resting and avoiding strenuous activities can help reduce fatigue and conserve energy. 2.Adequate nutrition and hydration: Eating a healthy diet and staying well hydrated can help support the body's immune system and promote recovery. 3.Medications to manage symptoms: Medications such as acetaminophen or nonsteroidal anti-inflammatory drugs (NSAIDs) may be recommended to relieve pain and fever. However, it is important to consult a healthcare provider before taking any medications, as some drugs can cause liver damage or interact with other medications. 4.Avoiding alcohol and drugs: Alcohol and certain drugs can cause liver damage and should be avoided during acute hepatitis B infection. 5.Monitoring liver function: Regular monitoring of liver function, through blood tests, can help assess the severity of the infection and monitor for complications. In some cases, antiviral medications may be prescribed to treat acute hepatitis B, particularly in people who have a high risk of developing chronic infection. However, antiviral treatment is generally not recommended for all cases of acute hepatitis B and should be evaluated on a case-by-case basis. It is important to consult a healthcare provider for proper diagnosis and treatment of acute hepatitis B. People with acute hepatitis B should also take precautions to prevent the spread of the virus to others, such as avoiding sharing of needles or other injection equipment, practicing safe sex, and using precautions to avoid exposure to infected blood and bodily fluids. | Acute hepatitis B (HBV) infection is a short-term illness that typically resolves on its own without specific treatment. However, there are several things that can be done to help manage the symptoms and support the body's natural healing process. Here are some common treatments for acute HBV: Rest: It's important to get plenty of rest and avoid strenuous physical activity, which can exacerbate symptoms and delay recovery. Good nutrition: Eating a healthy diet that includes plenty of fruits and vegetables can help support the body's immune system and promote healing. Hydration: Drinking plenty of fluids, such as water or herbal tea, can help prevent dehydration and promote overall health. Medications: In some cases, medications may be used to help manage symptoms of acute HBV, such as fever or pain. However, it's important to talk to a healthcare provider before taking any over-the-counter or prescription medications. Hospitalization: In rare cases, acute HBV infection can cause severe symptoms or complications, such as liver failure or encephalopathy, which may require hospitalization and supportive care. It's important to note that while acute HBV infection usually resolves on its own, some people may develop chronic HBV infection, which can lead to serious liver disease. If you think you may have been exposed to HBV or are experiencing symptoms of acute hepatitis B, it's important to speak with a healthcare provider for appropriate testing and management. Early diagnosis and treatment can help prevent liver damage and the progression of the disease. | No | No |  | No |
| 37 | Short Answer Questions | 简述急性乙肝治疗方案 | 急性乙肝的治疗方案主要包括以下几个方面： 1. 对症治疗：急性乙肝患者在发病初期，可能会出现乏力、食欲不振、恶心、呕吐等症状，这些症状可以通过对症治疗缓解。例如，口服解热镇痛药缓解发热和疼痛，抗恶心药物缓解恶心和呕吐等。 2. 应用抗病毒药物：对于急性乙肝患者，如果血清HBV-DNA水平较高，可以使用抗病毒药物进行治疗。常用的抗病毒药物包括拉米夫定、恩替卡韦、阿德福韦等。这些药物能够有效地抑制乙肝病毒的复制和繁殖，缩短病程，降低慢性乙肝的发生率。 3. 加强营养支持：急性乙肝患者在病程中可能会出现食欲不振等症状，因此需要加强营养支持，保证足够的营养和能量摄入，促进身体的康复。 需要注意的是，对于急性乙肝患者，治疗方案需要根据患者具体情况而定。如果病情较轻，通常可以进行对症治疗和观察，等待身体自行恢复。如果病情较重，需要进行综合治疗，包括对症治疗、抗病毒治疗、加强营养支持等措施。在治疗过程中，还需要定期进行血清HBV-DNA、ALT等指标的监测，以及注意药物的不良反应和副作用。 | 急性乙肝的治疗方案包括以下几个方面： 1. 对症治疗：根据患者的具体症状，可以采取对症治疗的措施，如发热时降温、口干时多饮水、恶心呕吐时口服抗恶心药等。 2. 休息和营养支持：在治疗期间，患者需要休息和适当的营养支持，以促进身体的康复和恢复。 3. 抗病毒治疗：对于急性乙肝患者，抗病毒治疗并不是必需的，因为多数患者会自愈。但对于高危人群、症状严重或病毒携带者等，抗病毒治疗是必要的。当前常用的抗病毒药物有阿德福韦、恩替卡韦等。 4. 预防肝功能损害：急性乙肝患者有可能出现肝功能损害，需要进行监测，并根据情况给予相应的治疗，如给予肝脏保护剂、补充足够的维生素等。 需要注意的是，急性乙肝的治疗方案应根据患者的具体情况进行个体化的制定和调整，同时在治疗期间需要密切观察患者的病情变化，并采取及时的措施，以保证治疗的效果和患者的安全。 | No | No |  | No |
| 38 | Short Answer Questions | How is chronic hepatitis B treated? | Chronic hepatitis B is a long-term infection caused by the hepatitis B virus (HBV), which can lead to liver damage, cirrhosis, and liver cancer if left untreated. Treatment for chronic hepatitis B aims to reduce the amount of virus in the blood, prevent liver damage and complications, and improve long-term outcomes. Here are some common treatments for chronic hepatitis B: 1.Antiviral medications: Antiviral medications, such as tenofovir and entecavir, can help reduce the amount of virus in the blood and prevent liver damage. These medications are usually taken orally, on a daily basis, and may need to be taken for several years. 2.Interferon injections: Interferon injections can help boost the body's immune system and reduce the amount of virus in the blood. However, interferon injections are less commonly used due to their side effects and limited effectiveness compared to antiviral medications. 3.Regular monitoring: People with chronic hepatitis B should undergo regular monitoring of liver function and virus levels, through blood tests and other imaging studies, to assess the severity of the infection and monitor for complications. 4.Liver cancer screening: People with chronic hepatitis B are at increased risk of developing liver cancer, and should undergo regular screening tests such as ultrasound and alpha-fetoprotein blood tests to detect any signs of cancer early. 5.Lifestyle modifications: People with chronic hepatitis B should avoid alcohol and certain drugs that can cause liver damage, and maintain a healthy diet and exercise routine to promote liver health. It is important to consult a healthcare provider for proper diagnosis and treatment of chronic hepatitis B. Treatment for chronic hepatitis B is usually long-term and may need to be adjusted based on individual response to treatment and disease progression. In addition, people with chronic hepatitis B should take precautions to prevent the spread of the virus to others, such as avoiding sharing of needles or other injection equipment, practicing safe sex, and using precautions to avoid exposure to infected blood and bodily fluids. | Chronic hepatitis B (CHB) is a viral infection of the liver that can lead to liver damage, cirrhosis, and liver cancer. The goal of treatment for CHB is to suppress the virus and prevent liver damage. Treatment options for CHB include antiviral medications, immune modulators, and regular monitoring. 1.Antiviral medications: Antiviral medications are the mainstay of treatment for CHB. They work by suppressing the replication of the virus and reducing the amount of virus in the blood. There are several antiviral medications available for CHB, including oral medications such as entecavir, tenofovir, and lamivudine. 2.Immune modulators: Immune modulators are medications that work by modulating the immune system's response to the virus. They can help reduce liver inflammation and prevent liver damage. Examples of immune modulators include interferon alpha-2a and pegylated interferon alpha-2a. 3.Regular monitoring: Regular monitoring is important for people with CHB to assess liver function, viral load, and the progression of liver disease. Monitoring may include blood tests, liver function tests, imaging studies, and liver biopsy. In addition to medical treatment, people with CHB are also advised to make lifestyle changes, such as avoiding alcohol and maintaining a healthy diet and exercise routine. People with CHB should also be vaccinated against hepatitis A to reduce the risk of co-infection. It's important to speak with a healthcare provider if you have CHB or think you may be at risk of CHB. Early diagnosis and treatment can help prevent liver damage and the progression of the disease. | No | No |  | No |
| 38 | Short Answer Questions | 简述慢性乙肝治疗方案 | 慢性乙肝的治疗方案主要包括以下几个方面： 1. 应用抗病毒药物：慢性乙肝病毒携带者需要长期应用抗病毒药物进行治疗。常用的抗病毒药物包括拉米夫定、恩替卡韦、阿德福韦、替比夫定等。这些药物能够有效地抑制乙肝病毒的复制和繁殖，降低血清HBV-DNA水平，防止肝病的发展和恶化。 2. 肝保护治疗：慢性乙肝患者还需要进行肝保护治疗，促进肝细胞修复和再生。常用的肝保护药物包括乙肝康复液、肝泰乐等，这些药物能够促进肝细胞代谢和修复，降低血清ALT水平，缓解肝脏病变的程度。 3. 对症治疗：对于慢性乙肝患者，在病程中可能会出现疲劳、肝区疼痛、食欲不振等症状，这些症状可以通过对症治疗缓解。 4. 加强营养支持：慢性乙肝患者需要加强营养支持，保证足够的营养和能量摄入，促进身体的康复。 需要注意的是，慢性乙肝的治疗需要长期进行，并且具有一定的复杂性和难度。在治疗过程中，需要定期进行血清HBV-DNA、ALT等指标的监测，以及注意药物的不良反应和副作用。另外，慢性乙肝病毒携带者还需要注意个人卫生，避免感染其他病毒和细菌，以减少肝病的发展和恶化。 | 慢性乙肝的治疗方案因患者个体差异而有所不同，需要根据病情、年龄、肝功能等情况进行个体化制定。一般而言，慢性乙肝的治疗方案包括以下几个方面： 1. 抗病毒治疗：对于乙肝病毒DNA水平较高的患者，抗病毒治疗是常见的治疗方法，可用于控制乙肝病毒复制和减轻病情。目前常用的抗病毒药物包括恩替卡韦、阿德福韦等。 2. 肝保护治疗：对于肝功能不全的患者，肝保护治疗是必不可少的，包括补充维生素和氨基酸、保肝药物等。 3. 饮食调理：慢性乙肝患者应该注意饮食调理，避免摄入过多的脂肪、糖分等，同时增加蛋白质的摄入。 4. 心理疏导：慢性乙肝治疗期间，患者可能会面临一些心理压力和负担，应该及时进行心理疏导和支持。 需要注意的是，慢性乙肝是一种长期的慢性疾病，治疗需要长期持续，并且疗效也因人而异。患者应该积极配合医生的治疗，同时加强自我护理，避免过度劳累、过度饮酒等行为，以维护肝功能和健康。 | No | No |  | No |
| 39 | Short Answer Questions | Symptoms of early cirrhosis | Cirrhosis is a late-stage liver disease that can develop over several years in people with chronic hepatitis B infection. In the early stages of cirrhosis, there may not be any noticeable symptoms. However, as the disease progresses, the liver becomes increasingly damaged and scarred, which can lead to the following symptoms: 1.Fatigue: Feeling tired or weak, even after getting enough rest, is a common symptom of early cirrhosis. 2.Loss of appetite: People with early cirrhosis may experience a loss of appetite, nausea, and vomiting. 3.Abdominal pain and bloating: As the liver becomes more damaged, it can cause abdominal pain and bloating. 4.Swelling: Swelling in the legs, ankles, and feet, or swelling in the abdomen, may occur due to a buildup of fluids in the body. 5.Jaundice: Jaundice is a condition where the skin and eyes become yellowish due to a buildup of bilirubin in the blood. It can be a sign of liver damage. 6.Itching: Itching, or pruritus, can be a symptom of liver damage and may be caused by a buildup of toxins in the blood. 7.Mental confusion: As cirrhosis progresses, it can lead to mental confusion, forgetfulness, and difficulty concentrating. It is important to consult a healthcare provider if you experience any of these symptoms, as early diagnosis and treatment can help slow or even reverse the progression of cirrhosis. People with chronic hepatitis B infection should undergo regular monitoring of liver function and virus levels to detect any signs of cirrhosis early. | Cirrhosis is a late-stage liver disease that results from long-term liver damage. In the early stages of cirrhosis, there may be no symptoms, or the symptoms may be mild and non-specific. However, as the disease progresses, the liver becomes more damaged, and symptoms become more severe. Here are some early symptoms of cirrhosis: 1.Fatigue: Fatigue is a common symptom of early cirrhosis. As the liver becomes damaged, it may not be able to produce enough energy to support the body's needs, leading to fatigue and weakness. 2.Abdominal pain: In early cirrhosis, you may experience mild or moderate pain in the upper right side of the abdomen, where the liver is located. The pain may be dull or sharp and may come and go. 3.Loss of appetite: As the liver becomes more damaged, you may lose your appetite and have trouble eating. This can lead to weight loss and malnutrition. 4.Nausea and vomiting: Nausea and vomiting are common symptoms of cirrhosis, especially in the early stages. These symptoms can be caused by the buildup of toxins in the body due to liver dysfunction. 5.Itchy skin: In early cirrhosis, you may experience itchy skin, which is caused by the buildup of bile salts in the body. 6.Spider angiomas: Spider angiomas are small, red, spider-like blood vessels that can appear on the skin. They are a common early sign of cirrhosis. It's important to speak with a healthcare provider if you experience any symptoms of liver disease, including early cirrhosis symptoms. Early diagnosis and treatment can help prevent further liver damage and the progression of the disease. | No | No |  | No |
| 39 | Short Answer Questions | 简述早期肝硬化症状 | 早期肝硬化症状比较隐匿，可能不容易被察觉，常见的早期症状包括： 1. 疲劳：早期肝硬化患者常常感到疲倦，即使没有进行剧烈的身体活动也会感到非常疲劳。 2. 消化不良：早期肝硬化患者可能会出现消化不良症状，如胃部胀气、食欲不振、恶心和呕吐等。 3. 腹部不适：早期肝硬化患者可能会出现腹部不适的症状，如腹胀、腹泻、便秘和腹痛等。 4. 体重下降：早期肝硬化患者可能会出现不明原因的体重下降。 5. 皮肤瘙痒：早期肝硬化患者可能会出现皮肤瘙痒的症状，尤其是在晚上更为明显。 6. 皮肤和黏膜出血：早期肝硬化患者可能会出现皮肤和黏膜出血的症状，如牙龈出血、鼻出血等。 7. 肝区不适：早期肝硬化患者可能会出现肝区不适的症状，如肝区疼痛、肝区胀痛等。 需要注意的是，早期肝硬化的症状比较隐匿，有时可能被忽视或误诊，因此患者应该密切关注身体状况，定期进行体检和血液检查，及时发现和治疗肝硬化。同时，避免饮酒、保持健康的生活方式和饮食习惯，对于预防和延缓肝硬化的发生和发展也具有重要的意义。 | 早期肝硬化症状不明显，可能只有轻微的不适和体征。以下是一些早期肝硬化症状的常见表现： 1. 乏力：早期肝硬化患者可能会感觉疲乏、虚弱，体力逐渐下降。 2. 食欲不振：患者可能会出现食欲不振、恶心、呕吐等症状。 3. 体重减轻：由于食欲不振、恶心、呕吐等影响，患者体重可能会出现下降。 4. 上腹部不适：患者可能会感觉上腹部不适、胀气、腹胀等症状。 5. 黄疸：部分患者可能会出现轻度的黄疸症状，表现为眼白、皮肤发黄等。 6. 腹水：少数早期肝硬化患者可能会出现腹水症状，表现为腹部肿胀、腹部胀痛等。 需要注意的是，早期肝硬化症状不典型，不同患者的症状表现可能存在差异，因此及早进行检查和诊断是非常重要的。如果有上述症状或疑似肝硬化的情况，应尽早就医，接受专业的检查和诊断。 | Yes | No | No | No |
| 40 | Clinical Problems | How are HBV infectious, cirrhosis and hepatocellular carcinoma related? | HBV (Hepatitis B virus) is a virus that infects the liver and can lead to a range of liver diseases, including cirrhosis and hepatocellular carcinoma (HCC). HBV is highly infectious and can be transmitted through blood or bodily fluids, including sexual contact, sharing needles or syringes, and from mother to baby during childbirth. Chronic HBV infection can cause liver damage over time, leading to cirrhosis. Cirrhosis is a condition in which the liver becomes scarred and hardened, making it difficult for the liver to function properly. This can lead to a range of complications, including fluid buildup in the abdomen, bleeding in the digestive tract, and increased risk of liver cancer. HCC is the most common type of liver cancer, and it is strongly associated with chronic HBV infection. Over time, the inflammation and liver damage caused by chronic HBV infection can increase the risk of developing HCC. In summary, HBV is a highly infectious virus that can cause liver damage and lead to cirrhosis and HCC over time. It is important to get tested for HBV and to take steps to prevent transmission, such as practicing safe sex and avoiding sharing needles or syringes. Vaccines are also available to prevent HBV infection. | HBV (Hepatitis B virus) is a viral infection that attacks the liver and can cause both acute and chronic hepatitis. The virus is transmitted through contact with infected blood, semen, and other body fluids. Chronic HBV infection can lead to liver cirrhosis, which is the scarring of the liver tissue that occurs over time due to ongoing liver damage. Cirrhosis can cause liver failure and can increase the risk of developing hepatocellular carcinoma (HCC), which is a type of liver cancer. HBV is a major risk factor for developing HCC. About 80% of HCC cases are associated with chronic HBV infection. Chronic infection causes damage to the liver, which increases the risk of HCC. The risk of developing HCC is highest in people with both HBV infection and cirrhosis. Overall, HBV infection can lead to liver damage, cirrhosis, and HCC. It is important to get vaccinated against HBV and to take precautions to prevent its transmission, such as practicing safe sex, not sharing needles or other injection equipment, and getting screened for HBV if you are at risk. Early detection and treatment of HBV infection can help reduce the risk of developing cirrhosis and HCC. | No | No |  | No |
| 40 | Clinical Problems | 乙肝感染与肝硬化、肝癌有何关系？ | 乙肝病毒感染与肝硬化、肝癌之间有密切的关系。以下是具体的解释： 1.肝硬化是乙肝感染的常见后遗症之一。当肝细胞受到乙肝病毒的长期侵害时，会引起肝细胞损伤和炎症反应，最终导致肝组织纤维化和硬化，形成肝硬化。 2.乙肝病毒感染是导致肝癌的主要原因之一。长期慢性乙肝病毒感染会导致肝细胞的慢性损伤和再生，这增加了肝细胞癌变的风险。据统计，慢性乙肝感染者患肝癌的风险是普通人的100倍以上。 3.患有乙肝病毒感染的肝硬化患者，患肝癌的风险更高。肝硬化患者的肝细胞已经受到了严重的损伤和纤维化，这会导致肝癌的发生率显著增加。而且，肝硬化患者更容易发展成为肝癌。 因此，对于乙肝感染者，及时采取措施进行治疗和预防，可以有效地降低肝硬化和肝癌的发生率。同时，对于已经患有肝硬化和肝癌的患者，也需要积极治疗和管理疾病，以延缓疾病进展和提高生活质量。 | 乙肝病毒感染是引起肝硬化和肝癌的主要原因之一。乙肝病毒通过血液和其他体液传播，感染后可导致肝炎和肝损伤。长期的乙肝病毒感染会导致肝脏的纤维化和硬化，最终发展成为肝硬化。 肝硬化是一种肝脏疾病，其特征是肝脏组织的纤维化和硬化，导致肝脏结构和功能的受损。乙肝病毒感染是肝硬化的主要原因之一，其发生率比其他原因更高。 乙肝病毒感染也是肝癌的主要原因之一。长期的乙肝病毒感染会导致肝细胞的DNA损伤和突变，从而增加肝癌的风险。乙肝病毒感染与肝癌的风险存在一定的剂量-反应关系，即感染乙肝病毒的时间越长，病毒载量越高，发生肝癌的风险越大。 因此，对于乙肝病毒感染者来说，及早诊断、积极治疗和定期检查是预防肝硬化和肝癌的关键。同时，疫苗接种是预防乙肝病毒感染最有效的方法之一，建议所有未感染乙肝病毒的人群接种乙肝疫苗。 | No | No |  | No |
| 41 | Clinical Problems | Give advice on usual tests for hepatitis B virus patients | If someone is diagnosed with Hepatitis B virus, their healthcare provider may recommend certain tests to monitor their condition and determine the best course of treatment. The specific tests recommended may vary depending on the individual's situation, but here are some usual tests that may be ordered: HBsAg (Hepatitis B surface antigen) test: This test detects the presence of the hepatitis B virus in the blood. If the result is positive, it means that the person is infected with the virus. HBV DNA (Hepatitis B virus DNA) test: This test measures the amount of virus in the blood. It helps to determine the severity of the infection and the effectiveness of treatment. Liver function tests: These tests measure the levels of various enzymes and proteins produced by the liver. Abnormal levels may indicate liver damage caused by the virus. HBeAg (Hepatitis B e antigen) test: This test detects a protein produced by the virus. If the result is positive, it means that the virus is actively replicating and the person is highly infectious. Anti-HBc (Hepatitis B core antibody) test: This test detects antibodies produced by the immune system in response to the virus. A positive result indicates a past or current infection. Anti-HBs (Hepatitis B surface antibody) test: This test detects antibodies produced by the immune system after vaccination or recovery from a past infection. A positive result indicates immunity to the virus. It is important to follow your healthcare provider's recommendations for testing and treatment of Hepatitis B virus to minimize the risk of liver damage and other complications. | If you or someone you know has been diagnosed with hepatitis B virus (HBV), there are several tests that may be recommended by a healthcare provider to monitor the condition and assess the progression of the disease. Here are some common tests and what they are used for: Hepatitis B surface antigen (HBsAg) test: This test is used to detect the presence of the HBV virus in the blood. A positive result means that the person is currently infected with HBV. Hepatitis B surface antibody (anti-HBs) test: This test measures the amount of antibodies in the blood that are produced in response to a previous HBV infection or vaccination. A positive result means that the person is immune to HBV. Hepatitis B e antigen (HBeAg) test: This test is used to determine if a person with HBV is actively replicating the virus. A positive result means that the person is highly infectious. Hepatitis B viral load test: This test measures the amount of HBV in the blood. It is used to monitor the progression of the disease and the effectiveness of treatment. Liver function tests: These tests measure levels of enzymes and proteins in the blood that indicate liver health. HBV can cause liver damage, so these tests are used to monitor the condition of the liver. It's important to work closely with a healthcare provider to determine which tests are necessary and how frequently they should be done. Treatment options for HBV may depend on the results of these tests, so it's important to keep up with regular monitoring to ensure the best possible outcomes. | Yes | Yes |  | Yes |
| 41 | Clinical Problems | 给出乙肝常规检查项目的建议 | 乙肝通常的检查项目包括： 1.HBsAg（乙肝表面抗原）：是检测乙型肝炎病毒是否存在的最常用指标，如果检测结果为阳性，则说明病毒存在。 2.HBeAg（乙肝e抗原）：检测病毒活跃性，如果检测结果为阳性，则说明病毒活跃性高，病情较为严重。 3.抗-HBc（乙肝核心抗体）：检测曾经是否感染过乙型肝炎病毒，如果检测结果为阳性，则说明曾经感染过乙型肝炎病毒。 4.抗-HBs（乙肝表面抗体）：检测身体是否已经产生了免疫力，如果检测结果为阳性，则说明身体已经产生了对乙型肝炎病毒的免疫力。 5.ALT（谷丙转氨酶）：是肝功能指标之一，如果ALT值升高，则说明肝细胞受到损伤。 6.AST（谷草转氨酶）：也是肝功能指标之一，如果AST值升高，则说明肝细胞受到损伤。 7.总胆红素：如果总胆红素升高，则说明肝脏排泄胆红素的能力降低，可能与乙肝感染有关。 除了上述常规检查项目外，根据具体病情和医生的建议，可能还需要进行肝脏B超、肝功能、肝穿刺等检查。 | 乙肝的常见检查项目包括： 1.乙肝表面抗原（HBsAg）：检测是否携带乙型肝炎病毒。 2.乙肝表面抗体（HBsAb）：检测是否对乙型肝炎病毒产生了免疫应答，即是否有乙肝疫苗接种史或曾经治愈过乙肝。 3.乙肝e抗原（HBeAg）：检测是否有正在活跃的乙型肝炎病毒感染。 4.乙肝e抗体（HBeAb）：检测是否已经恢复或控制住了乙型肝炎病毒感染。 5.乙肝核心抗体（HBcAb）：检测是否曾经感染过乙型肝炎病毒。 此外，还有肝功能检查、肝脏影像学检查（如B超、CT、MRI等）、肝穿刺活检等检查项目。具体检查项目和方案需要根据医生的建议进行选择。 | No | No |  | No |
| 42 | Clinical Problems | Explain clinical signification of each item in five indicator test for Hepatitis B | 1.The five indicator tests for Hepatitis B include: 2.Hepatitis B surface antigen (HBsAg) 3.Hepatitis B surface antibody (anti-HBs) 4.Hepatitis B core antibody (anti-HBc) 5.Hepatitis B e antigen (HBeAg) 6.Hepatitis B e antibody (anti-HBe) Each of these indicators plays a crucial role in the clinical significance of the disease: HBsAg: The presence of HBsAg indicates an active Hepatitis B infection. It is the earliest and most reliable indicator of acute infection and is detectable before symptoms appear. In chronic infections, HBsAg is present for more than six months. Anti-HBs: The presence of anti-HBs indicates that the person has been exposed to the Hepatitis B virus and has developed immunity against it. It can be due to a past infection or vaccination. Anti-HBc: The presence of anti-HBc indicates previous or ongoing Hepatitis B infection, whether acute or chronic. It is detectable during the window period, when HBsAg has cleared, but anti-HBs has not yet developed. HBeAg: The presence of HBeAg indicates an active and highly infectious phase of Hepatitis B. It indicates a high viral load and a high risk of transmission. It is most commonly found in acute infections and in chronic infections during the immune-tolerant phase. Anti-HBe: The presence of anti-HBe indicates a low infectious phase of Hepatitis B. It indicates a lower viral load and a lower risk of transmission. It is most commonly found in chronic infections during the immune clearance phase or after HBeAg seroconversion. Overall, these five indicators are used to diagnose, monitor and manage Hepatitis B infections. They provide valuable information on the stage and severity of the infection, as well as the likelihood of transmission and response to treatment. | The five indicator tests for Hepatitis B are: HBsAg (Hepatitis B surface antigen): This test is used to detect the presence of Hepatitis B virus in the blood. A positive HBsAg test result indicates that the person has an active Hepatitis B infection. It is the first sign of infection and can appear as early as 1-2 weeks after exposure. Anti-HBc (Antibody to Hepatitis B core antigen): This test detects the presence of antibodies to the Hepatitis B core antigen. If the result is positive, it indicates that the person has been infected with the virus at some point in the past. It can also indicate a current or recent infection, especially if the HBsAg test is negative. HBeAg (Hepatitis B e antigen): This test detects a protein produced by the Hepatitis B virus. A positive result indicates that the person is highly infectious and can easily transmit the virus to others. This test is used to monitor the progression of the disease and the effectiveness of treatment. Anti-HBe (Antibody to Hepatitis B e antigen): This test detects the presence of antibodies to the Hepatitis B e antigen. A positive result indicates that the person is no longer infectious and is in the recovery phase of the disease. It can also indicate successful treatment. HBV DNA (Hepatitis B virus DNA): This test detects the presence of Hepatitis B virus DNA in the blood. It is used to monitor the amount of virus in the blood and to determine the effectiveness of treatment. A high viral load indicates an active infection, while a low viral load indicates a less active infection or successful treatment. Overall, these five indicator tests are important for the diagnosis and monitoring of Hepatitis B infection. They provide information about the presence and activity of the virus in the body, as well as the immune response to the virus. | Yes | Yes |  | Yes |
| 42 | Clinical Problems | 解释乙肝五项检测中每个指标的临床意义 | 乙肝五项指的是血清乙型肝炎病毒表面抗原（HBsAg）、血清乙型肝炎病毒表面抗体（anti-HBs）、血清乙型肝炎病毒e抗原（HBeAg）、血清乙型肝炎病毒e抗体（anti-HBe）和血清乙型肝炎病毒核心抗体（anti-HBc）五项检测结果。以下是每一项的临床意义： 1.血清乙型肝炎病毒表面抗原（HBsAg）：该项检测结果是判断患者是否感染了乙型肝炎病毒的重要指标。如果检测结果为阳性，说明患者已经感染了乙型肝炎病毒，可能出现肝炎症状，并有可能成为慢性乙肝病毒携带者。 2.血清乙型肝炎病毒表面抗体（anti-HBs）：该项检测结果是判断患者是否具备乙肝病毒免疫保护能力的指标。如果检测结果为阳性，说明患者已经接种了乙肝疫苗或曾经感染过乙肝病毒，并已经产生了相应的抗体，具有免疫保护能力。 3.血清乙型肝炎病毒e抗原（HBeAg）：该项检测结果是判断患者乙肝病毒复制状态的指标。如果检测结果为阳性，说明患者目前处于乙肝病毒复制期，具有较高的传染性，需及时进行治疗。 4.血清乙型肝炎病毒e抗体（anti-HBe）：该项检测结果是判断患者乙肝病毒感染后病情变化的指标。如果检测结果为阳性，说明患者已经经历了乙肝病毒复制期，进入了免疫清除期，病情较为稳定。 5.血清乙型肝炎病毒核心抗体（anti-HBc）：该项检测结果是判断患者是否曾经感染过乙肝病毒的指标。如果检测结果为阳性，说明患者曾经感染过乙肝病毒，但并不能判断其是否已经痊愈或慢性感染。该项检测结果通常需要结合其他检查结果进行分析，以确定患者的乙肝感染情况。 需要注意的是，以上乙肝五项检测结果只是辅助诊断乙肝病毒感染的指标之一，诊断需要综合患者的病史、症状、体征和其他相关检查结果进行综合分析。如果有疑似乙肝病毒感染的症状，建议及时就医并进行相关检查，以确诊并进行治疗。 | 乙肝五项是指HBsAg（乙肝表面抗原）、HBsAb（乙肝表面抗体）、HBcAb（乙肝核心抗体）、HBeAg（乙肝e抗原）和HBeAb（乙肝e抗体）五个指标。下面是每个指标的临床意义： 1.HBsAg：乙肝表面抗原，检测结果阳性表示患者体内存在乙肝病毒，是乙肝病毒感染的重要标志，也是诊断乙肝病毒感染的首要指标。 2.HBsAb：乙肝表面抗体，检测结果阳性表示患者体内存在乙肝病毒表面抗体，一般情况下，这代表着患者已经免疫乙肝病毒，或者曾经感染过乙肝病毒，但已经痊愈。 3.HBcAb：乙肝核心抗体，检测结果阳性表示患者体内存在乙肝病毒核心抗体，这代表着患者曾经感染过乙肝病毒，但是否已经痊愈需要进一步观察和检查。 4.HBeAg：乙肝e抗原，检测结果阳性表示患者体内存在乙肝病毒e抗原，这通常代表着患者正在急性感染期，或者是慢性感染期的活动期。 5.HBeAb：乙肝e抗体，检测结果阳性表示患者体内存在乙肝病毒e抗体，这代表着患者已经对乙肝病毒e抗原产生了免疫反应，通常是慢性感染期的非活动期或已经治愈。 总之，乙肝五项检测结果的临床意义需要综合考虑，结合患者的临床表现和其他检查结果来评估患者的病情和治疗方案。 | Yes | Yes |  | Yes |
| 43 | Clinical Problems | Explain clinical signification of HBV-DNA quantification | HBV-DNA quantification is a laboratory test that measures the amount of hepatitis B virus (HBV) DNA in a patient's blood. This test is important in the clinical management of patients with chronic HBV infection because it provides important information about the disease progression and helps guide treatment decisions. Here are some of the key clinical implications of HBV-DNA quantification: Diagnosis of chronic HBV infection: A positive HBV-DNA test result is a key indicator of chronic HBV infection, which is defined as the presence of HBV DNA in the blood for more than 6 months. Monitoring disease progression: HBV-DNA levels in the blood are a key indicator of the activity of the virus and the degree of liver damage that is occurring. As such, HBV-DNA quantification is useful in monitoring disease progression and predicting the risk of complications such as cirrhosis and hepatocellular carcinoma. Guiding treatment decisions: The level of HBV-DNA in the blood is an important factor in determining the need for treatment and the choice of treatment options. Antiviral therapy is recommended for patients with high HBV-DNA levels and evidence of liver damage, while patients with low HBV-DNA levels may be monitored closely without treatment. Assessing treatment response: HBV-DNA quantification is also used to monitor the response to antiviral therapy. A decrease in HBV-DNA levels is a sign that the treatment is effective, while an increase in HBV-DNA levels may indicate treatment failure or the development of drug resistance. In summary, HBV-DNA quantification is an important laboratory test in the clinical management of patients with chronic HBV infection. It provides valuable information for diagnosis, monitoring disease progression, guiding treatment decisions, and assessing treatment response. | HBV-DNA quantification is a laboratory test that measures the amount of hepatitis B virus (HBV) DNA in a person's blood. This test is important in the clinical management of patients with chronic hepatitis B infection, as it provides information on the level of virus replication in the body and the effectiveness of antiviral therapy. The level of HBV-DNA in the blood is a key indicator of viral activity and can help predict disease progression, as well as response to treatment. For example, a high viral load indicates active viral replication and may suggest a higher risk of liver damage and progression to cirrhosis or hepatocellular carcinoma (HCC). In contrast, a low viral load suggests less active replication and a lower risk of disease progression. HBV-DNA quantification is also used to monitor the response to antiviral therapy, which is the mainstay of treatment for chronic hepatitis B. The goal of treatment is to suppress viral replication and reduce the risk of liver damage and HCC. By measuring HBV-DNA levels over time, clinicians can assess the effectiveness of therapy and adjust treatment as needed to optimize the patient's outcome. In summary, HBV-DNA quantification is a clinically significant test that provides important information for the diagnosis and management of chronic hepatitis B infection. It helps to assess the level of viral activity, predict disease progression, and monitor the response to antiviral therapy. | Yes | Yes |  | Yes |
| 43 | Clinical Problems | 解释HBV-DNA定量的临床意义，如何理解结果 | HBV-DNA定量是一种血液检测方法，用于测量乙型肝炎病毒（HBV）在患者体内的DNA含量。这项测试的结果可以提供有关患者肝炎病毒感染程度的信息，并用于评估疾病的进展和治疗方案的选择。 在HBV感染患者中，高水平的HBV-DNA通常与更严重的肝炎病变和疾病进展相关。此外，HBV-DNA定量也可以用来监测治疗效果。如果治疗有效，患者的HBV-DNA水平应该下降。如果HBV-DNA水平持续升高或未能降至指定水平，则可能需要调整治疗方案。 因此，HBV-DNA定量是评估HBV感染程度、预测疾病进展、确定治疗方案以及监测治疗效果的重要指标。 | HBV-DNA定量是测定患者血液中乙型肝炎病毒(HBV) DNA含量的实验室检测方法，可提供关于疾病进展的重要信息，帮助指导治疗决策，在慢性乙型肝炎病毒感染患者的临床管理中非常重要。病毒DNA阳性检测结果是慢性乙型肝炎病毒感染的关键指标，它标志着血液中HBV DNA的存在时间超过6个月。HBV-DNA水平是病毒活动和正在发生的肝损害程度的一个关键指标。因此，HBV-DNA定量有助于监测疾病进展和预测并发症(如肝硬化和肝细胞癌)的风险。HBV-DNA水平是决定是否需要治疗和选择治疗方案的重要因素。对于HBV-DNA水平高且有肝损害证据的患者建议抗病毒治疗，而HBV-DNA水平低的患者可在不治疗的情况下密切监测。 | Yes | Yes |  | Yes |
| 44 | Clinical Problems | Give clinical treatment advice for women of childbearing age infected with hepatitis B | The treatment of hepatitis B in women of childbearing age depends on several factors, such as the level of viral replication, the degree of liver damage, and the woman's pregnancy status. Here are some general treatment recommendations: Antiviral medications: Antiviral medications can help to reduce the replication of the hepatitis B virus in the body, which can help to prevent liver damage. Some antiviral medications that may be used include entecavir, tenofovir disoproxil fumarate (TDF), or tenofovir alafenamide (TAF). These medications are generally safe to use during pregnancy. Monitoring liver function: Women with chronic hepatitis B should have regular monitoring of their liver function through blood tests. This can help to detect any signs of liver damage and ensure that appropriate treatment is provided. Vaccination: If a woman is not already immune to hepatitis B, she should be vaccinated against the virus. Vaccination is safe to receive during pregnancy and can help to prevent transmission of the virus to the newborn. Counseling: Women with chronic hepatitis B should receive counseling about the risk of transmission of the virus to their partners and newborns. They should also be advised to avoid alcohol and other liver-damaging substances. Pregnancy planning: Women with chronic hepatitis B who are planning to become pregnant should be evaluated by their healthcare provider to ensure that their liver function is stable and that they are receiving appropriate treatment. Women who are pregnant should receive regular monitoring of their hepatitis B virus levels and liver function. It's important to note that the management of hepatitis B during pregnancy can be complex and should be done in consultation with a healthcare provider with experience in managing viral hepatitis. | Women of childbearing age who are infected with hepatitis B should receive prompt medical care and treatment to reduce the risk of transmitting the virus to their newborns. Here are some general clinical treatment advice: Antiviral medications: Antiviral medications such as tenofovir or entecavir can be used to suppress the replication of the hepatitis B virus and reduce the risk of liver damage. These medications are generally safe and effective for pregnant women with chronic hepatitis B. Monitoring liver function: Regular monitoring of liver function is recommended during pregnancy, particularly in women with chronic hepatitis B. This helps to detect any signs of liver damage or inflammation, which may require additional treatment. Hepatitis B immune globulin (HBIG): HBIG is a medication that contains antibodies against the hepatitis B virus. It is typically given to newborns of mothers with chronic hepatitis B within 12 hours of birth, along with the hepatitis B vaccine, to reduce the risk of transmission. Hepatitis B vaccine: The hepatitis B vaccine is recommended for all infants and children, as well as adults who are at increased risk of infection. It is safe and effective, and can provide long-term protection against the hepatitis B virus. Counseling and support: Women who are infected with hepatitis B may benefit from counseling and support to help them cope with the emotional and psychological effects of the disease. They may also need guidance on how to prevent transmission of the virus to others, including their sexual partners and household members. It's important to note that treatment for hepatitis B during pregnancy should be individualized based on the woman's medical history and the severity of her condition. Women with chronic hepatitis B should be closely monitored by a healthcare provider throughout their pregnancy and after delivery to ensure the best possible outcomes for both mother and baby. | Yes | Yes |  | Yes |
| 44 | Clinical Problems | 如果育龄女性感染乙肝，有何临床建议？ | 如果育龄女性感染了乙肝，需要根据具体情况制定相应的治疗方案。以下是一些可能的建议： 1.就医咨询：首先建议到医院进行检查和咨询，以确定乙肝感染的情况和病情严重程度。医生会根据检查结果和患者的具体情况制定相应的治疗方案。 2.接种疫苗：如果患者还没有接种乙肝疫苗，医生可能会建议接种。乙肝疫苗是预防乙肝感染的有效措施。 3.药物治疗：对于慢性乙肝感染的患者，医生可能会开具抗病毒药物进行治疗。这些药物可以帮助抑制病毒复制，减轻症状和延缓疾病的进展。 4.生活方式管理：乙肝感染者需要注意饮食和生活方式的管理。建议少喝酒、少吃油腻和刺激性食物，避免过度劳累，保持充足的睡眠等。 总之，对于育龄女性感染乙肝的情况，需要及时就医咨询，制定相应的治疗方案，注意生活方式管理，积极应对疾病。同时，建议定期复查，了解病情的变化情况。 | 如果育龄女性感染了乙肝，需要在咨询医生的指导下进行相应的治疗和管理。以下是一些可能有用的建议： 1.寻求专业医生的帮助：如果您怀疑自己感染了乙肝病毒，请尽快咨询医生。您的医生可以为您进行必要的检查和诊断，并制定适合您的治疗计划。 2.遵循医生的建议：如果您被诊断出携带乙肝病毒，您的医生可能会建议您接受抗病毒治疗，以减少病毒的复制和减轻症状。请务必遵循医生的建议，并遵守治疗计划。 3.接受乙肝疫苗接种：乙肝疫苗是一种预防乙肝感染的有效方法。如果您还没有接种过乙肝疫苗，那么接种疫苗可以帮助您预防病毒感染，并保护您的健康。 4.保持健康的生活方式：保持健康的生活方式可以帮助您保持身体健康，并降低患上乙肝病毒感染的风险。这包括：避免饮酒和吸烟，保持健康的饮食和锻炼习惯，避免性接触或使用安全套，避免分享注射器或其他注射用具。 总之，如果育龄女性感染了乙肝，需要及时寻求医生的帮助，遵循医生的建议进行治疗和管理，并保持健康的生活方式以预防感染和保护身体健康。 | Yes | No | No | No |
| 45 | Clinical Problems | Give advice for a patient who's HBsAb is positive | If a patient's HBsAb (hepatitis B surface antibody) test is positive, it indicates that the patient has been previously exposed to the hepatitis B virus or has received the hepatitis B vaccine. In either case, the patient is considered immune to hepatitis B. If the patient has no signs or symptoms of hepatitis B infection and their liver function tests are normal, then no further treatment may be necessary. However, if the patient has signs or symptoms of hepatitis B infection or their liver function tests are abnormal, then further evaluation and treatment may be needed. If the patient is diagnosed with chronic hepatitis B infection, treatment may be necessary to prevent liver damage and reduce the risk of developing liver cancer. Treatment options may include antiviral medications such as entecavir, tenofovir, or interferon, depending on the severity of the infection. It is important for the patient to continue to receive regular follow-up care and monitoring by a healthcare provider to ensure that their liver function tests remain normal and to detect any potential complications of chronic hepatitis B infection. Additionally, the patient should be counseled on ways to prevent the spread of hepatitis B to others, such as using condoms during sexual activity and avoiding sharing needles or other injection equipment. | If a patient's HBsAb (hepatitis B surface antibody) test is positive, it indicates that the patient has developed immunity to the hepatitis B virus. Here are some clinical treatment advice for such patients: Preventative measures: Patients who have developed immunity to hepatitis B should still take precautions to prevent the spread of the virus. They should avoid sharing personal items, such as razors and toothbrushes, and should use protection during sexual activity. Monitoring: Patients with a positive HBsAb test should be monitored regularly by their healthcare provider. The frequency of monitoring will depend on the patient's overall health and any other medical conditions they may have. Vaccination: Patients who have not yet developed immunity to hepatitis B should be vaccinated to prevent infection. Patients who have developed immunity should still receive routine booster shots to maintain their immunity. Treatment of acute infection: If a patient with a positive HBsAb test becomes infected with hepatitis B, they should be treated with antiviral medication to prevent the virus from causing liver damage. Treatment will depend on the severity of the infection and the patient's overall health. Treatment of chronic infection: If a patient has chronic hepatitis B, their healthcare provider will monitor their liver function and may recommend antiviral medication to prevent liver damage. In some cases, the patient may be referred to a specialist for further evaluation and treatment. It's important to note that treatment recommendations will vary depending on the patient's individual circumstances, and patients should always follow their healthcare provider's advice. | Yes | Yes |  | Yes |
| 45 | Clinical Problems | 乙型肝炎病毒表面抗体阳性怎么办？ | 乙型肝炎病毒表面抗体（HBsAb）阳性通常表示您的身体曾经感染过乙型肝炎病毒（HBV），或者曾经接种过乙型肝炎疫苗。如果您已经感染过HBV，您的身体已经产生了抗体以对抗该病毒。如果您已经接种过乙型肝炎疫苗，您的身体同样会产生抗体以保护您免受HBV感染。 如果您的HBsAb阳性，意味着您的身体已经产生了足够的抗体来保护您免受HBV感染。因此，您不需要采取任何特殊的行动。如果您担心自己的健康或者认为自己可能患有乙型肝炎，请咨询您的医生或医疗保健专业人士，进行更进一步的检查和治疗建议。 | 如果你的乙型肝炎病毒表面抗体（HBsAb）阳性，这意味着你的身体已经产生了对乙型肝炎病毒的免疫力。通常情况下，这意味着你已经接种过乙肝疫苗或者曾经感染过乙型肝炎病毒，但现在已经康复。 如果你之前没有接种过乙肝疫苗，但是HBsAb阳性，这表明你已经产生了对乙肝病毒的免疫力。如果你没有任何乙肝症状或体征，通常不需要进行进一步治疗或观察。 然而，如果你之前没有接种过乙肝疫苗，并且还有其他乙肝症状或体征，建议咨询医生进行进一步检查和治疗。 总之，如果你的HBsAb阳性，建议定期进行体检并接受医生的建议，以确保你的健康和安全。 | Yes | Yes |  | Yes |
| 46 | Clinical Problems | Give advice for a patient who's HBcAb is positive | HBcAb positive test result indicates that the patient has been exposed to the hepatitis B virus (HBV) at some point in their life. The presence of this antibody alone does not indicate current infection or active disease, but rather that the patient has been exposed to the virus at some point in the past. Clinical treatment advice for patients with a positive HBcAb test result may depend on the patient's overall health status, risk factors for HBV infection, and other factors that should be considered by a healthcare provider. However, some general recommendations are as follows: Evaluation of liver function: Patients with positive HBcAb should undergo liver function tests to assess the health of their liver. Vaccination: Patients who are HBcAb positive but have not been previously vaccinated against HBV should receive the HBV vaccine to protect them from future infections. Monitoring: Patients who are HBcAb positive but have no evidence of active HBV infection should be monitored periodically to check for signs of active infection or liver disease. Treatment: Patients with evidence of active HBV infection (i.e., HBsAg positive) should be evaluated for antiviral therapy. The decision to start antiviral therapy will depend on the patient's overall health, degree of liver damage, and other factors. Lifestyle changes: Patients with HBV infection should be advised to avoid alcohol and to maintain a healthy weight. It is important to note that the management of patients with HBcAb positive results should be individualized and may require additional testing and evaluation by a healthcare provider. | HBcAb positive refers to the presence of hepatitis B core antibody in the blood, which indicates past or ongoing hepatitis B virus infection. The management of patients with HBcAb positive depends on several factors, such as the patient's clinical history, laboratory tests, and presence of coexisting medical conditions. Here are some general clinical treatment advice for patients who are HBcAb positive: Monitor liver function: Patients who are HBcAb positive should have regular liver function tests to monitor for any signs of liver damage or disease progression. Consider antiviral therapy: Patients with chronic hepatitis B infection (HBsAg positive for more than 6 months) and elevated liver enzymes may benefit from antiviral therapy to suppress the virus and prevent liver damage. The decision to initiate treatment should be made by a specialist after considering the patient's individual circumstances. Avoid alcohol and certain medications: Patients with HBcAb positive should avoid alcohol and certain medications that can cause liver damage. Patients should always check with their doctor before taking any new medications. Practice safe sex: Hepatitis B can be transmitted through sexual contact, so patients should practice safe sex and use barrier protection (such as condoms) to reduce the risk of transmission. Vaccination: Patients who are not immune to hepatitis B virus (HBsAb negative) should receive the hepatitis B vaccine to prevent future infection. Regular follow-up: Patients with HBcAb positive should have regular follow-up with their healthcare provider to monitor their liver function and disease progression. It's important to note that the management of HBcAb positive patients can be complex and should be individualized based on the patient's specific circumstances. Therefore, patients should always consult with their healthcare provider for personalized treatment advice. | Yes | Yes |  | Yes |
| 46 | Clinical Problems | 乙型肝炎核心抗体阳性怎么办？ | 如果您的乙型肝炎核心抗体（anti-HBc）检测结果为阳性，这意味着您曾经感染过乙型肝炎病毒（HBV），但目前可能已经康复或是成为了携带者。 建议您进行以下措施： 建议您去医院进行进一步检查，包括HBV DNA、乙肝表面抗原（HBsAg）等指标的检测，以了解您的肝炎病毒感染情况和病情严重程度。 如果您是慢性乙型肝炎病毒携带者，则需要接受医生的治疗和监护，并遵循医生的建议进行规范治疗，以控制和预防疾病的恶化。 如果您还没有接种过乙型肝炎疫苗，建议您尽快接种乙型肝炎疫苗，以预防乙型肝炎病毒的感染。 建议您遵守卫生习惯，避免与血液和体液直接接触，避免高危行为，如注射毒品、性行为等。 请注意，以上建议仅供参考，具体诊治和治疗方案应根据医生的诊断和建议进行。 | 如果乙型肝炎核心抗体（anti-HBc）检测结果呈阳性，这通常意味着你曾经感染过乙型肝炎病毒（HBV），但可能已经恢复或者仍然是慢性感染。 如果你的乙型肝炎表面抗体（anti-HBs）也呈阳性，则说明你已经康复并且具有免疫力，无需特殊处理。如果你的表面抗体呈阴性，则说明你可能需要接种乙型肝炎疫苗以获得免疫保护。 如果你的肝功能检查结果异常，或者其他乙型肝炎相关检查结果异常，你应该咨询医生进行详细评估和治疗。另外，如果你的性伴侣或家庭成员感染了乙型肝炎病毒，你可能需要接受疫苗和/或其他治疗来预防感染。 | No | No |  | No |
| 47 | Clinical Problems | Give advice for a patient who's HBeAb is positive | HBeAb (hepatitis B e antibody) positivity indicates that a person has been exposed to hepatitis B virus (HBV) in the past and has developed an immune response to the e antigen of the virus. This may indicate a resolved infection, or it could be a result of vaccination against hepatitis B. If a patient's HBeAb test is positive, but their hepatitis B surface antigen (HBsAg) and HBV DNA tests are negative, then it suggests that the patient has been previously infected with HBV but has cleared the virus and is no longer infectious. In this case, no specific treatment is necessary, but the patient should be monitored periodically to ensure that the virus does not reactivate. However, if a patient is positive for both HBeAb and HBsAg, it suggests that they have chronic hepatitis B infection. In this case, the patient should be evaluated by a hepatologist or infectious disease specialist to determine the appropriate treatment regimen. The goal of treatment for chronic hepatitis B is to suppress viral replication and prevent progression of liver disease. The treatment options may include antiviral medications such as tenofovir or entecavir, which are effective at suppressing viral replication and reducing the risk of liver damage. These medications are usually prescribed for a prolonged period, and the patient will need to be monitored regularly with blood tests to assess their response to treatment and liver function. In addition to medication, lifestyle modifications such as avoiding alcohol and maintaining a healthy diet may also be recommended to help improve liver health. Vaccination against hepatitis A and B may also be recommended for patients with chronic hepatitis B to protect against co-infection with other types of viral hepatitis. It is important for patients with chronic hepatitis B to follow their treatment plan and attend regular follow-up appointments to monitor their condition and ensure that they are responding to treatment. | If a patient's HBeAb (Hepatitis B e-antibody) test result is positive, it means that they have been exposed to the Hepatitis B virus and have developed antibodies against it. A positive HBeAb result may indicate that the patient has resolved the infection or that they are in the inactive carrier state, which means that they have the virus but it is not actively replicating in their liver. In patients with a positive HBeAb result who are in the inactive carrier state, the goal of treatment is to prevent the development of liver damage or the progression of liver disease. The following clinical treatment advice may be considered: Regular Monitoring: Patients should be monitored regularly by a healthcare provider to assess the status of their liver disease and to detect any signs of disease progression. Antiviral Therapy: Antiviral therapy may be considered for patients with evidence of active liver disease, including elevated liver enzymes, evidence of liver fibrosis, or a high viral load. Antiviral medications can help to suppress viral replication and reduce the risk of disease progression. Vaccination: Patients who are HBeAb positive should be vaccinated against Hepatitis A and B to reduce the risk of co-infection with other types of viral hepatitis. Lifestyle Changes: Patients should be advised to make lifestyle changes to reduce the risk of liver damage, including avoiding alcohol, maintaining a healthy diet, and avoiding medications that can be toxic to the liver. Regular Follow-up: Patients should have regular follow-up appointments with their healthcare provider to monitor the status of their liver disease and to adjust treatment as needed. It is important to note that the management of patients with a positive HBeAb result may vary depending on individual patient factors and the recommendations of the healthcare provider. Therefore, patients should discuss their specific treatment plan with their healthcare provider. | No | No |  | No |
| 47 | Clinical Problems | 乙型肝炎e抗体阳性怎么办？ | 乙型肝炎e抗体（Anti-HBe）阳性意味着您的体内曾经感染过乙型肝炎病毒（HBV），但病毒已经不再活跃，您的肝脏已经康复或者正在康复过程中。如果您的HBV核心抗体（Anti-HBc）也呈阳性，则说明您曾经患过乙型肝炎，但现在已经康复。 如果您的乙型肝炎病毒表面抗原（HBsAg）和乙型肝炎核心抗体（Anti-HBc）也是阳性，则可能表示您正在患有慢性乙型肝炎，需要进行进一步的检查和治疗。 建议您咨询专业医生，根据您的具体情况进行治疗和管理。如果您没有接种过乙型肝炎疫苗，可以考虑接种来预防乙型肝炎的感染。此外，您还应该注意保持良好的卫生习惯，避免接触乙型肝炎病毒，以减少感染的风险。 | 乙型肝炎e抗体（Anti-HBe）阳性表示体内已经存在对乙型肝炎病毒e抗原（HBeAg）的免疫反应，通常表明病毒复制已经得到控制或进入了慢性感染状态。如果你还没有确诊为乙型肝炎，请进行进一步检查以确认是否患有该疾病。 如果已经确诊为乙型肝炎，并且Anti-HBe阳性，通常意味着体内的病毒量已经下降并停止复制。但是，仍然需要密切关注肝功能和病毒感染情况，并定期进行医学检查和评估。如果你有任何症状或疑虑，请立即咨询医生，并按照医生的建议进行治疗和管理。 | Yes | Yes |  | Yes |
| 48 | Clinical Problems | How to understand HBV-DNA quetification results | HBV-DNA quantification results are used to measure the amount of hepatitis B virus (HBV) DNA in a person's blood. The results can help to diagnose and monitor HBV infection and guide treatment decisions. The results of an HBV-DNA test are usually reported as a number of international units per milliliter (IU/mL) of blood. The higher the number, the more active the HBV infection is in the body. In general, the following guidelines can be used to interpret HBV-DNA quantification results: <20 IU/mL: Undetectable HBV DNA, which is a good sign indicating a successful treatment or a resolved infection. 20-2,000 IU/mL: Low viral load, may indicate early or inactive infection or a successful treatment. 2,000-20,000 IU/mL: Moderate viral load, usually indicates active infection and treatment may be necessary. \|”20,000 IU/mL: High viral load, indicating an active and potentially aggressive infection, which often requires treatment.” It is important to note that the interpretation of HBV-DNA results should be done in conjunction with other clinical and laboratory findings, such as liver function tests and physical examinations, and under the guidance of a healthcare provider experienced in the management of HBV infection. | HBV-DNA quantification results are used to measure the amount of Hepatitis B virus (HBV) in a person's blood. The results are reported as a number of copies of HBV-DNA per milliliter (ml) of blood. Generally, the higher the number of HBV-DNA copies, the more active the infection. However, the interpretation of HBV-DNA quantification results can vary depending on the context and the individual patient's situation. Here are some general guidelines to help understand HBV-DNA quantification results: Low viral load: <2000 IU/mL or <10^4 copies/mL. This suggests a low level of HBV activity, and may indicate that the patient is in the inactive carrier state. Moderate viral load: 2000-20,000 IU/mL or 10^4-10^5 copies/mL. This suggests some level of HBV activity, and may indicate the need for treatment or monitoring. High viral load: >20,000 IU/mL or >10^5 copies/mL. This suggests a high level of HBV activity, and may indicate the need for immediate treatment. It's important to keep in mind that HBV-DNA quantification results should be interpreted in conjunction with other tests and clinical information, such as liver function tests and the patient's medical history. Treatment decisions should be made in consultation with a healthcare provider who specializes in treating viral hepatitis. | Yes | No | Yes | Yes |
| 48 | Clinical Problems | HBV-DNA定量结果怎么看？ | HBV-DNA定量结果是用来测量乙型肝炎病毒（HBV）的病毒载量，通常以国际单位（IU）/毫升（mL）或拷贝数/毫升（mL）来表示。 当进行HBV-DNA定量检测时，结果通常会显示一个数字，例如：“1.0E+05 IU/mL”或“1.0E+08拷贝/mL”。这些数字中的“E”表示指数，后面的数字表示病毒载量。例如，“1.0E+05 IU/mL”表示病毒载量为10万IU/mL，而“1.0E+08拷贝/mL”表示病毒载量为1亿拷贝/mL。 通常情况下，病毒载量越高，患者的肝脏损伤就越严重。因此，医生会根据患者的HBV-DNA定量结果来确定治疗方案，并监测治疗的有效性。 需要注意的是，不同的实验室可能使用不同的方法进行HBV-DNA定量检测，因此，不同实验室得出的结果可能会有所不同。因此，应该仔细阅读实验室提供的结果说明，并在需要时咨询医生以更好地理解您的检测结果。 | HBV-DNA定量结果通常以国际单位（IU）/毫升（mL）或复制数/mL（cp/mL）的形式报告。这些结果显示了血液中乙型肝炎病毒（HBV）的数量。 以下是一些常见的HBV-DNA定量结果及其解释： 阴性：如果定量结果为“未检测到”或“阴性”，则表示在样本中未检测到HBV-DNA。 低病毒载量：定量结果在500-2000 IU/mL或10^1-10^3 cp/mL之间，通常表明患者患有慢性乙型肝炎（CHB），但病毒载量较低。 中等病毒载量：定量结果在2000-20000 IU/mL或10^3-10^5 cp/mL之间，表明患者的HBV病毒载量较高，但仍处于中等水平。 高病毒载量：定量结果在20000 IU/mL或10^5 cp/mL以上，表明患者的HBV病毒载量非常高。 请注意，HBV-DNA定量结果只是诊断和治疗的一部分，必须结合其他检查结果进行综合分析。如果您有任何疑问或需要更多信息，请咨询您的医生或医疗保健提供者。 | Yes | No | No | No |
| 49 | Clinical Problems | Give clinical treatment advice for ones who are exposed to hepatitis B virus | 1.If someone has been exposed to the hepatitis B virus, the first step is to get tested to determine if they have been infected. If the test is positive, treatment will depend on the severity of the infection. 2.Here are some general guidelines for the clinical treatment of hepatitis B virus: 3.Acute Hepatitis B: Acute hepatitis B is usually self-limited, meaning that it will resolve on its own without specific treatment. However, if the infection is severe or if the person is at risk for complications, they may require hospitalization and supportive care. 4.Chronic Hepatitis B: Chronic hepatitis B is a long-term infection that can lead to liver damage, liver failure, and liver cancer. Treatment for chronic hepatitis B may involve antiviral medications, such as entecavir, tenofovir, or pegylated interferon. These medications can help reduce the amount of virus in the blood and prevent liver damage. 5.Liver Transplant: If someone with chronic hepatitis B develops severe liver damage or liver failure, they may require a liver transplant. 6.Prevention: The best way to prevent hepatitis B is through vaccination. The hepatitis B vaccine is safe and effective, and it is recommended for all infants, children, and adults who are at risk of exposure to the virus. If you have been exposed to hepatitis B virus, it is important to speak with a healthcare provider as soon as possible to determine if you have been infected and to discuss treatment options. | If someone has been exposed to hepatitis B virus (HBV), they should seek medical advice immediately. The following steps are recommended for clinical treatment: Evaluation of HBV status: The first step is to determine the patient's HBV status. This is done by testing for hepatitis B surface antigen (HBsAg), hepatitis B core antibody (anti-HBc), and hepatitis B surface antibody (anti-HBs). The results of these tests will determine the patient's immune status and whether they have an acute or chronic infection. Treatment for acute HBV infection: Acute HBV infection usually resolves on its own without specific treatment. However, symptomatic treatment can be given for nausea, vomiting, and abdominal pain. Rest and adequate hydration are also recommended. Treatment for chronic HBV infection: If the patient is diagnosed with chronic HBV infection, antiviral therapy may be needed to suppress the virus and reduce the risk of liver damage. Antiviral medications such as tenofovir and entecavir are commonly used. Vaccination: If the patient is not immune to HBV, vaccination should be offered. The hepatitis B vaccine is highly effective and can prevent future infections. Follow-up: Patients with acute HBV infection should be monitored until the infection resolves. Patients with chronic HBV infection should be monitored regularly for liver function and virus activity. It's important to note that prevention is the best way to avoid hepatitis B infection. This includes practicing safe sex, avoiding needle-sharing, and getting vaccinated. | No | Yes | No | No |
| 49 | Clinical Problems | 乙肝病毒暴露的临床处理意见 | 如果您认为自己已经暴露于乙肝病毒，您应该尽快向医生寻求帮助。以下是可能的行动计划： 1.立即寻求医疗帮助：如果您认为自己已经暴露于乙肝病毒，您应该尽快联系您的医生或前往最近的急诊室，以便得到及时的检查和治疗。 2.检查乙肝病毒感染：医生可能会进行一系列测试，以确定您是否感染了乙肝病毒。这些测试可能包括血液测试和体格检查。 3.接种乙肝疫苗：如果您未接种过乙肝疫苗，医生可能会建议您接种疫苗，以帮助保护您免受感染。 4.接受治疗：如果您确诊感染了乙肝病毒，您的医生可能会建议您接受药物治疗或其他治疗方式。治疗可帮助控制病毒，减少肝脏损伤和防止疾病进展。 5.采取预防措施：乙肝病毒可以通过接触感染者的体液（如血液、唾液和性液体）传播。因此，采取预防措施，如使用安全套和避免分享针头或其他注射器是很重要的。 | 如果您认为自己可能暴露于乙肝病毒，请立即向您的医疗保健提供者咨询建议。以下是一些可能的步骤： 1.检查：医生会根据您的具体情况进行评估，可能会进行血液检查来确定您是否感染了乙肝病毒。 2.疫苗接种：如果您没有接种过乙肝疫苗，医生可能会建议您立即接种。乙肝疫苗是预防乙肝感染的最佳方法。 3.免疫球蛋白注射：如果您已经暴露于乙肝病毒，但尚未感染，医生可能会建议您注射免疫球蛋白。这是一种能够帮助您的免疫系统消灭病毒的药物。 4.治疗：如果您已经感染了乙肝病毒，医生可能会根据您的具体情况开具治疗方案。治疗的目标是控制病情，防止病毒进一步破坏肝脏，并降低传染他人的风险。 请注意，如果您认为自己已经暴露于乙肝病毒，请务必及时咨询您的医疗保健提供者。他们将为您提供专业的建议和治疗。 | Yes | Yes |  | Yes |
| 50 | Clinical Problems | Can Hepatitis B be cured？ | Hepatitis B is a viral infection that affects the liver. While there is no cure for hepatitis B, it can be managed and its progression can be slowed down with proper medical treatment. The primary goal of treatment for chronic hepatitis B is to reduce the risk of complications such as liver cirrhosis and liver cancer. This is typically done through antiviral medications that are designed to suppress the virus and slow down its replication. In some cases, the body's immune system may be able to clear the virus on its own, but this is relatively rare. In general, once a person has been infected with hepatitis B, they will carry the virus for life, even if they do not experience symptoms. It's important to note that the hepatitis B vaccine is highly effective at preventing infection, and is recommended for all individuals, particularly those who are at high risk of contracting the virus. | Currently, there is no cure for hepatitis B, but it can be effectively managed with medical treatment. Hepatitis B is a viral infection that affects the liver and can cause liver damage, cirrhosis, and liver cancer. There are antiviral medications available that can help control the virus and reduce the risk of liver damage. These medications work by slowing or stopping the replication of the virus in the liver. However, even with treatment, the virus may not be completely eliminated from the body, and a person with hepatitis B may remain a carrier of the virus. It is important to get diagnosed early and to receive ongoing medical care to monitor the progression of the disease and to manage any complications. Additionally, vaccination is recommended for those who are at risk of contracting the virus, as prevention is the best way to avoid the long-term consequences of hepatitis B. | Yes | Yes |  | Yes |
| 50 | Clinical Problems | 乙肝可以治愈吗 | 是的，乙肝可以治愈，但是这取决于许多因素，包括感染乙肝病毒的时间、病毒的血清学标志物、肝脏损伤程度和是否存在肝硬化或肝癌等并发症。 对于许多乙肝患者来说，特别是那些处于慢性感染早期的人，通过药物治疗可以控制病毒复制并使肝脏恢复正常功能。对于其他患者，可能需要更长时间的治疗以达到完全恢复的目标。 对于那些患有肝硬化或肝癌的人，治疗的目标是控制疾病的进展并预防并发症的发生。在这些情况下，治愈可能是不可能的，但可以通过治疗延长生命并提高生活质量。 总的来说，治疗乙肝需要一个综合的方法，并且应根据每个人的独特情况进行个性化治疗。如果您认为自己可能感染了乙肝病毒，请尽快咨询医生以获取诊断和治疗建议。 | 是的，乙肝是一种可以治愈的疾病，但治愈的难度和可能性因患者的情况而异。 对于急性乙肝，多数患者能够在数周内自行恢复，这种情况下治愈率很高。而对于慢性乙肝，治愈的难度会增加，但仍有一定的可能性。目前，乙肝的主要治疗方法是使用抗病毒药物来抑制病毒复制和减轻症状。在长期治疗的过程中，一些患者可能会出现乙肝病毒表面抗原（HBsAg）阴转的情况，即病毒被完全清除，患者已经治愈。 需要注意的是，乙肝治愈不仅仅是指病毒被清除，还包括患者肝功能恢复正常、肝脏组织病变逐渐消失等多个方面。同时，对于已经成为慢性乙肝患者的人来说，尽早发现、积极治疗是非常重要的，可以减少肝功能受损和肝癌的风险。 | Yes | Yes |  | Yes |
| 51 | Clinical Problems | If I have been infected with the hepatitis B virus in the past, can I get it again | If you have been previously infected with the hepatitis B virus and have recovered, you will have developed immunity to the virus. This means that you cannot get infected with the same strain of the hepatitis B virus again. However, there are several different strains or genotypes of the hepatitis B virus, and if you are exposed to a different strain, you may be at risk of getting infected again. Additionally, if your immune system is weakened, it may be less able to fight off the virus and you may be more susceptible to becoming infected again. It's also important to note that having a past hepatitis B infection does not provide protection against other hepatitis viruses, such as hepatitis A or hepatitis C. It's possible to become infected with these viruses even if you have previously had hepatitis B. If you have concerns about your risk of hepatitis B or other hepatitis viruses, it's important to speak with your healthcare provider about testing and vaccination options. | If you have been infected with hepatitis B virus in the past and have recovered, you should be immune to the virus and therefore cannot get infected again. This is because your body will have developed antibodies to fight the virus during the initial infection, and these antibodies will remain in your blood even after you have recovered. However, it is possible to get infected with a different strain or subtype of the hepatitis B virus. There are several subtypes of the virus, and if you were infected with one subtype, you may still be susceptible to infection with a different subtype. It is also possible for people with chronic hepatitis B infection to experience a reactivation of the virus, even if they had previously cleared the virus. This can happen if the immune system is weakened, for example, due to certain medications or medical conditions. Therefore, if you have been infected with hepatitis B in the past, it is important to get tested regularly to monitor your liver health and make sure that the virus has not reactivated or caused any long-term damage. | Yes | Yes |  | Yes |
| 51 | Clinical Problems | 我以前感染过乙肝，还会再感染吗 | 如果您曾经感染过乙肝病毒并且康复，您通常会获得持久性免疫，这意味着您不太可能再次感染乙肝病毒。这是因为您的免疫系统已经生成了对病毒的抗体，这些抗体可以保护您免受再次感染。 然而，如果您的免疫系统功能较差或者您接触了大量乙肝病毒，您仍然可能会再次感染乙肝病毒。此外，虽然罕见，但乙肝病毒的变异也可能导致您感染不同的乙肝病毒株。 因此，即使您曾经感染过乙肝病毒，也应该采取预防措施，例如接种乙肝疫苗、避免与感染乙肝病毒的人进行性接触或共用注射器等。 | 如果你已经感染过乙肝病毒并恢复了，那么你会获得一种被称为乙肝表面抗原（HBsAg）的免疫物质。这种免疫物质可以保护你免受再次感染乙肝病毒的侵害。因此，一般情况下，如果你已经感染过乙肝病毒并恢复了，你就不会再次感染乙肝。 然而，需要注意的是，如果你的免疫系统出现问题，比如因为某些疾病或药物而导致免疫力下降，那么你可能会再次感染乙肝病毒。此外，乙肝病毒有多个亚型和突变体，如果你之前感染的是一种亚型或突变体，而你又接触到了其他亚型或突变体，那么你也有可能再次感染乙肝病毒。 因此，即使你曾经感染过乙肝病毒并恢复了，你仍然需要保持良好的个人卫生习惯，并按时接种乙肝疫苗，以避免再次感染。同时，如果你有免疫系统缺陷或其他健康问题，应该在医生的建议下进行相应的治疗和预防措施。 | Yes | Yes |  | Yes |
| 52 | Clinical Problems | Can I donate blood/organs if I have hepatitis B? | In general, people with hepatitis B may not be eligible to donate blood or organs. This is because hepatitis B is a virus that can be transmitted through blood and other bodily fluids, and there is a risk of transmitting the virus to the recipient of the blood or organ. However, the eligibility to donate blood or organs may vary depending on several factors, such as the stage of the disease, the viral load, and the presence of other medical conditions. For blood donation, most blood donation centers will not accept donations from people with hepatitis B. However, in some countries, individuals with chronic hepatitis B who are being treated with antiviral medication and have a low viral load may be eligible to donate blood. It's important to check with your local blood donation center for their specific policies and guidelines. As for organ donation, people with hepatitis B may be eligible to donate organs in certain cases. For example, if the organ recipient also has hepatitis B, the donor's organ may be a good match. In other cases, the donor's liver or other organs may be used for research purposes. It's important to discuss your eligibility to donate blood or organs with your healthcare provider and local blood and organ donation centers. They can provide more specific information and guidance based on your individual situation. | The eligibility to donate blood and organs if you have hepatitis B depends on various factors, such as the type of donation, the stage of your hepatitis B infection, and the recipient's health condition. For blood donation, if you have chronic hepatitis B, you cannot donate blood because the virus can be transmitted through blood transfusion. However, if you have resolved hepatitis B infection (meaning you have cleared the virus from your body), you may be eligible to donate blood after a certain period of time has passed since your recovery. The specific eligibility criteria may vary by country and blood bank, so it's best to check with your local blood donation center for more information. Regarding organ donation, the eligibility of individuals with hepatitis B to donate organs depends on the type of organ and the recipient's health condition. If you have chronic hepatitis B, you may not be able to donate organs such as the liver, but you may be able to donate other organs, such as the kidneys, after evaluation by a medical professional. If you have resolved hepatitis B infection, you may be eligible to donate organs in certain circumstances. It's important to note that eligibility to donate blood and organs is determined on a case-by-case basis and may depend on various factors, including your medical history and the recipient's health condition. It's best to consult with your healthcare provider or a medical professional at a blood or organ donation center for specific guidance regarding your eligibility to donate. | Yes | Yes |  | Yes |
| 52 | Clinical Problems | 得了乙肝可以献血/捐献器官吗？ | 不建议乙肝患者献血或捐献器官。 乙肝是一种病毒性肝炎，由乙型肝炎病毒感染引起。乙肝病毒可以通过血液、体液、母婴传播等途径传染，因此乙肝患者的血液和器官中也可能存在病毒。 献血和捐献器官都是为了救助他人，但是乙肝病毒会对受血者和受器官者产生危害，因此乙肝患者不应该献血或捐献器官。 如果您怀疑自己可能感染了乙肝病毒，请尽早咨询医生，并遵循医生的建议进行治疗和预防传播。 | 不可以。乙肝病毒可以通过血液和体液传播，所以如果您患有乙肝病毒感染，您不能捐献血液或器官。 此外，根据世界卫生组织的建议，只有符合以下条件的人才能进行器官捐献： 1. 没有传染性疾病的人； 2. 死亡捐献器官的捐献者； 3. 捐献者及其家属同意器官捐献。 因此，如果您患有乙肝病毒感染，您不能进行器官捐献。 | No | No |  | No |
| 53 | Clinical Problems | Is it harmful to have an extra dose of hepatitis B vaccine or to repeat the entire hepatitis B vaccine series? | There is no evidence to suggest that receiving an extra dose of the hepatitis B vaccine or repeating the entire hepatitis B vaccine series would be harmful. In fact, in certain situations, such as a potential exposure to the virus or if there is uncertainty about whether someone has completed the vaccine series, it may be recommended to receive an extra dose or restart the series. According to the Centers for Disease Control and Prevention (CDC), there is no maximum number of doses for the hepatitis B vaccine, and there is no need to restart the vaccine series if an additional dose is given. However, it is important to follow the recommended dosing schedule for the vaccine, which typically involves receiving three or four doses over a period of several months. It is always a good idea to consult with a healthcare provider if you have questions or concerns about receiving an extra dose or repeating the hepatitis B vaccine series. | There is no evidence to suggest that receiving an extra dose of the hepatitis B vaccine or repeating the entire hepatitis B vaccine series is harmful. In fact, receiving extra doses or repeating the series is sometimes recommended in certain situations, such as for people who have a weakened immune system or who did not respond adequately to the initial vaccine series. The hepatitis B vaccine is a safe and effective vaccine that is recommended for all individuals, particularly those who are at higher risk of infection, such as healthcare workers, people with multiple sexual partners, people who inject drugs, and people with chronic liver disease. The vaccine is typically administered in a series of three doses, with the second dose given 1 month after the first and the third dose given 6 months after the first. If you are unsure about whether you need an extra dose of the hepatitis B vaccine or to repeat the entire vaccine series, you should consult with your healthcare provider. They can help you determine if you need additional doses based on your individual risk factors and medical history. | Yes | Yes |  | Yes |
| 53 | Clinical Problems | 乙肝疫苗多打几针有害吗 | 乙肝疫苗是一种安全有效的疫苗，接种多打几针并不会对身体造成明显的危害。在正常情况下，乙肝疫苗的接种方案是在出生后立即接种第一针，接着在一个月和六个月后各接种一针。这种接种方案可以有效预防乙肝病毒的感染。 有时候，如果在第一次接种后身体没有产生足够的免疫力，或者在接种过程中发生了错误，例如疫苗未存放妥当，可能需要打一些额外的疫苗。通常，这些额外的疫苗不会对身体造成任何危害。 但是，如果打乙肝疫苗后出现了不良反应，例如过敏反应或发热等，应及时向医生咨询并接受医生的治疗建议。如果出现严重不良反应，应立即就医。 | 一般来说，乙肝疫苗的多次接种不会对健康造成任何明显的负面影响。乙肝疫苗是一种安全有效的疫苗，通过接种可以激发人体免疫系统产生抗体来预防乙肝病毒的感染。 根据世界卫生组织的建议，乙肝疫苗的接种通常分为三剂，即在出生后的24小时内接种第一剂，然后在一个月后接种第二剂，最后在第六个月接种第三剂。如果需要加强免疫力，可以接种额外的剂量，但不建议超过4剂。 一些人可能会在接种疫苗后出现轻微的不适，如局部疼痛、红肿、发热等，这些反应通常在几天内消失。如果您有任何疑虑或担忧，建议咨询您的医生或接种医生。 | Yes | Yes |  | Yes |
| 54 | Clinical Problems | What should I do if I started but did not complete the full hepatitis B vaccine series | If you have started but not completed the full hepatitis B vaccine series, it's recommended that you complete the series as soon as possible to ensure you are fully protected against the hepatitis B virus. The standard hepatitis B vaccine series consists of three doses, typically given over a period of six months. However, the specific schedule and number of doses may vary based on individual circumstances and the type of vaccine used. If you missed a dose or are unsure about your vaccination status, it's best to speak with a healthcare provider who can review your medical history and recommend the appropriate course of action. Depending on your situation, they may recommend restarting the vaccine series or receiving a booster dose. It's important to note that completing the full hepatitis B vaccine series is the most effective way to prevent hepatitis B infection, which can lead to serious liver damage and other health complications if left untreated. | If you started but did not complete the full hepatitis B vaccine series, it is recommended that you complete the series as soon as possible. The hepatitis B vaccine is a series of three doses, and it is important to receive all three doses to ensure maximum protection against the virus. You can talk to your healthcare provider about getting the remaining doses of the vaccine. They may recommend restarting the series, or they may be able to give you the remaining doses to complete the series. It is important to note that even if you have only received one or two doses of the hepatitis B vaccine, you may still have some protection against the virus. However, without completing the full series, your protection may not be as strong as it could be. In addition to getting the hepatitis B vaccine, it is important to take other measures to prevent the spread of the virus. This includes practicing safe sex, not sharing needles or other injection equipment, and avoiding contact with blood or bodily fluids of others. | Yes | Yes |  | Yes |
| 54 | Clinical Problems | 乙肝疫苗没有全程接种完该怎么办 | 如果您没有完成乙肝疫苗的全程接种，建议尽快前往医疗机构进行补种。补种可以提高您免疫力的水平，降低患病的风险。 根据乙肝疫苗的接种方案，一般需要接种3针，每针之间的时间间隔为1个月。如果您错过了某次接种，可以在下次接种时继续按照接种方案接种，不需要从头开始。 如果您不确定自己是否完成了全程接种，可以向医疗机构或医生咨询，他们可以帮助您查看您的接种记录，并给出相应的建议。 | 如果您没有接种完乙肝疫苗，建议尽快前往接种医疗机构进行接种。如果您已经错过了预定的接种时间，您可以联系接种机构重新安排接种时间。在重新接种前，您无需重头开始接种，接种医生会根据您已经接种的疫苗剂次，确定下一次接种时间和剂次。 根据中国疾病预防控制中心的推荐，乙肝疫苗一般需要接种3剂次，第一剂和第二剂之间间隔1个月，第二剂和第三剂之间间隔6个月。如果您错过了其中一剂疫苗的接种，建议在接种周期内尽快补种，以确保接种效果和预防效果。如果您无法确定是否完成了乙肝疫苗接种，您可以咨询医生或前往接种机构查询接种记录。 | Yes | Yes |  | Yes |
| 55 | Clinical Problems | Is the hepatitis B vaccine recommended before international travel? | Yes, the hepatitis B vaccine is recommended before international travel, especially if you are traveling to a region where hepatitis B is prevalent. Hepatitis B is a serious viral infection that affects the liver, and it can be transmitted through contaminated blood or body fluids, such as through unprotected sex, sharing needles, or from mother to child during childbirth. If you are traveling to a region where hepatitis B is prevalent, the Centers for Disease Control and Prevention (CDC) recommends that you receive the hepatitis B vaccine before you leave. In addition, it is important to take other precautions to prevent the transmission of hepatitis B, such as practicing safe sex, avoiding sharing needles or other injection equipment, and avoiding exposure to contaminated blood or body fluids. It is important to note that the hepatitis B vaccine is given as a series of three injections over a period of several months, so it is important to plan ahead and get vaccinated well before your travel dates. If you are unsure whether you need the hepatitis B vaccine for your travel destination, you should consult with a healthcare provider or travel medicine specialist. | Yes, the hepatitis B vaccine is recommended before international travel to areas with high rates of hepatitis B infection. Hepatitis B is a viral infection that can cause liver disease, and it is spread through contact with infected blood or other body fluids. The vaccine is highly effective in preventing hepatitis B infection. The Centers for Disease Control and Prevention (CDC) recommends that all travelers get vaccinated against hepatitis B if they are traveling to areas where the infection is common, such as Africa, Asia, the Pacific Islands, the Middle East, Eastern Europe, and South America. The vaccine is also recommended for people who may be at risk of exposure to hepatitis B, such as healthcare workers, people with multiple sexual partners, and people who inject drugs. It's important to note that the hepatitis B vaccine is typically given as a series of three doses, so travelers should plan ahead and get vaccinated well before their trip. It's also a good idea to consult with a healthcare provider or travel medicine specialist to determine which vaccines are recommended for your specific travel plans. | Yes | Yes |  | Yes |
| 55 | Clinical Problems | 出国旅行前需要打乙肝疫苗吗 | 要不要打乙肝疫苗需要根据你所前往的国家和地区以及你的个人情况来确定。一些国家和地区的乙肝流行率较高，因此在前往这些地方旅行前打乙肝疫苗是有必要的。此外，如果你有以下情况之一，也建议接种乙肝疫苗： 经常需要输血或血制品 从事医疗卫生工作 有多个性伴侣或进行不安全性行为 使用注射药物或共用针头 因此，如果你要出国旅行，请先咨询你的医生或前往旅行医学中心，了解你所前往的国家和地区的乙肝流行情况，并根据自己的情况决定是否需要接种乙肝疫苗。 | 这取决于你要前往的国家和地区以及你的个人状况。有些国家对入境者的乙肝疫苗接种有要求，例如前往非洲、亚洲和南美洲的某些国家可能需要接种乙肝疫苗。此外，如果你计划在旅行期间进行某些活动，例如接触血液或体液，那么接种乙肝疫苗可能是一个好主意。 最好咨询你所在国家的医疗专业人士或旅行医生，了解你的旅行计划和健康状况是否需要接种乙肝疫苗。他们可以根据你的具体情况，提供更具体的建议和指导。 | No | Yes | No | No |
| 56 | Clinical Problems | What is an hepatitis B booster dose, and do I need one? | A hepatitis B booster dose is an additional dose of the hepatitis B vaccine that is given to people who have already received the full series of the vaccine. The hepatitis B vaccine is usually given as a series of three or four doses over a period of several months, depending on the brand of vaccine and the person's age and health status. A booster dose is typically recommended for certain groups of people who may be at increased risk of contracting hepatitis B, or who may not have developed adequate immunity after the initial vaccine series. This includes healthcare workers, people with chronic liver disease or HIV, people traveling to areas where hepatitis B is common, and people who have close household contact with someone who has chronic hepatitis B infection. If you are unsure whether you need a hepatitis B booster dose, it is best to consult with your healthcare provider. Your provider can assess your individual risk factors and vaccination history, and recommend whether a booster dose is needed. | A hepatitis B booster dose is an additional vaccine dose that is given after the initial series of hepatitis B vaccine doses to help maintain long-term protection against the hepatitis B virus. If you received the hepatitis B vaccine series as a child or adolescent, then you are likely to have long-lasting immunity and may not need a booster dose. However, if you are an adult and have received the hepatitis B vaccine series more than 5 years ago, your immunity may have decreased over time and a booster dose may be recommended. People who may benefit from a hepatitis B booster dose include healthcare workers, people who travel to areas with a high prevalence of hepatitis B, people with chronic liver disease, people with HIV infection, and people who have close contact with someone who has hepatitis B. It's important to talk to your healthcare provider about whether a hepatitis B booster dose is recommended for you based on your individual circumstances and medical history. | Yes | Yes |  | Yes |
| 56 | Clinical Problems | 乙肝疫苗加强针是什么，我需要打吗 | 乙肝疫苗加强针是一种用于加强人体对乙肝病毒免疫力的疫苗。一般来说，接种完初步的乙肝疫苗后，医生会建议在一定时间后进行加强针的接种，以增强对乙肝病毒的免疫力。加强针的接种次数和时间间隔会根据不同的情况而有所不同，具体应该咨询医生。 在一些地区，乙肝病毒较为常见，接种乙肝疫苗是保护自身健康的重要措施。如果你没有接种过乙肝疫苗，或者没有接种过加强针，那么建议咨询医生并考虑接种疫苗，以保护自己免受乙肝病毒的感染。 | 乙肝疫苗加强针是指在完成基础免疫接种（通常是在出生后的24小时内和后续几个月内完成的）之后，再次接种乙肝疫苗以增强身体对乙肝病毒的免疫力。加强针的接种时间通常在基础免疫接种完成后的6个月到1年之间。 对于需要打乙肝疫苗加强针的人群，通常包括以下几类人： 1.慢性乙肝病毒感染者：慢性乙肝病毒感染者的免疫系统可能无法有效清除病毒，因此需要加强针来提高免疫力。 2.具有乙肝感染风险的人群：例如医务工作者、卫生救援人员、实验室工作人员、有性接触风险的人群等。 3.具有乙肝疫苗接种失败风险的人群：例如免疫力低下、肝功能异常、重症疾病患者等。 如果您属于以上人群中的任何一类，建议您接种乙肝疫苗加强针以增强免疫力。如果您不确定是否需要接种，请咨询医生或前往当地的疾病预防控制中心咨询。 | No | No |  | No |
| 57 | Clinical Problems | Can hepatitis B vaccine be given during pregnancy or breast feeding? | The hepatitis B vaccine is generally considered safe for pregnant and breastfeeding women. However, the decision to administer the vaccine during pregnancy or breastfeeding should be made in consultation with a healthcare provider, taking into account the individual's medical history and risk factors. According to the Centers for Disease Control and Prevention (CDC), pregnancy is not a contraindication for hepatitis B vaccination. The CDC recommends that pregnant women who are at risk of acquiring hepatitis B infection, such as those with multiple sexual partners or who use intravenous drugs, receive the vaccine. Breastfeeding is also not a contraindication for hepatitis B vaccination. The vaccine is not known to be harmful to breastfeeding infants, and the antibodies produced by the mother in response to the vaccine can be passed to the infant through breast milk, providing additional protection against hepatitis B. As with any medication, it is important to consult with a healthcare provider before receiving the hepatitis B vaccine during pregnancy or breastfeeding. | The hepatitis B vaccine is generally considered safe and is recommended for pregnant women who are at risk of contracting hepatitis B. The vaccine can be given during pregnancy if the woman is at high risk of infection, such as healthcare workers, and the benefits outweigh the potential risks. However, as with any medical intervention during pregnancy, it is important to discuss the risks and benefits with a healthcare provider before getting the vaccine. It is also important to note that the hepatitis B vaccine is not recommended for routine use during pregnancy. Regarding breastfeeding, the hepatitis B vaccine is also considered safe for breastfeeding mothers and their infants. Breastfeeding is actually recommended for infants who receive the hepatitis B vaccine to provide additional protection against infection. Again, it is important to discuss any concerns with a healthcare provider before receiving the vaccine during pregnancy or while breastfeeding. | Yes | Yes |  | Yes |
| 57 | Clinical Problems | 妊娠或哺乳期可以打乙肝疫苗吗 | 根据世界卫生组织（WHO）的建议，妊娠期间可以接种乙肝疫苗，因为该疫苗在怀孕期间是安全的。 乙肝疫苗是一种疫苗，用于预防乙肝病毒感染。如果你正在怀孕或哺乳期间，你的医生可能会建议你接种乙肝疫苗，以帮助保护你和你的婴儿免受乙肝病毒感染。 然而，在接种乙肝疫苗之前，请务必咨询医生的意见，以确保这是安全和适当的做法。医生可能会考虑你的健康状况和任何其他特定风险因素，以决定是否接种疫苗。 | 妊娠或哺乳期的女性在一些情况下可以打乙肝疫苗，但需要根据具体情况来确定。 根据疫苗说明书，乙肝疫苗在妊娠期间应该仅在必要情况下使用。如果怀孕期间已经接种了部分乙肝疫苗，通常可以等待生产后再完成接种。如果怀孕期间未完成接种，建议在生产后的两个月内完成疫苗接种。 对于哺乳期妇女，目前没有证据表明乙肝疫苗对母乳产生不良影响。因此，如果有必要，在哺乳期间也可以进行乙肝疫苗接种。 然而，对于任何特定的个体，是否需要在妊娠或哺乳期接种乙肝疫苗，应该在医生的指导下进行决定。 | Yes | Yes |  | Yes |
| 58 | Clinical Problems | What should I do if I received the HEPLISAV-B vaccine while pregnant? | If you have received the HEPLISAV-B vaccine while pregnant, it is important that you inform your healthcare provider and discuss any concerns or questions you may have. HEPLISAV-B is a vaccine that helps to protect against hepatitis B virus (HBV) infection. It is currently recommended for use in adults aged 18 years and older. There is limited information available on the safety of HEPLISAV-B in pregnant women. Therefore, the Centers for Disease Control and Prevention (CDC) currently recommend that pregnant women should not receive the HEPLISAV-B vaccine unless they are at risk of HBV infection and have been evaluated by their healthcare provider. If you have received the HEPLISAV-B vaccine while pregnant, your healthcare provider may recommend additional monitoring and follow-up care to ensure the health and well-being of you and your baby. It is important to remember that each individual case is unique, and the best course of action will depend on your specific situation. Therefore, it is crucial to speak with your healthcare provider about any concerns or questions you may have about receiving the HEPLISAV-B vaccine while pregnant. | If you have received the HEPLISAV-B vaccine while pregnant, it is important to talk to your healthcare provider as soon as possible. They can evaluate your individual situation and advise you on the best course of action. HEPLISAV-B is a vaccine used to protect against hepatitis B virus infection, and it is recommended for individuals who are at risk of exposure to the virus. While there is limited data on the safety of this vaccine during pregnancy, it is generally recommended that pregnant women avoid receiving vaccines that contain live viruses. If you received the HEPLISAV-B vaccine during pregnancy, your healthcare provider may recommend additional monitoring during your pregnancy, such as ultrasound or blood tests, to assess the health of your developing fetus. They may also advise you to seek medical attention if you experience any unusual symptoms or complications during your pregnancy. In general, it is important to always discuss any medications, vaccines, or medical procedures with your healthcare provider when pregnant or planning to become pregnant, to ensure the safety of you and your baby. | Yes | No |  | No |
| 58 | Clinical Problems | NA |  |  |  |  |  |  |
| 59 | Clinical Problems | Where can I get the hepatitis B vaccine? | You can get the hepatitis B vaccine at various healthcare facilities such as doctor's offices, clinics, and hospitals. You can start by contacting your primary care physician or local health department to inquire about the vaccine's availability and schedule an appointment. Additionally, many pharmacies also offer vaccinations, so you may want to check with your local pharmacy as well. It is essential to ensure that you receive all three doses of the vaccine to achieve maximum protection against hepatitis B. | The hepatitis B vaccine is widely available in many countries and can be obtained from various healthcare providers. You can check with your local health department or primary care physician to see where the vaccine is offered in your area. In some countries, the vaccine may also be available at pharmacies or travel clinics. It is important to follow the recommended vaccine schedule for hepatitis B to ensure maximum protection against the virus. If you are traveling to a country where hepatitis B is prevalent, it is recommended that you get vaccinated before your trip. Additionally, healthcare workers and those with certain medical conditions may also be recommended to get the hepatitis B vaccine. It is important to consult with your healthcare provider to determine if the hepatitis B vaccine is recommended for you and to discuss any questions or concerns you may have about the vaccine. | Yes | Yes |  | Yes |
| 59 | Clinical Problems | 乙肝疫苗获取途径有哪些？ | 乙肝疫苗是预防乙型肝炎的有效措施之一，以下是获取乙肝疫苗的途径： 1.公立医院：在公立医院接种乙肝疫苗，通常是免费的，可以通过预约或现场排队的方式获得。 2.私立医院：私立医院也可以提供乙肝疫苗接种服务，但需要自费，费用可能会比公立医院高。 3.社区卫生服务中心：社区卫生服务中心也提供免费的乙肝疫苗接种服务。 4.诊所：一些私人诊所也可以提供乙肝疫苗接种服务，但需要自费。 5.政府的免疫接种计划：在某些国家，政府会提供免费的乙肝疫苗接种计划，这些计划通常适用于儿童和青少年。 总的来说，可以通过公立医院、私立医院、社区卫生服务中心、私人诊所以及政府的免疫接种计划等途径获取乙肝疫苗。 | 乙肝疫苗可以通过以下途径获取： 1.公共卫生机构：乙肝疫苗是我国国家免疫规划中的一种疫苗，可以在公共卫生机构（如社区卫生服务中心、医院等）接种。根据规定，新生儿出生后应在出生后24小时内接种第一剂乙肝疫苗。 2.私立医院：一些私立医院也可以提供乙肝疫苗接种服务。但是，这些机构的疫苗可能价格较高，建议到正规的公共卫生机构接种。 3.药店：一些大型药店也可以提供乙肝疫苗接种服务，但是这些机构的疫苗价格较高，建议到正规的公共卫生机构接种。 4.在线购买：一些药店、医院等机构提供在线购买乙肝疫苗的服务，但是建议到正规的公共卫生机构接种，避免购买假冒伪劣的疫苗。 需要注意的是，乙肝疫苗是一种疫苗，接种前应当咨询医生，了解自己的疫苗接种史和身体状况。 | Yes | Yes |  | Yes |
| 60 | Clinical Problems | How serious is chronic (long-term) hepatitis B? | Chronic hepatitis B is a serious viral infection that affects the liver. It is caused by the hepatitis B virus (HBV), which can lead to inflammation and damage to the liver over time. The severity of chronic hepatitis B can vary widely among individuals, and some people may have no symptoms while others may develop serious complications. If left untreated, chronic hepatitis B can lead to liver damage, cirrhosis, liver failure, and liver cancer. However, not everyone with chronic hepatitis B will develop these complications, and some people can live with the infection for many years without experiencing any problems. It's important to note that chronic hepatitis B can be managed with antiviral medications, and treatment can significantly reduce the risk of developing liver damage and other complications. It's also important to take steps to protect your liver, such as avoiding alcohol and certain medications that can be harmful to the liver. If you think you may have been exposed to hepatitis B or if you have any concerns about your liver health, it's important to speak with a healthcare provider. They can perform tests to check for the virus and determine the best course of treatment. | Chronic hepatitis B is a serious liver infection caused by the hepatitis B virus (HBV) that lasts more than six months. The severity of chronic hepatitis B can vary from mild to severe, depending on several factors such as age, sex, immune status, and the presence of other health conditions. In some people, chronic hepatitis B can be asymptomatic or cause only mild symptoms. However, in others, it can lead to serious complications such as cirrhosis (scarring of the liver), liver failure, and liver cancer. People with chronic hepatitis B are also at increased risk of developing other infections, such as HIV and hepatitis D. It is important to note that chronic hepatitis B is a leading cause of liver cancer worldwide. In fact, people with chronic hepatitis B are up to 100 times more likely to develop liver cancer than those without the virus. Fortunately, there are effective treatments available for chronic hepatitis B that can help slow down or even stop the progression of the disease, and reduce the risk of liver damage and liver cancer. It is important for people with chronic hepatitis B to receive regular medical care and follow their doctor's advice to manage their condition effectively. | Yes | Yes |  | Yes |
| 60 | Clinical Problems | 慢性乙肝严重吗 | 慢性乙肝是一种由乙型肝炎病毒（HBV）引起的肝炎，它可以长期存在于感染者的肝脏中。在某些情况下，慢性乙肝可以引起肝脏炎症和纤维化，甚至导致肝硬化和肝癌等严重后果。但并非所有感染HBV的人都会进展到这种情况。 慢性乙肝的严重程度取决于多个因素，包括感染时病毒量的高低、感染时间的长短、感染者的年龄和性别、是否同时感染其他病毒或患有其他疾病等等。一般来说，对于大多数成年人而言，慢性乙肝通常是一种慢性、渐进性的疾病，可能会导致肝脏损伤，但是患者的病情通常比较稳定，不会出现严重的并发症。 但是，对于婴儿和年幼的儿童而言，如果感染乙肝病毒，他们的免疫系统可能无法有效地应对病毒，因此他们很可能会进展到慢性乙肝，这种情况下，患者的病情可能会比成年人更严重。 总的来说，如果你感染了HBV，应该尽快就医，接受医生的治疗和建议，以预防并发症的发生，并维持良好的生活习惯和饮食习惯，以保持肝脏的健康。 | 慢性乙肝是一种由乙型肝炎病毒（HBV）引起的慢性病毒性肝炎。其严重程度因人而异，取决于感染者的免疫状态和病毒的活性水平。 对于某些人来说，慢性乙肝可能不会引起明显的症状，并且可能会在数十年内持续存在。但是，对于另一些人来说，慢性乙肝可能会导致严重的肝脏病变，如肝硬化和肝癌。 肝硬化是一种肝脏疾病，其特征是肝脏组织的瘢痕化和纤维化，从而导致肝脏功能受损。肝癌是一种恶性肿瘤，通常与慢性肝病有关，包括慢性乙肝。 因此，慢性乙肝可以是一种严重的疾病，需要定期监测和治疗。如果您感染了乙肝病毒，建议咨询医生，以便制定个性化的治疗计划和监测方案。 | Yes | Yes |  | Yes |
| 61 | Clinical Problems | Who should be tested for hepatitis B? | The Centers for Disease Control and Prevention (CDC) recommends that certain groups of people should be tested for hepatitis B. These groups include: 1.Infants born to mothers who have hepatitis B. 2.People who were born in countries where hepatitis B is common, such as in Asia, Africa, and parts of South America. 3.People who have ever injected drugs. 4.Men who have sex with men. 5.People with HIV or hepatitis C. 6.People who are on hemodialysis for kidney failure. 7.Healthcare workers who are at risk of exposure to blood or body fluids. In addition, the CDC recommends that all pregnant women should be tested for hepatitis B as part of routine prenatal care. It's important to note that these recommendations may vary depending on the individual's risk factors and health status. It's best to consult a healthcare provider to determine whether hepatitis B testing is necessary for you. | Hepatitis B is a viral infection that affects the liver. It can cause both acute and chronic infections, and in some cases, lead to severe liver damage and liver cancer. Here are the groups of people who should consider getting tested for hepatitis B: 1.Infants born to mothers who are infected with hepatitis B. 2.Individuals who have had unprotected sexual contact with someone who has hepatitis B or who is at high risk of the infection, such as people who have multiple sexual partners or who are men who have sex with men. 3.Healthcare workers who may come into contact with blood or body fluids of infected individuals. 4.People who inject drugs or who share needles or other drug paraphernalia. 5.People who have lived in regions with high rates of hepatitis B infection, such as Asia, Africa, and the Pacific Islands. 6.Individuals who have received a blood transfusion before 1992 or who have received an organ transplant. 7.People with liver disease or who are HIV positive. 8.Individuals who work or reside in settings where there is a risk of exposure to blood or other bodily fluids, such as correctional facilities, and those who provide care for individuals with developmental disabilities. It's important to note that some people with hepatitis B may not have any symptoms, so getting tested is the only way to know for sure if you have the virus. Testing can also help prevent the spread of the infection and provide opportunities for treatment and management of the condition. If you're unsure about whether you should get tested for hepatitis B, it's best to speak with your healthcare provider. | No | No |  | Yes |
| 61 | Clinical Problems | 哪些人应该检查乙肝 | 根据世界卫生组织（WHO）的建议，以下人群应该进行乙肝病毒感染的检测： 1.所有新生儿，应在出生后24小时内接种乙肝疫苗，并接受乙肝病毒的检测。 2.所有未曾感染乙肝病毒的家庭成员和性伴侣，特别是乙肝病毒携带者的家庭成员和性伴侣。 3.所有感染了乙肝病毒的人的亲属，因为他们可能也会感染乙肝病毒。 4.所有医疗和卫生保健工作者、卫生服务提供者，因为他们的工作性质决定了他们与乙肝病毒感染的风险较高。 5.所有注射毒品的人和其他高危人群，如性工作者等。 6.所有需要接受血液透析或器官移植的人，因为他们需要接受血液或血液制品的输注。 7.所有需要接受化疗或免疫抑制治疗的人，因为这些治疗可能会削弱他们的免疫系统。 所有计划前往乙肝高流行区域的人，因为这些地区的乙肝病毒感染率较高。 总之，如果你认为自己处于乙肝病毒感染的高风险群体之一，或者有乙肝病毒感染的症状，应尽快进行乙肝病毒的检测。 | 乙肝是一种由乙型肝炎病毒引起的肝炎，它可以引起肝脏炎症和损害。以下人群应该接受乙肝检查： 1.新生儿：所有新生儿在出生后24小时内应接受乙肝疫苗接种和血清学检查。 2.家庭成员和性伴侣：已知患有乙肝的人、或曾经感染过乙肝的人、或乙肝病毒携带者的家庭成员和性伴侣应接受乙肝检查。 3.医务人员：由于医务人员在工作中可能会接触到乙肝患者，他们应接受乙肝检查。 4.妊娠妇女：所有妊娠妇女应在孕前或孕期接受乙肝检查。 5.有肝病或疑似肝病的人：有肝病史或疑似肝病的人应接受乙肝检查。 6.移民或旅居国外人员：移民或旅居国外的人员应接受乙肝检查。 7.静脉吸毒者：静脉吸毒者因共用注射器或针头易感染乙肝病毒，应接受乙肝检查。 8.其他人群：其他具有感染乙肝风险的人群，如接受血液或血制品、注射毒品、进行皮肤刺青和纹身等人群，也应接受乙肝检查。 | Yes | No | No | No |
| 62 | Clinical Problems | How do I know if I have hepatitis B? | Hepatitis B is a viral infection that affects the liver. Some people with hepatitis B may not experience any symptoms, while others may have symptoms that range from mild to severe. Here are some common signs and symptoms of hepatitis B: 1.Fatigue and weakness 2.Loss of appetite 3.Nausea and vomiting 4.Abdominal pain and discomfort 5.Jaundice (yellowing of the skin and eyes) 6.Dark urine and pale stools 7.Joint pain 8.Fever If you have been exposed to hepatitis B, it is important to get tested. Your healthcare provider can order a blood test to determine if you have been infected with the virus. The blood test will look for antibodies that your body produces in response to the virus. If you have been diagnosed with hepatitis B, it is important to seek medical treatment. Treatment options include antiviral medications and supportive care to manage symptoms. In some cases, hepatitis B can lead to serious complications, such as cirrhosis and liver cancer, so it is important to get diagnosed and treated as soon as possible. | Hepatitis B is a viral infection that affects the liver. The symptoms of hepatitis B can vary from mild to severe, and some people may not experience any symptoms at all. Here are some common signs and symptoms of hepatitis B: 1.Fatigue and weakness 2.Jaundice (yellowing of the skin and eyes) 3.Abdominal pain 4.Nausea and vomiting 5.Loss of appetite 6.Dark urine 7.Clay-colored stools 8.Joint pain If you are experiencing any of these symptoms, you should see a healthcare provider. They may perform a blood test to check for the hepatitis B virus. In some cases, a liver biopsy may be needed to determine the extent of liver damage. It is important to note that hepatitis B can also be asymptomatic, which means you may not experience any symptoms even if you have the virus. Therefore, it is recommended that people at high risk for hepatitis B, such as those who have had unprotected sex, shared needles, or been in contact with infected blood, get tested for the virus even if they have no symptoms. | Yes | Yes |  | Yes |
| 62 | Clinical Problems | 如何确定自己有乙肝 | 乙肝是一种由乙型肝炎病毒引起的病毒性肝炎，通常表现为疲劳、食欲不振、肝区疼痛、黄疸等症状。以下是一些常见的确定自己是否患有乙肝的方法： 1.检测乙肝病毒标志物：乙肝病毒标志物包括HBsAg（乙肝表面抗原）、HBcAg（乙肝核心抗原）、HBeAg（乙肝e抗原）等，可以通过血液检测来确定是否感染了乙肝病毒。 2.检查肝功能：肝功能异常是乙肝病毒感染的常见表现，可以通过血液检查肝功能指标如ALT、AST等来判断肝脏是否受损。 3.观察症状：乙肝病毒感染常常伴随着疲劳、食欲不振、肝区疼痛、黄疸等症状，如果出现了这些症状，应该考虑是否感染了乙肝病毒。 | 如果您怀疑自己患有乙肝，建议尽快到医院进行检查和诊断。及早发现和治疗乙肝可以避免病情恶化和传染给他人。 1.确定自己是否感染了乙肝需要进行相关检测。以下是一些可能用到的检测方法： 2.乙肝病毒表面抗原检测：乙肝病毒表面抗原（HBsAg）是乙肝病毒感染的最早和最可靠的标志物之一。如果HBsAg检测结果呈阳性，说明您感染了乙肝病毒。 3.乙肝病毒表面抗体检测：如果您的乙肝病毒表面抗体（HBsAb）检测结果呈阳性，说明您曾经感染过乙肝病毒，或者曾经接种过乙肝疫苗。 4.乙肝病毒核心抗体检测：乙肝病毒核心抗体（HBcAb）是一种抗体，可以表明您曾经感染过乙肝病毒。 5.乙肝病毒e抗原检测：乙肝病毒e抗原（HBeAg）是一种表明乙肝病毒正在大量繁殖的标志。如果HBeAg检测结果呈阳性，说明您的病情较严重，乙肝病毒正在活跃。 如果您怀疑自己感染了乙肝病毒，建议前往医院或诊所进行相关检测。通过这些检测，医生可以对您的乙肝病毒感染状况进行评估，并为您提供相应的治疗建议。 | No | No |  | No |
| 63 | Clinical Problems | What should I do after learning that I have hepatitis B? | If you have been diagnosed with hepatitis B, it is important to take action to manage the condition and protect your health as well as the health of others. Here are some steps you can take: 1.Consult with a healthcare provider: It is important to speak with a healthcare provider who can evaluate your condition and provide guidance on how to manage your hepatitis B. Your healthcare provider may recommend additional tests to determine the extent of liver damage and help determine the appropriate course of treatment. 2.Follow recommended treatment plan: Depending on the severity of your hepatitis B, your healthcare provider may recommend antiviral medication to slow or stop the virus from causing further damage to your liver. It is important to follow your recommended treatment plan as directed. 3.Get vaccinated: If you have not already been vaccinated against hepatitis B, it is important to get vaccinated to protect yourself from additional infections. The vaccine is safe and effective. 4.Practice safe sex: Hepatitis B can be spread through sexual contact, so it is important to practice safe sex by using condoms and limiting the number of sexual partners. 5.Avoid sharing personal items: Hepatitis B can also be spread through sharing personal items such as razors, toothbrushes, or needles. It is important to avoid sharing these items with others to prevent the spread of the virus. 6.Educate yourself and others: Learn more about hepatitis B and how it is spread. Share this information with your loved ones to help prevent the spread of the virus. Remember, with proper management and care, people with hepatitis B can lead healthy and productive lives. | If you have been diagnosed with hepatitis B, it is important to take the following steps: Seek medical treatment: Hepatitis B is a serious liver infection that requires medical attention. You should see a doctor who specializes in liver diseases, such as a gastroenterologist or a hepatologist. Your doctor may prescribe antiviral medications to help manage the infection and prevent liver damage. Follow your doctor's advice: Your doctor may recommend lifestyle changes to help manage your hepatitis B infection. This may include avoiding alcohol and certain medications that can harm the liver, getting regular checkups to monitor your liver function, and getting vaccinated against hepatitis A and B. Inform your sexual partner and family members: Hepatitis B is contagious and can be spread through blood and bodily fluids. It is important to inform your sexual partner and family members so they can get tested and vaccinated if necessary. Practice good hygiene: Hepatitis B can be spread through contact with infected blood or bodily fluids. To reduce your risk of spreading the infection to others, practice good hygiene by washing your hands frequently and avoiding sharing personal items like razors and toothbrushes. Join a support group: Living with a chronic condition like hepatitis B can be challenging. Joining a support group can help you connect with others who are going through a similar experience and provide you with emotional support. Remember that hepatitis B is a manageable condition with the right medical care and lifestyle changes. It is important to follow your doctor's advice and take steps to protect your health and the health of others. | No | Yes | No | No |
| 63 | Clinical Problems | 知道自己乙肝后应该怎么办 | 如果你知道自己患有乙肝，建议你采取以下措施： 寻求医生的帮助：乙肝是一种慢性疾病，需要专业医生进行治疗和管理。建议你尽快寻求医生的帮助，进行详细的检查和诊断，以便制定适当的治疗计划。 遵守医嘱：医生会根据你的身体状况和乙肝病毒的活动程度，制定适当的治疗计划。你需要严格遵守医生的建议，按时服药或接受其他治疗，以保持病情稳定。 注重饮食：乙肝患者需要注重饮食，特别是应该避免摄入过多的脂肪和糖分，增加蛋白质的摄入量，有助于维持身体的健康和免疫力。 健康生活方式：保持健康的生活方式可以帮助乙肝患者降低病情加重的风险，包括戒烟限酒，适量运动，保持充足的睡眠等。 注意传播途径：乙肝是一种可以通过血液、体液等途径传播的疾病，因此要注意避免与他人分享个人用品、器具等，并注意性生活安全，避免传染给他人。 | 如果你发现自己感染了乙肝病毒，应该采取以下步骤： 1.寻求医疗帮助：首先，你需要寻求医疗帮助，咨询医生或专业医疗机构的医生，了解自己感染的乙肝病毒类型、病情和治疗方法。 2.接受检查：你需要进行一系列检查，包括肝功能、病毒量、肝脏超声等，以了解肝脏状况和病毒感染情况。 3.接受治疗：如果你的病情需要治疗，你应该接受专业医生的治疗建议和方案。目前有多种乙肝病毒治疗方案，包括抗病毒治疗、免疫调节剂等。 4.采取预防措施：为了防止乙肝病毒的传播，你需要采取措施，如不与他人共用个人卫生用品，不接触他人的血液和体液，避免性接触等。 5.注意饮食和生活：在治疗期间，你需要注意饮食和生活方式，保持身体健康，避免过度饮酒和疲劳。 需要注意的是，乙肝病毒感染是一种可以控制和治疗的疾病，及早发现和治疗可以提高治疗成功的机会。如果你感到焦虑或有其他的情绪问题，你也可以考虑寻求心理健康咨询师的帮助。 | No | Yes | No | No |
| 64 | Clinical Problems | If I am pregnant should I get tested for hepatitis B? | Yes, it is recommended that pregnant women get tested for hepatitis B. Hepatitis B is a viral infection that can be transmitted from mother to baby during childbirth, and it can cause serious health problems for the baby. Therefore, it is important to identify if a pregnant woman is infected with hepatitis B, so that appropriate measures can be taken to prevent transmission to the baby. Your healthcare provider can perform a simple blood test to check for hepatitis B. If you test positive for hepatitis B, your healthcare provider can recommend treatment options to help manage the infection and reduce the risk of transmission to your baby. They may also recommend that your baby receive the hepatitis B vaccine and hepatitis B immune globulin (HBIG) at birth to further reduce the risk of transmission. | Yes, it is recommended that all pregnant women be tested for hepatitis B. Hepatitis B is a viral infection that can be passed from a mother to her baby during childbirth. If a pregnant woman is infected with hepatitis B, there is a risk that her baby may become infected as well. Testing for hepatitis B during pregnancy is important because if the mother is infected, steps can be taken to reduce the risk of transmission to the baby. For example, the baby can be given the hepatitis B vaccine and hepatitis B immune globulin (HBIG) soon after birth, which can greatly reduce the risk of transmission. If you are pregnant, talk to your healthcare provider about getting tested for hepatitis B. They can advise you on when and where to get tested and what steps to take if you are found to be infected. | Yes | Yes |  | Yes |
| 64 | Clinical Problems | 怀孕后需要检测乙肝吗 | 是的，怀孕后需要进行乙肝病毒检测。乙肝病毒是一种可以通过血液、性行为和母婴传播的病毒。如果孕妇感染了乙肝病毒，婴儿有可能在出生时或后期感染该病毒。 如果孕妇感染乙肝病毒，医生会根据孕妇的病情和婴儿的风险进行治疗。如果孕妇没有感染乙肝病毒，医生可能会建议孕妇接种乙肝疫苗以预防感染。 因此，如果怀孕了，建议妇女在妊娠早期进行乙肝病毒筛查。如果您怀孕前未接种乙肝疫苗，医生可能会建议您接种。如果您已经接种了乙肝疫苗，请在怀孕前检查抗体水平，以确保您仍然具有充足的免疫力。 | 是的，孕妇需要进行乙肝病毒感染的检测。这是因为乙肝病毒可以通过母婴传播，如果孕妇感染了乙肝病毒，有可能会将病毒传染给新生儿，导致新生儿感染乙肝病毒的风险增加。 建议孕妇在怀孕期间接受医生的建议进行乙肝病毒感染的检测，特别是在乙肝高流行地区、乙肝病史家族史、以及孕妇出现乙肝相关症状时，都应该尽早检测。如果发现孕妇感染了乙肝病毒，医生会根据孕妇的具体情况制定相应的治疗计划和产前预防措施，以降低新生儿感染的风险。 | Yes | Yes |  | Yes |
| 65 | Clinical Problems | If a pregnant person has hepatitis B, is there a way to prevent their baby from getting hepatitis B? | Yes, there are ways to prevent transmission of hepatitis B from a pregnant person to their baby. One important method is to administer the hepatitis B vaccine to the newborn baby within 12 hours of birth. This vaccine is highly effective in preventing transmission of the virus from mother to child. In addition to the vaccine, newborns born to mothers with hepatitis B should also receive hepatitis B immune globulin (HBIG) at birth. HBIG is a medication that provides additional protection against hepatitis B infection. It is also important for pregnant people with hepatitis B to receive appropriate medical care during their pregnancy to monitor their liver health and to receive antiviral medications, if necessary, to reduce the amount of virus in their blood. Overall, with proper medical care and appropriate interventions, the risk of transmission of hepatitis B from a pregnant person to their baby can be greatly reduced. | Yes, there are ways to prevent the transmission of hepatitis B from a pregnant person to their baby. Hepatitis B is a viral infection that can be passed from an infected mother to her baby during childbirth. However, the transmission rate can be significantly reduced by following certain measures. Here are some ways to prevent the transmission of hepatitis B from a pregnant person to their baby: Hepatitis B vaccine: The hepatitis B vaccine is very effective at preventing the transmission of hepatitis B from a mother to her baby. Pregnant women who are carriers of the virus are recommended to receive the vaccine during pregnancy. The vaccine can be given at any time during pregnancy and is safe for both the mother and baby. Hepatitis B immune globulin (HBIG): HBIG is a medication that provides short-term protection against hepatitis B. It is given to the baby immediately after birth to prevent infection. HBIG is most effective when given within 12 hours of birth. Antiviral medication: Antiviral medications such as tenofovir or lamivudine can be given to the pregnant person during the last trimester to reduce the risk of transmission. Caesarean section delivery: In some cases, a caesarean section delivery may be recommended to reduce the risk of transmission of the virus during childbirth. It is important for pregnant individuals with hepatitis B to work closely with their healthcare provider to determine the best course of action to prevent transmission to their baby. With proper management, the risk of transmission can be significantly reduced. | No | Yes | No | No |
| 65 | Clinical Problems | 孕妇乙肝如何防止传给孩子 | 孕妇乙肝病毒感染可以通过以下几种方式防止传染给新生儿： 1.进行乙肝疫苗接种：孕妇在怀孕前或怀孕早期可以接种乙肝疫苗，这样可以减少乙肝病毒对新生儿的传播风险。 2.接受孕期监测和治疗：孕妇在怀孕期间需要定期接受医生的监测，以确保乙肝病毒感染没有对胎儿产生影响。如果孕妇确诊为乙肝病毒感染，医生会对其进行相应的治疗。 3.避免母婴传播：孕妇要避免与乙肝病毒感染者接触，避免性行为、血液和其他体液的接触等。此外，在分娩过程中，孕妇需要避免感染的风险，比如剖腹产等。 4.给新生儿接种乙肝疫苗：新生儿出生后，应及时接种乙肝疫苗，这样可以避免乙肝病毒的传播。 需要注意的是，如果孕妇已经感染了乙肝病毒，不能完全避免母婴传播的风险，因此需要在医生的指导下进行监测和治疗。 | 孕妇如果携带乙肝病毒，有可能会将病毒传给胎儿，导致胎儿感染乙肝。以下是预防孕妇将乙肝病毒传给胎儿的几种方法： 1.接种乙肝疫苗：孕妇在怀孕前接种乙肝疫苗可以有效预防乙肝病毒的传播，同时也可以保护自己和胎儿免受感染。 2.孕期监测：孕妇如果已经携带乙肝病毒，需要在孕期定期进行监测，以确保病毒的繁殖程度控制在安全范围内。 3.避免感染：孕妇应该避免接触可能传播乙肝病毒的物品和人员，比如血液和血制品、注射器、针头、口腔器具等。 4.妊娠期间药物治疗：如果孕妇病毒复制较为活跃，需要在医生指导下使用药物治疗，以控制病毒繁殖，减少感染胎儿的风险。 总之，孕妇携带乙肝病毒应该积极预防和治疗，避免将病毒传给胎儿。在怀孕前就应该接种乙肝疫苗，如果已经患有乙肝病毒，应该在孕期及时监测和治疗，同时避免接触可能传播病毒的物品和人员。 | Yes | Yes |  | Yes |
| 66 | Clinical Problems | What can people with chronic hepatitis B do to take care of their liver? | Chronic hepatitis B is a viral infection that can cause inflammation of the liver and lead to liver damage over time. While there is currently no cure for chronic hepatitis B, there are several things that people with the condition can do to take care of their liver: Get regular check-ups: It is important for people with chronic hepatitis B to get regular check-ups with their healthcare provider. This may include blood tests to monitor liver function and to check for signs of liver damage. Take medication as prescribed: There are several antiviral medications available that can help slow down or stop the progression of chronic hepatitis B. It is important to take these medications exactly as prescribed by a healthcare provider. Avoid alcohol: Drinking alcohol can further damage the liver, so people with chronic hepatitis B should avoid alcohol or drink it in moderation. Eat a healthy diet: A healthy diet can help support liver function. This may include foods that are high in fiber, such as fruits and vegetables, and lean protein sources. Exercise regularly: Regular exercise can help improve overall health and may also help improve liver function. Practice safe sex: Chronic hepatitis B is spread through blood and body fluids, including semen and vaginal fluids. It is important to practice safe sex to prevent the spread of the virus to others. Get vaccinated for other hepatitis viruses: People with chronic hepatitis B are at increased risk for other types of hepatitis, such as hepatitis A and C. Vaccines are available to help prevent these infections. It is important for people with chronic hepatitis B to work closely with their healthcare provider to develop a personalized care plan that meets their specific needs and helps protect their liver health. | Chronic hepatitis B is a serious viral infection that affects the liver and can lead to liver damage, cirrhosis, and liver cancer. If you have been diagnosed with chronic hepatitis B, there are several things you can do to take care of your liver and help manage the condition: Follow a healthy diet: A healthy diet is essential for people with chronic hepatitis B. A diet that is low in fat, salt, and processed foods can help reduce the workload on the liver and promote liver health. Avoid alcohol: Drinking alcohol can further damage the liver and worsen the effects of chronic hepatitis B. If you have chronic hepatitis B, it's important to avoid alcohol altogether. Get vaccinated for hepatitis A and B: People with chronic hepatitis B are at higher risk of developing hepatitis A and hepatitis B. Getting vaccinated for both of these viruses can help protect the liver. Take medication: There are several antiviral medications that can help manage chronic hepatitis B and reduce the risk of liver damage. Talk to your doctor about which medications are right for you. Monitor your liver function: People with chronic hepatitis B should have regular liver function tests to monitor the health of their liver. Your doctor may also recommend imaging tests or biopsies to assess liver damage. Practice safe sex: Hepatitis B can be transmitted through sexual contact. Practicing safe sex, including using condoms, can help reduce the risk of transmitting the virus to others or acquiring a new infection. Avoid sharing personal items: Hepatitis B can be transmitted through sharing personal items, such as razors, toothbrushes, or needles. Avoid sharing these items to reduce the risk of transmitting the virus. It's important to work closely with your doctor to develop a treatment plan that is right for you and to monitor the health of your liver regularly. With proper care and management, many people with chronic hepatitis B can live healthy lives. | No | No |  | No |
| 66 | Clinical Problems | 慢乙肝患者如何护肝 | 对于慢性乙肝患者，护肝非常重要，以下是一些建议： 1.遵守医生的治疗方案：慢性乙肝患者需要按照医生的建议进行治疗。常见的治疗方式包括抗病毒治疗和免疫调节治疗。请按时服药，并定期复查。 2.控制饮食：建议少吃高脂、高糖、高盐、高胆固醇、高蛋白等食物，多吃新鲜蔬菜、水果、粗粮等，保持饮食均衡。 3.饮食清淡：少喝酒，不吸烟，避免暴饮暴食，不吃过于刺激的食物。 4.定期检查：定期进行肝功能检查和病毒指标检查，及时发现病情变化。 5.避免接触有害物质：避免暴露在有害物质中，如化学品、重金属等，以免对肝脏造成伤害。 6.保持身体健康：加强体育锻炼，控制体重，保持充足睡眠，减轻压力，增强免疫力，有助于维持肝脏健康。 总之，慢性乙肝患者应该综合采取多种措施，保护肝脏，预防肝病的发展。同时，要定期到医院接受专业医生的指导和治疗。 | 慢性乙型肝炎是一种由乙型肝炎病毒引起的慢性病毒性肝炎，对肝脏有一定的损害。以下是一些护肝的建议： 1.保持健康的生活方式：避免饮酒和吸烟，控制体重，避免摄入过多脂肪和糖分的食物。 2.避免使用肝毒性药物：一些药物可能会对肝脏造成损害，因此在使用药物之前最好咨询医生的意见。 3.保持适当的运动：适当的运动可以帮助增强肝脏功能，减少肝脏负担。 4.注意饮食：饮食应以清淡、易消化、富含蛋白质、维生素和矿物质的食物为主。同时要保证饮食的卫生，避免食用过期或不洁的食物。 5.定期进行体检：定期进行体检，及时发现和治疗疾病，保护肝脏。 6.按医生建议进行治疗：如果患者有需要，应按医生的建议进行药物治疗，及时控制病情，减轻肝脏负担。 需要注意的是，慢性乙型肝炎是一种慢性病毒性肝炎，需要长期的治疗和护理。如果您是慢性乙型肝炎患者，请一定要定期到医院进行检查和治疗。 | No | No |  | No |
| 67 | Clinical Problems | I'm Hepatitis B Reactive, can i still have a healthy baby? | Yes, it is possible for you to have a healthy baby if you have Hepatitis B. However, it is important to take certain precautions to prevent the transmission of the virus to your baby. First, you should consult with a healthcare provider who can give you guidance on how to manage your Hepatitis B infection during pregnancy. They may recommend antiviral medications to reduce the risk of transmission to your baby. Second, it is important to ensure that your baby receives the Hepatitis B vaccine and hepatitis B immune globulin (HBIG) within 12 hours of birth. This can significantly reduce the risk of your baby contracting the virus. Third, you should avoid any behaviors or activities that may increase the risk of transmission of the virus to your baby. This may include avoiding sharing personal items such as razors or toothbrushes, and avoiding breastfeeding if your nipples are cracked or bleeding. By taking these precautions and working closely with your healthcare provider, you can increase the chances of having a healthy baby despite being Hepatitis B reactive. | Yes, it is possible for a person who is Hepatitis B Reactive to have a healthy baby with proper medical management and precautions. However, it is important to seek medical advice and treatment to minimize the risk of transmission of the virus to the baby. Here are some steps that can help ensure a healthy pregnancy and baby: Consult with a healthcare provider: If you have been diagnosed with Hepatitis B, it is important to consult with a healthcare provider who can provide you with the necessary medical care and guidance. Get vaccinated: Your healthcare provider may recommend that you receive the Hepatitis B vaccine, which can help protect your baby from getting the virus. Monitor your viral load: Your healthcare provider may also recommend monitoring your viral load during pregnancy to help reduce the risk of transmitting the virus to your baby. Consider antiviral medication: In some cases, your healthcare provider may recommend antiviral medication to help reduce the risk of transmitting the virus to your baby. Take precautions during delivery: Your healthcare provider may recommend delivering your baby via cesarean section to further reduce the risk of transmission. Additionally, your baby may receive immunoglobulin injections and the Hepatitis B vaccine at birth to help prevent infection. By working closely with your healthcare provider and following their recommendations, you can increase the chances of having a healthy baby despite being Hepatitis B Reactive. | No | No |  | Yes |
| 67 | Clinical Problems | 乙肝肝炎活动期能要小孩吗 | 如果您患有乙型肝炎并处于活动期，最好在考虑生育之前咨询医生的建议。因为在活动期，乙型肝炎病毒的数量在血液中很高，这增加了传染给婴儿的风险。 如果您想要怀孕，医生可能会建议您在乙肝病毒得到控制之后再尝试。治疗乙肝肝炎的方法包括使用抗病毒药物、注射乙肝疫苗和接受其他治疗措施。治疗的目的是将病毒负载降至很低，从而减少传染风险。 如果您已经怀孕，医生可能会建议您采取措施来减少婴儿接触病毒的风险。例如，让新生儿接种乙肝疫苗、避免接触您的血液或其他体液、及时治疗任何感染或其他健康问题等。 总之，如果您计划怀孕或已经怀孕并患有乙肝肝炎，请与医生咨询并根据医生的建议进行相应的处理。 | 如果您是患有乙肝肝炎的女性，建议在考虑生育之前先咨询您的医生。乙肝肝炎是一种病毒性感染，可能对孩子和母亲的健康造成影响。在乙肝肝炎活动期，即乙肝病毒DNA检测呈阳性的情况下，建议暂缓生育，直到乙肝病毒活动期得到控制。因为在这个时期，妊娠期间的激素变化和免疫抑制可能会使乙肝肝炎的病情恶化，增加胎儿感染的风险。如果您计划要小孩，请务必与您的医生密切合作，制定一个适合您情况的计划，并遵循医生的建议进行孕前检查和治疗。 | No | No |  | No |
| 68 | Clinical Problems | I was just told to be Hepatitis B Reactive. If it become severe, what will be the symptoms? | Hepatitis B is a viral infection that affects the liver. In most cases, people with hepatitis B have no symptoms, especially in the early stages of the disease. However, if the infection becomes severe, you may experience some of the following symptoms: 1. Abdominal pain 2. Dark urine 3. Yellowing of the skin and eyes (jaundice) 4. Fatigue 5. Nausea and vomiting 6. Loss of appetite 7. Joint pain 8. Clay-colored stools It's important to note that not everyone with hepatitis B will experience these symptoms. Some people may have no symptoms at all. If you have been diagnosed with hepatitis B, it's important to follow up with your healthcare provider for proper evaluation and management of the disease. | Hepatitis B is a viral infection that can cause inflammation of the liver, and it is spread through contact with blood or other body fluids of an infected person. The severity of symptoms can vary depending on the individual and the stage of the infection. In some cases, individuals with Hepatitis B may not experience any symptoms at all, while in others, symptoms may range from mild to severe. Some common symptoms of Hepatitis B include: 1. Fatigue 2. Loss of appetite 3. Nausea and vomiting 4. Abdominal pain 5. Jaundice (yellowing of the skin and eyes) 6. Dark urine 7. Clay-colored stools 8. In severe cases of Hepatitis B, individuals may experience symptoms such as: 9. Bleeding and bruising easily 10. Swollen abdomen due to fluid accumulation 11. Confusion or cognitive impairment 12. Severe liver damage (cirrhosis) 13. Liver failure If you have been diagnosed with Hepatitis B, it is important to speak with your healthcare provider about your condition and any potential symptoms or complications that may arise. Treatment options are available that can help manage symptoms and reduce the risk of liver damage. | No | No |  | No |
| 68 | Clinical Problems | 我现在乙肝肝炎活动期，如果病情加重会有什么表现？ | 乙肝肝炎的活动期分为两种类型：急性和慢性。 急性活动期的症状可能包括： 1.疲乏、乏力 2.肝区疼痛或不适 3.黄疸（黄绿色皮肤和眼睛） 4.恶心、呕吐 5.食欲不振 6.发热、头痛、肌肉酸痛等类似感冒的症状 慢性活动期的症状可能比较轻微或无症状，但可能会引起以下一些症状： 1.乏力、疲劳 2.肝区不适或轻度疼痛 3.肝功能异常（通过血液检查发现） 4.黄疸（在某些情况下） 如果您感觉症状加重或出现新症状，建议立即就医，并告诉医生您的乙肝肝炎病史，以便医生更好地评估和治疗您的病情。同时，注意保持良好的生活习惯，避免过度劳累和饮酒，以保持健康的身体状态。 | 乙肝肝炎活动期是指病毒在体内持续复制和感染肝细胞，导致肝炎病情加重的时期。在乙肝肝炎活动期，病情加重会导致以下表现： 1.黄疸：黄疸是由于肝脏受损，胆红素不能正常代谢，导致皮肤和眼睛呈现黄色的症状。 2.乏力和疲劳：肝炎活动期时，肝脏功能受损，会导致体力下降、疲劳感和乏力。 3.恶心和呕吐：乙肝肝炎活动期时，肝细胞受损会影响胆汁的产生和排泄，导致恶心和呕吐。 4.上腹部不适和疼痛：乙肝肝炎活动期时，肝脏受损会导致上腹部不适和疼痛。 5.腹水：乙肝肝炎活动期时，肝脏功能受损会导致血管内的水分渗出到腹腔内，形成腹水。 6.肝功能异常：在乙肝肝炎活动期，肝功能检查常常会显示肝功能异常。 如果您出现以上症状，请及时就医，接受医生的诊断和治疗。 | Yes | No | No | No |
| 69 | Clinical Problems | With hepatitis B, can I still live longer? | Yes, it is possible to live a long and healthy life with hepatitis B. However, the outcome of the disease can vary depending on several factors such as the age of the person at the time of infection, the degree of liver damage, and whether or not the person has other medical conditions. Hepatitis B is a viral infection that can cause inflammation of the liver. It can lead to chronic liver disease, liver failure, or liver cancer if left untreated. However, many people with hepatitis B do not experience any symptoms and are able to clear the virus on their own. For those who do develop chronic hepatitis B, there are effective treatments available that can help slow down or stop the progression of liver damage. These treatments can include antiviral medications that help suppress the virus and reduce the risk of complications. In addition to medical treatment, it is important for people with hepatitis B to take care of their liver by avoiding alcohol, eating a healthy diet, and getting regular exercise. It is also important to get regular check-ups and screenings for liver cancer. Overall, with proper management and care, many people with hepatitis B are able to live long and healthy lives. | Yes, it is possible to live a long and healthy life with hepatitis B. Many people with hepatitis B are able to manage their condition and prevent it from causing serious health problems by taking appropriate medical care, adopting a healthy lifestyle, and avoiding behaviors that can worsen the condition. The outcome of hepatitis B infection can vary greatly depending on several factors such as age, overall health, viral load, and whether or not the individual has developed chronic hepatitis B. While some people with chronic hepatitis B may eventually develop complications such as liver cirrhosis or liver cancer, others may never experience any serious health problems. It is important to work closely with your healthcare provider to manage your condition and reduce the risk of complications. This may involve taking antiviral medications to reduce viral load, getting regular liver function tests, and avoiding alcohol and other substances that can damage the liver. With appropriate medical care and a healthy lifestyle, many people with hepatitis B are able to lead long and fulfilling lives. | Yes | Yes |  | Yes |
| 69 | Clinical Problems | 得乙肝会影响寿命吗 | 慢性乙型肝炎（乙肝）是由乙型肝炎病毒（HBV）引起的慢性病毒性感染。乙肝会影响肝脏功能，并可能导致肝硬化和肝癌等严重后果。 如果不及时治疗，乙肝可能会影响寿命。乙肝患者在发展成肝硬化和肝癌之前的预后各有不同，但许多患者能够通过药物治疗和定期监测来控制疾病进展，降低患肝硬化和肝癌的风险。同时，积极的生活方式改变和避免饮酒等不良习惯也可以帮助减少肝损害的发生。 因此，如果你感染了乙肝，及早接受医生的治疗和建议，并遵循医生的治疗方案和生活方式建议，可以有效地控制乙肝的进展，提高生活质量并延长寿命。 | 乙型肝炎病毒感染可能会影响寿命，但并非所有感染者都会如此。大多数乙肝患者能够在生活中保持正常，并且没有任何明显的症状或影响他们的寿命。然而，如果乙肝感染长期存在并导致慢性肝炎、肝硬化或肝癌等并发症，这些并发症可能会对寿命造成影响。 肝硬化和肝癌是乙肝感染的常见并发症，尤其是对于长期感染、未接受治疗或治疗效果不佳的患者。这些并发症会导致肝功能受损和肝脏组织破坏，从而影响身体的正常功能。如果这些并发症未得到及时治疗，则可能导致患者死亡。因此，早期诊断和治疗对于减少乙肝病毒感染对寿命的影响非常重要。 | No | No |  | No |
| 70 | Clinical Problems | Can I travel Europe on a tourist visa if I have hepatitis b? | In general, having hepatitis B should not prevent you from traveling to Europe on a tourist visa. Hepatitis B is not a contagious disease that can be transmitted through casual contact, such as by sharing food or drinks, or by being in close proximity to others. However, it's important to keep in mind that visa requirements and medical regulations can vary by country. Some countries may require certain vaccinations or medical exams before granting a visa, and this could include a check for hepatitis B. Therefore, it's important to check the specific requirements for the country you plan to visit. Additionally, if you are taking medication for hepatitis B, you should make sure to bring enough medication for your trip and carry it with you in your hand luggage. You should also check with your doctor before traveling to make sure that your hepatitis B is well-managed and that you are healthy enough to travel. Overall, having hepatitis B should not prevent you from traveling to Europe, but it's important to check the specific visa requirements and medical regulations for the countries you plan to visit, and to take any necessary precautions and medications with you on your trip. | In general, having hepatitis B should not prevent you from traveling to Europe on a tourist visa. However, it is important to note that visa requirements can vary depending on your country of origin and the specific country or countries you plan to visit within Europe. It's always a good idea to check with the embassy or consulate of the country you plan to visit to confirm their visa requirements and any specific health restrictions that may apply. In some cases, you may need to provide documentation of your hepatitis B status or undergo additional health screening upon arrival. It's also important to take steps to protect your health while traveling, such as getting vaccinated against hepatitis B (if you haven't already), practicing good hygiene, and avoiding high-risk activities that could lead to infection. Overall, having hepatitis B should not necessarily prevent you from traveling to Europe on a tourist visa, but it's important to do your research and take appropriate precautions to ensure a safe and enjoyable trip. | Yes | Yes |  | Yes |
| 70 | Clinical Problems | 有乙肝能去欧洲旅游吗 | 可以，但需要注意一些事项。 乙肝是一种病毒性肝炎，主要通过血液和体液传播，包括血液、精液、阴道分泌物等。乙肝病毒在欧洲也是存在的，因此，如果你携带乙肝病毒，也需要注意保护自己，防止传播病毒。 首先，如果你计划前往欧洲旅游，建议在出发前向你的医生咨询是否需要进行乙肝疫苗接种，以预防感染乙肝病毒。如果你已经患有乙肝，建议咨询医生是否需要采取额外的预防措施。 另外，欧洲的医疗服务和卫生条件相对较好，如果你需要接受医疗服务，可以在当地寻找医疗机构进行治疗。但需要注意的是，在旅行过程中保持卫生习惯，避免与他人共用牙刷、剃须刀等个人卫生用品，以防止传播病毒。 最后，如果你计划前往欧洲旅游，建议购买旅游保险，以应对可能出现的突发状况。 | 乙肝病毒是一种传染病毒，但是它的传播方式是通过血液或其他体液的接触，而不是通过空气传播。因此，只要你不与他人共用注射器或个人卫生用品，旅游时并不会对他人构成威胁。不过，建议你在出国前咨询医生，了解你的健康状况是否适合旅游，以及是否需要采取特别的预防措施。另外，在旅游期间注意饮食卫生和个人卫生，避免接触有可能感染乙肝的血液或体液。 | Yes | No | No | No |
| 71 | Clinical Problems | My dad need a liver transplant due to acute hepatitis b, he is 55. What happens if he isn't in good enough health for a transplant? Do they just send him home to die? | I'm sorry to hear that your dad is facing this difficult health issue. In cases where a liver transplant is deemed too risky due to the patient's overall health or other medical conditions, the medical team will work with the patient and their family to develop a treatment plan that can provide the best possible quality of life. This might include medications, dietary changes, and other interventions that can help manage the symptoms and complications of acute hepatitis B. In some cases, the patient may be referred to hospice care or other supportive care programs that can provide emotional and practical support during this difficult time. It's important to keep in mind that every case is unique, and the medical team will work closely with your dad and his family to determine the best course of action based on his individual health status and medical history. It's also worth noting that liver transplants can be very successful in many cases, and your dad's medical team will likely explore all possible options to see if a transplant is a viable option for him. | I'm sorry to hear about your dad's health condition. Generally, eligibility for a liver transplant is determined based on various medical factors, including the severity of the liver disease, overall health, and other medical conditions. If your dad's health is not good enough for a transplant, it may be because he has other medical conditions that make the procedure too risky, or because his liver disease is too advanced to benefit from a transplant. In such cases, the doctors will work with your dad and your family to explore other treatment options that may help manage his symptoms and prolong his life. These options may include medications to help manage the symptoms of his liver disease, such as ascites and encephalopathy, or procedures to drain excess fluid from his abdomen. The doctors may also recommend lifestyle changes, such as a low-salt diet, to help manage his symptoms and improve his overall health. Ultimately, the goal of medical care in such cases is to provide comfort and improve quality of life, while managing the symptoms of the underlying disease. Hospice care may also be an option if your dad's health continues to decline, and the focus of care shifts to managing pain and ensuring a peaceful and comfortable end-of-life experience. It's important to have open and honest communication with your dad's healthcare team, so that you can fully understand his treatment options and make informed decisions about his care. | Yes | Yes |  | Yes |
| 71 | Clinical Problems | 我爸爸55岁，很严重的急性乙肝，需要肝移植，如果情况不好不能移植会怎样，会让他回家等死吗 | 乙肝是一种病毒感染引起的肝炎，它可以导致肝脏炎症和损伤，甚至导致肝硬化和肝癌。对于急性乙肝患者，治疗的关键在于及时采取措施，以避免疾病的恶化。 肝移植是一种治疗重度肝病和肝衰竭的方法，但是它并不适用于所有的患者。对于肝移植的适应症和手术风险需要进行仔细的评估。如果患者不适合肝移植，医生会根据患者的病情和身体状况制定其他治疗方案，如药物治疗、营养支持等，以减轻症状并延长患者的生命。 总的来说，即使患者不能进行肝移植手术，也不会被简单地让回家等死。医生会根据患者的病情和身体状况制定个性化的治疗方案，以帮助患者缓解症状，减轻疼痛，并提高生活质量。 | 我很抱歉听到您爸爸的病情。关于肝移植，确实是一种治疗乙肝肝炎的有效方法之一。然而，肝移植手术本身是一项高风险的手术，需要患者在手术前和手术后接受严格的管理和监护。因此，医生会对患者进行全面的评估，以确定是否适合进行肝移植手术。 如果医生认为您爸爸的情况不适合进行肝移植手术，他们可能会考虑其他治疗方法，例如药物治疗或其他的手术治疗。这些治疗方法可能无法完全治愈乙肝肝炎，但可以帮助控制疾病的发展，并且可能能够延长您爸爸的生命。 如果医生认为没有任何治疗方法可以延长您爸爸的生命，他们可能会建议进行姑息治疗。姑息治疗是一种以减轻症状为主的治疗方法，它的目的是使患者在有限的时间内获得最高质量的生活。 总之，医生会根据患者的具体情况，提供最合适的治疗方案。即使肝移植手术不可行，仍有许多其他治疗方法可供选择，可以帮助患者减轻症状，延长寿命。 | No | Yes | No | No |
| 72 | Clinical Problems | Is it safe to have hepatitis B vaccine 7 days after my second dose of covid-19 vaccine? | Yes, it is generally safe to have the hepatitis B vaccine 7 days after your second dose of the COVID-19 vaccine. According to the Centers for Disease Control and Prevention (CDC), the COVID-19 vaccine can be administered with other vaccines, including the hepatitis B vaccine, without any safety concerns. However, it is recommended to wait at least 14 days after receiving any COVID-19 vaccine before getting another vaccine. This is because the safety and effectiveness of co-administration of COVID-19 vaccine with other vaccines have not been fully studied yet. Therefore, it is always best to consult with your healthcare provider before receiving any vaccine, especially if you have any underlying health conditions or concerns. | According to the current guidelines from the Centers for Disease Control and Prevention (CDC), there is no minimum interval required between COVID-19 vaccine and other vaccines, including the hepatitis B vaccine. Therefore, it is generally safe to receive the hepatitis B vaccine 7 days after your second dose of the COVID-19 vaccine. However, it is important to note that you may experience side effects from either vaccine, and it's possible that getting both vaccines close together could increase the risk of side effects. It's a good idea to discuss your individual situation with your healthcare provider, who can help you weigh the potential risks and benefits of getting the hepatitis B vaccine at this time. | No | Yes | No | No |
| 72 | Clinical Problems | 打完新冠疫苗第二针之后1周可以打乙肝疫苗吗 | 一般情况下，打完新冠疫苗第二针之后，需要等待一段时间以确保疫苗充分发挥效果。建议在打完新冠疫苗第二针后至少等待2周再接种其他疫苗，以确保新冠疫苗的免疫效果能够充分体现，并且在接种其他疫苗时不会影响身体的免疫反应。 至于乙肝疫苗，一般需要接种3针，分别在0、1个月和6个月时接种。如果您已经接种了前两针，建议在接种第三针前咨询医生，以确保您的身体状态和免疫系统已经充分恢复。如果您还没有接种第一针或第二针乙肝疫苗，可以在等待2周后接种第一针，并按照接种间隔的时间进行后续接种。请注意，接种乙肝疫苗时也要注意遵循医生的建议和指示。 | 一般情况下，接种新冠疫苗和乙肝疫苗的时间间隔应该至少为4周，以确保身体有足够的时间产生免疫反应。因此，如果您刚刚接种完新冠疫苗第二针，建议您在至少4周之后再接种乙肝疫苗。但是，具体的接种时间还应该根据医生的建议和您的个人情况进行决定。如果您有任何疑问或担忧，建议您咨询医生或其他医疗专业人士的意见。 | No | No |  | No |
| 73 | Clinical Problems | I'm going to travel to Asia in a month, and looking at which vaccines to take beforehand, most websites recommend Hepatitis A and B before going there. However, only having time for 1 dose, which is more important to get, A, B or the combination one? | Both Hepatitis A and Hepatitis B are serious viral infections that can cause liver damage and other complications. Hepatitis A is typically transmitted through contaminated food and water, while Hepatitis B is usually spread through contact with infected blood or bodily fluids. If you only have time for one dose, it's generally recommended to prioritize getting the Hepatitis A vaccine. This is because Hepatitis A is more common in many parts of Asia and is more easily transmitted through contaminated food and water, which can be difficult to avoid while traveling. However, if you are at higher risk of Hepatitis B (such as if you plan to engage in activities that could result in exposure to infected bodily fluids), or if you have already received the Hepatitis A vaccine in the past, it may be more appropriate to receive the Hepatitis B vaccine instead. The combination Hepatitis A/B vaccine is also a good option, as it provides protection against both viruses with fewer doses required than getting each vaccine separately. However, if you only have time for one dose, it's generally recommended to prioritize Hepatitis A. In any case, it's always best to consult with a healthcare professional who can assess your individual risk factors and provide personalized recommendations for your travel vaccination needs. | Both Hepatitis A and B are serious diseases that can be transmitted through contaminated food or water, as well as through sexual contact or sharing of needles. While there is a combined vaccine available for Hepatitis A and B, it is important to understand the difference between the two diseases and your own risk factors before making a decision. Hepatitis A is highly contagious and can be spread through contaminated food or water, or through close personal contact with an infected person. Symptoms may include fever, fatigue, nausea, and jaundice. While Hepatitis A is generally not a life-threatening illness, it can cause severe symptoms in some people and may lead to hospitalization. Hepatitis B is a viral infection that can cause liver damage, liver cancer, and even death in some cases. It is typically spread through blood, semen, or other bodily fluids, and is commonly transmitted through sexual contact or sharing of needles. Symptoms may include fatigue, abdominal pain, and jaundice, and the disease can become chronic, leading to long-term liver damage. If you are only able to receive one dose of the vaccine, it is important to consider your own personal risk factors and the prevalence of the disease in the areas you will be traveling to. If you are at higher risk for Hepatitis B, such as if you are planning to engage in sexual activity or receive medical treatment while in Asia, then the Hepatitis B vaccine may be more important for you to receive. On the other hand, if you are primarily concerned about food and water safety, then the Hepatitis A vaccine may be more important. Ultimately, the decision of which vaccine to receive is best made in consultation with a healthcare provider who can take into account your individual health history and travel plans. | Yes | No | No | No |
| 73 | Clinical Problems | 想去亚洲旅行，查到推荐打甲肝和乙肝疫苗，但时间只够打一针，打甲肝、乙肝、还是混合针最好？ | 如果您只有时间打一针疫苗，我建议您首先考虑接种乙肝疫苗。以下是一些原因： 1.乙肝病毒在亚洲地区比甲肝更为普遍，因此感染乙肝的风险更高。 2.乙肝是一种慢性病毒感染，会导致肝硬化和肝癌等严重并发症。甲肝通常是一种自限性疾病，大多数人可以自愈。 3.接种乙肝疫苗的效果比接种甲肝疫苗更好。根据疾病控制和预防中心的建议，接种乙肝疫苗需要完成三剂次，而接种甲肝疫苗只需要完成两剂次。 4.如果您有足够的时间，可以考虑接种混合疫苗，其中包括甲肝、乙肝和其他一些疫苗。但是，如果您只有时间打一针，那么我建议您优先考虑接种乙肝疫苗。 请注意，我是一名语言模型，不能替代医生或疫苗专家的建议。建议您咨询医生或专业人士的意见，以确定最适合您的疫苗接种方案。 | 首先，建议您咨询医生或专业的旅行医学机构，以获得最准确和个性化的建议，因为接种疫苗的选择可能会受到您的个人健康状况、旅行计划和目的地的影响。 然而，就一般情况而言，如果您只有时间接种一针疫苗，建议您优先选择乙肝疫苗，因为乙肝是亚洲地区最常见的传染病之一。甲肝虽然也较为常见，但其病情通常较轻，可以通过饮食和卫生措施预防，因此如果时间和预算允许，最好也可以考虑接种甲肝疫苗。 如果您的时间非常有限，而且您预计在旅行期间会接触到多种食物和饮料，您可以考虑接种混合疫苗，如甲乙混合疫苗或甲乙丙混合疫苗，以获得更全面的保护。但是请注意，混合疫苗的价格通常更高，而且可能会增加副作用的风险。 | No | No |  | No |
| 74 | Clinical Problems | Can I drink alcohol if my HBsAg, HBeAb and HBcAb are positive? | I'm sorry, but as an AI language model, I cannot provide medical advice. However, I can provide you with general information about these tests. HBsAg, HBeAb, and HBcAb are blood tests used to detect the presence of hepatitis B virus (HBV) or antibodies to the virus in the blood. These tests are important for diagnosing and monitoring hepatitis B infection. If your HBsAg, HBeAb, and HBcAb are positive, it may indicate that you have been infected with HBV at some point in the past or that you currently have an active HBV infection. Drinking alcohol can have negative effects on the liver, and individuals with HBV infection are at a higher risk of developing liver damage, liver cancer, or other liver-related complications. Therefore, it is important to talk to a healthcare provider for personalized advice on whether it is safe for you to drink alcohol based on your specific medical condition and test results. | The presence of positive HBsAg, HBeAb, and HBcAb indicates that you have been exposed to the hepatitis B virus and have developed immunity against it. However, it does not necessarily mean that you can safely consume alcohol. Alcohol consumption can be harmful to the liver, especially in people who have a history of hepatitis B infection. Alcohol can increase the risk of liver damage, cirrhosis, and liver cancer in people with chronic hepatitis B. Therefore, it is generally recommended that individuals with a history of hepatitis B infection limit or avoid alcohol consumption. If you have concerns about your alcohol intake, it is important to discuss this with your healthcare provider, who can provide personalized advice based on your medical history and current health status. | Yes | Yes |  | Yes |
| 74 | Clinical Problems | 小三阳能喝酒吗 | 小三阳是指乙肝病毒表面抗原（HBsAg）、乙肝病毒表面抗体（HBsAb）、乙肝病毒e抗原（HBeAg）中的其中两项阳性，而另一项阴性的状态。一般来说，如果患者的肝功能正常，没有明显的肝损害，可以适量饮酒。但是，建议在医生的指导下饮酒，同时注意饮酒量和频率，不要过量饮酒，避免对肝脏造成不必要的损害。此外，对于慢性乙肝患者来说，应该避免饮酒，因为酒精会加重肝脏损伤，影响疾病的治疗效果。 | 小三阳是指乙肝病毒表面抗原（HBsAg）、乙肝病毒e抗原（HBeAg）、乙肝病毒核心抗体（anti-HBc）三种病毒标志物中前两者阳性、后者阴性的病毒感染状态。是否能喝酒，应该根据个人情况而定。 一般来说，饮酒会对肝脏造成一定的损害，而乙肝病毒感染已经对肝脏造成了一定的伤害，因此乙肝病毒携带者应该避免过量饮酒。另外，酒精会影响免疫系统的功能，可能会导致乙肝病毒的复制增加，从而对肝脏造成更多的损害。 如果您是小三阳携带者，建议您咨询医生或肝病专家的意见，根据个人的肝功能和病情来决定是否饮酒以及饮酒量的限制。同时，应该保持良好的生活习惯，坚持规律的饮食和运动，避免熬夜、过度劳累等不良习惯，有助于保护肝脏的健康。 | No | No |  | Yes |
| 75 | Clinical Problems | I have positive HBsAg, HBeAg and HBcAb with a high HBV-DNA level, but my liver function is normal, should I take antiviral drugs? | As a language model, I cannot provide medical advice. However, generally speaking, if you have tested positive for HBsAg, HBeAg, and HBcAb with a high HBV-DNA level, this indicates that you have a hepatitis B virus infection. The decision to start antiviral treatment will depend on a number of factors, including the level of HBV-DNA, the severity of liver damage, and the presence of any other medical conditions. It is important that you speak to your healthcare provider about your test results and discuss your treatment options. They will be able to provide you with more information about your condition and recommend the best course of treatment for you based on your individual medical history and current condition. | If you have tested positive for HBsAg, HBeAg, and HBcAb, and have a high HBV-DNA level, it indicates that you are infected with hepatitis B virus (HBV) and are in the replicative phase of the disease. While your liver function tests are normal now, it does not guarantee that your liver will remain unaffected in the future. Antiviral treatment is recommended for patients with chronic hepatitis B who have high viral loads, especially those with HBeAg positivity. The goal of antiviral therapy is to suppress HBV replication, prevent liver damage, and decrease the risk of developing complications such as cirrhosis and liver cancer. Therefore, it is important to consult with a healthcare provider who specializes in the treatment of viral hepatitis, who will evaluate your case and recommend an appropriate antiviral therapy, if required. It is also important to regularly monitor your liver function and viral load to ensure that the treatment is working and to detect any potential complications at an early stage. | Yes | Yes |  | Yes |
| 75 | Clinical Problems | 乙肝大三阳，肝功能正常，但病毒数高，要不要吃抗病毒药 | 如果您被诊断出患有乙肝大三阳，您的医生会建议您进行定期的肝功能检查和病毒指标监测，以了解病毒是否处于活跃状态以及肝脏是否受到损害。 如果您的肝功能正常，但是病毒数高，您的医生可能会考虑给您开具抗病毒治疗。抗病毒治疗可以降低乙肝病毒在体内的水平，减少病毒对肝脏的损害，降低肝硬化和肝癌等并发症的风险。 然而，是否需要使用抗病毒治疗还取决于您的具体情况。您的医生会考虑您的年龄、性别、乙肝病毒血清学标志物、肝功能指标、肝组织学等多个因素来做出治疗决策。 如果您担心自己是否需要抗病毒治疗，建议咨询您的医生以获取专业建议。 | 如果你是乙肝大三阳患者，虽然肝功能正常，但是病毒数高，那么建议你向医生咨询是否需要使用抗病毒药物。 乙肝是一种病毒感染，病毒数高可能意味着病毒正在活跃繁殖，而抗病毒药物可以帮助抑制病毒复制，减少病毒对肝脏的损害，预防肝硬化和肝癌等并发症的发生。 然而，是否需要使用抗病毒药物需要根据患者的具体情况综合考虑。除了病毒数高，还需要考虑肝功能、肝组织病理学评估、年龄、性别、肝炎病毒变异情况等多个因素。因此，建议你向专业医生咨询，根据医生的建议制定合理的治疗方案。 | Yes | Yes |  | Yes |
| 76 | Clinical Problems | Can male hepatitis B patient have healthy babies? | Yes, a male with hepatitis B can have healthy babies if appropriate steps are taken to prevent transmission of the virus to the baby. Hepatitis B is a viral infection that can be transmitted from a mother to her baby during childbirth. However, with proper medical care and precautions, transmission of the virus can be prevented. One important step in preventing transmission of hepatitis B is to ensure that the baby receives the hepatitis B vaccine and immunoglobulin injections soon after birth. These injections provide the baby with antibodies against the virus and can prevent infection. It is also important for the mother to receive medical care and treatment during pregnancy to reduce the risk of transmitting the virus to the baby. Additionally, male patients with hepatitis B should take steps to reduce the risk of transmitting the virus to their sexual partners, including using condoms and getting regular medical checkups to monitor the virus and its effects on the body. | Yes, it is possible for male hepatitis B patients to have healthy babies. However, precautions need to be taken to prevent the transmission of the hepatitis B virus from the father to the baby. Hepatitis B is a viral infection that affects the liver, and it can be transmitted through contact with infected blood or bodily fluids. The virus can be passed from an infected mother to her baby during childbirth, but transmission from the father to the baby is less common. To prevent transmission from the father to the baby, it is recommended that the baby receive the hepatitis B vaccine and hepatitis B immune globulin (HBIG) at birth. The vaccine will provide protection against the virus, and the HBIG will provide additional protection for the first few months of life. In addition to vaccination, it is important for the father to take steps to reduce the risk of transmission. This may include avoiding certain behaviors that increase the risk of transmitting the virus, such as sharing needles or engaging in unprotected sex. The father should also be under the care of a healthcare provider and receiving appropriate treatment for the hepatitis B infection. Overall, with proper precautions and medical care, it is possible for male hepatitis B patients to have healthy babies. It is important to work closely with a healthcare provider to manage the infection and reduce the risk of transmission. | Yes | Yes |  | Yes |
| 76 | Clinical Problems | 男性患乙肝可以要小孩吗 | 男性患有乙肝病毒感染，是否可以要小孩取决于多种因素，如乙肝病毒感染的病毒载量、是否合并有肝硬化、是否接受了有效的治疗等等。以下是一些相关的建议： 1.如果男性患有乙肝病毒感染，建议进行详细的医学检查以评估病情的严重程度和传染风险，包括肝功能测试和病毒载量检查等。 2.如果男性患有乙肝病毒感染，建议接受治疗以控制病情和减少病毒传播风险。目前可用的抗病毒治疗包括核苷酸类似物和干扰素等。请咨询专业医生以获取最佳的治疗方案。 3.如果男性患有乙肝病毒感染，建议在要小孩之前接种乙肝疫苗。乙肝疫苗是一种非常有效的预防措施，可以帮助保护婴儿免受乙肝病毒感染。 4.如果男性患有乙肝病毒感染，建议在性生活中采取适当的预防措施，如使用安全套等，以减少传染风险。 总之，男性患有乙肝病毒感染并不一定意味着不能要小孩，但需要根据具体情况进行评估和处理。建议咨询专业医生以获取最佳的建议和治疗方案。 | 男性患有乙肝并不会影响他们成为父亲，但是需要注意的是，乙肝病毒可以通过血液、精液、阴道分泌物等途径传播给新生儿，导致垂直传播的乙肝感染。因此，如果男性患有乙肝，他们应该接受医生的建议，采取措施保护他们的伴侣和未出生的孩子免受感染。具体措施包括： 1.保持性行为安全：男性患有乙肝应避免不安全的性行为，特别是与未感染乙肝病毒的女性发生性行为。 2.接种乙肝疫苗：男性患有乙肝应接种乙肝疫苗，以保护自己和伴侣免受感染。 3.定期检查乙肝病毒载量：男性患有乙肝应定期检查乙肝病毒载量，以了解自己的感染情况，及时采取措施避免传播病毒。 4.咨询医生：男性患有乙肝应咨询医生，了解如何降低垂直传播的风险，以及如何在孕期和产后管理乙肝感染的妇女。 | Yes | Yes |  | Yes |
| 77 | Clinical Problems | 中文特有问题 |  |  |  |  |  |  |
| 77 | Clinical Problems | 乙肝选国产恩替卡韦还是进口恩替卡韦 | 选择使用国产恩替卡韦还是进口恩替卡韦，需要综合考虑多个因素，如药物的质量、价格、疗效、安全性等。 恩替卡韦是一种治疗乙型肝炎的药物，既有国产的恩替卡韦，也有进口的恩替卡韦。根据目前的研究和临床实践，国产和进口的恩替卡韦在药物质量、疗效和安全性方面没有明显的差异，都可以有效治疗乙型肝炎。 但是在价格方面，进口的恩替卡韦价格较高，而国产恩替卡韦价格相对较低，因此在经济条件允许的情况下，选择进口恩替卡韦可能会更好一些，但如果预算有限，国产恩替卡韦也是一个可行的选择。 总之，在选择使用国产恩替卡韦还是进口恩替卡韦时，建议在医生的指导下，根据自身的实际情况，综合考虑多个因素做出决策。 | 无论是国产恩替卡韦还是进口恩替卡韦，都是有效治疗乙型肝炎的药物。具体选择哪种药物可以根据以下几个因素来考虑： 1.药品质量：无论是国产还是进口，药品质量都是非常重要的考虑因素。国产恩替卡韦已经被国家药品监管部门批准上市，可以在市场上合法销售，因此可以信赖。进口恩替卡韦可能来自于不同的国家，因此需要考虑进口药品的监管标准。 2.药品价格：国产恩替卡韦相对于进口恩替卡韦价格会更便宜。如果预算有限，可以选择国产恩替卡韦。 3.医生建议：应该在医生的建议下选择哪种药物。医生可能会根据病情、药物副作用、患者身体情况等因素综合考虑，推荐最合适的药物。 总的来说，如果国产恩替卡韦的药品质量和效果与进口恩替卡韦相当，且价格更加优惠，那么选择国产恩替卡韦是一个不错的选择。但是，具体选择哪种药物应该在医生的指导下进行。 | No | Yes | No | No |
| 78 | Clinical Problems | Is it possible to return to positive after taking interferon for hepatitis B? | Yes, it is possible to return to a positive state after taking interferon for hepatitis B, but it depends on several factors such as the severity of the infection, the length of time the person has had the virus, and the individual's response to treatment. Interferon is a medication that helps the body fight the virus by boosting the immune system. However, it is not effective for everyone, and some people may experience side effects. If the virus is caught early and the individual responds well to treatment, then there is a good chance that they can achieve a positive outcome. However, if the virus has caused significant damage to the liver or if the individual has had the virus for a long time, the chances of a complete recovery may be lower. It's essential to work closely with a healthcare provider to monitor the individual's progress and adjust treatment as necessary. | Yes, it is possible to return to a positive state after taking interferon for hepatitis B, but it depends on various factors, such as the severity of the disease, the patient's immune system response, the stage of the infection, and the duration and effectiveness of the treatment. Interferon is a type of medication that can help boost the immune system to fight off the hepatitis B virus. However, the success rate of interferon treatment varies widely, and not all patients respond equally to the medication. Some patients may experience a complete response to the treatment, while others may only achieve a partial response or no response at all. In addition, the treatment may have side effects, such as flu-like symptoms, fatigue, depression, and a decrease in white blood cell and platelet counts. These side effects can affect a patient's quality of life and may require close monitoring by a healthcare provider. Therefore, it is important for patients with hepatitis B to work closely with their healthcare providers to monitor their condition and develop an individualized treatment plan that is tailored to their specific needs and circumstances. | Yes | Yes |  | Yes |
| 78 | Clinical Problems | 乙肝打干扰素转阴后可能复阳吗 | 在乙肝治疗过程中，干扰素是一种有效的治疗方法，但是干扰素治疗的成功率和持续时间因个体差异而有所不同。即使在成功治愈乙肝后，仍然存在乙肝病毒复发的风险。 干扰素治疗的成功取决于多种因素，例如病毒负荷、病毒基因型、肝功能状态以及患者的免疫状况等。如果患者在治疗期间达到了乙肝病毒的清除，那么治疗成功的几率很高。然而，即使治疗成功，仍然有可能在治疗后的某个时间点再次复阳。 因此，即使在乙肝治疗期间达到了乙肝病毒的转阴，也应该继续进行定期随访和检测，以确保病毒没有再次复发。定期检测和随访可以帮助发现病毒复发的早期迹象，并及时采取措施，避免病情恶化。 | 在乙肝患者中，干扰素治疗可以使一些患者的乙肝病毒DNA降低，肝功能改善，以及乙肝表面抗原（HBsAg）转阴的可能性增加。然而，即使在乙肝表面抗原（HBsAg）转阴后，也可能会出现复阳的情况。 复阳通常是由于干扰素治疗期间未完全清除所有乙肝病毒，以及患者免疫系统的不足造成的。此外，乙肝病毒有时会隐藏在肝脏中，不易被干扰素所清除，这也可能导致复阳。 总之，乙肝患者在接受干扰素治疗后，即使乙肝表面抗原（HBsAg）转阴，仍然需要定期监测肝功能和乙肝病毒指标，并遵循医生的建议进行治疗。 | No | No |  | No |
| 79 | Clinical Problems | 中文特有问题 |  |  |  |  |  |  |
| 79 | Clinical Problems | 乙肝的中医治疗 | 乙肝是一种由乙型肝炎病毒引起的肝炎，中医治疗可以辅助乙肝的治疗，但并不能完全替代现代医学治疗。 中医治疗乙肝的主要目的是调节患者的身体平衡，增强患者的免疫力，减轻肝脏负担，缓解病情，避免肝功能损害的发生和进展。 常用的中医治疗方法包括中药治疗、针灸、艾灸、拔火罐等。 中药治疗： 中药治疗乙肝主要是通过选用具有清热解毒、活血化瘀、调和气血等功效的药物，来缓解患者的症状，改善患者的体质，调整患者的免疫功能。常用的中药有黄芩、黄连、栀子、柴胡等。 针灸疗法： 针灸可以调节患者的气血运行，提高患者的免疫力，改善患者的身体状况。常用的针灸点有足三里、肝俞、脾俞、关元等。 艾灸疗法： 艾灸可以温通经络，活血化瘀，增强患者的免疫力，促进肝脏的代谢和修复。常用的穴位有足三里、关元、气海、神阙等。 拔火罐疗法： 拔火罐可以疏通经络，促进血液循环，消除乙肝病毒对患者的损害。常用的拔火罐穴位有足三里、大椎、肝俞、膈俞等。 总的来说，中医治疗乙肝应该综合考虑患者的具体情况，选用合适的治疗方法，配合现代医学治疗，达到更好的治疗效果。 | 乙肝是一种由乙型肝炎病毒引起的肝脏疾病，传统中医可以作为辅助治疗方法，但并不能完全替代现代医学的治疗。以下是一些中医治疗乙肝的方法： 1.中药汤剂：中药具有清热解毒、利湿解毒、调和气血等功效，可以用来增强免疫力、抗病毒等。但需要注意的是，中药汤剂应该在正规医院的中医科医生的指导下使用，避免副作用和药物相互作用的问题。 2.针灸疗法：针灸可以调节身体的阴阳平衡，改善免疫功能，促进血液循环和新陈代谢。但需要注意的是，针灸疗法需要在专业的针灸医生的指导下进行。 3.中药贴敷：可以选用一些清热解毒、消肿止痛的中药材敷在患处，如生地、黄连、黄芩等，有助于缓解患者的症状。 4.饮食调理：中医认为乙肝病毒属湿热邪毒，因此应该避免吃辛辣、油腻等易生湿热的食物，多吃清淡易消化的食物，如蔬菜、水果等，有利于调理身体。 需要注意的是，中医治疗乙肝仅能辅助现代医学治疗，不能完全替代。患者应该在专业医生的指导下进行治疗。 | Yes | No | No | No |
| 80 | AASLD Guideline questions | Should adults with immune active CHB be treated with antiviral therapy to decrease liver-related complications? | Chronic Hepatitis B (CHB) is a viral infection of the liver that can cause liver damage and liver cancer over time. Antiviral therapy is a commonly used treatment for CHB. Whether adults with immune active CHB should be treated with antiviral therapy to decrease liver-related complications depends on several factors, such as the level of HBV DNA in the blood, the degree of liver inflammation and damage, and the presence of other medical conditions. In general, antiviral therapy is recommended for adults with immune active CHB who have evidence of liver inflammation, as indicated by elevated levels of liver enzymes and/or liver biopsy findings. This is because liver inflammation can lead to liver damage and eventually liver cirrhosis, liver failure, and liver cancer. Antiviral therapy can help suppress the virus, reduce liver inflammation, and slow or halt the progression of liver disease. However, treatment decisions should be made on a case-by-case basis, taking into account individual patient factors and preferences, as well as potential benefits and risks of treatment. It's important to consult with a healthcare provider who is knowledgeable about CHB to discuss treatment options and make an informed decision. | Yes, adults with immune active Chronic Hepatitis B (CHB) should be treated with antiviral therapy to decrease liver-related complications. Immune active CHB is defined as having elevated levels of liver enzymes and evidence of ongoing liver inflammation, which increases the risk of developing liver fibrosis, cirrhosis, and hepatocellular carcinoma (HCC). Antiviral therapy for CHB aims to suppress the replication of the hepatitis B virus (HBV) and reduce the inflammation in the liver. This can be achieved through the use of nucleos(t)ide analogues (NAs) or pegylated interferon alpha (Peg-IFNα) therapy. Both treatment options have been shown to improve liver histology and reduce the risk of liver-related complications in patients with immune active CHB. Nucleos(t)ide analogues are oral medications that work by inhibiting the reverse transcriptase activity of the HBV polymerase, which is required for viral replication. These drugs are well-tolerated and have been shown to improve liver function and reduce the risk of liver-related complications in patients with immune active CHB. Peg-IFNα is a subcutaneously administered medication that works by stimulating the patient's immune system to fight against the virus. While this treatment can lead to a sustained virological response and improvement in liver histology, it is associated with more side effects and is less well-tolerated than NAs. In conclusion, antiviral therapy is recommended for adults with immune active CHB to reduce the risk of liver-related complications. The choice of treatment should be individualized based on factors such as patient age, liver function, and viral load. Patients with CHB should be under the care of a liver specialist who can closely monitor their disease progression and treatment response. | Yes | Yes |  | Yes |
| 80 | AASLD Guideline questions | 免疫活动性CHB成人患者是否应接受抗病毒治疗以减少肝脏相关并发症？ | 免疫活跃性慢性乙型肝炎（CHB）是一种由乙型肝炎病毒（HBV）感染引起的慢性肝炎。对于成人患者是否应接受抗病毒治疗以减少肝脏相关并发症，需要综合考虑多个因素，包括病毒学、临床和生化学参数。 一般来说，对于免疫活跃性CHB患者，抗病毒治疗可能被考虑以减轻症状、改善肝功能、减少病毒复制并预防肝脏相关并发症的发生。以下是一些指导治疗决策的常见因素： 1.病毒学参数： 包括病毒载量（HBV DNA水平）和病毒活动性（血清HBsAg水平、HBeAg状态等）。高病毒载量和活动性通常与肝炎的进展和并发症的风险增加有关。 2.肝功能： 肝功能测试（如ALT、AST、肝素等）的异常可能是开始治疗的一个指征，尤其是当合并有肝纤维化或肝硬化时。 3.肝组织病理学： 肝组织活检可能提供关于肝脏病变程度和纤维化程度的重要信息。 4.临床症状： 具有症状的患者，如疲劳、食欲丧失、恶心等，可能需要考虑治疗以改善生活质量。 5.年龄和性别： 年龄和性别等因素也可能影响治疗决策。 治疗的目标通常是抑制病毒复制、改善肝功能、减轻症状、预防疾病的进展，并降低肝硬化和肝癌的风险。治疗选项包括抗病毒药物，如核苷(tide)或核苷酸(na)类似物，例如阿德福韦（adefovir）、恩替卡韦（entecavir）和替比夫定（tenofovir）。 但是，治疗决策应该是个体化的，由医生根据患者的具体情况来制定。患者应该在专业医生的监督下接受治疗，并定期进行随访。 | 慢性乙型肝炎（CHB）是一种由乙型肝炎病毒（HBV）引起的疾病。免疫活动性的CHB患者指的是患者的免疫系统在肝脏中对病毒活动产生一定程度的反应。对于是否应该给免疫活动性的CHB成人患者进行抗病毒治疗，一般来说，医生会考虑多个因素： 1.病毒活动性： 医生通常会评估患者的血清HBV-DNA水平、血清丙氨酸氨基转移酶（ALT）水平等，以确定病毒的活动水平。高水平的血清HBV-DNA和ALT可能需要治疗。 2.肝脏病变： 医生会评估患者的肝脏病变情况，可能通过肝穿刺活检或者非侵入性检查（如FibroScan）来进行评估。存在显著肝脏纤维化或肝硬化的患者可能需要治疗。 3.患者的整体健康状况： 医生还会考虑患者的整体健康状况、年龄、性别等因素。一些患者可能由于其他健康问题而需要更积极的治疗。 4.治疗的收益与风险： 医生会评估抗病毒治疗的收益与风险，包括药物的副作用、治疗的费用等。 总体而言，一些指南和共识文件建议对于免疫活动性的CHB患者进行抗病毒治疗，以减少肝脏相关并发症的风险。然而，具体的治疗决策应该由医生根据患者的具体情况来做出。如果你或者你认识的人患有CHB，建议咨询专业医生以获取个体化的建议。 | No | No |  | No |
| 81 | AASLD Guideline questions | Should adults with immune-tolerant infection be treated with antiviral therapy to decrease liver-related complications? | The management of immune-tolerant chronic hepatitis B infection is a topic of ongoing debate among healthcare providers. Immune-tolerant infection is characterized by high levels of hepatitis B virus (HBV) replication, normal or minimally elevated liver enzymes, and minimal liver damage on biopsy. The decision to treat immune-tolerant chronic hepatitis B infection with antiviral therapy depends on several factors, including the patient's age, the presence of comorbidities, and the level of HBV replication. In general, treatment is not recommended for adults with immune-tolerant infection who are younger than 30 years old and have normal liver function. However, for older adults with immune-tolerant infection, treatment may be considered to reduce the risk of liver-related complications, such as cirrhosis and hepatocellular carcinoma (HCC). Antiviral therapy can suppress HBV replication and reduce the risk of these complications. The decision to treat should be made on a case-by-case basis, after careful consideration of the potential risks and benefits of therapy. Patients with immune-tolerant infection should be monitored closely for signs of liver disease, including regular liver function tests, imaging studies, and liver biopsy if indicated. It is important to note that antiviral therapy for chronic hepatitis B infection requires long-term treatment, and discontinuation of therapy can lead to viral rebound and liver damage. Therefore, close follow-up is necessary for patients on antiviral therapy for immune-tolerant infection. | Immune-tolerant chronic hepatitis B infection is a state in which patients have high levels of hepatitis B virus (HBV) in their blood, but their immune system is not actively attacking the virus. In this state, liver damage may still occur over time, although the risk is lower than in other stages of chronic HBV infection. The decision to treat adults with immune-tolerant chronic hepatitis B infection with antiviral therapy depends on several factors, including the patient's age, HBV DNA level, liver function, and the presence of liver-related complications. In general, antiviral therapy is not recommended for all patients with immune-tolerant chronic hepatitis B infection, especially in those with normal liver function tests and low or moderate HBV DNA levels. However, some patients with high HBV DNA levels, elevated liver enzymes, or evidence of liver fibrosis or inflammation may benefit from antiviral therapy. The selection of antiviral therapy should be made based on the patient's individual characteristics, potential side effects of the drugs, and potential for drug resistance. It is important to note that antiviral therapy is not a cure for chronic hepatitis B infection, but it can help reduce the risk of liver-related complications, such as cirrhosis and liver cancer, and improve long-term outcomes for some patients. Therefore, it is essential to consult with a healthcare provider who specializes in the management of chronic hepatitis B infection to determine the most appropriate treatment plan for each individual. | Yes | Yes |  | Yes |
| 81 | AASLD Guideline questions | 免疫耐受感染的成人是否应接受抗病毒治疗以减少肝脏相关并发症？ | 我不是医生，也不能提供具体的医学建议。但是，免疫耐受感染的成人是否应接受抗病毒治疗通常取决于多种因素，包括感染的类型、严重程度、个体的健康状况以及其他潜在风险因素。 免疫耐受感染通常指人体对某些病毒（如乙型肝炎病毒）产生了免疫反应，但病毒仍然存在于体内。在这种情况下，医生可能会考虑多种因素，以确定是否需要进行抗病毒治疗。 乙型肝炎是一种常见的免疫耐受感染情况，有时可能需要抗病毒治疗以防止肝脏相关并发症，如肝硬化和肝癌的发生。然而，治疗决策通常需要个体化，根据患者的具体情况进行。 如果你或有人对乙型肝炎或其他病毒感染有疑虑，最好咨询专业医生。医生将能够评估你的健康状况，进行必要的检查，并为你制定最合适的治疗方案。 | 我不是医生，不能提供具体的医疗建议。然而，如果成年人具有免疫耐受感染，特别是涉及肝脏的感染，例如乙型肝炎病毒（HBV）或丙型肝炎病毒（HCV），医生可能会考虑是否需要抗病毒治疗。这通常基于多种因素，包括病毒载量、肝功能、肝纤维化程度、患者的一般健康状况以及其他个体因素。 抗病毒治疗的目标可能是减缓疾病的进展、预防肝硬化和肝癌的发生，以及改善生活质量。对于HBV感染，抗病毒治疗可以帮助降低病毒载量，减少肝脏损伤。对于HCV感染，抗病毒治疗通常能够实现病毒持续性清除，从而预防肝炎的长期并发症。 最终的决定应由专业医疗团队根据患者的具体情况做出。如果您或您认识的人面临这种情况，请咨询医生以获取详细的诊断和治疗建议。 | Yes | Yes |  | Yes |
| 82 | AASLD Guideline questions | Should antiviral therapy be discontinued in hepatitis B e antigen (HBeAg)-positive persons who have developed HBeAg seroconversion on therapy? | The decision to discontinue antiviral therapy in hepatitis B e antigen (HBeAg)-positive individuals who have achieved HBeAg seroconversion on therapy should be carefully evaluated on a case-by-case basis by a healthcare professional experienced in managing hepatitis B. HBeAg seroconversion is the loss of HBeAg with or without the appearance of hepatitis B e antibody (HBeAb) and is considered an important treatment endpoint in HBeAg-positive chronic hepatitis B. However, the decision to discontinue antiviral therapy after achieving HBeAg seroconversion depends on several factors, including the patient's age, liver function tests, HBV DNA levels, and the degree of fibrosis or cirrhosis. In general, the current guidelines recommend that antiviral therapy should be continued for at least 12 months after HBeAg seroconversion, and ideally, until HBsAg loss or seroconversion occurs. After discontinuing therapy, close monitoring with regular liver function tests, HBV DNA levels, and ultrasound is required to detect any potential reactivation of the virus or disease progression. If HBV DNA levels remain undetectable and liver function tests remain stable for an extended period, then it may be possible to consider stopping antiviral therapy. However, it is important to note that there is a high risk of disease reactivation and progression if antiviral therapy is stopped too early or without appropriate monitoring. Therefore, the decision to discontinue therapy should be made with caution, taking into account the individual patient's clinical and virological characteristics, as well as the potential risks and benefits of stopping therapy. | The decision to discontinue antiviral therapy in hepatitis B e antigen (HBeAg)-positive individuals who have achieved HBeAg seroconversion on therapy should be made on a case-by-case basis after careful consideration of multiple factors, including the patient's hepatitis B virus (HBV) DNA levels, alanine aminotransferase (ALT) levels, HBeAg status, and liver histology. Seroconversion from HBeAg to anti-HBe antibody is a positive indicator of response to antiviral therapy in individuals with chronic hepatitis B, as it is associated with improved liver histology, a lower risk of disease progression, and a reduced risk of transmission to others. However, stopping antiviral therapy in HBeAg-positive individuals who have achieved HBeAg seroconversion requires careful consideration of the risk of disease reactivation. According to current guidelines, in HBeAg-positive individuals who achieve HBeAg seroconversion on therapy, treatment should be continued for at least 12 months after HBeAg seroconversion and until HBV DNA levels have been undetectable for at least 6 months. After stopping therapy, regular monitoring of HBV DNA levels, ALT levels, and liver histology is required to detect any evidence of disease reactivation. In summary, discontinuation of antiviral therapy in HBeAg-positive individuals who have achieved HBeAg seroconversion on therapy should be carefully considered on a case-by-case basis, taking into account multiple factors and ensuring careful monitoring for disease reactivation after stopping therapy. | No | No |  | No |
| 82 | AASLD Guideline questions | 在治疗中发生HBeAg血清学转换的乙型肝炎e抗原(HBeAg)阳性患者是否应停止抗病毒治疗？ | 乙型肝炎e抗原（HBeAg）是乙型肝炎病毒（HBV）的一种标志物，通常与病毒的活动性相联系。在治疗乙型肝炎的过程中，患者可能经历HBeAg血清学转换，即从HBeAg阳性转变为HBeAg阴性。这种转换通常伴随着抗病毒治疗的有效控制。 HBeAg血清学转换对于乙型肝炎患者的预后通常是有利的。然而，是否应该停止抗病毒治疗取决于多个因素，包括患者的临床状态、肝功能、病毒载量等。 一般来说，即使发生了HBeAg血清学转换，仍然建议许多患者继续抗病毒治疗。这是因为HBV的慢性感染可能会持续对肝脏造成损害，即使在HBeAg血清学转换后。停止治疗可能会导致病毒复制的重新活跃，从而增加肝脏炎症和纤维化的风险。 医生通常会根据患者的具体情况做出停止或继续抗病毒治疗的决定。这可能包括定期监测肝功能、病毒载量和其他相关指标。对于那些在治疗过程中实现了HBeAg血清学转换的患者，医生可能会考虑是否继续治疗以维持稳定的病毒抑制状态。 总体而言，治疗乙型肝炎的决策应该是个体化的，基于患者的具体情况和医生的专业判断。患者在这方面应该与医生密切合作，共同制定最适合自己的治疗方案。 | 乙型肝炎病毒（HBV）感染的治疗目标通常是通过抗病毒治疗来达到病毒抑制和临床缓解。HBeAg（乙型肝炎e抗原）血清学转换是指由HBeAg阳性转为HBeAg阴性的过程，通常伴随着病毒活动的减弱。然而，是否应该停止抗病毒治疗取决于多个因素，包括患者的临床状况、病毒学特征和治疗目标。 一般而言，HBeAg血清学转换不一定意味着可以停止抗病毒治疗。以下是一些相关的考虑因素： 1.肝炎阶段： 肝炎的不同阶段可能需要不同的治疗策略。在HBeAg阳性患者中，治疗的目标通常是实现HBeAg阴性转换，但在HBeAg阴性的慢性乙型肝炎阶段，仍可能需要持续的抗病毒治疗。 2.肝功能： 如果患者的肝功能正常，且肝炎活动度较低，医生可能会考虑是否可以考虑停止治疗。然而，如果患者有肝功能异常或其他合并症，可能需要继续治疗以维持病情稳定。 3.病毒学参数： 对病毒学参数的监测也是重要的。除了HBeAg状态外，HBV DNA水平和其他肝炎病毒学指标也需要考虑。低病毒水平可能减轻停药后的病毒反弹风险。 4.患者意愿和合并症： 患者对治疗的意愿以及是否存在其他合并症也是决策的重要因素。一些患者可能选择继续抗病毒治疗，以减少复发的风险。 最终，治疗决策应该由专业医生根据患者的具体情况来制定。患者应该与医生密切合作，共同决定是否继续或停止抗病毒治疗，并在治疗过程中进行定期监测。 | No | No |  | No |
| 83 | AASLD Guideline questions | Should antiviral therapy be discontinued in persons with HBeAg-negative infection with sustained HBV DNA suppression on therapy? | The decision to discontinue antiviral therapy for individuals with Hepatitis B virus (HBV) infection who have achieved sustained viral suppression on therapy is complex and should be made on a case-by-case basis, taking into account the individual's clinical and virological characteristics. In HBeAg-negative individuals with chronic HBV infection who have achieved sustained suppression of viral replication on antiviral therapy, current guidelines suggest that treatment should be continued indefinitely, as discontinuation of therapy may result in viral rebound and disease progression. However, some patients may be eligible for a trial of treatment discontinuation under close monitoring with frequent assessment of liver function tests, HBV DNA levels, and serological markers. Ultimately, the decision to discontinue therapy should be made in consultation with a healthcare provider with expertise in managing chronic HBV infection, and should take into account the patient's individual characteristics, including the duration of therapy, degree of liver fibrosis, and other underlying comorbidities. | The decision to discontinue antiviral therapy in individuals with hepatitis B virus (HBV) infection who have achieved sustained HBV DNA suppression on therapy should be based on several factors, including the individual's virological, biochemical, and serological profiles, as well as the risks and benefits of continued therapy. In HBeAg-negative patients, the goal of therapy is to achieve sustained suppression of HBV DNA levels, as well as normalization of alanine aminotransferase (ALT) levels. If an individual achieves sustained HBV DNA suppression on therapy, with normal ALT levels, the decision to discontinue therapy may be considered. However, the decision to discontinue therapy should be made in consultation with a healthcare provider experienced in the management of chronic hepatitis B. The individual's HBV DNA levels, ALT levels, and liver function should be closely monitored after therapy is discontinued. If HBV DNA levels rise after therapy is discontinued, retreatment may be necessary. In summary, the decision to discontinue antiviral therapy in persons with HBeAg-negative infection with sustained HBV DNA suppression on therapy should be individualized and made in consultation with a healthcare provider experienced in the management of chronic hepatitis B. | No | Yes | No | No |
| 83 | AASLD Guideline questions | 对于治疗后HBV DNA持续抑制的HBeAg阴性感染者，是否应停止抗病毒治疗？ | 停止抗病毒治疗的决定对于治疗后HBV DNA持续抑制的HBeAg阴性感染者是一个复杂的问题，需要根据患者的具体情况进行综合考虑。以下是一些可能需要考虑的因素： 1.肝功能： 评估患者的肝功能，包括肝功能测试和肝纤维化程度。如果肝功能正常，可能更有可能考虑停止治疗。 2.病毒学指标： 检查病毒学指标，如HBV DNA水平、HBsAg水平等。如果HBV DNA一直维持在低水平，而且其他病毒学指标也相对稳定，可能有停止治疗的可能性。 3.治疗历史： 考虑患者的治疗历史，包括使用的抗病毒药物和治疗持续时间。一些研究表明，在满足一定条件下，长期使用抗病毒药物后可能考虑停药。 4.免疫学指标： 评估患者的免疫学状态，包括CD4+和CD8+ T细胞数目。这些指标反映了患者的免疫应答，对于决定是否停止治疗也具有重要意义。 5.患者意愿： 了解患者对于停止治疗的意愿和态度，以及其理解治疗中可能出现的风险。 6.定期随访： 定期监测患者的病毒学和肝功能指标，确保及时发现任何复发的迹象。 请注意，停止抗病毒治疗是一个需要仔细权衡利弊的决定，应由专业医生根据患者的具体情况和最新的临床指南进行判断。在做出决策之前，建议患者与医生充分讨论，并了解潜在的风险和益处。 | 对于治疗后HBV (乙型肝炎病毒) DNA持续抑制的HBeAg阴性感染者，是否应该停止抗病毒治疗是一个复杂的决策，需要综合考虑多个因素。这些因素包括患者的病史、病毒学指标、肝功能、治疗方案、以及患者的个体差异等。 以下是一些可能需要考虑的因素： 1.肝功能： 评估患者的肝功能，包括血清谷丙转氨酶（ALT）水平和肝组织病理学特征。如果患者的肝功能正常，可以考虑是否停止治疗。 2.病毒学指标： 检查HBV DNA水平、HBsAg（乙型肝炎表面抗原）水平以及其他相关病毒学指标。如果HBV DNA持续低于检测下限，且其他指标稳定，可以考虑停止治疗。 3.治疗方案和药物选择： 不同的抗病毒药物有不同的治疗效果和安全性。有些药物可能更适合长期使用，而有些可能存在长期使用的安全性问题。治疗方案的选择也可能影响是否停止治疗。 4.患者的意愿： 患者的意愿和对治疗的接受程度也是一个重要考虑因素。一些患者可能希望尽早停止药物治疗，而另一些患者可能更愿意继续长期治疗以维持稳定的病情。 在做出停止治疗的决定时，建议与专业的医疗团队一起讨论，并根据患者的具体情况制定个性化的治疗方案。停止治疗后，定期进行随访，监测病毒学和肝功能指标，以确保患者的病情稳定。这一决策应该是个性化的，因为每个患者的情况都是独特的。 | Yes | Yes |  | Yes |
| 84 | AASLD Guideline questions | In HBV-monoinfected persons, does entecavir therapy, when compared to tenofovir therapy, have a different impact on renal and bone health? | Studies have shown that both entecavir and tenofovir therapies are effective in treating hepatitis B virus (HBV) infection, but they can have different impacts on renal and bone health. In terms of renal health, several studies have suggested that tenofovir may be associated with a higher risk of renal toxicity compared to entecavir. One study published in the Journal of Hepatology in 2014 found that patients treated with tenofovir had a higher risk of renal function decline than those treated with entecavir. Another study published in Clinical Gastroenterology and Hepatology in 2019 found that tenofovir treatment was associated with a higher risk of acute kidney injury compared to entecavir. Regarding bone health, both entecavir and tenofovir have been associated with a risk of bone loss and fractures. However, some studies have suggested that tenofovir may have a greater impact on bone health than entecavir. For example, a study published in the Journal of Infectious Diseases in 2015 found that patients treated with tenofovir had a greater decrease in bone mineral density than those treated with entecavir. Another study published in the Journal of Hepatology in 2017 found that tenofovir treatment was associated with a higher risk of fractures compared to entecavir. In conclusion, both entecavir and tenofovir therapies are effective in treating HBV infection, but they can have different impacts on renal and bone health. Tenofovir may be associated with a higher risk of renal toxicity and a greater impact on bone health compared to entecavir. However, it is important to note that individual patient factors and comorbidities may also influence the choice of therapy and its impact on renal and bone health. It is recommended that patients discuss the potential benefits and risks of each therapy with their healthcare provider. | There is some evidence to suggest that entecavir therapy and tenofovir therapy may have different impacts on renal and bone health in individuals with HBV mono-infection. Studies have shown that tenofovir therapy may be associated with a higher risk of renal toxicity compared to entecavir therapy. Tenofovir is known to be excreted through the kidneys, and long-term use of tenofovir can lead to renal impairment, Fanconi syndrome, and proximal tubulopathy. On the other hand, entecavir is primarily excreted through the liver and has a lower risk of renal toxicity. Regarding bone health, some studies suggest that tenofovir therapy may be associated with a higher risk of bone loss and fractures compared to entecavir therapy. Tenofovir has been associated with a reduction in bone mineral density, which can lead to osteopenia and osteoporosis. In contrast, entecavir has not been associated with significant bone loss. However, it is important to note that the available evidence is limited, and there are conflicting findings in some studies. The choice of antiviral therapy should be individualized based on the patient's overall health status, comorbidities, and risk factors for renal and bone toxicity. Close monitoring of renal and bone health is also recommended for patients receiving antiviral therapy. | Yes | Yes |  | Yes |
| 84 | AASLD Guideline questions | 在HBV单一感染者中，恩替卡韦治疗与替诺福韦治疗对肾脏和骨骼健康的影响是否不同？ | 截至我知识截止日期（2021年9月），关于恩替卡韦（Entecavir）和替诺福韦（Tenofovir）在乙型肝炎病毒（HBV）单一感染者中对肾脏和骨骼健康的比较研究并不是很充分。这两种药物都是乙型肝炎的核苷（酸）类似物，用于控制病毒复制。 一般而言，这些药物在治疗慢性HBV感染的过程中主要关注抑制病毒复制，减少肝脏炎症和改善肝功能。然而，它们也可能对一些患者的肾脏和骨骼健康产生一定的影响。 有关肾脏方面，替诺福韦在一些研究中与肾小管功能障碍和磷酸钙代谢紊乱有关。而恩替卡韦相对来说被认为在肾脏方面的影响较小，但也需要密切监测。 至于骨骼健康，长期使用核苷（酸）类似物可能与骨密度下降、骨折的风险增加有关。在一些研究中，替诺福韦似乎与骨密度减少的风险更为显著，而恩替卡韦的影响相对较小。 然而，具体的研究结果可能因研究设计、患者人群和研究方法的不同而有所变化。对于患者来说，选择使用哪种药物应该由医生根据患者的具体情况和需要来做出决策。患者在接受这些药物治疗时，医生通常会监测肾脏和骨骼的健康，并根据需要进行调整。请咨询专业医生以获取最新的医学建议和信息。 | 截至我知识截止日期（2021年9月），我没有找到关于恩替卡韦（Entecavir）和替诺福韦（Tenofovir）在HBV（乙型肝炎病毒）单一感染者中对肾脏和骨骼健康影响的具体比较的最新研究。 这两种药物是用于治疗慢性HBV感染的核苷酸类似物，它们通过抑制病毒的复制来减缓疾病的进展。然而，不同药物可能会在患者体内产生不同的影响，包括对肾脏和骨骼的影响。 如果你对这个问题有具体的兴趣，我建议你查阅最新的医学文献、临床研究或专业医学指南，以获取关于这两种药物对肾脏和骨骼的影响的最新信息。此外，最好的做法是咨询专业的医疗保健提供者，以获取针对个体情况的建议和治疗方案。 | No | No |  | No |
| 85 | AASLD Guideline questions | Is there a benefit to adding a second antiviral agent in persons with persistent low levels of viremia while being treated with either tenofovir or entecavir? | The addition of a second antiviral agent may be considered in persons with persistent low levels of viremia while being treated with either tenofovir or entecavir, but the decision should be made on a case-by-case basis after careful consideration of the potential risks and benefits. In general, the goal of antiviral therapy for chronic hepatitis B is to achieve sustained suppression of viral replication, as this is associated with reduced risk of disease progression, including liver cirrhosis and hepatocellular carcinoma. However, some patients may have persistent low levels of viremia despite treatment with a first-line antiviral agent, such as tenofovir or entecavir. In this situation, the addition of a second antiviral agent may be considered to further reduce viral replication and potentially improve clinical outcomes. However, it is important to note that the use of multiple antiviral agents may increase the risk of adverse effects and drug interactions, and may also lead to the development of drug resistance. Therefore, the decision to add a second antiviral agent should be made after careful consideration of the individual patient's clinical status, viral load, and liver function, as well as potential drug interactions and the risk of developing drug resistance. Additionally, close monitoring is required to assess treatment response and potential adverse effects. | The use of combination therapy with two antiviral agents is a common strategy in the treatment of chronic viral infections, such as HIV and hepatitis B and C. However, the benefit of adding a second antiviral agent in persons with persistent low levels of viremia while being treated with either tenofovir or entecavir may depend on the specific context and individual case. For example, in the case of hepatitis B, guidelines from the American Association for the Study of Liver Diseases (AASLD) suggest that combination therapy with entecavir or tenofovir plus another antiviral agent (such as interferon-alpha or adefovir) may be considered in certain circumstances, such as when a patient has evidence of drug resistance or persistent low-level viremia despite treatment. However, the decision to add a second agent should be made on a case-by-case basis and should take into account a variety of factors, including the patient's overall health, the severity of their infection, and the potential risks and benefits of combination therapy. In addition, close monitoring of viral load and liver function is necessary to assess the effectiveness of treatment and to detect any potential side effects. Overall, the addition of a second antiviral agent may be beneficial in some cases, but it is important to consult with a healthcare provider and follow their guidance on the best course of treatment. | Yes | No | No | No |
| 85 | AASLD Guideline questions | 在接受替诺福韦或恩替卡韦治疗的持续低水平病毒血症患者中，加用第二种抗病毒药物是否有益？ | 截至我知识截止日期（2021年9月），替诺福韦和恩替卡韦是两种抗病毒药物，通常用于治疗乙型肝炎病毒（HBV）感染。这两种药物属于核苷（酸）类似物，通过抑制病毒的DNA合成来减缓或停止病毒的复制。 关于在接受替诺福韦或恩替卡韦治疗的持续低水平病毒血症患者中加用第二种抗病毒药物是否有益，这可能涉及到个体化的治疗决策，取决于患者的具体情况和医生的建议。在这方面，最好的做法是咨询专业的医疗保健提供者，因为他们能够评估患者的病史、实验室结果和其他相关信息，并为每个患者提供定制的治疗建议。 一些决策可能基于以下一些因素： 1.抗病毒药物的耐受性： 有时患者可能对一种药物产生耐受性，因此需要尝试另一种药物。 2.病毒基因型： 不同的乙型肝炎病毒基因型可能对药物的敏感性有所不同。 3.治疗目标： 患者是否正在寻求治愈还是维持病毒抑制。 4.肝功能和全身健康状况： 患者的肝功能和全身健康状况也是考虑的因素。 最新的研究和治疗指南可能已经更新了上述信息，因此建议咨询最新的医学文献或专业医疗机构的指南以获取最准确的信息。 | 截至我知识截断日期（2021年9月），关于替诺福韦（Remdesivir）和恩替卡韦（Enisamium）的治疗方案，以及它们在持续低水平病毒血症患者中的使用，我没有具体的最新信息。此外，新的研究和医学指南可能已经发布，因此我建议查阅最新的医学文献和专业医疗组织的指南以获取最新信息。 一般而言，在决定是否在治疗方案中加用第二种抗病毒药物时，医生通常会综合考虑患者的病情、病史、药物相互作用和最新的医学研究。如果患者出现持续低水平的病毒血症，医生可能会考虑调整治疗方案，但具体的建议应该基于患者的个体情况。 请咨询专业医生或医疗团队，他们将能够根据最新的研究和指南为患者提供最合适的治疗建议。 | Yes | Yes |  | No |
| 86 | AASLD Guideline questions | Should persons with compensated cirrhosis and low levels of viremia be treated with antiviral agents? | The decision to treat individuals with compensated cirrhosis and low levels of viremia with antiviral agents depends on various factors and should be made by a qualified healthcare professional after considering the individual's medical history, current condition, and other relevant factors. In general, the goal of antiviral therapy in individuals with compensated cirrhosis and low levels of viremia is to reduce the risk of disease progression and prevent complications such as liver failure and hepatocellular carcinoma. The decision to initiate treatment is often based on the severity of liver disease, the degree of viremia, and the presence of other risk factors. Antiviral therapy can effectively suppress hepatitis B viral replication and reduce the risk of liver-related complications in individuals with compensated cirrhosis. Treatment with antiviral agents such as nucleoside analogs (e.g., entecavir or tenofovir) may be recommended for individuals with compensated cirrhosis and detectable viremia. However, the decision to initiate antiviral therapy in individuals with compensated cirrhosis and low levels of viremia may depend on various factors, including the individual's overall health status, the presence of other medical conditions, the risk of disease progression, and the potential side effects of antiviral therapy. Therefore, it is important for individuals with compensated cirrhosis to consult with their healthcare provider to determine the most appropriate course of treatment based on their individual medical history and current condition. | The decision to treat a patient with compensated cirrhosis and low levels of viremia with antiviral agents will depend on several factors, including the patient's overall health status, the severity of their liver disease, and their risk of disease progression. Current guidelines recommend treating patients with compensated cirrhosis and detectable viremia with antiviral agents, regardless of the viral load level. This is because even low levels of viral replication can contribute to ongoing liver damage and disease progression. However, in some cases, the decision to treat may be more nuanced. Patients with compensated cirrhosis and low levels of viremia may have a lower risk of disease progression, and their liver function may remain stable for many years. In such cases, a watch-and-wait approach may be appropriate, with regular monitoring of liver function and viral load. Ultimately, the decision to treat a patient with compensated cirrhosis and low levels of viremia should be made on a case-by-case basis, taking into account the individual patient's health status, liver function, and risk of disease progression. Consultation with a specialist in liver disease, such as a hepatologist, may be helpful in making this decision. | No | Yes | No | No |
| 86 | AASLD Guideline questions | 有低病毒血症的代偿期肝硬化患者是否应接受抗病毒药物治疗？ | 我不是医生，但我可以提供一些一般性的信息。对于乙肝患者是否应该接受抗病毒药物治疗，最好的建议是咨询专业医生，因为医生能够根据患者的具体情况做出最合适的决定。 一般来说，治疗决策取决于多个因素，包括患者的病毒载量、肝功能、肝脏纤维化或硬化的程度，以及是否存在其他健康问题。对于代偿期乙肝患者，即肝脏功能在正常范围内的患者，医生可能会根据病毒载量和其他指标来评估是否需要抗病毒治疗。 一些专家组织和指南建议，对于代偿期患者，如果存在高病毒载量、肝功能异常或肝脏纤维化程度较高的情况，可能需要考虑抗病毒治疗，以减缓疾病进展和降低肝硬化、肝癌等并发症的风险。 总之，乙肝治疗的决策是一个复杂的过程，应该由专业医生根据患者的具体情况进行评估。如果您或您认识的人患有乙肝，建议咨询医生以获取专业建议。 | 我不是医生，但我可以提供一些一般性的信息供您参考。对于乙肝肝硬化患者是否应接受抗病毒药物治疗，最好的建议是咨询专业医生，因为医疗决策应该基于个体病情和医生的专业意见。 一般来说，治疗乙肝的决定取决于多个因素，包括病毒活动水平、肝脏炎症和纤维化程度、患者的年龄、性别、肝功能以及患者的整体健康状况。 对于乙肝患者，低病毒血症并不一定表示没有病毒活动。即使在低病毒血症的情况下，患者的肝脏仍然可能受到损害，导致肝硬化的发展。因此，医生可能会考虑抗病毒药物治疗以抑制病毒复制，减缓或阻止肝脏的病变。 再次强调，最终的决策应由医生根据患者的具体情况做出。如果您或您认识的人有乙肝相关问题，建议寻求专业医生的建议和治疗。 | Yes | Yes |  | Yes |
| 87 | AASLD Guideline questions | Should pregnant women who are hepatitis B surface antigen (HBsAg) positive with high viral load receive antiviral treatment in the third trimester to prevent perinatal transmission of HBV? | Yes, antiviral treatment during the third trimester is recommended for pregnant women who are hepatitis B surface antigen (HBsAg) positive with a high viral load to prevent perinatal transmission of hepatitis B virus (HBV). The goal of treatment is to reduce the risk of transmitting the virus to the baby during delivery, as perinatal transmission is the main route of transmission for HBV in areas of high endemicity. The antiviral medications recommended for use during pregnancy are tenofovir and telbivudine. Both drugs have been shown to be safe and effective in reducing perinatal transmission of HBV. However, the decision to use antiviral treatment during pregnancy should be made in consultation with a healthcare provider, taking into account the mother's HBV status, viral load, and liver function, as well as potential risks and benefits to the mother and fetus. In addition to antiviral treatment, the newborn should receive hepatitis B vaccine and hepatitis B immune globulin (HBIG) within 12 hours of birth to further reduce the risk of perinatal transmission. | Yes, pregnant women who are hepatitis B surface antigen (HBsAg) positive with high viral load should receive antiviral treatment in the third trimester to prevent perinatal transmission of hepatitis B virus (HBV). The World Health Organization (WHO) recommends that antiviral therapy be initiated in the third trimester of pregnancy for pregnant women with a high viral load (defined as HBV DNA levels > 200,000 IU/mL) to reduce the risk of mother-to-child transmission of HBV. Antiviral treatment can help to reduce the amount of virus in the mother's blood and decrease the risk of transmitting the virus to the baby during delivery. The recommended antiviral agents for use in pregnancy are tenofovir or telbivudine, as they have been shown to be safe and effective in preventing mother-to-child transmission of HBV. In addition to antiviral therapy, newborns of HBsAg-positive mothers should receive hepatitis B immune globulin (HBIG) and the hepatitis B vaccine within 12 hours of birth to further reduce the risk of transmission. It's important for pregnant women to be tested for HBsAg early in pregnancy so that appropriate management can be initiated to prevent perinatal transmission of HBV. Pregnant women who are HBsAg positive but have low viral loads may not require antiviral therapy, but should still receive immunoprophylaxis for their newborns. | Yes | Yes |  | Yes |
| 87 | AASLD Guideline questions | 高病毒载量的乙型肝炎表面抗原阳性孕妇是否应在妊娠晚期接受抗病毒治疗以预防HBV的围产期传播？ | 我不是医生，因此不能提供具体的医学建议。然而，我可以向您提供一些一般性的信息。 乙型肝炎病毒（HBV）是一种可以通过血液和其他体液传播的病毒，而孕妇如果感染了HBV，有可能将病毒传给新生儿。高病毒载量的乙型肝炎表面抗原（HBsAg）阳性是一种孕妇可能会传播给胎儿的风险因素。 抗病毒治疗是否适合孕妇需要根据具体情况来判断，包括孕妇的病毒载量、肝功能状况、孕期、医学历史等因素。对于乙肝携带者的孕妇，医生可能会根据孕妇的具体情况来评估是否需要进行抗病毒治疗，以降低围产期传播的风险。 如果您或您认识的人是乙型肝炎表面抗原阳性的孕妇，建议咨询专业医生的意见。医生将能够根据具体情况制定适当的治疗计划，并权衡治疗的利弊，以确保母婴的健康。 | 我不是医生，但我可以提供一些一般性的信息。乙型肝炎（HBV）是一种由乙型肝炎病毒引起的病毒性感染。对于怀孕的女性，特别是在妊娠晚期，如果她们是高病毒载量的乙型肝炎表面抗原（HBsAg）阳性，医生可能会考虑采取一些预防措施，以减少新生儿HBV感染的风险。 一般而言，垂直传播是新生儿感染HBV的主要途径。在这种情况下，医生可能会考虑采取以下预防措施： 1.乙肝免疫球蛋白（HBIG）注射： 在新生儿出生后的12小时内，可以给予HBIG注射，以提供即时 passively 获得的被动免疫，减少HBV传播的风险。 2.乙肝疫苗接种： 新生儿应该在出生后尽早接种乙肝疫苗，这样可以提高他们对乙型肝炎的主动免疫水平。 此外，医生还可能根据具体情况考虑给予抗病毒药物，以降低母体病毒载量，减少传播的风险。然而，对于是否使用抗病毒药物以及具体使用哪种药物，应该由专业的医疗团队评估患者的个体情况，并根据权衡利弊作出决策。 如果你或者你认识的人有类似的情况，强烈建议咨询专业医生的建议，他们能够提供个性化的医疗建议和治疗方案。 | No | No |  | No |
| 88 | AASLD Guideline questions | Should children with HBeAg-positive CHB be treated with antiviral therapy to decrease liver-related complications? | Yes, children with HBeAg-positive chronic hepatitis B (CHB) should be treated with antiviral therapy to decrease liver-related complications. HBeAg-positive CHB is a chronic viral infection of the liver caused by the hepatitis B virus (HBV) that can cause liver inflammation, liver scarring, liver failure, and liver cancer. Antiviral therapy can suppress the virus, decrease liver inflammation, and improve liver function. The American Association for the Study of Liver Diseases (AASLD) and the European Association for the Study of the Liver (EASL) recommend antiviral therapy for children with HBeAg-positive CHB who have evidence of liver inflammation or elevated liver enzymes, regardless of the presence or absence of symptoms. The goal of therapy is to achieve long-term suppression of HBV replication and prevent progression of liver disease. The choice of antiviral therapy depends on several factors, including the child's age, degree of liver inflammation, and HBV DNA levels. Current antiviral medications include oral nucleoside/nucleotide analogs such as tenofovir or entecavir. It is important to note that antiviral therapy for CHB is usually long-term, and treatment duration should be determined on a case-by-case basis. Additionally, regular monitoring of liver function and viral load is necessary to assess treatment response and ensure optimal outcomes. | Yes, antiviral therapy is recommended for children with chronic hepatitis B (CHB) who are positive for hepatitis B e antigen (HBeAg) to decrease the risk of liver-related complications. HBeAg-positive CHB is associated with a higher risk of liver-related complications, such as cirrhosis, liver failure, and hepatocellular carcinoma (HCC). Antiviral therapy can suppress viral replication, improve liver histology, and reduce the risk of these complications. The most commonly used antiviral agents for the treatment of HBeAg-positive CHB in children include interferon alpha and nucleos(t)ide analogs such as entecavir and tenofovir. The choice of therapy depends on various factors such as age, viral load, liver function, and presence of other medical conditions. It is important to note that the treatment of children with CHB requires specialized care and close monitoring, and should be managed by a pediatric hepatologist or gastroenterologist experienced in the management of viral hepatitis. | Yes | No | Yes | Yes |
| 88 | AASLD Guideline questions | 阳性慢性乙型病毒性肝炎患儿是否应接受抗病毒治疗以减少肝脏相关并发症？ | 乙型病毒性肝炎（HBV）是一种由乙型肝炎病毒（HBV）引起的慢性病毒感染。对于阳性慢性乙型病毒性肝炎患儿是否应接受抗病毒治疗，通常需要综合考虑多个因素，包括病毒活动性、肝功能、肝组织病理学改变、患者的年龄和全身状况等。 一般而言，对于患有慢性乙型病毒性肝炎的患者，抗病毒治疗的目标是降低病毒载量、减缓肝脏病变进程，从而减少肝脏相关并发症的发生，例如肝硬化和肝癌。国际上通常采用的治疗指南（例如美国肝病学会和欧洲肝脏病学会的指南）提供了相应的治疗建议。 在考虑治疗时，医生可能会考虑以下一些因素： 1.病毒活动性： 根据病毒载量、HBV DNA水平以及肝功能等指标来评估病毒活动性。 2.肝功能： 评估肝功能的严重程度，包括肝酶水平和肝功能测试。 3.肝组织病理学： 通过肝活检等手段评估肝脏的病理学改变，以确定是否存在显著的炎症和纤维化。 4.年龄和全身状况： 考虑患者的年龄、全身状况以及患者是否有其他健康问题。 5.治疗禁忌症： 考虑是否存在任何禁忌症，例如对抗病毒药物的过敏反应。 在制定治疗方案时，医生会根据患者的具体情况权衡治疗的利弊。对于一些患者，可能会推荐开始抗病毒治疗，而对于另一些患者，可能会选择定期监测并延迟治疗。 因此，如果你或你认识的人患有慢性乙型病毒性肝炎，建议咨询专业的医生或肝脏病专家，以便根据具体情况制定最合适的治疗方案。医生会根据患者的情况制定个性化的治疗计划。 | 我不是医生，因此不能提供专业医学建议。但一般来说，对于慢性乙型病毒性肝炎的患者，是否需要接受抗病毒治疗应该由医生根据患者的具体情况来决定。以下是一些通常考虑的因素： 1.病毒活动性： 医生通常会评估乙型肝炎病毒的活动程度。如果病毒活动性较高，可能会倾向于开始抗病毒治疗，以减少病毒复制并防止肝脏进一步受损。 2.肝功能： 医生会评估患者的肝功能，包括肝酶水平和其他指标。如果肝功能受损，可能需要考虑抗病毒治疗以减轻肝脏负担。 3.肝纤维化和肝硬化： 如果患者已经出现了肝纤维化或肝硬化的迹象，可能需要更积极地干预，以防止疾病进展。 4.患者的整体健康状况： 医生还会考虑患者的整体健康状况，包括其他慢性病、免疫状态等因素。 请务必咨询专业医生，以获取针对个体情况的建议。他们可以评估患者的病历、实验室结果和其他相关信息，并制定最合适的治疗方案。 | No | No |  | No |
| 89 | Simulated Patients | A 62-year-old male patient, HBeAg positive, HBsAg 10^4 IU/mL, without cirrhosis, ALT 25 U/L, with hypertension, was not on antiviral therapy. Please provide him with advice on therapy and daily monitor and management from the perspective of liver disease treatment and health management. | I am not a medical professional, but I can provide you with some general information that might be helpful. However, it's crucial to consult with a healthcare professional for advice tailored to the specific situation of the patient. The information provided here may not be up-to-date or applicable to individual cases. Given the patient's profile, it seems like he has chronic hepatitis B (CHB) with a positive HBeAg and a moderate viral load. The management of chronic hepatitis B typically involves antiviral therapy to suppress the viral replication and reduce the risk of liver complications. Here are some general considerations for therapy and management: 1.Antiviral Therapy: Patients with chronic hepatitis B and elevated viral load, like in this case, may be candidates for antiviral therapy. Consult with a hepatologist or gastroenterologist to determine the most appropriate antiviral medication based on the patient's overall health, potential drug interactions, and guidelines in force at the time. Common antiviral medications for hepatitis B include entecavir, tenofovir disoproxil fumarate (TDF), and tenofovir alafenamide (TAF). 2.Monitoring: Regular monitoring is essential to assess the effectiveness of treatment and detect any potential side effects. Liver function tests (including ALT levels) should be monitored regularly to evaluate liver health. Viral load and serological markers (such as HBsAg and HBeAg) should be checked periodically to assess the response to antiviral therapy. 3.Liver Disease Management: Management of comorbidities, such as hypertension, is crucial. Blood pressure should be monitored regularly, and appropriate medications may be prescribed to control hypertension. Lifestyle modifications, including a healthy diet, regular exercise, and abstaining from alcohol, can contribute to overall liver health. 1.Vaccination: Ensure that the patient is vaccinated against hepatitis A and B if not already immune to these viruses. 2.Regular Follow-Up: Schedule regular follow-up appointments with the healthcare provider to adjust the treatment plan as needed and monitor the patient's overall health. It's important to note that this information is general and may not cover all aspects of the patient's unique situation. The healthcare provider overseeing the patient's care will make the final decisions regarding therapy and management based on the specific circumstances of the case. | I am not a doctor, but I can offer some general information that might help guide the discussion with a healthcare professional. It's important for the patient to consult with a healthcare provider for personalized advice based on their specific health condition. Given the provided information, it appears that the patient is HBeAg positive, indicating active viral replication in the hepatitis B virus (HBV). The HBsAg level is 10^4 IU/mL, which suggests a moderate to high viral load. The ALT level is within the normal range, indicating that the liver enzyme levels are not elevated at the moment. The patient also has hypertension. Treatment decisions for chronic hepatitis B are complex and depend on various factors, including liver function, viral load, and the presence of other medical conditions. The guidelines for managing chronic hepatitis B may vary, and it's crucial for the patient to consult with a hepatologist or infectious disease specialist for a personalized treatment plan. Here are some general considerations for the patient: 1.Consult with a Specialist: The patient should schedule an appointment with a hepatologist or an infectious disease specialist who has expertise in managing chronic hepatitis B. 2.Antiviral Therapy: Based on current guidelines, antiviral therapy may be considered for HBeAg-positive patients with a high viral load. Commonly prescribed antiviral medications include tenofovir or entecavir. The decision to start antiviral therapy should be made by the healthcare provider after considering the patient's overall health and potential side effects of the medications. 3.Regular Monitoring: Regular follow-up visits and monitoring are essential to assess the response to treatment, liver function, and the progression of the disease. This may involve periodic blood tests, including liver function tests and viral load measurements. 4.Management of Hypertension: Given the presence of hypertension, the patient should also receive appropriate management for blood pressure control. Lifestyle modifications, such as a healthy diet and regular exercise, may be recommended in addition to any prescribed medications. 5.Vaccination for Hepatitis A and B: If the patient has not been previously vaccinated for hepatitis A and B, vaccination should be considered to prevent additional liver-related complications. 6.Avoidance of Alcohol and Hepatotoxic Substances: The patient should be advised to avoid alcohol and substances that can be harmful to the liver. It's important for the patient to communicate openly with their healthcare provider, adhere to the recommended treatment plan, and make lifestyle modifications to promote overall liver health. Additionally, regular monitoring and follow-up visits are crucial for managing chronic hepatitis B effectively. | Yes | Yes |  | Yes |
| 89 | Simulated Patients | 一个62岁的男性患者，HBeAg阳性，HBsAg 10^4 IU/mL，没有肝硬化，ALT 25 U/L，有高血压，未进行抗病毒治疗。请从肝病治疗和健康管理的角度，给他提供治疗和日常管理方面的建议。 | 这位患者的情况涉及到慢性乙型肝炎（Chronic Hepatitis B，CHB），需要综合考虑肝病治疗和相关的健康管理方面的建议。请注意，以下建议仅供参考，具体的治疗和管理决策应由专业医生根据患者的具体情况进行制定。 1. 肝病治疗： 由于患者是HBeAg阳性，血清HBV-DNA水平较高，建议考虑进行抗病毒治疗，以减少病毒复制和防止疾病的进展。常用的抗病毒药物包括去氨酸阿德福韦（adefovir）、恩替卡韦（entecavir）、替比夫定（tenofovir）等。 治疗的目标是通过抑制病毒复制，降低血清HBV-DNA水平，改善肝功能，并降低患者发展肝硬化和肝癌的风险。 2. 健康管理和生活方式： 控制高血压：积极管理高血压，采取药物治疗和生活方式干预，如饮食控制、体育锻炼、戒烟等，以降低心血管疾病风险。 定期监测肝功能：通过定期检查ALT、AST、肝功能等指标，评估肝脏健康状况，及时发现问题并采取相应的干预措施。 饮食控制：建议患者采取低脂、低盐、高纤维的饮食，避免酗酒，以减轻肝脏负担。 防止传播：患者应采取预防措施，以防止HBV传播给他人。接受家庭成员的疫苗接种，避免共用个人卫生用品。 3. 定期随访： 患者应该定期进行随访，监测病情变化，调整治疗方案。 随访时医生可能会根据病情调整药物剂量或更换药物，确保最佳的治疗效果。 总体而言，这位患者需要一个综合的治疗和管理计划，涵盖抗病毒治疗、高血压管理、饮食控制以及定期随访。治疗方案应该由专业的医生制定，根据患者的具体情况进行调整。在整个治疗过程中，患者需要密切配合医生的建议，保持良好的生活方式和定期的随访。 | 对于这位62岁的男性患者，他是HBeAg阳性，HBsAg水平较高，但ALT水平正常，没有肝硬化，同时有高血压。以下是一些建议，但请注意这只是一般性建议，具体治疗方案应该由专业医生根据病人的具体情况来确定。 肝病治疗建议： 1.评估病毒活动性： 进一步检查HBV DNA水平，以确定病毒活动性的程度。 2.抗病毒治疗： 对于HBeAg阳性的患者，抗病毒治疗是必要的。常用的药物包括拜瑞那韦（Entecavir）或替比夫定（Tenofovir）。 抗病毒治疗有助于抑制病毒复制，减缓肝脏病变进程。 3.定期随访： 定期监测HBV DNA、ALT、AST等指标，以及肝功能的变化。 考虑进行肝脏超声等影像学检查，监测肝脏病变的发展。 健康管理建议： 1.高血压管理： 控制血压在目标范围内，以减轻心脏和肾脏的负担。 遵循医生的建议，按时服用抗高血压药物。 2.生活方式调整： 避免饮酒，因为酒精可能对肝脏产生不良影响。 保持健康的饮食，避免高脂肪、高糖和高盐饮食。 进行适度的体育锻炼，但要遵循医生的建议。 3.疫苗接种： 接种乙肝疫苗，以防止其他人感染。 4.医学监测： 定期进行肝功能检查、血液检查以及其他相关检查，确保早期发现和处理任何潜在的问题。 5.药物相互作用： 注意药物相互作用，特别是抗病毒药物和其他药物之间的相互影响。 6.心理健康： 维持良好的心理健康，可以考虑参与支持团体或与医疗团队沟通，以解决可能的情绪和心理压力。 总体而言，制定适当的治疗方案和生活方式调整计划需要在专业医生的指导下进行。请务必咨询医生，以制定适合该患者独特情况的个体化计划。 | No | No |  | No |
| 90 | Simulated Patients | A 51-year-old male patient, HBeAg-negative, HBsAg 10^1 IU/mL, without cirrhosis, ALT 28 U/L, was not on antiviral therapy and was an habitual smoker. Does he need antiviral treatment and why? | I am not a doctor, but I can provide some general information that might be helpful for you to discuss with a healthcare professional. In this case, the patient is HBeAg-negative, has a low HBsAg level, normal ALT (alanine transaminase), and no cirrhosis. The decision to start antiviral treatment in chronic hepatitis B depends on various factors, and it's crucial to consult with a healthcare provider for personalized advice. Here are some considerations: 1.HBV DNA Levels: While you provided the HBsAg level, information about the HBV DNA viral load is essential. The decision to start antiviral treatment often considers the level of viral replication in the blood. 2.Liver Function: The ALT level is normal, suggesting the liver is not currently inflamed. However, periodic monitoring is necessary to assess any changes over time. 3.Smoking: Smoking is a risk factor for liver disease progression. It's important for the patient to address lifestyle factors, such as smoking, to improve overall health. 4.Age and Other Health Conditions: The patient's age may be a factor in the decision-making process. Other health conditions, such as diabetes or obesity, may also influence the decision. 5.Guidelines: Treatment decisions are often guided by established medical guidelines. Healthcare providers may consider guidelines from organizations like the American Association for the Study of Liver Diseases (AASLD) or the European Association for the Study of the Liver (EASL). 6.Patient Preferences: The patient's preferences, concerns, and willingness to adhere to long-term treatment are important factors in the decision-making process. It's crucial for the patient to have a detailed discussion with a healthcare provider who can assess the individual's overall health, review test results, and consider various factors to determine the most appropriate course of action. Antiviral therapy may be recommended to suppress viral replication, prevent liver damage, and reduce the risk of complications, but this decision should be made on a case-by-case basis. | I am not a medical professional, but I can provide some general information based on the details you've provided. It's crucial to consult with a healthcare professional for personalized advice tailored to the specific situation. The decision to start antiviral treatment for hepatitis B (HBV) is complex and depends on various factors, including liver function, viral load, and the presence of other medical conditions. Here are some considerations: 1.HBV DNA (viral load): The patient's HBV DNA level is an important factor in determining the need for treatment. An HBsAg level of 10^1 IU/mL suggests a moderate viral load. High viral loads are associated with an increased risk of disease progression and may influence the decision to start antiviral therapy. 2.Liver enzymes (ALT): A normal ALT level (28 U/L in this case) is generally a positive indicator, suggesting that the liver is not currently experiencing significant inflammation or damage. However, it's essential to consider trends in ALT levels over time. 3.Liver cirrhosis: The absence of cirrhosis is a positive factor, as cirrhosis is a more advanced stage of liver disease and may influence the decision to initiate treatment. 4.HBeAg-negative status: The patient is HBeAg-negative, which may indicate a transition to a less active phase of the infection. However, other factors must be considered in the overall assessment. 5.Smoking habit: Smoking can contribute to overall health issues, including liver health. Addressing lifestyle factors, such as smoking cessation, is generally beneficial for overall well-being. It's crucial for the patient to consult with a hepatologist or a healthcare professional experienced in managing hepatitis B. They will consider the overall clinical picture, including the factors mentioned above, and may order additional tests if needed. The decision to start antiviral treatment is typically individualized, and healthcare providers consider the potential risks and benefits for each patient. Please note that guidelines for the management of hepatitis B may vary, and the information provided here is not a substitute for professional medical advice. | No | No |  | No |
| 90 | Simulated Patients | 一个51岁的男性患者，HBeAg阴性，HBsAg 10^1 IU/mL，没有肝硬化，ALT 28 U/L，未进行抗病毒治疗，平时有吸烟习惯。他是否需要应该进行抗病毒治疗，理由是什么？ | 首先，我不是医生，因此我的回答仅供参考，具体的医疗决策应由专业医生根据详细的患者状况进行。在这种情况下，这位男性患者的HBeAg阴性、HBsAg为10^1 IU/mL、ALT正常，且没有肝硬化的情况下，通常不是立即进行抗病毒治疗的迹象。 当前的临床指南一般是根据乙型肝炎病毒（HBV）感染的肝功能、病毒复制水平、肝纤维化等因素来判断是否需要抗病毒治疗。在这个患者的情况下，ALT正常，表示肝脏目前没有活跃的炎症，而HBeAg阴性通常表示病毒复制相对较低。此外，缺乏肝硬化的证据也是一个积极的因素。 然而，吸烟是一个可能影响肝炎患者病程的不良因素。吸烟可能导致肝炎的进展，增加肝癌的风险。因此，医生可能会建议患者改变生活方式，戒烟以及保持健康的生活习惯。 最好的做法是咨询专业医生，他们将根据患者的个体情况、实验室检查结果和最新的治疗指南为患者提供最合适的治疗建议。 | 我不是医生，但我可以提供一些一般性的信息。对于慢性乙型肝炎（HBV）的治疗决策通常需要综合考虑多个因素，并且最好由专业医生进行评估。在这种情况下，需要考虑的因素包括患者的病毒载量、肝功能、肝组织病理学评估、家庭和个体史，以及是否存在合并症等。 在提供具体建议之前，建议您咨询专业医生，进行全面的体格检查和相关检测。通常情况下，对于HBeAg阴性、HBsAg阳性的慢性HBV感染，医生可能会考虑以下一些建议： 1.HBV DNA水平： 患者的病毒载量（HBV DNA水平）是一个重要的指标。如果病毒载量较高，可能需要考虑抗病毒治疗。 2.肝功能： ALT水平正常是一个好的指标，但仍需要考虑其他肝功能指标，以确保肝脏正常。 3.肝组织病理学评估： 肝活检可能有助于评估肝脏的病理学情况，了解是否存在肝纤维化或炎症。 4.合并症： 是否存在其他合并症，如肝硬化、肝细胞癌等，也会影响治疗的决策。 5.年龄和生活方式： 患者的年龄、吸烟史等因素也可能影响治疗决策。 总体而言，决定是否进行抗病毒治疗应该是个体化的，并且需要医生的综合评估。因此，建议您尽快咨询专业医生，以获取准确的建议和治疗方案。 | No | No |  | No |
| 91 | Simulated Patients | A 37-year-old female patient with HBV-DNA 10^7 IU/ml, HBeAg positive, cirrhosis, eGFR 102 ml/min, ALT 215 U/L, BMI 24.3kg/m^2, no alcohol habit, had just started treatment with tenofovir. How often should the patient be monitored? What should the project include? | The management and monitoring of a patient with chronic hepatitis B (HBV) undergoing antiviral therapy, such as tenofovir, involves several parameters to assess treatment response, liver function, and potential side effects. The frequency and scope of monitoring may vary based on guidelines and individual patient characteristics, and it's important for the healthcare provider to tailor the monitoring plan accordingly. Below is a suggested monitoring schedule and key parameters to include in the follow-up of this patient:  Monitoring Schedule: 1.Initial assessment: Baseline laboratory tests before starting treatment. 2.Early follow-up (1-3 months after initiation): Laboratory tests: Liver function tests (LFTs): ALT, AST, bilirubin, albumin. HBV-DNA to assess viral load. Complete blood count (CBC). Virological markers: HBeAg status. Renal function: Serum creatinine. Estimated glomerular filtration rate (eGFR). 3.Regular follow-up (every 3-6 months): Laboratory tests: LFTs. HBV-DNA. CBC. Virological markers: HBeAg status. Renal function: Serum creatinine. eGFR. 4.Specific monitoring: Bone mineral density (especially in long-term use of tenofovir). Regular blood pressure checks. Monitoring for signs of resistance or treatment failure.  Projected Monitoring Parameters: 1.Liver Function Tests (LFTs): Alanine aminotransferase (ALT). Aspartate aminotransferase (AST). Bilirubin. Albumin. 2.Virological Markers: HBV-DNA quantification. HBeAg status. 3.Renal Function: Serum creatinine. Estimated Glomerular Filtration Rate (eGFR). 4.Complete Blood Count (CBC): Hemoglobin. Platelet count. White blood cell count. 5.Other Monitoring: Bone mineral density assessments. Blood pressure measurements. Monitoring for potential side effects, adherence, and signs of resistance. 6.Imaging studies: Liver imaging (e.g., ultrasound) may be considered based on clinical indications.  Considerations: 1.The patient's age, gender, and BMI are important baseline characteristics. 2.Regular assessment of adherence to medication. 3.Patient education on signs of liver decompensation, renal dysfunction, and other potential side effects.  It's crucial to note that these recommendations are general, and individual patient management may require adjustments based on the healthcare provider's clinical judgment, specific guidelines, and the patient's response to treatment. Regular communication between the patient and healthcare provider is essential for optimizing care. Always refer to the latest guidelines and protocols for managing chronic hepatitis B in your region. | The management and monitoring of a patient with chronic hepatitis B (CHB) undergoing antiviral therapy involve various aspects to ensure optimal care and early detection of potential complications. Here are some general recommendations; however, it's important to note that individual cases may vary, and specific recommendations should be tailored by healthcare providers based on the patient's unique situation.  Monitoring Schedule: 1.Baseline Assessments (before starting treatment): Liver function tests (including ALT, AST, and alkaline phosphatase) Hepatitis B serology (HBeAg status, HBV-DNA levels) Renal function (eGFR) Imaging studies to assess the degree of liver cirrhosis Complete blood count (CBC) Hepatitis B e-antigen (HBeAg) status 2.During Treatment: Every 3 Months: Liver function tests (ALT, AST) HBV-DNA levels Renal function (eGFR) Monitoring for adverse effects related to tenofovir Every 6 Months: Hepatitis B serology (HBeAg status) 3.Annually: Imaging studies to assess the degree of liver cirrhosis (e.g., ultrasound, fibroscan) Complete blood count (CBC)  Projected Monitoring Parameters: 1.Liver Function Tests (LFTs): ALT and AST: Monitor for liver inflammation and assess treatment response. Alkaline Phosphatase: Evaluate liver function and assess for bile duct obstruction. 2.Viral Load (HBV-DNA): Regular monitoring to assess the effectiveness of antiviral therapy and determine the need for adjustments. 3.Renal Function: eGFR: Regular assessment to monitor for potential nephrotoxicity associated with tenofovir. 4.Hepatitis B Serology: HBeAg Status: Assess changes in HBeAg status, as it may indicate a shift from an active to an inactive phase of infection. 5.Imaging Studies: Assess liver cirrhosis and screen for hepatocellular carcinoma. Frequency may vary based on individual risk factors and provider discretion. 6.Complete Blood Count (CBC): Monitor for any abnormalities, including anemia or thrombocytopenia. 7.Adverse Effects Monitoring: Keep a vigilant eye for any adverse effects related to tenofovir, particularly renal toxicity and bone health. 8.Patient Education: Provide education on the importance of medication adherence and the potential risks and benefits of treatment.  Encourage lifestyle modifications (e.g., no alcohol consumption) to promote liver health. It's crucial for the patient to have regular follow-ups with their healthcare provider to ensure proper management and timely adjustments to the treatment plan based on the monitoring results. This information is a general guideline, and the specific monitoring schedule may vary based on the individual patient's response to treatment and their overall health status. Always consult with a healthcare professional for personalized advice and management. | Yes | Yes |  | Yes |
| 91 | Simulated Patients | 一个37岁的女性患者，HBV-DNA 10^7 IU/ml，HBeAg阳性，有肝硬化，eGFR 102 ml/min，ALT 215 U/L，BMI 24.3kg/m^2，没有饮酒习惯，刚开始服用替诺福韦治疗。这位患者日常监测的频率应当为多久一次？项目应当包括哪些？ | 对于这位37岁的女性患者，患有乙型肝炎病毒（HBV）感染，具有以下一些关键的监测项目，监测频率和项目选择可能会有些许变化，具体的管理应该由专业医生根据患者的具体情况进行。 1.HBV-DNA水平监测： 在替诺福韦治疗开始后的前3个月内，建议每4周监测一次。 之后，如果患者稳定，可以逐渐延长监测间隔，比如每3-6个月进行一次。 2.肝功能监测： ALT（丙氨酸氨基转移酶）水平应该定期监测，初始阶段可能需要更频繁的监测，每4周一次，以确保治疗的有效性。 之后，如果患者稳定，可以逐渐延长监测间隔，比如每3-6个月进行一次。 3.肾功能监测： 由于患者有肝硬化，eGFR（肾小球滤过率）值也需要定期监测，以确保替诺福韦对肾功能没有不良影响。初始阶段可以每3个月进行一次，之后可以逐渐延长监测间隔。 4.乙型肝炎e抗原（HBeAg）和抗体（anti-HBe）： HBeAg和anti-HBe的监测有助于评估疾病的发展和治疗的效果。初期可能需要更频繁的监测，每3-6个月一次。 5.BMI（身体质量指数）监测： BMI的监测有助于了解患者的整体健康状况，但不需要经常性监测。初始阶段和治疗调整期间可能需要每6个月进行一次。 6.其他检查： 定期进行肝脾超声检查，以评估肝硬化和肝脾的状态。 总体而言，治疗监测的频率和项目的选择应该根据患者的具体情况、治疗反应以及任何潜在的不良反应而定。医生会根据患者的具体状况来调整监测计划。此外，患者还应该遵循医生的建议，按时完成所有的检查和监测。 | 对于这位37岁的女性患者，患有乙型肝炎病毒（HBV）感染，已经发展成肝硬化，正在接受替诺福韦治疗。在治疗过程中，需要进行定期监测以评估疗效和安全性。监测的频率和项目可以根据患者的情况进行调整，但通常包括以下方面： 1.HBV-DNA测定： 初始治疗后的2-3个月内，需要定期监测HBV-DNA水平以评估病毒抑制的效果。在病毒负荷明显下降后，可以逐渐减少监测频率。 2.肝功能测试： 包括ALT（谷丙转氨酶）水平，用于评估肝细胞的损伤和炎症程度。初始治疗后的2-3个月内，可以更频繁地进行监测，然后根据稳定的情况减少监测频率。  3.肾功能测试： 由于替诺福韦可以对肾功能产生一定影响，需要定期监测患者的eGFR（肾小球滤过率）。初始治疗后的2-3个月内，可以更频繁地进行监测，然后根据稳定的情况减少监测频率。 4.乙型肝炎e抗原（HBeAg）检测： 对于HBeAg阳性的患者，需要监测HBeAg是否转阴（变为阴性），以评估疾病的进展和治疗效果。 5.影像学检查： 对于存在肝硬化的患者，可能需要进行定期的肝脾CT或超声检查，以评估肝脏的形态和结构，以及检测肿块等异常。 6.体重和BMI监测： 需要监测患者的体重和BMI，以评估患者的整体健康状况。 治疗的具体计划和监测频率应该由专业医生根据患者的具体情况来确定。患者应该定期复诊，医生会根据监测结果调整治疗方案。 | Yes | Yes |  | Yes |
| 92 | Simulated Patients | A 36-year-old female patient with HBV-DNA 10^6 IU/ml, HBeAg positive, uncertain whether she had cirrhosis, eGFR 109 ml/min, ALT 119 U/L, BMI 30.2kg/m^2, diabetes mellitus, had been treated with entecavir for 1 year. How should this patient conduct daily monitoring and lifestyle management? | I am not a medical professional, but I can offer some general suggestions based on the information provided. It's important for the patient to consult with their healthcare provider for personalized advice. 1. Medication Adherence: Continue taking entecavir as prescribed by the healthcare provider. 2. Monitoring: Regular monitoring of liver function tests (ALT, AST) to assess the response to treatment and liver health. Periodic measurement of HBV-DNA levels to evaluate the effectiveness of antiviral therapy. Imaging studies or liver biopsy may be considered to assess cirrhosis status. 3. Lifestyle Management: Diet and Exercise: Maintain a healthy diet and engage in regular physical activity to manage BMI and promote overall health. Weight loss, if needed, can positively impact liver health. Blood Sugar Control: Given the presence of diabetes mellitus, it's crucial to manage blood glucose levels through medication, diet, and exercise. Alcohol Avoidance: As the patient has liver disease, avoiding alcohol is essential to prevent further damage. Regular Follow-up: Schedule regular follow-up appointments with the healthcare provider to monitor overall health and adjust the treatment plan if necessary. 4. Vaccination: Ensure up-to-date vaccinations, including hepatitis A and hepatitis B vaccines if not already received. 5. Assessing Cirrhosis: Further evaluation for cirrhosis may be necessary, such as imaging studies (ultrasound, FibroScan) or a liver biopsy. 6. Managing Comorbidities: Address and manage other comorbidities, such as diabetes, through appropriate medications, lifestyle modifications, and regular monitoring. 7. Kidney Function Monitoring: Regularly monitor kidney function, especially considering the patient's eGFR. Adjustments to medication may be needed if kidney function changes. 8. Consultation with Specialists: Collaborate with hepatologists, endocrinologists, and other specialists as needed for comprehensive care. It's crucial for the patient to maintain open communication with their healthcare team, adhere to prescribed medications, and make lifestyle modifications to manage overall health effectively. This information is for general guidance, and the patient's specific circumstances may warrant individualized recommendations from their healthcare provider. | I am not a doctor, but I can offer some general information that might be helpful for you to discuss with a healthcare professional. It's important for the patient to have regular follow-ups with a healthcare provider for proper management of chronic conditions like hepatitis B, diabetes, and obesity. Here are some general suggestions: 1.Hepatitis B Monitoring: Regular follow-ups with a hepatologist or infectious disease specialist are crucial. Monitoring of HBV-DNA levels, liver function tests (including ALT levels), and HBeAg status may be necessary to assess the response to entecavir therapy. 2.Liver Imaging: Consider liver imaging studies (such as ultrasound, FibroScan, or MRI) to assess the degree of liver fibrosis or cirrhosis. 3.Diabetes Management: Strict management of diabetes is essential. Regular monitoring of blood glucose levels and HbA1c is important. Lifestyle modifications, including a healthy diet and regular exercise, can help control diabetes and manage weight. 4.Weight Management: Achieving and maintaining a healthy weight is crucial, especially with a BMI of 30.2 kg/m^2. Weight loss can help improve liver function and reduce the risk of complications. A balanced diet with a focus on fruits, vegetables, whole grains, and lean proteins is recommended. 5.Kidney Function Monitoring: Regular monitoring of kidney function is important, especially with an eGFR of 109 ml/min. This can include regular testing of serum creatinine and other kidney function markers. 6.Regular Exercise: Engaging in regular physical activity is beneficial for both diabetes management and weight control. Consult with a healthcare provider before starting a new exercise program. 7.Alcohol Avoidance: Given the liver condition, it is advisable to avoid or limit alcohol consumption. 8.Medication Adherence: Ensure strict adherence to the prescribed entecavir treatment regimen. 9.Vaccination: Confirm that the patient is up-to-date on hepatitis B vaccinations and other recommended vaccinations. It's crucial for the patient to work closely with their healthcare team to develop an individualized care plan based on their specific medical history and current condition. This information is general in nature and not a substitute for professional medical advice. Always consult with a healthcare provider for personalized guidance. | Yes | Yes |  | Yes |
| 92 | Simulated Patients | 一个36岁的女性患者，HBV-DNA 10^6 IU/ml，HBeAg阳性，不确定自己是否有肝硬化，eGFR 109 ml/min，ALT 119 U/L，BMI 30.2kg/m^2，有糖尿病，已经服用恩替卡韦治疗1年。这位患者应当如何进行日常监测与生活方式管理？ | 对于这位36岁的女性患者，患有乙型肝炎病毒（HBV）感染，以下是一些建议： 1.肝功能监测： 定期监测血清转氨酶（ALT）水平，以评估肝脏炎症的活动性。 考虑进行肝弹性测定或肝组织活检来评估是否存在肝硬化。 2.病毒学监测： 定期监测HBV-DNA水平，以评估病毒复制水平和抗病毒治疗的有效性。 跟踪HBeAg和抗-HBe抗体的转换，以了解患者的病毒学状态。 3.肾功能监测： 定期监测估算的肾小球滤过率（eGFR），以评估肾功能。 注意糖尿病患者通常有较高的肾脏风险，需要更加密切的监测。 4.生活方式管理： 通过调整饮食和增加体育活动来控制体重，以改善BMI。 管理糖尿病，包括药物治疗、饮食控制和体育锻炼。 避免饮酒，因为酒精可能对肝脏有害。 5.抗病毒治疗： 恩替卡韦是一种抗病毒药物，患者应定期检查病毒学和肝功能参数，以确保治疗的有效性。 如果患者肝功能异常或病毒学反弹，可能需要重新评估抗病毒治疗方案。 6.定期随访： 患者应该定期接受医生的随访，以监测疾病进展并调整治疗方案。 这些建议只是一般性的指导，具体的治疗和管理方案应该由专业医生根据患者的具体情况进行制定。因此，建议患者与医生保持密切联系，共同制定和执行个性化的治疗和管理计划。 | 针对这位36岁的女性患者，患有慢性乙型肝炎（HBV），已经接受恩替卡韦治疗1年，具体的日常监测与生活方式管理建议如下： 1.定期监测肝功能和病毒指标： 肝功能监测： 比如ALT水平，以评估肝脏的炎症情况。 HBV指标监测： 持续监测HBV-DNA水平和HBeAg状态，以评估病毒复制活跃程度。 2.评估肝硬化风险： 进行肝脏成像检查，如肝脏超声或 FibroScan，以评估是否存在肝硬化。 3.监测肾功能： 定期检查肾功能，包括eGFR。高eGFR可能是由于糖尿病引起的肾脏功能异常，需要密切监测。 4.生活方式管理： 体重管理： 由于BMI较高，建议采取措施进行体重管理，以减轻脂肪肝的风险。 糖尿病管理： 对糖尿病的治疗需要定期监测血糖水平，遵循医生的治疗方案，注意饮食和锻炼。 饮酒限制： 避免过量饮酒，因为酒精对肝脏有害，尤其是对乙型肝炎患者。 5.药物治疗： 恩替卡韦是一种乙型肝炎病毒核苷(t)酸类似物，需要持续使用，同时需要定期检查病毒指标来评估治疗效果。 6.接种疫苗： 如果尚未完成，应接种乙型肝炎疫苗以预防新的HBV感染。 7.定期随访医生： 定期复诊，确保定期进行各项检查，根据检查结果进行治疗调整。 患者的治疗和管理需要由专业的医生根据具体情况制定，因此上述建议仅供参考，具体的治疗方案应由专业医生根据患者的详细病史、检查结果和临床状况来制定。 | Yes | Yes |  | Yes |
| 93 | Simulated Patients | A 44-year-old female patient with HBV-DNA 10^8 IU/ml, HBeAg positive, no cirrhosis, eGFR 105 ml/min, ALT 110 U/L, BMI 22.5kg/m^2, a history of hypertension, smoking and drinking habits, and no antiviral therapy. Please advise the medication regimen and lifestyle of the patient. | I am not a doctor, and it's crucial to consult with a healthcare professional for personalized medical advice. However, I can provide some general information based on the details you've provided. Given the patient's clinical profile with chronic hepatitis B (CHB), it's important to consider antiviral therapy to manage the infection and prevent disease progression. The decision to start antiviral treatment should be made by a healthcare professional after a thorough assessment of the patient's medical history, laboratory results, and overall health. In the case of HBeAg-positive CHB with a high viral load, one of the commonly used antiviral medications is oral antiviral therapy with nucleoside or nucleotide analogs. Medications like tenofovir or entecavir are often considered as first-line treatment options. The choice of medication should be based on factors such as drug resistance profiles, potential side effects, and the patient's overall health. Lifestyle modifications are also essential for managing chronic hepatitis B. Here are some general recommendations: 1.Alcohol and Smoking Cessation: Strongly advise the patient to quit smoking and limit alcohol consumption. Both smoking and excessive alcohol intake can exacerbate liver damage. 2.Diet and Weight Management: Encourage a healthy, well-balanced diet to maintain a healthy weight. Consider consultation with a dietitian for personalized dietary recommendations. 3.Blood Pressure Control: Monitor and manage hypertension to reduce the risk of cardiovascular complications. 4.Regular Exercise: Promote regular physical activity, as it contributes to overall health. 5.Regular Follow-up and Monitoring: Schedule regular follow-up appointments with healthcare providers for monitoring liver function, viral load, and other relevant parameters. 6.Vaccination: Ensure the patient is up-to-date on vaccinations, including hepatitis A and B. It's crucial for the patient to adhere to the prescribed medication regimen and attend regular follow-up appointments. The healthcare provider will assess the patient's response to treatment, monitor liver function, and make adjustments to the treatment plan if necessary. This information is general, and individual cases may vary. The patient should consult with their healthcare provider for specific guidance tailored to their health status and needs. | I am not a medical professional, and it's important to consult with a healthcare provider for personalized advice. However, based on the information provided, here are some general considerations:  Medication Regimen: Given that the patient has chronic hepatitis B (CHB) with a high viral load (HBV-DNA 10^8 IU/ml) and is HBeAg positive, antiviral therapy may be considered. Commonly used antiviral medications for chronic hepatitis B include: 1.Nucleos(t)ide Analogues (NAs): Tenofovir disoproxil fumarate (TDF) or Tenofovir alafenamide (TAF): These are potent antiviral drugs commonly used for CHB. OR Entecavir: Another effective antiviral option for CHB. The choice between these medications should be based on various factors, including patient preference, potential side effects, and individual health considerations. The decision to start antiviral therapy should be made in consultation with a hepatologist or gastroenterologist.  Monitoring: Regular monitoring of liver function tests, including ALT, and HBV-DNA levels, is essential. HBeAg status and liver imaging may be monitored periodically.  Lifestyle Recommendations: 1.Alcohol Consumption: Given the history of drinking habits, reducing or eliminating alcohol consumption is crucial. Chronic alcohol use can exacerbate liver damage. 2.Smoking Cessation: Quitting smoking is highly recommended. Smoking can contribute to the progression of liver disease and overall health issues. 3.Blood Pressure Management: Given the history of hypertension, it's important to manage blood pressure through lifestyle modifications and, if necessary, medication. 4.Healthy Diet and Weight Management: Encourage a well-balanced diet with an emphasis on fruits, vegetables, and whole grains. Maintain a healthy BMI. 5.Regular Exercise: Engage in regular physical activity as tolerated, considering the patient's overall health status. 6.Regular Follow-up: Regularly follow up with healthcare providers to monitor liver function, viral load, and overall health. It's crucial for the patient to work closely with a healthcare team, including a hepatologist or gastroenterologist, to tailor the management plan based on individual needs and any specific contraindications. Additionally, lifestyle changes should be discussed with the patient's primary care physician or specialists as needed. | No | No |  | No |
| 93 | Simulated Patients | 一个44岁的女性患者，HBV-DNA 10^8 IU/ml，HBeAg阳性，没有肝硬化，eGFR 105 ml/min，ALT 110 U/L，BMI 22.5kg/m^2，有高血压病史，有吸烟和饮酒习惯，未进行抗病毒治疗。请给出用药方案和生活方式建议。 | 首先，需要说明我不是医生，不能提供专业医疗建议。针对HBV感染的治疗，最好咨询专业医生以获取个性化的建议。以下是一般性的信息，仅供参考。 对于这位患者，考虑到她的HBeAg阳性，高血压，吸烟和饮酒习惯，以及ALT水平升高，可能需要综合治疗的方法，包括抗病毒治疗、生活方式干预和相关药物治疗高血压。 1.抗病毒治疗： 针对患者的HBV-DNA水平，可能需要考虑抗病毒治疗。抗病毒药物的选择应由专业医生决定，通常会考虑核苷(tide)或核酸酸(tide)类药物，如阿德福韦酯或替比夫定。治疗的目标是抑制病毒复制，减缓疾病的进展。 2.生活方式建议： 戒烟戒酒：吸烟和饮酒都会对肝脏健康产生不良影响，对于HBV感染的患者来说更是如此。尽量戒烟和限制饮酒。 饮食和运动：保持健康的饮食习惯，避免高脂肪、高糖分的食物。适量的锻炼对于整体健康有益，但在开始运动之前最好咨询医生的建议。 控制体重：保持适当的体重对于控制高血压和改善肝脏健康都很重要。 3.管理高血压： 考虑高血压病史，可能需要药物治疗。抗高血压药物的选择应该由医生根据患者的具体情况来确定。 4.监测： 定期进行检查，包括HBV病毒载量、肝功能、肾功能和血压监测等，以便及时调整治疗方案。 请记住，以上建议是一般性的，具体治疗方案需要根据患者的详细病史和体检结果由专业医生来制定。患者应该密切关注医生的建议，定期进行复查，并积极参与治疗过程。 | 请注意，我不是医生，以下只是一些建议，具体的治疗方案应该由专业医生根据详细的临床情况进行制定。对于慢性乙型肝炎（HBV）的治疗，建议您咨询专业医生以获取最准确的建议。 根据提供的信息，您的患者属于慢性HBV感染，目前HBV-DNA水平较高，HBeAg阳性，ALT升高，但无肝硬化。以下是可能的治疗方案和生活方式建议： 1. 抗病毒治疗方案： 抗病毒药物： 对于HBV患者，抗病毒治疗是关键。一些常用的抗病毒药物包括： Nucleos(t)ide analogs (NAs)： 如恩替卡韦（Entecavir）、替比夫定（Tenofovir）等。这些药物可以有效抑制病毒复制，减少炎症和改善肝功能。 2. 生活方式建议： 戒烟和限制饮酒： 戒烟和限制饮酒是非常重要的。吸烟和过量饮酒都会对肝脏健康产生负面影响，尤其是在慢性乙型肝炎的情况下。 控制体重： 维持健康的体重对于肝脏健康至关重要。尽量保持适当的体重，避免肥胖。 健康饮食： 采用均衡的饮食，多摄取新鲜水果、蔬菜、全谷物和低脂蛋白质，有助于支持免疫系统和维持肝脏功能。 控制高血压： 由于患者有高血压病史，需要积极控制血压，可能需要药物治疗，同时也可以通过生活方式改变，如控制钠盐摄入、增加锻炼等。 3. 定期监测： 定期检查肝功能： 包括ALT、AST等指标，以监测肝脏炎症的程度。 HBV-DNA检测： 定期检测HBV-DNA水平，以评估病毒复制的情况。 血压监测： 定期监测血压，确保血压在合适范围内。 请务必在制定治疗方案之前与专业医生咨询。这些建议仅供参考，具体治疗方案需要根据患者的具体病情和体征由医生来确定。 | No | No |  | No |
| 94 | Simulated Patients | on antiviral therapy, came to console whether interferon could be used. Please give medical advice. | I am not a doctor, but I can provide some general information. It's important for the patient to consult with a healthcare professional for personalized advice based on their specific medical history and condition. That being said, here are some considerations: 1.HBeAg-Positive Status: The patient being HBeAg positive suggests active viral replication. This is a factor that healthcare professionals consider when deciding on antiviral therapy. 2.ALT Level: The elevated ALT (alanine aminotransferase) level indicates liver inflammation. High ALT levels are often associated with hepatitis B infection, but the specific level alone may not be the only factor to determine the need for treatment. 3.Hyperlipidemia: The presence of hyperlipidemia should also be taken into account, as it may affect overall health and complicate treatment decisions. 4.Interferon Therapy: Interferon therapy has been used in the treatment of hepatitis B, but its use can be limited by side effects and the availability of newer, more effective antiviral medications. Interferon is not typically the first-line treatment for chronic hepatitis B, and its use is often considered in specific cases. 5.Antiviral Therapy Options: Newer antiviral medications, such as nucleoside or nucleotide analogs (e.g., entecavir, tenofovir), are often preferred for the treatment of chronic hepatitis B due to their efficacy and tolerability. 6.Consultation with a Hepatologist or Infectious Disease Specialist: Given the complexity of the case and the need for individualized treatment decisions, the patient should consult with a specialist, such as a hepatologist or infectious disease physician. They will be able to assess the patient's overall health, the severity of the hepatitis B infection, and any other relevant factors to determine the most appropriate course of action. Remember, this information is not a substitute for professional medical advice. The patient should discuss their specific situation, concerns, and treatment options with a qualified healthcare professional who can provide personalized guidance based on a thorough assessment of the patient's health. | I am not a doctor, but I can provide some general information that might be helpful for you to discuss with a healthcare professional. It's important to consult with a healthcare provider for personalized advice based on the specific details of the individual's medical history and condition. 1.HBeAg Positive: HBeAg positivity indicates active viral replication in hepatitis B. The decision to start antiviral therapy is often based on a combination of factors, including viral load, liver function tests, and the presence of liver inflammation. 2.ALT Level: An elevated ALT level (231 U/L) suggests liver inflammation. Persistent elevation may indicate ongoing liver damage. Treatment decisions are often guided by both ALT levels and other factors such as viral load. 3.Hyperlipidemia: Hyperlipidemia can be a comorbidity that needs to be managed along with hepatitis B. Some antiviral medications may have interactions with lipid-lowering agents, so it's crucial to discuss this with a healthcare provider. 4.Interferon Therapy: Interferon therapy is one of the options for treating chronic hepatitis B. However, its use is less common now due to the availability of oral antiviral medications that are generally better tolerated. The decision to use interferon or other antiviral medications depends on various factors, including the patient's overall health, liver function, viral load, and the presence of cirrhosis. 5.Consultation with a Hepatologist: The patient should consult with a hepatologist or a healthcare provider specializing in liver diseases. They can assess the patient's individual case, conduct further tests if necessary, and recommend an appropriate treatment plan. 6.Regular Monitoring: Regular monitoring of liver function, viral load, and other relevant parameters is essential for managing chronic hepatitis B. This helps in adjusting the treatment plan if needed. Remember, this information is not a substitute for professional medical advice. The patient should seek guidance from a qualified healthcare provider who can provide advice based on a thorough examination and understanding of the individual's health status. | No | No |  | No |
| 94 | Simulated Patients | 一个50岁的男性患者，HBeAg阳性，ALT 231 U/L，有高脂血症，现在未进行抗病毒治疗，前来咨询是否可以使用干扰素。请给出诊疗意见。 | 首先，我不是医生，以下只是一些建议，具体治疗方案应该由专业医生根据患者的详细情况和检查结果来确定。这里提供一些一般性的信息供参考。 患者是乙型肝炎表面抗原（HBeAg）阳性，表明病毒正在复制。ALT（丙氨酸氨基转移酶）水平升高可能表明肝脏受到病毒感染的影响。另外，患者有高脂血症，这也是需要关注和管理的因素。 关于干扰素的使用，干扰素是一种抗病毒药物，通常用于治疗乙型肝炎。然而，干扰素治疗可能伴随一些副作用，包括发热、疲劳、抑郁等，因此医生通常会在患者和医生之间共同权衡治疗的利弊。 在制定治疗方案时，医生可能会考虑以下因素： 1.病毒活动性： HBeAg阳性表明病毒在复制，但还需要考虑其他病毒指标，如病毒载量。 2.肝功能： ALT升高可能反映了肝脏受到病毒感染的影响，医生可能会考虑其他肝功能指标。 3.患者的整体健康状况： 年龄、其他健康问题、药物过敏等都是制定治疗方案时需要考虑的因素。 4.患者的意愿和配合度： 干扰素需要注射，患者需能够接受这种治疗方式，并能够遵循医嘱进行治疗。 由于干扰素治疗可能会有一些副作用，医生可能会考虑其他抗病毒药物，如核苷（酸）类似物（NUC）治疗，这类药物通常口服，患者更容易接受。 因此，建议患者咨询专业肝病医生，根据详细的检查结果和患者的整体情况，制定最合适的治疗方案。 | 在处理这位50岁男性患者的情况时，需要考虑多个因素，包括HBeAg阳性、ALT水平升高、高脂血症等。以下是一些建议： 1.评估肝炎病毒复制水平： 确定患者HBV-DNA的水平，以确定病毒活动程度。这可以通过检测病毒载量来实现。 2.评估肝纤维化和肝硬化的程度： 进行肝功能、肝超声、肝组织活检等检查，以了解肝脏的损害程度和是否存在纤维化或肝硬化。 3.综合考虑患者全身健康状况： 评估患者的全身健康状况，包括其他慢性疾病、心血管疾病、代谢综合征等。 4.考虑抗病毒治疗： 对于HBeAg阳性的患者，尤其是ALT水平升高的情况，抗病毒治疗可能是必要的。一些抗病毒药物，如核苷(tide)类似物（如阿德福韦酯、恩替卡韦）或核苷酸(tide)类似物（如替比夫定），可能被考虑用于抑制病毒复制。 5.谨慎考虑干扰素治疗： 干扰素治疗通常在患者合并明显免疫抑制状态或无法耐受抗病毒药物时考虑。但由于干扰素的不良反应较多，包括发热、疲劳、抑郁等，需要综合考虑患者的全身状况。 6.管理高脂血症： 考虑制定适当的高脂血症管理计划，包括饮食控制、运动、药物治疗等，以减少心血管风险。 7.个体化治疗计划： 最终的治疗计划应该是个体化的，基于患者的具体情况和健康需求。在制定治疗计划时，最好由专业的肝病医生来进行评估和建议。 总体而言，建议患者尽早就医，接受专业医生的评估和建议，以制定合适的治疗计划。 | No | No |  | No |
| 95 | Simulated Patients | A 46-year-old man came to the hospital because of "positive hepatitis B surface antigen detected by physical examination." What tests might he need? | When a 46-year-old man presents with a positive hepatitis B surface antigen (HBsAg) detected during a physical examination, further testing is necessary to assess the extent of the infection and its impact on the liver. Here are some tests that the healthcare provider may order: 1.Hepatitis B Serology: Hepatitis B Surface Antibody (anti-HBs): This test determines if the person has developed immunity to hepatitis B, either through previous infection or vaccination. Hepatitis B Core Antibody (anti-HBc): It helps distinguish between acute and chronic infections. The presence of IgM anti-HBc indicates acute infection, while the presence of IgG anti-HBc suggests past or chronic infection. Hepatitis B e Antigen (HBeAg) and Antibody (anti-HBe): These markers provide information about the infectivity of the virus. Presence of HBeAg indicates active viral replication, while the presence of anti-HBe may suggest a lower level of viral replication. 2.Hepatitis B DNA (Viral Load): Quantitative PCR for HBV DNA: This test measures the amount of hepatitis B virus in the blood, indicating the level of viral replication. It helps in assessing the severity of the infection and monitoring treatment effectiveness. 3.Liver Function Tests: Alanine Aminotransferase (ALT) and Aspartate Aminotransferase (AST): These enzymes are released into the bloodstream when the liver is damaged. Elevated levels may indicate liver inflammation or damage. 4.Liver Imaging: Abdominal Ultrasound or Fibroscan: These imaging studies can help assess the degree of liver damage and fibrosis. 5.Screening for Hepatitis Delta Virus (HDV): Anti-HDV IgM and HDV RNA: HDV is a defective virus that relies on the presence of hepatitis B virus. Co-infection with HDV can worsen liver disease. 6.Assessment for Cirrhosis: Prothrombin Time (PT) and International Normalized Ratio (INR): These tests assess blood clotting function and may indicate the presence of cirrhosis. 7.Evaluation for Other Liver Diseases: Hepatitis C Testing: As co-infection with hepatitis C is not uncommon, it is often screened for. Tests for other causes of liver disease: Depending on clinical suspicion, other tests for liver diseases (such as autoimmune hepatitis or hemochromatosis) may be considered. It's important for the patient to discuss the results and treatment options with their healthcare provider. The management of chronic hepatitis B often involves antiviral medications to suppress viral replication and regular monitoring of liver function. | When a 46-year-old man presents with a positive hepatitis B surface antigen (HBsAg) detected during a physical examination, further testing is necessary to assess the status of the hepatitis B infection and its potential impact on the liver. The following tests may be recommended: 1.Hepatitis B Panel: HBsAg: Confirms the presence of the hepatitis B virus. Anti-HBs (Hepatitis B surface antibody): Indicates immunity to hepatitis B. HBeAg (Hepatitis B e antigen): Presence indicates active viral replication. Anti-HBe (Hepatitis B e antibody): Indicates a decrease in viral replication. 2.Hepatitis B DNA (Viral Load) Test: Measures the amount of hepatitis B virus in the blood, helping to assess the level of viral replication. 3.Liver Function Tests: Alanine Aminotransferase (ALT) and Aspartate Aminotransferase (AST): Assess liver enzyme levels, which may be elevated in hepatitis B infection, indicating liver inflammation or damage. 4.Complete Blood Count (CBC): Checks for abnormalities in blood cell counts that may be associated with liver disease. 5.Liver Imaging: Ultrasound or CT scan: These imaging studies can assess the structure of the liver and identify any signs of cirrhosis or liver tumors. 6.Liver Biopsy (if needed): A biopsy may be recommended to assess the extent of liver damage and to guide treatment decisions. 7.Screening for Co-infections: Testing for other viral infections, such as hepatitis C or HIV, which can impact the course of hepatitis B and affect treatment decisions. 8.Screening for Hepatocellular Carcinoma (Liver Cancer): Regular monitoring for liver cancer, especially in individuals with chronic hepatitis B, may involve imaging studies such as ultrasound and alpha-fetoprotein (AFP) blood tests. It's essential to note that the specific tests ordered may vary based on the patient's medical history, clinical presentation, and the healthcare provider's judgment. The results of these tests will help determine the stage and severity of the hepatitis B infection, guide treatment decisions, and monitor the patient's liver health over time. Consultation with a healthcare professional is crucial for appropriate evaluation and management. | No | No |  | No |
| 95 | Simulated Patients | 一位46岁男性患者，因“体检发现乙肝表面抗原阳性”前来就诊。他可能需要进行何种检查？ | 对于一位46岁男性患者，体检发现乙肝表面抗原（HBsAg）阳性，医生可能会建议进行以下一些检查： 1.乙肝病毒DNA检测： 通过检测患者血液中的乙肝病毒DNA水平，可以评估病毒的活跃程度。 2.肝功能检查： 包括血清谷丙转氨酶（ALT）、谷草转氨酶（AST）、碱性磷酸酶（ALP）等指标，以评估肝脏功能和是否存在炎症或损伤。 3.乙肝e抗原和乙肝e抗体检测： 了解患者是否有乙肝e抗原（HBeAg）阳性，以及是否产生了乙肝e抗体（HBeAb），这有助于判断病毒的活跃程度和传播风险。 4.肝脏B超或CT扫描： 通过影像学检查评估肝脏是否受损、是否存在肝硬化或肝癌等情况。 5.肝穿刺活检： 如果有必要，医生可能会建议进行肝穿刺活检，以获取更详细的肝脏组织信息，判断肝脏病变的类型和程度。 6.丙肝病毒和艾滋病毒检测： 由于乙肝、丙肝和艾滋病毒的传播途径有重叠，因此在诊断和管理过程中可能需要检测其他病毒感染。 这些检查可以帮助医生全面评估患者的病情，制定合适的治疗和管理计划。患者应当咨询专业医生，根据具体情况制定个体化的检查和治疗方案。 | 对于一位46岁男性患者，乙肝表面抗原（HBsAg）阳性的情况，医生可能会建议进行以下一些检查： 1.乙肝相关检查： 乙肝表面抗体（anti-HBs）： 用于判断是否曾经感染过乙肝病毒，并且是否具有免疫保护力。 乙肝e抗原（HBeAg）和乙肝e抗体（anti-HBe）： 用于评估病毒复制活跃性和传染性。 乙肝核心抗体（anti-HBc）： 用于确定感染的历史。 2.肝功能检查： 血清谷丙转氨酶（ALT）和天冬氨酸转氨酶（AST）： 检查肝脏功能是否正常。 总胆红素： 用于评估胆红素代谢情况。 3.乙肝病毒DNA测定： 通过检测血液中的乙肝病毒DNA水平来评估病毒的活跃性和复制水平。 4.肝脏超声检查： 可以检查肝脏是否有结构性的异常，例如肝硬化或肝癌。 5.肝活检： 在一些情况下，医生可能建议进行肝脏活检以获取更详细的肝脏组织信息。 6.临床评估： 医生可能会详细询问患者的病史，了解症状，并进行身体检查，以全面评估患者的健康状况。 这些检查有助于确定患者乙肝感染的状况、评估病毒的活跃性、了解肝功能状态，并采取相应的治疗和管理措施。请注意，最终的诊断和治疗方案应该由专业医生根据具体情况来制定。 | No | No |  | No |
| 96 | Simulated Patients | A 32-year-old woman presented with "abdominal distention and poor appetite". The patient had severe yellowing of the skin and sclera, abdominal distention, and positive shifting dullness. Her mother had hepatitis B without treatment. List tests she might need and give possible diagnoses. | The symptoms described in the case suggest a potential liver-related issue. Given the family history of hepatitis B, there is a concern for viral hepatitis as a possible cause. Here are some tests that the 32-year-old woman might need: 1.Hepatitis B Panel: Hepatitis B surface antigen (HBsAg) Hepatitis B surface antibody (anti-HBs) Hepatitis B e antigen (HBeAg) Hepatitis B e antibody (anti-HBe) Hepatitis B core antibody (anti-HBc) 2.Liver Function Tests: Alanine aminotransferase (ALT) Aspartate aminotransferase (AST) Alkaline phosphatase (ALP) Total bilirubin Direct bilirubin Albumin Prothrombin time (PT) or international normalized ratio (INR) 3.Viral Hepatitis Serology: Hepatitis A antibodies (anti-HAV) Hepatitis C antibodies (anti-HCV) 4.Abdominal Imaging: Abdominal ultrasound or CT scan to assess the liver and identify any signs of cirrhosis, hepatomegaly, or ascites. 5.Additional Tests: Complete blood count (CBC) Serum ferritin and iron studies to rule out hemochromatosis Alpha-fetoprotein (AFP) to assess for liver cancer  Possible Diagnoses based on the presented symptoms and family history: 1.Hepatitis B Infection: Chronic hepatitis B may cause yellowing of the skin (jaundice), abdominal distention, and liver dysfunction. 2.Cirrhosis: Chronic liver damage, often seen in advanced stages of hepatitis B, can lead to cirrhosis, resulting in abdominal distention and liver dysfunction. 3.Liver Cancer (Hepatocellular Carcinoma): Hepatitis B infection increases the risk of developing liver cancer. Jaundice and abdominal distention can be symptoms of advanced liver cancer. 4.Ascites: Accumulation of fluid in the abdominal cavity, often associated with advanced liver disease. It's important for the patient to consult a healthcare professional for a thorough evaluation. The tests and diagnoses mentioned above are indicative and not a substitute for professional medical advice. | The symptoms described in the case suggest potential liver dysfunction, and given the family history of hepatitis B, there is a concern for viral hepatitis. Here are some tests that the 32-year-old woman might need: 1.Hepatitis B Panel: HBsAg (Hepatitis B surface antigen) HBcAb (Hepatitis B core antibody) HBeAg (Hepatitis B e antigen) Anti-HBe (Antibody to Hepatitis B e antigen) Anti-HBs (Antibody to Hepatitis B surface antigen) 2.Liver Function Tests (LFTs): Serum bilirubin (total and direct) Alanine aminotransferase (ALT) Aspartate aminotransferase (AST) Alkaline phosphatase (ALP) Albumin INR (International Normalized Ratio) 3.Complete Blood Count (CBC): To assess for anemia, which can be associated with chronic liver disease. 4.Imaging Studies: Abdominal ultrasound or CT scan to evaluate the liver size, texture, and identify any abnormalities such as cirrhosis or masses. 5.Viral Hepatitis Serology: Hepatitis A antibodies (IgM and IgG) Hepatitis C antibodies Hepatitis D antibodies (if Hepatitis B is confirmed)  Possible Diagnoses: 1.Chronic Hepatitis B: Given the family history and the patient's symptoms, chronic hepatitis B infection is a significant consideration. 2.Hepatitis B-related Cirrhosis: Chronic hepatitis B can progress to cirrhosis, leading to symptoms such as abdominal distention and poor appetite. 3.Hepatocellular Carcinoma (HCC): Chronic hepatitis B infection increases the risk of developing hepatocellular carcinoma. Imaging studies would help evaluate for any liver masses. 4.Other Liver Diseases: Other causes of liver dysfunction, such as autoimmune hepatitis, alcoholic liver disease, or non-alcoholic fatty liver disease, should also be considered and evaluated based on test results. It's important for the patient to consult with a healthcare professional for a thorough evaluation, proper diagnosis, and appropriate management based on the test results and clinical findings. | No | No |  | No |
| 96 | Simulated Patients | 一位32岁女性患者，因“腹胀、胃口差”前来就诊。患者皮肤和巩膜严重发黄，腹部膨隆，移动性浊音阳性。她母亲患有乙肝，没有进行过治疗。她可能需要接受哪些检查？列举并给出可能的诊断。 | 这位患者的症状和家族病史提示可能存在肝脏问题，尤其是与乙型肝炎相关的问题。以下是可能建议的检查以及可能的诊断： 1.血液检查： 乙肝病毒标志物检测： 包括HBsAg（乙肝表面抗原）、Anti-HBc（乙肝核心抗体）等。 肝功能检查： 包括ALT（丙氨酸氨基转移酶）、AST（天冬氨酸氨基转移酶）、总胆红素等指标。 2.影像学检查： 腹部超声： 用于评估肝脏形态、大小以及是否存在结节、囊肿等异常。 CT扫描或MRI： 进一步评估肝脏的结构和功能。 3.肝活检： 在一些情况下，可能需要进行肝脏活检以确定病变的类型和程度。 4.病毒学检查： 检测其他可能的肝病毒感染，如丙型肝炎病毒。  可能的诊断包括： 1.慢性乙型肝炎： 由乙型肝炎病毒引起，与患者母亲的乙肝患病史相关。 2.肝硬化： 由于慢性肝炎引起的肝脏组织瘢痕化。 3.肝癌： 长期的肝炎和肝硬化可能增加患肝癌的风险。  请注意，这只是初步的推测，具体的诊断需要结合患者的详细病史、体格检查和进一步的实验室和影像学检查结果。患者应及时就医，由专业医生根据具体情况制定进一步的诊断和治疗方案。 | 这位32岁女性患者的症状和体征提示可能存在肝脏问题。考虑到她母亲患有乙肝，这使得乙型肝炎成为一个可能的病因。以下是可能需要进行的检查和一些可能的诊断： 1.乙型肝炎病毒检测： 乙型肝炎表面抗原（HBsAg）检测：阳性结果可能表明患者感染了乙型肝炎病毒。 乙型肝炎表面抗体（anti-HBs）检测：有助于区分乙肝的感染和康复阶段。 乙型肝炎e抗原（HBeAg）和抗体（anti-HBe）检测：有助于评估病毒活动性和传播性。 2.肝功能检查： 肝功能指标（AST、ALT、碱性磷酸酶、总胆红素等）：用于评估肝脏功能和是否存在炎症、坏死等情况。 3.影像学检查： 腹部超声：用于评估肝脏形态、大小，检测是否有肿块、胆囊问题等。 4.病毒学检查： 乙肝病毒DNA检测：确定是否存在病毒复制和病毒载量。 5.其他检查： 凝血功能检查：肝脏疾病可能导致凝血功能异常。 血清学检查：抗核抗体（ANA）等，排除自身免疫性肝病。  可能的诊断： 1.慢性乙型肝炎： 肝功能异常，乙型肝炎病毒检测阳性。 2.肝硬化： 腹胀、腹水、移动性浊音阳性。 肝功能异常，腹部超声显示肝脏结构异常。 3.乙肝病毒相关肝硬化或肝癌： 乙型肝炎病毒检测阳性，同时伴随肝硬化或肝癌的表现。  需要强调的是，这只是基于提供的病历信息的一种可能性。最终的诊断应该由专业医生根据详细的临床评估和相关检查结果来确定。如果患者确诊为乙型肝炎或其他肝脏疾病，医生将会制定相应的治疗方案。 | No | No |  | No |
