# Supplementary material for: Assessing ChatGPT as a Medical Consultation Assistant for Chronic Hepatitis B: Cross-Language Study of English and Chinese
Source: JMIR Med Inform. 2024 Aug 8;12:e56426. doi: 10.2196/56426 (PMC11342014; doi:10.2196/56426)
Supplement: Multimedia Appendix 6 [file medinform_v12i1e56426_app6.docx]

**Multimedia Appendix 6**. Responses provided with sufficient emotional management guidance in ChatGPT-3.5.

| Number | Classicfication | Question | Response1 | Response2 | Emotional support1 | Emotional support2 |
| --- | --- | --- | --- | --- | --- | --- |
| 5 | Clinical Problems | Give clinical treatment advice for women of childbearing age infected with hepatitis B | The treatment of hepatitis B in women of childbearing age depends on several factors, such as the level of viral replication, the degree of liver damage, and the woman's pregnancy status. Here are some general treatment recommendations: Antiviral medications: Antiviral medications can help to reduce the replication of the hepatitis B virus in the body, which can help to prevent liver damage. Some antiviral medications that may be used include entecavir, tenofovir disoproxil fumarate (TDF), or tenofovir alafenamide (TAF). These medications are generally safe to use during pregnancy. Monitoring liver function: Women with chronic hepatitis B should have regular monitoring of their liver function through blood tests. This can help to detect any signs of liver damage and ensure that appropriate treatment is provided. Vaccination: If a woman is not already immune to hepatitis B, she should be vaccinated against the virus. Vaccination is safe to receive during pregnancy and can help to prevent transmission of the virus to the newborn. Counseling: Women with chronic hepatitis B should receive counseling about the risk of transmission of the virus to their partners and newborns. They should also be advised to avoid alcohol and other liver-damaging substances. Pregnancy planning: Women with chronic hepatitis B who are planning to become pregnant should be evaluated by their healthcare provider to ensure that their liver function is stable and that they are receiving appropriate treatment. Women who are pregnant should receive regular monitoring of their hepatitis B virus levels and liver function. It's important to note that the management of hepatitis B during pregnancy can be complex and should be done in consultation with a healthcare provider with experience in managing viral hepatitis. | Women of childbearing age who are infected with hepatitis B should receive prompt medical care and treatment to reduce the risk of transmitting the virus to their newborns. Here are some general clinical treatment advice: Antiviral medications: Antiviral medications such as tenofovir or entecavir can be used to suppress the replication of the hepatitis B virus and reduce the risk of liver damage. These medications are generally safe and effective for pregnant women with chronic hepatitis B. Monitoring liver function: Regular monitoring of liver function is recommended during pregnancy, particularly in women with chronic hepatitis B. This helps to detect any signs of liver damage or inflammation, which may require additional treatment. Hepatitis B immune globulin (HBIG): HBIG is a medication that contains antibodies against the hepatitis B virus. It is typically given to newborns of mothers with chronic hepatitis B within 12 hours of birth, along with the hepatitis B vaccine, to reduce the risk of transmission. Hepatitis B vaccine: The hepatitis B vaccine is recommended for all infants and children, as well as adults who are at increased risk of infection. It is safe and effective, and can provide long-term protection against the hepatitis B virus. Counseling and support: Women who are infected with hepatitis B may benefit from counseling and support to help them cope with the emotional and psychological effects of the disease. They may also need guidance on how to prevent transmission of the virus to others, including their sexual partners and household members. It's important to note that treatment for hepatitis B during pregnancy should be individualized based on the woman's medical history and the severity of her condition. Women with chronic hepatitis B should be closely monitored by a healthcare provider throughout their pregnancy and after delivery to ensure the best possible outcomes for both mother and baby. | 2 | 1 |
| 24 | Clinical Problems | 知道自己乙肝后应该怎么办 | 如果你知道自己患有乙肝，建议你采取以下措施： 寻求医生的帮助：乙肝是一种慢性疾病，需要专业医生进行治疗和管理。建议你尽快寻求医生的帮助，进行详细的检查和诊断，以便制定适当的治疗计划。 遵守医嘱：医生会根据你的身体状况和乙肝病毒的活动程度，制定适当的治疗计划。你需要严格遵守医生的建议，按时服药或接受其他治疗，以保持病情稳定。 注重饮食：乙肝患者需要注重饮食，特别是应该避免摄入过多的脂肪和糖分，增加蛋白质的摄入量，有助于维持身体的健康和免疫力。 健康生活方式：保持健康的生活方式可以帮助乙肝患者降低病情加重的风险，包括戒烟限酒，适量运动，保持充足的睡眠等。 注意传播途径：乙肝是一种可以通过血液、体液等途径传播的疾病，因此要注意避免与他人分享个人用品、器具等，并注意性生活安全，避免传染给他人。 | 如果你发现自己感染了乙肝病毒，应该采取以下步骤： 1.寻求医疗帮助：首先，你需要寻求医疗帮助，咨询医生或专业医疗机构的医生，了解自己感染的乙肝病毒类型、病情和治疗方法。 2.接受检查：你需要进行一系列检查，包括肝功能、病毒量、肝脏超声等，以了解肝脏状况和病毒感染情况。 3.接受治疗：如果你的病情需要治疗，你应该接受专业医生的治疗建议和方案。目前有多种乙肝病毒治疗方案，包括抗病毒治疗、免疫调节剂等。 4.采取预防措施：为了防止乙肝病毒的传播，你需要采取措施，如不与他人共用个人卫生用品，不接触他人的血液和体液，避免性接触等。 5.注意饮食和生活：在治疗期间，你需要注意饮食和生活方式，保持身体健康，避免过度饮酒和疲劳。 需要注意的是，乙肝病毒感染是一种可以控制和治疗的疾病，及早发现和治疗可以提高治疗成功的机会。如果你感到焦虑或有其他的情绪问题，你也可以考虑寻求心理健康咨询师的帮助。 | 2 | 1 |
| 24 | Clinical Problems | What should I do after learning that I have hepatitis B? | If you have been diagnosed with hepatitis B, it is important to take action to manage the condition and protect your health as well as the health of others. Here are some steps you can take: 1.Consult with a healthcare provider: It is important to speak with a healthcare provider who can evaluate your condition and provide guidance on how to manage your hepatitis B. Your healthcare provider may recommend additional tests to determine the extent of liver damage and help determine the appropriate course of treatment. 2.Follow recommended treatment plan: Depending on the severity of your hepatitis B, your healthcare provider may recommend antiviral medication to slow or stop the virus from causing further damage to your liver. It is important to follow your recommended treatment plan as directed. 3.Get vaccinated: If you have not already been vaccinated against hepatitis B, it is important to get vaccinated to protect yourself from additional infections. The vaccine is safe and effective. 4.Practice safe sex: Hepatitis B can be spread through sexual contact, so it is important to practice safe sex by using condoms and limiting the number of sexual partners. 5.Avoid sharing personal items: Hepatitis B can also be spread through sharing personal items such as razors, toothbrushes, or needles. It is important to avoid sharing these items with others to prevent the spread of the virus. 6.Educate yourself and others: Learn more about hepatitis B and how it is spread. Share this information with your loved ones to help prevent the spread of the virus. Remember, with proper management and care, people with hepatitis B can lead healthy and productive lives. | If you have been diagnosed with hepatitis B, it is important to take the following steps: Seek medical treatment: Hepatitis B is a serious liver infection that requires medical attention. You should see a doctor who specializes in liver diseases, such as a gastroenterologist or a hepatologist. Your doctor may prescribe antiviral medications to help manage the infection and prevent liver damage. Follow your doctor's advice: Your doctor may recommend lifestyle changes to help manage your hepatitis B infection. This may include avoiding alcohol and certain medications that can harm the liver, getting regular checkups to monitor your liver function, and getting vaccinated against hepatitis A and B. Inform your sexual partner and family members: Hepatitis B is contagious and can be spread through blood and bodily fluids. It is important to inform your sexual partner and family members so they can get tested and vaccinated if necessary. Practice good hygiene: Hepatitis B can be spread through contact with infected blood or bodily fluids. To reduce your risk of spreading the infection to others, practice good hygiene by washing your hands frequently and avoiding sharing personal items like razors and toothbrushes. Join a support group: Living with a chronic condition like hepatitis B can be challenging. Joining a support group can help you connect with others who are going through a similar experience and provide you with emotional support. Remember that hepatitis B is a manageable condition with the right medical care and lifestyle changes. It is important to follow your doctor's advice and take steps to protect your health and the health of others. | 2 | 1 |
| 32 | Clinical Problems | My dad need a liver transplant due to acute hepatitis b, he is 55. What happens if he isn't in good enough health for a transplant? Do they just send him home to die? | I'm sorry to hear that your dad is facing this difficult health issue. In cases where a liver transplant is deemed too risky due to the patient's overall health or other medical conditions, the medical team will work with the patient and their family to develop a treatment plan that can provide the best possible quality of life. This might include medications, dietary changes, and other interventions that can help manage the symptoms and complications of acute hepatitis B. In some cases, the patient may be referred to hospice care or other supportive care programs that can provide emotional and practical support during this difficult time. It's important to keep in mind that every case is unique, and the medical team will work closely with your dad and his family to determine the best course of action based on his individual health status and medical history. It's also worth noting that liver transplants can be very successful in many cases, and your dad's medical team will likely explore all possible options to see if a transplant is a viable option for him. | I'm sorry to hear about your dad's health condition. Generally, eligibility for a liver transplant is determined based on various medical factors, including the severity of the liver disease, overall health, and other medical conditions. If your dad's health is not good enough for a transplant, it may be because he has other medical conditions that make the procedure too risky, or because his liver disease is too advanced to benefit from a transplant. In such cases, the doctors will work with your dad and your family to explore other treatment options that may help manage his symptoms and prolong his life. These options may include medications to help manage the symptoms of his liver disease, such as ascites and encephalopathy, or procedures to drain excess fluid from his abdomen. The doctors may also recommend lifestyle changes, such as a low-salt diet, to help manage his symptoms and improve his overall health. Ultimately, the goal of medical care in such cases is to provide comfort and improve quality of life, while managing the symptoms of the underlying disease. Hospice care may also be an option if your dad's health continues to decline, and the focus of care shifts to managing pain and ensuring a peaceful and comfortable end-of-life experience. It's important to have open and honest communication with your dad's healthcare team, so that you can fully understand his treatment options and make informed decisions about his care. | 1 | 1 |
| 41 | Simulated Patients | 一个62岁的男性患者，HBeAg阳性，HBsAg 10^4 IU/mL，没有肝硬化，ALT 25 U/L，有高血压，未进行抗病毒治疗。请从肝病治疗和健康管理的角度，给他提供治疗和日常管理方面的建议。 | 这位患者的情况涉及到慢性乙型肝炎（Chronic Hepatitis B，CHB），需要综合考虑肝病治疗和相关的健康管理方面的建议。请注意，以下建议仅供参考，具体的治疗和管理决策应由专业医生根据患者的具体情况进行制定。 1. 肝病治疗： 由于患者是HBeAg阳性，血清HBV-DNA水平较高，建议考虑进行抗病毒治疗，以减少病毒复制和防止疾病的进展。常用的抗病毒药物包括去氨酸阿德福韦（adefovir）、恩替卡韦（entecavir）、替比夫定（tenofovir）等。 治疗的目标是通过抑制病毒复制，降低血清HBV-DNA水平，改善肝功能，并降低患者发展肝硬化和肝癌的风险。 2. 健康管理和生活方式： 控制高血压：积极管理高血压，采取药物治疗和生活方式干预，如饮食控制、体育锻炼、戒烟等，以降低心血管疾病风险。 定期监测肝功能：通过定期检查ALT、AST、肝功能等指标，评估肝脏健康状况，及时发现问题并采取相应的干预措施。 饮食控制：建议患者采取低脂、低盐、高纤维的饮食，避免酗酒，以减轻肝脏负担。 防止传播：患者应采取预防措施，以防止HBV传播给他人。接受家庭成员的疫苗接种，避免共用个人卫生用品。 3. 定期随访： 患者应该定期进行随访，监测病情变化，调整治疗方案。 随访时医生可能会根据病情调整药物剂量或更换药物，确保最佳的治疗效果。 总体而言，这位患者需要一个综合的治疗和管理计划，涵盖抗病毒治疗、高血压管理、饮食控制以及定期随访。治疗方案应该由专业的医生制定，根据患者的具体情况进行调整。在整个治疗过程中，患者需要密切配合医生的建议，保持良好的生活方式和定期的随访。 | 对于这位62岁的男性患者，他是HBeAg阳性，HBsAg水平较高，但ALT水平正常，没有肝硬化，同时有高血压。以下是一些建议，但请注意这只是一般性建议，具体治疗方案应该由专业医生根据病人的具体情况来确定。 肝病治疗建议： 1.评估病毒活动性： 进一步检查HBV DNA水平，以确定病毒活动性的程度。 2.抗病毒治疗： 对于HBeAg阳性的患者，抗病毒治疗是必要的。常用的药物包括拜瑞那韦（Entecavir）或替比夫定（Tenofovir）。 抗病毒治疗有助于抑制病毒复制，减缓肝脏病变进程。 3.定期随访： 定期监测HBV DNA、ALT、AST等指标，以及肝功能的变化。 考虑进行肝脏超声等影像学检查，监测肝脏病变的发展。 健康管理建议： 1.高血压管理： 控制血压在目标范围内，以减轻心脏和肾脏的负担。 遵循医生的建议，按时服用抗高血压药物。 2.生活方式调整： 避免饮酒，因为酒精可能对肝脏产生不良影响。 保持健康的饮食，避免高脂肪、高糖和高盐饮食。 进行适度的体育锻炼，但要遵循医生的建议。 3.疫苗接种： 接种乙肝疫苗，以防止其他人感染。 4.医学监测： 定期进行肝功能检查、血液检查以及其他相关检查，确保早期发现和处理任何潜在的问题。 5.药物相互作用： 注意药物相互作用，特别是抗病毒药物和其他药物之间的相互影响。 6.心理健康： 维持良好的心理健康，可以考虑参与支持团体或与医疗团队沟通，以解决可能的情绪和心理压力。 总体而言，制定适当的治疗方案和生活方式调整计划需要在专业医生的指导下进行。请务必咨询医生，以制定适合该患者独特情况的个体化计划。 | 2 | 1 |
