# Supplementary material for: Assessing ChatGPT as a Medical Consultation Assistant for Chronic Hepatitis B: Cross-Language Study of English and Chinese
Source: JMIR Med Inform. 2024 Aug 8;12:e56426. doi: 10.2196/56426 (PMC11342014; doi:10.2196/56426)
Supplement: Multimedia Appendix 8 [file medinform_v12i1e56426_app8.docx]

**Multimedia Appendix 8.** Responses mentioning “I am not a doctor” or “I cannot give diagnosis or treatment” in ChatGPT-3.5.

| Number | Classicfication | Question | Response1 | Response2 | Mention1 | Mention2 |
| --- | --- | --- | --- | --- | --- | --- |
| 73 | Clinical Problems | 想去亚洲旅行，查到推荐打甲肝和乙肝疫苗，但时间只够打一针，打甲肝、乙肝、还是混合针最好？ | 如果您只有时间打一针疫苗，我建议您首先考虑接种乙肝疫苗。以下是一些原因： 1.乙肝病毒在亚洲地区比甲肝更为普遍，因此感染乙肝的风险更高。 2.乙肝是一种慢性病毒感染，会导致肝硬化和肝癌等严重并发症。甲肝通常是一种自限性疾病，大多数人可以自愈。 3.接种乙肝疫苗的效果比接种甲肝疫苗更好。根据疾病控制和预防中心的建议，接种乙肝疫苗需要完成三剂次，而接种甲肝疫苗只需要完成两剂次。 4.如果您有足够的时间，可以考虑接种混合疫苗，其中包括甲肝、乙肝和其他一些疫苗。但是，如果您只有时间打一针，那么我建议您优先考虑接种乙肝疫苗。 请注意，我是一名语言模型，不能替代医生或疫苗专家的建议。建议您咨询医生或专业人士的意见，以确定最适合您的疫苗接种方案。 | 首先，建议您咨询医生或专业的旅行医学机构，以获得最准确和个性化的建议，因为接种疫苗的选择可能会受到您的个人健康状况、旅行计划和目的地的影响。 然而，就一般情况而言，如果您只有时间接种一针疫苗，建议您优先选择乙肝疫苗，因为乙肝是亚洲地区最常见的传染病之一。甲肝虽然也较为常见，但其病情通常较轻，可以通过饮食和卫生措施预防，因此如果时间和预算允许，最好也可以考虑接种甲肝疫苗。 如果您的时间非常有限，而且您预计在旅行期间会接触到多种食物和饮料，您可以考虑接种混合疫苗，如甲乙混合疫苗或甲乙丙混合疫苗，以获得更全面的保护。但是请注意，混合疫苗的价格通常更高，而且可能会增加副作用的风险。 | Yes | No |
| 75 | Clinical Problems | I have positive HBsAg, HBeAg and HBcAb with a high HBV-DNA level, but my liver function is normal, should I take antiviral drugs? | As a language model, I cannot provide medical advice. However, generally speaking, if you have tested positive for HBsAg, HBeAg, and HBcAb with a high HBV-DNA level, this indicates that you have a hepatitis B virus infection. The decision to start antiviral treatment will depend on a number of factors, including the level of HBV-DNA, the severity of liver damage, and the presence of any other medical conditions. It is important that you speak to your healthcare provider about your test results and discuss your treatment options. They will be able to provide you with more information about your condition and recommend the best course of treatment for you based on your individual medical history and current condition. | If you have tested positive for HBsAg, HBeAg, and HBcAb, and have a high HBV-DNA level, it indicates that you are infected with hepatitis B virus (HBV) and are in the replicative phase of the disease. While your liver function tests are normal now, it does not guarantee that your liver will remain unaffected in the future. Antiviral treatment is recommended for patients with chronic hepatitis B who have high viral loads, especially those with HBeAg positivity. The goal of antiviral therapy is to suppress HBV replication, prevent liver damage, and decrease the risk of developing complications such as cirrhosis and liver cancer. Therefore, it is important to consult with a healthcare provider who specializes in the treatment of viral hepatitis, who will evaluate your case and recommend an appropriate antiviral therapy, if required. It is also important to regularly monitor your liver function and viral load to ensure that the treatment is working and to detect any potential complications at an early stage. | Yes | No |
| 81 | AASLD Guideline questions | 免疫耐受感染的成人是否应接受抗病毒治疗以减少肝脏相关并发症？ | 我不是医生，也不能提供具体的医学建议。但是，免疫耐受感染的成人是否应接受抗病毒治疗通常取决于多种因素，包括感染的类型、严重程度、个体的健康状况以及其他潜在风险因素。 免疫耐受感染通常指人体对某些病毒（如乙型肝炎病毒）产生了免疫反应，但病毒仍然存在于体内。在这种情况下，医生可能会考虑多种因素，以确定是否需要进行抗病毒治疗。 乙型肝炎是一种常见的免疫耐受感染情况，有时可能需要抗病毒治疗以防止肝脏相关并发症，如肝硬化和肝癌的发生。然而，治疗决策通常需要个体化，根据患者的具体情况进行。 如果你或有人对乙型肝炎或其他病毒感染有疑虑，最好咨询专业医生。医生将能够评估你的健康状况，进行必要的检查，并为你制定最合适的治疗方案。 | 我不是医生，不能提供具体的医疗建议。然而，如果成年人具有免疫耐受感染，特别是涉及肝脏的感染，例如乙型肝炎病毒（HBV）或丙型肝炎病毒（HCV），医生可能会考虑是否需要抗病毒治疗。这通常基于多种因素，包括病毒载量、肝功能、肝纤维化程度、患者的一般健康状况以及其他个体因素。 抗病毒治疗的目标可能是减缓疾病的进展、预防肝硬化和肝癌的发生，以及改善生活质量。对于HBV感染，抗病毒治疗可以帮助降低病毒载量，减少肝脏损伤。对于HCV感染，抗病毒治疗通常能够实现病毒持续性清除，从而预防肝炎的长期并发症。 最终的决定应由专业医疗团队根据患者的具体情况做出。如果您或您认识的人面临这种情况，请咨询医生以获取详细的诊断和治疗建议。 | Yes | Yes |
| 86 | AASLD Guideline questions | 有低病毒血症的代偿期肝硬化患者是否应接受抗病毒药物治疗？ | 我不是医生，但我可以提供一些一般性的信息。对于乙肝患者是否应该接受抗病毒药物治疗，最好的建议是咨询专业医生，因为医生能够根据患者的具体情况做出最合适的决定。 一般来说，治疗决策取决于多个因素，包括患者的病毒载量、肝功能、肝脏纤维化或硬化的程度，以及是否存在其他健康问题。对于代偿期乙肝患者，即肝脏功能在正常范围内的患者，医生可能会根据病毒载量和其他指标来评估是否需要抗病毒治疗。 一些专家组织和指南建议，对于代偿期患者，如果存在高病毒载量、肝功能异常或肝脏纤维化程度较高的情况，可能需要考虑抗病毒治疗，以减缓疾病进展和降低肝硬化、肝癌等并发症的风险。 总之，乙肝治疗的决策是一个复杂的过程，应该由专业医生根据患者的具体情况进行评估。如果您或您认识的人患有乙肝，建议咨询医生以获取专业建议。 | 我不是医生，但我可以提供一些一般性的信息供您参考。对于乙肝肝硬化患者是否应接受抗病毒药物治疗，最好的建议是咨询专业医生，因为医疗决策应该基于个体病情和医生的专业意见。 一般来说，治疗乙肝的决定取决于多个因素，包括病毒活动水平、肝脏炎症和纤维化程度、患者的年龄、性别、肝功能以及患者的整体健康状况。 对于乙肝患者，低病毒血症并不一定表示没有病毒活动。即使在低病毒血症的情况下，患者的肝脏仍然可能受到损害，导致肝硬化的发展。因此，医生可能会考虑抗病毒药物治疗以抑制病毒复制，减缓或阻止肝脏的病变。 再次强调，最终的决策应由医生根据患者的具体情况做出。如果您或您认识的人有乙肝相关问题，建议寻求专业医生的建议和治疗。 | Yes | Yes |
| 87 | AASLD Guideline questions | 高病毒载量的乙型肝炎表面抗原阳性孕妇是否应在妊娠晚期接受抗病毒治疗以预防HBV的围产期传播？ | 我不是医生，因此不能提供具体的医学建议。然而，我可以向您提供一些一般性的信息。 乙型肝炎病毒（HBV）是一种可以通过血液和其他体液传播的病毒，而孕妇如果感染了HBV，有可能将病毒传给新生儿。高病毒载量的乙型肝炎表面抗原（HBsAg）阳性是一种孕妇可能会传播给胎儿的风险因素。 抗病毒治疗是否适合孕妇需要根据具体情况来判断，包括孕妇的病毒载量、肝功能状况、孕期、医学历史等因素。对于乙肝携带者的孕妇，医生可能会根据孕妇的具体情况来评估是否需要进行抗病毒治疗，以降低围产期传播的风险。 如果您或您认识的人是乙型肝炎表面抗原阳性的孕妇，建议咨询专业医生的意见。医生将能够根据具体情况制定适当的治疗计划，并权衡治疗的利弊，以确保母婴的健康。 | 我不是医生，但我可以提供一些一般性的信息。乙型肝炎（HBV）是一种由乙型肝炎病毒引起的病毒性感染。对于怀孕的女性，特别是在妊娠晚期，如果她们是高病毒载量的乙型肝炎表面抗原（HBsAg）阳性，医生可能会考虑采取一些预防措施，以减少新生儿HBV感染的风险。 一般而言，垂直传播是新生儿感染HBV的主要途径。在这种情况下，医生可能会考虑采取以下预防措施： 1.乙肝免疫球蛋白（HBIG）注射： 在新生儿出生后的12小时内，可以给予HBIG注射，以提供即时 passively 获得的被动免疫，减少HBV传播的风险。 2.乙肝疫苗接种： 新生儿应该在出生后尽早接种乙肝疫苗，这样可以提高他们对乙型肝炎的主动免疫水平。 此外，医生还可能根据具体情况考虑给予抗病毒药物，以降低母体病毒载量，减少传播的风险。然而，对于是否使用抗病毒药物以及具体使用哪种药物，应该由专业的医疗团队评估患者的个体情况，并根据权衡利弊作出决策。 如果你或者你认识的人有类似的情况，强烈建议咨询专业医生的建议，他们能够提供个性化的医疗建议和治疗方案。 | Yes | Yes |
| 88 | AASLD Guideline questions | 阳性慢性乙型病毒性肝炎患儿是否应接受抗病毒治疗以减少肝脏相关并发症？ | 乙型病毒性肝炎（HBV）是一种由乙型肝炎病毒（HBV）引起的慢性病毒感染。对于阳性慢性乙型病毒性肝炎患儿是否应接受抗病毒治疗，通常需要综合考虑多个因素，包括病毒活动性、肝功能、肝组织病理学改变、患者的年龄和全身状况等。 一般而言，对于患有慢性乙型病毒性肝炎的患者，抗病毒治疗的目标是降低病毒载量、减缓肝脏病变进程，从而减少肝脏相关并发症的发生，例如肝硬化和肝癌。国际上通常采用的治疗指南（例如美国肝病学会和欧洲肝脏病学会的指南）提供了相应的治疗建议。 在考虑治疗时，医生可能会考虑以下一些因素： 1.病毒活动性： 根据病毒载量、HBV DNA水平以及肝功能等指标来评估病毒活动性。 2.肝功能： 评估肝功能的严重程度，包括肝酶水平和肝功能测试。 3.肝组织病理学： 通过肝活检等手段评估肝脏的病理学改变，以确定是否存在显著的炎症和纤维化。 4.年龄和全身状况： 考虑患者的年龄、全身状况以及患者是否有其他健康问题。 5.治疗禁忌症： 考虑是否存在任何禁忌症，例如对抗病毒药物的过敏反应。 在制定治疗方案时，医生会根据患者的具体情况权衡治疗的利弊。对于一些患者，可能会推荐开始抗病毒治疗，而对于另一些患者，可能会选择定期监测并延迟治疗。 因此，如果你或你认识的人患有慢性乙型病毒性肝炎，建议咨询专业的医生或肝脏病专家，以便根据具体情况制定最合适的治疗方案。医生会根据患者的情况制定个性化的治疗计划。 | 我不是医生，因此不能提供专业医学建议。但一般来说，对于慢性乙型病毒性肝炎的患者，是否需要接受抗病毒治疗应该由医生根据患者的具体情况来决定。以下是一些通常考虑的因素： 1.病毒活动性： 医生通常会评估乙型肝炎病毒的活动程度。如果病毒活动性较高，可能会倾向于开始抗病毒治疗，以减少病毒复制并防止肝脏进一步受损。 2.肝功能： 医生会评估患者的肝功能，包括肝酶水平和其他指标。如果肝功能受损，可能需要考虑抗病毒治疗以减轻肝脏负担。 3.肝纤维化和肝硬化： 如果患者已经出现了肝纤维化或肝硬化的迹象，可能需要更积极地干预，以防止疾病进展。 4.患者的整体健康状况： 医生还会考虑患者的整体健康状况，包括其他慢性病、免疫状态等因素。 请务必咨询专业医生，以获取针对个体情况的建议。他们可以评估患者的病历、实验室结果和其他相关信息，并制定最合适的治疗方案。 | No | Yes |
| 89 | Simulated Patients | A 62-year-old male patient, HBeAg positive, HBsAg 10^4 IU/mL, without cirrhosis, ALT 25 U/L, with hypertension, was not on antiviral therapy. Please provide him with advice on therapy and daily monitor and management from the perspective of liver disease treatment and health management. | I am not a medical professional, but I can provide you with some general information that might be helpful. However, it's crucial to consult with a healthcare professional for advice tailored to the specific situation of the patient. The information provided here may not be up-to-date or applicable to individual cases. Given the patient's profile, it seems like he has chronic hepatitis B (CHB) with a positive HBeAg and a moderate viral load. The management of chronic hepatitis B typically involves antiviral therapy to suppress the viral replication and reduce the risk of liver complications. Here are some general considerations for therapy and management: 1.Antiviral Therapy: Patients with chronic hepatitis B and elevated viral load, like in this case, may be candidates for antiviral therapy. Consult with a hepatologist or gastroenterologist to determine the most appropriate antiviral medication based on the patient's overall health, potential drug interactions, and guidelines in force at the time. Common antiviral medications for hepatitis B include entecavir, tenofovir disoproxil fumarate (TDF), and tenofovir alafenamide (TAF). 2.Monitoring: Regular monitoring is essential to assess the effectiveness of treatment and detect any potential side effects. Liver function tests (including ALT levels) should be monitored regularly to evaluate liver health. Viral load and serological markers (such as HBsAg and HBeAg) should be checked periodically to assess the response to antiviral therapy. 3.Liver Disease Management: Management of comorbidities, such as hypertension, is crucial. Blood pressure should be monitored regularly, and appropriate medications may be prescribed to control hypertension. Lifestyle modifications, including a healthy diet, regular exercise, and abstaining from alcohol, can contribute to overall liver health. 1.Vaccination: Ensure that the patient is vaccinated against hepatitis A and B if not already immune to these viruses. 2.Regular Follow-Up: Schedule regular follow-up appointments with the healthcare provider to adjust the treatment plan as needed and monitor the patient's overall health. It's important to note that this information is general and may not cover all aspects of the patient's unique situation. The healthcare provider overseeing the patient's care will make the final decisions regarding therapy and management based on the specific circumstances of the case. | I am not a doctor, but I can offer some general information that might help guide the discussion with a healthcare professional. It's important for the patient to consult with a healthcare provider for personalized advice based on their specific health condition. Given the provided information, it appears that the patient is HBeAg positive, indicating active viral replication in the hepatitis B virus (HBV). The HBsAg level is 10^4 IU/mL, which suggests a moderate to high viral load. The ALT level is within the normal range, indicating that the liver enzyme levels are not elevated at the moment. The patient also has hypertension. Treatment decisions for chronic hepatitis B are complex and depend on various factors, including liver function, viral load, and the presence of other medical conditions. The guidelines for managing chronic hepatitis B may vary, and it's crucial for the patient to consult with a hepatologist or infectious disease specialist for a personalized treatment plan. Here are some general considerations for the patient: 1.Consult with a Specialist: The patient should schedule an appointment with a hepatologist or an infectious disease specialist who has expertise in managing chronic hepatitis B. 2.Antiviral Therapy: Based on current guidelines, antiviral therapy may be considered for HBeAg-positive patients with a high viral load. Commonly prescribed antiviral medications include tenofovir or entecavir. The decision to start antiviral therapy should be made by the healthcare provider after considering the patient's overall health and potential side effects of the medications. 3.Regular Monitoring: Regular follow-up visits and monitoring are essential to assess the response to treatment, liver function, and the progression of the disease. This may involve periodic blood tests, including liver function tests and viral load measurements. 4.Management of Hypertension: Given the presence of hypertension, the patient should also receive appropriate management for blood pressure control. Lifestyle modifications, such as a healthy diet and regular exercise, may be recommended in addition to any prescribed medications. 5.Vaccination for Hepatitis A and B: If the patient has not been previously vaccinated for hepatitis A and B, vaccination should be considered to prevent additional liver-related complications. 6.Avoidance of Alcohol and Hepatotoxic Substances: The patient should be advised to avoid alcohol and substances that can be harmful to the liver. It's important for the patient to communicate openly with their healthcare provider, adhere to the recommended treatment plan, and make lifestyle modifications to promote overall liver health. Additionally, regular monitoring and follow-up visits are crucial for managing chronic hepatitis B effectively. | Yes | Yes |
| 90 | Simulated Patients | 一个51岁的男性患者，HBeAg阴性，HBsAg 10^1 IU/mL，没有肝硬化，ALT 28 U/L，未进行抗病毒治疗，平时有吸烟习惯。他是否需要应该进行抗病毒治疗，理由是什么？ | 首先，我不是医生，因此我的回答仅供参考，具体的医疗决策应由专业医生根据详细的患者状况进行。在这种情况下，这位男性患者的HBeAg阴性、HBsAg为10^1 IU/mL、ALT正常，且没有肝硬化的情况下，通常不是立即进行抗病毒治疗的迹象。 当前的临床指南一般是根据乙型肝炎病毒（HBV）感染的肝功能、病毒复制水平、肝纤维化等因素来判断是否需要抗病毒治疗。在这个患者的情况下，ALT正常，表示肝脏目前没有活跃的炎症，而HBeAg阴性通常表示病毒复制相对较低。此外，缺乏肝硬化的证据也是一个积极的因素。 然而，吸烟是一个可能影响肝炎患者病程的不良因素。吸烟可能导致肝炎的进展，增加肝癌的风险。因此，医生可能会建议患者改变生活方式，戒烟以及保持健康的生活习惯。 最好的做法是咨询专业医生，他们将根据患者的个体情况、实验室检查结果和最新的治疗指南为患者提供最合适的治疗建议。 | 我不是医生，但我可以提供一些一般性的信息。对于慢性乙型肝炎（HBV）的治疗决策通常需要综合考虑多个因素，并且最好由专业医生进行评估。在这种情况下，需要考虑的因素包括患者的病毒载量、肝功能、肝组织病理学评估、家庭和个体史，以及是否存在合并症等。 在提供具体建议之前，建议您咨询专业医生，进行全面的体格检查和相关检测。通常情况下，对于HBeAg阴性、HBsAg阳性的慢性HBV感染，医生可能会考虑以下一些建议： 1.HBV DNA水平： 患者的病毒载量（HBV DNA水平）是一个重要的指标。如果病毒载量较高，可能需要考虑抗病毒治疗。 2.肝功能： ALT水平正常是一个好的指标，但仍需要考虑其他肝功能指标，以确保肝脏正常。 3.肝组织病理学评估： 肝活检可能有助于评估肝脏的病理学情况，了解是否存在肝纤维化或炎症。 4.合并症： 是否存在其他合并症，如肝硬化、肝细胞癌等，也会影响治疗的决策。 5.年龄和生活方式： 患者的年龄、吸烟史等因素也可能影响治疗决策。 总体而言，决定是否进行抗病毒治疗应该是个体化的，并且需要医生的综合评估。因此，建议您尽快咨询专业医生，以获取准确的建议和治疗方案。 | Yes | Yes |
| 90 | Simulated Patients | A 51-year-old male patient, HBeAg-negative, HBsAg 10^1 IU/mL, without cirrhosis, ALT 28 U/L, was not on antiviral therapy and was an habitual smoker. Does he need antiviral treatment and why? | I am not a doctor, but I can provide some general information that might be helpful for you to discuss with a healthcare professional. In this case, the patient is HBeAg-negative, has a low HBsAg level, normal ALT (alanine transaminase), and no cirrhosis. The decision to start antiviral treatment in chronic hepatitis B depends on various factors, and it's crucial to consult with a healthcare provider for personalized advice. Here are some considerations: 1.HBV DNA Levels: While you provided the HBsAg level, information about the HBV DNA viral load is essential. The decision to start antiviral treatment often considers the level of viral replication in the blood. 2.Liver Function: The ALT level is normal, suggesting the liver is not currently inflamed. However, periodic monitoring is necessary to assess any changes over time. 3.Smoking: Smoking is a risk factor for liver disease progression. It's important for the patient to address lifestyle factors, such as smoking, to improve overall health. 4.Age and Other Health Conditions: The patient's age may be a factor in the decision-making process. Other health conditions, such as diabetes or obesity, may also influence the decision. 5.Guidelines: Treatment decisions are often guided by established medical guidelines. Healthcare providers may consider guidelines from organizations like the American Association for the Study of Liver Diseases (AASLD) or the European Association for the Study of the Liver (EASL). 6.Patient Preferences: The patient's preferences, concerns, and willingness to adhere to long-term treatment are important factors in the decision-making process. It's crucial for the patient to have a detailed discussion with a healthcare provider who can assess the individual's overall health, review test results, and consider various factors to determine the most appropriate course of action. Antiviral therapy may be recommended to suppress viral replication, prevent liver damage, and reduce the risk of complications, but this decision should be made on a case-by-case basis. | I am not a medical professional, but I can provide some general information based on the details you've provided. It's crucial to consult with a healthcare professional for personalized advice tailored to the specific situation. The decision to start antiviral treatment for hepatitis B (HBV) is complex and depends on various factors, including liver function, viral load, and the presence of other medical conditions. Here are some considerations: 1.HBV DNA (viral load): The patient's HBV DNA level is an important factor in determining the need for treatment. An HBsAg level of 10^1 IU/mL suggests a moderate viral load. High viral loads are associated with an increased risk of disease progression and may influence the decision to start antiviral therapy. 2.Liver enzymes (ALT): A normal ALT level (28 U/L in this case) is generally a positive indicator, suggesting that the liver is not currently experiencing significant inflammation or damage. However, it's essential to consider trends in ALT levels over time. 3.Liver cirrhosis: The absence of cirrhosis is a positive factor, as cirrhosis is a more advanced stage of liver disease and may influence the decision to initiate treatment. 4.HBeAg-negative status: The patient is HBeAg-negative, which may indicate a transition to a less active phase of the infection. However, other factors must be considered in the overall assessment. 5.Smoking habit: Smoking can contribute to overall health issues, including liver health. Addressing lifestyle factors, such as smoking cessation, is generally beneficial for overall well-being. It's crucial for the patient to consult with a hepatologist or a healthcare professional experienced in managing hepatitis B. They will consider the overall clinical picture, including the factors mentioned above, and may order additional tests if needed. The decision to start antiviral treatment is typically individualized, and healthcare providers consider the potential risks and benefits for each patient. Please note that guidelines for the management of hepatitis B may vary, and the information provided here is not a substitute for professional medical advice. | Yes | Yes |
| 92 | Simulated Patients | A 36-year-old female patient with HBV-DNA 10^6 IU/ml, HBeAg positive, uncertain whether she had cirrhosis, eGFR 109 ml/min, ALT 119 U/L, BMI 30.2kg/m^2, diabetes mellitus, had been treated with entecavir for 1 year. How should this patient conduct daily monitoring and lifestyle management? | I am not a medical professional, but I can offer some general suggestions based on the information provided. It's important for the patient to consult with their healthcare provider for personalized advice. 1. Medication Adherence: Continue taking entecavir as prescribed by the healthcare provider. 2. Monitoring: Regular monitoring of liver function tests (ALT, AST) to assess the response to treatment and liver health. Periodic measurement of HBV-DNA levels to evaluate the effectiveness of antiviral therapy. Imaging studies or liver biopsy may be considered to assess cirrhosis status. 3. Lifestyle Management: Diet and Exercise: Maintain a healthy diet and engage in regular physical activity to manage BMI and promote overall health. Weight loss, if needed, can positively impact liver health. Blood Sugar Control: Given the presence of diabetes mellitus, it's crucial to manage blood glucose levels through medication, diet, and exercise. Alcohol Avoidance: As the patient has liver disease, avoiding alcohol is essential to prevent further damage. Regular Follow-up: Schedule regular follow-up appointments with the healthcare provider to monitor overall health and adjust the treatment plan if necessary. 4. Vaccination: Ensure up-to-date vaccinations, including hepatitis A and hepatitis B vaccines if not already received. 5. Assessing Cirrhosis: Further evaluation for cirrhosis may be necessary, such as imaging studies (ultrasound, FibroScan) or a liver biopsy. 6. Managing Comorbidities: Address and manage other comorbidities, such as diabetes, through appropriate medications, lifestyle modifications, and regular monitoring. 7. Kidney Function Monitoring: Regularly monitor kidney function, especially considering the patient's eGFR. Adjustments to medication may be needed if kidney function changes. 8. Consultation with Specialists: Collaborate with hepatologists, endocrinologists, and other specialists as needed for comprehensive care. It's crucial for the patient to maintain open communication with their healthcare team, adhere to prescribed medications, and make lifestyle modifications to manage overall health effectively. This information is for general guidance, and the patient's specific circumstances may warrant individualized recommendations from their healthcare provider. | I am not a doctor, but I can offer some general information that might be helpful for you to discuss with a healthcare professional. It's important for the patient to have regular follow-ups with a healthcare provider for proper management of chronic conditions like hepatitis B, diabetes, and obesity. Here are some general suggestions: 1.Hepatitis B Monitoring: Regular follow-ups with a hepatologist or infectious disease specialist are crucial. Monitoring of HBV-DNA levels, liver function tests (including ALT levels), and HBeAg status may be necessary to assess the response to entecavir therapy. 2.Liver Imaging: Consider liver imaging studies (such as ultrasound, FibroScan, or MRI) to assess the degree of liver fibrosis or cirrhosis. 3.Diabetes Management: Strict management of diabetes is essential. Regular monitoring of blood glucose levels and HbA1c is important. Lifestyle modifications, including a healthy diet and regular exercise, can help control diabetes and manage weight. 4.Weight Management: Achieving and maintaining a healthy weight is crucial, especially with a BMI of 30.2 kg/m^2. Weight loss can help improve liver function and reduce the risk of complications. A balanced diet with a focus on fruits, vegetables, whole grains, and lean proteins is recommended. 5.Kidney Function Monitoring: Regular monitoring of kidney function is important, especially with an eGFR of 109 ml/min. This can include regular testing of serum creatinine and other kidney function markers. 6.Regular Exercise: Engaging in regular physical activity is beneficial for both diabetes management and weight control. Consult with a healthcare provider before starting a new exercise program. 7.Alcohol Avoidance: Given the liver condition, it is advisable to avoid or limit alcohol consumption. 8.Medication Adherence: Ensure strict adherence to the prescribed entecavir treatment regimen. 9.Vaccination: Confirm that the patient is up-to-date on hepatitis B vaccinations and other recommended vaccinations. It's crucial for the patient to work closely with their healthcare team to develop an individualized care plan based on their specific medical history and current condition. This information is general in nature and not a substitute for professional medical advice. Always consult with a healthcare provider for personalized guidance. | Yes | Yes |
| 93 | Simulated Patients | 一个44岁的女性患者，HBV-DNA 10^8 IU/ml，HBeAg阳性，没有肝硬化，eGFR 105 ml/min，ALT 110 U/L，BMI 22.5kg/m^2，有高血压病史，有吸烟和饮酒习惯，未进行抗病毒治疗。请给出用药方案和生活方式建议。 | 首先，需要说明我不是医生，不能提供专业医疗建议。针对HBV感染的治疗，最好咨询专业医生以获取个性化的建议。以下是一般性的信息，仅供参考。 对于这位患者，考虑到她的HBeAg阳性，高血压，吸烟和饮酒习惯，以及ALT水平升高，可能需要综合治疗的方法，包括抗病毒治疗、生活方式干预和相关药物治疗高血压。 1.抗病毒治疗： 针对患者的HBV-DNA水平，可能需要考虑抗病毒治疗。抗病毒药物的选择应由专业医生决定，通常会考虑核苷(tide)或核酸酸(tide)类药物，如阿德福韦酯或替比夫定。治疗的目标是抑制病毒复制，减缓疾病的进展。 2.生活方式建议： 戒烟戒酒：吸烟和饮酒都会对肝脏健康产生不良影响，对于HBV感染的患者来说更是如此。尽量戒烟和限制饮酒。 饮食和运动：保持健康的饮食习惯，避免高脂肪、高糖分的食物。适量的锻炼对于整体健康有益，但在开始运动之前最好咨询医生的建议。 控制体重：保持适当的体重对于控制高血压和改善肝脏健康都很重要。 3.管理高血压： 考虑高血压病史，可能需要药物治疗。抗高血压药物的选择应该由医生根据患者的具体情况来确定。 4.监测： 定期进行检查，包括HBV病毒载量、肝功能、肾功能和血压监测等，以便及时调整治疗方案。 请记住，以上建议是一般性的，具体治疗方案需要根据患者的详细病史和体检结果由专业医生来制定。患者应该密切关注医生的建议，定期进行复查，并积极参与治疗过程。 | 请注意，我不是医生，以下只是一些建议，具体的治疗方案应该由专业医生根据详细的临床情况进行制定。对于慢性乙型肝炎（HBV）的治疗，建议您咨询专业医生以获取最准确的建议。 根据提供的信息，您的患者属于慢性HBV感染，目前HBV-DNA水平较高，HBeAg阳性，ALT升高，但无肝硬化。以下是可能的治疗方案和生活方式建议： 1. 抗病毒治疗方案： 抗病毒药物： 对于HBV患者，抗病毒治疗是关键。一些常用的抗病毒药物包括： Nucleos(t)ide analogs (NAs)： 如恩替卡韦（Entecavir）、替比夫定（Tenofovir）等。这些药物可以有效抑制病毒复制，减少炎症和改善肝功能。 2. 生活方式建议： 戒烟和限制饮酒： 戒烟和限制饮酒是非常重要的。吸烟和过量饮酒都会对肝脏健康产生负面影响，尤其是在慢性乙型肝炎的情况下。 控制体重： 维持健康的体重对于肝脏健康至关重要。尽量保持适当的体重，避免肥胖。 健康饮食： 采用均衡的饮食，多摄取新鲜水果、蔬菜、全谷物和低脂蛋白质，有助于支持免疫系统和维持肝脏功能。 控制高血压： 由于患者有高血压病史，需要积极控制血压，可能需要药物治疗，同时也可以通过生活方式改变，如控制钠盐摄入、增加锻炼等。 3. 定期监测： 定期检查肝功能： 包括ALT、AST等指标，以监测肝脏炎症的程度。 HBV-DNA检测： 定期检测HBV-DNA水平，以评估病毒复制的情况。 血压监测： 定期监测血压，确保血压在合适范围内。 请务必在制定治疗方案之前与专业医生咨询。这些建议仅供参考，具体治疗方案需要根据患者的具体病情和体征由医生来确定。 | Yes | Yes |
| 93 | Simulated Patients | A 44-year-old female patient with HBV-DNA 10^8 IU/ml, HBeAg positive, no cirrhosis, eGFR 105 ml/min, ALT 110 U/L, BMI 22.5kg/m^2, a history of hypertension, smoking and drinking habits, and no antiviral therapy. Please advise the medication regimen and lifestyle of the patient. | I am not a doctor, and it's crucial to consult with a healthcare professional for personalized medical advice. However, I can provide some general information based on the details you've provided. Given the patient's clinical profile with chronic hepatitis B (CHB), it's important to consider antiviral therapy to manage the infection and prevent disease progression. The decision to start antiviral treatment should be made by a healthcare professional after a thorough assessment of the patient's medical history, laboratory results, and overall health. In the case of HBeAg-positive CHB with a high viral load, one of the commonly used antiviral medications is oral antiviral therapy with nucleoside or nucleotide analogs. Medications like tenofovir or entecavir are often considered as first-line treatment options. The choice of medication should be based on factors such as drug resistance profiles, potential side effects, and the patient's overall health. Lifestyle modifications are also essential for managing chronic hepatitis B. Here are some general recommendations: 1.Alcohol and Smoking Cessation: Strongly advise the patient to quit smoking and limit alcohol consumption. Both smoking and excessive alcohol intake can exacerbate liver damage. 2.Diet and Weight Management: Encourage a healthy, well-balanced diet to maintain a healthy weight. Consider consultation with a dietitian for personalized dietary recommendations. 3.Blood Pressure Control: Monitor and manage hypertension to reduce the risk of cardiovascular complications. 4.Regular Exercise: Promote regular physical activity, as it contributes to overall health. 5.Regular Follow-up and Monitoring: Schedule regular follow-up appointments with healthcare providers for monitoring liver function, viral load, and other relevant parameters. 6.Vaccination: Ensure the patient is up-to-date on vaccinations, including hepatitis A and B. It's crucial for the patient to adhere to the prescribed medication regimen and attend regular follow-up appointments. The healthcare provider will assess the patient's response to treatment, monitor liver function, and make adjustments to the treatment plan if necessary. This information is general, and individual cases may vary. The patient should consult with their healthcare provider for specific guidance tailored to their health status and needs. | I am not a medical professional, and it's important to consult with a healthcare provider for personalized advice. However, based on the information provided, here are some general considerations:  Medication Regimen: Given that the patient has chronic hepatitis B (CHB) with a high viral load (HBV-DNA 10^8 IU/ml) and is HBeAg positive, antiviral therapy may be considered. Commonly used antiviral medications for chronic hepatitis B include: 1.Nucleos(t)ide Analogues (NAs): Tenofovir disoproxil fumarate (TDF) or Tenofovir alafenamide (TAF): These are potent antiviral drugs commonly used for CHB. OR Entecavir: Another effective antiviral option for CHB. The choice between these medications should be based on various factors, including patient preference, potential side effects, and individual health considerations. The decision to start antiviral therapy should be made in consultation with a hepatologist or gastroenterologist.  Monitoring: Regular monitoring of liver function tests, including ALT, and HBV-DNA levels, is essential. HBeAg status and liver imaging may be monitored periodically.  Lifestyle Recommendations: 1.Alcohol Consumption: Given the history of drinking habits, reducing or eliminating alcohol consumption is crucial. Chronic alcohol use can exacerbate liver damage. 2.Smoking Cessation: Quitting smoking is highly recommended. Smoking can contribute to the progression of liver disease and overall health issues. 3.Blood Pressure Management: Given the history of hypertension, it's important to manage blood pressure through lifestyle modifications and, if necessary, medication. 4.Healthy Diet and Weight Management: Encourage a well-balanced diet with an emphasis on fruits, vegetables, and whole grains. Maintain a healthy BMI. 5.Regular Exercise: Engage in regular physical activity as tolerated, considering the patient's overall health status. 6.Regular Follow-up: Regularly follow up with healthcare providers to monitor liver function, viral load, and overall health. It's crucial for the patient to work closely with a healthcare team, including a hepatologist or gastroenterologist, to tailor the management plan based on individual needs and any specific contraindications. Additionally, lifestyle changes should be discussed with the patient's primary care physician or specialists as needed. | Yes | Yes |
| 94 | Simulated Patients | 一个50岁的男性患者，HBeAg阳性，ALT 231 U/L，有高脂血症，现在未进行抗病毒治疗，前来咨询是否可以使用干扰素。请给出诊疗意见。 | 首先，我不是医生，以下只是一些建议，具体治疗方案应该由专业医生根据患者的详细情况和检查结果来确定。这里提供一些一般性的信息供参考。 患者是乙型肝炎表面抗原（HBeAg）阳性，表明病毒正在复制。ALT（丙氨酸氨基转移酶）水平升高可能表明肝脏受到病毒感染的影响。另外，患者有高脂血症，这也是需要关注和管理的因素。 关于干扰素的使用，干扰素是一种抗病毒药物，通常用于治疗乙型肝炎。然而，干扰素治疗可能伴随一些副作用，包括发热、疲劳、抑郁等，因此医生通常会在患者和医生之间共同权衡治疗的利弊。 在制定治疗方案时，医生可能会考虑以下因素： 1.病毒活动性： HBeAg阳性表明病毒在复制，但还需要考虑其他病毒指标，如病毒载量。 2.肝功能： ALT升高可能反映了肝脏受到病毒感染的影响，医生可能会考虑其他肝功能指标。 3.患者的整体健康状况： 年龄、其他健康问题、药物过敏等都是制定治疗方案时需要考虑的因素。 4.患者的意愿和配合度： 干扰素需要注射，患者需能够接受这种治疗方式，并能够遵循医嘱进行治疗。 由于干扰素治疗可能会有一些副作用，医生可能会考虑其他抗病毒药物，如核苷（酸）类似物（NUC）治疗，这类药物通常口服，患者更容易接受。 因此，建议患者咨询专业肝病医生，根据详细的检查结果和患者的整体情况，制定最合适的治疗方案。 | 在处理这位50岁男性患者的情况时，需要考虑多个因素，包括HBeAg阳性、ALT水平升高、高脂血症等。以下是一些建议： 1.评估肝炎病毒复制水平： 确定患者HBV-DNA的水平，以确定病毒活动程度。这可以通过检测病毒载量来实现。 2.评估肝纤维化和肝硬化的程度： 进行肝功能、肝超声、肝组织活检等检查，以了解肝脏的损害程度和是否存在纤维化或肝硬化。 3.综合考虑患者全身健康状况： 评估患者的全身健康状况，包括其他慢性疾病、心血管疾病、代谢综合征等。 4.考虑抗病毒治疗： 对于HBeAg阳性的患者，尤其是ALT水平升高的情况，抗病毒治疗可能是必要的。一些抗病毒药物，如核苷(tide)类似物（如阿德福韦酯、恩替卡韦）或核苷酸(tide)类似物（如替比夫定），可能被考虑用于抑制病毒复制。 5.谨慎考虑干扰素治疗： 干扰素治疗通常在患者合并明显免疫抑制状态或无法耐受抗病毒药物时考虑。但由于干扰素的不良反应较多，包括发热、疲劳、抑郁等，需要综合考虑患者的全身状况。 6.管理高脂血症： 考虑制定适当的高脂血症管理计划，包括饮食控制、运动、药物治疗等，以减少心血管风险。 7.个体化治疗计划： 最终的治疗计划应该是个体化的，基于患者的具体情况和健康需求。在制定治疗计划时，最好由专业的肝病医生来进行评估和建议。 总体而言，建议患者尽早就医，接受专业医生的评估和建议，以制定合适的治疗计划。 | Yes | No |
| 94 | Simulated Patients | on antiviral therapy, came to console whether interferon could be used. Please give medical advice. | I am not a doctor, but I can provide some general information. It's important for the patient to consult with a healthcare professional for personalized advice based on their specific medical history and condition. That being said, here are some considerations: 1.HBeAg-Positive Status: The patient being HBeAg positive suggests active viral replication. This is a factor that healthcare professionals consider when deciding on antiviral therapy. 2.ALT Level: The elevated ALT (alanine aminotransferase) level indicates liver inflammation. High ALT levels are often associated with hepatitis B infection, but the specific level alone may not be the only factor to determine the need for treatment. 3.Hyperlipidemia: The presence of hyperlipidemia should also be taken into account, as it may affect overall health and complicate treatment decisions. 4.Interferon Therapy: Interferon therapy has been used in the treatment of hepatitis B, but its use can be limited by side effects and the availability of newer, more effective antiviral medications. Interferon is not typically the first-line treatment for chronic hepatitis B, and its use is often considered in specific cases. 5.Antiviral Therapy Options: Newer antiviral medications, such as nucleoside or nucleotide analogs (e.g., entecavir, tenofovir), are often preferred for the treatment of chronic hepatitis B due to their efficacy and tolerability. 6.Consultation with a Hepatologist or Infectious Disease Specialist: Given the complexity of the case and the need for individualized treatment decisions, the patient should consult with a specialist, such as a hepatologist or infectious disease physician. They will be able to assess the patient's overall health, the severity of the hepatitis B infection, and any other relevant factors to determine the most appropriate course of action. Remember, this information is not a substitute for professional medical advice. The patient should discuss their specific situation, concerns, and treatment options with a qualified healthcare professional who can provide personalized guidance based on a thorough assessment of the patient's health. | I am not a doctor, but I can provide some general information that might be helpful for you to discuss with a healthcare professional. It's important to consult with a healthcare provider for personalized advice based on the specific details of the individual's medical history and condition. 1.HBeAg Positive: HBeAg positivity indicates active viral replication in hepatitis B. The decision to start antiviral therapy is often based on a combination of factors, including viral load, liver function tests, and the presence of liver inflammation. 2.ALT Level: An elevated ALT level (231 U/L) suggests liver inflammation. Persistent elevation may indicate ongoing liver damage. Treatment decisions are often guided by both ALT levels and other factors such as viral load. 3.Hyperlipidemia: Hyperlipidemia can be a comorbidity that needs to be managed along with hepatitis B. Some antiviral medications may have interactions with lipid-lowering agents, so it's crucial to discuss this with a healthcare provider. 4.Interferon Therapy: Interferon therapy is one of the options for treating chronic hepatitis B. However, its use is less common now due to the availability of oral antiviral medications that are generally better tolerated. The decision to use interferon or other antiviral medications depends on various factors, including the patient's overall health, liver function, viral load, and the presence of cirrhosis. 5.Consultation with a Hepatologist: The patient should consult with a hepatologist or a healthcare provider specializing in liver diseases. They can assess the patient's individual case, conduct further tests if necessary, and recommend an appropriate treatment plan. 6.Regular Monitoring: Regular monitoring of liver function, viral load, and other relevant parameters is essential for managing chronic hepatitis B. This helps in adjusting the treatment plan if needed. Remember, this information is not a substitute for professional medical advice. The patient should seek guidance from a qualified healthcare provider who can provide advice based on a thorough examination and understanding of the individual's health status. | Yes | Yes |
