# Supplementary material for: Assessing ChatGPT as a Medical Consultation Assistant for Chronic Hepatitis B: Cross-Language Study of English and Chinese
Source: JMIR Med Inform. 2024 Aug 8;12:e56426. doi: 10.2196/56426 (PMC11342014; doi:10.2196/56426)
Supplement: Multimedia Appendix 9 [file medinform_v12i1e56426_app9.docx]

**Multimedia Appendix 9.** Summary of the information accuracy grades and consistency of ChatGPT-4.0.

|  |  | Chinese | | | | English | | | | Total | | | |
| --- | --- | --- | --- | --- | --- | --- | --- | --- | --- | --- | --- | --- | --- |
| Assessment Content | Grades | Clinical Problems | AASLD Guideline | Simulated Patients | Total | Clinical Problems | AASLD Guideline | Simulated Patients | Total | Clinical Problems | AASLD Guideline | Simulated Patients | Total |
|  |  |  |  |  |  |  |  |  |  |  |  |  |  |
| Information Accuracy | 1 | 64 (82.05%) | 10 (55.56%) | 8 (50.00%) | 82(73.21%) | 69 (90.79%) | 12 (66.67%) | 9 (56.25%) | 90 (81.82%) | 133 (86.36%) | 22 (61.11%) | 17 (53.13%) | 172 (77.48%) |
|  | 2 | 14 (30.77%) | 8 (44.44%) | 8 (50.00%) | 30 (26.79%) | 7 (9.21%) | 6 (33.337%) | 7 (43.75%) | 20 (18.18%) | 21 (13.64%) | 14 (38.89%) | 15 (46.88%) | 50 (22.52%) |
|  | 3 | 0 (0.00%) | 0 (0.00%) | 0 (0.00%) | 0 (0.00%) | 0 (0.00%) | 0 (0.00%) | 0 (0.00%) | 0 (0.00%) | 0 (0.00%) | 0 (0.00%) | 0 (0.00%) | 0 (0.00%) |
|  | 4 | 0 (0.00%) | 0 (0.00%) | 0 (0.00%) | 0 (0.00%) | 0 (0.00%) | 0 (0.00%) | 0 (0.00%) | 0 (0.00%) | 0 (0.00%) | 0 (0.00%) | 0 (0.00%) | 0 (0.00%) |
|  | Total of Grades | 78 | 18 | 16 | 112 | 76 | 18 | 16 | 110 | 154 | 36 | 32 | 222 |
|  | P | 0.1249^a^ | | | | | | | | 0.1142^b^ | 0.6836 | 0.7232 | <0.0001^c^ |
| Consistency | Consistent  responses | 15 (38.46%) | 4 (44.44%) | 2 (25.00.00%) | 21 (37.50%) | 22 (57.89%) | 3 (33.33%) | 3 (37.50.00%) | 28 (50.91%) | 37 (48.05%) | 7 (38.89%) | 5 (31.25%) | 49 (44.14%) |
|  | Inconsistent  responses | 24 | 5 | 6 | 35 | 16 | 6 | 5 | 27 | 40 | 11 | 11 | 62 |
|  | Total responses | 39 | 9 | 8 | 56 | 38 | 9 | 8 | 55 | 77 | 18 | 16 | 111 |
|  | P | 0.1549^d^ | | | | | | | | 0.0879^e^ | >0.9999 | >0.9999 | 0.4153^f^ |

^a^Indicating the P value of the difference between grades in the two languages.

^b^Indicating the P value of the different grades between the two languages in the section Term Explanation.

^c^Indicating the P value of the difference in grades across the sections.

^d^Indicating the P value of the difference between consistency in the two languages.

^e^Indicating the P value of the different consistency between the two languages in the section Term Explanation.

^f^Indicating the P value of the difference in consistency across the sections.
