# Supplementary material for: Assessing ChatGPT as a Medical Consultation Assistant for Chronic Hepatitis B: Cross-Language Study of English and Chinese
Source: JMIR Med Inform. 2024 Aug 8;12:e56426. doi: 10.2196/56426 (PMC11342014; doi:10.2196/56426)
Supplement: Multimedia Appendix 10 [file medinform_v12i1e56426_app10.docx]

**Multimedia Appendix 10.** Assessment of the information accuracy of ChatGPT-4.0.

| Number | Classicfication | Question | Response 1 | Response 2 | Reviewer1 | Reviewer2 | Reviewer3 | Grade |
| --- | --- | --- | --- | --- | --- | --- | --- | --- |
| 40 | Clinical Problems | How are HBV infectious, cirrhosis and hepatocellular carcinoma related? | Hepatitis B virus (HBV) infection, cirrhosis, and hepatocellular carcinoma (HCC) are interrelated in a number of ways, forming a progression of disease that can lead to serious health consequences.  Here’s how they are related:  1.HBV Infection: Hepatitis B is a viral infection that attacks the liver and can cause both acute and chronic diseases. The virus is transmitted through contact with the blood or other body fluids of an infected person. It is a major global health problem and the most serious type of viral hepatitis. It can cause chronic liver disease and puts people at high risk of death from cirrhosis of the liver and liver cancer.  2.Cirrhosis: Chronic HBV infection can lead to liver damage and cirrhosis over time. Cirrhosis is a late stage of scarring (fibrosis) of the liver caused by many forms of liver diseases and conditions, such as hepatitis and chronic alcoholism. The liver damage caused by cirrhosis generally can’t be undone, but if liver cirrhosis is diagnosed early and the cause is treated, further damage can be limited and, rarely, reversed.  3.Hepatocellular Carcinoma (HCC): This is the most common type of primary liver cancer. It occurs most often in people with chronic liver diseases, such as cirrhosis caused by hepatitis B or hepatitis C infection. Thus, the progression from HBV infection to cirrhosis and finally to HCC is a common pathway.  The relationship can be summarized as follows:  $ \text{HBV Infection} \rightarrow \text{Cirrhosis} \rightarrow \text{Hepatocellular Carcinoma (HCC)} $  It’s important to note that not everyone with HBV will develop cirrhosis or HCC. Factors such as the duration of infection, the age at which a person became infected, and other health conditions can influence disease progression. Moreover, early detection and treatment can help prevent the progression of the disease. | Hepatitis B Virus (HBV) infection, cirrhosis, and hepatocellular carcinoma (HCC) are interrelated in a sequential manner, contributing to the progression of liver disease.  1.HBV Infection: Hepatitis B is a viral infection that attacks the liver and can cause both acute and chronic diseases. It is a major global health problem and the most serious type of viral hepatitis. It can cause chronic liver disease and puts people at high risk of death from cirrhosis of the liver and liver cancer.  2.Cirrhosis: Chronic HBV infection can lead to cirrhosis, a late stage of scarring (fibrosis) of the liver caused by many forms of liver diseases and conditions, such as hepatitis and chronic alcoholism. In cirrhosis, the liver cells are replaced by scar tissue which obstructs the flow of blood through the liver, impairing liver function. Cirrhosis itself can lead to other complications, including liver cancer.  3.Hepatocellular Carcinoma (HCC): HCC is the most common type of primary liver cancer, and it often occurs in people with chronic liver diseases, such as cirrhosis caused by hepatitis B infection. The process of cirrhosis from chronic HBV infection changes the liver’s architecture and function, leading to an environment in which aberrant cells can proliferate and eventually form a cancerous tumor.  In summary, HBV infection can cause chronic liver disease, leading to cirrhosis. The cirrhotic liver is then a fertile ground for the development of HCC. This is why the HBV vaccine is so important - it’s a cancer prevention vaccine. The World Health Organization (WHO) recommends that all infants receive the HBV vaccine as soon as possible after birth, preferably within 24 hours. The low coverage of HBV vaccine in some regions of the world explains the high incidence of HBV-related HCC in these regions. | 1, 1 | 1, 1 |  | 1, 1 |
| 40 | Clinical Problems | 乙肝感染与肝硬化、肝癌有何关系？ | 乙型肝炎病毒（HBV）是全球最常见的病毒感染之一，可以导致肝脏疾病的各种形式，包括急性和慢性肝炎、肝硬化以及肝癌。  以下是乙肝感染与肝硬化、肝癌之间的关系：  1.乙肝与肝硬化：乙型肝炎病毒（HBV）感染可引起肝脏的炎症反应。如果感染持续存在，就会导致肝脏长期受到损害，进而出现肝纤维化，最终可能发展为肝硬化。肝硬化是指肝脏组织发生广泛坏死，被纤维组织替代并形成假性肝小叶，导致肝脏结构和功能的严重障碍。肝硬化的进展过程可能需要数年甚至数十年。  2.乙肝与肝癌：乙型肝炎病毒（HBV）感染是肝癌的主要风险因素之一。长期的乙肝感染会导致肝脏的持续炎症和损伤，这可能会导致肝细胞的基因突变，从而增加肝癌的风险。此外，由乙肝引起的肝硬化也是导致肝癌的重要途径。据估计，乙肝感染者中，约15-40%的人会发展为肝硬化或肝癌。  因此，预防和及早治疗乙型肝炎病毒（HBV）感染是防止肝硬化和肝癌的关键。 | 乙型肝炎病毒（HBV）是一种常见的病毒，可以引发急性和慢性肝病。乙肝病毒感染后，大部分成年人的免疫系统能够有效地清除病毒并恢复健康，但小部分人可能会发展成慢性感染。慢性乙型肝炎的患者有更高的风险发展为肝硬化和肝癌。  1.乙肝与肝硬化的关系： 慢性乙型肝炎的患者，如果不进行有效的治疗，病毒会持续攻击肝脏，引发肝炎，随着时间的推移，肝脏会出现炎症和纤维化，最终可能导致肝硬化。肝硬化是一种病理状态，肝脏组织遭受持续损伤后，被大量瘢痕组织所替代，导致肝脏硬化并失去正常功能。  2.乙肝与肝癌的关系： 慢性乙型肝炎和肝硬化是肝细胞癌（HCC）的主要风险因素。长期的炎症和纤维化可能会导致肝脏细胞发生异常，这些异常细胞有可能会发展成肝癌。此外，乙型肝炎病毒可以整合进宿主基因组，这可能导致基因突变和肝细胞的恶性转化。  因此，对于乙肝病毒感染者，特别是慢性感染者，进行定期检查和适当的抗病毒治疗是非常重要的，以防止疾病的进一步发展。 | 2,2 | 2,2 |  | 2,2 |
| 41 | Clinical Problems | Give advice on usual tests for hepatitis B virus patients | Hepatitis B is a viral infection that attacks the liver. It can cause both acute and chronic diseases. To diagnose, monitor, and manage Hepatitis B, various tests are usually performed.  Here are some of the most common ones:  1.Hepatitis B Surface Antigen (HBsAg): This test is used to screen for, diagnose, and monitor treatment of an HBV infection. It is usually the first test to become positive after exposure to HBV.  2.Hepatitis B Surface Antibody (anti-HBs): This test is used to determine if a person is immune to Hepatitis B, either due to past infection or vaccination.  3.Hepatitis B Core Antibody (anti-HBc): This test is used to help determine if a person is currently infected or has been previously infected with HBV.  4.Hepatitis B e Antigen (HBeAg): This test is used to determine if the virus is actively replicating, which may indicate a high level of infectivity.  5.Hepatitis B e Antibody (anti-HBe): The presence of this antibody usually indicates lower levels of HBV and less infectivity.  6.Hepatitis B Virus DNA: This test is used to measure the amount of HBV DNA in the blood. It helps to determine the severity of the infection and to monitor the effectiveness of treatment.  7.Liver function tests (LFTs): These tests are used to assess the liver’s health and function. They include tests for enzymes such as alanine aminotransferase (ALT) and aspartate aminotransferase (AST), which can indicate liver damage if their levels are elevated.  8.Liver biopsy: In some cases, a liver biopsy may be performed to assess the degree of liver damage or to monitor the progress of treatment.  9.Genotype testing: This test is used to identify the specific strain of HBV, which can affect disease severity, progression, and treatment response.  It’s crucial to remember that the interpretation of these tests should be done by a healthcare professional, as the results can be complex and often need to be considered in the context of the patient’s overall health and medical history. Furthermore, regular follow-ups are important for patients with Hepatitis B to monitor their liver health and to adjust treatment if necessary. | Hepatitis B is a viral infection that attacks the liver and can cause both acute and chronic disease. The virus is transmitted through contact with the blood or other body fluids of an infected person.  Diagnosis of Hepatitis B usually requires several tests because the virus’s behavior is complex. Here are some common tests for Hepatitis B:  1.Hepatitis B Surface Antigen (HBsAg): This test is used to detect the presence of the Hepatitis B virus in your blood. If this test is positive, it indicates that you’re infectious and can spread the virus to others.  2.Hepatitis B Surface Antibody (Anti-HBs): This test is used to determine if you’re immune to the Hepatitis B virus. A positive test indicates that you’re either immune due to successful vaccination or you’ve recovered from a past infection.  3.Hepatitis B Core Antibody (Anti-HBc): This test is used to detect a previous or ongoing infection with the Hepatitis B virus in your blood. A positive test indicates past or present infection.  4.Hepatitis B e Antigen (HBeAg): This test is used to monitor the infectivity of the Hepatitis B virus. A positive test indicates high infectivity.  5.Hepatitis B e Antibody (Anti-HBe): A positive test typically indicates lower infectivity.  6.Hepatitis B DNA: This test is used to measure the amount of Hepatitis B virus in your blood (viral load). A higher viral load often correlates with greater infectivity.  7.Liver function tests: These tests assess the liver’s health and function by measuring levels of proteins, liver enzymes, and bilirubin in your blood. They help to determine the extent of liver damage or inflammation.  8.Liver biopsy: In some cases, a doctor may recommend a liver biopsy to check for liver damage or cancer.  9.Imaging tests: Ultrasound, CT, or MRI can show liver damage.  10.Genotype test: This test identifies the strain of the virus, which can affect disease severity, treatment options, and outcome.  These tests are typically used in combination to provide a complete picture of a patient’s Hepatitis B infection. It’s important to note that these tests should be done under the supervision of a healthcare provider. The results can be complex and may require expert interpretation. Also, it’s important to have regular monitoring and follow-up tests if you’re a Hepatitis B patient, to track the progress of the disease and the effectiveness of treatment. | 1,1 | 2,1 | 1,1 | 1, 1 |
| 41 | Clinical Problems | 给出乙肝常规检查项目的建议 | 乙肝（Hepatitis B）是一种由乙型肝炎病毒（HBV）引起的疾病。对于乙肝的检查，一般包括以下几项：  1.乙型肝炎病毒表面抗原（HBsAg）：这是最早也是最常用来检测乙型肝炎感染的标志物。  2.乙型肝炎病毒表面抗体（anti-HBs）：这是对HBsAg的免疫反应，如果检测结果为阳性，可能表示患者已经恢复或者接种过疫苗。  3.乙型肝炎病毒e抗原（HBeAg）和乙型肝炎病毒e抗体（anti-HBe）：这两个指标可以反映病毒的复制情况和传染性。  4.乙型肝炎病毒核心抗体（anti-HBc）：这是反映乙型肝炎病毒感染历史的一个重要指标。  5.乙型肝炎病毒DNA定量（HBV DNA）：这是评估病毒复制水平和判断疾病活动性的重要指标。  6,肝功能检查：包括丙氨酸氨基转移酶（ALT）、天冬氨酸氨基转移酶（AST）、总胆红素（TBIL）、直接胆红素（DBIL）、间接胆红素（IBIL）、血清白蛋白（ALB）、凝血酶原时间（PT）等，这些都可以反映肝脏的功能状态。  7.肝硬度测定（FibroScan）：这是一种无创的检查方法，可以评估肝纤维化和肝硬化的程度。  以上就是乙肝常规检查项目的建议，具体的检查项目和频率应由医生根据患者的具体情况来决定。 | 乙肝（Hepatitis B）是由乙型肝炎病毒（HBV）引起的一种肝炎，主要通过血液、性接触以及母婴传播。为了了解乙肝的感染情况，以下是一些常规检查项目的建议：  1.HBsAg（乙型肝炎表面抗原）：这是乙肝病毒感染的首要标志。阳性结果可能表示慢性或急性的HBV感染。  2.HBsAb（乙型肝炎表面抗体）：这是体内对乙型肝炎病毒的免疫反应。阳性结果可能表示病人已经恢复并对病毒产生了免疫，或者已经接种了乙肝疫苗。  3.HBeAg（乙型肝炎e抗原）和HBeAb（乙型肝炎e抗体）：这两项指标可以反映病毒的复制活动和传染性。HBeAg阳性可能表示病毒复制活跃，传染性强；HBeAb阳性可能表示病毒复制活跃程度减弱。  4.HBcAb（乙型肝炎核心抗体）：这是体内对乙型肝炎病毒的免疫反应。IgM抗-HBc阳性可能表示急性或近期感染；IgG抗-HBc阳性可能表示慢性感染或以前的感染。  5.HBV DNA（乙型肝炎病毒DNA）：这是直接检测病毒的复制。数值较高可能表示病毒复制活跃，病情可能较重。  6.肝功能检查：如ALT（丙氨酸氨基转移酶）、AST（天冬氨酸氨基转移酶）、ALP（碱性磷酸酶）、GGT（γ-谷氨酰转肽酶）、总胆红素、直接胆红素、间接胆红素等，可以了解肝脏损伤的情况。  7.肝硬度测量（FibroScan）：这是一种无创检查，可以了解肝纤维化或肝硬化的程度。  8.肝脏彩超：可以了解肝脏的形态、大小、内部结构等情况，对肝硬化、肝癌等的早期筛查有重要意义。  以上检查项目需要根据个人的具体情况进行选择，定期进行，以及时了解乙肝的状况并进行必要的治疗。如有任何疑问，建议及时咨询医生。 | 1,1 | 1,1 |  | 1, 1 |
| 42 | Clinical Problems | Explain clinical signification of each item in five indicator test for Hepatitis B | Hepatitis B is a potentially life-threatening liver infection caused by the hepatitis B virus (HBV). There are several indicators used to diagnose and monitor the disease progression, each of which has clinical significance. Here are the five key indicators:  1.Hepatitis B Surface Antigen (HBsAg): This is the first detectable viral antigen in a Hepatitis B infection. It signifies that the person is potentially infectious. The presence of HBsAg indicates an ongoing Hepatitis B infection, either acute or chronic. If it remains detectable for more than six months, it generally signifies chronic Hepatitis B infection.  2.Hepatitis B Surface Antibody (Anti-HBs): This is an antibody produced by the immune system in response to the Hepatitis B surface antigen. It provides immunity against Hepatitis B and appears after the infection has resolved or after successful vaccination. Its presence indicates recovery and immunity from future Hepatitis B infections.  3.Hepatitis B Core Antibody (Anti-HBc): This antibody is produced in response to the core antigen of the Hepatitis B virus. The presence of anti-HBc indicates previous or ongoing infection with Hepatitis B. It can be detected in both acute and chronic infections. It remains in the system indefinitely, hence it can also be a marker of past infection.  4.Hepatitis B e Antigen (HBeAg): This is a viral protein secreted by HBV-infected cells. Its presence in the blood indicates high viral replication and high infectivity. It is often used as a marker of ability to spread the virus to other people (high infectivity).  5.Hepatitis B e Antibody (Anti-HBe): This antibody is produced by the immune system temporarily during acute HBV infection or persistently during or after a burst of HBV replication. Anti-HBe is usually associated with reduced viral replication and lower infectivity. However, some mutant strains of HBV do not produce HBeAg, and in these cases, the presence of anti-HBe does not necessarily indicate lower infectivity.  Each of these indicators plays a crucial role in diagnosing, understanding the stage of the disease, and guiding the treatment of Hepatitis B. | Sure, Hepatitis B is a viral infection that attacks the liver and can cause both acute and chronic diseases. A five-indicator test for Hepatitis B typically includes the following items:  1.HBsAg (Hepatitis B Surface Antigen): This is the first marker that appears in the blood following infection with the Hepatitis B virus. It’s the most commonly used indicator of active infection. A positive HBsAg test indicates that the person is infectious and can spread the virus to others.  2.Anti-HBs (Antibodies to Hepatitis B Surface Antigen): This is an antibody produced by the immune system in response to the presence of HBsAg. A positive Anti-HBs test usually indicates recovery and immunity from Hepatitis B virus infection. It can also indicate successful vaccination.  3.HBeAg (Hepatitis B e Antigen): This is a viral protein that’s present in the blood when the virus is actively replicating. A positive HBeAg test indicates that the virus is multiplying and the infected person has high levels of HBV. This person is highly contagious.  4.Anti-HBe (Antibodies to Hepatitis B e Antigen): These antibodies develop after the HBeAg disappears, indicating the virus is no longer replicating actively, but the person can still be infectious.  5.Anti-HBc (Antibodies to Hepatitis B core Antigen): This antibody is produced shortly after the appearance of HBsAg and persists for life. Anti-HBc is an indicator of past or ongoing infection.  It’s important to note that these tests are often used together to provide a more complete picture of a person’s Hepatitis B infection status. The results of these tests can help guide decisions about treatment and follow-up care. | 1,1 | 1,1 |  | 1, 1 |
| 42 | Clinical Problems | 解释乙肝五项检测中每个指标的临床意义 | 乙肝五项检测是对乙型肝炎病毒感染和复制情况进行评估的一种常规检测。这五项包括：  1.乙型肝炎表面抗原（HBsAg）  2.乙型肝炎表面抗体（HBsAb）  3.乙型肝炎e抗原（HBeAg）  4.乙型肝炎e抗体（HBeAb）  5.乙型肝炎核心抗体（HBcAb）  以下是每个指标的临床意义：  1.乙型肝炎表面抗原（HBsAg）：HBsAg 是乙型肝炎病毒（HBV）的重要组成部分，其存在表明体内存在 HBV，可能为慢性或急性感染。如果 HBsAg 长时间（通常超过6个月）保持阳性，可能表示慢性 HBV 感染。  2.乙型肝炎表面抗体（HBsAb）：HBsAb 是体内对 HBV 的免疫反应产生的抗体。其存在通常表明已经成功对 HBV 建立了免疫，可能是由于疫苗接种或自然感染后的恢复。如果 HBsAb 阳性，一般意味着体内没有 HBV。  3.乙型肝炎e抗原（HBeAg）：HBeAg 是 HBV 活跃复制的标志。HBeAg 阳性通常意味着高度传染性，并可能与肝病进展快有关。  4.乙型肝炎e抗体（HBeAb）：HBeAb 是体内对 HBeAg 的反应产生的抗体。其出现通常表明病毒复制活动降低，但并不一定表示病毒已被清除。  5.乙型肝炎核心抗体（HBcAb）：HBcAb 是体内对 HBV 核心抗原的反应产生的抗体。HBcAb 的存在表示曾经或现在有 HBV 感染。总的 HBcAb 可以分为 IgM 和 IgG 两种类型，其中 IgM HBcAb 阳性通常表示近期感染或急性感染，而 IgG HBcAb 阳性则可能表示过去的感染或慢性感染。  乙肝五项检测的结果需要综合分析，以便准确评估患者的感染状态和病毒活动水平。 | 乙肝五项检测是诊断乙型肝炎的重要手段，其中包含以下五个指标：  1.乙肝表面抗原 (HBsAg): 这是诊断乙肝感染最早、最直接的标志，如果这个检测结果呈阳性，可能意味着正在发生乙型肝炎感染。如果持续阳性超过半年，可能表示为慢性乙型肝炎或健康携带者。  2.乙肝表面抗体 (HBsAb): 这个抗体通常在疫苗接种或者乙型肝炎病毒感染后出现。如果这个检测结果呈阳性，可能表示已经产生了免疫，可以防止乙型肝炎病毒再次感染。  3.乙肝e抗原 (HBeAg): 这个抗原的存在表示乙型肝炎病毒正在积极复制，也就是说，病毒的传染性很强。如果这个检测结果呈阳性，可能意味着疾病正在活跃期，需要积极治疗。  4.乙肝e抗体 (HBeAb): 这个抗体的出现表示乙型肝炎病毒复制活动减少，疾病可能正在进入恢复期或者静止期。如果这个检测结果呈阳性，可能意味着疾病状态正在改善。  5.乙肝核心抗体 (HBcAb): 这个抗体的存在表示曾经有过乙型肝炎病毒感染，无论是现在还是过去。如果这个检测结果呈阳性，可能表示有过乙型肝炎病毒感染的历史。  以上五项指标的阳性或阴性，以及它们的组合，可以帮助医生判断乙型肝炎的感染状态、病程阶段以及疾病预后，从而制定个体化的治疗方案。 | 1,1 | 1,1 |  | 1, 1 |
| 43 | Clinical Problems | Explain clinical signification of HBV-DNA quantification | Hepatitis B virus (HBV) is a major global health problem. It can cause acute and chronic infection, leading to significant morbidity and mortality due to liver disease, including cirrhosis and hepatocellular carcinoma.  HBV-DNA quantification, also known as viral load testing, is a crucial tool in the management of patients with chronic hepatitis B. It measures the amount of HBV-DNA in a patient’s blood, which reflects the activity of the virus within the body.  Clinical Significance of HBV-DNA Quantification  1.Disease Progression and Activity: The level of HBV-DNA in the blood is directly related to the risk of disease progression. High levels of HBV-DNA are associated with an increased risk of liver cirrhosis and hepatocellular carcinoma. It’s also an indicator of the activity of the virus in the liver, and can be used to assess the degree of liver inflammation and damage.  2.Treatment Decision: HBV-DNA quantification is crucial in making decisions about when to start antiviral therapy. According to guidelines, antiviral treatment is recommended for patients with high HBV-DNA levels (>20,000 IU/mL in HBeAg positive patients and >2,000 IU/mL in HBeAg negative patients) and evidence of active liver disease.  3.Monitoring Treatment Response: HBV-DNA levels are monitored during treatment to assess the patient’s response to antiviral therapy. A decrease in HBV-DNA levels usually indicates a good response to treatment. The ultimate goal of treatment is to achieve undetectable HBV-DNA levels, which is associated with improved long-term outcomes.  4.Detecting Drug Resistance: During antiviral therapy, if the HBV-DNA level increases again after an initial decrease, it may suggest the development of drug resistance. In this case, a change in treatment may be required.  5.Assessing Risk of Transmission: HBV-DNA levels also correlate with the infectivity of the patient. Patients with high HBV-DNA levels are more likely to transmit the virus to others.  Therefore, HBV-DNA quantification is a vital tool in the management of chronic hepatitis B, helping clinicians to decide when to start treatment, monitor the response to therapy, detect drug resistance, and assess the risk of disease progression and transmission. | Hepatitis B virus (HBV) DNA quantification is a laboratory test that measures the amount of HBV DNA in a person’s blood. It is a crucial tool in the diagnosis and management of patients with chronic Hepatitis B infection.  The clinical significance of HBV-DNA quantification includes:  1.Diagnosis: Detecting HBV DNA in the blood confirms the presence of active HBV infection. It is especially useful in the “window period” of infection when hepatitis B surface antigen (HBsAg) may not yet be detectable.  2.Disease progression: High levels of HBV DNA are generally associated with more active disease. They correlate with higher rates of liver damage, progression to cirrhosis, and hepatocellular carcinoma (liver cancer).  3.Monitoring treatment: HBV DNA levels are used to monitor the response to antiviral therapy. A decrease in HBV DNA levels indicates a positive response to treatment, while a lack of decrease or an increase may suggest treatment failure or the development of drug resistance.  4.Infectiousness: Patients with high HBV DNA levels are more likely to transmit the virus to others.  The World Health Organization (WHO) has defined the following levels of HBV DNA in relation to treatment decisions:  (1)Low viral load: Less than 2000 IU/mL  (2)Intermediate viral load: Between 2000 and 20000 IU/mL  (3)High viral load: More than 20000 IU/mL  Patients with high viral loads are usually considered for treatment, especially if they have evidence of liver disease. However, the decision to start treatment depends on various factors, including the patient’s age, overall health, and liver function tests.  It’s important to note that HBV DNA quantification should be used in conjunction with other clinical and laboratory parameters to make a comprehensive assessment of a patient’s condition. It’s also necessary to repeat the test at regular intervals to monitor the disease progression and response to treatment. | 1,1 | 1,2 | 1,2 | 1,2 |
| 43 | Clinical Problems | 解释HBV-DNA定量的临床意义，如何理解结果 | 乙型肝炎病毒（HBV）是引起肝炎的一种重要病原体，可以导致慢性肝炎、肝硬化、肝癌等严重疾病。HBV-DNA定量检测是对乙型肝炎病毒复制活动的直接反映，其结果可以反映病毒在体内的数量和活性。  HBV-DNA定量的临床意义：  1.**病毒复制状态的判断：**HBV-DNA定量可以直接反映HBV在体内的复制状态，是评价病毒复制活动的重要指标。HBV-DNA的高低与病毒的复制活动程度密切相关。  2.**抗病毒治疗的指导：**对于乙型肝炎的治疗，HBV-DNA定量是判断抗病毒治疗效果的重要指标。治疗过程中，如果HBV-DNA水平下降，说明病毒复制受到了抑制，表明治疗有效；如果HBV-DNA水平持续升高或不下降，可能说明治疗无效或产生了耐药。  3.**疾病进展和预后的评估：**研究发现，HBV-DNA的水平与乙型肝炎的疾病进展和预后有关。HBV-DNA水平高的患者，疾病进展可能更快，预后可能更差。  如何理解HBV-DNA定量结果：  1.HBV-DNA定量的结果通常以每毫升血液中的病毒拷贝数或国际单位数（IU/mL）表示。结果的高低反映了病毒的复制活动程度。  2.HBV-DNA阴性（或者说HBV-DNA低于检测下限）：说明体内没有或者很少有病毒复制活动，可能是由于抗病毒治疗有效，或者患者的免疫系统成功控制了病毒。  3.HBV-DNA阳性，但低于治疗阈值（通常是2000 IU/mL或者2万拷贝/mL，但具体数值可能根据不同指南和情况有所不同）：说明有一定的病毒复制活动，但可能无需立即进行抗病毒治疗，需要结合其他临床情况综合判断。  4.HBV-DNA阳性，且高于治疗阈值：说明病毒复制活动较高，可能需要进行抗病毒治疗。  总的来说，HBV-DNA定量是评价乙型肝炎病毒复制活动、指导治疗、评估疾病进展和预后的重要检查。但HBV-DNA定量结果并不能单独用来判断疾病的严重程度或治疗方案，需要结合其他临床信息和检查结果，由医生进行综合判断。 | HBV-DNA定量是一种用于测定体内乙型肝炎病毒（Hepatitis B Virus, HBV）复制水平的检测方法。其结果可以用来评估病毒活动程度，指导治疗方案的选择，以及监测治疗效果和病毒抗药性。  HBV-DNA定量的临床意义  1.病毒复制水平的判断：HBV-DNA定量可以反映病毒在体内的复制情况，其浓度与病毒复制活性密切相关。HBV-DNA水平较高通常表示病毒复制活跃。  2.疾病进展和预后评估：HBV-DNA的水平与肝炎的活动性、肝硬化和肝癌的发生风险有关。持续的高水平HBV-DNA复制可能导致肝炎的进展和肝细胞癌的发展。  3.指导治疗方案的选择：根据HBV-DNA的水平，可以判断是否需要抗病毒治疗。在治疗过程中，HBV-DNA水平的变化可以反映药物的疗效和病毒对药物的抵抗情况。  4.治疗效果和病毒抗药性的监测：通过定期检测HBV-DNA，可以监测治疗效果和病毒的抗药性。如果HBV-DNA水平持续升高或者无法降低，可能提示病毒对药物产生了抗性。  如何理解HBV-DNA定量结果  HBV-DNA定量结果通常以每毫升血液中的病毒拷贝数或国际单位数（IU/mL）表示。不同实验室的检测方法和单位可能不同，因此理解结果时需要参考具体实验室的参考值。以下是一种常见的参考范围：  1.未检出：HBV-DNA水平低于检测下限，可能表示病毒复制非常低或者不存在，或者已经达到治疗目标。  2.低水平复制（小于2,000 IU/mL或10^4拷贝/mL）：可能表示病毒复制活动较低，但并不能完全排除肝病的进展。  3.中度复制（2,000-20,000 IU/mL或10^4-10^5拷贝/mL）：可能表示病毒复制活动较高，需要定期检查肝功能和病毒学指标。  4.高水平复制（大于20,000 IU/mL或10^5拷贝/mL）：通常表示病毒复制活跃，可能存在肝炎活动，需要评估治疗方案。  请注意，HBV-DNA定量结果只是评估HBV感染和治疗效果的一部分，需要结合其他临床和实验室参数（如肝功能、肝硬度、HBeAg状态等）进行综合评估。具体的诊断和治疗决策应由医生进行。 | 2,2 | 1,2 | 2,2 | 2,2 |
| 44 | Clinical Problems | Give clinical treatment advice for women of childbearing age infected with hepatitis B | As an AI developed by OpenAI, I’m capable of providing some general guidance based on available data and research. However, it’s crucial to remember that any health-related advice should always be confirmed and supervised by a healthcare professional.  Hepatitis B is a viral infection that attacks the liver. It can cause both acute and chronic disease. Here are some general considerations for the clinical treatment of women of childbearing age who are infected with Hepatitis B:  1.Antiviral Therapy: Antiviral therapy may be recommended for women with active hepatitis B virus (HBV) replication, evidence of liver inflammation, and/or liver damage. The decision to initiate therapy should be made in consultation with a healthcare provider experienced in managing hepatitis B. First-line oral antiviral agents include tenofovir disoproxil fumarate (TDF), tenofovir alafenamide (TAF), and entecavir (ETV). These drugs have been shown to be safe and effective in suppressing HBV replication.  2.Pregnancy Considerations: Women of childbearing age who have HBV need to consider the risk of vertical transmission (mother to child during childbirth). Antiviral therapy may be recommended in the third trimester of pregnancy to reduce the risk of perinatal transmission, especially for women with high viral load. Again, TDF and TAF are considered safe in pregnancy.  3.Breastfeeding: Women on antiviral therapy may wish to breastfeed. TDF and TAF are considered safe during breastfeeding.  4.Contraception: Women with HBV who do not wish to become pregnant should be counseled on contraception. Barrier methods, hormonal contraception, and intrauterine devices are all safe in women with HBV.  5.Regular Monitoring: Regular monitoring is important to assess the status of liver disease, the effectiveness of treatment, and to screen for liver cancer (which HBV patients are at increased risk for). This usually includes blood tests, and may include imaging of the liver and/or liver biopsy.  6.Vaccination of Close Contacts: All household and sexual contacts should be vaccinated against HBV if they are not already immune.  7.Healthy Lifestyle: It’s beneficial to maintain a healthy lifestyle. This includes avoiding alcohol, maintaining a balanced diet, and regular exercise.  Remember, it’s crucial to consult with a healthcare provider for personalized medical advice. This information is intended to be a general guide and may not cover all individual healthcare needs. | I’m an AI developed by OpenAI and I can provide some general advice based on available information. However, please consult a healthcare professional for medical advice as this information is subject to change and individual circumstances can significantly affect treatment options.  Hepatitis B is a viral infection that attacks the liver. In women of childbearing age, the treatment approach should consider both the health of the woman and the risk of mother-to-child transmission.  1.Monitoring  Regular monitoring of hepatitis B virus (HBV) DNA levels, alanine aminotransferase (ALT) levels, and screening for hepatocellular carcinoma (HCC) are important in managing chronic HBV infection.  2.Antiviral Therapy  Antiviral therapy may be considered in women of childbearing age with chronic hepatitis B if they have high HBV DNA levels (> 20,000 IU/mL) and/or elevated ALT levels. The choice of antiviral therapy should consider safety in pregnancy, as unplanned pregnancies may occur.  3.First-line antiviral agents recommended by the World Health Organization (WHO) include entecavir (ETV) and tenofovir disoproxil fumarate (TDF) or tenofovir alafenamide (TAF). These are potent inhibitors of HBV replication and have a high barrier to resistance. They are considered safe in pregnancy.  4.Pregnancy and Breastfeeding  Pregnant women with high HBV DNA levels (> 200,000 IU/mL) in the third trimester (from week 28) should be offered antiviral prophylaxis with TDF or TAF to prevent mother-to-child transmission of HBV, in addition to the administration of hepatitis B vaccine and hepatitis B immunoglobulin (HBIG) to the newborn within 24 hours of birth.  Breastfeeding is not contraindicated in women taking ETV, TDF, or TAF.  5.Contraception  Women of childbearing age who are not planning to become pregnant should be counseled about the use of effective contraception. Contraception should be continued for at least 6 months after stopping antiviral therapy due to the risk of flare of hepatitis B.  6.Vaccination  All household and sexual contacts of women with chronic hepatitis B should be vaccinated.  Remember that this is general advice and individual treatment plans may vary. Always consult with a healthcare provider for personalized advice.  Sources:  World Health Organization. Guidelines for the prevention, care and treatment of persons with chronic hepatitis B infection. Geneva: World Health Organization; 2015.  Terrault NA, Lok ASF, McMahon BJ, et al. Update on prevention, diagnosis, and treatment of chronic hepatitis B: AASLD 2018 hepatitis B guidance. Hepatology. 2018;67(4):1560-1599. | 1,1 | 1, 1 |  | 1, 1 |
| 44 | Clinical Problems | 如果育龄女性感染乙肝，有何临床建议？ | 乙型肝炎是一种由乙型肝炎病毒（HBV）引起的疾病，可以导致肝炎、肝硬化甚至肝癌。如果育龄女性感染乙肝，以下是一些临床建议：  1.及时就医：一旦被诊断出感染乙肝，应立即就医，接受专业医生的诊疗。  2.药物治疗：根据医生的建议，可能需要接受抗病毒治疗，如使用核苷（酸）类抗病毒药物。  3.健康生活方式：保持健康的生活方式有助于提高身体对疾病的抵抗力。这包括均衡饮食、适量运动、避免饮酒和吸烟。  4.避免传播病毒：乙肝病毒可以通过血液、性行为、母婴传播等途径传播。因此，应避免与他人共享牙刷、剃须刀、针头等可能有血液的物品。在性行为中应使用安全套。已经怀孕或打算怀孕的女性应告知医生自己的乙肝状况，以采取适当的预防措施防止病毒传给婴儿。  5.预防接种：尽管乙肝疫苗无法治疗已经感染的乙肝，但可以防止病毒传播给未感染的人。因此，与乙肝患者密切接触的人（包括家庭成员和性伴侣）应接种乙肝疫苗。  6.定期检查：定期进行肝功能检查和病毒载量检查，以监测病情的进展和药物治疗的效果。  7.妊娠期间的管理：如果你正在怀孕或计划怀孕，应与医生密切沟通，制定合适的治疗和预防方案。例如，可能需要在孕期或分娩前开始抗病毒治疗，以降低母婴传播的风险。新生儿出生后应立即接种乙肝免疫球蛋白和乙肝疫苗。  以上都是一些通用的建议，具体的治疗方案应根据个人的病情和医生的建议确定。 | 如果育龄女性感染乙肝，以下是一些临床建议：  1.定期检查：定期进行乙肝相关的检查，如乙肝病毒载量、肝功能、肝硬度等。  2.药物治疗：如果病情需要，医生可能会建议使用抗病毒药物治疗。目前主要的抗乙肝病毒药物包括干扰素和核苷（酸）类似物。  3.孕期管理：如果在孕期，应该在医生的指导下对乙肝进行管理，如定期检查、药物治疗等。特别是在孕晚期，如果乙肝病毒载量较高，可能需要使用抗病毒药物以减少母婴传播的风险。  4.分娩后管理：新生儿出生后应立即接种乙肝疫苗和乙肝免疫球蛋白（HBIG），以进一步减少母婴传播的风险。  5.健康生活方式：保持健康的生活方式对于乙肝患者来说非常重要，包括合理饮食、适当运动、戒烟戒酒等。  6.心理支持：乙肝患者可能会面临一些心理压力，包括对疾病的恐惧、对未来的不确定等。心理咨询或支持可能会有所帮助。  以上建议仅供参考，具体的治疗方案应由医生根据患者的具体情况制定。 | 1,1 | 1,2 | 1,1 | 1, 1 |
| 45 | Clinical Problems | Give advice for a patient who's HBsAb is positive | HBsAb, also known as Hepatitis B surface antibody, is a part of your body’s immune response to a past or recent Hepatitis B infection. When HBsAb is positive, it generally means one of two things:  You have been successfully vaccinated against Hepatitis B, and your body has developed immunity to the virus.  You have recovered from a past Hepatitis B infection, and your body has developed immunity as a result.  Here are some pieces of advice for you:  1.Understand your results: It’s important to understand that a positive HBsAb test is usually a good sign. It indicates that your body has developed immunity to the Hepatitis B virus, either through vaccination or by successfully fighting off an infection.  2.Consult your doctor: Always consult your healthcare provider to interpret your results and guide you about the next steps. They can provide advice tailored to your specific situation.  3.Continue regular check-ups: Regular check-ups are essential to monitor your overall health status. Even if you’re immune to Hepatitis B, you should still continue with routine health screenings.  4.Healthy lifestyle: Maintain a healthy lifestyle. This includes balanced nutrition, regular exercise, sufficient sleep, and avoiding harmful habits such as smoking and excessive alcohol consumption.  5.Preventive measures: Even though you’re immune to Hepatitis B, it’s still important to take preventive measures to avoid other infectious diseases. This includes safe sex practices, avoiding sharing needles, and getting vaccinated against other diseases as recommended by your healthcare provider.  Remember, this advice is general in nature, and individual health circumstances can vary. Always consult with your healthcare provider for advice that’s specific to your situation. | HBsAb, also known as Hepatitis B surface antibody, is a type of antibody produced by your immune system in response to the hepatitis B surface antigen (HBsAg). If your HBsAb test comes back positive, it generally means one of two things:  You’ve been successfully vaccinated against Hepatitis B, and your immune system has produced antibodies in response to the vaccine.  You’ve had a past Hepatitis B infection, and your immune system has successfully fought it off, leaving you with immunity.  Here are some pieces of advice:  1.Understand Your Results: A positive HBsAb result typically indicates immunity, either due to successful vaccination or recovery from a past infection. However, the interpretation of hepatitis B serology can be complex and should be made in the context of clinical and other laboratory findings.  2.Follow-Up: Regular follow-ups with your healthcare provider are important to monitor your health status, especially if you have been exposed to the virus or have other risk factors for liver disease.  3.Vaccination: If your positive result is due to vaccination, this is a good thing. It means that the vaccine has worked and you’re protected against the Hepatitis B virus. Ensure you’re up-to-date with your vaccines, and discuss any future required doses with your doctor.  4.Prevent Spread: If your positive result is due to a past infection, remember Hepatitis B is contagious. Even if you’ve cleared the infection, you should always take steps to prevent spreading it to others. This includes not sharing needles, toothbrushes, razors, or other personal items that might have your blood on them, and using barrier methods during sexual activity.  5.Healthy Lifestyle: Maintaining a healthy lifestyle is important for everyone, but especially for those who have had a Hepatitis B infection. Avoid alcohol and drugs, maintain a balanced diet, exercise regularly, and get enough sleep.  6.Discuss with Your Doctor: It’s always important to discuss your results with your healthcare provider. They can provide you with personalized advice based on your overall health, lifestyle, and medical history.  Remember, this advice is general in nature. Always consult with a healthcare professional for advice tailored to your specific circumstances. | 1,1 | 1,1 |  | 1, 1 |
| 45 | Clinical Problems | 乙型肝炎病毒表面抗体阳性怎么办？ | 乙型肝炎病毒表面抗体（HBsAb）阳性通常表示两种可能的情况：  1.疫苗接种后产生的免疫反应：如果你曾经接种过乙型肝炎疫苗，你的身体会产生抗体来对抗可能的病毒感染。这种情况下，HBsAb阳性是正常且健康的表现，表示你对乙型肝炎病毒有了免疫保护。  2.自然感染后的自我康复：如果你曾经被乙型肝炎病毒感染过，然后你的身体成功地清除了病毒，你的身体也会产生HBsAb。这也是一种健康的反应，表示你已经从乙型肝炎病毒感染中恢复，并对未来的感染有了免疫保护。  总的来说，如果你的HBsAb检测结果是阳性，那么你应该是对乙型肝炎病毒有了免疫。然而，你仍然应该继续进行定期的肝功能检查，并遵循医生的建议，以确保你的肝脏健康。  请注意，以上信息仅供参考，并不能替代专业医疗建议。如果你有任何疑问或担忧，你应该联系你的医疗保健提供者。 | 乙型肝炎病毒表面抗体阳性（HBsAb阳性）通常表示两种可能的情况：  1.疫苗接种后产生的免疫反应：如果你接种过乙型肝炎疫苗，身体会产生抗体来对抗乙型肝炎病毒。这种情况下，抗体阳性是正常的，表示疫苗接种成功，你已经对乙型肝炎病毒有了免疫保护。  2.自然感染后的康复：如果你曾经感染过乙型肝炎病毒并且成功康复，身体也会产生抗体。这种情况下，抗体阳性表示你已经成功抵抗了乙型肝炎病毒，且在未来有一定的抵抗力。  无论哪种情况，乙型肝炎病毒表面抗体阳性通常是一个好消息，表示你的身体已经能够抵抗乙型肝炎病毒。不过，对于任何健康问题，都应该及时咨询医生或医疗专业人士，确保得到准确的诊断和治疗建议。  请注意，以上信息仅供参考，如果有任何疑问或不适，请立即寻求医疗专业人士的帮助。 | 1,1 | 1,1 |  | 1, 1 |
| 46 | Clinical Problems | Give advice for a patient who's HBcAb is positive | If you test positive for Hepatitis B core antibody (HBcAb), it suggests that you have been infected with the Hepatitis B virus at some point in your life. Here are some general recommendations to follow:  1.Follow-Up Testing: HBcAb positive status alone is not enough to determine the current state of your Hepatitis B infection. It would be best if you had further testing, including Hepatitis B surface antigen (HBsAg), Hepatitis B surface antibody (HBsAb), and Hepatitis B e antigen (HBeAg) tests. These will help determine whether the infection is new, old, chronic, or resolved.  2.Regular Monitoring: If you have an ongoing Hepatitis B infection, regular monitoring is crucial. This may involve blood tests and liver scans to track the progress of the disease and to prevent severe complications like liver cirrhosis or liver cancer.  3.Vaccination: If you are HBcAb positive and HBsAb negative, you might still be susceptible to future Hepatitis B infections. Discuss with your doctor about the Hepatitis B vaccine.  4.Healthy Lifestyle: Maintain a healthy lifestyle to support your immune system. This includes balanced nutrition, regular exercise, avoiding alcohol and drugs, and not smoking.  5.Prevent Transmission: Hepatitis B is highly contagious. It’s important to avoid sharing personal items such as toothbrushes or razors and to use barrier methods during sexual activity to prevent transmission.  6.Treatment: If you have a chronic Hepatitis B infection, you might need antiviral medication. Your healthcare provider can provide the best advice based on your condition.  7.Support: Living with Hepatitis B can be challenging. Seek support from friends, family, or support groups. Mental health is as important as physical health.  Remember, this advice is general in nature, and the specific recommendations may vary depending on the individual’s health status and circumstances. Always consult with your healthcare provider for personalized advice. | If you have tested positive for Hepatitis B core antibody (HBcAb), it means you have been infected with the Hepatitis B virus at some point in your life. This may be a current infection or a past infection. Here are some pieces of advice:  1.Follow-up Testing: Further tests are needed to confirm if the infection is current or past. This includes Hepatitis B surface antigen (HBsAg), Hepatitis B surface antibody (HBsAb), and Hepatitis B e antigen (HBeAg) tests. You should discuss these with your healthcare provider.  2.Monitor Your Health: Regular monitoring of your liver function is essential. Your doctor may recommend blood tests and ultrasound scans to check the health of your liver.  3.Healthy Lifestyle: Adopt a healthy lifestyle. This includes a balanced diet, regular exercise, limiting alcohol intake, and avoiding smoking. These actions can help to protect your liver.  4.Avoid Spreading the Virus: Hepatitis B is highly infectious. It can be transmitted through blood, semen, vaginal fluids, and from an infected mother to her newborn. You should avoid sharing needles, razors, toothbrushes, and ensure you use protection during sexual intercourse.  5.Vaccination: While the Hepatitis B vaccine won’t help you if you’re already infected, it’s important to ensure your close contacts (like family members, sexual partners) are vaccinated to prevent them from getting the virus.  6.Treatment: If you have a current infection, antiviral medications may be recommended to slow down the virus and prevent liver damage.  7.Support: Reach out to support groups or counseling services if you’re feeling stressed or anxious about your diagnosis. This can help you cope with any emotional stress related to your condition.  Remember, it’s crucial to regularly consult with your healthcare provider, follow their advice, and inform them of any changes in your health. | 1,1 | 1,1 |  | 1, 1 |
| 46 | Clinical Problems | 乙型肝炎核心抗体阳性怎么办？ | 乙型肝炎核心抗体阳性意味着您曾经接触过乙型肝炎病毒。但是，这并不一定意味着您当前有活跃的乙型肝炎病毒感染。乙型肝炎核心抗体是对乙型肝炎病毒感染的一种免疫反应，可能在病毒感染后的很长一段时间内保持阳性。  如果您的乙型肝炎核心抗体测试结果为阳性，这是您需要做的：  1.进一步的检测：您需要进行进一步的测试，以确定您是否有活跃的乙型肝炎病毒感染。这可能包括乙型肝炎表面抗原（HBsAg）测试、乙型肝炎e抗原（HBeAg）测试和/或乙型肝炎病毒DNA定量检测。  2.咨询医生：根据您的检测结果，您应该咨询医生以获取适当的医疗建议。如果您有活跃的乙型肝炎病毒感染，您可能需要接受抗病毒治疗。  3.预防措施：如果您的乙型肝炎核心抗体阳性，您应该避免与他人共享针头、牙刷或剃须刀，以减少传播病毒的风险。您还应该告诉您的性伴侣您的状况，因为乙型肝炎可以通过性接触传播。  4.定期检查：即使您没有活跃的乙型肝炎病毒感染，您仍然需要定期进行肝脏健康检查，因为有乙型肝炎病毒感染历史的人有更高的肝病风险。  请记住，这只是一般性的建议。您应该咨询您的医生以获取针对您具体情况的医疗建议。 | 乙型肝炎核心抗体阳性（HBcAb阳性）可能表示您曾经接触过乙型肝炎病毒（HBV）。但这并不一定意味着您目前有乙型肝炎。您可能需要进行更多的检查以确定您的健康状况。  这里是一些您可能需要采取的步骤：  1.寻求专业医疗咨询：您应该尽快向医生或专业医疗机构寻求咨询。他们可以为您提供更多关于乙型肝炎核心抗体阳性的信息，并告诉您接下来需要做什么。  2.进行进一步的检查：医生可能会建议您做更多的血液检查，以更准确地了解您是否有乙型肝炎。这些检查可能包括乙型肝炎表面抗原（HBsAg）、乙型肝炎e抗原（HBeAg）、乙型肝炎e抗体（HBeAb）以及乙型肝炎病毒DNA（HBV DNA）等。  3.遵循医嘱：如果您被诊断为乙型肝炎，您需要按照医生的建议进行治疗。乙型肝炎可以通过药物控制，但不能完全治愈。治疗的目标是减少病毒复制，从而减少肝炎引起的肝损伤。  4.保护他人：乙型肝炎是通过血液、性接触或从母亲到婴儿的垂直传播来传播的。如果您被诊断为乙型肝炎，您应该采取措施防止将病毒传播给他人。  5.保持健康的生活方式：避免饮酒，保持健康的饮食习惯，进行适当的运动，都可以帮助保护您的肝脏。  请记住，这些都只是一般性的建议，您应该寻求医生的专业意见。 | 2,1 | 1,1 | 1,1 | 1, 1 |
| 47 | Clinical Problems | Give advice for a patient who's HBeAb is positive | If your Hepatitis B e Antibody (HBeAb or anti-HBe) test result is positive, it generally indicates that the virus is not actively replicating in your liver and the disease is less likely to progress rapidly. Here are a few important points to consider:  1.Monitoring: Regular check-ups with your healthcare provider are crucial. These visits allow for the monitoring of liver function and potential changes in your Hepatitis B virus (HBV) status.  2.Understanding the Test: Remember, a positive HBeAb test does not necessarily mean you are free from the virus or cannot transmit it to others. You might still be a carrier of the virus.  3.Preventing Transmission: Hepatitis B can be transmitted through blood, semen, or other body fluids, so it’s essential to avoid activities that could spread the virus to others. This includes unprotected sex, sharing needles, razors, toothbrushes, or any item that could have traces of blood.  4.Healthy Lifestyle: Maintain a healthy lifestyle to support your liver. This includes a balanced diet, regular exercise, and avoiding alcohol and tobacco.  5.Medication: Some patients with chronic Hepatitis B might need antiviral medication, even if they are HBeAb positive. The need for treatment should be determined by your healthcare provider, based on your HBV DNA levels, liver function tests, and liver damage assessment.  6.Vaccination: Encourage close contacts and family members to get vaccinated against Hepatitis B to prevent transmission.  7.Regular Screening: Regular screening for liver cancer is recommended for individuals with chronic Hepatitis B, as they have an increased risk of developing this type of cancer.  Remember, this advice is general in nature. Each patient’s situation is unique, and your healthcare provider is the best source of advice for your specific circumstances. Always consult with them regarding your condition and any changes in your health. | HBeAb, also known as Hepatitis B e-antibody, is a serological marker in the blood that is part of the testing for Hepatitis B virus (HBV). It typically indicates that the virus is less active and the disease is less likely to be severe.  However, a positive HBeAb test alone doesn’t provide a complete picture of your HBV infection status. It needs to be interpreted in conjunction with other tests such as Hepatitis B surface antigen (HBsAg), Hepatitis B e-antigen (HBeAg), and Hepatitis B core antibody (HBcAb), as well as liver function tests.  Here are some general pieces of advice if you have a positive HBeAb result:  1.Follow-up Testing: Continue with regular monitoring and follow-up tests as recommended by your healthcare provider. These may include tests for liver function and imaging studies to check for liver damage.  2.Lifestyle Changes: Adopt a healthy lifestyle. This includes eating a balanced diet, getting regular exercise, avoiding alcohol and illicit drugs, and not smoking. These steps can help to protect your liver from further damage.  3.Avoid Spreading the Virus: Hepatitis B can be transmitted through blood, semen, or other body fluids. So, it’s important to avoid sharing needles, razors, toothbrushes, or having unprotected sex.  4.Medication: Depending on the overall assessment of your HBV status, your doctor may recommend antiviral medications to help slow the progression of the disease and reduce the risk of liver damage.  5.Vaccination: While there’s no vaccine for you if you’re already infected, your close contacts (like family members and sexual partners) should get vaccinated to prevent getting the infection from you.  6.Regular Check-ups: Regular check-ups with your healthcare provider are essential to monitor your health condition and adjust your treatment plan as necessary.  Remember, every patient’s condition is unique. The above advice is general in nature and may not apply to everyone. Please consult with your healthcare provider for advice tailored to your specific circumstances. | 1,1 | 1,1 |  | 1, 1 |
| 47 | Clinical Problems | 乙型肝炎e抗体阳性怎么办？ | 乙型肝炎e抗体（HBeAb）阳性通常表示乙型肝炎病毒（HBV）的活动度较低，病毒复制的能力较弱。这是一个相对较好的情况，但仍需要定期监测和医疗专业人员的指导。以下是一些可能的建议：  1.定期检查：即使HBeAb阳性，您仍需要定期进行血液检查，监测肝功能和病毒载量。这将有助于医生评估病情的变化并及时调整治疗方案。  2,保持健康的生活方式：合理的饮食和规律的运动可以帮助保持肝脏健康。避免饮酒和吸烟，因为这些都可能损害肝脏。  3.避免传播病毒：乙型肝炎是一种可以通过血液和体液传播的病毒。避免与他人共用牙刷、剃须刀等可能导致病毒传播的物品。此外，使用安全的性行为也可以防止病毒传播。  4.接种疫苗：对于您的亲密接触者和家人，建议他们接种乙型肝炎疫苗，以防止病毒传播。  5.药物治疗：虽然HBeAb阳性通常意味着病毒活动度较低，但在某些情况下，医生可能仍会推荐药物治疗。请遵照医生的指导使用药物。  请注意，以上的建议并不能替代医生的专业意见。如果您对自己的病情有任何疑虑或问题，应及时向医疗专业人员咨询。 | 乙型肝炎e抗体阳性（HBeAb阳性）是乙型肝炎病毒感染的一个重要指标，它通常表明病毒的复制活动较低，也可能是病毒感染进入了慢性阶段。然而，这不是一个完全无害的状况，仍然需要定期监测和评估。以下是你应该采取的步骤：  1.寻求医疗咨询：如果你被检测出乙型肝炎e抗体阳性，首先应该寻求医生的建议。医生会根据你的病史、症状和其他相关检测结果，制定出适合你的诊疗方案。  2.定期检查：你需要定期进行肝功能检查和病毒载量检测，以监测病情的变化。这可以帮助你及时发现并处理可能出现的并发症。  3.保持健康的生活方式：这包括保持良好的饮食习惯、避免酗酒、避免接触有害化学物质、保持适当的运动等。这些都可以帮助保护你的肝脏健康。  4.接种疫苗：如果你的家人或者密切接触者没有接种乙型肝炎疫苗，他们应该接种，以防止病毒的传播。  5.遵守医嘱：如果医生建议你服用抗病毒药物，你应该按照医嘱服用，以控制病毒的复制。  以上都是对乙型肝炎e抗体阳性的一般建议，具体的诊疗方案还需要根据你的具体情况和医生的建议来确定。 | 1,1 | 2,1 | 1,1 | 1, 1 |
| 48 | Clinical Problems | How to understand HBV-DNA quetification results | Hepatitis B virus (HBV) DNA quantification is a lab test that measures the amount of HBV DNA in a person’s blood. This test is often used to monitor the progression of chronic hepatitis B infection and to assess the effectiveness of antiviral treatment. It provides a numerical value, usually in international units per milliliter (IU/mL) or copies per milliliter (copies/mL).  The interpretation of HBV DNA quantification results can be complex, as it depends on various factors such as the phase of the disease, patient’s immune status, and whether or not the patient is undergoing antiviral treatment. However, here’s a general guideline:  1.Undetectable HBV DNA: This means that the level of HBV DNA in the blood is too low to be detected by the test. This could indicate successful antiviral therapy or an inactive phase of the disease.  2.Low HBV DNA level: Usually less than 2,000 IU/mL (or 10,000 copies/mL), this may indicate an inactive carrier state, especially if the liver enzymes are normal and the hepatitis B surface antigen (HBsAg) is negative.  3.Moderate HBV DNA level: This typically ranges between 2,000 to 20,000 IU/mL (or 10,000 to 100,000 copies/mL). Patients with these levels may be in the ‘immune control’ phase of chronic hepatitis B, or they may have a low level of viral replication.  4.High HBV DNA level: Usually greater than 20,000 IU/mL (or 100,000 copies/mL), this may indicate active viral replication. Patients with high HBV DNA levels may be in the ‘immune clearance’ or ‘immune escape’ phase of chronic hepatitis B, and they may be at a higher risk of liver damage.  Remember that these are just general guidelines. The interpretation of HBV DNA quantification results should be done by a healthcare professional who takes into account the overall clinical picture, including the patient’s history, physical examination findings, and other laboratory results. | Hepatitis B virus (HBV) DNA quantification is a laboratory test that measures the amount of HBV DNA in a person’s blood. This test is often used to monitor the progress of chronic hepatitis B infection, assess the efficacy of antiviral therapy, and predict the risk of disease progression. The results are typically reported in international units per milliliter (IU/mL) or copies per milliliter (copies/mL).  Understanding your HBV-DNA quantification results involves a few key points:  1.HBV-DNA Level: This is the amount of HBV DNA in your blood. A higher level generally indicates a higher viral load, meaning the virus is more active and you’re more likely to transmit the virus to others. However, it’s important to remember that a higher viral load doesn’t necessarily correlate with the severity of liver disease.  2.Unit of Measurement: The results may be presented in either IU/mL or copies/mL. The conversion between these two units is not straightforward because it depends on the specific assay used. However, it’s commonly accepted that 1 IU/mL is approximately equivalent to 5-6 copies/mL.  3.Detection Limit: Each test has a lower limit of detection, which is the smallest amount of virus that the test can accurately measure. If your viral load is below this limit, it will be reported as “undetectable.” This doesn’t necessarily mean there’s no virus in your blood, but rather that the amount is too small to be measured.  4.Logarithmic Scale: HBV DNA levels are often reported on a logarithmic scale because the amount of virus in the blood can vary greatly among individuals. On a logarithmic scale, an increase of 1 log is equivalent to a 10-fold increase in the amount of virus.  Here are some general interpretations:  1.Undetectable or very low HBV DNA level (<2000 IU/mL): This usually indicates inactive disease or effective response to antiviral therapy.  2.Moderate HBV DNA level (2000-20,000 IU/mL): This may indicate an active infection, and further monitoring or treatment may be needed.  3.High HBV DNA level (>20,000 IU/mL): This typically indicates active viral replication and a higher risk of liver damage. Antiviral treatment is usually recommended.  Remember, these are just general guidelines. The interpretation of your HBV-DNA quantification results should be made in conjunction with other tests and clinical assessments by your healthcare provider. | 1,1 | 1,1 |  | 1, 1 |
| 48 | Clinical Problems | HBV-DNA定量结果怎么看？ | 乙型肝炎病毒 (HBV) 的 DNA 定量检测是评估病毒复制活动、指导抗病毒治疗和监测治疗效果的重要手段。HBV-DNA 定量结果的解读需要结合临床表现、其他实验室检查结果和治疗情况等因素。但是，从一般的角度来看，HBV-DNA 水平可以反映病毒的复制活动，其数值越高，说明病毒复制越活跃。  HBV-DNA 的测量结果通常以每毫升血液中的拷贝数或者国际单位数 (IU) 表示。以下是一种常见的解读方法：  1.未检出或低于检测下限：这表示病毒在血液中的数量非常低或者无法检测。如果病人正在接受抗病毒治疗，这可能是一个积极的迹象，表明治疗效果良好。  2.低水平阳性：这表示病毒在血液中的数量相对较低，但是仍然可以检测到。这可能是病毒复制活动较低或者抗病毒治疗效果较好的迹象。  3.中等水平阳性：这表示病毒在血液中的数量适中。这可能是病毒复制活动较高或者抗病毒治疗效果不理想的迹象。  4.高水平阳性：这表示病毒在血液中的数量很高。这通常是病毒复制活动很高的迹象，可能需要开始或者调整抗病毒治疗。  这只是一个大概的解读方法，具体的解读应当根据病人的具体情况和医生的建议进行。如果你对你的 HBV-DNA 定量结果有疑问，应当向你的医生或者其他医疗专业人员咨询。 | 乙型肝炎病毒 (HBV) DNA 检测是一种用于评估乙型肝炎病毒在体内复制活动的检测。这种检测有助于医生决定是否开始治疗以及评估治疗的有效性。  以下是如何解读 HBV DNA 定量结果：  1.未检出：HBV DNA 未检出通常意味着病毒复制活动较低或者无活动，或者您可能未被感染乙型肝炎病毒。然而，即使结果为未检出，也可能存在一定量的病毒，只是低于实验室能够检测的下限。  2.HBV DNA 定量值：这是一个数字结果，通常以每毫升血液中的病毒拷贝数表示。数值越高，表示体内病毒复制活动越强烈。  以下是一个参考范围（请注意，这些数值可能因实验室和检测方法的不同而有所不同）：  (1)高病毒负荷：大于10^7 拷贝/mL 或 10^5 IU/mL  (2)中等病毒负荷：10^5 - 10^7 拷贝/mL 或 10^3 - 10^5 IU/mL  (3)低病毒负荷：小于 10^5 拷贝/mL 或 10^3 IU/mL  请注意，结果的解读应在医生的指导下进行，因为需要综合考虑您的其他相关检测结果和病情。 | 1,1 | 1,2 | 1,1 | 1, 1 |
| 49 | Clinical Problems | Give clinical treatment advice for ones who are exposed to hepatitis B virus | I’m an AI developed by OpenAI and while I can provide general advice based on available medical literature, I must emphasize that the following information should not replace professional medical advice. If you believe you have been exposed to the hepatitis B virus, please consult with a healthcare professional immediately. Here’s some general information about the management and treatment of potential exposure to the hepatitis B virus (HBV):  1.Post-exposure prophylaxis  Post-exposure prophylaxis (PEP) is a preventive medical treatment that begins immediately after exposure to a pathogen, such as the hepatitis B virus. For hepatitis B, PEP usually consists of a combination of hepatitis B immunoglobulin (HBIG) and the hepatitis B vaccine.  The treatment should ideally be initiated within 24 hours, but it can still be effective if started within 2 weeks of exposure.  2.Hepatitis B Immunoglobulin (HBIG)  HBIG is a type of immunoglobulin (antibody) that can provide short-term protection against hepatitis B. It is often given as a single dose as soon as possible after exposure to the virus.  3.Hepatitis B Vaccine  The hepatitis B vaccine stimulates the body’s immune system to protect against the virus. It is typically given in a series of 3-4 injections over a six-month period.  4.Monitoring  Even if PEP is initiated, it is crucial to monitor for signs and symptoms of hepatitis B, which can include jaundice (yellowing of the skin and eyes), fatigue, abdominal pain, loss of appetite, intermittent nausea and vomiting.  5.Follow-up testing  Follow-up testing is needed to ensure that the PEP has been effective and that the person has not developed hepatitis B. This testing typically involves blood tests 3-6 months after the potential exposure.  6.Other Considerations  Prevention is always better than treatment. It is highly recommended that individuals who are at a higher risk of exposure to hepatitis B, such as healthcare workers, individuals with multiple sexual partners, intravenous drug users, and people traveling to regions with high rates of hepatitis B, receive the hepatitis B vaccination as a preventive measure.  Please consult with a healthcare provider for personalized advice and treatment. | I’m an AI developed by OpenAI and while I can provide general advice based on available information, please note that this advice is not a substitute for professional medical advice. Always consult with a healthcare professional for accurate information.  Hepatitis B is a viral infection that attacks the liver and can cause both acute and chronic disease. If you believe you have been exposed to the hepatitis B virus (HBV), it is important to seek medical attention immediately. Here are general steps that may be advised:  1.Post-Exposure Prophylaxis (PEP): If you seek medical attention soon after exposure (preferably within 24 hours and not later than one week), your healthcare provider may administer a dose of hepatitis B immune globulin (HBIG) and initiate the hepatitis B vaccine series. This is known as post-exposure prophylaxis (PEP).  2.Hepatitis B Vaccination: If you are not already vaccinated against hepatitis B, your healthcare provider will likely recommend starting the hepatitis B vaccine series. The vaccine can protect you from developing hepatitis B in the future.  3.Testing: Your healthcare provider will likely order blood tests to determine if you have been infected with the virus.  4.Monitoring: If you test positive for HBV, you will be monitored for signs of liver disease. This may involve regular blood tests and possibly a liver biopsy.  5.Antiviral Treatment: If you develop chronic hepatitis B, you may need treatment with antiviral medications. These medications can help slow the progression of the disease and reduce the risk of complications.  6.Healthy Lifestyle Choices: It’s important to take care of your liver if you have hepatitis B. This includes avoiding alcohol and maintaining a healthy diet.  Remember, hepatitis B can be prevented with vaccination. The vaccine is safe and effective and is usually given as 3-4 shots over a six-month period.  For any medical concerns, always consult with a healthcare professional. They can provide you with the most accurate information and treatment options based on your specific circumstances. | 1,1 | 1,1 |  | 1, 1 |
| 49 | Clinical Problems | 乙肝病毒暴露的临床处理意见 | 乙肝病毒（HBV）暴露后的临床处理是一种关键的预防措施，可以降低乙肝感染的风险。具体的处理步骤可能因个体差异和暴露程度的不同而有所不同。下面是一些常见的处理步骤：  1.评估暴露的严重程度和风险：评估患者的疫苗接种历史，以及他们是否已经接触过HBV。这可能涉及到血液测试来检查抗体水平。  2.乙肝免疫球蛋白（HBIG）的注射：如果患者在暴露后的24-48小时内接受HBIG的注射，那么他们感染乙肝的风险可以降低75%。HBIG可以提供短期保护，但不会提供长期免疫力。  3.乙肝疫苗：如果患者尚未接种疫苗，或者他们的抗体水平不足，那么应该尽快接种乙肝疫苗。乙肝疫苗通常需要三次注射，每次注射间隔一个月。  4.跟踪和监控：在接受治疗后，患者需要定期进行血液检查，以确保他们没有感染HBV。如果他们出现任何症状，例如黄疸或疲劳，那么他们应该立即寻求医疗帮助。  5.预防性抗病毒治疗：对于高风险的患者，例如那些免疫系统受损的患者，可能需要预防性的抗病毒治疗。  请注意，这只是一般的处理建议，具体的处理方案需要根据患者的具体情况和医生的建议来确定。 | 乙型肝炎病毒（HBV）暴露后的临床处理主要包括以下几个步骤：  1.评估暴露风险： 首先，需要评估接触者的疫苗接种历史和HBV感染状况。如果接触者已经接种乙肝疫苗并且疫苗反应良好，那么他们通常会有抵抗HBV感染的能力。  后暴露预防： 对于未接种或接种情况不明的接触者，应立即接种乙肝疫苗。对于高风险暴露（例如，暴露者是已知的HBV携带者），同时给予乙肝免疫球蛋白（HBIG）可以提供短期保护。  2.监测和跟踪： 在接种乙肝疫苗和/或接种HBIG后，应定期进行血清学检测，以监测HBV感染的可能性。  3.预防性抗病毒治疗： 对于高风险的接触者（例如，接触者是HBV携带者的性伴侣或者共享针头的药物使用者），可能需要预防性抗病毒治疗，以减少HBV感染的风险。  请注意，这些都是一般性的建议，具体的处理方案需要根据接触者的具体情况和医生的建议来确定。 | 1,2 | 2,2 | 1,2 | 1,2 |
| 50 | Clinical Problems | Can Hepatitis B be cured？ | Hepatitis B is a viral infection that attacks the liver and can cause both acute and chronic diseases. The virus is transmitted through contact with the blood or other body fluids of an infected person.  As for the treatment, it depends on the type of Hepatitis B infection:  1.Acute Hepatitis B: Acute Hepatitis B is a short-term illness that occurs within the first 6 months after someone is exposed to the Hepatitis B virus. Acute infection can — but does not always — lead to chronic infection. Treatment for acute Hepatitis B usually involves supportive care, meaning that the body is allowed to heal on its own while symptoms are managed.  2.Chronic Hepatitis B: Chronic Hepatitis B is a long-term illness that occurs when the Hepatitis B virus remains in a person’s body. While there is currently no definitive cure for chronic Hepatitis B, the virus can be controlled with treatment, potentially stopping or slowing down the progression of liver disease. Antiviral medications can help fight the virus and slow its ability to damage your liver.  3.In addition to antiviral medications, monitoring of liver health is also a critical part of managing chronic Hepatitis B. This is because people with chronic Hepatitis B have an increased risk of developing liver disease, cirrhosis, or liver cancer. Regular screening can help detect these conditions early and improve outcomes.  4.Vaccination is the most effective measure to prevent Hepatitis B infection. The Hepatitis B vaccine is safe and effective and is usually given as 3-4 shots over a six-month period.  Please note that while treatments can help manage Hepatitis B, they should be prescribed and monitored by a healthcare professional. This information is intended for general knowledge and should not be used as a substitute for medical advice. Always consult with a healthcare provider for any health-related concerns. | Hepatitis B is a viral infection that attacks the liver and can cause both acute and chronic diseases. The virus is transmitted through contact with the blood or other body fluids of an infected person.  While there isn’t a complete cure for chronic Hepatitis B as of 2024, it can be managed with effective treatment to reduce the risk of liver disease and prevent its spread to others. Acute Hepatitis B, on the other hand, often resolves on its own and may not require treatment.  The treatment for chronic Hepatitis B includes antiviral medications that can slow down the virus’s ability to damage the liver. These drugs include lamivudine (Epivir), adefovir (Hepsera), entecavir (Baraclude), telbivudine (Tyzeka), tenofovir (Viread), and others. Interferon alfa-2b (Intron A) is also used as a treatment.  Regular monitoring to assess the progression of the disease or the effectiveness of the treatment is also necessary.  It’s important to note that vaccination can prevent Hepatitis B. The Hepatitis B vaccine is routinely given to infants as part of their immunization schedule, and it’s also recommended for certain high-risk adults.  Always consult a healthcare professional for accurate information. | 1,1 | 1,1 |  | 1, 1 |
| 50 | Clinical Problems | 乙肝可以治愈吗 | 乙型肝炎（乙肝）是由乙型肝炎病毒（HBV）引起的一种疾病。乙型肝炎的临床表现多样，有的患者可能出现黄疸、疲劳、恶心、腹痛等症状，有的患者可能无任何症状。  乙型肝炎是否可以治愈，取决于病毒感染的类型（急性或慢性）和患者的身体反应。  1.急性乙型肝炎：大多数成年人在感染后能够自身清除病毒，恢复健康。这种情况下，他们的身体产生了对乙型肝炎病毒的免疫反应，可以防止再次感染。因此，可以说他们“治愈”了乙型肝炎。  2.慢性乙型肝炎：如果乙型肝炎病毒持续存在于患者体内超过6个月，那么就被诊断为慢性乙型肝炎。这种情况下，病毒无法被完全清除，但是可以通过药物治疗控制病毒的复制，减少肝炎的活动，防止肝硬化和肝癌的发生。尽管不能完全“治愈”，但是通过持续的治疗和管理，患者可以维持正常的生活质量和预期寿命。  总的来说，乙型肝炎是否可以治愈取决于具体情况，但无论怎样，早期发现和适当的管理都是关键。 | 乙型肝炎（HBV）是一种由乙型肝炎病毒引起的肝脏疾病。乙肝的治疗目标主要是控制病毒复制，减少肝炎活动，防止肝硬化和肝癌的发生。  对于大多数成年人来说，他们的免疫系统能够在感染后的六个月内成功清除乙型肝炎病毒，这被称为急性乙型肝炎。对于这些人来说，他们通常不需要特别的治疗。  然而，一部分人的身体无法成功清除病毒，他们会发展成慢性乙型肝炎。这种情况下，虽然乙肝目前还无法完全治愈，但有许多抗病毒药物可以有效控制病毒，延缓疾病进程，提高生活质量，降低并发症的风险。这些药物包括干扰素和核苷（酸）类似物等。  总的来说，乙型肝炎是否可以治愈取决于病毒感染的类型（急性或慢性），以及个体的身体反应。每个人的病情都是不同的，因此治疗方案也需要根据个人情况进行定制。如果您或您认识的人被诊断为乙型肝炎，应与医生讨论最佳的治疗选项。 | 1,1 | 1,1 |  | 1, 1 |
| 51 | Clinical Problems | If I have been infected with the hepatitis B virus in the past, can I get it again | If you’ve been infected with the hepatitis B virus (HBV) in the past and your body successfully fought off the infection, your immune system would have developed antibodies against HBV. This would make you immune to future infections with the same virus, similar to how vaccination works.  However, not all people fully clear the virus from their bodies. In some cases, HBV can become a chronic infection, which means the virus remains in the person’s body for a long time, potentially their whole life. These individuals are at risk for severe liver disease.  If you had a chronic HBV infection and were treated, it’s technically possible to get reinfected, particularly if you engage in behaviors that increase your risk of exposure to the virus. But this is generally rare, especially if you follow recommended precautions to prevent exposure to HBV.  If you’re unsure about your immunity status, you should discuss this with your healthcare provider. They can perform tests to check for the presence of HBV antibodies in your blood, which would indicate whether you’re immune. They can also advise you on any necessary precautions or treatments.  Please note that while this answer is based on current scientific understanding, research is ongoing, and our understanding of viral infections and immunity continues to evolve. Always consult with a healthcare professional for the most accurate information. | Once you have been infected with the Hepatitis B virus (HBV) and your body has cleared the infection, you cannot get infected with the virus again. This is because your immune system builds a protective response, producing specific antibodies against the virus.  When you’re exposed to HBV, your immune system fights off the infection over several months. If successful, you become immune to the virus, meaning you cannot get infected again. This immunity is often lifelong.  However, not everyone is able to clear the infection. Some people, especially those infected as infants or young children, develop a chronic (long-lasting) HBV infection. These individuals continue to carry the virus and can transmit it to others.  Also, keep in mind that immunity to HBV does not protect you against other types of hepatitis viruses, such as Hepatitis A, C, D, or E. Therefore, it’s important to take necessary precautions and consider vaccinations where available. | 1,1 | 1,2 | 1,1 | 1, 1 |
| 51 | Clinical Problems | 我以前感染过乙肝，还会再感染吗 | 乙型肝炎是由乙型肝炎病毒（HBV）引起的，这是一种能够攻击并损伤肝脏的病毒。如果你已经感染过乙型肝炎并且康复了，那么你的身体就已经产生了对HBV的免疫。这种免疫可以防止你再次感染同一种病毒。  然而，这并不意味着你完全不会再感染肝炎。尽管你对乙型肝炎病毒有了免疫，但你仍然可能感染其他类型的肝炎病毒，如甲型、丙型、丁型和戊型肝炎病毒。  为了保持健康，你应该避免与已知的肝炎病毒携带者接触，避免使用未经消毒的针头，避免无保护的性行为，避免接触他人的血液和体液，以及确保你的食物和水是安全的。 | 乙肝（乙型肝炎）是一种由乙型肝炎病毒（HBV）引起的疾病。乙肝的感染状态取决于个体的免疫反应，感染乙肝后的大多数成年人能够清除病毒并获得免疫，这意味着他们将不再对乙型肝炎病毒（HBV）敏感。  然而，有些人在感染后无法清除病毒，成为慢性乙肝患者。这些人体内的病毒活跃度可能会有所变化，有时可能无症状，有时可能出现病毒复制活跃的症状。这并不是再次感染，而是原有感染的活跃度变化。  如果你以前感染过乙肝并已经清除了病毒，通常你不会再次感染。如果你是慢性乙肝患者，你可能会经历病毒活动度的变化，但这不是再次感染。但无论如何，你应该定期进行医学检查，以监测你的肝脏健康状况。 | 1,1 | 1,1 |  | 1, 1 |
| 52 | Clinical Problems | Can I donate blood/organs if I have hepatitis B? | If you have hepatitis B, there are certain restrictions on your ability to donate blood or organs.  Blood Donation:  The American Red Cross, along with many other blood donation organizations around the world, does not accept blood donations from individuals who have tested positive for hepatitis B. This is because hepatitis B is a blood-borne virus that can be transmitted through transfusions.  Organ Donation:  When it comes to organ donation, the situation is a bit more complex. In general, it’s possible for a person with hepatitis B to donate organs, but there are several factors that need to be considered:  1.The health of the organ: If the hepatitis B virus has caused significant damage to the organ, it may not be suitable for transplantation.  2.The recipient’s health: If the recipient is already infected with hepatitis B, receiving an organ from a donor with the same virus might not pose an additional risk. However, this is something that the medical team would need to assess on a case-by-case basis.  3.The urgency of the transplant: In some cases, the immediate need for a transplant might outweigh the risks associated with receiving an organ from a donor with hepatitis B.  It’s important to note that these decisions are made by medical professionals on a case-by-case basis. If you have hepatitis B and are considering organ donation, you should discuss this with your healthcare provider. They can provide you with the most accurate information based on your specific circumstances. | Hepatitis B is a viral infection that attacks the liver and can cause both acute and chronic disease. It is a major global health problem and can lead to chronic liver disease and puts people at high risk of death from cirrhosis of the liver and liver cancer.  Blood Donation  People with Hepatitis B are generally not allowed to donate blood. This is because the virus can be transmitted through blood transfusion. Blood banks typically screen potential donors for several types of hepatitis to prevent the spread of these diseases. According to the American Red Cross, individuals who have ever tested positive for hepatitis B are not eligible to donate blood.  Organ Donation  When it comes to organ donation, the situation is a bit more complex. While people with Hepatitis B can’t donate organs in most cases, there are exceptions. For instance, in some cases, a liver from a donor who was infected with Hepatitis B can be transplanted into a recipient who also has the disease.  However, the use of organs from donors with Hepatitis B varies depending on several factors, including the severity of the potential recipient’s condition, the availability of organs, and the specific policies of the transplant center.  It’s important to note that organ transplantation is a complex medical procedure and decisions about donor eligibility should be made by healthcare professionals.  Always consult with your healthcare provider or local blood/organ donation center for the most accurate information. | 1,1 | 1,1 |  | 1, 1 |
| 52 | Clinical Problems | 得了乙肝可以献血/捐献器官吗？ | 乙肝病毒（HBV）是一种严重的肝病病毒，可以通过血液、体液或母婴传播。因此，如果你有乙肝，你不能献血或捐献器官。  献血：献血中心通常会对所有潜在的献血者进行健康检查，包括对各种疾病（如乙肝、艾滋病等）的筛查。如果筛查结果为阳性，那么这个人将不被允许献血。这是为了保护接受者的健康，防止通过输血传播疾病。  器官捐献：虽然有乙肝的人可能不能捐献大部分器官，但在某些情况下，他们可能仍然能够捐献。例如，有乙肝的人可能可以捐献肝脏给另一个已经有乙肝的人。这种情况下，接受者已经有乙肝，所以不会因为移植而增加感染的风险。然而，这需要在医生的指导下进行，并且必须在患者之间进行详细的匹配和评估。  总的来说，如果你有乙肝，你应该向医生咨询你是否能够捐献器官。然而，你不能献血，因为这可能会将病毒传播给接受者。 | 乙型肝炎病毒（HBV）是一种严重的肝脏疾病，可以通过血液、性接触或从母亲传给新生儿等方式传播。因此，如果您患有乙型肝炎，那么您可能不适合献血或捐献器官。  1.献血：乙型肝炎患者通常不能献血。献血中心在接受捐赠前会对所有潜在的献血者进行一系列的健康检查，包括对各种疾病的筛查，其中就包括乙型肝炎。这是为了确保接受血液的患者不会被传染上任何疾病。  2.捐献器官：乙型肝炎患者能否捐献器官则取决于多种因素。一些情况下，如果接受器官的患者已经有乙型肝炎，或者其他治疗方式无效，医生可能会考虑使用乙型肝炎患者的器官。但这需要在医生的严格评估和接受器官的患者明确知情同意的前提下进行。并且，即使在这种情况下，乙型肝炎患者也可能不被允许捐献某些器官，如心脏和肺部。  总的来说，如果您患有乙型肝炎并且正在考虑献血或捐献器官，最好是咨询医生或相关的健康专业人士。他们可以为您提供最准确的信息，并根据您的具体情况给出建议。 | 1,1 | 1,1 |  | 1, 1 |
| 53 | Clinical Problems | Is it harmful to have an extra dose of hepatitis B vaccine or to repeat the entire hepatitis B vaccine series? | According to the Centers for Disease Control and Prevention (CDC) and other health organizations, receiving an extra dose of the hepatitis B vaccine or repeating the entire vaccine series is not harmful.  Hepatitis B vaccine is produced from non-infectious parts of the virus and cannot cause hepatitis B infection. It is a safe vaccine that has a low rate of side effects. In the case of any potential side effects, they are usually mild and temporary.  However, there is usually no need to receive extra doses or to repeat the vaccine series if you have already completed it. The hepatitis B vaccine provides long-term protection. According to the CDC, for most people, the hepatitis B vaccine provides protection for at least 20 years and possibly for a lifetime.  If you’re unsure about your vaccination status or if you need an extra dose, it’s best to consult with your healthcare provider. They can check your immunity to hepatitis B with a blood test and provide you with the best advice based on your personal health history.  Remember that while the hepatitis B vaccine is very safe, like any medicine, it could potentially cause a serious problem, such as a severe allergic reaction. However, the risk of a vaccine causing serious harm, or death, is extremely small.  It’s important to note that information about vaccines and their safety can change over time as new research is conducted, so it’s always a good idea to check the latest guidelines from trusted health organizations or consult with your healthcare provider.  Please note: This information is intended to provide general knowledge and is not a substitute for professional medical advice or treatment for specific medical conditions. Always seek the advice of your physician or other qualified healthcare provider with any questions you may have regarding a medical condition. | Hepatitis B vaccine is given to provide protection against the hepatitis B virus, which can cause serious illness, including liver damage, cirrhosis, and even liver cancer. The vaccine is typically given in a series of three or four shots over six months.  While it is important to follow the recommended vaccine schedule for optimal protection, receiving an extra dose of the hepatitis B vaccine or repeating the entire series is generally not harmful. The Centers for Disease Control and Prevention (CDC) states that there is no risk for overdose or adverse effects from receiving extra doses of the hepatitis B vaccine.  However, it is not typically necessary to repeat the entire vaccine series if you have already completed it. The vaccine provides long-term protection, and booster doses are not usually recommended for most people.  As always, if you have any concerns about your vaccination status or schedule, it is best to consult with a healthcare provider. They can provide guidance based on your specific health history and needs.  Please note that while the hepatitis B vaccine is very safe and effective, like any medicine, it can have side effects. Most people do not have any side effects, and when they do occur, they are usually mild and go away on their own. Common side effects can include soreness at the injection site and a low-grade fever.  Again, always consult with a healthcare provider if you have any concerns or questions about vaccinations.  References:  1.Centers for Disease Control and Prevention. (2023). Hepatitis B Questions and Answers for the Public. Retrieved from CDC website.  2.World Health Organization. (2024). Hepatitis B. Retrieved from WHO website. | 1,1 | 1,1 |  | 1, 1 |
| 53 | Clinical Problems | 乙肝疫苗多打几针有害吗 | 乙肝疫苗通常是按照固定的程序进行接种的，一般需要接种3针，分别在出生后的0、1、6个月进行。这个疫苗接种程序是经过大量科学研究和临床实践确定的，以确保疫苗的有效性和安全性。  如果在规定的接种程序中漏打了疫苗，应尽快补打。如果不小心多打了一针，一般情况下不会产生严重的副作用。乙肝疫苗的副作用通常比较轻微，如接种部位红肿、疼痛，或者轻度发热等。  然而，过度接种乙肝疫苗并无额外的健康益处，反而可能增加体验副作用的风险。每次接种疫苗都可能引发免疫系统的反应，虽然这些反应通常是短暂的和轻微的，但如果频繁接种，可能会对身体产生不必要的压力。  总的来说，乙肝疫苗的接种应该按照推荐的程序进行。如果你对乙肝疫苗接种有疑问或担忧，应该咨询医生或专业的医疗保健提供者。 | 乙肝疫苗通常是一个三剂量的疫苗，分别在出生后的时间点接种：出生后立即接种，1个月后，以及6个月后。这是为了最有效地刺激免疫系统产生对乙肝病毒的抵抗力。  如果你意外地多打了一针乙肝疫苗，通常不会造成严重的问题。乙肝疫苗是一种非常安全的疫苗，过量接种可能会导致短暂的不适，如肌肉疼痛或发热，但这些症状通常会很快消退。  然而，这并不意味着可以随意增加疫苗的剂量。疫苗接种应该按照推荐的剂量和时间表进行，以确保其效果并减少任何潜在的副作用。如果你不确定你的疫苗接种状况，你应该咨询你的医生或医疗保健提供者。  总的来说，尽管多打一针乙肝疫苗通常不会造成严重的问题，但最好还是按照推荐的剂量和接种时间表进行接种，以确保最佳的保护效果。 | 1,1 | 1,1 |  | 1, 1 |
| 54 | Clinical Problems | What should I do if I started but did not complete the full hepatitis B vaccine series | If you started but did not complete the full Hepatitis B vaccine series, it’s important to know that you don’t need to start the series over. Here’s what you should do:  1.Schedule an appointment with your healthcare provider as soon as possible. Your doctor will be able to provide the best advice based on your specific health situation and vaccine history.  2.Continue the vaccine series where you left off. The Hepatitis B vaccine series typically consists of three doses. If you missed the second or third dose, you should get the next dose as soon as possible. You do not need to start the series over.  3.Keep track of your vaccine schedule. It’s essential to keep a record of your vaccinations to ensure you’re up-to-date. You can use a vaccination card, an app, or a digital health record system to track this information.  4.Follow-up testing. In some cases, your healthcare provider might recommend blood tests after the completion of the vaccine series to confirm that you have developed immunity against Hepatitis B.  Remember, Hepatitis B can cause serious liver disease, including liver cancer. Completing the full vaccine series is crucial in protecting your health.  Always consult with your healthcare provider for personalized advice regarding vaccinations. | If you started but did not complete the full Hepatitis B vaccine series, it’s important to complete the series for full protection.  The Hepatitis B vaccine is typically given as a series of 3 doses:  1.The first dose (at any time)  2.The second dose (at least one month after the first dose)  3.The third dose (at least two months after the second dose and at least four months after the first dose)  If you missed any dose in the series, you should get the missed dose as soon as possible. You do not need to start the series over again if you missed the second or third dose.  However, please remember that the information provided here is a general guideline. It is important to consult with your healthcare provider for the best advice tailored to your specific situation. Your healthcare provider can provide the most accurate information based on your health history and current health status.  Please also note that while the vaccine is very effective at preventing Hepatitis B, it does not provide protection against other forms of hepatitis, such as Hepatitis A or C. It’s also important to take other precautions to prevent Hepatitis B, such as using a condom during sex, not sharing needles, and avoiding direct contact with the blood or open sores of an infected person. | 1,2 | 1,2 |  | 1,2 |
| 54 | Clinical Problems | 乙肝疫苗没有全程接种完该怎么办 | 乙肝疫苗的接种通常分为三次，以确保免疫效果。然而，如果你没有完成全程接种，你可以采取以下步骤：  1.立即联系医生或接种单位：他们会为你提供适当的建议，如何继续你的疫苗接种。一般来说，只要你没有完成全程接种，就需要继续接种。  2.确定你的接种时间表：乙肝疫苗通常在出生后的第一个月，第二个月和第六个月进行接种。如果你错过了接种时间，通常可以在任何时间补充接种。  3.进行抗体水平检测：如果你已经接种了一部分疫苗，但不确定是否需要再次接种，可以进行乙肝表面抗体（HBsAb）检测。如果你的HBsAb水平大于10 mIU/mL，那么你被认为对乙肝有免疫力。  4.继续接种：如果你的抗体水平不足，你可能需要继续接种疫苗。在某些情况下，你可能需要重新开始整个疫苗接种过程。  请注意，这只是一般性的建议，具体的处理方式可能会因个体情况和地方政策的不同而有所不同。请咨询你的医生或接种单位以获取适合你的最佳建议。 | 乙肝疫苗的接种通常分为三次，分别在出生后的24小时内，第1个月和第6个月。如果你没有按照这个时间表接种完所有的疫苗，你可以按照以下的步骤进行：  1.查明疫苗接种情况：首先，你需要明确你已经接种了多少剂乙肝疫苗。这可能需要你查阅你的疫苗接种记录或者联系你的医疗提供者。  2.尽快接种剩余疫苗：如果你发现你没有接种完全程的疫苗，你应该尽快接种剩余的疫苗。乙肝疫苗的接种间隔可以调整，但每一剂疫苗的接种都是重要的。  3.咨询医生：如果你对你的疫苗接种情况有任何疑问，或者你不确定你是否需要接种更多的疫苗，你应该咨询你的医生或者其他医疗提供者。  请注意，即使你错过了原定的接种时间，你也不需要重新开始接种全程的疫苗。你只需要接种剩余的疫苗即可。乙肝疫苗的接种是为了保护你免受乙肝病毒的感染，所以尽快完成接种是非常重要的。 | 1,1 | 1,2 | 1,1 | 1, 1 |
| 55 | Clinical Problems | Is the hepatitis B vaccine recommended before international travel? | Yes, the Hepatitis B vaccine is commonly recommended for international travelers, especially if they are likely to be exposed to blood or other bodily fluids, have sexual contact with the local population, or be exposed through medical treatment.  Hepatitis B is a serious infection that affects the liver and can be spread through contact with the blood, semen, or other body fluids of an infected person. It’s more prevalent in certain parts of the world, including Asia, Africa, and Eastern Europe.  The Hepatitis B vaccine is safe and effective, providing long-term protection against the virus. It’s typically given as a series of three or four shots over a six-month period. However, even if your travel date is soon, starting the vaccine series before leaving can still provide some protection.  Before traveling internationally, it’s always a good idea to consult with a healthcare provider or a travel medicine specialist to ensure that you are up-to-date on all recommended vaccines and health precautions based on your specific travel plans. | Yes, the Hepatitis B vaccine is commonly recommended for international travelers, especially if they are likely to be exposed to blood, have sexual contact with the local population, or be exposed through medical treatment.  Hepatitis B is a liver infection caused by the Hepatitis B virus (HBV). It can be a mild illness lasting for a few weeks, or it can become a serious, long-term (chronic) condition that can lead to liver disease or liver cancer.  The Hepatitis B vaccine is safe and effective. The Centers for Disease Control and Prevention (CDC) recommends that all unvaccinated adults and children traveling to areas with an intermediate to high level of endemic HBV infection receive the Hepatitis B vaccine.  However, it’s always best to consult with a healthcare provider or a travel medicine specialist before traveling to discuss individual health conditions and the specifics of the planned trip, as these could influence the need for specific vaccines. | 1,2 | 1,2 |  | 1,2 |
| 55 | Clinical Problems | 出国旅行前需要打乙肝疫苗吗 | 是否需要接种乙肝疫苗取决于几个因素：  1.目的地：您前往的国家或地区的乙肝疫情如何。如果您前往的国家或地区乙肝感染率较高，接种乙肝疫苗是非常重要的。  2.个人状况：如果您已经接种过乙肝疫苗，并且疫苗产生了足够的抗体，那么您可能不需要再次接种。然而，如果您从未接种过，或者您的抗体水平低于防护水平，那么您可能需要接种乙肝疫苗。  3.活动类型：您在旅行期间可能进行的活动类型也会影响您是否需要接种乙肝疫苗。例如，如果您可能接触到血液或体液，或者可能发生性接触，那么您可能需要接种乙肝疫苗。  建议在出国旅行前咨询您的医生或者当地的旅行健康诊所，以确定是否需要接种乙肝疫苗。 | 是否需要接种乙肝疫苗取决于你要去的目的地，你的健康状况，以及你是否已经接种过乙肝疫苗。乙肝疫苗主要用于预防乙型肝炎，这是一种通过血液、性接触或从母亲传给新生儿的病毒。  以下是一些你可能需要考虑接种乙肝疫苗的情况：  1.**你计划去的地方乙型肝炎的发病率高。**一些地区，特别是亚洲、太平洋群岛、非洲和东欧的某些地方，乙型肝炎的发病率较高。  2.**你可能暴露在乙型肝炎病毒中。**这可能包括通过性接触、使用注射药物、或者在医疗机构工作。  3.**你还没有接种过乙肝疫苗。**大多数国家都会在儿童时期给予乙肝疫苗，但如果你没有接种过，那么在旅行前接种可能是个好主意。  最好的建议是在你的旅行前咨询医生或者旅行医疗专家，他们可以根据你的具体情况提供最好的建议。同时，记住接种乙肝疫苗需要一段时间才能建立免疫力，所以如果你需要接种，最好提前几周或几个月计划。 | 1,1 | 1,1 |  | 1, 1 |
| 56 | Clinical Problems | What is an hepatitis B booster dose, and do I need one? | A Hepatitis B booster dose is an additional dose of the Hepatitis B vaccine that is given to individuals after they have completed the initial series of vaccinations. This booster dose is meant to “boost” or strengthen the immune response against the Hepatitis B virus.  The Hepatitis B vaccine is typically given as a series of 3-4 doses over a six-month period. The immune response to this vaccine is generally very strong and long-lasting, often providing lifelong immunity.  However, for some people, the immunity might decrease over time. In such cases, a booster dose might be recommended. These can include:  1.Healthcare workers and others at high risk of exposure: These individuals are often recommended to have their antibody levels checked periodically. If their immunity has decreased, a booster dose might be recommended.  2.People with certain medical conditions: Some conditions, such as kidney disease or HIV, can affect the immune system’s response to the vaccine. People with these conditions might need a booster dose.  3.Travelers to high-risk areas: If you’re traveling to an area where Hepatitis B is common, and it’s been many years since your last vaccine, your doctor might recommend a booster dose.  If you’re unsure whether you need a Hepatitis B booster dose, the best course of action is to consult with your healthcare provider. They can review your medical history, risk factors, and potentially test your Hepatitis B immunity level to make a recommendation. | A Hepatitis B booster dose is an additional dose of the Hepatitis B vaccine that is given after the initial series of vaccinations. The purpose of a booster dose is to “boost” or strengthen the immune response against the Hepatitis B virus, especially in individuals who might not have developed sufficient immunity after the initial vaccination series.  Hepatitis B is a viral infection that attacks the liver and can cause both acute and chronic disease. The virus is transmitted through contact with the blood or other body fluids of an infected person.  The need for a Hepatitis B booster dose depends on various factors:  1.Your antibody levels: After the initial series of Hepatitis B vaccinations, it’s recommended to check the Hepatitis B surface antibody (anti-HBs) levels. If the anti-HBs level is less than 10 mIU/mL, then a booster dose may be needed.  2.Your risk of exposure: Certain groups of people who have a higher risk of Hepatitis B infection might need a booster. This includes healthcare workers, people who frequently require blood or blood products, dialysis patients, and people with an increased risk due to sexual exposure.  3.Your age at initial vaccination: The immunity from Hepatitis B vaccine decreases over time. So, if you were vaccinated as a child and now you’re an adult at high risk of Hepatitis B infection, you might need a booster dose.  4.Your overall health status: People with weakened immune systems (for example, due to HIV infection, end-stage renal disease, or because they’re receiving certain types of immunosuppressive therapies) may require booster doses because they might not respond as well to the vaccine.  It’s important to consult with a healthcare provider to understand whether a Hepatitis B booster dose is needed in your specific case. They will consider your risk factors, your antibody levels, and your overall health status to make the best recommendation for you. | 2,1 | 1,1 | 2,1 | 1, 1 |
| 56 | Clinical Problems | 乙肝疫苗加强针是什么，我需要打吗 | 乙肝疫苗加强针，也称为乙肝疫苗补充剂或者乙肝疫苗追加剂，是在完成乙肝疫苗基础免疫后，为了进一步提高免疫效果而进行的额外接种。一般来说，乙肝疫苗加强针的接种时间是在基础免疫后的5年或者10年。  是否需要接种乙肝疫苗加强针，取决于您的具体情况。以下是一些可能需要考虑接种乙肝疫苗加强针的情况：  1.如果您的血液抗体水平降低到一定程度，可能需要接种乙肝疫苗加强针。  2.如果您属于高风险人群，例如医护人员、血液透析患者或者免疫系统较弱的人群，可能需要定期接种乙肝疫苗加强针。  3.如果您经常需要接触乙肝病毒，例如您的工作环境或生活环境中存在乙肝病毒，那么您可能需要定期接种乙肝疫苗加强针。  总的来说，是否需要接种乙肝疫苗加强针，最好是咨询医生或者专业的医疗机构，他们会根据您的具体情况来给出建议。 | 乙肝疫苗加强针通常是在初次接种乙肝疫苗的基础上，为了提高或保持免疫力，再次接种的疫苗。  乙肝疫苗一般在新生儿出生后的24小时内接种第一针，随后在1个月和6个月时接种第二针和第三针。这是标准的乙肝疫苗接种程序。然而，有些人可能需要接种加强针，比如：  1.抗体水平低于预期的人。这通常在完成初次接种后6-12个月进行抗-HBs抗体水平检测后发现。如果抗体水平低于10 mIU/ml，那么可能需要接种加强针。  2.免疫系统功能低下的人，比如HIV感染者、器官移植接受者或正在接受免疫抑制治疗的人。这些人可能需要定期检查抗-HBs抗体水平，并根据需要接种加强针。  3.暴露于高风险环境的人，比如医疗工作者或有可能接触乙肝病毒的人。这些人可能需要定期检查抗-HBs抗体水平，并根据需要接种加强针。  是否需要接种乙肝疫苗加强针，需要根据你的个人健康状况、职业风险、生活环境等因素，与医疗专业人员进行详细的讨论和评估。如果你对此有疑虑，建议尽快咨询医生。 | 1,1 | 1,1 |  | 1, 1 |
| 57 | Clinical Problems | Can hepatitis B vaccine be given during pregnancy or breast feeding? | Yes, the hepatitis B vaccine can be given during pregnancy or breastfeeding.  The Centers for Disease Control and Prevention (CDC) and the World Health Organization (WHO) both recommend the hepatitis B vaccine for pregnant women if they are at risk for hepatitis B virus infection. The vaccine is made from non-live virus, so it cannot cause hepatitis B in the mother or the baby.  Hepatitis B can be a serious illness that can lead to liver disease or liver cancer. If a pregnant woman has hepatitis B, she can pass the virus to her baby during childbirth. The vaccine can protect both the mother and the baby from this virus.  Breastfeeding is also considered safe after receiving the hepatitis B vaccine. The vaccine does not affect the safety of breast milk or the health of the baby. In fact, all infants, including those born to mothers who are hepatitis B surface antigen (HBsAg) positive, should receive the hepatitis B vaccine as part of their routine immunization schedule.  As always, it’s important to discuss any questions or concerns about vaccines with a healthcare provider. | Yes, the Hepatitis B vaccine can be safely given during pregnancy or breastfeeding.  According to the Centers for Disease Control and Prevention (CDC) and the Advisory Committee on Immunization Practices (ACIP), Hepatitis B vaccination should be administered to unvaccinated pregnant women who are at risk for infection. The vaccine has not been shown to cause adverse effects in pregnant women or their newborns.  In addition, breastfeeding is not a contraindication for the Hepatitis B vaccine. Therefore, the vaccine can be given to breastfeeding mothers who are unvaccinated and at risk for Hepatitis B infection.  However, as with any healthcare decision, it is important for pregnant or breastfeeding women to discuss the benefits and risks of vaccination with their healthcare provider.  Please note that this information is up-to-date as of June 2024. Always consult with a healthcare professional for the most current advice. | 1,1 | 1,1 |  | 1, 1 |
| 57 | Clinical Problems | 妊娠或哺乳期可以打乙肝疫苗吗 | 乙型肝炎疫苗在妊娠或哺乳期通常被认为是安全的，没有明显的风险。这是因为乙型肝炎疫苗是一种灭活疫苗，不含有活的病毒，因此不会引起疾病。  然而，尽管乙型肝炎疫苗被广泛接受并认为是安全的，每个人的情况都是独特的，所以任何疫苗接种的决定都应在医生的指导下进行。如果你正在妊娠或哺乳期，你应该与你的医生讨论接种乙型肝炎疫苗的可能的风险和好处。  总的来说，乙型肝炎疫苗可以帮助防止乙型肝炎，这是一种严重的肝病，可以导致肝硬化和肝癌。如果你处于乙型肝炎的高风险环境中，接种疫苗可能是一个好的选择。 | 是的，妊娠或哺乳期的妇女可以接种乙肝疫苗。乙肝疫苗并不会对孕妇或其胎儿产生不良影响。实际上，如果孕妇处于乙肝病毒的高风险环境中，接种乙肝疫苗是非常重要的，因为这可以防止妇女和她的新生儿感染乙肝病毒。  然而，如同接种任何疫苗一样，你应该在接种乙肝疫苗前咨询你的医生或医疗服务提供者，以确保这是对你和你的宝宝最安全的选择。 | 1,1 | 1,1 |  | 1, 1 |
| 58 | Clinical Problems | What should I do if I received the HEPLISAV-B vaccine while pregnant? | If you received the HEPLISAV-B vaccine while pregnant, the first thing you should do is consult your healthcare provider. As of my last training data in October 2021, the safety of the HEPLISAV-B vaccine for use during pregnancy had not been determined.  However, the Centers for Disease Control and Prevention (CDC) and the Advisory Committee on Immunization Practices (ACIP) had not made a specific recommendation against its use in pregnant women. They suggest that the potential benefits and risks should be considered and discussed with a healthcare provider.  It’s important to note that while the vaccine is not live, and therefore cannot cause hepatitis B in the mother or the baby, there is limited data on its safety during pregnancy.  Here are some steps to take:  1.Contact your healthcare provider: Inform them about your vaccination. They will be able to provide advice based on the most current research and guidelines.  2.Monitor your health: While adverse reactions to the HEPLISAV-B vaccine are rare, you should still monitor for any unusual symptoms and report them to your healthcare provider.  3.Follow regular prenatal care: Continue with your regular prenatal visits and care. This will allow your healthcare provider to monitor both your health and the health of your baby.  4.Stay informed: Guidelines and research can change. Stay informed about the latest information on vaccines during pregnancy.  Remember, this advice is based on information available up to October 2021. Always consult with your healthcare provider for the most recent and personalized advice. | HEPLISAV-B is a vaccine used to prevent Hepatitis B, a potentially serious liver infection caused by the Hepatitis B virus. It is typically given in two doses over a month.  Pregnancy is not a contraindication for Hepatitis B vaccination. In fact, the Centers for Disease Control and Prevention (CDC) recommends Hepatitis B vaccination for pregnant women who are at risk for the infection. This includes women who have had more than one sex partner during the previous 6 months, those with a sex partner who is a Hepatitis B carrier, and others with risk factors.  However, the specific safety of HEPLISAV-B in pregnant women has not been thoroughly studied. Therefore, it is considered a “Pregnancy Category C” medication, which means risk cannot be ruled out.  If you received the HEPLISAV-B vaccine while pregnant, you should:  1.Don’t Panic: Receiving the vaccine doesn’t necessarily mean there will be negative outcomes. In fact, getting infected with Hepatitis B during pregnancy can be more harmful, as it can lead to serious issues for both mother and baby.  2.Inform your healthcare provider: If you haven’t done so already, let your healthcare provider know that you received the vaccine. They can provide advice tailored to your specific situation.  3.Monitor for any adverse reactions: While the vaccine is generally safe, it can sometimes cause side effects. Common side effects include pain and swelling at the injection site, headache, fatigue, and fever. If you experience any unusual or severe symptoms, seek medical attention.  4.Follow up with your healthcare provider: Depending on your risk level for Hepatitis B, your healthcare provider may recommend additional testing or follow-up.  Remember, it’s always best to discuss any questions or concerns you have with your healthcare provider. They can provide the most accurate information based on your personal health history and circumstances. | 2,2 | 2,2 |  | 2,2 |
| 58 | Clinical Problems | NA |  |  |  |  |  |  |
| 59 | Clinical Problems | Where can I get the hepatitis B vaccine? | You can get the Hepatitis B vaccine at several types of locations:  1.Doctor’s Office: Your primary care physician can administer the Hepatitis B vaccine.  2.Health Departments: Many city or county health departments offer the Hepatitis B vaccine.  3.Pharmacies: Some pharmacies, such as those in large chains like CVS or Walgreens, offer the Hepatitis B vaccine.  4.Travel Clinics: If you’re planning to travel to a region where Hepatitis B is common, you can often get vaccinated at a travel clinic.  5.Community Health Centers: These centers often provide vaccinations, including the Hepatitis B vaccine.  6.Workplace Health Clinics: Some employers offer the Hepatitis B vaccine through their health clinics.  Remember to call ahead to confirm availability of the vaccine. If you’re unsure where to go, your healthcare provider should be able to give you advice.  Note: The availability of these services may vary based on your location and healthcare system. | The Hepatitis B vaccine is widely available and can be obtained from a variety of sources:  1.Primary Care Physician or Family Doctor: They can usually administer the vaccine right in their office. If they can’t, they should be able to give you a referral to a place that can.  2.Pharmacies: Many pharmacies, especially larger chains like CVS, Walgreens, and Walmart, have walk-in clinics that can administer vaccines.  3.Health Departments: Your local or state health department may offer vaccines, often at reduced cost for those who qualify.  4.Travel Clinics: If you’re planning on traveling internationally, you may be able to get the vaccine at a travel clinic. These clinics specialize in providing vaccinations and other health services for travelers.  5.Occupational Health Clinics: If you work in healthcare or another profession that puts you at risk for Hepatitis B, your employer may offer the vaccine through an occupational health clinic.  6.Community Health Clinics: Many community health clinics, especially those in larger cities, offer a wide range of vaccines.  7.Universities and Colleges: If you’re a student, your school’s health center may offer the Hepatitis B vaccine.  Before going to any of these places, it’s a good idea to call ahead and confirm that they have the vaccine available. You should also check whether your insurance covers the vaccine and if any copayments apply. If you don’t have insurance, ask about the cost and any available assistance programs. | 2,1 | 2,1 |  | 2,1 |
| 59 | Clinical Problems | 乙肝疫苗获取途径有哪些？ | 乙肝疫苗是预防乙型肝炎的重要手段，以下是获取乙肝疫苗的常见途径：  1.公共卫生部门：在许多国家和地区，公共卫生部门会提供免费或者低价的乙肝疫苗接种服务。在中国，乙肝疫苗是儿童免疫规划的一部分，新生儿出生后24小时内免费接种第一剂，后续剂次在医疗机构按照规定时间接种。  2.医院和诊所：大部分医院和诊所都可以提供乙肝疫苗接种服务，但可能需要支付一定的费用。  3.药店：在一些地区，药店也有提供乙肝疫苗，但需要注意的是，药店购买的疫苗需要在合格的医疗机构进行接种。  4.学校和工作场所：一些学校和工作场所会定期组织乙肝疫苗接种活动，这是获取疫苗的一个方便途径。  5.旅行疫苗接种中心：如果你打算去乙肝高发的地区旅行，可以考虑在旅行疫苗接种中心接种乙肝疫苗。  获取乙肝疫苗的具体途径可能因地区和国家的政策而不同，建议向当地的公共卫生部门或者医疗机构咨询。 | 乙肝疫苗（Hepatitis B vaccine）是预防乙型肝炎的最有效手段之一。以下是获取乙肝疫苗的几种途径：  1.医疗机构：大部分医院、诊所和社区卫生服务中心都可以提供乙肝疫苗。  2.预防接种服务中心：许多城市有专门的预防接种服务中心，可以提供各种疫苗接种服务。  3.药店：一些药店也提供乙肝疫苗，但需要咨询药店是否有资质提供接种服务。  4.学校和工作场所：一些学校和工作场所会组织员工和学生接种乙肝疫苗。  5.旅行接种服务：如果您计划前往乙肝高发地区旅行，可以通过旅行接种服务提前接种乙肝疫苗。  无论在哪里接种乙肝疫苗，都要确保是在有资质的医疗机构，并由专业医务人员进行。同时，接种前应咨询医生，了解自己是否适合接种，以及可能的副作用。 | 1,1 | 1,1 |  | 1, 1 |
| 60 | Clinical Problems | How serious is chronic (long-term) hepatitis B? | Chronic hepatitis B is a serious health condition that affects the liver. It is caused by the hepatitis B virus (HBV), which can lead to long-term health problems like cirrhosis (scarring of the liver), liver failure, liver cancer, and even death.  The course of chronic hepatitis B can vary greatly among individuals. Some people may be asymptomatic (without symptoms) for many years or even a lifetime, while others may develop serious liver disease. This variation depends on several factors including the age at which infection was acquired, the individual’s immune system, and whether they have other health conditions.  Here are some key points about chronic hepatitis B:  1.Cirrhosis: Chronic hepatitis B can lead to cirrhosis, a condition where the liver tissue is gradually replaced by scar tissue, impairing the liver’s ability to function. It’s estimated that 15-40% of people with chronic hepatitis B will develop cirrhosis over their lifetime.  2.Liver cancer: People with chronic hepatitis B are at an increased risk of developing hepatocellular carcinoma (HCC), a type of liver cancer. HBV is the second most common cause of cancer worldwide, after tobacco smoking.  3.Liver failure: This is a life-threatening condition that requires urgent medical attention. It can occur suddenly (acute liver failure) or over a long period of time (chronic liver failure). Chronic hepatitis B is a leading cause of liver failure.  4.Mortality: Chronic hepatitis B is a major global health problem and one of the leading causes of death worldwide. According to the World Health Organization (WHO), in 2019, hepatitis B resulted in an estimated 820,000 deaths, mostly from complications including cirrhosis and hepatocellular carcinoma.  5.Treatment: While there is no cure for chronic hepatitis B, antiviral medications can help to slow the progression of the disease, reduce the risk of complications and improve quality of life.  6.Prevention: Hepatitis B is preventable with a safe and effective vaccine, which has been available since 1982. The WHO recommends that all infants receive the hepatitis B vaccine as soon as possible after birth, ideally within 24 hours.  In conclusion, chronic hepatitis B is a serious disease that requires careful management and regular monitoring. Early diagnosis and treatment can significantly improve the long-term health outcomes for individuals with this disease. | Chronic hepatitis B is a serious health condition that requires careful management. It’s an infection of the liver caused by the hepatitis B virus (HBV) that persists for six months or longer.  Here are some of the reasons why chronic hepatitis B is serious:  1.Liver Damage: Chronic hepatitis B can lead to severe liver damage. This includes conditions such as cirrhosis (scarring of the liver) and liver cancer.  2.Liver Cancer: Hepatitis B is one of the most common causes of liver cancer, which is the second most lethal type of cancer worldwide.  3.Liver Failure: The damage caused by chronic hepatitis B can ultimately lead to liver failure, which is a life-threatening condition that requires a liver transplant.  4.Transmission: The virus can be easily transmitted to others through blood, semen, or other body fluids, making it a significant public health concern.  5.Complications: Chronic hepatitis B can lead to other complications such as ascites (fluid in the abdomen), esophageal varices (enlarged veins in the esophagus), and hepatic encephalopathy (a decline in brain function due to severe liver disease).  It’s important to note that not everyone with chronic hepatitis B will develop these complications. The progression of the disease varies greatly among individuals and is influenced by a number of factors, including the person’s age at the time of infection, overall health, and lifestyle.  Treatment can slow the progression of the disease, reduce the risk of complications, and improve a person’s quality of life. Antiviral medications are commonly used, and in some cases, a liver transplant may be necessary. Regular monitoring and screening are also important to detect any complications early.  Therefore, if you or someone you know has chronic hepatitis B, it’s important to work closely with a healthcare provider to manage the condition and to discuss the most appropriate treatment options. | 1,1 | 1,1 |  | 1, 1 |
| 60 | Clinical Problems | 慢性乙肝严重吗 | 乙型肝炎（HBV）是一种由乙型肝炎病毒引起的肝炎，可以引起急性和慢性感染。乙型肝炎的严重程度因人而异，取决于许多因素，包括病毒复制水平、肝脏的损伤程度、个体的免疫反应以及是否存在其他肝脏疾病。  慢性乙型肝炎是一种长期存在的肝炎病毒感染，可能会导致肝硬化和肝癌。慢性乙型肝炎的进展通常非常缓慢，可能需要几十年才能出现症状或并发症。  这种疾病的严重性因人而异，有些人可能一生都没有症状，而其他人可能会发展出严重的肝病。关键是定期进行医疗检查，以监控疾病的进展并采取适当的治疗措施。  值得注意的是，尽管慢性乙型肝炎无法被完全治愈，但是有有效的抗病毒药物可以控制病毒复制，降低并发症的风险。同时，保持健康的生活方式（如避免饮酒，保持健康的体重等）也可以帮助保护肝脏。  总的来说，慢性乙型肝炎是一种严重的疾病，需要定期医疗监测和适当的治疗。如果你或你认识的人被诊断为慢性乙型肝炎，务必与医生密切合作，定期检查并按医嘱用药。 | 乙型肝炎（HBV）是一种严重的肝脏疾病，可以成为慢性疾病。慢性乙型肝炎是全球公共卫生问题的重要部分，特别是在亚洲和太平洋地区。  乙型肝炎的严重性取决于多种因素，包括：  1.病毒复制的活跃程度  2.患者的年龄和健康状况  3.是否有并发症，如肝硬化或肝癌  一些人可能在感染后的数十年内没有症状，而其他人可能会发展出严重的肝脏疾病。慢性乙型肝炎可以导致肝硬化、肝衰竭、肝癌，甚至死亡。  不过，慢性乙型肝炎是可以管理的。有一些抗病毒药物可以帮助控制病毒复制，降低并发症的风险。定期的医疗检查也是关键，以便及时发现和处理可能的并发症。  总的来说，慢性乙型肝炎的严重性因人而异，但它是一种需要严肃对待的疾病。如果你或你认识的人被诊断为慢性乙型肝炎，应该定期接受医疗检查，并遵循医生的治疗建议。 | 1,1 | 1,1 |  | 1, 1 |
| 61 | Clinical Problems | Who should be tested for hepatitis B? | Testing for Hepatitis B is important to prevent the spread of the disease and initiate early treatment if necessary. The Centers for Disease Control and Prevention (CDC) recommends Hepatitis B testing for the following groups:  1.People born in regions with high rates of Hepatitis B infection: This includes regions like Asia, Africa, the Amazon Basin in South America, the Pacific Islands, Eastern Europe, and the Middle East.  2.People born to parents who have immigrated from regions with high Hepatitis B infection rates: The risk of Hepatitis B can be higher in these individuals, even if they were not born in these regions themselves.  3.People who live with someone who has Hepatitis B: Hepatitis B can spread from person to person through blood, semen, or other body fluids.  4.Healthcare workers and others who come into contact with blood: This includes people who may be exposed to blood through their work, like doctors, nurses, and first responders.  5.People with HIV: People with HIV are often recommended to get tested for other viral infections, including Hepatitis B.  6.Men who have sex with men: This group has a higher risk of Hepatitis B infection.  7.People who inject drugs: Sharing needles can spread Hepatitis B.  8.People with multiple sexual partners: Having unprotected sex with multiple partners can increase the risk of Hepatitis B.  9.People with chronic liver disease or kidney disease: These individuals are at a higher risk of complications if they get Hepatitis B.  10.Patients needing immunosuppressive therapy: As their immune system is suppressed, they are more susceptible to infections.  11.Pregnant women: All pregnant women should be tested for Hepatitis B to prevent mother-to-child transmission during birth.  It’s important to remember that anyone can get Hepatitis B, and these are just high-risk groups. If you think you might be at risk, talk to your healthcare provider about getting tested. The best way to prevent Hepatitis B is by getting vaccinated. | Hepatitis B is a serious liver infection caused by the Hepatitis B virus (HBV). It’s a major global health problem and can cause chronic infection, leading to cirrhosis of the liver, liver cancer, liver failure, and death.  The Centers for Disease Control and Prevention (CDC) recommends Hepatitis B testing for the following groups:  1.People born in regions with high rates of HBV infection: This includes Asia, Africa, the Amazon Basin in South America, the Pacific Islands, Eastern Europe, and the Middle East.  2.People whose parents were born in regions with high HBV infection rates: This is especially important for those whose mothers are HBV positive, as the virus can be transmitted from mother to child during birth.  3.People with certain medical conditions: This includes those who are HIV-positive, on hemodialysis, or who have a weakened immune system.  4.People who live with or have sex with a person who has an HBV infection: Hepatitis B can be sexually transmitted.  5.Men who have sex with men: This group has a higher risk of HBV infection.  6.People who inject drugs: Sharing needles can transmit the virus.  7.Healthcare and public safety workers at risk of exposure to blood or body fluids: This includes doctors, nurses, paramedics, and police officers.  8.Residents and staff of facilities for developmentally disabled persons: Close living conditions can facilitate the spread of the virus.  9.Inmates of correctional facilities: Similar to the above, close living conditions can facilitate the spread of the virus.  10.People with elevated alanine aminotransferase levels (a liver enzyme): This could indicate liver damage, possibly from HBV infection.  11.Pregnant women: Routine prenatal testing for HBV is recommended for all pregnant women.  12.Infants born to HBV-infected mothers: These infants should be tested and vaccinated within 12 hours of birth.  Remember, early detection of Hepatitis B can lead to better management and treatment of the condition, reducing the risk of serious liver disease. It’s important to consult with a healthcare provider for personalized advice regarding Hepatitis B testing. | 1,1 | 1,1 |  | 1, 1 |
| 61 | Clinical Problems | 哪些人应该检查乙肝 | 乙型肝炎是一种严重的肝病，由乙型肝炎病毒（HBV）引起。以下是一些应该进行乙肝检查的人群：  1.与乙型肝炎病毒感染者有密切接触的人：例如家庭成员、性伴侣或者与病毒感染者共享过针头的人。  2.来自乙型肝炎病毒高发地区的人：例如亚洲、非洲和东欧的人。  3.没有接种过乙型肝炎疫苗的新生儿：特别是如果他们的母亲是乙型肝炎病毒感染者。  4.有慢性肝病或者肝炎的人。  5.有肾病的人，包括接受透析治疗的人。  6.有HIV感染的人。  7.接受血液制品治疗的人。  8.工作环境可能接触到他人血液的人：例如医护人员、消防员、警察和紧急医疗服务人员。  9.吸毒者，尤其是注射毒品的人。  10.有多个性伴侣或者有男男性行为的男性。  以上人群应定期进行乙肝检查，如果发现自己有乙型肝炎病毒感染，应尽早接受治疗，以防止病情进一步恶化。同时，接种乙型肝炎疫苗也是预防乙型肝炎的有效方法。 | 乙肝（乙型肝炎）是一种由乙型肝炎病毒（HBV）引起的肝脏疾病。以下是一些需要检查乙肝的人群：  1.与乙肝患者有亲密接触的人：包括与乙肝患者共享住所、食物或个人卫生用品的人，以及性伴侣。  2.从未接种乙肝疫苗的人：乙肝疫苗是预防乙型肝炎的最有效方法。没有接种疫苗的人应定期进行乙肝检查。  3.注射药物使用者：注射药物使用者常常会共享针筒，这是乙型肝炎病毒传播的常见途径。  4.医疗保健工作者：医疗保健工作者有可能接触到患者的血液和体液，因此他们应定期进行乙肝检查。  5.来自乙肝高发区的人：某些地区的乙肝感染率比其他地方高，如亚洲、非洲和东欧。来自这些地区的人应进行乙肝检查。  6.有肝病或者肝病风险的人：例如，患有肝硬化或者肝癌的人，或者有家族肝病史的人。  7.血液透析患者：这些患者由于接触到的血液比较多，因此有更高的感染乙肝的风险。  请注意，这只是一份不完全的清单，实际上可能有更多需要进行乙肝检查的人群。如果你有任何疑虑，应该咨询医生。 | 1,2 | 1,2 |  | 1,2 |
| 62 | Clinical Problems | How do I know if I have hepatitis B? | Hepatitis B is a serious liver infection caused by the hepatitis B virus (HBV). Some people, especially young children, may not have any symptoms during the initial infection. Chronic hepatitis B often remains symptomless for many years until it leads to liver damage.  However, some common symptoms can indicate Hepatitis B:  1.Fatigue  2.Nausea and vomiting  3.Loss of appetite  4.Dark urine  5.Pale or light-colored stools  6.Joint pain  7.Abdominal pain  8.Jaundice (yellowing of the skin and the whites of the eyes)  Please note that having these symptoms doesn’t necessarily mean you have Hepatitis B, as these symptoms are common to many different conditions.  To confirm whether you have Hepatitis B, you need to get tested. The tests for Hepatitis B include:  1.Hepatitis B surface antigen test (HBsAg): This test checks for the presence of the Hepatitis B virus in your blood. If it’s positive, it means you’re currently infected with the virus.  2.Hepatitis B core antigen test (HBcAg): This test checks for an antigen produced by the virus. It’s used mainly in research settings, as it’s not usually present in the blood.  3.Hepatitis B surface antibody test (Anti-HBs): This test checks for antibodies that are produced in response to a Hepatitis B infection. A positive result means you’re immune to the virus, either from vaccination or from recovering from a past infection.  4.Hepatitis B core antibody test (Anti-HBc): This test checks for antibodies that your body makes in response to the core antigen. A positive result could mean either a current or past infection.  5.Hepatitis B e antigen test (HBeAg): This test checks for an antigen that indicates the virus is replicating and you’re highly infectious.  6.Hepatitis B e antibody test (Anti-HBe): This test checks for antibodies that are produced when the virus is no longer replicating, but you could still be infected.  7.Hepatitis B DNA test: This test checks for the genetic material of the virus. It indicates how much virus is present and how quickly it’s multiplying.  If you suspect you have Hepatitis B, please consult a healthcare professional immediately. They will be able to guide you on the next steps and treatment if necessary.  Please remember that this information is meant to be informative and is not a substitute for professional medical advice. | Hepatitis B is a serious infection of the liver caused by the hepatitis B virus (HBV). It can lead to chronic conditions such as liver disease, liver cancer, or even death.  The only sure way to know if you have Hepatitis B is to get tested. However, some people may experience symptoms which can include:  1.Jaundice (yellowing of the skin and eyes)  2.Dark urine  3.Extreme fatigue  4.Nausea  5.Vomiting  6.Abdominal pain, particularly on the right side beneath the lower ribs (where the liver is located)  7.Joint pain  8.Loss of appetite  9.Fever  It’s important to note that many people with hepatitis B do not have symptoms and may not know they’re infected. Therefore, if you think you have been exposed to the virus, or you are in a high-risk group (such as healthcare workers, people who inject drugs, people who have sex with an infected partner, people with multiple sexual partners, people with a sexually transmitted infection, or people who travel to countries with high rates of hepatitis B), you should get tested.  Testing for hepatitis B typically involves blood tests that look for markers of the virus in your body, such as:  1.Hepatitis B surface antigen (HBsAg): This test is used to detect the presence of the virus in your blood. A positive result indicates an active hepatitis B infection.  2.Hepatitis B surface antibody (anti-HBs): This test is used to determine if you are immune to hepatitis B, either through vaccination or past infection.  3.Hepatitis B core antibody (anti-HBc): This test is used to determine if you have been infected with hepatitis B in the past or are currently infected.  4.Hepatitis B e antigen (HBeAg): This test is used to determine if the virus is actively replicating, indicating a high level of infectiousness.  Please consult with a healthcare professional if you think you may have been exposed to the hepatitis B virus or are experiencing symptoms. They can provide you with the appropriate testing and treatment options. | 1,1 | 1,1 |  | 1, 1 |
| 62 | Clinical Problems | 如何确定自己有乙肝 | 乙肝，即乙型肝炎，是由乙型肝炎病毒（HBV）引起的一种肝脏疾病。确定自己是否患有乙肝，主要依赖于以下几个步骤：  1.自我观察症状：乙型肝炎的常见症状包括乏力、食欲不振、恶心、黄疸（皮肤和眼白变黄）、深色尿液和肝区疼痛。但值得注意的是，很多乙肝患者在早期阶段可能无任何症状。  2.血液检测：如果你有上述症状，或者你有可能暴露在乙型肝炎病毒的环境中（例如，你的性伴侣或家人患有乙肝，或者你接触过被乙肝病毒污染的血液），你应该进行血液检测。血液检测可以检测到血液中的乙型肝炎病毒以及抗体。  3.进一步的医学检查：如果血液检测结果呈阳性，医生可能会建议进行进一步的检查，例如肝脏超声波检查、肝脏活检等，以评估肝脏的健康状况和疾病的严重程度。  如果你认为自己有可能患有乙肝，应该尽快寻求医疗建议。早期发现和治疗乙肝可以帮助防止肝炎发展为肝硬化或肝癌。 | 乙型肝炎，简称乙肝，是一种由乙型肝炎病毒（Hepatitis B virus, HBV）引起的疾病。以下是确定自己是否有乙肝的步骤：  1.自我观察症状：乙肝的症状可能包括疲劳，恶心，肝区疼痛，黄疸（眼睛和皮肤变黄），尿液颜色深，便便颜色浅等。需要注意的是，有些人可能没有明显的症状。  2.进行血液检测：如果你有上述症状或你认为自己可能接触到乙型肝炎病毒，你应该进行血液检测。血液检测可以检测到体内是否有乙型肝炎病毒或体内是否有抗乙型肝炎病毒的抗体。以下是一些常见的乙肝相关的血液检测：  (1)乙肝表面抗原（HBsAg）：如果此项检测结果为阳性，可能意味着你有乙肝。  (2)乙肝表面抗体（HBsAb或anti-HBs）：如果此项检测结果为阳性，可能意味着你已经接种过乙肝疫苗或你曾经感染过乙肝并已经康复。  (3)乙肝e抗原（HBeAg）和乙肝e抗体（anti-HBe）：这两项检测可以帮助医生了解病毒在体内的活跃程度。  (4)乙肝核心抗体（HBcAb或anti-HBc）：如果此项检测结果为阳性，可能意味着你曾经感染过乙肝病毒。  (5)乙肝病毒DNA（HBV DNA）：这项检测可以帮助医生了解体内病毒的数量。  请注意，这些检测结果需要由医生进行解读。如果你有任何疑虑，你应该尽快寻求医生的帮助。  3.获取医生的诊断：基于你的症状和血液检测结果，医生会给你做出诊断。  记住，乙肝是可以预防的。乙肝疫苗是预防乙肝的最有效方法。如果你还没有接种乙肝疫苗，你应该考虑接种。 | 2,1 | 2,2 | 2,1 | 2,1 |
| 63 | Clinical Problems | What should I do after learning that I have hepatitis B? | I’m an AI developed by OpenAI and while I can provide some general guidance, please consult with a healthcare professional for advice tailored to your specific situation. If you have been diagnosed with Hepatitis B, here are some steps you might consider:  1.Consult with a Healthcare Provider: The most important step is to consult with a healthcare provider. They will be able to provide you with specific advice based on your health history and current situation.  2.Understand Your Diagnosis: Hepatitis B is a viral infection that attacks the liver and can cause both acute and chronic disease. The virus is transmitted through contact with the blood or other body fluids of an infected person.  3.Treatment Options: Depending on the type of Hepatitis B (acute or chronic), your doctor will discuss treatment options. Acute Hepatitis B usually doesn’t require treatment and most people fully recover. Chronic Hepatitis B is a long-term illness that may require ongoing treatment.  4.Monitoring: Regular monitoring is important as Hepatitis B can lead to severe liver problems, such as cirrhosis or liver cancer. Your doctor may order regular blood tests or ultrasounds to monitor your liver health.  5.Prevent Transmission: Hepatitis B is contagious. Follow guidelines to prevent spreading the virus to others. This includes not sharing personal items such as toothbrushes or razors, using protection during sexual activity, and not donating blood or organs.  6.Healthy Lifestyle: A healthy lifestyle can help your liver. This includes avoiding alcohol, maintaining a balanced diet, exercising regularly, and getting enough sleep.  7.Vaccination: If you live with others, they should get tested and consider getting vaccinated against Hepatitis B to prevent transmission.  8.Support: Reach out to support groups and resources. Living with a chronic illness can be challenging, and it can be helpful to connect with others who are going through the same experience.  Remember, each person’s situation is unique and your healthcare provider is the best source of information for your individual circumstances. | I’m an AI language model, not a doctor, but I can suggest some general steps that are typically recommended after a hepatitis B diagnosis. Remember, it’s very important to consult with a healthcare professional for personalized advice.  1.Confirm the Diagnosis: Hepatitis B is diagnosed through blood tests. If you’ve been diagnosed, it’s important to understand whether you have an acute (short-term) or chronic (long-term) infection. This will influence the treatment approach.  2.Consult with a Specialist: It’s important to consult with a healthcare professional, such as a hepatologist, gastroenterologist, or infectious disease specialist, who specializes in liver diseases. They can provide you with the most accurate information and treatment options.  3.Understand the Disease: Learn about hepatitis B, its transmission methods, and how to prevent spreading it to others. Hepatitis B is primarily spread through blood and body fluids.  4.Begin Treatment if Necessary: Not all people with hepatitis B require treatment. If you do, antiviral medications are typically used. The goal of treatment is to reduce the risk of liver disease and prevent the spread of the virus to others.  5.Regular Monitoring: Regular monitoring is necessary to manage the disease. This usually involves blood tests and liver scans to assess the health of your liver and the status of the virus.  6.Maintain a Healthy Lifestyle: Avoid alcohol and maintain a balanced diet, as alcohol can cause additional liver damage. Regular exercise is also beneficial.  7.Inform Close Contacts and Family Members: Hepatitis B can be transmitted to others. It’s important to inform those close to you about your condition so they can also get tested and vaccinated if necessary.  8.Prevent Transmission: Use barrier methods during sex, don’t share needles, and avoid sharing personal items like toothbrushes or razors that could have your blood on them.  9.Get Vaccinated for Hepatitis A: If you have hepatitis B, it’s a good idea to also get vaccinated against hepatitis A to prevent co-infection, which could lead to more severe liver disease.  Remember, this information is general advice and may not fully apply to your specific situation. Please consult with a healthcare professional for advice tailored to your circumstances. | 1,1 | 1,2 | 1,1 | 1, 1 |
| 63 | Clinical Problems | 知道自己乙肝后应该怎么办 | 首先，我要强调，我是一个人工智能助手，我不能提供医疗建议。但是，我可以告诉你，如果你发现自己患有乙型肝炎（乙肝），你应该尽快寻找医疗专业人士进行咨询。以下是一些可能的步骤，但这些并不替代专业医疗建议：  1.寻求医疗咨询：你应该尽快找到一个医生，最好是肝脏疾病专家（肝病专科医生或肝脏病学家）进行咨询。他们会对你的病情进行全面评估，并制定最适合你的治疗方案。  2.定期检查：乙肝是一种慢性疾病，需要定期进行血液检查和肝脏超声检查以监测病情的进展。  3.药物治疗：根据你的病情，医生可能会建议你使用抗病毒药物。这些药物可以帮助控制病毒复制，减少肝脏炎症和纤维化的风险。  4.生活方式改变：避免饮酒，因为酒精可以加速肝脏损伤。保持健康的饮食和运动习惯也有助于维持肝脏健康。  5.防止传播：乙肝是通过血液和体液传播的，因此你应该避免与他人共享牙刷、剃须刀、针头等。在性行为中应使用安全措施，如使用避孕套。你的亲密伴侣和家庭成员也可能需要接种乙肝疫苗。  6.心理支持：发现自己患有慢性疾病可能会引发压力和焦虑。寻求心理咨询和支持可能有助于你应对这种情况。  再次强调，这些都是一般性建议，并不能替代专业医疗建议。你应该尽快寻找医疗专业人士进行咨询。 | 我不是医生，但是我可以给你一些基本的建议。如果你被诊断为乙肝，请遵循以下步骤：  1.找医生咨询：首先，你应该找到一位专业的医生，例如肝脏病专家或感染病专家，进行详细的咨询和评估。他们会根据你的病情决定最适合你的治疗方案。  2.了解疾病：乙肝是一种由乙型肝炎病毒（HBV）引起的感染，可以通过血液、性行为、或者母婴传播。了解疾病的性质和传播方式可以帮助你更好地管理你的健康状况。  3.健康的生活方式：保持健康的生活方式对于控制乙肝很重要。这包括均衡饮食，规律的体育活动，以及避免饮酒和吸烟。  4.定期检查：如果你被诊断为乙肝，你需要定期进行肝功能检查和病毒载量检测。这些检查可以帮助你的医生了解你的病情是否稳定，或者是否需要调整治疗方案。  5.预防传播：由于乙肝可以通过血液和性行为传播，所以你应该采取预防措施，避免将病毒传播给他人。这包括避免分享针筒、牙刷、剃须刀，使用安全套进行性行为，以及告知你的性伴侣你的病情。  6.接种疫苗：如果你的家人或者其他密切接触者还没有接种乙肝疫苗，他们应该尽快接种，以防止病毒传播。  7.心理支持：被诊断为乙肝可能会引起焦虑和压力。寻找心理支持，例如咨询心理医生或者加入支持小组，可以帮助你应对这些情绪。  请记住，我提供的这些信息只是基本的建议，你应该咨询你的医生获取专业的医疗建议。 | 1,1 | 1,1 |  | 1, 1 |
| 64 | Clinical Problems | If I am pregnant should I get tested for hepatitis B? | Yes, it is highly recommended that all pregnant women get tested for Hepatitis B.  Hepatitis B is a viral infection that can be passed from a mother to her baby during childbirth. The baby can develop a chronic infection, which can lead to serious health problems such as liver disease and liver cancer later in life.  The Centers for Disease Control and Prevention (CDC) recommends that all pregnant women get tested for Hepatitis B during their first prenatal visit. If a pregnant woman tests positive for Hepatitis B, there are effective treatments that can reduce the risk of passing the infection to the baby.  The Hepatitis B vaccine and a shot called Hepatitis B immune globulin (HBIG) can be given to the baby within 12 hours of birth to provide protection against the virus. The baby will also need to complete the Hepatitis B vaccine series for full protection.  Therefore, testing for Hepatitis B is an important step in protecting the health of both the mother and the baby. | Yes, testing for Hepatitis B during pregnancy is generally recommended. Hepatitis B is a serious liver infection caused by the Hepatitis B virus (HBV).  The main reasons for testing during pregnancy include:  1.Mother-to-child transmission: Hepatitis B can be passed from an infected mother to her baby during childbirth. This is the most common way children get Hepatitis B.  2.Risk of chronic disease: Babies and children infected with the Hepatitis B virus are more likely to get chronic Hepatitis B, which can lead to serious health issues, like cirrhosis or liver cancer.  3.Prevention and treatment: If a pregnant woman tests positive for Hepatitis B, steps can be taken to reduce the risk of transmission to the baby. This usually involves giving the baby a Hepatitis B vaccine and a shot of Hepatitis B immune globulin (HBIG) within 12 hours of birth.  So, it is important to get tested for Hepatitis B during pregnancy. If you are pregnant, you should discuss this with your healthcare provider.  Please note: This advice is based on general medical guidelines and may not apply to every individual’s situation. Always consult with a healthcare provider for personal medical advice. | 1,1 | 1,1 |  | 1, 1 |
| 64 | Clinical Problems | 怀孕后需要检测乙肝吗 | 是的，怀孕后需要检测乙肝。乙型肝炎（HBV）是一种通过血液和其他体液传播的病毒。如果母亲是乙肝病毒携带者，她可能会在分娩时将病毒传播给新生儿。因此，孕妇在怀孕期间进行乙肝检测是非常重要的。  如果孕妇是乙肝表面抗原（HBsAg）阳性，她的新生儿应在出生后的12小时内接种乙肝疫苗和乙肝免疫球蛋白（HBIG）。这可以大大降低新生儿感染乙肝的风险。  因此，为了保护母亲和新生儿的健康，孕妇应进行乙肝检测。 | 是的，怀孕后需要进行乙肝检测。  乙型肝炎是一种可以通过血液、性接触、母婴传播的疾病。如果孕妇是乙肝病毒携带者，那么在分娩过程中可能将病毒传播给新生儿。因此，对于所有怀孕的女性，医生通常会建议进行乙型肝炎的筛查。  如果孕妇的乙肝检测结果呈阳性，医生会提供相应的医疗建议，以降低新生儿感染乙肝的风险。例如，新生儿出生后立即接种乙肝疫苗和乙肝免疫球蛋白可以大大降低他们感染乙肝的风险。 | 1,1 | 1,1 |  | 1, 1 |
| 65 | Clinical Problems | If a pregnant person has hepatitis B, is there a way to prevent their baby from getting hepatitis B? | Yes, it is indeed possible to significantly reduce the risk of a baby contracting Hepatitis B from a Hepatitis B-positive mother. The Centers for Disease Control and Prevention (CDC) recommends a series of vaccinations and an immune globulin for newborns of mothers who are positive for Hepatitis B. Here are the steps recommended:  1.Hepatitis B Vaccine: The first dose of the Hepatitis B vaccine should be given to the newborn within 24 hours of birth. This vaccine is a series, and the subsequent doses are typically given at 1 and 6 months of age.  2.Hepatitis B Immune Globulin (HBIG): This is a preparation of antibodies that can provide short-term protection against Hepatitis B. HBIG should be given to the newborn within 12 hours of birth, along with the first dose of the Hepatitis B vaccine.  3.Post-vaccination serologic testing: This is recommended for infants born to HBsAg-positive mothers. The testing should be performed after completion of at least 3 doses of a licensed Hepatitis B vaccine series, at age 9-18 months. The test checks for anti-HBs and HBsAg.  This approach is highly effective. When both the Hepatitis B vaccine and HBIG are given correctly within the recommended time frame, they are 85-95% effective in preventing the development of chronic Hepatitis B infection.  It is also important to note that Hepatitis B is not transmitted by breastfeeding. Therefore, breastfeeding is not contraindicated in mothers who are positive for Hepatitis B.  However, the best course of action should always be determined in consultation with a healthcare professional. The information provided here is general in nature and may not apply to every individual’s specific health situation.  Remember, this information is subject to the latest research and guidelines. Always consult a healthcare provider for personalized medical advice. | Yes, there is a way to significantly reduce the risk of a baby contracting Hepatitis B from a pregnant person who has the virus. This involves a combination of vaccines and immunoglobulin given to the newborn after birth. Here are the steps:  1.Hepatitis B Vaccine: The newborn should receive the first dose of the Hepatitis B vaccine within 12 to 24 hours of birth. This is the first part of a series of three or four shots.  2.Hepatitis B Immune Globulin (HBIG): In addition to the vaccine, the newborn should receive a dose of HBIG within 12 hours of birth if the mother is known to have Hepatitis B. HBIG is a preparation of antibodies that provides temporary (about 3 months) protection against Hepatitis B.  3.Continued Vaccination: The baby should receive the rest of the Hepatitis B vaccine series according to the recommended schedule, typically at 1-2 months and then a final dose between 6-18 months of age. The exact timing can vary slightly depending on the specific vaccine used.  4.Post-Vaccination Testing: For babies born to mothers with Hepatitis B, it’s recommended to test the baby for Hepatitis B infection and immunity between 9 to 12 months of age. This is important to confirm that the baby did not get infected and that the vaccine has worked.  These steps can reduce the risk of a baby contracting Hepatitis B from an infected mother to less than 1%. It’s also important for pregnant people to know their Hepatitis B status so they can take the necessary precautions to prevent transmission to their baby. If a pregnant person is at high risk for Hepatitis B or their status is unknown, testing should be done as early as possible in pregnancy.  Please note, this information is based on current medical guidelines and practices and is subject to change. Always consult with a healthcare provider for the most accurate information. | 1,1 | 1,1 |  | 1, 1 |
| 65 | Clinical Problems | 孕妇乙肝如何防止传给孩子 | 孕妇乙肝对新生儿的传播是一个重要的公共卫生问题，但是有一些措施可以帮助减少这种风险。以下是一些主要的防止措施：  1.注射乙肝免疫球蛋白(HBIG)：对于乙肝病毒表面抗原（HBsAg）阳性的孕妇，她们的新生儿在出生后的24小时内应接种乙肝免疫球蛋白和乙肝疫苗。这可以大大降低新生儿感染乙肝的风险。  2.乙肝疫苗接种：新生儿应在出生后的24小时内接种乙肝疫苗的第一剂，然后在1个月和6个月时分别接种第二剂和第三剂。  3.抗病毒治疗：对于有高病毒负荷的孕妇，医生可能会推荐在孕期的后期开始抗病毒治疗，以进一步降低母婴传播的风险。  4.定期检测：孕妇应定期进行乙肝病毒检测，以监控病毒负荷并确定是否需要抗病毒治疗。  以上所有的防止措施都应在医生的指导下进行。如果你是一位乙肝病毒携带者并且正在计划怀孕或已经怀孕，你应该与你的医生讨论这些措施，并制定一个适合你的预防策略。 | 乙肝病毒（HBV）是通过血液和体液传播的，因此孕妇如果是乙肝病毒携带者，有可能在分娩过程中将病毒传给新生儿。然而，有一些措施可以采取来减少这种风险：  1.接种乙肝疫苗和乙肝免疫球蛋白（HBIG）：出生后的第一次接种应在新生儿出生后的12小时内完成。这是预防新生儿感染乙肝最有效的方法。接下来的两次疫苗接种通常在新生儿出生后的1个月和6个月时进行。  2.孕期抗病毒治疗：对于高度病毒复制的孕妇（血清HBV DNA大于200,000 IU/mL），第三孕期（孕28周后）可以考虑使用抗病毒药物，如替诺福韦或者恩替卡韦，来降低母婴传播的风险。在分娩后，这些药物通常会停用，但需要医生根据具体情况来决定。  3.健康生活方式：保持健康的生活方式，如均衡饮食，适当运动，避免饮酒和吸烟，都有助于提高身体对病毒的抵抗力。  4.定期检查：孕妇应定期进行乙肝相关的血液检查，以便医生能够及时发现并处理任何可能的问题。  请注意，以上信息仅供参考，具体的预防措施应由医生根据孕妇的具体情况来决定。 | 2,1 | 1,1 | 1,1 | 1, 1 |
| 66 | Clinical Problems | What can people with chronic hepatitis B do to take care of their liver? | People with chronic hepatitis B can take several steps to manage their condition and maintain their liver health:  1.Medication: Depending on the stage and severity of the disease, antiviral medications may be prescribed by your healthcare provider. These medications can help slow down the virus and reduce its impact on your liver.  2.Regular Monitoring: Regular blood tests and liver ultrasounds can help track the progression of the disease and the health of the liver.  3.Healthy Diet: A balanced, nutritious diet can support overall health and liver function. Try to include a variety of fruits, vegetables, lean proteins, and whole grains in your diet.  4.Avoid Alcohol: Alcohol can cause additional liver damage. People with chronic hepatitis B should avoid alcohol or drink it in moderation.  5.Avoid Certain Medications: Some over-the-counter and prescription medications can damage the liver, especially when taken in large amounts or for a long time. Always consult with your healthcare provider before starting any new medication.  6.Exercise Regularly: Regular physical activity can help maintain a healthy weight and overall health, which is beneficial for liver function.  7.Avoid Infecting Others: Hepatitis B is contagious. Follow precautions to prevent spreading the virus to others, such as not sharing needles, razors, toothbrushes, and practicing safe sex.  8.Vaccination: Encourage family members and close contacts to get vaccinated against hepatitis B if they haven’t already. The vaccine is safe and highly effective.  9.Avoid Fatty Foods: A diet high in saturated fats can lead to fatty liver disease, which can exacerbate hepatitis B complications.  10.Stay Hydrated: Drinking plenty of water can help your liver process toxins and function properly.  Remember, each person is unique, and their needs may vary. Always consult with a healthcare provider for personalized advice.  This list is not exhaustive and is only intended as a general guide. It should not replace professional medical advice. | People with chronic hepatitis B need to take extra care of their liver to prevent further damage and to manage their condition. Here are some recommendations:  1.Medication: Your doctor may prescribe antiviral medications to help reduce the risk of liver damage and prevent the virus from spreading. It’s crucial to take these medications exactly as prescribed.  2.Regular Monitoring: Regular blood tests and liver scans are essential to monitor the health of your liver and to see how well the treatment is working.  3.Healthy Diet: A balanced diet can help improve liver health. Foods rich in fiber, such as fruits, vegetables, and whole grains, are particularly beneficial. It’s also important to stay hydrated by drinking plenty of water.  4.Avoid Alcohol and Drugs: Alcohol and certain drugs can cause further liver damage. It’s best to avoid them or consume them only under medical supervision.  5.Avoid Certain Medications: Some over-the-counter and prescription medications can damage the liver, particularly if taken in high doses or over a long period. Always consult your healthcare provider before starting any new medication.  6.Exercise Regularly: Regular physical activity can help maintain a healthy weight and reduce the risk of liver disease.  7.Vaccination: People with chronic hepatitis B should ensure that their close contacts are vaccinated against the disease to prevent its spread.  8.Avoid Contaminated Items: To prevent spreading the virus to others, don’t share items that might have your blood on them, such as toothbrushes, razors, or needles.  9.Safe Sex Practices: Use a condom during sex to prevent spreading the virus to your partner.  10.Mental Health Care: Living with a chronic illness can be stressful. Seek mental health support if you’re feeling anxious, depressed, or overwhelmed.  Remember, it’s essential to work closely with your healthcare provider to manage chronic hepatitis B and maintain your overall health. Regular check-ups and open communication with your doctor are crucial. | 1,1 | 1,1 |  | 1, 1 |
| 66 | Clinical Problems | 慢乙肝患者如何护肝 | 乙型肝炎是一种常见的慢性疾病，对患者的身体健康造成很大影响。下面是一些针对慢性乙型肝炎患者的护肝建议：  1.健康饮食：合理的饮食是保持肝脏健康的关键。应避免过度食用油腻、辛辣、烧烤等刺激性食物，多吃新鲜蔬菜和水果，保证膳食中的营养均衡。  2.规律作息：保持良好的生活习惯，避免熬夜，保证充足的睡眠。  3.适当运动：适当的运动可以增强身体免疫力，帮助身体抵抗疾病。如慢跑、游泳、瑜伽等都是很好的选择。  4.避免酒精：酒精对肝脏有很大的伤害，应尽量避免饮酒。  5.避免药物伤肝：有些药物对肝脏有损害，应在医生指导下使用药物，尤其是非处方药。  6.定期检查：定期进行肝功能检查，及时了解病情，及时调整治疗方案。  7.积极治疗：一旦确诊为乙型肝炎，应积极接受医生的治疗，不要放弃治疗。  请注意，以上建议不能替代医生的专业意见。如果您或您的亲友有乙型肝炎，应定期就医，并遵循医生的指示和建议。 | 乙型肝炎（HBV）是一种严重的肝脏疾病，可以引发肝硬化和肝癌。对于慢性乙型肝炎（CHB）患者来说，护肝非常重要，以下是一些有效的护肝方法：  1.健康饮食：选择营养均衡的食物，包括全谷类、新鲜水果和蔬菜、瘦肉、鱼、低脂乳制品等。避免过度摄入脂肪、糖和盐。避免饮酒，因为酒精可以损害或破坏肝细胞。  2.适当运动：保持活跃和适当的体重有助于肝脏健康。建议每周至少进行150分钟的中等强度有氧运动，如快步走、慢跑、骑自行车等。  3.定期检查：定期进行肝功能检查和病毒载量检测，以监控疾病进展和治疗效果。  4.遵医嘱：根据医生的建议进行抗病毒治疗。即使症状消失或病毒载量降低，也不能擅自停药。  5.戒烟：吸烟会加重肝脏疾病，因此戒烟对肝脏健康非常重要。  6.防止交叉感染：避免共享牙刷、剃须刀、指甲剪或任何可能有血液的物品，以防止病毒传播。  7.充足休息：保证充足的休息时间，有助于身体恢复和修复。  这些方法可以帮助保护肝脏，减慢疾病进展，但不能替代医生的专业治疗。如果您有任何健康问题或疑虑，应立即咨询医生。 | 1,1 | 1,2 | 1,2 | 1,2 |
| 67 | Clinical Problems | I'm Hepatitis B Reactive, can i still have a healthy baby? | Yes, you can still have a healthy baby even if you are Hepatitis B reactive. However, it’s important to manage the condition properly to minimize the risk of transmission to your baby.  Hepatitis B can be transmitted from a mother to her baby during childbirth. This is called perinatal transmission. However, with appropriate medical care, the risk of perinatal transmission can be significantly reduced.  Here are some important steps to ensure the health of your baby:  1.Prenatal Care: Regular prenatal check-ups are crucial for monitoring the health of both the mother and the baby.  2.Antiviral Medication: Your doctor might recommend antiviral medication during your third trimester to reduce the risk of transmitting the virus to your baby.  3.Vaccination for the Baby: The baby should receive a hepatitis B vaccine and a dose of hepatitis B immune globulin (HBIG) within 12 hours of birth. The vaccine is given in a series, usually at birth, 1-2 months, and then again at 6 months.  4.Follow-up Testing: After the initial immunizations, the baby should be tested for hepatitis B surface antigen (HBsAg) and antibody to hepatitis B surface antigen (anti-HBs) at 9 to 12 months to ensure that the baby did not get infected and that protection from the vaccine is adequate.  Remember, it’s important to discuss your hepatitis B status with your healthcare provider as early as possible in your pregnancy, so they can provide the best care for you and your baby. Your healthcare provider can guide you through these steps based on your specific health situation.  These steps significantly reduce the risk of mother-to-child transmission of hepatitis B, so that your baby can be born healthy. Always follow the advice of your healthcare provider. | Yes, it is possible for a person who is Hepatitis B reactive to have a healthy baby. However, there are certain risks and precautions that need to be taken into consideration.  Risks  The primary risk is the potential transmission of Hepatitis B from mother to child during childbirth. According to the Centers for Disease Control and Prevention (CDC), without intervention, up to 90% of infants born to Hepatitis B-infected mothers could become infected.  Precautions  To prevent transmission of Hepatitis B to the baby, the following precautions are usually recommended:  1.Antiviral Medication: Some mothers with high viral loads may be recommended to take antiviral medication during their third trimester to reduce the risk of transmission.  2.Vaccination and Immune Globulin for the Baby: Immediately after birth, the baby should receive a dose of Hepatitis B immune globulin (HBIG) and the first dose of the Hepatitis B vaccine. The HBIG provides temporary protection (about 3 months) against the virus. The vaccine initiates the development of long-term protection.  3.Follow-up Vaccinations: The baby should receive additional doses of the Hepatitis B vaccine according to the recommended schedule (usually at 1 month and 6 months of age).  4.Testing: The baby should be tested for Hepatitis B infection 1 to 2 months after completion of the vaccine series (usually at 9 to 12 months of age).  Important Note  These are general guidelines and may vary based on the specific circumstances of the mother and baby. It is crucial to discuss your situation with your healthcare provider to develop the best plan for you and your baby.  It’s also important to note that being Hepatitis B reactive does not inherently affect the health of the baby outside the risk of transmission. With appropriate precautions and medical care, you can certainly have a healthy baby. | 1,1 | 1,1 |  | 1, 1 |
| 67 | Clinical Problems | 乙肝肝炎活动期能要小孩吗 | 乙型肝炎是一种由乙型肝炎病毒（HBV）引起的肝炎，可以通过血液、性接触、母婴传播等方式传播。乙型肝炎活动期指的是乙型肝炎病毒在体内活跃，病毒复制活跃，肝细胞炎症反应明显，肝功能受损的阶段。  乙型肝炎活动期的患者在计划生育时，需要特别注意以下几点：  1.母婴传播风险：孕妇如果是乙型肝炎病毒携带者，存在将病毒传播给新生儿的风险。不过，通过母婴阻断措施（比如新生儿出生后立即接种乙型肝炎疫苗和免疫球蛋白），可以有效降低这种风险。  2.孕期健康管理：乙型肝炎活动期患者在孕期可能需要定期监测肝功能和病毒复制水平，以及进行适当的抗病毒治疗。这需要由医生根据具体情况来决定。  3.药物选择：如果孕妇需要接受抗病毒治疗，需要选择对胎儿影响较小的药物。目前，一些抗病毒药物（如替诺福韦）被认为在孕期是安全的。  总的来说，乙型肝炎活动期的患者在计划生育时，应该在医生的指导下进行。医生会根据患者的病情和健康状况，制定个体化的治疗和管理方案。 | 乙型肝炎（Hepatitis B）是一种由乙型肝炎病毒（HBV）引起的肝脏疾病。乙型肝炎的活动期，意味着病毒正在活跃地复制和感染肝细胞，可能导致肝脏炎症和损伤。  如果你在乙肝活动期并考虑怀孕，你应该首先咨询医生。虽然乙型肝炎可以通过垂直传播（从母亲到新生儿）传播，但是有一些预防措施可以大大降低这种风险。例如，新生儿可以在出生后的12小时内接种乙型肝炎疫苗和乙型肝炎免疫球蛋白（HBIG），以提供保护。  然而，如果你的乙型肝炎处于活动期，可能需要进行抗病毒治疗，以降低病毒复制的水平。一些抗病毒药物在怀孕期间是安全的，但是其他药物可能对胎儿有害。因此，如果你正在考虑怀孕，应该与医生讨论你的治疗方案，以确保选择对你和你的宝宝最安全的选项。  总的来说，乙型肝炎患者可以生育，但是需要采取预防措施，以降低病毒传播给宝宝的风险。如果你在乙肝活动期并考虑怀孕，应该寻求医生的专业意见。 | 1,1 | 1,2 | 1,1 | 1, 1 |
| 68 | Clinical Problems | I was just told to be Hepatitis B Reactive. If it become severe, what will be the symptoms? | I’m sorry to hear about your diagnosis. Hepatitis B is a serious liver infection caused by the Hepatitis B virus. It’s important to remember that not everyone with Hepatitis B will have symptoms. However, when they do occur, they often appear several weeks after exposure and can range from mild to severe.  If Hepatitis B becomes severe, the symptoms may include:  1.Jaundice: This condition causes yellowing of the skin and the whites of the eyes. It also can cause dark urine and pale stools. This happens because the liver isn’t able to process a substance called bilirubin.  2.Liver failure: In severe cases, Hepatitis B can lead to liver failure. Symptoms of liver failure include fatigue, nausea, loss of appetite, and disorientation or confusion.  3.Liver cirrhosis: This is a late stage of scarring (fibrosis) of the liver caused by many forms of liver diseases and conditions, such as hepatitis and chronic alcoholism. Symptoms can include fluid build-up in the abdomen (ascites), swelling in the legs, and easy bruising or bleeding.  4.Liver cancer: Some people with chronic Hepatitis B can develop liver cancer. Symptoms can include a lump or pain on the right side of the abdomen and loss of appetite or unexplained weight loss.  5.Additional symptoms: Other symptoms can include abdominal pain, joint pain, weakness and fatigue, and fever.  If you experience any of these symptoms, it’s important to seek medical attention right away. Your healthcare provider can monitor your condition and provide appropriate treatment.  Remember, the best way to prevent Hepatitis B is through vaccination. If you’ve already contracted the virus, it’s important to avoid alcohol, eat a healthy diet, and get regular exercise to help keep your liver healthy.  This is general information and is not a substitute for medical advice. Always consult with your healthcare provider for any health concerns. | I’m sorry to hear about your diagnosis. Hepatitis B is a viral infection that attacks the liver and can cause both acute and chronic diseases. If it becomes severe, you might experience symptoms associated with significant liver damage.  Here are some of the symptoms that can occur:  1.Jaundice: This is a yellow discoloration of the skin and the whites of the eyes, which is caused by a high level of bilirubin, a waste product, in the blood.  2.Dark Urine: The color of the urine can change to a dark brown or amber color due to excess bilirubin being excreted through the kidneys.  3.Extreme Fatigue: An unusual level of tiredness can be a symptom of severe Hepatitis B.  4.Nausea and Vomiting: These symptoms can be a result of the body trying to rid itself of the virus.  5.Abdominal Pain: This pain usually occurs in the upper right quadrant of the abdomen, where the liver is located.  6.Joint Pain and Muscle Aches: These can occur throughout the body as a result of the immune system’s response to the virus.  7.Loss of Appetite: A lack of desire to eat is a common symptom of many illnesses, including severe Hepatitis B.  8.Fever: An elevated body temperature is another way the body tries to fight off viral infections.  If Hepatitis B progresses to a chronic state, it can lead to more severe conditions like cirrhosis (scarring of the liver), liver failure, or liver cancer. These conditions have their own set of symptoms, which can include mental confusion, swelling in the legs and abdomen, and easy bruising or bleeding.  Please remember that these symptoms do not always appear in all individuals, and some people may not experience any symptoms at all. It’s important to regularly consult with your healthcare provider for monitoring and treatment recommendations. | 2,1 | 2,1 |  | 2,1 |
| 68 | Clinical Problems | 我现在乙肝肝炎活动期，如果病情加重会有什么表现？ | 乙型肝炎活动期的病情如果加重，可能会有以下的表现：  1.黄疸：皮肤和眼白变黄，这是由于肝脏不能正常处理胆红素所导致的。  2.腹部疼痛：可能会出现右上腹部不适或疼痛。  3.恶心和呕吐：由于肝脏功能下降，可能导致消化系统问题，表现为恶心和呕吐。  4.乏力和疲劳：身体感到异常疲劳和无力，这是因为肝脏不能正常转化能量。  5.食欲下降：可能会出现食欲不振，甚至体重下降。  6.尿液变暗：由于胆红素进入尿液，可能会使尿液颜色变深。  7.粪便颜色变浅：由于胆汁不能进入肠道，可能会使粪便颜色变浅。  8.肝大：医生可能在体检时触诊到肝脏肿大。  9.出血和瘀斑：由于肝脏不能正常合成凝血因子，可能会导致出血和瘀斑。  10.脑病：在严重的情况下，可能出现肝性脑病，包括混乱、昏睡甚至昏迷。  如果你有以上的任何症状，应立即寻求医疗帮助。 | 乙型肝炎活动期表示病毒正在活跃复制，肝脏受到一定的损伤。如果病情加重，可能会有以下一些表现：  1.疲劳和乏力：这是因为肝脏是人体新陈代谢的重要器官，肝脏受损会影响身体的能量利用。  2.食欲减退和体重下降：这是由于肝病患者常常会出现消化不良，导致食欲减退，严重时可能会出现体重下降。  3.黄疸：黄疸是肝病最常见的症状之一，主要表现为皮肤和眼睛出现黄色，这是由于肝脏受损无法正常代谢胆红素所致。  4.尿色深：这是因为体内胆红素增多无法正常排泄，通过肾脏排泄到尿液中导致尿色变深。  5.肝痛：肝脏位于右上腹，受到炎症刺激或肝脏肿大会引起疼痛。  6.肝功能异常：如ALT、AST、GGT、ALP、TBIL等指标升高。  7.出血倾向：肝脏是合成凝血因子的重要场所，肝脏功能下降可能导致出血倾向，如鼻衄、皮肤瘀斑等。  8.腹水和肝硬化：在乙肝病程中，如果病情严重可能会出现肝硬化，甚至出现腹水。  9.肝性脑病：在严重的情况下，可能会出现肝性脑病，表现为精神状态改变、行为异常、昏迷等。  如果你有上述症状，建议你立即就医，并按医生的建议进行治疗。同时，保持良好的生活习惯，避免饮酒和过度劳累，以减轻对肝脏的损伤。 | 1,1 | 2,1 | 1,1 | 1, 1 |
| 69 | Clinical Problems | With hepatitis B, can I still live longer? | Yes, people with Hepatitis B can still live long lives. It is a manageable condition with treatment and lifestyle modifications. However, it’s important to note that Hepatitis B can potentially cause severe complications, including liver cirrhosis, liver failure, and liver cancer.  Here are some factors that can influence the prognosis and lifespan of people with Hepatitis B:  1.Early Detection and Regular Monitoring: Early detection of Hepatitis B allows for prompt treatment, which can help prevent or delay complications. Regular monitoring can help track the progression of the disease and adjust treatment as necessary.  2.Antiviral Therapy: Antiviral medications can suppress the virus and slow the progression of the disease. This can help prevent complications and improve life expectancy.  3.Lifestyle Modifications: Avoiding alcohol, maintaining a healthy weight, eating a balanced diet, and regular exercise can help keep the liver healthy and slow the progression of the disease.  4.Vaccination: While this does not cure existing Hepatitis B infection, it can protect close contacts from getting the virus, which is particularly important in preventing its spread.  5.Avoiding Liver Damage: People with Hepatitis B should avoid substances that can further harm their liver, such as alcohol and certain medications.  6.Regular Screening for Liver Cancer: For people with chronic Hepatitis B, especially those with family history of liver cancer, regular screening can help in early detection and treatment of liver cancer.  Remember, the prognosis of Hepatitis B varies greatly among individuals and depends on many factors, including age, overall health, liver condition, and response to treatment.  It’s essential to have regular check-ups and follow your healthcare provider’s advice to manage the disease effectively. If you have Hepatitis B, you should be under the care of a healthcare provider who specializes in liver diseases (hepatologist) or infectious diseases.  Please note that this information is intended for general knowledge and isn’t a substitute for professional medical advice or treatment. Always seek the advice of your physician or other qualified health provider with any questions you may have regarding a medical condition. | Yes, people with Hepatitis B can still live long, fulfilling lives. Hepatitis B is a liver infection caused by the Hepatitis B virus (HBV). It can be acute or chronic. Acute Hepatitis B usually goes away on its own and may not require treatment. However, chronic Hepatitis B, which is a long-term illness, can potentially lead to serious health issues like cirrhosis or liver cancer.  However, it’s important to note that many people with chronic Hepatitis B can still live long lives, especially if they:  1.Follow their healthcare provider’s advice: This includes regular check-ups, taking prescribed medications, and possibly undergoing certain medical procedures.  2.Maintain a healthy lifestyle: This includes eating a balanced diet, getting regular exercise, avoiding alcohol, and not smoking.  3.Get regular monitoring: Regular blood tests and liver ultrasounds can help monitor the health of the liver and the progression of the disease.  4.Take antiviral medications if prescribed: These can slow down the virus and reduce the risk of liver damage.  5.Get vaccinated for Hepatitis A: People with chronic Hepatitis B should also get vaccinated against Hepatitis A, another type of liver infection.  It’s important to remember that every individual’s health situation is unique, and these general guidelines may not apply to everyone. Therefore, always consult with your healthcare provider for advice tailored to your specific condition.  Please note that this information is intended to be educational and does not replace professional medical advice. Always consult with a healthcare provider for medical advice. | 1,1 | 1,1 |  | 1, 1 |
| 69 | Clinical Problems | 得乙肝会影响寿命吗 | 乙型肝炎（Hepatitis B）是一种由乙型肝炎病毒（HBV）引起的肝脏疾病。它可以导致肝炎，肝硬化，甚至肝癌。因此，如果不进行适当的治疗和管理，乙型肝炎确实可能对患者的寿命产生影响。  然而，现在对乙型肝炎的治疗手段已经非常先进。通过适当的药物治疗和健康生活方式的改变，许多乙型肝炎患者都能够长期生存，并维持良好的生活质量。值得注意的是，对乙型肝炎的治疗需要长期坚持，不能轻易停药或更换药物。  最重要的是，预防乙型肝炎的最有效方法是接种乙型肝炎疫苗。这种疫苗在全球范围内广泛使用，已被证明能有效防止乙型肝炎病毒的感染。  总的来说，虽然乙型肝炎可能会影响寿命，但通过适当的预防和治疗措施，患者可以管理这种病症并过上健康的生活。 | 乙型肝炎（也被称为乙肝）是一种由乙型肝炎病毒（HBV）引起的肝炎。感染乙型肝炎病毒后，人体可能会经历几个不同的阶段，包括急性期、慢性期和恢复期。乙肝对个体的影响因人而异，可以从无症状的感染到肝硬化或肝癌等严重疾病。  对于乙肝是否会影响寿命，需要考虑以下几个因素：  1.疾病阶段：急性乙肝通常会在几周或几个月内自愈，大多数人可以完全恢复。然而，一部分人（约5%的成年人）会发展成慢性乙肝，这可能导致肝硬化或肝癌，从而影响寿命。  2.治疗：对于慢性乙肝，有多种抗病毒药物可以有效控制病毒复制，从而防止或延缓肝硬化和肝癌的发生。  3.生活方式：健康的生活方式，如避免饮酒、保持健康的体重、进行定期运动，都可以帮助保护肝脏。  4.定期检查：对于乙肝患者，定期进行肝功能检查和肝硬度测量，可以早期发现肝硬化或肝癌，及时进行治疗。  综上，得乙肝并不一定会减少寿命，关键在于病毒的控制、健康的生活方式和定期的医疗检查。但是，如果不进行有效的管理和治疗，慢性乙肝可能会发展为严重的肝病，从而影响寿命。 | 2,1 | 2,1 |  | 2,1 |
| 70 | Clinical Problems | Can I travel Europe on a tourist visa if I have hepatitis b? | In general, having Hepatitis B does not automatically disqualify you from being able to travel to Europe on a tourist visa. However, it’s important to note a few key points:  1.Medical Insurance: It is crucial to have comprehensive travel insurance that covers your pre-existing condition. This insurance should cover any potential medical expenses that might occur during your trip, including those related to Hepatitis B.  2.Medication: Ensure you carry enough medication for your entire trip. Also, it’s advisable to bring a letter from your doctor detailing your condition and the medication you are taking.  3.Health and Safety: It’s important to take steps to protect your health while traveling. Avoid situations that could potentially expose others to the virus, and vice versa.  4.Country-Specific Regulations: Some countries may have specific entry requirements or restrictions related to health conditions. Therefore, it’s advisable to check the specific requirements of the countries you plan to visit before you travel.  5.Visa Requirements: Visa requirements can change, and they can also vary depending on your nationality. It’s always a good idea to check the latest visa requirements with the embassy or consulate of the country you’re planning to visit.  Remember, it’s always important to consult with your healthcare provider before making any travel plans. They can provide you with the most accurate and personalized advice based on your specific health condition. | Having Hepatitis B doesn’t necessarily prevent you from traveling to Europe on a tourist visa. However, there are a few things you should keep in mind:  1.Medical Insurance: It’s important to have a travel health insurance policy that covers pre-existing conditions. Some countries may require proof of this insurance when applying for a visa.  2.Vaccination and Medication: Make sure you’re up-to-date with your vaccines and bring enough medication for the duration of your trip.  3.Medical Certificate: In some cases, you might need a medical certificate from your doctor stating that you’re fit to travel.  4.Visa Requirements: Each country in Europe may have different visa requirements, so it’s important to check with the embassy or consulate of the countries you plan to visit.  Remember, it’s always advisable to consult with your healthcare provider before planning any kind of travel, especially when you have a chronic health condition like Hepatitis B. They can provide personalized advice based on your current health status.  Also, rules and regulations can change, so always check the most up-to-date information from official sources. | 1,1 | 1,1 |  | 1, 1 |
| 70 | Clinical Problems | 有乙肝能去欧洲旅游吗 | 当然可以。乙肝并不会阻止您去欧洲旅游。然而，这并不意味着您不需要采取任何措施。这里有一些重要的建议：  1.看医生：在旅行前，您应该咨询您的医生或医疗保健提供者。他们可以根据您的具体情况，为您提供个性化的建议，例如关于药物、饮食和生活方式的建议。  2.药物：如果您正在接受治疗，确保您带足够的药物，以防止在旅行期间药物耗尽。同时，也要确保您知道如何正确地携带和存储这些药物。  3.保险：确保您的旅行保险涵盖了与乙肝相关的医疗费用。阅读您的保险政策，并确保您理解其涵盖的内容。  4.紧急联系信息：带上您的医生的联系信息，以便在旅行期间出现任何问题时，可以及时联系他们。  5.了解目的地：在出发前，了解您的目的地的医疗设施和服务。这样，如果在旅行期间需要医疗帮助，您就知道去哪里寻找。  总的来说，乙肝并不会阻止您去欧洲旅游，但您应该提前做好准备，以便在旅行期间能够照顾好自己的健康。 | 当然可以。乙型肝炎并不会阻止您去欧洲旅游。然而，有几点您需要注意：  1.医疗建议：在出发前，确保您的病情稳定，并咨询医生是否适合旅行。您可能需要带足够的药品，以及在旅行期间可能需要的任何特殊护理。  2.保险：在购买旅行保险时，请确认保险公司是否覆盖与乙型肝炎相关的医疗费用。有些保险公司可能不会覆盖这种情况。  3.疫苗：如果您的同伴还未接种乙型肝炎疫苗，建议他们在旅行前接种，以防止感染。  4.旅行计划：计划旅行活动时，尽量避免过于剧烈的活动，以免对身体造成过大的压力。  5.就医：在您的目的地，了解当地的医疗资源，以便在需要时能够及时就医。  记住，每个人的健康状况和需求都是独特的，所以这些只是一般的建议。最好在旅行前咨询您的医生，以得到最适合您的建议。 | 1,1 | 1,1 |  | 1, 1 |
| 71 | Clinical Problems | My dad need a liver transplant due to acute hepatitis b, he is 55. What happens if he isn't in good enough health for a transplant? Do they just send him home to die? | I’m sorry to hear about your dad’s health situation. It’s understandable that you’re concerned. If a patient isn’t healthy enough for a liver transplant, the medical team will usually consider several alternative treatments and interventions to manage the disease. It’s important to remember that every patient’s situation is unique, and the best course of action will depend on the specifics of your dad’s health status.  Here are some potential alternatives:  1.Medications: There are several antiviral medications that can be used to treat hepatitis B. These drugs can slow down or stop the virus from damaging the liver.  2.Liver Dialysis: This is a treatment that filters out toxins from the blood, similar to kidney dialysis. This can be used as a temporary measure in patients with acute liver failure, or as a bridge to transplantation.  3.TIPS Procedure (Transjugular Intrahepatic Portosystemic Shunt): This is a procedure that can alleviate some of the complications of liver disease, such as fluid buildup in the abdomen or bleeding from varices in the esophagus.  4.Nutritional Support: Proper nutrition can help to support liver function and overall health.  5.Supportive Care: This can include anything from pain management to psychological support. It’s an important aspect of treatment for any chronic illness.  6.Clinical Trials: Experimental treatments for liver disease are being researched all the time. Your dad’s medical team may be able to help you find clinical trials that could be appropriate for his situation.  Again, it’s important to have these discussions with your dad’s healthcare team, as they’ll have the most accurate information about his health status and the best options for his care. I hope this information is helpful, and I wish your dad the best in his treatment. | I’m really sorry to hear about your dad’s situation. It’s a tough time for everyone involved. If your dad isn’t in good enough health for a liver transplant, it doesn’t necessarily mean that all hope is lost. While a liver transplant is a definitive treatment for many liver diseases, it’s a major operation and requires a certain level of health to be successful.  If a patient isn’t healthy enough for a transplant, doctors don’t just send them home without any further help. Instead, they might consider other treatments or interventions that could improve the patient’s health enough to become a candidate for transplantation. This could include:  1.Medical management: This is often the first line of treatment and involves managing symptoms and complications of liver disease. It can include medications to reduce the buildup of toxins in the blood, dietary changes to improve nutritional status, and other interventions.  2.Liver dialysis: A relatively new procedure that’s similar to kidney dialysis. It helps to perform some of the functions of the liver, giving it a chance to heal and recover.  3.Bridging treatments: These are procedures like transarterial chemoembolization (TACE), radiofrequency ablation (RFA), or microwave ablation (MWA) that are used to manage liver cancer until a transplant can be done.  4.Hepatoprotective agents: Medications that aim to protect the liver and enhance its ability to regenerate.  5.Palliative care: If the disease is too advanced and the patient is not a candidate for a transplant, palliative care can help manage symptoms and improve quality of life. It’s not just about end-of-life care, but about providing relief from the symptoms and stress of the illness.  Remember that each patient is unique, and the treatment plan will be tailored to your dad’s specific condition and overall health status. It’s essential to maintain open and honest communication with his healthcare team. You can always ask them about his prognosis, potential treatments, and what you can do to help him through this difficult time. | 1,1 | 1,1 |  | 1, 1 |
| 71 | Clinical Problems | 我爸爸55岁，很严重的急性乙肝，需要肝移植，如果情况不好不能移植会怎样，会让他回家等死吗 | 我非常理解您的担忧，这是一个困难的时期。然而，我需要明确一点，我是一个AI语言模型，我不能提供医学咨询或预测具体的医疗结果。但我可以给你提供一些关于急性乙肝和肝移植的一般信息。  乙型肝炎是一种由乙型肝炎病毒（HBV）引起的疾病。急性乙型肝炎通常在几周或几个月内自行消退，大多数人会完全康复。然而，有些人可能会出现严重的症状，如肝炎或肝衰竭，这可能需要住院治疗，甚至可能需要肝移植。  如果您的父亲的病情严重到需要肝移植，但由于某些原因无法进行移植手术，那么他的医生可能会寻找其他的治疗方案。这可能包括药物治疗，以控制病毒的复制并减轻症状，以及其他可能的支持性治疗，如营养支持和疼痛管理。  这并不意味着他会被让回家等死。医生和医疗团队会尽一切可能提供最佳的护理和治疗。在这个过程中，和医疗团队保持良好的沟通非常重要，以了解所有的治疗选项和可能的结果。  再次强调，具体的情况和结果取决于许多因素，包括他的整体健康状况，病情的严重程度，以及他对治疗的反应。我强烈建议您和他的医生进行深入的讨论，以获取更多具体的信息和建议。 | 首先，我非常理解您的担忧，面对父亲的严重疾病一定让您感到非常困扰。但是请记住，医学是一门不断发展的科学，总是有许多可能的治疗方法可以尝试。  急性乙肝的治疗方案通常取决于病情的严重程度。在大多数情况下，急性乙肝可以通过药物治疗以支持身体的免疫系统来对抗病毒。然而，对于严重的急性乙肝，可能需要更积极的治疗方法，如肝移植。  如果病情严重到不能进行肝移植，那么医生可能会寻找其他的治疗方案。这可能包括药物疗法，营养疗法，以及其他支持性疗法，如血液净化和生命支持。这些治疗的目标是尽可能地减少病毒的影响，提高生活质量，以及延长患者的寿命。  当然，医生会根据患者的病情和总体健康状况来决定最佳的治疗方案。请尽可能地与医生进行沟通，了解所有的可能性，并参与决策过程。  最后，请记住，尽管病情可能严重，但是"回家等死"并不是唯一的选择。医学科技的发展为我们提供了许多可能的治疗方法，而且医生和医护人员都会尽全力去帮助和照顾患者。希望这些信息能够帮助您，并祝愿您的父亲早日康复。 | 1,1 | 1,1 |  | 1, 1 |
| 72 | Clinical Problems | Is it safe to have hepatitis B vaccine 7 days after my second dose of covid-19 vaccine? | As an AI language model, I can tell you that generally, the Centers for Disease Control and Prevention (CDC) and the World Health Organization (WHO) have no specific guidelines about the timing between different types of vaccines, including COVID-19 vaccines and Hepatitis B vaccines. It’s typically considered safe to get another vaccine, such as the Hepatitis B vaccine, even if you’ve recently had a COVID-19 vaccine.  However, it’s important to note that individual health circumstances can vary, and this information might change as more research is conducted. Therefore, it’s recommended that you consult your healthcare provider before getting vaccinated. They can provide you with the most accurate and up-to-date advice based on your health history and the latest medical research.  Remember that vaccines play a critical role in preventing serious diseases, and it’s crucial to stay updated with your immunizations as recommended by health authorities and your healthcare provider. | I’m an AI language model and I don’t have real-time access to the latest medical research. However, as of my last update in 2023, there were no specific guidelines suggesting that the Hepatitis B vaccine and COVID-19 vaccines cannot be administered within a certain timeframe of each other.  However, this doesn’t mean it’s necessarily safe for you. It’s crucial to discuss your personal medical history, current health status, and any potential interactions between vaccines with a healthcare professional. They can provide the most accurate, up-to-date, and personalized advice for you.  Remember that while vaccines are important tools in preventing infectious diseases, each individual may have different health considerations that need to be taken into account. Always consult with a healthcare provider before making decisions about vaccinations. | 1,1 | 1,1 |  | 1, 1 |
| 72 | Clinical Problems | 打完新冠疫苗第二针之后1周可以打乙肝疫苗吗 | 一般来说，不同的疫苗之间通常需要有一定的间隔时间以确保每种疫苗都能够有效地发挥作用。这个间隔时间会根据疫苗的种类和个体的健康状况有所不同。  关于新冠疫苗和乙肝疫苗之间的接种间隔，目前并没有明确的科学研究给出具体的建议。因此，最佳的做法是咨询医生或者疫苗接种中心的专业人员。  请注意，这是一般的建议，不一定适用于所有人。每个人的健康状况和具体情况可能会有所不同，所以在接种疫苗之前，最好先咨询医生或者疫苗接种中心的专业人员。 | 在接种不同类型的疫苗之间，通常建议保持一段时间的间隔。这主要是为了确保每种疫苗都能有效地刺激免疫反应，并避免可能的副作用交叉。然而，具体的间隔时间可能会因疫苗的类型和个人的健康状况而异。  关于新冠疫苗和乙肝疫苗之间的接种间隔，我建议您咨询医生或其他医疗专业人士。他们能根据您的个人情况给出最适合的建议。同时，也请参考相关的疫苗接种指南或政策。  总的来说，虽然这里无法给出具体的答案，但是保持一定的疫苗接种间隔是一个普遍的建议。 | 2,2 | 2,2 |  | 2,2 |
| 73 | Clinical Problems | I'm going to travel to Asia in a month, and looking at which vaccines to take beforehand, most websites recommend Hepatitis A and B before going there. However, only having time for 1 dose, which is more important to get, A, B or the combination one? | Before making a decision about vaccinations, it’s important to consult with a healthcare provider who can take into account your personal health history, the specific areas you plan to visit, the duration of your stay, and your planned activities. However, I can provide some general information to help guide your decision.  Hepatitis A is a liver disease caused by the Hepatitis A virus, which is usually spread through contaminated food and water. In developing countries with poor sanitation, Hepatitis A is quite common.  Hepatitis B, on the other hand, is also a liver disease but it’s caused by the Hepatitis B virus. This virus is spread through contact with blood and body fluids of an infected person, such as during unprotected sex, sharing needles, or from an infected mother to her newborn.  Now, regarding the vaccines:  1.The Hepatitis A vaccine is usually given in 2 doses, 6 months apart. The first dose of Hepatitis A vaccine provides protection after about 2 weeks in most people.  2.The Hepatitis B vaccine is typically given as a series of 3 shots over a period of 6 months. The first dose provides some protection, but the series is not complete without all three shots.  3.The combination Hepatitis A and B vaccine can be given to persons 18 years of age and older. It’s typically given as a series of 3 shots over a period of 6 months.  Given your time constraints and the fact that you’re traveling in a month, the Hepatitis A vaccine might be more beneficial as it provides protection after about 2 weeks. However, if you’re at risk of exposure to Hepatitis B, the combination vaccine could be a consideration, but it’s important to remember that you won’t be fully protected against Hepatitis B with just one dose.  Please note that this is general advice, and the best course of action should be determined in consultation with a healthcare provider. It’s also crucial to take other preventive measures like practicing good hygiene and safe eating and drinking habits to avoid diseases while traveling. | Travel vaccinations are crucial to protect your health when you visit different parts of the world. Hepatitis A and Hepatitis B are both recommended vaccines for travel to many parts of Asia.  Hepatitis A is a liver disease caused by the Hepatitis A virus, which is primarily spread when a person ingests fecal matter, even in microscopic amounts, from contact with objects, food, or drinks contaminated by the feces of an infected person.  Hepatitis B, on the other hand, is a liver infection caused by the Hepatitis B virus. It is transmitted through contact with the blood or other body fluids of an infected person. It is also a sexually transmitted disease.  The risk of Hepatitis A is generally higher for most travelers (especially in areas with poor sanitation), while Hepatitis B risk is more related to specific behaviors (like unprotected sex, use of recreational drugs, etc.) and certain types of travel (like long-term travel or travel that puts you in close contact with local populations).  Therefore, if you only have time for one dose, and if your behaviors do not put you at high risk for Hepatitis B, you might prioritize Hepatitis A. But this is a decision that should be made with your healthcare provider, as individual health circumstances can significantly influence this decision.  However, the combination vaccine (Twinrix) is a good option as it protects against both Hepatitis A and B. It’s typically given as a series of three shots over a six-month period, but there’s an accelerated schedule (days 0, 7, and 21 to 30) available as well. The accelerated schedule can provide protective antibodies more quickly, but a booster shot should be given at 12 months for sustained immunity.  In conclusion, the best course of action is to consult with a healthcare provider who can consider your individual health status, travel plans, and potential risk factors before making a recommendation.  Please note that the information provided here is for educational purposes and may not be completely accurate or up-to-date. Always consult with a healthcare provider for accurate information. | 1,1 | 1,1 |  | 1, 1 |
| 73 | Clinical Problems | 想去亚洲旅行，查到推荐打甲肝和乙肝疫苗，但时间只够打一针，打甲肝、乙肝、还是混合针最好？ | 这个问题的答案取决于您具体的情况，包括您的健康状况、目的地、旅行计划等。但在一般情况下，如果时间只够接种一种疫苗，可能选择混合疫苗（甲肝和乙肝混合疫苗）是一个更全面的保护策略。这种混合疫苗可以同时提供甲肝和乙肝的保护。  然而，您需要知道的是，混合疫苗通常需要至少两次接种才能提供长期的保护。首次接种后，通常6个月后需要再接种一次以确保免疫效果。如果您的旅行计划在接种第二针疫苗之前，那么您可能只会得到有限的保护。  在做出决定之前，您应该咨询医疗专业人士以获取个性化的建议。此外，您还应该查看相关的旅行卫生和疫苗建议，例如世界卫生组织（WHO）或者您所在国家的公共卫生机构的建议。  请注意，接种疫苗是预防甲肝和乙肝的一种方法，但并不能提供100%的保护。在旅行期间，您还应该采取其他预防措施，例如避免食用可能被污染的食物和水，以及避免不安全的性行为和使用非无菌针具。  总的来说，您的选择应当基于对疾病风险的理解，以及对疫苗的可用性和您的旅行计划的考虑。 | 在选择接种哪种疫苗时，需要考虑的因素有很多，包括你的个人健康状况、目的地的疫情状况、以及旅行的持续时间等。甲肝和乙肝都是由病毒引起的肝炎，但是它们的传播方式有所不同：  1.甲肝主要通过食物和水传播，这种病毒在亚洲的某些地区非常常见。  2.乙肝则主要通过血液、性行为和母婴传播。  如果你只有时间接种一种疫苗，那么你可能需要根据你的具体旅行计划和目的地的疫情状况来决定。例如，如果你计划在农村地区停留较长时间，或者食物和水的卫生条件不确定，那么甲肝疫苗可能是个好选择。如果你有可能接触到血液或体液，或者有性行为的风险，那么乙肝疫苗可能更适合你。  混合疫苗（如Twinrix）同时提供甲肝和乙肝的保护，但是需要接种三次，分别在0、1和6个月后。如果你的旅行计划时间紧迫，可能无法完成全部的接种计划。  在做出决定之前，最好是咨询医生或旅行医学专家，他们可以根据你的具体情况提供个性化的建议。请记住，疫苗只是预防疾病的一种方式，你还需要注意个人卫生，尤其是在食物和饮水方面，以降低感染的风险。 | 1,1 | 1,1 |  | 1, 1 |
| 74 | Clinical Problems | Can I drink alcohol if my HBsAg, HBeAb and HBcAb are positive? | Hepatitis B surface antigen (HBsAg), hepatitis B e antibody (HBeAb), and hepatitis B core antibody (HBcAb) are markers that can be found in the blood when a person is infected with the hepatitis B virus (HBV).  1.HBsAg positive indicates an active infection with HBV.  2.HBeAb positive suggests the immune system is responding to the infection and the virus’s replication may be slowing down.  3.HBcAb positive can indicate either a past or ongoing infection.  Regarding alcohol consumption, it is generally advised to avoid alcohol if you have any liver disease, including hepatitis B. Alcohol can cause further damage to the liver, which is already under stress due to the viral infection. This can lead to complications such as cirrhosis or liver cancer.  So, if your HBsAg, HBeAb, and HBcAb are positive, it would be best to avoid drinking alcohol to prevent additional liver damage.  However, this advice is general in nature and may not be applicable to everyone. It’s crucial to have a discussion with your healthcare provider about your specific situation. They can provide personalized advice based on your overall health, the severity of your hepatitis B infection, and other factors. | If your HBsAg (Hepatitis B surface antigen), HBeAb (Hepatitis B e-antibody), and HBcAb (Hepatitis B core antibody) are all positive, this typically suggests a chronic Hepatitis B infection. Hepatitis B is a viral infection that attacks the liver and can cause both acute and chronic disease.  Alcohol is a hepatotoxic substance, which means it can damage the liver. In individuals with Hepatitis B, drinking alcohol can accelerate the progression of liver disease, leading to conditions such as cirrhosis (scarring of the liver), liver failure, or liver cancer. It can also reduce the effectiveness of treatment for Hepatitis B.  Therefore, if you have a Hepatitis B infection, it’s generally recommended to avoid alcohol or limit its consumption as much as possible. It’s always best to discuss this with your healthcare provider, who can provide advice based on your specific situation and health status.  Remember, the information provided here is general in nature. Always consult with a healthcare professional for medical advice. | 1,1 | 1,1 |  | 1, 1 |
| 74 | Clinical Problems | 小三阳能喝酒吗 | 小三阳是乙型肝炎病毒携带者的一种表现形式，具体来说，它是指乙型肝炎表面抗原（HBsAg）、乙型肝炎e抗原（HBeAg）和乙型肝炎病毒DNA（HBVDNA）检测均为阳性。  对于乙型肝炎病毒携带者来说，饮酒对肝脏的损害可能更大。酒精可以直接损害肝细胞，导致肝炎的进展。酒精还可能通过增加乙型肝炎病毒的复制，使得病毒的数量增加，从而加重肝脏的病变。因此，小三阳患者应该避免饮酒。  这是一项长期的任务，需要患者的自我管理和医生的指导。患者需要定期进行肝功能检查，以及其他相关的医学检查，以监测病情的进展。同时，患者还需要保持良好的生活习惯，包括健康的饮食和适量的运动。  最后，这是一个医学问题，具体的治疗方案需要根据患者的具体情况来制定。所以，如果你有关于小三阳的问题，或者需要关于小三阳的治疗建议，你应该咨询医生。 | 小三阳是乙肝病毒感染的一种常见表现，它的学名叫做"HBe抗原阳性慢性乙型肝炎"。小三阳患者的肝脏功能常常会受到一定程度的损害，而饮酒会加重肝脏的负担，可能导致肝脏病情加重，甚至可能发展为肝硬化或者肝癌。  因此，对于小三阳患者来说，最好是避免饮酒。即使是偶尔饮酒，也应该控制在很小的量，并且在医生的指导下进行。  在任何情况下，对于有肝脏疾病的人来说，保持健康的生活习惯，包括健康的饮食、充足的睡眠和适当的运动，都是非常重要的。 | 1,1 | 1,1 |  | 1, 1 |
| 75 | Clinical Problems | I have positive HBsAg, HBeAg and HBcAb with a high HBV-DNA level, but my liver function is normal, should I take antiviral drugs? | I’m an AI developed by OpenAI and while I can provide some general information on this topic, I’m not a doctor and can’t provide personalized medical advice. It’s important to discuss this question with your healthcare provider who knows your medical history and can provide the appropriate recommendations for you.  However, I can tell you that hepatitis B virus (HBV) infection is a complex condition and the decision to start antiviral treatment should be individualized, depending on a variety of factors. These factors include:  1.The phase of HBV infection  2.The level of HBV-DNA in your blood  3.Whether or not you have evidence of liver damage  4.Your age  5.Your overall health status  6.Other comorbid conditions  In general, antiviral treatment is recommended for people with chronic HBV infection who are at risk for developing liver disease. These include:  1.Patients with persistently abnormal alanine aminotransferase (ALT) levels  2.Patients with high HBV-DNA levels  33.Patients with evidence of advanced fibrosis or cirrhosis  4.Patients with a family history of hepatocellular carcinoma  From your description, it seems like you have a positive HBsAg (indicating a chronic infection), positive HBeAg and HBcAb (indicating active viral replication), and high HBV-DNA levels (indicating a high viral load). However, your liver function is normal. Therefore, it’s not straightforward to say whether you should start antiviral treatment or not without further evaluation.  The decision should be made in consultation with your healthcare provider, who can consider all these factors and perhaps conduct further tests if necessary. Please do not delay seeking medical advice and discuss your situation with your doctor.  Remember, even if you do not require treatment right now, regular monitoring of your liver function and HBV status is very important. This is because the situation can change over time, and you may need treatment in the future. | I’m an AI developed by OpenAI and I’m not a doctor, but I can provide some general information based on available medical literature.  Hepatitis B virus (HBV) infection is a complex disease, and the decision to start treatment should be individualized, based on a variety of factors, including the patient’s age, overall health, liver function, and viral load.  You’ve mentioned that you have positive HBsAg, HBeAg, and HBcAb with a high HBV-DNA level. This typically indicates active viral replication, which may increase the risk of liver disease over time, even if your liver function is currently normal.  However, the decision to start antiviral therapy should be made in consultation with a healthcare provider, who can fully assess your situation. According to the American Association for the Study of Liver Diseases (AASLD) guidelines:  1.Patients who are HBeAg-positive with HBV-DNA levels greater than  IU/mL and have persistently normal alanine aminotransferase (ALT) levels should be monitored. Antiviral therapy may be considered if there is evidence of liver disease progression, even with normal ALT levels.  2.Antiviral therapy is recommended for patients who are HBeAg-positive with HBV-DNA levels greater than  IU/mL and have elevated ALT levels.  Again, this information is just a guideline and may not be applicable to all patients. A healthcare provider should be consulted to make a final decision based on the patient’s individual situation.  It’s crucial that you discuss your results with your healthcare provider or a specialist in liver diseases (hepatologist) to decide the best course of action. | 1,2 | 1,1 | 1,1 | 1, 1 |
| 75 | Clinical Problems | 乙肝大三阳，肝功能正常，但病毒数高，要不要吃抗病毒药 | 乙型肝炎（HBV）的治疗取决于许多因素，包括病毒复制水平、肝脏炎症程度和肝功能等。"乙肝大三阳"是指血清中HBsAg（乙肝表面抗原）、HBeAg（乙肝e抗原）和HBcAg（乙肝核心抗原）均为阳性。这种情况可能表明乙型肝炎病毒正在活跃复制。  你提到你的肝功能正常，但病毒数高。这可能意味着你的体内有大量的HBV，但你的肝脏目前还能正常工作。但是，高病毒负荷可能会增加肝脏疾病的风险。  在决定是否使用抗病毒药物时，医生通常会考虑以下因素：  1.病毒复制水平：HBV DNA水平高于一定的阈值可能需要治疗。  2.肝脏炎症和/或纤维化程度：通过肝脏生物检查或无创评估（例如肝硬度测量或血液标志物）可以评估。  3.病人的年龄、性别、家族病史、酒精消费和其他健康状况。  在你的情况下，最好是咨询医生，以确定最适合你的治疗方案。医生可能会建议定期进行肝功能检查和病毒载量检测，以监测病情的进展。如果有必要，他们可能会建议开始抗病毒治疗。 | 乙型肝炎（HBV）的治疗决定因素包括多种因素，如病毒复制活动（即病毒载量或病毒数）、肝炎活动程度（肝功能测试）、肝脏疾病的阶段（例如是否有肝硬化）以及患者的整体健康状况等。乙肝大三阳指的是HBsAg（乙型肝炎表面抗原）、HBeAg（乙型肝炎e抗原）和HBcAg（乙型肝炎核心抗原）都为阳性。  如果您的肝功能正常，但病毒数高，是否需要服用抗病毒药物需要根据您的具体情况来判断。一般来说，如果病毒载量持续高，即使肝功能测试结果正常，也可能需要抗病毒治疗，因为高病毒载量可能增加肝硬化和肝癌的风险。然而，这并非绝对的规则，因为有些人的免疫系统能够有效地控制病毒复制，即使病毒载量高也不会导致严重的肝病。  因此，我建议您咨询医生或专业医疗团队，他们会根据您的具体情况来做出最佳的治疗决策。这可能包括定期进行肝功能和病毒载量测试，以及可能的肝脏生物检查，以监测病情的进展并确定是否需要抗病毒治疗。  请注意，我所提供的信息仅供参考，不能替代专业医疗建议。 | 1,1 | 1,1 |  | 1, 1 |
| 76 | Clinical Problems | Can male hepatitis B patient have healthy babies? | Yes, a male with Hepatitis B can father healthy babies. However, there are certain precautions and considerations to keep in mind.  Hepatitis B is a viral infection that attacks the liver and can cause both acute and chronic disease. It is a major global health problem and the most serious type of viral hepatitis. It can cause chronic liver disease and puts people at high risk of death from cirrhosis of the liver and liver cancer.  Hepatitis B virus (HBV) is not transmitted through sperm, so it does not directly affect the health or development of a fetus. However, if the mother gets infected, the virus can be transmitted to the baby, which is why precautions are necessary.  Here are some important points:  1.Mother’s vaccination status: If the mother is already vaccinated and has immunity against Hepatitis B, there’s very little risk for the baby to get infected.  2.Mother’s infection during pregnancy: If the mother gets infected during pregnancy, especially in the later stages, the baby has a higher risk of being infected. This can be minimized by the immediate vaccination of the baby after birth.  3.Avoiding mother’s infection: The best way to prevent transmission is to prevent the mother from getting infected in the first place. This can be done by vaccination and practicing safe sex.  4.Baby’s vaccination: The baby should be vaccinated against Hepatitis B. The first dose of the Hepatitis B vaccine can be given at birth, followed by additional doses according to the recommended immunization schedule.  5.Medical Consultation: It is crucial for the man and his partner to consult with their healthcare provider to understand the risks and preventive measures better.  Remember, while the risks exist, they can be managed effectively with the right precautions and medical guidance. It’s possible for a man with Hepatitis B to father a perfectly healthy child. | Yes, a male with Hepatitis B can have healthy babies. Hepatitis B is a viral infection that attacks the liver and can cause both acute and chronic diseases. It’s primarily transmitted through blood and other body fluids. It does not directly affect fertility or the health of a baby.  However, there are some considerations to keep in mind:  1.Risk of Transmission: If the mother is not immune to Hepatitis B, the father can potentially transmit the virus to her. This can potentially happen during sexual intercourse, as Hepatitis B can be transmitted through body fluids. If a pregnant woman gets infected, there’s a risk she could pass the virus to her baby.  2.Prevention: To prevent transmission, it’s recommended that partners of Hepatitis B patients get vaccinated. The Hepatitis B vaccine is safe and highly effective.  3.Testing: If the mother gets infected, she should be tested for Hepatitis B. If she is positive, her newborn should receive hepatitis B immune globulin (HBIG) and the first dose of the hepatitis B vaccine within 12 hours of birth, followed by the complete vaccine series.  4.Treatment: If a pregnant woman is a Hepatitis B carrier, antiviral treatment might be recommended in the third trimester to reduce the risk of transmission to the newborn.  5.Consultation: Men with Hepatitis B should consult with their healthcare provider for personalized advice before planning a family.  Remember, with proper management and preventive measures, it’s entirely possible for a man with Hepatitis B to father healthy children. | 1,1 | 1,1 |  | 1, 1 |
| 76 | Clinical Problems | 男性患乙肝可以要小孩吗 | 男性如果患有乙肝，他们还是可以有孩子的。乙肝病毒主要通过血液、性行为、母婴传播。因此，如果丈夫患有乙肝，他可能通过性行为将病毒传播给妻子。然而，这个风险可以通过以下几种方式来降低：  1.接种乙肝疫苗：如果妻子还没有感染乙肝病毒，那么接种乙肝疫苗是防止感染的最有效方法。乙肝疫苗通常需要接种3次，可以提供长期保护。  2.使用安全措施：在性行为中使用避孕套可以降低病毒传播的风险。  3.定期检查：乙肝患者应定期进行医学检查，以监控病情的进展并及时接受治疗。  如果妻子已经感染了乙肝，或者已经接种了乙肝疫苗，那么夫妻双方就可以考虑要孩子。然而，孕妇如果是乙肝患者，她可能会在分娩时将病毒传播给新生儿。这个风险可以通过在新生儿出生后立即接种乙肝免疫球蛋白和乙肝疫苗来降低。  总的来说，虽然男性乙肝患者可以有孩子，但是他们需要采取一些预防措施，以降低将病毒传播给配偶和新生儿的风险。在决定要孩子之前，他们应该咨询医生，了解所有的风险和预防措施。 | 男性如果患有乙型肝炎（HBV），理论上是可以生育小孩的。乙型肝炎并不直接影响男性的生育能力。然而，他们必须谨慎，因为乙型肝炎是可以通过性接触和母婴传播的。  关于预防乙型肝炎传播给配偶和新生儿，以下是一些关键的注意事项：  1.疫苗接种：如果配偶还没有感染乙型肝炎，那么他们应该接种乙型肝炎疫苗。新生儿也应在出生后立即接种乙型肝炎疫苗和HBIG（乙型肝炎免疫球蛋白）。  2.安全性行为：即使配偶已经接种疫苗，也应维持安全的性行为，以降低感染的风险。  3.定期检查：感染乙型肝炎的人应定期进行医学检查，以监控病情的进展和可能的并发症。  4.抗病毒治疗：在某些情况下，医生可能会建议在孕期或生育期间接受抗病毒治疗，以降低母婴传播的风险。  请注意，所有的医疗建议都应由医疗专业人员提供。如果你或你的伴侣有乙型肝炎，并考虑生育，你们应该寻求医疗专业人员的建议。 | 1,1 | 1,1 |  | 1, 1 |
| 77 | Clinical Problems | NA |  |  |  |  |  |  |
| 77 | Clinical Problems | 乙肝选国产恩替卡韦还是进口恩替卡韦 | 乙肝的治疗药物选择取决于多种因素，包括药物的效果、副作用、价格、以及患者的个人偏好。恩替卡韦（Entecavir）是一种广泛用于乙型肝炎治疗的药物。  对于国产和进口恩替卡韦的选择，以下是一些需要考虑的因素：  1.药品质量：一般来说，进口药品的质量控制可能更严格，但这并不意味着国产药品的质量就一定较差。国产药品也需符合中国的药品生产质量管理规范（GMP）。  2.价格：国产药品通常价格更为亲民。如果长期使用，国产药品可能会更经济。  3.药物效果：恩替卡韦无论是国产还是进口，其主要成分是一样的，理论上应该具有相似的药效。然而，每个人对药物的反应可能会有所不同。  4，医生建议：医生会根据你的病情、身体状况以及其他医疗因素给出最适合你的建议。  因此，建议你在选择国产还是进口恩替卡韦时，与你的医生进行详细的讨论，根据你的具体情况来做决定。 | 在选择恩替卡韦（Entecavir）药品时，重要的是确保药品的质量和有效性，而不仅仅是考虑药品的来源（国产或进口）。以下是一些你应该考虑的因素：  1.质量和有效性：国产和进口的恩替卡韦都需要通过严格的质量和有效性测试。确保你选择的药品是经过相关药品监管机构批准的，例如中国的国家药品监督管理局（NMPA）。  2.价格：通常，国产药品的价格比进口药品更便宜。但是，价格并不能直接决定药品的质量和有效性。选择时应综合考虑药品的质量、有效性和价格。  3.医生建议：你的医生可能会根据你的具体情况（如病情、身体状况、经济状况等）给出最佳的药品选择建议。  4.药品的适应症和副作用：不同的恩替卡韦药品可能会有不同的适应症和副作用。在选择药品时，应仔细阅读药品说明书，或咨询医生或药师。  总的来说，国产恩替卡韦和进口恩替卡韦在质量和效果上应该是类似的，只要它们都通过了相关药品监管机构的批准。你的选择应基于以上因素，特别是你的医生的建议。 | 1,1 | 1,2 |  | 1, 1 |
| 78 | Clinical Problems | Is it possible to return to positive after taking interferon for hepatitis B? | Yes, it is possible for people with chronic hepatitis B to see a positive response after taking Interferon. Interferon is one of the treatments used to manage chronic hepatitis B infection.  Interferon works by boosting the immune system to fight the hepatitis B virus. It can reduce the viral load (the amount of virus in the body), decrease inflammation of the liver, and in some cases, lead to the loss of hepatitis B surface antigen (HBsAg), a marker of active infection.  The ultimate goal of hepatitis B treatment is to achieve a “functional cure,” defined as the loss of HBsAg and the development of anti-HBs antibodies. This doesn’t mean the virus is completely eradicated from the body, but rather it’s controlled to the point where it’s not causing ongoing liver damage and the risk of transmitting the virus is greatly reduced.  However, not everyone responds to Interferon treatment. The response rate can vary widely, depending on factors such as the individual’s immune system, the genotype of the virus, and the stage of the disease.  It’s also important to note that Interferon treatment can have significant side effects, including flu-like symptoms, depression, and low white blood cell counts, which can lead to an increased risk of infections.  Therefore, the decision to use Interferon or any other treatment for chronic hepatitis B should be made in consultation with a healthcare provider, taking into account the potential benefits and risks. Regular monitoring is also necessary to assess the response to treatment and to detect any potential side effects. | Yes, it is possible for individuals with chronic Hepatitis B to become Hepatitis B surface antigen (HBsAg) negative after treatment with interferon or other antiviral medications. However, it’s important to note that this doesn’t necessarily mean that the virus has been completely eradicated from the body.  Chronic Hepatitis B is a long-term infection of the hepatitis B virus. It can cause serious liver damage and is a major risk factor for liver cancer. The goal of treatment is to prevent these complications by suppressing viral replication.  Interferon is one of the treatment options for chronic hepatitis B. It is an immunomodulatory drug, meaning it boosts the body’s immune response to the virus. It can be effective in suppressing the virus and reducing liver inflammation.  However, the rate of patients becoming HBsAg negative after interferon treatment is relatively low. According to some studies, about 3% to 7% of patients become HBsAg negative after one year of interferon treatment. The rate can be higher in certain subgroups of patients.  It’s also important to note that even if a patient becomes HBsAg negative, they may still have the virus in their liver cells in a dormant form. This is known as occult hepatitis B. Therefore, regular monitoring is still necessary.  In conclusion, while it is possible to become HBsAg negative after interferon treatment for chronic hepatitis B, the rates are relatively low, and it does not necessarily mean complete eradication of the virus. The decision to start treatment should be individualized and discussed thoroughly with a healthcare provider, considering the potential benefits and risks.  It’s also essential to remember that the best way to prevent hepatitis B is vaccination, which is safe and highly effective.  Disclaimer: This information is intended to be educational and should not be used as a substitute for professional medical advice. Always consult with a healthcare provider for advice about your health. | 1,1 | 1,1 |  | 1, 1 |
| 78 | Clinical Problems | 乙肝打干扰素转阴后可能复阳吗 | 乙型肝炎（HBV）的患者在接受干扰素治疗后，虽然可能会出现HBV病毒的血清学标志物（如HBeAg和HBsAg）转阴的情况，但这并不能保证永久性的病毒清除。研究显示，即使在治疗结束后病毒标志物转阴，也有可能在一段时间后复阳，即病毒再次活跃。这种情况称为"复发"或"复燃"。  复阳的可能性取决于许多因素，包括患者的免疫状态，病毒的耐药性，治疗方案的适当性，以及患者对治疗的依从性。此外，一些患者可能携带有病毒的肝细胞，这些细胞在某些条件下可能再次开始生产病毒。  总的来说，乙肝患者在接受干扰素治疗后转阴，确实存在复阳的可能性。因此，对于接受治疗的患者来说，定期的医学监测是非常重要的，以便及时发现并处理可能的复发。 | 乙型肝炎是一种由乙型肝炎病毒（HBV）引起的肝炎，可以通过血液、性接触或从母亲传给新生儿来传播。乙型肝炎的治疗目标是防止肝病进展，降低肝硬化和肝癌的风险。干扰素是治疗乙型肝炎的一种常用药物，通过提高机体免疫力来抑制病毒复制。  乙型肝炎患者在接受干扰素治疗后，其血清中的乙型肝炎表面抗原（HBsAg）可能会转阴，即从阳性变为阴性，这通常被认为是疾病治愈的标志。然而，即使HBsAg转阴，也不能保证病毒已被完全清除，因为HBV可以在肝细胞中形成"隐性感染"，即所谓的cccDNA。  因此，乙型肝炎患者在HBsAg转阴后，确实存在复阳（即HBsAg由阴性再次变为阳性）的可能性。这可能是由于病毒在体内的隐性感染，或者是由于患者再次接触到HBV病毒。  复阳的风险因人而异，可能与个体的免疫状态、病毒的类型和数量、是否持续接受抗病毒治疗等因素有关。因此，即使HBsAg转阴，患者也应定期进行血液检查，以便及时发现可能的复阳情况。  总的来说，虽然乙型肝炎在接受干扰素治疗后有可能复阳，但是通过持续的抗病毒治疗和定期的医学监测，可以大大降低这种风险。 | 2,1 | 2,2 | 2,1 | 2,1 |
| 79 | Clinical Problems | NA |  |  |  |  |  | 1, 1 |
| 79 | Clinical Problems | 乙肝的中医治疗 | 在中医中，乙型肝炎（乙肝）被认为是“湿热”、“瘀血”、“肝郁”等病理改变的结果，治疗的目标是调和肝脏，消除湿热，活血化瘀，舒肝解郁。以下是一些常见的中医治疗方法：  1.中药治疗：中药治疗乙肝的方法种类繁多，选择的药物主要取决于患者的病症和体质。常见的药物包括茵陈蒿、大黄、丹皮、柴胡、黄芩、生地黄、牡丹皮、赤芍、郁金等。这些药物有助于清热解毒，疏肝解郁，活血化瘀。  2.针灸治疗：针灸可以通过刺激特定的穴位来调和气血，疏通经络，改善肝脏功能。常用的穴位包括太冲、中脘、足三里、肝俞、脾俞等。  3.拔罐和刮痧：这些疗法可以通过刺激皮肤和肌肉来改善血液循环，消除瘀血，调和气血。  4.食疗和生活方式调整：饮食上应选择清淡、易消化的食物，避免油腻、辛辣、酒精等刺激性食物。同时，保持良好的生活习惯，如适度运动，保持充足的休息，避免过度劳累，都有助于恢复肝脏健康。  值得注意的是，虽然中医治疗可以帮助缓解乙肝的症状和改善肝脏功能，但乙肝是一种病毒性疾病，需要抗病毒治疗。因此，患者在接受中医治疗的同时，也应遵医嘱接受西医的抗病毒治疗。  此外，乙肝的治疗需要长期坚持，患者应定期做肝功能检查和乙肝病毒载量检测，以监测病情的变化。 | 乙型肝炎是一种严重的肝病，中医治疗乙型肝炎有着悠久的历史和丰富的经验。下面是一些常见的中医治疗方法：  1.草药治疗：中医草药是治疗乙型肝炎的主要方法之一。一些常见的草药包括茵陈蒿、柴胡、黄芩、当归、白芍、甘草等。这些草药可以清热解毒，疏肝解郁，益气养血，强壮肝脏。  2.食疗：中医食疗也是治疗乙型肝炎的有效方法之一。一些对肝脏有益的食物，如山楂、枸杞、黄豆、绿茶等，都可以用来治疗乙型肝炎。  3.针灸：针灸是中医的一种重要治疗手段，可以通过刺激特定的穴位来调节身体的气血，从而达到治疗乙型肝炎的效果。  4.推拿和按摩：推拿和按摩可以帮助改善肝脏的血液循环，减轻疲劳，提高免疫力，对乙型肝炎的治疗有一定的帮助。  5.太极和气功：太极和气功是中医的一种身心调节方法，可以帮助调节气血，强健肝脏，对乙型肝炎的治疗有一定的帮助。  以上的治疗方法都需要在专业医生的指导下进行，每个人的身体状况不同，需要个体化的治疗方案。在接受中医治疗的同时，也需要配合西医的治疗，如抗病毒药物等，以达到最佳的治疗效果。  请注意，这些信息仅供参考，如果您或他人可能患有乙型肝炎，请立即就医，并在医生指导下进行治疗。 | 2,2 | 1,2 | 1,1 | 1, 1 |
| 80 | AASLD Guideline questions | Should adults with immune active CHB be treated with antiviral therapy to decrease liver-related complications? | Yes, antiviral therapy is typically recommended for adults with immune active Chronic Hepatitis B (CHB) to decrease liver-related complications. CHB is a significant global health issue that can lead to severe liver diseases such as cirrhosis, liver failure, and hepatocellular carcinoma (HCC).  Immune active CHB is characterized by high levels of hepatitis B virus (HBV) DNA and elevated serum alanine aminotransferase (ALT) levels, indicating ongoing liver inflammation.  Antiviral therapy, such as nucleos(t)ide analogs (NAs) or interferon, can suppress HBV replication and thus halt the progression of liver disease. They have been shown to decrease the incidence of liver cirrhosis and HCC, as well as to improve liver function and survival rates in CHB patients.  However, the decision to initiate antiviral therapy should be individualized, taking into account various factors such as the patient’s age, disease stage, viral load, liver enzyme levels, presence of cirrhosis, and potential for side effects.  Long-term monitoring is also crucial, as treatment does not generally eradicate the virus and there is a risk of relapse or development of drug resistance.  It’s important to note that these recommendations are based on current medical understanding and guidelines, and may change as new research emerges. It’s always best to consult with a healthcare professional for personal medical advice. | Yes, adults with immune active Chronic Hepatitis B (CHB) should generally be treated with antiviral therapy to decrease liver-related complications.  Chronic Hepatitis B is a long-term infection of the hepatitis B virus (HBV) which can lead to serious health issues like cirrhosis, liver failure, or liver cancer. The immune active phase is characterized by high levels of viral replication and liver inflammation, which can lead to progressive liver damage.  Antiviral therapy plays a crucial role in controlling the viral replication, reducing liver inflammation, preventing progression to cirrhosis, and decreasing the risk of hepatocellular carcinoma. The goal of the therapy is to suppress the HBV replication and thereby minimize liver damage.  Common antiviral medications used for the treatment of CHB include nucleos(t)ide analogues like Entecavir, Tenofovir disoproxil fumarate, and Tenofovir alafenamide.  However, the decision to start antiviral therapy should be individualized, taking into account factors such as the patient’s age, overall health status, liver disease stage, HBV DNA levels, and the presence of liver inflammation or fibrosis.  Always, it’s important to consult with a healthcare professional for appropriate management strategies.  Please note, this is general advice, and treatment decisions should always be made in consultation with a healthcare provider. | 1,1 | 1, 1 |  | 1, 1 |
| 80 | AASLD Guideline questions | 免疫活动性CHB成人患者是否应接受抗病毒治疗以减少肝脏相关并发症？ | 免疫活动性慢性乙型肝炎（CHB）是一种重要的全球公共卫生问题，可导致肝硬化和肝癌。对于这种患者，抗病毒治疗已被证明可以有效降低肝硬化和肝癌的风险。  根据世界卫生组织（WHO）和美国肝病学会（AASLD）的指南，以下是一些关于何时开始抗病毒治疗的建议：  1.所有ALT水平持续升高和HBV DNA水平高于2000 IU/mL的HBeAg阳性和阴性成人患者。  2.所有肝炎活动（肝活检组织学评分≥A2或F1）的患者，无论ALT和HBV DNA水平如何。  3.所有肝硬化患者，无论ALT和HBV DNA水平如何。  4.其他具有特殊考虑的患者群体，例如家族中有肝癌病史的患者，或是考虑孕妇以防止母婴传播。  然而，所有患者都应接受定期的肝病监测，包括肝功能、HBV DNA水平和肝硬化标志物。对于已经开始抗病毒治疗的患者，应定期评估病毒学反应、生化反应和临床反应。  在开始抗病毒治疗前，患者应接受详细的咨询，以了解治疗的好处和潜在风险，以及治疗期间的监测和依从性的重要性。  需要注意的是，这些指南可能会随着新的研究和临床试验结果的出现而发生变化。因此，医生应根据最新的证据和患者的具体情况做出最佳决策。 | 根据世界卫生组织（WHO）和各大肝病学会的指南，免疫活动性慢性乙型肝炎（CHB）成人患者应该接受抗病毒治疗以减少肝脏相关并发症，包括肝硬化和肝癌。以下是一些关键的考虑因素：  1.病毒复制水平：血清HBV DNA水平高于2,000 IU/mL的患者可能会从抗病毒治疗中受益。  2.肝炎活动：ALT（丙氨酸氨基转移酶）水平升高或肝活检显示有炎症或纤维化的患者应考虑抗病毒治疗。  3.肝硬化的存在：对于已经有肝硬化的患者，即使HBV DNA水平低于2,000 IU/mL，也应该接受抗病毒治疗，因为这可以降低肝癌和肝衰竭的风险。  4.年龄和性别：年轻的男性患者以及年龄大于30岁的女性患者如果HBV DNA水平高于20,000 IU/mL，ALT水平升高，应考虑抗病毒治疗。  在选择抗病毒治疗方案时，应考虑患者的个人偏好、并发症、耐受性、药物抗性、以及疗效。目前，主要的抗病毒治疗药物包括核苷（酸）类似物（如恩替卡韦、替诺福韦）和干扰素。这些药物可以有效抑制HBV的复制，改善肝炎活动，阻止肝硬化和肝癌的发展。  然而，所有的治疗决策都应在医生的指导下进行，因为每个患者的情况都是独特的，需要根据患者的具体状况制定个性化的治疗方案。 | 2,1 | 1, 1 | 1, 1 | 1, 1 |
| 81 | AASLD Guideline questions | Should adults with immune-tolerant infection be treated with antiviral therapy to decrease liver-related complications? | The decision to treat adults with immune-tolerant chronic hepatitis B infection is a complex one and should be made on a case-by-case basis.  The immune-tolerant phase of chronic hepatitis B is characterized by high levels of hepatitis B virus (HBV) DNA and hepatitis B e antigen (HBeAg) positivity, normal or minimally elevated alanine aminotransferase (ALT) levels, and minimal or no liver inflammation or fibrosis on biopsy. Historically, this phase was thought to be benign and treatment was not recommended due to the low risk of disease progression and the potential for adverse effects from long-term antiviral therapy.  However, more recent studies have suggested that immune-tolerant chronic hepatitis B may not be as benign as previously thought. Some patients may progress to cirrhosis and hepatocellular carcinoma (HCC) despite having normal ALT levels. In addition, it has been suggested that early antiviral therapy may be beneficial in preventing disease progression in these patients.  The decision to initiate antiviral therapy should be individualized and take into account several factors, including:  1.The patient’s age, as the risk of disease progression increases with age.  2.The presence of other risk factors for disease progression, such as male sex, family history of HCC, alcohol use, coinfection with other viruses (e.g., hepatitis C, HIV), and metabolic syndrome.  3.The potential risks and benefits of antiviral therapy, including the risk of drug resistance and side effects from long-term therapy.  Further research is needed to determine the optimal management strategy for adults with immune-tolerant chronic hepatitis B. Current guidelines from professional organizations such as the American Association for the Study of Liver Diseases (AASLD) and the European Association for the Study of the Liver (EASL) do not recommend routine antiviral therapy in the immune-tolerant phase, but they do suggest that it may be considered in certain high-risk patients.  For these reasons, it is crucial to have a thorough discussion with a healthcare provider who can provide personalized advice based on the patient’s individual circumstances. | While I’m an AI and can’t provide personal medical advice, I can provide some general information about this topic based on current scientific knowledge.  Immune-tolerant infection typically refers to a phase of chronic hepatitis B infection. This phase is characterized by high levels of HBV DNA and normal alanine aminotransferase (ALT) levels, with minimal or no inflammation or fibrosis on liver biopsy. The immune system is not actively fighting the virus, hence the term “immune-tolerant.”  The decision to treat adults with immune-tolerant hepatitis B infection with antiviral therapy is often complex and depends on various factors, including patient age, presence of family history of hepatocellular carcinoma (HCC), and patient preference.  As per the current guidelines by the American Association for the Study of Liver Diseases (AASLD) and the European Association for the Study of the Liver (EASL), antiviral therapy is generally not recommended for adults in the immune-tolerant phase of chronic hepatitis B, due to the low risk of disease progression and the potential for long-term side effects from the medication.  However, some studies suggest that early antiviral therapy could potentially decrease the risk of liver-related complications such as cirrhosis and HCC. This is an area of ongoing research.  Therefore, the decision to start antiviral therapy in immune-tolerant adults should be individualized, taking into account the potential benefits and risks. Regular monitoring of ALT and HBV DNA levels, as well as liver imaging, are essential to detect any changes in disease status.  It’s important for anyone with chronic hepatitis B, including those in the immune-tolerant phase, to discuss their treatment options with a healthcare provider who is knowledgeable about the disease.  Please note that this information might change as new research findings emerge. Always consult with a healthcare professional for the most accurate information. | 1,1 | 1, 1 |  | 1, 1 |
| 81 | AASLD Guideline questions | 免疫耐受感染的成人是否应接受抗病毒治疗以减少肝脏相关并发症？ | 免疫耐受性感染，特别是对于慢性乙型肝炎（CHB）的患者，是一个复杂的问题。免疫耐受期是指血清中乙型肝炎病毒（HBV）DNA水平高，血清中的乙型肝炎表面抗原（HBsAg）阳性，但是丙氨酸氨基转移酶（ALT）水平正常，肝活检显示炎症程度轻的状态。  对于是否应接受抗病毒治疗以减少肝脏相关并发症，这主要取决于患者的具体情况。一般来说，如果患者的病毒载量持续高，ALT水平持续正常或升高，并且/或者肝活检显示有中度至重度炎症或者纤维化，那么可能需要考虑抗病毒治疗。但是，如果患者在免疫耐受期并且没有明显的肝脏疾病症状或者病理改变，那么通常不推荐抗病毒治疗，因为这可能带来不必要的副作用和费用。  在做出决定时，医生会考虑许多因素，包括患者的年龄、性别、病毒载量、ALT水平、肝病历史、家族病史、合并症以及患者的生活质量等。因此，对于是否接受抗病毒治疗，应由医生和患者共同决定。  需要注意的是，这些建议可能会随着新的研究结果和治疗策略的发展而变化。因此，患者应该定期和医生进行讨论，以便获取最新的信息和建议。  这些信息仅供参考，并不能替代专业医疗建议。如果你有关于这个问题的疑虑，你应该咨询你的医生或者其他合格的医疗专业人士。  参考文献：  1.Terrault NA, Lok ASF, McMahon BJ, et al. Update on prevention, diagnosis, and treatment of chronic hepatitis B: AASLD 2018 hepatitis B guidance. Hepatology. 2018;67(4):1560-1599. doi:10.1002/hep.29800  2.European Association for the Study of the Liver. EASL 2017 Clinical Practice Guidelines on the management of hepatitis B virus infection. J Hepatol. 2017;67(2):370-398. doi:10.1016/j.jhep.2017.03.021  3.Sarin SK, Kumar M, Lau GK, et al. Asian-Pacific clinical practice guidelines on the management of hepatitis B: a 2015 update. Hepatol Int. 2016;10(1):1-98. doi:10.1007/s12072-015-9675-4 | 免疫耐受感染是指患者体内存在某种病原体，但是他们的免疫系统并未对此产生反应，也就没有明显的病症。对于这种情况，是否需要进行抗病毒治疗以减少肝脏相关并发症，是一个需要考虑多个因素的决定。  首先，需要明确病原体的类型。例如，对于慢性乙型肝炎病毒（HBV）感染，如果患者处于免疫耐受阶段（通常表现为血清中HBV DNA水平高，但ALT水平正常，肝脏炎症程度轻或无），一般不推荐抗病毒治疗。这是因为在这个阶段，抗病毒治疗的益处可能不会超过其可能的风险，如药物副作用和耐药性的发展。  然而，每个患者的情况都是独特的，所以应由医疗专业人员进行详细评估。他们会考虑患者的年龄、病毒载量、肝脏疾病的严重程度、合并症、以及患者的整体健康状况等因素。  此外，需要定期进行监测，以便在患者的免疫状态发生变化时，及时调整治疗策略。例如，如果HBV DNA水平持续高昂，ALT水平开始升高，或者肝脏病变进展，可能需要开始抗病毒治疗。  因此，免疫耐受感染的成人是否应接受抗病毒治疗以减少肝脏相关并发症，需要根据他们的具体情况和医生的专业建议来决定。 | 2,2 | 2,2 |  | 2,2 |
| 82 | AASLD Guideline questions | Should antiviral therapy be discontinued in hepatitis B e antigen (HBeAg)-positive persons who have developed HBeAg seroconversion on therapy? | The decision to discontinue antiviral therapy in hepatitis B e antigen (HBeAg)-positive individuals who have achieved HBeAg seroconversion is a complex one and must be made on an individual basis, considering several factors.  HBeAg seroconversion, which is the loss of HBeAg and the development of antibodies against HBeAg (anti-HBe), is a favorable prognostic sign in chronic hepatitis B infection. It is usually associated with a decrease in viral replication and a reduction in liver inflammation. However, it does not necessarily mean that the virus has been completely eradicated from the body. Thus, the risk of relapse (reappearance of HBeAg or increase in HBV DNA) after stopping treatment is a major concern.  Several guidelines have been developed to assist clinicians in making this decision. For instance, the American Association for the Study of Liver Diseases (AASLD) guidelines suggest that antiviral therapy can be discontinued in HBeAg-positive individuals who have achieved HBeAg seroconversion, provided that they have completed at least 12 months of consolidation therapy after seroconversion and that their HBV DNA levels remain undetectable. However, these patients should be closely monitored for relapse.  It’s also important to note that other factors may influence the decision to discontinue therapy, such as the patient’s age, presence of liver cirrhosis, family history of hepatocellular carcinoma, and the type of antiviral medication used.  In conclusion, while HBeAg seroconversion is a positive sign, the decision to discontinue antiviral therapy in HBeAg-positive individuals should be made carefully, considering the risk of relapse and other individual factors. Regular monitoring is essential for those who discontinue therapy.  Please consult with a healthcare provider for advice tailored to the individual’s circumstances. | Hepatitis B e antigen (HBeAg) seroconversion, which involves the loss of HBeAg and the appearance of anti-HBe antibodies, is often used as a benchmark to determine the efficacy of antiviral therapy in patients with chronic hepatitis B infection. However, the decision to discontinue antiviral therapy after HBeAg seroconversion is a complex one and should be made by a healthcare professional based on individual patient factors.  It’s important to note that HBeAg seroconversion is generally a positive sign indicating lower levels of viral replication and a more favorable prognosis. Nevertheless, this doesn’t necessarily mean that the virus has been completely eradicated from the body. Reactivation of the virus and disease progression can still occur, particularly in those with ongoing liver damage.  Some guidelines suggest that antiviral therapy can be stopped after HBeAg seroconversion, provided that the patient has completed a sufficient duration of consolidation therapy (usually at least 6-12 months) after seroconversion, and that there’s evidence of sustained suppression of hepatitis B virus (HBV) DNA. However, these patients should be closely monitored for relapse, indicated by rising HBV DNA levels, reappearance of HBeAg, or increase in liver enzymes.  Patients with cirrhosis, even if they have undergone HBeAg seroconversion, are generally recommended to continue antiviral therapy indefinitely due to the risk of hepatic decompensation and hepatocellular carcinoma.  In conclusion, while HBeAg seroconversion is a positive outcome, the decision to discontinue antiviral therapy should be individualized based on the patient’s overall health, liver function, and risk of disease progression. Regular monitoring is crucial in all cases.  Always consult a healthcare professional for personalized medical advice. | 1,1 | 1, 1 |  | 1, 1 |
| 82 | AASLD Guideline questions | 在治疗中发生HBeAg血清学转换的乙型肝炎e抗原(HBeAg)阳性患者是否应停止抗病毒治疗？ | 乙型肝炎e抗原(HBeAg)阳性患者在抗病毒治疗中出现HBeAg血清学转换（即从HBeAg阳性转为HBeAg阴性，并且乙型肝炎e抗体（anti-HBe）阳性）是治疗的一个重要目标。这通常表明病毒复制的降低和肝炎活动的减轻。  然而，即使出现HBeAg血清学转换，也不一定意味着应该立即停止抗病毒治疗。研究显示，即使在HBeAg血清学转换后，一部分患者的病毒仍可能反弹，导致病情恶化。因此，是否停止治疗需要考虑多种因素，如患者的年龄、基础疾病、肝病损伤程度、病毒载量、抗病毒治疗的种类等。  在很多指南中，建议在HBeAg血清学转换后继续使用核苷（酸）类似物（NAs）治疗至少6-12个月，以降低病毒反弹的风险。如果使用的是干扰素，那么在HBeAg血清学转换后可以考虑停止治疗。  在决定是否停止抗病毒治疗时，患者应与医生进行详细的讨论，以达成最佳的个体化决策。 | 乙型肝炎e抗原（HBeAg）血清学转换是乙型肝炎治疗的一个重要目标，它通常意味着病毒复制的控制和肝炎活动的减少。然而，对于是否应在HBeAg血清学转换后停止抗病毒治疗，需要根据多个因素来决定，包括患者的年龄、疾病阶段、抗病毒治疗的类型和持续时间等。  1.一般来说，如果患者接受的是核苷（酸）类似物（Nucleos(t)ide analogues，NAs）治疗，那么在HBeAg血清学转换后，大多数指南建议继续治疗至少12个月，以减少病毒反弹和HBeAg再阳性的风险。这段时间也被称为"保护期"。  2.对于接受干扰素治疗的患者，他们可以在HBeAg血清学转换后立即停止治疗，因为干扰素有持久的抗病毒效果和较低的病毒反弹风险。  3.在任何情况下，停止抗病毒治疗都需要定期监测肝功能和HBV DNA，以便及时发现和处理可能的病毒反弹。  这些是一般的建议，具体的决定应根据患者的个体情况和医生的专业判断来做。如果你或你的亲友有这个问题，应该咨询医生以获取最适合的建议。 | 2,2 | 1, 1 | 1, 1 | 1, 1 |
| 83 | AASLD Guideline questions | Should antiviral therapy be discontinued in persons with HBeAg-negative infection with sustained HBV DNA suppression on therapy? | The decision to discontinue antiviral therapy in persons with HBeAg-negative Hepatitis B virus (HBV) infection with sustained HBV DNA suppression on therapy is complex and should be individualized based on several factors.  Hepatitis B e-antigen (HBeAg) negative chronic hepatitis B (CHB) is often associated with a higher risk of liver disease progression, including cirrhosis and hepatocellular carcinoma. Therefore, it’s crucial to maintain viral suppression to prevent these complications. Antiviral therapy is typically continued indefinitely in HBeAg-negative patients until specific endpoints are met, such as HBsAg loss or seroconversion.  However, long-term antiviral therapy can also have potential drawbacks such as medication side effects, cost, and the risk of developing drug-resistant HBV strains.  Therefore, the decision to discontinue therapy should consider:  1.The patient’s age and overall health  2.The presence of liver cirrhosis or hepatocellular carcinoma  3.The patient’s ability to adhere to medication regimens  4.The patient’s HBV DNA levels and alanine aminotransferase (ALT) levels  5.The risk of HBV reactivation upon discontinuation of therapy  Any decision to stop antiviral therapy should be made in consultation with a healthcare provider experienced in managing hepatitis B. Frequent monitoring of liver function and HBV DNA levels is required after stopping therapy to detect any potential viral rebound.  Please note, this advice is based on the current understanding and guidelines and may change as more research is conducted in this field. Always consult a healthcare provider for personalized medical advice. | The decision to discontinue antiviral therapy in individuals with HBeAg-negative hepatitis B virus (HBV) infection who have achieved sustained HBV DNA suppression on therapy should be made on an individual basis, considering various factors such as the patient’s overall health, liver disease stage, potential for drug resistance, and risk of disease reactivation.  Some research suggests that long-term suppression of HBV replication can lead to regression of liver fibrosis and cirrhosis, and a reduction in the risk of hepatocellular carcinoma. Hence, continuous antiviral therapy might be beneficial for some patients.  However, prolonged therapy has its own risks, including potential side effects, drug resistance, and high cost. Additionally, sustained virologic response (SVR) does not guarantee complete eradication of the virus due to the persistence of covalently closed circular DNA (cccDNA) in the liver cells.  Therefore, the decision to discontinue antiviral therapy must be carefully weighed and should involve a thorough discussion between the patient and their healthcare provider. Monitoring for disease reactivation should be conducted if therapy is discontinued. Guidelines by some liver societies also recommend that antiviral therapy should not be stopped until HBsAg loss is achieved, which is the best indicator of functional cure in chronic hepatitis B.  Please note: This information is based on current medical knowledge and guidelines, and it’s crucial to consult with healthcare professionals for individual treatment decisions. The state of knowledge may evolve over time, and newer treatments or guidelines may be available.  Sources:  1.Terrault N, et al. AASLD guidelines for treatment of chronic hepatitis B. Hepatology, 2016.  2.European Association for the Study of the Liver. EASL 2017 Clinical Practice Guidelines on the management of hepatitis B virus infection. Journal of Hepatology, 2017.  3.Sarin SK, et al. Asian-Pacific clinical practice guidelines on the management of hepatitis B: a 2015 update. Hepatology International, 2016. | 2,2 | 1,2 | 2,2 | 2,2 |
| 83 | AASLD Guideline questions | 对于治疗后HBV DNA持续抑制的HBeAg阴性感染者，是否应停止抗病毒治疗？ | 对于HBeAg阴性的患者，他们的病毒复制活动可能会在没有任何警告的情况下反弹，因此他们通常需要长期或者持续的抗病毒治疗。  然而，关于何时停止抗病毒治疗，还是存在一些争议。一些专家认为，只要患者的HBV DNA持续抑制，ALT水平正常，可以考虑停止治疗。但是，这种做法可能会增加病毒反弹和肝炎复发的风险。  因此，是否应该停止抗病毒治疗需要根据患者的具体情况来判断，包括他们的病毒负荷，肝功能，以及他们的健康状况等。在任何情况下，这种决定都应由医生和患者共同做出。  需要注意的是，停止抗病毒治疗后，患者需要定期进行监测，以便及时发现并处理可能出现的病毒复活或肝炎复发。  总的来说，对于治疗后HBV DNA持续抑制的HBeAg阴性感染者，是否应停止抗病毒治疗，需要根据患者的具体情况和医生的建议来决定。 | 对于慢性乙型肝炎（HBV）的治疗，是否应停止抗病毒治疗这个问题，一直是临床决策的难题。这主要取决于患者的病情、抗病毒治疗的类型和持续时间、HBV DNA水平、肝功能等多个因素。  对于HBeAg阴性的HBV感染者，如果经过长期抗病毒治疗后，HBV DNA持续抑制，那么是否停止治疗需要综合考虑以下因素：  1.患者的年龄、性别和基础疾病状况。  2.治疗期间的HBV DNA水平变化和持续时间。  3.患者的肝功能，包括ALT（丙氨酸氨基转移酶）水平。  4.患者是否存在肝硬化或肝癌等并发症。  5.患者的依从性和可能的药物副作用。  在一些情况下，医生可能会建议停止抗病毒治疗，但这需要在严密的监测下进行，包括定期检查HBV DNA水平和肝功能，以及定期进行肝脏影像学检查。但是，即使在抗病毒治疗停止后，也可能出现HBV复发或肝炎活动加重的情况，因此需要密切监测。  请注意，这个问题的答案可能因地区和具体的临床实践而有所不同，所以建议您咨询医生或医疗专业人士以获取最准确和适合您个人情况的建议。  以上信息仅供参考，不应替代医生的专业医疗建议。 | 2,1 | 2,1 |  | 2,1 |
| 84 | AASLD Guideline questions | In HBV-monoinfected persons, does entecavir therapy, when compared to tenofovir therapy, have a different impact on renal and bone health? | As an AI model developed by OpenAI, I provide information based on the data I was trained on, up to my last training cut-off in 2021. As of that time, both entecavir and tenofovir are antiviral medications used to treat chronic hepatitis B virus (HBV) infection. The choice between the two may depend on various factors, including patient’s renal function, bone health, and the risk of drug resistance.  Tenofovir disoproxil fumarate (TDF), one form of tenofovir, has been associated with renal and bone toxicity. Renal toxicity may manifest as a decrease in estimated glomerular filtration rate (eGFR), and bone toxicity may result in a decrease in bone mineral density (BMD). This is due to the drug’s impact on proximal renal tubular function and its potential to cause renal tubular toxicity.  Entecavir, on the other hand, has not been shown to be associated with significant renal or bone toxicity. It is generally considered safer than TDF in terms of renal and bone health.  However, a newer form of tenofovir, known as tenofovir alafenamide (TAF), has been developed to lower the risk of renal and bone toxicity while maintaining strong antiviral efficacy. TAF provides a similar level of viral suppression as TDF but with less impact on the kidneys and bones.  That being said, the decision to use entecavir or tenofovir should be individualized, taking into consideration the patient’s overall health status, potential for drug resistance, and other factors.  Please consult with a healthcare provider for the most recent and relevant information as my knowledge is up to 2021 and does not include any new studies or findings that may have emerged after this time. | I’m an AI language model and can provide information based on available data up to the year 2021. As of that time, both entecavir (ETV) and tenofovir (TDF) are considered first-line treatments for chronic hepatitis B virus (HBV) infection.  In terms of renal and bone health:  1.Renal Health: Tenofovir has been associated with renal impairment, including acute kidney injury and Fanconi syndrome. This risk appears to be greater in individuals with pre-existing renal disease or other risk factors for renal dysfunction. On the other hand, entecavir has not been significantly associated with renal dysfunction.  2.Bone Health: Tenofovir has been linked with a decrease in bone mineral density (BMD), which can lead to osteoporosis and increased fracture risk over time. This risk appears to be higher in patients with risk factors for bone loss. As for entecavir, it has not been significantly associated with bone loss.  Therefore, while both drugs are effective against HBV, entecavir might have a more favorable safety profile regarding renal and bone health. However, it’s important to note that individual patient characteristics and comorbidities should always guide the choice of antiviral therapy.  Please consult with a healthcare provider for the most recent and personalized advice. This information might have changed after 2021, and newer drugs might have become available. | 1,1 | 1, 1 |  | 1, 1 |
| 84 | AASLD Guideline questions | 在HBV单一感染者中，恩替卡韦治疗与替诺福韦治疗对肾脏和骨骼健康的影响是否不同？ | 恩替卡韦（Entecavir）和替诺福韦（Tenofovir）都是抗病毒药物，主要用于治疗乙型肝炎病毒（HBV）感染。这两种药物都已被证明在抑制HBV复制，改善肝炎相关症状，甚至预防肝硬化和肝癌方面都非常有效。  然而，关于这两种药物对肾脏和骨骼健康的影响，有一些研究显示它们可能有所不同。  肾脏健康：  替诺福韦已知可能导致肾功能损害。一些研究表明，长期使用替诺福韦可能会导致肾小管功能障碍和肾功能下降。相比之下，恩替卡韦对肾脏的影响通常被认为较小，其引起的肾功能损害的风险相对较低。  骨骼健康：  替诺福韦也可能对骨骼健康产生影响。有研究报告表明，替诺福韦可能会导致骨矿物质密度下降，增加骨折的风险。而恩替卡韦对骨骼健康的影响相对较小，其导致骨密度下降的风险较低。  总的来说，虽然恩替卡韦和替诺福韦在治疗HBV方面都非常有效，但是对于某些患者（如有肾脏疾病或骨质疏松症风险的患者）来说，可能需要更仔细地权衡这两种药物的效果和副作用。在开始任何药物治疗前，都应该与医生详细讨论可能的风险和好处。  请注意，这些信息都是基于现有的科学研究。随着更多研究的进行，我们对这些药物的理解可能会有所改变。 | 恩替卡韦（Entecavir，ETV）和替诺福韦（Tenofovir，TDF）都是用于治疗乙型肝炎病毒（HBV）感染的抗病毒药物。这两种药物都已经显示出非常强大的抗HBV活性，但是，它们对肾脏和骨骼健康的影响可能会有所不同。  一般来说，大部分的研究显示，ETV的肾毒性较低，且对骨骼健康的影响也较小。而TDF虽然是一种非常有效的抗HBV药物，但是已知可能对肾脏功能产生一定的负面影响，并可能增加骨密度下降的风险。  但是，这些影响的程度和临床意义会因人而异，取决于患者的年龄、基线肾功能、并发症和其他可能影响肾脏和骨骼健康的因素。因此，选择这两种药物中的哪一种作为治疗方案，需要考虑到患者的整体健康状况，以及潜在的风险和收益。  最后，虽然我们已经知道TDF可能对肾脏和骨骼健康产生一定的影响，但是新一代的替诺福韦盐酸盐（Tenofovir alafenamide，TAF）已经显示出在保持抗HBV活性的同时，对肾脏和骨骼健康的影响较小。  在选择治疗方案时，应该咨询医生，以便了解最新的研究结果和个人的风险因素。 | 1,1 | 1, 1 |  | 1, 1 |
| 85 | AASLD Guideline questions | Is there a benefit to adding a second antiviral agent in persons with persistent low levels of viremia while being treated with either tenofovir or entecavir? | Adding a second antiviral agent in persons with persistent low levels of viremia while being treated with either tenofovir or entecavir may be beneficial in certain scenarios. It’s important to note that the decision to add another antiviral should be individualized and based on a number of factors, including the patient’s overall health, the presence of other illnesses, their adherence to medication, and the potential for drug resistance.  The main potential benefits include:  1.Improved viral suppression: The addition of a second antiviral agent may help to further reduce viral load in patients who have persistent viremia despite being on treatment. This could potentially lead to better overall outcomes.  2.Prevention of drug resistance: If a patient’s virus has developed resistance to one antiviral, adding a second agent with a different mechanism of action can help to prevent further resistance and maintain the effectiveness of the treatment regimen.  However, there are also potential downsides to consider:  1.Increased side effects: Each antiviral medication has its own set of potential side effects. Adding a second agent could increase the risk of these side effects, which could negatively impact the patient’s quality of life.  2.Increased cost: Antiviral medications can be expensive, and adding a second agent could increase the financial burden on the patient.  3.Complexity of treatment regimen: Adding a second medication could make the treatment regimen more complex, which could potentially impact patient adherence.  It’s also worth noting that tenofovir and entecavir are both potent antivirals with a high genetic barrier to resistance. Therefore, persistent viremia in patients on these medications could potentially be due to issues with adherence or absorption, rather than resistance.  In conclusion, while adding a second antiviral agent could potentially offer benefits in certain situations, it’s a decision that should be made on a case-by-case basis, taking into account the potential benefits and risks, and the individual patient’s circumstances. Always consult with a healthcare provider for personalized medical advice. | While I don’t have real-time data or the ability to access the latest scientific literature, I can provide a general perspective based on my training data up until September 2021.  Tenofovir and Entecavir are both first-line antiviral agents used in the treatment of chronic hepatitis B infection. They have high potency and a high barrier to resistance, which means that they are very effective at reducing viral load and less likely to lead to the development of drug-resistant viruses.  Persistent low levels of viremia (presence of virus in the blood) while being treated with either Tenofovir or Entecavir may indicate suboptimal response to treatment, development of resistance, or poor adherence to medication.  As of my last training data, there was no definitive evidence to support the routine use of combination antiviral therapy in patients with persistent low-level viremia. The decision to add a second antiviral agent should be individualized, taking into account the patient’s overall health, the likelihood of adherence to medication, the potential for drug-drug interactions, and the potential for side effects.  However, there were some ongoing studies investigating the potential benefits of combination therapy in certain patient populations. It’s important to consult with a healthcare provider or specialist to discuss the potential benefits and risks in the context of the latest scientific evidence.  For the most current and accurate information, please refer to the most recent guidelines from reputable health organizations or consult with a healthcare provider. | 2,2 | 1,2 | 2,2 | 2,2 |
| 85 | AASLD Guideline questions | 在接受替诺福韦或恩替卡韦治疗的持续低水平病毒血症患者中，加用第二种抗病毒药物是否有益？ | 对于接受替诺福韦或恩替卡韦治疗的持续低水平病毒血症患者，是否加用第二种抗病毒药物有益，这取决于多种因素，包括但不限于患者的健康状况、病毒的类型和阶段、以及已使用的药物的效果。目前，对于这个问题并没有一致的答案，因为这还需要通过临床试验来验证。  一些研究表明，对于一些患者来说，加用第二种抗病毒药物可能有益。这可能是因为不同的药物可能对不同的病毒生命周期阶段有影响，或者对于某些病毒株有特定的效果。然而，这并不是所有情况。  另一方面，加用第二种抗病毒药物也可能会带来更多的副作用和药物相互作用。因此，医生会根据患者的具体情况来决定是否加用第二种抗病毒药物。  总的来说，对于这个问题，需要根据患者的具体情况和医生的建议来决定。如果您或者您认识的人正在接受这种治疗，并且正在考虑加用第二种抗病毒药物，强烈建议您咨询医生。 | 在慢性乙型肝炎（CHB）的治疗中，替诺福韦（TDF）和恩替卡韦（ETV）是两种常用的核苷（酸）类似物（NAs）。它们能有效抑制乙型肝炎病毒（HBV）的复制，但也有患者在使用这些药物后仍然出现持续的低水平病毒血症。对于这种情况，是否需要加用第二种抗病毒药物，目前的科学研究给出的答案并不一致。  一些研究认为，对于TDF或ETV治疗后仍然存在持续低水平病毒血症的患者，加用第二种抗病毒药物可能有益。这种策略可能能进一步降低HBV DNA的水平，从而降低肝硬化和肝癌的风险。然而，这种策略可能会增加药物的副作用和耐药性的风险，因此需要在临床上谨慎使用。  另一些研究则认为，对于这些患者，加用第二种抗病毒药物可能并无明显益处。这是因为，尽管这种策略可能能进一步降低HBV DNA的水平，但其对患者的长期临床结局（如生存率、肝硬化和肝癌的风险）可能无显著影响。此外，这种策略可能会增加药物的副作用和耐药性的风险。  因此，对于TDF或ETV治疗后仍然存在持续低水平病毒血症的患者，是否加用第二种抗病毒药物，需要根据患者的具体情况（如HBV DNA的水平、肝炎的活动程度、肝功能的状态、药物的耐受性等）和医生的专业判断来决定。  在制定治疗方案时，医生通常会考虑多种因素，包括病人的年龄、性别、病史、合并症、生活方式、个人偏好等。因此，如果你或你的亲友正面临这样的问题，建议咨询专业医生的意见。  请注意，以上内容仅供参考，不应被视为医学建议。如果你有任何健康问题，应立即咨询医生或其他合格的医疗服务提供者。 | 2,2 | 2,1 | 2,1 | 2,1 |
| 86 | AASLD Guideline questions | Should persons with compensated cirrhosis and low levels of viremia be treated with antiviral agents? | The management of chronic viral hepatitis, particularly in patients with compensated cirrhosis and low levels of viremia, is a complex issue and depends on a variety of factors. These include the specific virus involved, the patient’s overall health, the presence of other co-morbidities, and the potential for adverse effects from antiviral therapy.  For Hepatitis B, the American Association for the Study of Liver Diseases (AASLD) recommends antiviral therapy for all patients with compensated cirrhosis and detectable HBV DNA, regardless of the level of viremia. This is due to the risk of disease progression and the development of hepatocellular carcinoma.  For Hepatitis C, with the advent of direct-acting antivirals (DAAs), the level of viremia is no longer a primary determinant for treatment. The AASLD and Infectious Diseases Society of America (IDSA) recommend treating all patients with chronic Hepatitis C infection, including those with compensated cirrhosis, due to the high cure rates and the potential to prevent disease progression.  However, individual patient factors must always be taken into consideration and decisions should be made in consultation with a healthcare provider experienced in the management of viral hepatitis.  Please note that this information is accurate as of the current date and guidelines may change as more research becomes available.  Disclaimer: This information is intended for educational purposes only and should not be used as a substitute for professional medical advice. Always consult with a healthcare provider for advice about your health. | The decision to treat persons with compensated cirrhosis and low levels of viremia with antiviral agents depends on a variety of factors and should always be individualized. It should be made in consultation with the patient and their healthcare provider, considering the potential benefits and risks of treatment.  Here are a few factors to consider:  1.The type of virus causing the viremia: For instance, Hepatitis B and C viruses can cause cirrhosis, and there are effective antiviral therapies for both. Antiviral therapy can slow disease progression and reduce the risk of complications in these cases.  2.The patient’s overall health status: If the patient has significant comorbidities that might be worsened by antiviral therapy, or if their life expectancy is limited due to other factors, the risks of therapy may outweigh the benefits.  3.The stage of cirrhosis: In compensated cirrhosis, the liver is still able to perform its essential functions, despite the damage. The decision to initiate antiviral therapy might depend on the degree of liver function remaining and the risk of progression to decompensated cirrhosis.  4.Potential for disease transmission: Antiviral therapy can reduce the viral load and potentially decrease the risk of transmitting the virus to others.  5.Patient preferences and quality of life considerations: Some patients may choose to take antiviral therapy to reduce the risk of future health problems, even if their current symptoms are mild or nonexistent.  6.Risk of hepatocellular carcinoma (HCC): Patients with cirrhosis are at an increased risk of developing HCC. Antiviral therapy can potentially reduce this risk.  In general, the American Association for the Study of Liver Diseases (AASLD) recommends antiviral therapy for all patients with Hepatitis B virus (HBV) and cirrhosis, regardless of their level of viremia, because of the risk of disease progression and development of HCC.  For Hepatitis C virus (HCV), the decision to treat should be individualized, considering the patient’s comorbidities and life expectancy. However, the advent of direct-acting antiviral (DAA) therapy has made treatment of HCV easier and more effective, and it is generally recommended for all patients with HCV and cirrhosis.  In summary, while low levels of viremia might suggest a less aggressive disease course, the presence of cirrhosis indicates significant liver disease, and antiviral therapy should be strongly considered. However, the decision should be individualized based on the factors discussed above. Always consult with a healthcare provider for personalized advice. | 1,1 | 1, 1 |  | 1, 1 |
| 86 | AASLD Guideline questions | 有低病毒血症的代偿期肝硬化患者是否应接受抗病毒药物治疗？ | 肝硬化是由于长期慢性肝病导致的肝脏结构和功能的严重改变。肝硬化患者如果合并有病毒感染，如乙肝、丙肝等，虽然病毒载量低，但仍有可能加重肝脏损伤，加快疾病进展。  对于低病毒血症的代偿期肝硬化患者，根据当前的医学指南，接受抗病毒药物治疗是推荐的。研究表明，抗病毒治疗可以改善肝功能，减少并发症的发生，延长患者的生存期，降低肝癌的风险。  然而，抗病毒治疗的决策应基于多种因素，包括病人的年龄、健康状况、病毒类型和病毒载量等。因此，具体的治疗方案应由医生根据病人的具体情况来决定。  总的来说，虽然病毒载量低，但代偿期肝硬化患者仍建议接受抗病毒药物治疗，以减少并发症的风险和改善生存质量。  请注意，这些信息仅供参考，具体的医疗决策应由医生根据患者的具体情况来制定。 | 肝硬化是一种慢性疾病，由于肝脏长期受到病毒、酒精、药物或其他因素的损害，导致肝脏细胞大量死亡，被纤维组织所取代，从而导致肝脏结构和功能的严重损害。在许多情况下，肝硬化是由乙型或丙型肝炎病毒引起的。  对于低病毒血症的代偿期肝硬化患者，是否应接受抗病毒药物治疗，这取决于多种因素，包括：  1.病毒类型和活动度：如果病毒仍然活跃，即使病毒载量较低，也可能需要抗病毒治疗。  2.病人的整体健康状况：如果患者身体状况较差，可能无法承受抗病毒药物的副作用。  3.肝脏损伤的程度：如果肝脏已经严重损伤，抗病毒治疗可能无法改变病情进程。  这些决定都需要在医生的指导下进行。医生会根据患者的具体病情和身体状况，以及抗病毒药物的效果和副作用，来决定是否使用抗病毒治疗。  在一些情况下，即使病毒载量较低，抗病毒治疗也可能有益，因为它可以帮助阻止病毒的复制和传播，降低病毒对肝脏的损伤，延缓肝硬化的进程。  总的来说，对于低病毒血症的代偿期肝硬化患者，是否应接受抗病毒药物治疗，需要根据患者的具体情况和医生的建议来决定。 | 2,1 | 1, 1 | 1, 1 | 1, 1 |
| 87 | AASLD Guideline questions | Should pregnant women who are hepatitis B surface antigen (HBsAg) positive with high viral load receive antiviral treatment in the third trimester to prevent perinatal transmission of HBV? | Yes, antiviral treatment in the third trimester is recommended for pregnant women who are hepatitis B surface antigen (HBsAg) positive with high viral load to prevent perinatal transmission of hepatitis B virus (HBV).  Hepatitis B can be transmitted from an infected mother to her child during birth, a process known as vertical or perinatal transmission. This transmission route is particularly common in regions where HBV is highly endemic.  High maternal HBV DNA levels (>200,000 IU/mL) are associated with an increased risk of perinatal transmission, even after appropriate immunoprophylaxis with hepatitis B vaccine and hepatitis B immune globulin (HBIG).  Therefore, current guidelines recommend antiviral therapy in the third trimester (usually starting at 28 to 32 weeks of gestation) for HBsAg-positive pregnant women with high viral load to further reduce the risk of perinatal transmission. The antiviral drugs commonly used include tenofovir and lamivudine, which have been shown to be safe and effective in this context.  However, the decision to start antiviral therapy should be individualized, taking into account factors such as the mother’s liver disease status, potential side effects of the medication, and the risk of developing antiviral resistance.  It’s crucial that any pregnant woman with HBV infection should be under the care of a healthcare provider experienced in managing hepatitis B.  References:  1.Pan CQ, Duan Z, Dai E, et al. Tenofovir to Prevent Hepatitis B Transmission in Mothers with High Viral Load. N Engl J Med. 2016;374(24):2324-2334.  2.WHO Guidelines for the Prevention, Care and Treatment of Persons with Chronic Hepatitis B Infection. Geneva: World Health Organization; 2015.  3.EASL 2017 Clinical Practice Guidelines on the management of hepatitis B virus infection. J Hepatol. 2017;67(2):370-398. | Yes, it is generally recommended that pregnant women who are hepatitis B surface antigen (HBsAg) positive with high viral load receive antiviral treatment in the third trimester to prevent perinatal transmission of hepatitis B virus (HBV). This recommendation is based on several clinical studies demonstrating the efficacy of this approach.  Perinatal transmission of HBV is a significant route of HBV infection worldwide. Without intervention, infants born to HBsAg-positive mothers have a 70% to 90% chance of perinatal HBV transmission if the mother is also hepatitis B e antigen (HBeAg) positive, and a 10% to 40% chance if the mother is HBeAg negative.  Antiviral therapy (with medications such as tenofovir or lamivudine) initiated in the third trimester (from 28 weeks gestation) in HBsAg-positive pregnant women with high viral loads can significantly reduce perinatal HBV transmission. This is particularly important for mothers with high HBV DNA levels (>200,000 IU/mL), where the risk of transmission remains high despite immunoprophylaxis with hepatitis B vaccine and hepatitis B immunoglobulin in the newborn.  However, the decision to start antiviral therapy should be individualized and made in consultation with the patient, considering the potential benefits and risks. Potential side effects and the risk of developing antiviral resistance should be taken into account.  Postpartum, breastfeeding is generally considered safe if the infant receives appropriate immunoprophylaxis, including the hepatitis B vaccine and hepatitis B immune globulin, even if the mother is on antiviral therapy.  These recommendations are consistent with guidelines from the American Association for the Study of Liver Diseases (AASLD) and the European Association for the Study of the Liver (EASL).  Please note, the information provided here should not replace individual medical advice. It’s always recommended to consult with healthcare professionals for personal medical decisions. | 2,1 | 1,1 | 2,1 | 2,1 |
| 87 | AASLD Guideline questions | 高病毒载量的乙型肝炎表面抗原阳性孕妇是否应在妊娠晚期接受抗病毒治疗以预防HBV的围产期传播？ | 是的，对于高病毒载量的乙型肝炎表面抗原阳性孕妇，研究表明在妊娠晚期接受抗病毒治疗可以有效降低HBV的围产期传播风险。  乙型肝炎病毒（HBV）在孕妇中的母婴传播是全球HBV感染的主要途径之一。特别是对于那些HBV DNA水平高（>200,000 IU/mL）的孕妇，如果不进行干预，新生儿在出生后即使接受了乙型肝炎疫苗和HBV免疫球蛋白（HBIG）的免疫预防，也有高达10%的感染风险。  因此，世界卫生组织（WHO）和美国肝病学会（AASLD）推荐在妊娠第三孕期（通常在妊娠28-32周）开始抗病毒治疗，以降低HBV的围产期传播风险。常用的抗病毒药物包括替诺福韦（TDF）和替诺福韦醋酸酯（TAF），这两种药物在孕妇和胎儿中的安全性已得到充分验证。  然而，孕妇在接受抗病毒治疗时仍需要进行密切的监测，以便及时发现和处理可能的药物副作用。在分娩后，根据个体情况，医生可能会建议继续或停止抗病毒治疗。  注意，这些建议可能会随着新的研究证据和临床实践经验的积累而变化，因此在实际操作中，应始终遵循最新的临床指南，并在专业医生的指导下进行治疗决策。 | 乙型肝炎病毒（HBV）的围产期传播是儿童乙型肝炎的主要传播途径，尤其在高病毒载量的乙型肝炎表面抗原（HBsAg）阳性孕妇中。因此，对于这类孕妇，接受抗病毒治疗以预防HBV的围产期传播是十分重要的。  根据世界卫生组织（WHO）的指南，高病毒载量的HBsAg阳性孕妇（即HBV DNA水平高于200,000 IU/ml）应在妊娠第三孕期（28至32周）开始接受抗病毒治疗，以减少HBV的围产期传播。最常用的药物是替诺福韦（Tenofovir）和拉米夫定（Lamivudine），这两种药物在孕期被证明是安全的。  然而，任何治疗决策都应在医生和患者之间进行充分的沟通和共享决策中进行，考虑到患者的个体情况、可能的副作用以及治疗的成本和可行性。此外，抗病毒治疗并不能完全阻止HBV的围产期传播，因此，新生儿应在出生后尽快接种HBV疫苗和HBV免疫球蛋白。  参考文献：  1.World Health Organization. Guidelines for the prevention, care and treatment of persons with chronic hepatitis B infection. Geneva: World Health Organization; 2015.  2.Terrault NA, Lok ASF, McMahon BJ, et al. Update on prevention, diagnosis, and treatment of chronic hepatitis B: AASLD 2018 hepatitis B guidance. Hepatology. 2018;67(4):1560-1599. | 2,2 | 2,2 |  | 2,2 |
| 88 | AASLD Guideline questions | Should children with HBeAg-positive CHB be treated with antiviral therapy to decrease liver-related complications? | Chronic Hepatitis B (CHB) is a significant health concern worldwide. The decision to initiate antiviral therapy in children with HBeAg-positive CHB is complex and should be individualized based on several factors.  Several professional societies have developed guidelines for the management of CHB in children. According to the guidelines from the American Association for the Study of Liver Diseases (AASLD) and the European Association for the Study of the Liver (EASL), antiviral therapy should be considered in children with HBeAg-positive CHB who have persistently elevated alanine aminotransferase (ALT) levels and/or significant histological disease (moderate to severe inflammation and/or fibrosis).  It’s important to note that the goal of treatment in children with CHB is to reduce the risk of long-term complications, such as cirrhosis and hepatocellular carcinoma, by suppressing viral replication and thereby decreasing liver inflammation and fibrosis.  However, there are some caveats:  1.Long-term treatment: Children who start antiviral therapy may require long-term or even lifelong treatment, given the low rates of HBeAg seroconversion. This could increase the risk of antiviral resistance and affect the child’s quality of life.  2.Potential side effects: Antiviral agents have potential side effects, which need to be considered.  3.Drug resistance: Long-term treatment can lead to drug resistance, especially with drugs that have a low genetic barrier to resistance.  Therefore, the decision to initiate antiviral therapy in children with HBeAg-positive CHB should be based on a careful assessment of the potential benefits and risks, and the child’s overall health, growth, and development should be monitored closely during treatment.  This information should not replace a consultation with a healthcare professional. It’s always recommended to discuss these matters with a qualified healthcare provider.  Please note that the field of medicine is continually evolving, and new research may change the current understanding and guidelines.  Sources:  1.AASLD 2018 Hepatitis B Guidance  2.EASL 2017 Clinical Practice Guidelines on the management of hepatitis B virus infection | HBeAg-positive chronic hepatitis B (CHB) in children is a significant global health problem. The management of HBeAg-positive CHB in children is complex and should be individualized based on several factors such as the child’s age, disease phase, the presence of liver disease (e.g., cirrhosis), family history of hepatocellular carcinoma (HCC), and the child’s ability to adhere to treatment.  Antiviral therapy can be beneficial in children with HBeAg-positive CHB, but it is not without potential risks. The primary goal of antiviral therapy is to prevent or delay the progression of liver disease, reduce the risk of hepatocellular carcinoma, and improve the quality of life.  In general, antiviral treatment is recommended for children with HBeAg-positive CHB who have:  1.Persistent or intermittent elevations in alanine aminotransferase (ALT) levels.  2.Evidence of moderate to severe inflammation or fibrosis on liver biopsy.  3.A family history of HCC.  Treatment should be considered in children with normal ALT levels and evidence of moderate to severe inflammation or fibrosis on liver biopsy.  However, the long-term safety and efficacy of antiviral therapy in children are not as well-established as in adults. Moreover, the risk of drug resistance, potential side effects, and the possibility of life-long treatment should be taken into account.  It is important to note that antiviral therapy does not cure CHB but controls the virus. Therefore, regular monitoring of viral load, HBeAg status, and liver function is essential during and after treatment.  In conclusion, the decision to treat a child with HBeAg-positive CHB with antiviral therapy should be made on a case-by-case basis, considering the potential benefits and risks. It is crucial to involve a multidisciplinary team, including a pediatric hepatologist, in the decision-making process.  Please note that this information is based on the current understanding and guidelines. It is always advisable to consult with a healthcare provider for the most appropriate treatment options.  Disclaimer: This information is intended for educational purposes only and should not be used as a substitute for professional medical advice, diagnosis, or treatment. Always seek the advice of your physician or other qualified health provider with any questions you may have regarding a medical condition. | 2,1 | 1, 1 | 2,1 | 2,1 |
| 88 | AASLD Guideline questions | 阳性慢性乙型病毒性肝炎患儿是否应接受抗病毒治疗以减少肝脏相关并发症？ | 乙型病毒性肝炎（HBV）是一种慢性疾病，如果不进行治疗，可能会导致肝硬化、肝衰竭、肝癌等严重并发症。在儿童中，乙型病毒性肝炎的进展速度通常比成人慢，但是，对于阳性慢性乙型病毒性肝炎的儿童，仍然需要考虑抗病毒治疗。  抗病毒治疗的目的是阻止或延缓肝脏病变的进展，减少并发症的发生。然而，是否开始抗病毒治疗并不是一项简单的决定。需要考虑多种因素，包括患者的年龄、疾病的严重程度（如ALT水平，HBV DNA水平，肝脏病变的程度等）、以及患者的个体情况等。  对于儿童，需要特别考虑的是，抗病毒药物可能需要长期使用，而这些药物可能有一些长期的副作用。因此，治疗决策需要在医生、患儿及其家庭之间进行充分的讨论，以权衡治疗的益处和可能的风险。  在一些指南中，如美国肝病学会（AASLD）和欧洲肝病学会（EASL）的指南，建议对于年龄大于2岁，且有活动性病变（ALT水平升高，HBV DNA水平高，肝脏病变明显）的儿童，可以考虑进行抗病毒治疗。  总的来说，对于阳性慢性乙型病毒性肝炎的儿童，应该根据其具体情况来决定是否进行抗病毒治疗。这需要医生根据患儿的病情，以及各种可能的风险和益处，进行个体化的治疗决策。  请注意，这些信息仅供参考，并不能替代医生的专业医疗建议。如果你或你的孩子有任何健康问题，你应该立即联系医疗专业人士。  参考文献：  1.Terrault NA, Lok ASF, McMahon BJ, et al. Update on prevention, diagnosis, and treatment of chronic hepatitis B: AASLD 2018 hepatitis B guidance. Hepatology. 2018;67(4):1560-1599.  2.European Association for the Study of the Liver. EASL 2017 Clinical Practice Guidelines on the management of hepatitis B virus infection. J Hepatol. 2017;67(2):370-398. | 乙型病毒性肝炎（HBV）是一种全球范围内的公共卫生问题。在儿童中，它通常是通过母婴传播或在儿童早期通过接触传播的。乙型病毒性肝炎的治疗旨在防止肝硬化、肝癌和其他肝脏相关并发症。  对于阳性慢性乙型病毒性肝炎的儿童是否应接受抗病毒治疗，这取决于多个因素，包括病毒复制的活跃性、肝炎活动的严重性（例如，ALT水平）、肝脏疾病的严重性（例如，肝纤维化或肝硬化的证据），以及患者的年龄和共患疾病。此外，也要考虑到潜在的药物副作用和耐药性的发展。  一般来说，如果患儿存在以下情况，可能需要抗病毒治疗：  1.高水平的HBV DNA复制  2.ALT水平持续升高  3.肝脏病理学检查显示中度或重度的肝炎和/或显著的肝纤维化  治疗药物包括干扰素和核苷（酸）类似物。干扰素具有抗病毒、抗增殖和免疫调节作用，而核苷（酸）类似物则通过抑制HBV DNA聚合酶来阻止病毒复制。  总的来说，对于阳性慢性乙型病毒性肝炎的儿童，是否接受抗病毒治疗应由医生根据每个患儿的具体情况来决定。医生需要平衡治疗的潜在益处和可能的风险，并与患儿和家庭进行充分的讨论。 | 2,2 | 2,2 |  | 2,2 |
| 89 | Simulated Patients | A 62-year-old male patient, HBeAg positive, HBsAg 10^4 IU/mL, without cirrhosis, ALT 25 U/L, with hypertension, was not on antiviral therapy. Please provide him with advice on therapy and daily monitor and management from the perspective of liver disease treatment and health management. | Your patient is a 62-year-old male with chronic hepatitis B, indicated by HBeAg positivity and a high HBsAg level. He also has hypertension. He does not have cirrhosis and his alanine aminotransferase (ALT) level is 25 U/L, which is within the normal range.  Considering these factors, here is the advice for therapy, monitoring, and management:  Therapy  1.Antiviral therapy: Since the patient is HBeAg positive and has a high HBsAg level, antiviral therapy is recommended to prevent the progression of the disease. First-line antiviral agents include entecavir and tenofovir, which are highly potent with a high barrier to resistance.  2.Hypertension management: The patient should continue managing his hypertension. This may include medication such as ACE inhibitors, beta-blockers, calcium channel blockers, or diuretics, as well as lifestyle changes.  Monitoring  The patient should have regular follow-up visits to monitor his response to antiviral therapy and manage his hypertension. Here are the specific tests he should have:  1.Liver function tests: These should be done every 3-6 months. These tests include ALT, aspartate aminotransferase (AST), and bilirubin.  2.HBV DNA levels: These should be checked every 3-6 months to monitor the response to antiviral therapy.  3.HBeAg and anti-HBe: These should be checked every 6-12 months. If HBeAg seroconversion (loss of HBeAg and gain of anti-HBe) occurs, this is a favorable response to therapy.  4.HBsAg levels: These should be checked every 12 months. Loss of HBsAg is the ultimate goal of therapy, although this is rarely achieved.  5.Screening for hepatocellular carcinoma (HCC): Given the patient’s age and chronic hepatitis B infection, he is at risk for HCC. He should have an ultrasound of the liver and alpha-fetoprotein level checked every 6 months.  6.Blood pressure measurements: These should be checked regularly to ensure his hypertension is well-controlled.  Health Management  1.Healthy diet: The patient should follow a balanced diet, low in saturated and trans fats, cholesterol, salt, and added sugars.  2.Exercise: Regular physical activity can help control blood pressure and maintain a healthy weight.  3.Avoid alcohol: Alcohol can cause additional liver damage and should be avoided.  4.Avoid hepatotoxic drugs: Certain medications can damage the liver and should be avoided.  5.Vaccinations: The patient should receive vaccinations against hepatitis A and other liver-infecting viruses, if not already immune or vaccinated.  6.Regular follow-ups: The patient should have regular follow-ups with his healthcare provider to monitor his condition and adjust his treatment plan as necessary.  Please note that this advice is based on typical practice and may need to be adjusted based on the patient’s individual circumstances. Always consult with a healthcare provider for personalized medical advice. | The patient’s profile indicates that he has chronic hepatitis B infection, which is evident from his HBeAg positivity and high HBsAg level. His liver function, as indicated by the ALT level, is within the normal range, and he also has hypertension. Here’s a general approach for his treatment and management:  1. Antiviral Therapy:  Given the patient’s age, HBeAg positivity, and high HBsAg levels, antiviral therapy should be considered to prevent disease progression. The decision to start therapy should be made based on a combination of factors including age, ALT levels, HBV DNA levels, and the presence of liver disease. The American Association for the Study of Liver Diseases (AASLD) recommends antiviral therapy in HBeAg-positive patients with HBV DNA > 20,000 IU/mL and ALT > 2 times the upper limit of normal[^1^].  In this patient, the ALT level is normal, so a discussion about the risks and benefits of antiviral therapy should be had with the patient. Options for first-line antiviral therapy include entecavir, tenofovir disoproxil fumarate, or tenofovir alafenamide[^1^].  2. Regular Monitoring:  The patient should be monitored regularly. This includes:  (1)Liver Function Tests: To monitor the disease progression, regular liver function tests (including ALT, AST, bilirubin, albumin, and INR) should be done.  (2)HBV DNA Levels: The patient should have his HBV DNA levels monitored every 3-6 months to assess the response to antiviral therapy.  (3)Ultrasound: An ultrasound of the liver should be performed every 6 months to monitor for signs of liver cirrhosis or hepatocellular carcinoma.  3. Management of Hypertension:  The patient’s hypertension should be managed according to guidelines. This usually involves lifestyle modifications (e.g., diet, exercise), and if necessary, antihypertensive medications.  4. Lifestyle Modifications:  The patient should be advised to:  (1)Maintain a healthy weight  (2)Limit alcohol consumption  (3)Avoid hepatotoxic medications  (4)Eat a balanced diet  (5)Regular physical activity  (6)Regular follow-up with a healthcare provider  5. Vaccinations:  Ensure that the patient is up to date on vaccinations, especially hepatitis A, as co-infection can lead to severe disease.  6. Screening for Hepatocellular Carcinoma (HCC):  Because of the increased risk of HCC in patients with chronic hepatitis B, the patient should undergo regular screening with ultrasound, with or without alpha-fetoprotein measurement, every 6 months[^1^].  7. Family and Close Contacts:  Family and close contacts of the patient should be screened for HBV infection and vaccinated if they are not immune.  Please note that this advice is based on general guidelines and may need to be modified based on the patient’s individual circumstances, preferences, and the treating physician’s clinical judgment.  [^1^]: Terrault NA, Lok ASF, McMahon BJ, et al. Update on prevention, diagnosis, and treatment of chronic hepatitis B: AASLD 2018 hepatitis B guidance. Hepatology. 2018;67(4):1560-1599. doi:10.1002/hep.29800 | 1,1 | 1, 1 |  | 1, 1 |
| 89 | Simulated Patients | 一个62岁的男性患者，HBeAg阳性，HBsAg 10^4 IU/mL，没有肝硬化，ALT 25 U/L，有高血压，未进行抗病毒治疗。请从肝病治疗和健康管理的角度，给他提供治疗和日常管理方面的建议。 | 这个患者是慢性乙型肝炎患者，HBeAg阳性，HBsAg水平相对较高，但ALT正常。他还有高血压病史。以下是对他的治疗和健康管理的一些建议：  1.抗病毒治疗：尽管ALT水平正常，但是由于他的年龄和HBeAg阳性，抗病毒治疗是推荐的。可以考虑使用核苷(酸)类似物，如恩替卡韦或替诺福韦，这些药物有较低的耐药性，适合长期使用。  2.高血压管理：需要定期监测血压，并采取适当的药物和生活方式干预。避免高盐、高脂肪食物，增加运动，保持良好的生活习惯。  3.定期监测：需要定期监测肝功能（包括ALT）、HBV DNA、HBeAg/抗HBe、HBsAg等指标，评估病毒复制情况和肝炎活动程度。  4.生活方式：建议保持健康的生活方式，包括适当的运动，保持良好的饮食习惯，避免饮酒和吸烟。  5.疫苗接种：建议接种肺炎疫苗和季节性流感疫苗，以防止并发症。  6.心理健康：慢性疾病可能对心理健康产生影响，建议寻求必要的心理支持。  以上建议只是基于所提供的信息，具体治疗方案应由医生根据患者的全面病情决定。 | 这位患者是一位62岁的男性，HBeAg阳性，HBsAg 10^4 IU/mL，ALT 25 U/L，没有肝硬化但有高血压的情况。首先，他的HBeAg阳性和较高的HBsAg水平意味着他有活跃的乙型肝炎病毒（HBV）感染。尽管他的ALT水平处于正常范围内，但是鉴于他的年龄和活跃的HBV感染，建议他开始接受抗病毒治疗。  药物治疗：  对于乙型肝炎的抗病毒治疗，通常推荐的第一线药物包括骨化三磷酸酶酮（Tenofovir）和恩曲他滨（Entecavir）。这些药物可以有效抑制HBV的复制，减少肝炎的活跃程度，降低肝硬化和肝癌的风险。这位患者应该与医生讨论开始使用这些药物的可能性。  健康管理：  1.饮食：保持均衡的饮食，避免过度摄入脂肪、盐和糖，以控制高血压。避免饮酒，因为酒精可以损害肝脏。  2.运动：适度的运动可以帮助控制高血压，同时也有利于整体健康。建议每周进行150分钟的中等强度运动或者75分钟的高强度运动。  3.定期检查：定期进行血压检查，以及HBV感染的监测，包括ALT、HBsAg和HBV DNA水平。这有助于及时发现病情变化，及时调整治疗方案。  4.避免传染他人：乙型肝炎是可以通过血液和体液传播的。应避免共用刀具，使用避孕套，不共用牙刷和剃须刀等。  5.药物管理：如果开始抗病毒治疗，需要每天按时服药，避免漏服。同时，需要定期随访，监测药物的效果和可能的副作用。  以上是针对这位患者的一些基本建议。具体的治疗方案应由医生根据患者的具体情况来决定。 | 2,1 | 2,1 |  | 2,1 |
| 90 | Simulated Patients | A 51-year-old male patient, HBeAg-negative, HBsAg 10^1 IU/mL, without cirrhosis, ALT 28 U/L, was not on antiviral therapy and was an habitual smoker. Does he need antiviral treatment and why? | According to current clinical guidelines, the need for antiviral therapy in chronic hepatitis B patients is determined based on several factors, including:  1.Hepatitis B e antigen (HBeAg) status  2,Hepatitis B surface antigen (HBsAg) levels  3.Presence of cirrhosis  4.Alanine aminotransferase (ALT) levels  5.Patient’s age  6.History of antiviral treatment  In this case, the patient is a 51-year-old male, HBeAg-negative, with HBsAg levels at 10^1 IU/mL (which is considered low), without cirrhosis, and ALT levels at 28 U/L. The patient is not currently on antiviral therapy and is a habitual smoker.  Given these parameters, let’s analyze the need for antiviral treatment:  1.HBeAg Status: The patient is HBeAg-negative. This generally indicates a lower viral replication rate, but it doesn’t necessarily mean that the virus is inactive or that liver damage isn’t occurring.  2.HBsAg Levels: The patient has a HBsAg level of  IU/mL. This is a low level, which generally indicates a lower level of viral activity.  3.Presence of Cirrhosis: The patient does not have cirrhosis. This means the liver is not severely damaged, which is a positive factor in this case.  4.ALT Levels: ALT level of the patient is 28 U/L. Normal ALT levels for men can range up to 45 U/L. So, the ALT level of this patient is within the normal range, suggesting no significant liver inflammation or damage at the moment.  5.Age and Smoking: The patient is 51 years old and a habitual smoker. Age is a risk factor for progression of liver disease, and smoking may contribute to liver damage and progression of liver disease as well.  Based on these parameters, the patient does not appear to need antiviral therapy at the moment. His low HBsAg levels, absence of cirrhosis, and normal ALT levels suggest a low level of viral activity and minimal liver damage. However, the patient’s age and smoking habit are risk factors for liver disease progression.  Therefore, the patient should be closely monitored, with regular liver function tests, HBV DNA levels, and ultrasound examinations. It’s also crucial for him to quit smoking to reduce the risk of liver disease progression.  Please note that this is a general interpretation based on the information provided, and actual treatment decisions should be made by a healthcare provider based on a comprehensive assessment of the patient’s health status. | According to the guidelines for managing chronic hepatitis B, antiviral treatment is recommended based on a combination of factors such as the patient’s hepatitis B e antigen (HBeAg) status, levels of hepatitis B surface antigen (HBsAg), alanine aminotransferase (ALT) levels, and the presence of cirrhosis.  The patient in question is HBeAg-negative, has HBsAg levels of 10^1 IU/mL, ALT of 28 U/L, and does not have cirrhosis. The patient is also a habitual smoker, which is a risk factor for liver disease, but does not directly influence the decision to start antiviral therapy for hepatitis B.  The criteria for starting antiviral treatment in HBeAg-negative patients without cirrhosis are typically:  1.ALT >2 times the upper limit of normal (ULN). The ULN is often considered around 40 U/L, so ALT >80 U/L would meet this criterion.  2.HBV DNA >2000 IU/mL.  In this patient, the ALT is 28 U/L, which is less than the ULN. The level of HBsAg is provided, but the level of HBV DNA, which is a different measure, is not given. Therefore, based on the provided information, the patient does not meet the criteria for antiviral treatment.  However, it’s important to note that individual patient characteristics and clinical judgement should always be taken into account when making treatment decisions. This patient should be monitored closely given his smoking history and the potential for progression of liver disease.  This advice is based on the current guidelines, but treatment decisions should be made in consultation with a healthcare provider who can evaluate the patient’s overall health status and consider the potential benefits and risks of antiviral therapy. | 1,1 | 1, 1 |  | 1, 1 |
| 90 | Simulated Patients | 一个51岁的男性患者，HBeAg阴性，HBsAg 10^1 IU/mL，没有肝硬化，ALT 28 U/L，未进行抗病毒治疗，平时有吸烟习惯。他是否需要应该进行抗病毒治疗，理由是什么？ | 对于是否需要抗病毒治疗的决定，一般基于患者的年龄、HBeAg状态、HBsAg水平、ALT（丙氨酸氨基转移酶）水平、肝病状态以及是否有其他并发症等因素。这些因素都是影响患者治疗决策的重要参数。  在这个具体的病例中，我们有以下信息：  1.年龄：51岁  2.HBeAg状态：阴性  3.HBsAg水平：10^1 IU/mL  4.ALT水平：28 U/L  5.肝病状态：没有肝硬化  6.吸烟习惯：有  根据这些信息，我们可以进行以下分析：  1.年龄：患者年龄51岁，属于成年人群，需要考虑治疗。  2.HBeAg状态：HBeAg阴性可能表明患者的病毒复制活动较低，但并不能排除肝炎的可能性。  3.HBsAg水平：HBsAg水平低（10^1 IU/mL）可能表明患者的病毒负荷较低，但也需要结合其他指标综合判断。  4.ALT水平：ALT是肝脏受损的重要指标。患者的ALT水平28 U/L，低于两倍的正常上限（通常为40 U/L），表明患者的肝脏受损程度可能较轻。  5.肝病状态：患者没有肝硬化，这是一个积极的信号，表明他的肝脏功能可能尚未严重受损。  6.吸烟习惯：吸烟可能增加肝病的风险，这是一个需要关注的因素。  根据以上信息，这位患者可能不需要立即进行抗病毒治疗，因为他的ALT水平和HBsAg水平都相对较低，没有肝硬化的迹象。然而，他的吸烟习惯可能增加肝病的风险，因此应当戒烟，并定期进行肝功能检查和病毒载量监测，以便及时发现任何可能的问题。  然而，这仅仅是基于所提供信息的一种推测。最终的决定需要由医生根据患者的全面病情和身体状况来做出。 | 根据《世界卫生组织（WHO）乙型肝炎治疗指南》以及一些国家的肝炎指南，对于HBeAg阴性的患者，需要考虑以下几个因素来决定是否开始抗病毒治疗：  1.HBsAg 水平：HBsAg 是乙型肝炎病毒表面抗原，其浓度反映了病毒在体内的活跃程度。这位患者的 HBsAg 水平为 10^1 IU/mL，这个水平较低，说明病毒活跃程度较低。  2.肝功能（ALT 水平）：ALT 是肝脏中的酶，当肝脏受损时，ALT 会释放到血液中，因此 ALT 水平是衡量肝脏炎症和损伤程度的重要指标。对于男性，ALT 正常范围通常是 7-55 U/L。这位患者的 ALT 水平是 28 U/L，属于正常范围。  3.肝硬化：肝硬化是乙型肝炎病毒持续感染和慢性炎症导致的肝脏纤维化和结构重塑。这位患者没有肝硬化。  4.抽烟：抽烟可以增加肝病的风险，尤其是肝癌。但是，抽烟本身并不能直接决定是否需要进行抗病毒治疗。  综上所述，考虑到这位患者的 HBsAg 水平较低，ALT 水平正常，没有肝硬化，他可能不需要立即进行抗病毒治疗。然而，他应该定期进行肝功能检测和病毒载量监测，以便在必要时及时开始治疗。此外，他应该考虑戒烟，以减少肝病的风险。  这些建议基于现有的临床指南，但每个患者的情况都是独特的，所以他应该与医生详细讨论他的病情和治疗选择。 | 1,1 | 1, 1 |  | 1, 1 |
| 91 | Simulated Patients | A 37-year-old female patient with HBV-DNA 10^7 IU/ml, HBeAg positive, cirrhosis, eGFR 102 ml/min, ALT 215 U/L, BMI 24.3kg/m^2, no alcohol habit, had just started treatment with tenofovir. How often should the patient be monitored? What should the project include? | The monitoring of a patient with chronic hepatitis B, especially with high viral load and cirrhosis, should be diligent and comprehensive.  The American Association for the Study of Liver Diseases (AASLD) and the European Association for the Study of the Liver (EASL) have published guidelines for the management and monitoring of patients with chronic hepatitis B.  Here’s a suggested monitoring plan based on those guidelines:  1. Frequency of monitoring:  Every 3 months during the first year of treatment. This is particularly important because your patient has a high viral load (HBV-DNA 10^7 IU/ml) and elevated ALT (215 U/L).  After the first year, if the patient is responding well to treatment, monitoring can be reduced to every 6 months.  If the patient develops any complications or the treatment needs to be changed, more frequent monitoring may be necessary.  2. Monitoring should include:  (1)Virologic response: HBV DNA levels should be checked regularly to assess the effectiveness of the treatment. A decline in HBV DNA levels indicates a good response to treatment. HBV DNA should ideally become undetectable with effective antiviral therapy.  (2)Biochemical response: Monitor ALT levels. A decline towards the normal range is a positive sign.  (3)Serologic response: Check HBeAg and anti-HBe status. In HBeAg-positive patients, loss of HBeAg and seroconversion to anti-HBe is a favorable prognostic sign.  (4)Hepatic function: Regularly monitor liver function tests to assess the progression of liver disease.  (5)Renal function: As your patient is on tenofovir, regular monitoring of renal function (eGFR) is important due to the potential nephrotoxicity of this drug.  (6)Screening for hepatocellular carcinoma (HCC): Patients with chronic hepatitis B and cirrhosis are at high risk for developing HCC. Regular screening with liver ultrasound, with or without alpha-fetoprotein (AFP) measurement, every 6 months is recommended.  (7)Assessment of cirrhosis and liver fibrosis: This can be done via imaging studies, transient elastography, or serum biomarkers.  (8)Assessment of patient’s overall health and potential drug side effects: This should include monitoring BMI, checking for signs of lactic acidosis (a rare side effect of tenofovir), and monitoring for any signs of drug intolerance.  Remember that this is a general plan and individual patient needs may vary. It’s important to tailor the monitoring plan to each patient’s specific condition, response to treatment, and tolerance of the medication.  Please consult with a specialist or refer to the original guidelines for the most accurate information. | According to the American Association for the Study of Liver Diseases (AASLD) and European Association for the Study of the Liver (EASL) guidelines, the patient should be monitored as follows:  1.Frequency of Monitoring: The patient should be monitored every 3-6 months.  2.Monitoring Parameters: The monitoring should include the following:  (1)Biochemical Response: Regular monitoring of Alanine aminotransferase (ALT) levels is necessary. This is an enzyme found mostly in the liver and it’s used as a marker of liver inflammation and damage. Normal ALT levels range from 7 to 56 units per liter (U/L). The patient’s ALT level is significantly high (215 U/L), suggesting active liver inflammation.  (2)Virological Response: The HBV DNA levels should be checked every 3-6 months. The aim is to achieve undetectable HBV DNA levels using a sensitive PCR assay (<10-15 IU/mL).  (3)Serological Response: The HBeAg status should be monitored every 6-12 months. The goal is to achieve HBeAg seroconversion (loss of HBeAg and development of anti-HBe).  (4)Hepatic Function: Regular monitoring of hepatic function is important to assess the progress of cirrhosis and potential development of hepatic decompensation. This typically includes tests such as albumin, bilirubin, INR, and platelet count.  (5)Screening for Hepatocellular Carcinoma (HCC): Given the patient has cirrhosis, they are at an increased risk for HCC. Therefore, ultrasound screening every 6 months is recommended.  (6)Renal Function: Tenofovir can potentially cause renal dysfunction. Therefore, monitoring renal function (eGFR) every 3-6 months is necessary.  (7)Bone Mineral Density (BMD): Tenofovir may also affect bone health. Therefore, monitoring of BMD may be needed, especially in patients with risk factors for osteoporosis.  These monitoring parameters and frequency could change based on the patient’s response to treatment and any new symptoms or complications that might arise. It’s also important to continue counseling on avoiding alcohol and maintaining a healthy weight (BMI). | 1,1 | 1, 1 |  | 1, 1 |
| 91 | Simulated Patients | 一个37岁的女性患者，HBV-DNA 10^7 IU/ml，HBeAg阳性，有肝硬化，eGFR 102 ml/min，ALT 215 U/L，BMI 24.3kg/m^2，没有饮酒习惯，刚开始服用替诺福韦治疗。这位患者日常监测的频率应当为多久一次？项目应当包括哪些？ | 这位患者是慢性乙型肝炎（CHB）合并肝硬化的患者，现在开始使用替诺福韦进行抗病毒治疗。对于这类患者，我们通常会建议以下的监测方案：  1.治疗早期阶段（治疗的头三个月）：每月进行一次血常规、肝功能（包括ALT、AST、TBil、DBil、ALP、GGT、TP、Alb）、肾功能（包括BUN、Cr、eGFR）、电解质（包括K、Na、Cl、Ca）、尿常规、HBV-DNA定量、HBeAg/抗HBe、AFP等检测。这是为了及时了解药物的疗效和潜在的副作用。  2.治疗稳定阶段（治疗后三个月以后）：如果患者的病情稳定，HBV-DNA水平明显下降，肝肾功能正常，可以适当延长监测的间隔，比如每3个月进行一次血常规、肝功能、肾功能、电解质、尿常规、HBV-DNA定量、HBeAg/抗HBe、AFP等检测。  3.治疗中期和长期阶段：每6个月进行一次肝脏的影像学检查（如肝脏超声、MRI或CT），以及食管胃底静脉曲张的筛查（如胃镜检查）。  4.如果患者出现疲乏、食欲不振、恶心、呕吐、尿色深、大便色浅、皮肤或眼睛黄染等症状，应立即进行相关检查。  以上监测方案是一种常规的建议，具体的监测频率和项目应根据患者的具体病情和临床反应来定。 | 首先，让我们明确一点：所有的医学建议应当由医生或医疗专业人员提供。我可以提供一些关于慢性乙型肝炎病毒（HBV）感染的一般监测和管理建议，但这些仅供参考，不能替代专业医疗建议。  这位患者的情况下，她的HBV-DNA水平非常高，表明病毒复制活跃，而且她已经有肝硬化的情况。这可能需要更密集的监测。替诺福韦是一种抗病毒药物，常用于治疗乙型肝炎。  通常，对于正在接受抗病毒治疗的慢性乙型肝炎患者，以下是一些可能的监测项目和频率：  1.ALT（丙氨酸氨基转移酶）：ALT是肝脏疾病的一个重要指标。这位患者的ALT已经超过正常范围，需要密切监测。通常，治疗初期可以每1-3个月进行一次ALT检测。  2.HBV-DNA：HBV-DNA水平可以反映病毒复制的活跃程度。治疗初期，可以每3个月进行一次HBV-DNA检测。  3.HBeAg：HBeAg阳性表示病毒复制活跃。治疗过程中，如果HBeAg转为阴性，可能表示病毒活动降低。可以每6-12个月进行一次HBeAg检测。  4.肝硬度：由于这位患者已经有肝硬化，可能需要定期进行肝硬度或肝脏超声检查，以监测肝脏疾病的进展。频率可以每6-12个月一次。  5.肾功能：替诺福韦可能对肾功能产生影响。这位患者的eGFR（估计肾小球滤过率）表明她的肾功能目前正常，但在治疗过程中可能需要定期进行肾功能检查，包括血肌酐和尿蛋白。频率可以每3-6个月一次。  以上都是一般的建议，具体的监测项目和频率应由医生根据患者的具体情况来决定。 | 1,1 | 1, 1 |  | 1, 1 |
| 92 | Simulated Patients | A 36-year-old female patient with HBV-DNA 10^6 IU/ml, HBeAg positive, uncertain whether she had cirrhosis, eGFR 109 ml/min, ALT 119 U/L, BMI 30.2kg/m^2, diabetes mellitus, had been treated with entecavir for 1 year. How should this patient conduct daily monitoring and lifestyle management? | This patient is dealing with chronic hepatitis B infection and other comorbidities, including obesity and diabetes mellitus. Here are some general suggestions for daily monitoring and lifestyle management:  1. Regular Monitoring:  (1)Hepatitis B: Regular monitoring with liver function tests, HBV DNA, and HBeAg every 3-6 months is recommended. The patient should also be screened for hepatocellular carcinoma (HCC) every 6 months using ultrasound, especially if cirrhosis is confirmed.  (2)Diabetes Mellitus: Regular monitoring of blood glucose levels is crucial. HbA1c tests should be done every 3 months to assess the long-term control of blood glucose.  (3)Obesity: Monitor weight and BMI regularly. Consider a body composition analysis if available.  2. Medication:  (1)Hepatitis B: The patient should continue taking entecavir as prescribed by the doctor. Adherence to antiviral therapy is crucial for controlling viral replication.  (2)Diabetes Mellitus: Regular use of antidiabetic medications as prescribed. Insulin therapy might be required depending on the severity of diabetes.  3. Diet:  (1)Hepatitis B: A balanced diet is important. Try to avoid alcohol and limit foods that are high in fat and sugar. Drink plenty of fluids.  (2)Diabetes Mellitus: The patient should follow a diabetes-friendly diet, rich in fiber, and low in simple carbohydrates and unhealthy fats.  (3)Obesity: A low-calorie diet can be considered under the supervision of a dietitian.  4. Exercise:  Regular physical activity is beneficial for all three conditions. Aim for at least 150 minutes of moderate-intensity or 75 minutes of high-intensity exercise per week.  5. Mental Health:  Chronic diseases can lead to stress and mental health issues. Regular mental health check-ups, stress management, and relaxation techniques can be beneficial.  6. Regular Check-ups:  Regular follow-ups with the healthcare provider to monitor the progress of the disease and adjust treatments as necessary.  Remember, these are general recommendations and the patient’s individual plan may vary based on her specific circumstances and overall health. Always consult with healthcare professionals for personalized advice. | The patient should follow a comprehensive approach for the management of her chronic hepatitis B virus (HBV) infection, along with her diabetes mellitus and obesity. Here are some recommendations:  1. Regular Monitoring:  (1)Hepatitis B: The patient should continue to have regular follow-up with her hepatologist or infectious disease specialist. This typically involves blood tests every 3-6 months to monitor HBV-DNA levels, HBeAg status, and liver function tests (including ALT). The patient may also need regular imaging studies (like ultrasound) to monitor for signs of liver damage or cirrhosis.  (2)Diabetes Mellitus: The patient should monitor her blood glucose levels regularly as advised by her healthcare provider. HbA1c levels should be checked every 3-6 months to monitor long-term blood glucose control. Regular follow-up with her endocrinologist or primary care physician is essential.  (3)Obesity: The patient should monitor her weight regularly and aim for a gradual weight loss (if advised by her healthcare provider). Her BMI should be calculated at each visit.  (4)Kidney function: As the patient’s eGFR is currently normal, regular monitoring of kidney function is recommended, especially given her use of antiviral medication and presence of diabetes.  2. Lifestyle Management:  (1)Diet: The patient should follow a balanced diet that is low in saturated fats, sugar, and salt. She should consume a diet rich in fruits, vegetables, lean proteins, and whole grains. Given her diabetes, she should work with a dietician to create a meal plan that helps control her blood sugar levels.  (2)Exercise: Regular physical activity is important. She should aim for at least 150 minutes of moderate-intensity or 75 minutes of vigorous-intensity aerobic physical activity per week, along with muscle-strengthening activities on 2 or more days a week.  (3)Alcohol and Smoking: The patient should avoid alcohol and smoking, as they can exacerbate liver damage and negatively affect diabetes control.  (4)Medication Adherence: It’s crucial that the patient adheres to her prescribed medications, including entecavir for HBV and any medications she may be on for diabetes.  (5)Vaccinations: The patient should receive all recommended vaccinations, including the annual flu vaccine and the pneumococcal vaccine, given her chronic medical conditions.  (6)Mental Health: Chronic diseases can often lead to stress, anxiety, and depression. Regular mental health check-ups should be incorporated into her healthcare routine.  It’s important that the patient has regular follow-ups with her healthcare team and that any changes in her condition are communicated promptly. Any changes in her symptoms, such as jaundice, abdominal pain, persistent nausea/vomiting, or worsening fatigue, should be reported immediately to her healthcare provider. | 2,2 | 2,1 | 2,1 | 2,1 |
| 92 | Simulated Patients | 一个36岁的女性患者，HBV-DNA 10^6 IU/ml，HBeAg阳性，不确定自己是否有肝硬化，eGFR 109 ml/min，ALT 119 U/L，BMI 30.2kg/m^2，有糖尿病，已经服用恩替卡韦治疗1年。这位患者应当如何进行日常监测与生活方式管理？ | 这位患者的情况显示她有慢性乙型肝炎（HBV），并且可能存在肝硬化的风险。她的HBV-DNA水平很高，这可能表示病毒复制活跃，肝脏疾病可能正在进展。此外，她的ALT（丙氨酸氨基转移酶）水平超过正常值，这可能表示肝脏受损。她的eGFR（肾小球滤过率）在正常范围内，表示肾功能良好。然而，她的BMI（体重指数）显示她的体重超重，可能存在肥胖，并且已经有糖尿病。这可能会加重她的肝病和其他健康问题。  对于这位患者的日常监测和生活方式管理，以下是一些建议：  日常监测：  1.HBV-DNA: 每3-6个月进行一次检测。如果病毒载量持续升高，可能需要调整治疗方案。  2.ALT: 每3-6个月进行一次检测。如果ALT水平持续升高，可能表示肝病进展，需要进一步的检查和治疗。  3.肝硬度检测: 建议定期进行肝硬度检测（如声波弹性成像）以评估是否有肝硬化的迹象。  4.肾功能检测: 继续监测eGFR，以确保肾功能良好。特别是在使用恩替卡韦等抗病毒药物时，需要定期检查肾功能。  5.血糖和血压: 由于糖尿病，需要定期监测血糖和血压，确保其控制在合理范围内。  生活方式管理：  1.饮食: 建议低盐、低脂、高蛋白质的饮食。尽量避免饮酒和吃油腻、辛辣食物。增加新鲜蔬菜和水果的摄入。  2.体重管理: 由于BMI超标，需要进行体重管理。可以通过合理的饮食和定期运动来控制体重。  3.运动: 建议定期运动，如散步、瑜伽或游泳，以保持健康的体重和改善血糖控制。  4.服药: 坚持每日服用恩替卡韦，不要随意停药或改变剂量。定期向医生报告药物的副作用。  5.定期随访: 与医生保持密切的联系，定期进行随访，及时报告任何新的症状或不适。  这些都是一般性的建议，具体的监测频率和生活方式管理还需要根据患者的个体情况和医生的建议进行调整。 | 对于这位患者，我们需要考虑她的慢性乙型肝炎，可能的肝硬化，糖尿病，以及肥胖症的综合管理。下面是一些关于日常监测和生活方式管理的建议：  1.药物治疗和监测：恩替卡韦是一种抗病毒药物，用于治疗乙型肝炎。患者应继续按照医生的建议服用，并定期监测肝功能和HBV-DNA水平，以评估病毒的活动和药物的效果。一般情况下，这些检查应该每3-6个月进行一次。  2.肝硬化的评估：虽然患者没有明确的肝硬化病史，但是乙型肝炎的患者有发展为肝硬化的风险。因此，她需要定期进行肝脏超声检查，以便及时发现可能的肝硬化或肝癌。这些检查通常每6-12个月进行一次。  3.糖尿病管理：患者应定期监测血糖和HbA1c水平，以评估糖尿病的控制情况。同时，她应该遵循一个低糖、低盐、低脂肪的饮食，并进行适量的运动，以帮助控制血糖和体重。  4.体重管理：BMI 30.2kg/m^2表明患者处于肥胖状态，这可能会加重糖尿病和肝病的病情。因此，患者需要进行体重管理，包括合理饮食和运动。她应该寻求营养师的帮助，制定一个个性化的饮食计划。  5.生活方式：患者应该避免饮酒和吸烟，因为这些都可能加重肝病和糖尿病的病情。同时，她应该保持充足的休息和良好的心理状态，以帮助身体应对这些慢性疾病。  6.定期体检：患者应定期进行全面的体检，包括血压、血脂、心脏等方面的检查，以便及时发现和处理可能的并发症。  以上建议仅供参考，具体的治疗和管理方案应由患者的主治医生根据她的具体情况制定。 | 1, 1 | 1,2 | 1,2 | 1,2 |
| 93 | Simulated Patients | A 44-year-old female patient with HBV-DNA 10^8 IU/ml, HBeAg positive, no cirrhosis, eGFR 105 ml/min, ALT 110 U/L, BMI 22.5kg/m^2, a history of hypertension, smoking and drinking habits, and no antiviral therapy. Please advise the medication regimen and lifestyle of the patient. | The patient’s profile suggests chronic Hepatitis B infection with high viral load and elevated ALT, indicating active viral replication and liver inflammation. This necessitates antiviral therapy. The patient also has risk factors such as hypertension, smoking, and drinking habits that need to be addressed.  Here is a suggested management plan:  Antiviral Therapy  The first-line treatment options for chronic Hepatitis B include:  1.Entecavir (ETV): It’s a potent antiviral with a high barrier to resistance. The usual dose is 0.5 mg once daily.  2.Tenofovir disoproxil fumarate (TDF) or Tenofovir alafenamide (TAF): TDF (300 mg once daily) or TAF (25 mg once daily) are potent antivirals with a high barrier to resistance. They are also recommended as first-line therapy. TAF has a better renal and bone safety profile than TDF.  Given the patient’s eGFR is 105 ml/min, either ETV, TDF, or TAF could be considered. However, TAF might be a safer option considering the patient’s history of hypertension.  Lifestyle Modification  1.Quit Smoking and Drinking: It is crucial for the patient to stop smoking and alcohol consumption, as both can exacerbate liver damage. She should be referred to smoking cessation and alcohol cessation programs.  2.Healthy Diet and Exercise: The patient should maintain a balanced diet, rich in fruits, vegetables, lean proteins, and whole grains. Regular exercise will also help maintain a healthy weight and lower blood pressure.  3.Regular Monitoring: The patient should have regular follow-ups with her doctor to monitor her liver function, viral load, and the effectiveness of the treatment.  4.Vaccination: Vaccination against Hepatitis A and pneumococcal disease is recommended because of the increased risk of severe disease in individuals with liver disease. The patient should also receive an annual flu shot.  5.Blood Pressure Control: The patient’s hypertension should be managed according to standard guidelines. This may include lifestyle modifications and antihypertensive medications.  6.Safe Practices: To prevent transmission to others, the patient should avoid sharing personal items such as toothbrushes, razors, or needles. She should also practice safe sex.  Disclaimer: This is a general advice based on the information provided and might not be fully applicable to the patient. The patient should consult with her healthcare provider for a personalized treatment plan.  Please note that any changes to medication or lifestyle should be discussed and monitored by a healthcare provider. | Given the patient’s condition, it is recommended to start antiviral therapy to reduce the risk of disease progression. Based on the patient’s high viral load (HBV-DNA  IU/ml), HBeAg positivity, elevated ALT levels, and absence of cirrhosis, first-line antiviral medications would be appropriate.  Medication Regimen  The first-line antiviral medications for Hepatitis B include:  1.Entecavir (ETV): This is a potent inhibitor of HBV replication with a high barrier to resistance. The recommended dose is 0.5 mg once daily.  2.Tenofovir Disoproxil Fumarate (TDF): This is another potent inhibitor of HBV replication with a high barrier to resistance. The recommended dose is 300 mg once daily.  3.Tenofovir Alafenamide (TAF): This is a newer prodrug of tenofovir that is associated with less renal and bone toxicity. The recommended dose is 25 mg once daily.  Given the patient’s good renal function (eGFR 105 ml/min), either of these medications could be used. However, the choice between these medications should take into account the patient’s comorbid conditions, potential side effects, and the patient’s preferences.  The patient should also continue to manage her hypertension, potentially with antihypertensive medication if it is not already controlled. The specific antihypertensive medication would depend on the patient’s blood pressure levels, other medical conditions, and potential side effects.  Lifestyle Changes  In addition to medication, lifestyle changes are also crucial for managing Hepatitis B and reducing the risk of disease progression:  1.Quit Smoking and Limit Alcohol: Smoking and alcohol can increase liver damage and reduce the effectiveness of treatment. The patient should be advised to quit smoking and limit or avoid alcohol.  2.Healthy Diet: A balanced diet rich in fruits, vegetables, lean proteins, and whole grains can help maintain overall health and support liver function.  3.Regular Exercise: Regular physical activity can help maintain a healthy weight and overall health. The patient’s BMI is currently in the normal range, so the goal of exercise would be to maintain this.  4.Regular Monitoring: Regular follow-ups with the healthcare provider are necessary to monitor the patient’s liver function, viral load, and response to treatment.  5.Avoid Sharing Personal Items: The patient should avoid sharing personal items like toothbrushes and razors to prevent spreading the virus to others.  6.Safe Sex Practices: Use of condoms can prevent the transmission of the virus to sexual partners.  7.Vaccination: Family members and close contacts who are not already immune to Hepatitis B should be vaccinated.  Remember, this is just a general recommendation and the specific treatment plan should be personalized based on the patient’s individual circumstances. It’s important that the patient discusses these recommendations with her healthcare provider. | 1,1 | 1, 1 |  | 1, 1 |
| 93 | Simulated Patients | 一个44岁的女性患者，HBV-DNA 10^8 IU/ml，HBeAg阳性，没有肝硬化，eGFR 105 ml/min，ALT 110 U/L，BMI 22.5kg/m^2，有高血压病史，有吸烟和饮酒习惯，未进行抗病毒治疗。请给出用药方案和生活方式建议。 | 这位患者的HBV-DNA水平很高，ALT也超过正常范围，这表明她的肝脏受到了病毒的严重损害。以下是我对她的治疗建议：  药物治疗方案：  1.核苷（酸）类似物（NAs）：这是治疗慢性乙型肝炎的首选药物。目前，国际上推荐的首选药物有替诺福韦酯（TDF）和恩替卡韦酯（ETV）。这两种药物的耐药率低，长期使用安全性高。鉴于这位患者的eGFR正常，可以考虑使用替诺福韦酯。  2.干扰素：干扰素具有抗病毒和免疫调节作用，但因为有一定的副作用，且与高血压、饮酒和吸烟等因素有关，可能不适合这位患者。  生活方式建议：  1.戒烟戒酒：吸烟和饮酒都可能加重肝脏损害，并增加肝癌的风险。因此，这位患者应该立即停止吸烟和饮酒。  2.健康饮食：保持均衡的饮食，减少盐和糖的摄入，增加蔬菜和水果的摄入，有助于控制高血压。  3.定期运动：适量的运动可以帮助保持健康的体重，同时也有助于降低血压。  4.定期检查：应定期进行肝功能检查和病毒载量检测，以监测病情和治疗效果。  这些建议只是基于目前的临床信息，具体的治疗方案应由医生根据患者的病情和身体状况制定。 | 首先，需要明确这是一名乙型肝炎病毒(HBV)活动性感染的患者，HBV-DNA 10^8 IU/ml，HBeAg阳性，ALT 110 U/L均提示病毒活跃，且有肝脏炎症。此外，患者存在高血压，吸烟和饮酒的不良生活习惯，这些都是对肝脏健康不利的因素。下面是针对这位患者的药物治疗建议和生活方式建议。  药物治疗建议：  1.抗病毒治疗：考虑到患者的HBV-DNA水平、HBeAg状态和ALT水平，抗病毒治疗是必要的。可以选择核苷（酸）类似物（Nucleos(t)ide analogues, NAs）如恩替卡韦（Entecavir）或替诺福韦酯（Tenofovir disoproxil fumarate）或替诺福韦磷酸醋酸酯（Tenofovir alafenamide）。这些药物可以有效抑制HBV复制，降低病毒载量，改善肝炎症，防止肝硬化和肝癌的发展。  2.高血压管理：对于高血压，需要继续使用抗高血压药物，如ACE抑制剂，ARBs，钙通道阻滞剂等，具体药物需要根据患者的血压控制情况和可能存在的其他疾病（如糖尿病、心脏病等）来选择。  生活方式建议：  1.饮食管理：建议患者保持均衡的饮食，多食用蔬菜、水果和全谷类，减少盐和油的摄入，避免过度饮酒和吃油腻、辛辣、煎炸食物。  2.戒烟戒酒：吸烟和饮酒都会加重肝脏损伤，尤其是在乙肝患者中，吸烟和饮酒可以增加肝癌的风险。因此，强烈建议患者戒烟和戒酒。  3.适量运动：建议患者每周进行至少150分钟的中等强度有氧运动（如快走，慢跑等），或者75分钟的高强度有氧运动（如跑步，游泳等），以及每周至少两次的大肌群力量训练。  4.定期随访：患者需要定期进行血常规、肝功能、肾功能、HBV-DNA、HBeAg、肝硬度等检查，以及定期心电图和血压监测，以便及时调整治疗方案。  以上建议仅供参考，具体治疗方案请按照医生的建议进行。 | 2,1 | 2,1 |  | 2,1 |
| 94 | Simulated Patients | A 50-year-old male patient, HBeAg positive, ALT 231 U/L, with hyperlipidemia and currently not on antiviral therapy, came to console whether interferon could be used. Please give medical advice. | The patient in question is HBeAg positive, which indicates active viral replication of hepatitis B, and has an elevated ALT level (Alanine Aminotransferase, a liver enzyme), suggesting liver inflammation or damage. This usually necessitates antiviral treatment.  Interferon is one of the treatment options for chronic hepatitis B. It’s an immune modulator that enhances the body’s immune response to the virus, aiming to achieve sustained viral suppression even after treatment cessation. Interferon can be used in certain patients with chronic hepatitis B infection, but it’s not suitable for everyone due to its side effects and contraindications.  However, the patient also has hyperlipidemia, a condition characterized by high levels of fats (lipids) in the blood. Interferon therapy is not directly contraindicated in patients with hyperlipidemia, but it can cause a wide range of side effects including flu-like symptoms, mood disturbances, thyroid dysfunction, and, importantly, it may exacerbate existing conditions.  Before deciding on interferon therapy, several factors should be considered:  1.The patient’s overall health status: If the patient has significant comorbidities, interferon might not be the best choice due to its potential side effects.  2.The patient’s lifestyle and personal preferences: Interferon treatment involves regular injections, usually for a period of 48 weeks. This can have a significant impact on the patient’s quality of life.  3.The patient’s viral load and liver disease stage: Interferon is generally more effective in patients with lower viral loads and less advanced liver disease.  4.Potential for drug interactions: If the patient is taking medication for hyperlipidemia, potential interactions with interferon should be evaluated.  In conclusion, while interferon could potentially be used in this patient, it’s crucial to discuss the potential risks and benefits with the patient, considering his overall health status, lifestyle, and personal preferences. Moreover, monitoring the patient’s condition closely during treatment would be essential.  As an AI language model, I can provide general advice, but the final decision should be made by a healthcare professional based on a comprehensive evaluation of the patient’s condition. | Based on the information provided, the patient is a 50-year-old male with HBeAg-positive chronic hepatitis B, elevated ALT levels (231 U/L), and hyperlipidemia.  Interferon is one of the treatment options for chronic hepatitis B. It works by boosting the immune system to fight the virus. It’s often considered as a treatment option due to its finite treatment duration and lack of resistance. However, the use of interferon is limited by its side effects and contraindications. Side effects can include flu-like symptoms, depression, and a drop in white blood cells.  As for contraindications, interferon is generally not recommended for patients with:  1.Decompensated cirrhosis.  2.A history of severe psychiatric disease, especially depression.  3.Autoimmune disease.  4.Uncontrolled thyroid disease.  5.Heart disease.  6.Seizure disorders.  In this case, the patient’s hyperlipidemia would not necessarily preclude the use of interferon, but the patient’s overall health status, including liver disease stage, cardiovascular risk, and mental health, should be thoroughly evaluated.  Also, it’s important to note that the patient’s elevated ALT levels may indicate active liver inflammation or damage. This should be further investigated to understand the extent of liver disease.  Before making any treatment decisions, further tests should be performed including:  1.Hepatitis B virus DNA level  2.Liver ultrasound  3.Complete blood count  4.Coagulation parameters  5.Tests to rule out co-infection with hepatitis C, hepatitis D, and HIV  6.Possibly a liver biopsy  The patient should also be counseled on lifestyle modifications including a healthy diet, regular exercise, avoidance of alcohol, and regular monitoring.  Remember that the ultimate decision on treatment type and timing should be individualized, taking into account the patient’s age, disease phase, viral replication, ALT level, presence of liver inflammation or fibrosis, family history of hepatocellular carcinoma, patient’s willingness, and possible side effects.  Please consult with a hepatologist or an infectious disease specialist for a comprehensive evaluation and personalized treatment plan.  Disclaimer: This advice is intended to be general in nature, and specific causes may not apply to individual cases. It should not replace consultation with a healthcare provider. | 2,2 | 2,2 |  | 2,2 |
| 94 | Simulated Patients | 一个50岁的男性患者，HBeAg阳性，ALT 231 U/L，有高脂血症，现在未进行抗病毒治疗，前来咨询是否可以使用干扰素。请给出诊疗意见。 | 首先，我们需要考虑到患者的年龄，ALT（丙氨酸氨基转移酶）水平，HBeAg（乙型肝炎e抗原）阳性状态，以及高脂血症的存在。对于这位患者来说，他的ALT水平较高，这可能表明他的肝脏炎症活跃。  干扰素是一种抗病毒药物，用于治疗乙型肝炎。但是，干扰素的使用存在一定的限制。年龄较大的患者，尤其是超过40岁的患者，使用干扰素可能效果较差。此外，干扰素可能会引起一些副作用，如流感样症状、发热、疲劳、食欲减退、抑郁等。  对于高脂血症的患者，需要注意的是干扰素可能会影响脂质代谢，进一步增加血脂水平。  因此，对于这位50岁的乙肝患者，他的年龄和高脂血症可能会限制干扰素的使用。在决定治疗方案时，应充分考虑这些因素，并与患者进行详细的讨论。可能需要考虑其他的抗病毒治疗方案，如核苷（酸）类似物（NAs）。  最后，这个决定应由患者的医生进行，他们会根据患者的具体情况和病史，以及患者的意愿，来确定最佳的治疗方案。  请注意：以上信息仅供参考，具体治疗方案应由医生决定。 | 在对该患者是否可以使用干扰素进行治疗的问题上，我们需要考虑以下几个关键因素：  1.年龄：干扰素疗法通常对年轻患者效果较好。50岁的患者可能会有一些副作用，例如流感样症状、疲劳、抑郁等。  2.ALT水平：ALT（丙氨酸氨基转移酶）是肝脏疾病的一个重要指标。ALT 231 U/L 表明患者的肝脏受到了一定的损伤。干扰素可能会加重肝脏损伤，因此在开始治疗之前，需要详细评估患者的肝脏功能。  3.HBeAg阳性：HBeAg阳性表明患者体内病毒复制活跃，这可能使干扰素治疗的效果降低。  4.高脂血症：干扰素可能会影响脂质代谢，进一步加重高脂血症。  综上所述，我建议该患者在开始干扰素治疗之前，应该进行详细的健康评估，包括肝脏功能、心血管风险等。同时，应与医生讨论其他可能的抗病毒治疗选项，例如核苷（酸）类似物（NAs）治疗，这种治疗方式通常副作用较小，对高脂血症患者更安全。请遵照医生的建议，并定期进行健康检查和监测。  请注意，这只是基于您提供的信息做出的一般性建议。具体的治疗方案应由医生根据患者的整体健康状况和病史制定。 | 2,1 | 2,2 | 2,1 | 2,1 |
| 95 | Simulated Patients | A 46-year-old man came to the hospital because of "positive hepatitis B surface antigen detected by physical examination." What tests might he need? | The presence of Hepatitis B surface antigen (HBsAg) in a patient’s blood usually indicates an active Hepatitis B virus (HBV) infection. To confirm the diagnosis and assess the stage and severity of the disease, several additional tests may be required:  1.Hepatitis B core antibody (anti-HBc): This test is used to detect a previous or ongoing infection with HBV. The presence of anti-HBc suggests a past or current infection.  2.Hepatitis B surface antibody (anti-HBs): The presence of anti-HBs indicates that the patient has developed immunity to the virus, which could be either from successful vaccination or from recovery from a past infection.  3.Hepatitis B e antigen (HBeAg) and Hepatitis B e antibody (anti-HBe): The presence of HBeAg in a patient’s serum indicates high viral replication and high infectivity. Conversely, the presence of anti-HBe often signifies lower levels of the virus and reduced infectivity.  4.Hepatitis B DNA: This test measures the amount of HBV DNA in the blood, which can indicate the level of viral replication. High levels of HBV DNA correlate with more severe liver disease.  5.Liver function tests (LFTs): These tests, including ALT and AST, assess the functionality of the liver and can indicate the degree of liver damage.  6.Liver biopsy: In some cases, a liver biopsy may be required to assess the extent of liver damage.  7.Ultrasound of the liver: This imaging test can help identify any structural changes to the liver, such as cirrhosis or liver cancer, which can result from chronic HBV infection.  Remember, the appropriate tests depend on the clinical scenario and should be decided by the healthcare provider. It’s also important to consider vaccination and follow-up testing for close contacts of the patient, given the infectious nature of HBV. | A positive test for hepatitis B surface antigen (HBsAg) indicates that a person is infectious and can spread the virus to others. This finding can also suggest an active Hepatitis B infection, either acute or chronic. Therefore, to better understand the patient’s condition and develop an appropriate management plan, the following tests may be recommended:  1.Hepatitis B core antibody (anti-HBc): This test is usually positive in people who have been infected with the hepatitis B virus at some point. It can remain positive even after the infection has cleared.  2.Hepatitis B e antigen (HBeAg) and Hepatitis B e antibody (anti-HBe): The presence of HBeAg in a patient’s serum indicates active viral replication and high infectivity. Conversely, the presence of anti-HBe often signifies lower levels of viral replication and lower infectivity.  3.Hepatitis B virus DNA (HBV DNA): This test measures the amount of hepatitis B virus in the blood. High levels suggest active viral replication.  4.Liver function tests (LFTs): These tests, including ALT and AST, assess the health of the liver and can indicate whether the virus is causing liver damage.  5.Liver biopsy: In some cases, a liver biopsy may be recommended to determine the degree of liver damage or to confirm a diagnosis of hepatitis B.  6.Tests for co-infections: Hepatitis B patients are at risk of co-infection with other viruses such as hepatitis C and HIV, so screening for these conditions may also be recommended.  7.Hepatitis B surface antibody (anti-HBs): This test measures the immunity against hepatitis B. It’s usually positive after vaccination or after the resolution of an acute hepatitis B infection.  These tests will provide a more comprehensive understanding of the patient’s Hepatitis B infection status and help guide further management. It’s also crucial to monitor the patient’s condition regularly and assess their response to any treatments provided. | 2,2 | 2,2 |  | 2,2 |
| 95 | Simulated Patients | 一位46岁男性患者，因“体检发现乙肝表面抗原阳性”前来就诊。他可能需要进行何种检查？ | 如果一位患者的乙肝表面抗原（HBsAg）检测结果阳性，那么他可能需要进行以下检查来更全面地评估他的乙肝状态：  1.乙肝e抗原（HBeAg）和乙肝e抗体（anti-HBe）：这些标志物有助于判断病毒复制的活跃程度和感染的阶段。  2.乙肝核心抗体（anti-HBc）：这是一种用来判断是否曾经或正在感染乙肝病毒的标志物。  3.乙肝病毒DNA定量（HBV DNA Quantitative Test）：这种检查有助于判断病毒复制的活跃程度，以及评估治疗效果和监控病情。  4.肝功能检查：包括ALT、AST、ALP、GGT、总胆红素、直接胆红素等，用来评估肝脏损伤的程度。  5.肝脏超声波或肝脏CT扫描：这些影像学检查有助于评估肝脏的结构和大小，以及是否有肝硬化或肝癌等并发症的出现。  6.肝脏活检：在某些情况下，可能需要通过肝脏活检来评估肝脏病变的程度和类型。  以上检查项目需要根据患者的具体情况由医生来决定。 | 如果一位46岁的男性患者在体检中发现乙型肝炎（HBV）表面抗原（HBsAg）阳性，这可能意味着他正在经历乙型肝炎的活动感染。他可能需要进行以下进一步的检查：  1.乙型肝炎病毒DNA定量检测（HBV DNA）：这项检查可以帮助评估病毒复制的活跃程度和感染的严重程度。  2.乙型肝炎表面抗体（Anti-HBs）：如果这项检查结果为阳性，可能表明患者已经产生了免疫反应，或者曾经接种过乙肝疫苗。  3.乙型肝炎e抗原（HBeAg）和乙型肝炎e抗体（Anti-HBe）：这两项检查可以帮助评估病毒的活跃程度和感染的严重程度。  4.乙型肝炎核心抗体（Anti-HBc）：这项检查可以帮助确定是否曾经或正在经历乙肝感染。  5.肝功能检测：包括ALT、AST、ALP、GGT、总蛋白、白蛋白、总胆红素、直接胆红素等，这些指标可以评估肝脏的损伤程度和功能状态。  6.肝脏影像学检查：如超声波、CT、MRI等，这些检查可以帮助评估肝脏的解剖结构，检查是否有肝硬化、肝肿瘤等并发症。  7.肝脏活检：在一些情况下，可能需要进行肝脏活检以评估肝炎的活动程度和肝纤维化的程度。  这些检查结果将有助于医生确定患者的病情，并制定适当的治疗方案。 | 2,2 | 2,2 |  | 2,2 |
| 96 | Simulated Patients | A 32-year-old woman presented with "abdominal distention and poor appetite". The patient had severe yellowing of the skin and sclera, abdominal distention, and positive shifting dullness. Her mother had hepatitis B without treatment. List tests she might need and give possible diagnoses. | The symptoms presented by the patient indicate a possible liver disease. The yellowing of the skin and sclera (jaundice) suggests a high level of bilirubin, which may be due to liver dysfunction. Abdominal distention and positive shifting dullness could be signs of ascites, a condition often associated with severe liver diseases. The history of untreated Hepatitis B in her mother could also suggest a genetic predisposition or a direct transmission of the disease.  Tests  1.Complete Blood Count (CBC): This can reveal anemia, thrombocytopenia, or leucopenia, which can occur in liver diseases.  2.Liver Function Tests (LFTs): These tests, including ALT, AST, ALP, GGT, total bilirubin, and albumin, can provide information about the functional status of the liver and help diagnose liver disease.  3.Serologic tests for Hepatitis B and C: Given her mother’s history, testing for Hepatitis B is crucial. Hepatitis C should also be ruled out.  4.Ultrasound of the abdomen: This can help detect the presence of ascites, and provide information about the size and structure of the liver and spleen.  5.Computed Tomography (CT) or Magnetic Resonance Imaging (MRI): These can provide more detailed images of the liver and other abdominal organs.  6.Paracentesis: If ascites is present, a sample of the fluid can be taken and analyzed to help determine the cause.  7.Liver Biopsy: If other tests do not provide a clear diagnosis, a biopsy can be performed to directly examine the liver tissue.  Possible Diagnoses  1.Hepatitis B: Given her mother’s history, this is a likely possibility. Hepatitis B can lead to serious liver damage if left untreated.  2.Cirrhosis: This is a late stage of scarring (fibrosis) of the liver caused by many forms of liver diseases and conditions, such as hepatitis and chronic alcoholism.  3.Hepatocellular Carcinoma (HCC): This is a type of liver cancer that often occurs in people with chronic liver diseases, such as Hepatitis B or cirrhosis.  4.Cholestasis: This is a condition where bile cannot flow from the liver to the duodenum. The two basic types of this condition are intrahepatic cholestasis, where the problem exists inside the liver, and extrahepatic cholestasis, where the problem exists outside the liver.  5.Primary Biliary Cholangitis (PBC): This is a type of liver disease caused by damage to the bile ducts in the liver.  The definitive diagnosis will depend on the results of the diagnostic tests. | The patient’s presentation suggests a possible liver disease, given her symptoms of jaundish (yellowing of skin and sclera), abdominal distention, and positive shifting dullness which is indicative of ascites (accumulation of fluid in the peritoneal cavity). Her family history of untreated Hepatitis B is also a significant risk factor.  Tests Needed:  1.Liver Function Tests (LFTs): This will include tests for serum bilirubin, serum albumin, alanine transaminase (ALT), aspartate transaminase (AST), alkaline phosphatase (ALP), and prothrombin time.  2.Complete Blood Count (CBC): To evaluate for anemia, infection, or other hematologic abnormalities.  3.Abdominal Ultrasound: To visualize the liver and assess for cirrhosis, hepatomegaly, or other structural abnormalities. It can also confirm the presence of ascites.  4.Hepatitis B and C Serology: Given her mother’s history of untreated Hepatitis B, it is important to rule out viral hepatitis.  5.Autoimmune Markers: Such as antinuclear antibody (ANA), anti-smooth muscle antibody (ASMA), and anti-liver kidney microsomal antibody (LKM-1) to evaluate for autoimmune hepatitis.  6.Serum Alpha-fetoprotein (AFP): This is a tumor marker that can be elevated in hepatocellular carcinoma.  7.Paracentesis with Ascitic Fluid Analysis: If ascites is present, a sample of the fluid can be analyzed for protein, cell count, and culture to help determine the cause.  Possible Diagnoses:  1.Hepatitis B: Given her mother’s history, this is a possible diagnosis. Chronic hepatitis B can lead to cirrhosis and other complications.  2.Alcoholic Liver Disease: If the patient has a history of alcohol abuse, this could explain her symptoms.  3.Non-alcoholic Steatohepatitis (NASH): This is a type of fatty liver disease that can lead to cirrhosis.  4.Autoimmune Hepatitis: The patient’s symptoms could be due to an autoimmune attack on the liver.  5.Hepatocellular Carcinoma (HCC): This is a type of liver cancer that can present with her symptoms. It’s often a complication of chronic liver disease.  6.Cirrhosis: This could be the result of many types of chronic liver diseases, including hepatitis B, alcoholic liver disease, and NASH.  7.Cholestatic Liver Disease: This could be due to a blockage in the bile ducts, which can cause jaundice and ascites.  Please note that these are possible diagnoses based on the provided symptoms. The patient should undergo further evaluation to confirm the diagnosis. It’s also important to consider that she may have more than one of these conditions concurrently. | 2,2 | 2,1 | 2,2 | 2,2 |
| 96 | Simulated Patients | 一位32岁女性患者，因“腹胀、胃口差”前来就诊。患者皮肤和巩膜严重发黄，腹部膨隆，移动性浊音阳性。她母亲患有乙肝，没有进行过治疗。她可能需要接受哪些检查？列举并给出可能的诊断。 | 根据这位患者的症状，可能的诊断有很多种，包括但不限于肝硬化、肝炎、胆囊疾病、胆管疾病或肝癌。因为她的母亲患有乙型肝炎，这可能增加了她患有肝病的风险。以下是一些可能需要进行的检查：  1.血液检查：可以检查肝功能指标（ALT、AST、ALP、GGT、总胆红素、直接胆红素等）、肾功能、电解质、凝血功能等。  2.乙肝相关血清学标志物检查：包括HBsAg、HBsAb、HBeAg、HBeAb、HBcAb等，用于判断是否感染乙型肝炎，以及病毒复制的活跃程度。  3.腹部超声：可以检查肝、胆、胰、脾等器官的形态和结构，查看是否有腹水、肝硬化、肝占位性病变等情况。  4.CT或MRI：如果超声发现异常，可能需要进一步的影像学检查，以了解病变的具体位置和范围。  5.肝穿刺活检：如果需要明确诊断，可能需要进行肝穿刺活检，对肝脏组织进行病理学检查。  根据这位患者的症状和家族史，可能的诊断包括：  1.肝硬化：症状可能包括腹胀（由于腹水积聚）、食欲不振、皮肤和巩膜黄染等。肝硬化可能由乙型肝炎引起。  2.乙型肝炎：如果她的母亲患有乙型肝炎，她可能也有患病风险。乙型肝炎可能导致肝损伤和肝硬化。  3.肝癌：肝癌的症状可能包括食欲不振、腹部肿胀和黄疸。乙型肝炎是肝癌的一个重要危险因素。  请注意，以上信息只是根据你提供的信息做出的可能性推测，实际诊断需要医生根据实际检查结果来确定。 | 根据这位女性患者的症状，我们可以考虑以下几种可能的检查：  1.血液检查：包括肝功能检查（ALT、AST、ALP、GGT、总胆红素、直接胆红素、间接胆红素等）、全血细胞计数、凝血功能检查（PT、APTT、INR、Fib）等，以评估肝脏功能和是否存在贫血或凝血功能异常。  2.腹部超声检查：可以检查肝脏、胆囊、胆管、脾脏等腹部器官的形态和大小，以及是否存在腹水。  3.乙型肝炎病毒相关检查：包括乙肝表面抗原（HBsAg）、乙肝表面抗体（HBsAb）、乙肝e抗原（HBeAg）、乙肝e抗体（HBeAb）、乙肝核心抗体（HBcAb）、乙型肝炎病毒DNA（HBV DNA）等，以评估是否存在乙型肝炎病毒感染以及感染的活跃程度。  4.肝硬化相关评估：可以通过肝脏弹性测量（如瞬时弹性成像，Transient Elastography，TE）或血清学标志物（如肝硬化指数，Cirrhosis Index）评估是否存在肝硬化。  5.腹腔穿刺检查：如果有大量腹水，可以进行腹腔穿刺抽取腹水进行检查，以评估是否存在感染（如自发性细菌性腹膜炎）或者肝硬化腹水。  可能的诊断包括：  1.乙型肝炎：考虑到她的母亲有乙肝，她可能也患有乙肝。  2.肝硬化：她的腹胀、移动性浊音阳性可能是由于腹水引起的，这是肝硬化的常见症状。  3.肝功能衰竭：皮肤和巩膜严重发黄可能是由于胆红素代谢紊乱引起的，这可能是肝功能衰竭的表现。  4.肝癌：如果肝硬化得不到控制，可能会发展成肝癌。  以上是基于目前信息的初步分析，具体诊断需要依赖于医生的临床经验和相关检查结果。 | 2,2 | 2,2 |  | 2,2 |
