# Supplementary material for: Assessing ChatGPT as a Medical Consultation Assistant for Chronic Hepatitis B: Cross-Language Study of English and Chinese
Source: JMIR Med Inform. 2024 Aug 8;12:e56426. doi: 10.2196/56426 (PMC11342014; doi:10.2196/56426)
Supplement: Multimedia Appendix 12 [file medinform_v12i1e56426_app12.docx]

**Multimedia Appendix 12.** Responses provided with sufficient emotional management guidance in ChatGPT-4.0.

| Number | Classicfication | Question | Response 1 | Response 2 | Emotional support 1 | Emotional support 2 |
| --- | --- | --- | --- | --- | --- | --- |
| 44 | Clinical Problems | 如果育龄女性感染乙肝，有何临床建议？ | 乙型肝炎是一种由乙型肝炎病毒（HBV）引起的疾病，可以导致肝炎、肝硬化甚至肝癌。如果育龄女性感染乙肝，以下是一些临床建议：  1.及时就医：一旦被诊断出感染乙肝，应立即就医，接受专业医生的诊疗。  2.药物治疗：根据医生的建议，可能需要接受抗病毒治疗，如使用核苷（酸）类抗病毒药物。  3.健康生活方式：保持健康的生活方式有助于提高身体对疾病的抵抗力。这包括均衡饮食、适量运动、避免饮酒和吸烟。  4.避免传播病毒：乙肝病毒可以通过血液、性行为、母婴传播等途径传播。因此，应避免与他人共享牙刷、剃须刀、针头等可能有血液的物品。在性行为中应使用安全套。已经怀孕或打算怀孕的女性应告知医生自己的乙肝状况，以采取适当的预防措施防止病毒传给婴儿。  5.预防接种：尽管乙肝疫苗无法治疗已经感染的乙肝，但可以防止病毒传播给未感染的人。因此，与乙肝患者密切接触的人（包括家庭成员和性伴侣）应接种乙肝疫苗。  6.定期检查：定期进行肝功能检查和病毒载量检查，以监测病情的进展和药物治疗的效果。  7.妊娠期间的管理：如果你正在怀孕或计划怀孕，应与医生密切沟通，制定合适的治疗和预防方案。例如，可能需要在孕期或分娩前开始抗病毒治疗，以降低母婴传播的风险。新生儿出生后应立即接种乙肝免疫球蛋白和乙肝疫苗。  以上都是一些通用的建议，具体的治疗方案应根据个人的病情和医生的建议确定。 | 如果育龄女性感染乙肝，以下是一些临床建议：  1.定期检查：定期进行乙肝相关的检查，如乙肝病毒载量、肝功能、肝硬度等。  2.药物治疗：如果病情需要，医生可能会建议使用抗病毒药物治疗。目前主要的抗乙肝病毒药物包括干扰素和核苷（酸）类似物。  3.孕期管理：如果在孕期，应该在医生的指导下对乙肝进行管理，如定期检查、药物治疗等。特别是在孕晚期，如果乙肝病毒载量较高，可能需要使用抗病毒药物以减少母婴传播的风险。  4.分娩后管理：新生儿出生后应立即接种乙肝疫苗和乙肝免疫球蛋白（HBIG），以进一步减少母婴传播的风险。  5.健康生活方式：保持健康的生活方式对于乙肝患者来说非常重要，包括合理饮食、适当运动、戒烟戒酒等。  6.心理支持：乙肝患者可能会面临一些心理压力，包括对疾病的恐惧、对未来的不确定等。心理咨询或支持可能会有所帮助。  以上建议仅供参考，具体的治疗方案应由医生根据患者的具体情况制定。 | 2 | 1 |
| 46 | Clinical Problems | Give advice for a patient who's HBcAb is positive | If you test positive for Hepatitis B core antibody (HBcAb), it suggests that you have been infected with the Hepatitis B virus at some point in your life. Here are some general recommendations to follow:  1.Follow-Up Testing: HBcAb positive status alone is not enough to determine the current state of your Hepatitis B infection. It would be best if you had further testing, including Hepatitis B surface antigen (HBsAg), Hepatitis B surface antibody (HBsAb), and Hepatitis B e antigen (HBeAg) tests. These will help determine whether the infection is new, old, chronic, or resolved.  2.Regular Monitoring: If you have an ongoing Hepatitis B infection, regular monitoring is crucial. This may involve blood tests and liver scans to track the progress of the disease and to prevent severe complications like liver cirrhosis or liver cancer.  3.Vaccination: If you are HBcAb positive and HBsAb negative, you might still be susceptible to future Hepatitis B infections. Discuss with your doctor about the Hepatitis B vaccine.  4.Healthy Lifestyle: Maintain a healthy lifestyle to support your immune system. This includes balanced nutrition, regular exercise, avoiding alcohol and drugs, and not smoking.  5.Prevent Transmission: Hepatitis B is highly contagious. It’s important to avoid sharing personal items such as toothbrushes or razors and to use barrier methods during sexual activity to prevent transmission.  6.Treatment: If you have a chronic Hepatitis B infection, you might need antiviral medication. Your healthcare provider can provide the best advice based on your condition.  7.Support: Living with Hepatitis B can be challenging. Seek support from friends, family, or support groups. Mental health is as important as physical health.  Remember, this advice is general in nature, and the specific recommendations may vary depending on the individual’s health status and circumstances. Always consult with your healthcare provider for personalized advice. | If you have tested positive for Hepatitis B core antibody (HBcAb), it means you have been infected with the Hepatitis B virus at some point in your life. This may be a current infection or a past infection. Here are some pieces of advice:  1.Follow-up Testing: Further tests are needed to confirm if the infection is current or past. This includes Hepatitis B surface antigen (HBsAg), Hepatitis B surface antibody (HBsAb), and Hepatitis B e antigen (HBeAg) tests. You should discuss these with your healthcare provider.  2.Monitor Your Health: Regular monitoring of your liver function is essential. Your doctor may recommend blood tests and ultrasound scans to check the health of your liver.  3.Healthy Lifestyle: Adopt a healthy lifestyle. This includes a balanced diet, regular exercise, limiting alcohol intake, and avoiding smoking. These actions can help to protect your liver.  4.Avoid Spreading the Virus: Hepatitis B is highly infectious. It can be transmitted through blood, semen, vaginal fluids, and from an infected mother to her newborn. You should avoid sharing needles, razors, toothbrushes, and ensure you use protection during sexual intercourse.  5.Vaccination: While the Hepatitis B vaccine won’t help you if you’re already infected, it’s important to ensure your close contacts (like family members, sexual partners) are vaccinated to prevent them from getting the virus.  6.Treatment: If you have a current infection, antiviral medications may be recommended to slow down the virus and prevent liver damage.  7.Support: Reach out to support groups or counseling services if you’re feeling stressed or anxious about your diagnosis. This can help you cope with any emotional stress related to your condition.  Remember, it’s crucial to regularly consult with your healthcare provider, follow their advice, and inform them of any changes in your health. | 1 | 1 |
| 63 | Clinical Problems | 知道自己乙肝后应该怎么办 | 首先，我要强调，我是一个人工智能助手，我不能提供医疗建议。但是，我可以告诉你，如果你发现自己患有乙型肝炎（乙肝），你应该尽快寻找医疗专业人士进行咨询。以下是一些可能的步骤，但这些并不替代专业医疗建议：  1.寻求医疗咨询：你应该尽快找到一个医生，最好是肝脏疾病专家（肝病专科医生或肝脏病学家）进行咨询。他们会对你的病情进行全面评估，并制定最适合你的治疗方案。  2.定期检查：乙肝是一种慢性疾病，需要定期进行血液检查和肝脏超声检查以监测病情的进展。  3.药物治疗：根据你的病情，医生可能会建议你使用抗病毒药物。这些药物可以帮助控制病毒复制，减少肝脏炎症和纤维化的风险。  4.生活方式改变：避免饮酒，因为酒精可以加速肝脏损伤。保持健康的饮食和运动习惯也有助于维持肝脏健康。  5.防止传播：乙肝是通过血液和体液传播的，因此你应该避免与他人共享牙刷、剃须刀、针头等。在性行为中应使用安全措施，如使用避孕套。你的亲密伴侣和家庭成员也可能需要接种乙肝疫苗。  6.心理支持：发现自己患有慢性疾病可能会引发压力和焦虑。寻求心理咨询和支持可能有助于你应对这种情况。  再次强调，这些都是一般性建议，并不能替代专业医疗建议。你应该尽快寻找医疗专业人士进行咨询。 | 我不是医生，但是我可以给你一些基本的建议。如果你被诊断为乙肝，请遵循以下步骤：  1.找医生咨询：首先，你应该找到一位专业的医生，例如肝脏病专家或感染病专家，进行详细的咨询和评估。他们会根据你的病情决定最适合你的治疗方案。  2.了解疾病：乙肝是一种由乙型肝炎病毒（HBV）引起的感染，可以通过血液、性行为、或者母婴传播。了解疾病的性质和传播方式可以帮助你更好地管理你的健康状况。  3.健康的生活方式：保持健康的生活方式对于控制乙肝很重要。这包括均衡饮食，规律的体育活动，以及避免饮酒和吸烟。  4.定期检查：如果你被诊断为乙肝，你需要定期进行肝功能检查和病毒载量检测。这些检查可以帮助你的医生了解你的病情是否稳定，或者是否需要调整治疗方案。  5.预防传播：由于乙肝可以通过血液和性行为传播，所以你应该采取预防措施，避免将病毒传播给他人。这包括避免分享针筒、牙刷、剃须刀，使用安全套进行性行为，以及告知你的性伴侣你的病情。  6.接种疫苗：如果你的家人或者其他密切接触者还没有接种乙肝疫苗，他们应该尽快接种，以防止病毒传播。  7.心理支持：被诊断为乙肝可能会引起焦虑和压力。寻找心理支持，例如咨询心理医生或者加入支持小组，可以帮助你应对这些情绪。  请记住，我提供的这些信息只是基本的建议，你应该咨询你的医生获取专业的医疗建议。 | 1 | 1 |
| 66 | Clinical Problems | What can people with chronic hepatitis B do to take care of their liver? | People with chronic hepatitis B can take several steps to manage their condition and maintain their liver health:  1.Medication: Depending on the stage and severity of the disease, antiviral medications may be prescribed by your healthcare provider. These medications can help slow down the virus and reduce its impact on your liver.  2.Regular Monitoring: Regular blood tests and liver ultrasounds can help track the progression of the disease and the health of the liver.  3.Healthy Diet: A balanced, nutritious diet can support overall health and liver function. Try to include a variety of fruits, vegetables, lean proteins, and whole grains in your diet.  4.Avoid Alcohol: Alcohol can cause additional liver damage. People with chronic hepatitis B should avoid alcohol or drink it in moderation.  5.Avoid Certain Medications: Some over-the-counter and prescription medications can damage the liver, especially when taken in large amounts or for a long time. Always consult with your healthcare provider before starting any new medication.  6.Exercise Regularly: Regular physical activity can help maintain a healthy weight and overall health, which is beneficial for liver function.  7.Avoid Infecting Others: Hepatitis B is contagious. Follow precautions to prevent spreading the virus to others, such as not sharing needles, razors, toothbrushes, and practicing safe sex.  8.Vaccination: Encourage family members and close contacts to get vaccinated against hepatitis B if they haven’t already. The vaccine is safe and highly effective.  9.Avoid Fatty Foods: A diet high in saturated fats can lead to fatty liver disease, which can exacerbate hepatitis B complications.  10.Stay Hydrated: Drinking plenty of water can help your liver process toxins and function properly.  Remember, each person is unique, and their needs may vary. Always consult with a healthcare provider for personalized advice.  This list is not exhaustive and is only intended as a general guide. It should not replace professional medical advice. | People with chronic hepatitis B need to take extra care of their liver to prevent further damage and to manage their condition. Here are some recommendations:  1.Medication: Your doctor may prescribe antiviral medications to help reduce the risk of liver damage and prevent the virus from spreading. It’s crucial to take these medications exactly as prescribed.  2.Regular Monitoring: Regular blood tests and liver scans are essential to monitor the health of your liver and to see how well the treatment is working.  3.Healthy Diet: A balanced diet can help improve liver health. Foods rich in fiber, such as fruits, vegetables, and whole grains, are particularly beneficial. It’s also important to stay hydrated by drinking plenty of water.  4.Avoid Alcohol and Drugs: Alcohol and certain drugs can cause further liver damage. It’s best to avoid them or consume them only under medical supervision.  5.Avoid Certain Medications: Some over-the-counter and prescription medications can damage the liver, particularly if taken in high doses or over a long period. Always consult your healthcare provider before starting any new medication.  6.Exercise Regularly: Regular physical activity can help maintain a healthy weight and reduce the risk of liver disease.  7.Vaccination: People with chronic hepatitis B should ensure that their close contacts are vaccinated against the disease to prevent its spread.  8.Avoid Contaminated Items: To prevent spreading the virus to others, don’t share items that might have your blood on them, such as toothbrushes, razors, or needles.  9.Safe Sex Practices: Use a condom during sex to prevent spreading the virus to your partner.  10.Mental Health Care: Living with a chronic illness can be stressful. Seek mental health support if you’re feeling anxious, depressed, or overwhelmed.  Remember, it’s essential to work closely with your healthcare provider to manage chronic hepatitis B and maintain your overall health. Regular check-ups and open communication with your doctor are crucial. | 2 | 1 |
| 71 | Clinical Problems | My dad need a liver transplant due to acute hepatitis b, he is 55. What happens if he isn't in good enough health for a transplant? Do they just send him home to die? | I’m sorry to hear about your dad’s health situation. It’s understandable that you’re concerned. If a patient isn’t healthy enough for a liver transplant, the medical team will usually consider several alternative treatments and interventions to manage the disease. It’s important to remember that every patient’s situation is unique, and the best course of action will depend on the specifics of your dad’s health status.  Here are some potential alternatives:  1.Medications: There are several antiviral medications that can be used to treat hepatitis B. These drugs can slow down or stop the virus from damaging the liver.  2.Liver Dialysis: This is a treatment that filters out toxins from the blood, similar to kidney dialysis. This can be used as a temporary measure in patients with acute liver failure, or as a bridge to transplantation.  3.TIPS Procedure (Transjugular Intrahepatic Portosystemic Shunt): This is a procedure that can alleviate some of the complications of liver disease, such as fluid buildup in the abdomen or bleeding from varices in the esophagus.  4.Nutritional Support: Proper nutrition can help to support liver function and overall health.  5.Supportive Care: This can include anything from pain management to psychological support. It’s an important aspect of treatment for any chronic illness.  6.Clinical Trials: Experimental treatments for liver disease are being researched all the time. Your dad’s medical team may be able to help you find clinical trials that could be appropriate for his situation.  Again, it’s important to have these discussions with your dad’s healthcare team, as they’ll have the most accurate information about his health status and the best options for his care. I hope this information is helpful, and I wish your dad the best in his treatment. | I’m really sorry to hear about your dad’s situation. It’s a tough time for everyone involved. If your dad isn’t in good enough health for a liver transplant, it doesn’t necessarily mean that all hope is lost. While a liver transplant is a definitive treatment for many liver diseases, it’s a major operation and requires a certain level of health to be successful.  If a patient isn’t healthy enough for a transplant, doctors don’t just send them home without any further help. Instead, they might consider other treatments or interventions that could improve the patient’s health enough to become a candidate for transplantation. This could include:  1.Medical management: This is often the first line of treatment and involves managing symptoms and complications of liver disease. It can include medications to reduce the buildup of toxins in the blood, dietary changes to improve nutritional status, and other interventions.  2.Liver dialysis: A relatively new procedure that’s similar to kidney dialysis. It helps to perform some of the functions of the liver, giving it a chance to heal and recover.  3.Bridging treatments: These are procedures like transarterial chemoembolization (TACE), radiofrequency ablation (RFA), or microwave ablation (MWA) that are used to manage liver cancer until a transplant can be done.  4.Hepatoprotective agents: Medications that aim to protect the liver and enhance its ability to regenerate.  5.Palliative care: If the disease is too advanced and the patient is not a candidate for a transplant, palliative care can help manage symptoms and improve quality of life. It’s not just about end-of-life care, but about providing relief from the symptoms and stress of the illness.  Remember that each patient is unique, and the treatment plan will be tailored to your dad’s specific condition and overall health status. It’s essential to maintain open and honest communication with his healthcare team. You can always ask them about his prognosis, potential treatments, and what you can do to help him through this difficult time. | 1 | 1 |
| 71 | Clinical Problems | 我爸爸55岁，很严重的急性乙肝，需要肝移植，如果情况不好不能移植会怎样，会让他回家等死吗 | 我非常理解您的担忧，这是一个困难的时期。然而，我需要明确一点，我是一个AI语言模型，我不能提供医学咨询或预测具体的医疗结果。但我可以给你提供一些关于急性乙肝和肝移植的一般信息。  乙型肝炎是一种由乙型肝炎病毒（HBV）引起的疾病。急性乙型肝炎通常在几周或几个月内自行消退，大多数人会完全康复。然而，有些人可能会出现严重的症状，如肝炎或肝衰竭，这可能需要住院治疗，甚至可能需要肝移植。  如果您的父亲的病情严重到需要肝移植，但由于某些原因无法进行移植手术，那么他的医生可能会寻找其他的治疗方案。这可能包括药物治疗，以控制病毒的复制并减轻症状，以及其他可能的支持性治疗，如营养支持和疼痛管理。  这并不意味着他会被让回家等死。医生和医疗团队会尽一切可能提供最佳的护理和治疗。在这个过程中，和医疗团队保持良好的沟通非常重要，以了解所有的治疗选项和可能的结果。  再次强调，具体的情况和结果取决于许多因素，包括他的整体健康状况，病情的严重程度，以及他对治疗的反应。我强烈建议您和他的医生进行深入的讨论，以获取更多具体的信息和建议。 | 首先，我非常理解您的担忧，面对父亲的严重疾病一定让您感到非常困扰。但是请记住，医学是一门不断发展的科学，总是有许多可能的治疗方法可以尝试。  急性乙肝的治疗方案通常取决于病情的严重程度。在大多数情况下，急性乙肝可以通过药物治疗以支持身体的免疫系统来对抗病毒。然而，对于严重的急性乙肝，可能需要更积极的治疗方法，如肝移植。  如果病情严重到不能进行肝移植，那么医生可能会寻找其他的治疗方案。这可能包括药物疗法，营养疗法，以及其他支持性疗法，如血液净化和生命支持。这些治疗的目标是尽可能地减少病毒的影响，提高生活质量，以及延长患者的寿命。  当然，医生会根据患者的病情和总体健康状况来决定最佳的治疗方案。请尽可能地与医生进行沟通，了解所有的可能性，并参与决策过程。  最后，请记住，尽管病情可能严重，但是"回家等死"并不是唯一的选择。医学科技的发展为我们提供了许多可能的治疗方法，而且医生和医护人员都会尽全力去帮助和照顾患者。希望这些信息能够帮助您，并祝愿您的父亲早日康复。 | 1 | 1 |
| 89 | Simulated Patients | 一个62岁的男性患者，HBeAg阳性，HBsAg 10^4 IU/mL，没有肝硬化，ALT 25 U/L，有高血压，未进行抗病毒治疗。请从肝病治疗和健康管理的角度，给他提供治疗和日常管理方面的建议。 | 这个患者是慢性乙型肝炎患者，HBeAg阳性，HBsAg水平相对较高，但ALT正常。他还有高血压病史。以下是对他的治疗和健康管理的一些建议：  1.抗病毒治疗：尽管ALT水平正常，但是由于他的年龄和HBeAg阳性，抗病毒治疗是推荐的。可以考虑使用核苷(酸)类似物，如恩替卡韦或替诺福韦，这些药物有较低的耐药性，适合长期使用。  2.高血压管理：需要定期监测血压，并采取适当的药物和生活方式干预。避免高盐、高脂肪食物，增加运动，保持良好的生活习惯。  3.定期监测：需要定期监测肝功能（包括ALT）、HBV DNA、HBeAg/抗HBe、HBsAg等指标，评估病毒复制情况和肝炎活动程度。  4.生活方式：建议保持健康的生活方式，包括适当的运动，保持良好的饮食习惯，避免饮酒和吸烟。  5.疫苗接种：建议接种肺炎疫苗和季节性流感疫苗，以防止并发症。  6.心理健康：慢性疾病可能对心理健康产生影响，建议寻求必要的心理支持。  以上建议只是基于所提供的信息，具体治疗方案应由医生根据患者的全面病情决定。 | 这位患者是一位62岁的男性，HBeAg阳性，HBsAg 10^4 IU/mL，ALT 25 U/L，没有肝硬化但有高血压的情况。首先，他的HBeAg阳性和较高的HBsAg水平意味着他有活跃的乙型肝炎病毒（HBV）感染。尽管他的ALT水平处于正常范围内，但是鉴于他的年龄和活跃的HBV感染，建议他开始接受抗病毒治疗。  药物治疗：  对于乙型肝炎的抗病毒治疗，通常推荐的第一线药物包括骨化三磷酸酶酮（Tenofovir）和恩曲他滨（Entecavir）。这些药物可以有效抑制HBV的复制，减少肝炎的活跃程度，降低肝硬化和肝癌的风险。这位患者应该与医生讨论开始使用这些药物的可能性。  健康管理：  1.饮食：保持均衡的饮食，避免过度摄入脂肪、盐和糖，以控制高血压。避免饮酒，因为酒精可以损害肝脏。  2.运动：适度的运动可以帮助控制高血压，同时也有利于整体健康。建议每周进行150分钟的中等强度运动或者75分钟的高强度运动。  3.定期检查：定期进行血压检查，以及HBV感染的监测，包括ALT、HBsAg和HBV DNA水平。这有助于及时发现病情变化，及时调整治疗方案。  4.避免传染他人：乙型肝炎是可以通过血液和体液传播的。应避免共用刀具，使用避孕套，不共用牙刷和剃须刀等。  5.药物管理：如果开始抗病毒治疗，需要每天按时服药，避免漏服。同时，需要定期随访，监测药物的效果和可能的副作用。  以上是针对这位患者的一些基本建议。具体的治疗方案应由医生根据患者的具体情况来决定。 | 1 | 2 |
| 92 | Simulated Patients | A 36-year-old female patient with HBV-DNA 10^6 IU/ml, HBeAg positive, uncertain whether she had cirrhosis, eGFR 109 ml/min, ALT 119 U/L, BMI 30.2kg/m^2, diabetes mellitus, had been treated with entecavir for 1 year. How should this patient conduct daily monitoring and lifestyle management? | This patient is dealing with chronic hepatitis B infection and other comorbidities, including obesity and diabetes mellitus. Here are some general suggestions for daily monitoring and lifestyle management:  1. Regular Monitoring:  (1)Hepatitis B: Regular monitoring with liver function tests, HBV DNA, and HBeAg every 3-6 months is recommended. The patient should also be screened for hepatocellular carcinoma (HCC) every 6 months using ultrasound, especially if cirrhosis is confirmed.  (2)Diabetes Mellitus: Regular monitoring of blood glucose levels is crucial. HbA1c tests should be done every 3 months to assess the long-term control of blood glucose.  (3)Obesity: Monitor weight and BMI regularly. Consider a body composition analysis if available.  2. Medication:  (1)Hepatitis B: The patient should continue taking entecavir as prescribed by the doctor. Adherence to antiviral therapy is crucial for controlling viral replication.  (2)Diabetes Mellitus: Regular use of antidiabetic medications as prescribed. Insulin therapy might be required depending on the severity of diabetes.  3. Diet:  (1)Hepatitis B: A balanced diet is important. Try to avoid alcohol and limit foods that are high in fat and sugar. Drink plenty of fluids.  (2)Diabetes Mellitus: The patient should follow a diabetes-friendly diet, rich in fiber, and low in simple carbohydrates and unhealthy fats.  (3)Obesity: A low-calorie diet can be considered under the supervision of a dietitian.  4. Exercise:  Regular physical activity is beneficial for all three conditions. Aim for at least 150 minutes of moderate-intensity or 75 minutes of high-intensity exercise per week.  5. Mental Health:  Chronic diseases can lead to stress and mental health issues. Regular mental health check-ups, stress management, and relaxation techniques can be beneficial.  6. Regular Check-ups:  Regular follow-ups with the healthcare provider to monitor the progress of the disease and adjust treatments as necessary.  Remember, these are general recommendations and the patient’s individual plan may vary based on her specific circumstances and overall health. Always consult with healthcare professionals for personalized advice. | The patient should follow a comprehensive approach for the management of her chronic hepatitis B virus (HBV) infection, along with her diabetes mellitus and obesity. Here are some recommendations:  1. Regular Monitoring:  (1)Hepatitis B: The patient should continue to have regular follow-up with her hepatologist or infectious disease specialist. This typically involves blood tests every 3-6 months to monitor HBV-DNA levels, HBeAg status, and liver function tests (including ALT). The patient may also need regular imaging studies (like ultrasound) to monitor for signs of liver damage or cirrhosis.  (2)Diabetes Mellitus: The patient should monitor her blood glucose levels regularly as advised by her healthcare provider. HbA1c levels should be checked every 3-6 months to monitor long-term blood glucose control. Regular follow-up with her endocrinologist or primary care physician is essential.  (3)Obesity: The patient should monitor her weight regularly and aim for a gradual weight loss (if advised by her healthcare provider). Her BMI should be calculated at each visit.  (4)Kidney function: As the patient’s eGFR is currently normal, regular monitoring of kidney function is recommended, especially given her use of antiviral medication and presence of diabetes.  2. Lifestyle Management:  (1)Diet: The patient should follow a balanced diet that is low in saturated fats, sugar, and salt. She should consume a diet rich in fruits, vegetables, lean proteins, and whole grains. Given her diabetes, she should work with a dietician to create a meal plan that helps control her blood sugar levels.  (2)Exercise: Regular physical activity is important. She should aim for at least 150 minutes of moderate-intensity or 75 minutes of vigorous-intensity aerobic physical activity per week, along with muscle-strengthening activities on 2 or more days a week.  (3)Alcohol and Smoking: The patient should avoid alcohol and smoking, as they can exacerbate liver damage and negatively affect diabetes control.  (4)Medication Adherence: It’s crucial that the patient adheres to her prescribed medications, including entecavir for HBV and any medications she may be on for diabetes.  (5)Vaccinations: The patient should receive all recommended vaccinations, including the annual flu vaccine and the pneumococcal vaccine, given her chronic medical conditions.  (6)Mental Health: Chronic diseases can often lead to stress, anxiety, and depression. Regular mental health check-ups should be incorporated into her healthcare routine.  It’s important that the patient has regular follow-ups with her healthcare team and that any changes in her condition are communicated promptly. Any changes in her symptoms, such as jaundice, abdominal pain, persistent nausea/vomiting, or worsening fatigue, should be reported immediately to her healthcare provider. | 1 | 1 |
| 92 | Simulated Patients | 一个36岁的女性患者，HBV-DNA 10^6 IU/ml，HBeAg阳性，不确定自己是否有肝硬化，eGFR 109 ml/min，ALT 119 U/L，BMI 30.2kg/m^2，有糖尿病，已经服用恩替卡韦治疗1年。这位患者应当如何进行日常监测与生活方式管理？ | 这位患者的情况显示她有慢性乙型肝炎（HBV），并且可能存在肝硬化的风险。她的HBV-DNA水平很高，这可能表示病毒复制活跃，肝脏疾病可能正在进展。此外，她的ALT（丙氨酸氨基转移酶）水平超过正常值，这可能表示肝脏受损。她的eGFR（肾小球滤过率）在正常范围内，表示肾功能良好。然而，她的BMI（体重指数）显示她的体重超重，可能存在肥胖，并且已经有糖尿病。这可能会加重她的肝病和其他健康问题。  对于这位患者的日常监测和生活方式管理，以下是一些建议：  日常监测：  1.HBV-DNA: 每3-6个月进行一次检测。如果病毒载量持续升高，可能需要调整治疗方案。  2.ALT: 每3-6个月进行一次检测。如果ALT水平持续升高，可能表示肝病进展，需要进一步的检查和治疗。  3.肝硬度检测: 建议定期进行肝硬度检测（如声波弹性成像）以评估是否有肝硬化的迹象。  4.肾功能检测: 继续监测eGFR，以确保肾功能良好。特别是在使用恩替卡韦等抗病毒药物时，需要定期检查肾功能。  5.血糖和血压: 由于糖尿病，需要定期监测血糖和血压，确保其控制在合理范围内。  生活方式管理：  1.饮食: 建议低盐、低脂、高蛋白质的饮食。尽量避免饮酒和吃油腻、辛辣食物。增加新鲜蔬菜和水果的摄入。  2.体重管理: 由于BMI超标，需要进行体重管理。可以通过合理的饮食和定期运动来控制体重。  3.运动: 建议定期运动，如散步、瑜伽或游泳，以保持健康的体重和改善血糖控制。  4.服药: 坚持每日服用恩替卡韦，不要随意停药或改变剂量。定期向医生报告药物的副作用。  5.定期随访: 与医生保持密切的联系，定期进行随访，及时报告任何新的症状或不适。  这些都是一般性的建议，具体的监测频率和生活方式管理还需要根据患者的个体情况和医生的建议进行调整。 | 对于这位患者，我们需要考虑她的慢性乙型肝炎，可能的肝硬化，糖尿病，以及肥胖症的综合管理。下面是一些关于日常监测和生活方式管理的建议：  1.药物治疗和监测：恩替卡韦是一种抗病毒药物，用于治疗乙型肝炎。患者应继续按照医生的建议服用，并定期监测肝功能和HBV-DNA水平，以评估病毒的活动和药物的效果。一般情况下，这些检查应该每3-6个月进行一次。  2.肝硬化的评估：虽然患者没有明确的肝硬化病史，但是乙型肝炎的患者有发展为肝硬化的风险。因此，她需要定期进行肝脏超声检查，以便及时发现可能的肝硬化或肝癌。这些检查通常每6-12个月进行一次。  3.糖尿病管理：患者应定期监测血糖和HbA1c水平，以评估糖尿病的控制情况。同时，她应该遵循一个低糖、低盐、低脂肪的饮食，并进行适量的运动，以帮助控制血糖和体重。  4.体重管理：BMI 30.2kg/m^2表明患者处于肥胖状态，这可能会加重糖尿病和肝病的病情。因此，患者需要进行体重管理，包括合理饮食和运动。她应该寻求营养师的帮助，制定一个个性化的饮食计划。  5.生活方式：患者应该避免饮酒和吸烟，因为这些都可能加重肝病和糖尿病的病情。同时，她应该保持充足的休息和良好的心理状态，以帮助身体应对这些慢性疾病。  6.定期体检：患者应定期进行全面的体检，包括血压、血脂、心脏等方面的检查，以便及时发现和处理可能的并发症。  以上建议仅供参考，具体的治疗和管理方案应由患者的主治医生根据她的具体情况制定。 | 2 | 1 |
| 94 | Simulated Patients | A 50-year-old male patient, HBeAg positive, ALT 231 U/L, with hyperlipidemia and currently not on antiviral therapy, came to console whether interferon could be used. Please give medical advice. | The patient in question is HBeAg positive, which indicates active viral replication of hepatitis B, and has an elevated ALT level (Alanine Aminotransferase, a liver enzyme), suggesting liver inflammation or damage. This usually necessitates antiviral treatment.  Interferon is one of the treatment options for chronic hepatitis B. It’s an immune modulator that enhances the body’s immune response to the virus, aiming to achieve sustained viral suppression even after treatment cessation. Interferon can be used in certain patients with chronic hepatitis B infection, but it’s not suitable for everyone due to its side effects and contraindications.  However, the patient also has hyperlipidemia, a condition characterized by high levels of fats (lipids) in the blood. Interferon therapy is not directly contraindicated in patients with hyperlipidemia, but it can cause a wide range of side effects including flu-like symptoms, mood disturbances, thyroid dysfunction, and, importantly, it may exacerbate existing conditions.  Before deciding on interferon therapy, several factors should be considered:  1.The patient’s overall health status: If the patient has significant comorbidities, interferon might not be the best choice due to its potential side effects.  2.The patient’s lifestyle and personal preferences: Interferon treatment involves regular injections, usually for a period of 48 weeks. This can have a significant impact on the patient’s quality of life.  3.The patient’s viral load and liver disease stage: Interferon is generally more effective in patients with lower viral loads and less advanced liver disease.  4.Potential for drug interactions: If the patient is taking medication for hyperlipidemia, potential interactions with interferon should be evaluated.  In conclusion, while interferon could potentially be used in this patient, it’s crucial to discuss the potential risks and benefits with the patient, considering his overall health status, lifestyle, and personal preferences. Moreover, monitoring the patient’s condition closely during treatment would be essential.  As an AI language model, I can provide general advice, but the final decision should be made by a healthcare professional based on a comprehensive evaluation of the patient’s condition. | Based on the information provided, the patient is a 50-year-old male with HBeAg-positive chronic hepatitis B, elevated ALT levels (231 U/L), and hyperlipidemia.  Interferon is one of the treatment options for chronic hepatitis B. It works by boosting the immune system to fight the virus. It’s often considered as a treatment option due to its finite treatment duration and lack of resistance. However, the use of interferon is limited by its side effects and contraindications. Side effects can include flu-like symptoms, depression, and a drop in white blood cells.  As for contraindications, interferon is generally not recommended for patients with:  1.Decompensated cirrhosis.  2.A history of severe psychiatric disease, especially depression.  3.Autoimmune disease.  4.Uncontrolled thyroid disease.  5.Heart disease.  6.Seizure disorders.  In this case, the patient’s hyperlipidemia would not necessarily preclude the use of interferon, but the patient’s overall health status, including liver disease stage, cardiovascular risk, and mental health, should be thoroughly evaluated.  Also, it’s important to note that the patient’s elevated ALT levels may indicate active liver inflammation or damage. This should be further investigated to understand the extent of liver disease.  Before making any treatment decisions, further tests should be performed including:  1.Hepatitis B virus DNA level  2.Liver ultrasound  3.Complete blood count  4.Coagulation parameters  5.Tests to rule out co-infection with hepatitis C, hepatitis D, and HIV  6.Possibly a liver biopsy  The patient should also be counseled on lifestyle modifications including a healthy diet, regular exercise, avoidance of alcohol, and regular monitoring.  Remember that the ultimate decision on treatment type and timing should be individualized, taking into account the patient’s age, disease phase, viral replication, ALT level, presence of liver inflammation or fibrosis, family history of hepatocellular carcinoma, patient’s willingness, and possible side effects.  Please consult with a hepatologist or an infectious disease specialist for a comprehensive evaluation and personalized treatment plan.  Disclaimer: This advice is intended to be general in nature, and specific causes may not apply to individual cases. It should not replace consultation with a healthcare provider. | 2 | 1 |
