# Supplementary material for: Assessing ChatGPT as a Medical Consultation Assistant for Chronic Hepatitis B: Cross-Language Study of English and Chinese
Source: JMIR Med Inform. 2024 Aug 8;12:e56426. doi: 10.2196/56426 (PMC11342014; doi:10.2196/56426)
Supplement: Multimedia Appendix 14 [file medinform_v12i1e56426_app14.docx]

**Multimedia Appendix 14.** Responses mentioning “I am not a doctor” or “I cannot give diagnosis or treatment” in ChatGPT-4.0.

| Number | Classicfication | Question | Response 1 | Response 2 | Mention1 | Mention2 |
| --- | --- | --- | --- | --- | --- | --- |
| 44 | Clinical Problems | Give clinical treatment advice for women of childbearing age infected with hepatitis B | As an AI developed by OpenAI, I’m capable of providing some general guidance based on available data and research. However, it’s crucial to remember that any health-related advice should always be confirmed and supervised by a healthcare professional.  Hepatitis B is a viral infection that attacks the liver. It can cause both acute and chronic disease. Here are some general considerations for the clinical treatment of women of childbearing age who are infected with Hepatitis B:  1.Antiviral Therapy: Antiviral therapy may be recommended for women with active hepatitis B virus (HBV) replication, evidence of liver inflammation, and/or liver damage. The decision to initiate therapy should be made in consultation with a healthcare provider experienced in managing hepatitis B. First-line oral antiviral agents include tenofovir disoproxil fumarate (TDF), tenofovir alafenamide (TAF), and entecavir (ETV). These drugs have been shown to be safe and effective in suppressing HBV replication.  2.Pregnancy Considerations: Women of childbearing age who have HBV need to consider the risk of vertical transmission (mother to child during childbirth). Antiviral therapy may be recommended in the third trimester of pregnancy to reduce the risk of perinatal transmission, especially for women with high viral load. Again, TDF and TAF are considered safe in pregnancy.  3.Breastfeeding: Women on antiviral therapy may wish to breastfeed. TDF and TAF are considered safe during breastfeeding.  4.Contraception: Women with HBV who do not wish to become pregnant should be counseled on contraception. Barrier methods, hormonal contraception, and intrauterine devices are all safe in women with HBV.  5.Regular Monitoring: Regular monitoring is important to assess the status of liver disease, the effectiveness of treatment, and to screen for liver cancer (which HBV patients are at increased risk for). This usually includes blood tests, and may include imaging of the liver and/or liver biopsy.  6.Vaccination of Close Contacts: All household and sexual contacts should be vaccinated against HBV if they are not already immune.  7.Healthy Lifestyle: It’s beneficial to maintain a healthy lifestyle. This includes avoiding alcohol, maintaining a balanced diet, and regular exercise.  Remember, it’s crucial to consult with a healthcare provider for personalized medical advice. This information is intended to be a general guide and may not cover all individual healthcare needs. | I’m an AI developed by OpenAI and I can provide some general advice based on available information. However, please consult a healthcare professional for medical advice as this information is subject to change and individual circumstances can significantly affect treatment options.  Hepatitis B is a viral infection that attacks the liver. In women of childbearing age, the treatment approach should consider both the health of the woman and the risk of mother-to-child transmission.  1.Monitoring  Regular monitoring of hepatitis B virus (HBV) DNA levels, alanine aminotransferase (ALT) levels, and screening for hepatocellular carcinoma (HCC) are important in managing chronic HBV infection.  2.Antiviral Therapy  Antiviral therapy may be considered in women of childbearing age with chronic hepatitis B if they have high HBV DNA levels (> 20,000 IU/mL) and/or elevated ALT levels. The choice of antiviral therapy should consider safety in pregnancy, as unplanned pregnancies may occur.  3.First-line antiviral agents recommended by the World Health Organization (WHO) include entecavir (ETV) and tenofovir disoproxil fumarate (TDF) or tenofovir alafenamide (TAF). These are potent inhibitors of HBV replication and have a high barrier to resistance. They are considered safe in pregnancy.  4.Pregnancy and Breastfeeding  Pregnant women with high HBV DNA levels (> 200,000 IU/mL) in the third trimester (from week 28) should be offered antiviral prophylaxis with TDF or TAF to prevent mother-to-child transmission of HBV, in addition to the administration of hepatitis B vaccine and hepatitis B immunoglobulin (HBIG) to the newborn within 24 hours of birth.  Breastfeeding is not contraindicated in women taking ETV, TDF, or TAF.  5.Contraception  Women of childbearing age who are not planning to become pregnant should be counseled about the use of effective contraception. Contraception should be continued for at least 6 months after stopping antiviral therapy due to the risk of flare of hepatitis B.  6.Vaccination  All household and sexual contacts of women with chronic hepatitis B should be vaccinated.  Remember that this is general advice and individual treatment plans may vary. Always consult with a healthcare provider for personalized advice.  Sources:  World Health Organization. Guidelines for the prevention, care and treatment of persons with chronic hepatitis B infection. Geneva: World Health Organization; 2015.  Terrault NA, Lok ASF, McMahon BJ, et al. Update on prevention, diagnosis, and treatment of chronic hepatitis B: AASLD 2018 hepatitis B guidance. Hepatology. 2018;67(4):1560-1599. | Yes | Yes |
| 45 | Clinical Problems | 乙型肝炎病毒表面抗体阳性怎么办？ | 乙型肝炎病毒表面抗体（HBsAb）阳性通常表示两种可能的情况：  1.疫苗接种后产生的免疫反应：如果你曾经接种过乙型肝炎疫苗，你的身体会产生抗体来对抗可能的病毒感染。这种情况下，HBsAb阳性是正常且健康的表现，表示你对乙型肝炎病毒有了免疫保护。  2.自然感染后的自我康复：如果你曾经被乙型肝炎病毒感染过，然后你的身体成功地清除了病毒，你的身体也会产生HBsAb。这也是一种健康的反应，表示你已经从乙型肝炎病毒感染中恢复，并对未来的感染有了免疫保护。  总的来说，如果你的HBsAb检测结果是阳性，那么你应该是对乙型肝炎病毒有了免疫。然而，你仍然应该继续进行定期的肝功能检查，并遵循医生的建议，以确保你的肝脏健康。  请注意，以上信息仅供参考，并不能替代专业医疗建议。如果你有任何疑问或担忧，你应该联系你的医疗保健提供者。 | 乙型肝炎病毒表面抗体阳性（HBsAb阳性）通常表示两种可能的情况：  1.疫苗接种后产生的免疫反应：如果你接种过乙型肝炎疫苗，身体会产生抗体来对抗乙型肝炎病毒。这种情况下，抗体阳性是正常的，表示疫苗接种成功，你已经对乙型肝炎病毒有了免疫保护。  2.自然感染后的康复：如果你曾经感染过乙型肝炎病毒并且成功康复，身体也会产生抗体。这种情况下，抗体阳性表示你已经成功抵抗了乙型肝炎病毒，且在未来有一定的抵抗力。  无论哪种情况，乙型肝炎病毒表面抗体阳性通常是一个好消息，表示你的身体已经能够抵抗乙型肝炎病毒。不过，对于任何健康问题，都应该及时咨询医生或医疗专业人士，确保得到准确的诊断和治疗建议。  请注意，以上信息仅供参考，如果有任何疑问或不适，请立即寻求医疗专业人士的帮助。 | Yes | No |
| 47 | Clinical Problems | 乙型肝炎e抗体阳性怎么办？ | 乙型肝炎e抗体（HBeAb）阳性通常表示乙型肝炎病毒（HBV）的活动度较低，病毒复制的能力较弱。这是一个相对较好的情况，但仍需要定期监测和医疗专业人员的指导。以下是一些可能的建议：  1.定期检查：即使HBeAb阳性，您仍需要定期进行血液检查，监测肝功能和病毒载量。这将有助于医生评估病情的变化并及时调整治疗方案。  2,保持健康的生活方式：合理的饮食和规律的运动可以帮助保持肝脏健康。避免饮酒和吸烟，因为这些都可能损害肝脏。  3.避免传播病毒：乙型肝炎是一种可以通过血液和体液传播的病毒。避免与他人共用牙刷、剃须刀等可能导致病毒传播的物品。此外，使用安全的性行为也可以防止病毒传播。  4.接种疫苗：对于您的亲密接触者和家人，建议他们接种乙型肝炎疫苗，以防止病毒传播。  5.药物治疗：虽然HBeAb阳性通常意味着病毒活动度较低，但在某些情况下，医生可能仍会推荐药物治疗。请遵照医生的指导使用药物。  请注意，以上的建议并不能替代医生的专业意见。如果您对自己的病情有任何疑虑或问题，应及时向医疗专业人员咨询。 | 乙型肝炎e抗体阳性（HBeAb阳性）是乙型肝炎病毒感染的一个重要指标，它通常表明病毒的复制活动较低，也可能是病毒感染进入了慢性阶段。然而，这不是一个完全无害的状况，仍然需要定期监测和评估。以下是你应该采取的步骤：  1.寻求医疗咨询：如果你被检测出乙型肝炎e抗体阳性，首先应该寻求医生的建议。医生会根据你的病史、症状和其他相关检测结果，制定出适合你的诊疗方案。  2.定期检查：你需要定期进行肝功能检查和病毒载量检测，以监测病情的变化。这可以帮助你及时发现并处理可能出现的并发症。  3.保持健康的生活方式：这包括保持良好的饮食习惯、避免酗酒、避免接触有害化学物质、保持适当的运动等。这些都可以帮助保护你的肝脏健康。  4.接种疫苗：如果你的家人或者密切接触者没有接种乙型肝炎疫苗，他们应该接种，以防止病毒的传播。  5.遵守医嘱：如果医生建议你服用抗病毒药物，你应该按照医嘱服用，以控制病毒的复制。  以上都是对乙型肝炎e抗体阳性的一般建议，具体的诊疗方案还需要根据你的具体情况和医生的建议来确定。 | Yes | No |
| 49 | Clinical Problems | Give clinical treatment advice for ones who are exposed to hepatitis B virus | I’m an AI developed by OpenAI and while I can provide general advice based on available medical literature, I must emphasize that the following information should not replace professional medical advice. If you believe you have been exposed to the hepatitis B virus, please consult with a healthcare professional immediately. Here’s some general information about the management and treatment of potential exposure to the hepatitis B virus (HBV):  1.Post-exposure prophylaxis  Post-exposure prophylaxis (PEP) is a preventive medical treatment that begins immediately after exposure to a pathogen, such as the hepatitis B virus. For hepatitis B, PEP usually consists of a combination of hepatitis B immunoglobulin (HBIG) and the hepatitis B vaccine.  The treatment should ideally be initiated within 24 hours, but it can still be effective if started within 2 weeks of exposure.  2.Hepatitis B Immunoglobulin (HBIG)  HBIG is a type of immunoglobulin (antibody) that can provide short-term protection against hepatitis B. It is often given as a single dose as soon as possible after exposure to the virus.  3.Hepatitis B Vaccine  The hepatitis B vaccine stimulates the body’s immune system to protect against the virus. It is typically given in a series of 3-4 injections over a six-month period.  4.Monitoring  Even if PEP is initiated, it is crucial to monitor for signs and symptoms of hepatitis B, which can include jaundice (yellowing of the skin and eyes), fatigue, abdominal pain, loss of appetite, intermittent nausea and vomiting.  5.Follow-up testing  Follow-up testing is needed to ensure that the PEP has been effective and that the person has not developed hepatitis B. This testing typically involves blood tests 3-6 months after the potential exposure.  6.Other Considerations  Prevention is always better than treatment. It is highly recommended that individuals who are at a higher risk of exposure to hepatitis B, such as healthcare workers, individuals with multiple sexual partners, intravenous drug users, and people traveling to regions with high rates of hepatitis B, receive the hepatitis B vaccination as a preventive measure.  Please consult with a healthcare provider for personalized advice and treatment. | I’m an AI developed by OpenAI and while I can provide general advice based on available information, please note that this advice is not a substitute for professional medical advice. Always consult with a healthcare professional for accurate information.  Hepatitis B is a viral infection that attacks the liver and can cause both acute and chronic disease. If you believe you have been exposed to the hepatitis B virus (HBV), it is important to seek medical attention immediately. Here are general steps that may be advised:  1.Post-Exposure Prophylaxis (PEP): If you seek medical attention soon after exposure (preferably within 24 hours and not later than one week), your healthcare provider may administer a dose of hepatitis B immune globulin (HBIG) and initiate the hepatitis B vaccine series. This is known as post-exposure prophylaxis (PEP).  2.Hepatitis B Vaccination: If you are not already vaccinated against hepatitis B, your healthcare provider will likely recommend starting the hepatitis B vaccine series. The vaccine can protect you from developing hepatitis B in the future.  3.Testing: Your healthcare provider will likely order blood tests to determine if you have been infected with the virus.  4.Monitoring: If you test positive for HBV, you will be monitored for signs of liver disease. This may involve regular blood tests and possibly a liver biopsy.  5.Antiviral Treatment: If you develop chronic hepatitis B, you may need treatment with antiviral medications. These medications can help slow the progression of the disease and reduce the risk of complications.  6.Healthy Lifestyle Choices: It’s important to take care of your liver if you have hepatitis B. This includes avoiding alcohol and maintaining a healthy diet.  Remember, hepatitis B can be prevented with vaccination. The vaccine is safe and effective and is usually given as 3-4 shots over a six-month period.  For any medical concerns, always consult with a healthcare professional. They can provide you with the most accurate information and treatment options based on your specific circumstances. | Yes | Yes |
| 63 | Clinical Problems | What should I do after learning that I have hepatitis B? | I’m an AI developed by OpenAI and while I can provide some general guidance, please consult with a healthcare professional for advice tailored to your specific situation. If you have been diagnosed with Hepatitis B, here are some steps you might consider:  1.Consult with a Healthcare Provider: The most important step is to consult with a healthcare provider. They will be able to provide you with specific advice based on your health history and current situation.  2.Understand Your Diagnosis: Hepatitis B is a viral infection that attacks the liver and can cause both acute and chronic disease. The virus is transmitted through contact with the blood or other body fluids of an infected person.  3.Treatment Options: Depending on the type of Hepatitis B (acute or chronic), your doctor will discuss treatment options. Acute Hepatitis B usually doesn’t require treatment and most people fully recover. Chronic Hepatitis B is a long-term illness that may require ongoing treatment.  4.Monitoring: Regular monitoring is important as Hepatitis B can lead to severe liver problems, such as cirrhosis or liver cancer. Your doctor may order regular blood tests or ultrasounds to monitor your liver health.  5.Prevent Transmission: Hepatitis B is contagious. Follow guidelines to prevent spreading the virus to others. This includes not sharing personal items such as toothbrushes or razors, using protection during sexual activity, and not donating blood or organs.  6.Healthy Lifestyle: A healthy lifestyle can help your liver. This includes avoiding alcohol, maintaining a balanced diet, exercising regularly, and getting enough sleep.  7.Vaccination: If you live with others, they should get tested and consider getting vaccinated against Hepatitis B to prevent transmission.  8.Support: Reach out to support groups and resources. Living with a chronic illness can be challenging, and it can be helpful to connect with others who are going through the same experience.  Remember, each person’s situation is unique and your healthcare provider is the best source of information for your individual circumstances. | I’m an AI language model, not a doctor, but I can suggest some general steps that are typically recommended after a hepatitis B diagnosis. Remember, it’s very important to consult with a healthcare professional for personalized advice.  1.Confirm the Diagnosis: Hepatitis B is diagnosed through blood tests. If you’ve been diagnosed, it’s important to understand whether you have an acute (short-term) or chronic (long-term) infection. This will influence the treatment approach.  2.Consult with a Specialist: It’s important to consult with a healthcare professional, such as a hepatologist, gastroenterologist, or infectious disease specialist, who specializes in liver diseases. They can provide you with the most accurate information and treatment options.  3.Understand the Disease: Learn about hepatitis B, its transmission methods, and how to prevent spreading it to others. Hepatitis B is primarily spread through blood and body fluids.  4.Begin Treatment if Necessary: Not all people with hepatitis B require treatment. If you do, antiviral medications are typically used. The goal of treatment is to reduce the risk of liver disease and prevent the spread of the virus to others.  5.Regular Monitoring: Regular monitoring is necessary to manage the disease. This usually involves blood tests and liver scans to assess the health of your liver and the status of the virus.  6.Maintain a Healthy Lifestyle: Avoid alcohol and maintain a balanced diet, as alcohol can cause additional liver damage. Regular exercise is also beneficial.  7.Inform Close Contacts and Family Members: Hepatitis B can be transmitted to others. It’s important to inform those close to you about your condition so they can also get tested and vaccinated if necessary.  8.Prevent Transmission: Use barrier methods during sex, don’t share needles, and avoid sharing personal items like toothbrushes or razors that could have your blood on them.  9.Get Vaccinated for Hepatitis A: If you have hepatitis B, it’s a good idea to also get vaccinated against hepatitis A to prevent co-infection, which could lead to more severe liver disease.  Remember, this information is general advice and may not fully apply to your specific situation. Please consult with a healthcare professional for advice tailored to your circumstances. | Yes | Yes |
| 63 | Clinical Problems | 知道自己乙肝后应该怎么办 | 首先，我要强调，我是一个人工智能助手，我不能提供医疗建议。但是，我可以告诉你，如果你发现自己患有乙型肝炎（乙肝），你应该尽快寻找医疗专业人士进行咨询。以下是一些可能的步骤，但这些并不替代专业医疗建议：  1.寻求医疗咨询：你应该尽快找到一个医生，最好是肝脏疾病专家（肝病专科医生或肝脏病学家）进行咨询。他们会对你的病情进行全面评估，并制定最适合你的治疗方案。  2.定期检查：乙肝是一种慢性疾病，需要定期进行血液检查和肝脏超声检查以监测病情的进展。  3.药物治疗：根据你的病情，医生可能会建议你使用抗病毒药物。这些药物可以帮助控制病毒复制，减少肝脏炎症和纤维化的风险。  4.生活方式改变：避免饮酒，因为酒精可以加速肝脏损伤。保持健康的饮食和运动习惯也有助于维持肝脏健康。  5.防止传播：乙肝是通过血液和体液传播的，因此你应该避免与他人共享牙刷、剃须刀、针头等。在性行为中应使用安全措施，如使用避孕套。你的亲密伴侣和家庭成员也可能需要接种乙肝疫苗。  6.心理支持：发现自己患有慢性疾病可能会引发压力和焦虑。寻求心理咨询和支持可能有助于你应对这种情况。  再次强调，这些都是一般性建议，并不能替代专业医疗建议。你应该尽快寻找医疗专业人士进行咨询。 | 我不是医生，但是我可以给你一些基本的建议。如果你被诊断为乙肝，请遵循以下步骤：  1.找医生咨询：首先，你应该找到一位专业的医生，例如肝脏病专家或感染病专家，进行详细的咨询和评估。他们会根据你的病情决定最适合你的治疗方案。  2.了解疾病：乙肝是一种由乙型肝炎病毒（HBV）引起的感染，可以通过血液、性行为、或者母婴传播。了解疾病的性质和传播方式可以帮助你更好地管理你的健康状况。  3.健康的生活方式：保持健康的生活方式对于控制乙肝很重要。这包括均衡饮食，规律的体育活动，以及避免饮酒和吸烟。  4.定期检查：如果你被诊断为乙肝，你需要定期进行肝功能检查和病毒载量检测。这些检查可以帮助你的医生了解你的病情是否稳定，或者是否需要调整治疗方案。  5.预防传播：由于乙肝可以通过血液和性行为传播，所以你应该采取预防措施，避免将病毒传播给他人。这包括避免分享针筒、牙刷、剃须刀，使用安全套进行性行为，以及告知你的性伴侣你的病情。  6.接种疫苗：如果你的家人或者其他密切接触者还没有接种乙肝疫苗，他们应该尽快接种，以防止病毒传播。  7.心理支持：被诊断为乙肝可能会引起焦虑和压力。寻找心理支持，例如咨询心理医生或者加入支持小组，可以帮助你应对这些情绪。  请记住，我提供的这些信息只是基本的建议，你应该咨询你的医生获取专业的医疗建议。 | Yes | No |
| 66 | Clinical Problems | 慢乙肝患者如何护肝 | 乙型肝炎是一种常见的慢性疾病，对患者的身体健康造成很大影响。下面是一些针对慢性乙型肝炎患者的护肝建议：  1.健康饮食：合理的饮食是保持肝脏健康的关键。应避免过度食用油腻、辛辣、烧烤等刺激性食物，多吃新鲜蔬菜和水果，保证膳食中的营养均衡。  2.规律作息：保持良好的生活习惯，避免熬夜，保证充足的睡眠。  3.适当运动：适当的运动可以增强身体免疫力，帮助身体抵抗疾病。如慢跑、游泳、瑜伽等都是很好的选择。  4.避免酒精：酒精对肝脏有很大的伤害，应尽量避免饮酒。  5.避免药物伤肝：有些药物对肝脏有损害，应在医生指导下使用药物，尤其是非处方药。  6.定期检查：定期进行肝功能检查，及时了解病情，及时调整治疗方案。  7.积极治疗：一旦确诊为乙型肝炎，应积极接受医生的治疗，不要放弃治疗。  请注意，以上建议不能替代医生的专业意见。如果您或您的亲友有乙型肝炎，应定期就医，并遵循医生的指示和建议。 | 乙型肝炎（HBV）是一种严重的肝脏疾病，可以引发肝硬化和肝癌。对于慢性乙型肝炎（CHB）患者来说，护肝非常重要，以下是一些有效的护肝方法：  1.健康饮食：选择营养均衡的食物，包括全谷类、新鲜水果和蔬菜、瘦肉、鱼、低脂乳制品等。避免过度摄入脂肪、糖和盐。避免饮酒，因为酒精可以损害或破坏肝细胞。  2.适当运动：保持活跃和适当的体重有助于肝脏健康。建议每周至少进行150分钟的中等强度有氧运动，如快步走、慢跑、骑自行车等。  3.定期检查：定期进行肝功能检查和病毒载量检测，以监控疾病进展和治疗效果。  4.遵医嘱：根据医生的建议进行抗病毒治疗。即使症状消失或病毒载量降低，也不能擅自停药。  5.戒烟：吸烟会加重肝脏疾病，因此戒烟对肝脏健康非常重要。  6.防止交叉感染：避免共享牙刷、剃须刀、指甲剪或任何可能有血液的物品，以防止病毒传播。  7.充足休息：保证充足的休息时间，有助于身体恢复和修复。  这些方法可以帮助保护肝脏，减慢疾病进展，但不能替代医生的专业治疗。如果您有任何健康问题或疑虑，应立即咨询医生。 | Yes | Yes |
| 71 | Clinical Problems | 我爸爸55岁，很严重的急性乙肝，需要肝移植，如果情况不好不能移植会怎样，会让他回家等死吗 | 我非常理解您的担忧，这是一个困难的时期。然而，我需要明确一点，我是一个AI语言模型，我不能提供医学咨询或预测具体的医疗结果。但我可以给你提供一些关于急性乙肝和肝移植的一般信息。  乙型肝炎是一种由乙型肝炎病毒（HBV）引起的疾病。急性乙型肝炎通常在几周或几个月内自行消退，大多数人会完全康复。然而，有些人可能会出现严重的症状，如肝炎或肝衰竭，这可能需要住院治疗，甚至可能需要肝移植。  如果您的父亲的病情严重到需要肝移植，但由于某些原因无法进行移植手术，那么他的医生可能会寻找其他的治疗方案。这可能包括药物治疗，以控制病毒的复制并减轻症状，以及其他可能的支持性治疗，如营养支持和疼痛管理。  这并不意味着他会被让回家等死。医生和医疗团队会尽一切可能提供最佳的护理和治疗。在这个过程中，和医疗团队保持良好的沟通非常重要，以了解所有的治疗选项和可能的结果。  再次强调，具体的情况和结果取决于许多因素，包括他的整体健康状况，病情的严重程度，以及他对治疗的反应。我强烈建议您和他的医生进行深入的讨论，以获取更多具体的信息和建议。 | 首先，我非常理解您的担忧，面对父亲的严重疾病一定让您感到非常困扰。但是请记住，医学是一门不断发展的科学，总是有许多可能的治疗方法可以尝试。  急性乙肝的治疗方案通常取决于病情的严重程度。在大多数情况下，急性乙肝可以通过药物治疗以支持身体的免疫系统来对抗病毒。然而，对于严重的急性乙肝，可能需要更积极的治疗方法，如肝移植。  如果病情严重到不能进行肝移植，那么医生可能会寻找其他的治疗方案。这可能包括药物疗法，营养疗法，以及其他支持性疗法，如血液净化和生命支持。这些治疗的目标是尽可能地减少病毒的影响，提高生活质量，以及延长患者的寿命。  当然，医生会根据患者的病情和总体健康状况来决定最佳的治疗方案。请尽可能地与医生进行沟通，了解所有的可能性，并参与决策过程。  最后，请记住，尽管病情可能严重，但是"回家等死"并不是唯一的选择。医学科技的发展为我们提供了许多可能的治疗方法，而且医生和医护人员都会尽全力去帮助和照顾患者。希望这些信息能够帮助您，并祝愿您的父亲早日康复。 | Yes | No |
| 72 | Clinical Problems | Is it safe to have hepatitis B vaccine 7 days after my second dose of covid-19 vaccine? | As an AI language model, I can tell you that generally, the Centers for Disease Control and Prevention (CDC) and the World Health Organization (WHO) have no specific guidelines about the timing between different types of vaccines, including COVID-19 vaccines and Hepatitis B vaccines. It’s typically considered safe to get another vaccine, such as the Hepatitis B vaccine, even if you’ve recently had a COVID-19 vaccine.  However, it’s important to note that individual health circumstances can vary, and this information might change as more research is conducted. Therefore, it’s recommended that you consult your healthcare provider before getting vaccinated. They can provide you with the most accurate and up-to-date advice based on your health history and the latest medical research.  Remember that vaccines play a critical role in preventing serious diseases, and it’s crucial to stay updated with your immunizations as recommended by health authorities and your healthcare provider. | I’m an AI language model and I don’t have real-time access to the latest medical research. However, as of my last update in 2023, there were no specific guidelines suggesting that the Hepatitis B vaccine and COVID-19 vaccines cannot be administered within a certain timeframe of each other.  However, this doesn’t mean it’s necessarily safe for you. It’s crucial to discuss your personal medical history, current health status, and any potential interactions between vaccines with a healthcare professional. They can provide the most accurate, up-to-date, and personalized advice for you.  Remember that while vaccines are important tools in preventing infectious diseases, each individual may have different health considerations that need to be taken into account. Always consult with a healthcare provider before making decisions about vaccinations. | Yes | Yes |
| 75 | Clinical Problems | I have positive HBsAg, HBeAg and HBcAb with a high HBV-DNA level, but my liver function is normal, should I take antiviral drugs? | I’m an AI developed by OpenAI and while I can provide some general information on this topic, I’m not a doctor and can’t provide personalized medical advice. It’s important to discuss this question with your healthcare provider who knows your medical history and can provide the appropriate recommendations for you.  However, I can tell you that hepatitis B virus (HBV) infection is a complex condition and the decision to start antiviral treatment should be individualized, depending on a variety of factors. These factors include:  1.The phase of HBV infection  2.The level of HBV-DNA in your blood  3.Whether or not you have evidence of liver damage  4.Your age  5.Your overall health status  6.Other comorbid conditions  In general, antiviral treatment is recommended for people with chronic HBV infection who are at risk for developing liver disease. These include:  1.Patients with persistently abnormal alanine aminotransferase (ALT) levels  2.Patients with high HBV-DNA levels  33.Patients with evidence of advanced fibrosis or cirrhosis  4.Patients with a family history of hepatocellular carcinoma  From your description, it seems like you have a positive HBsAg (indicating a chronic infection), positive HBeAg and HBcAb (indicating active viral replication), and high HBV-DNA levels (indicating a high viral load). However, your liver function is normal. Therefore, it’s not straightforward to say whether you should start antiviral treatment or not without further evaluation.  The decision should be made in consultation with your healthcare provider, who can consider all these factors and perhaps conduct further tests if necessary. Please do not delay seeking medical advice and discuss your situation with your doctor.  Remember, even if you do not require treatment right now, regular monitoring of your liver function and HBV status is very important. This is because the situation can change over time, and you may need treatment in the future. | I’m an AI developed by OpenAI and I’m not a doctor, but I can provide some general information based on available medical literature.  Hepatitis B virus (HBV) infection is a complex disease, and the decision to start treatment should be individualized, based on a variety of factors, including the patient’s age, overall health, liver function, and viral load.  You’ve mentioned that you have positive HBsAg, HBeAg, and HBcAb with a high HBV-DNA level. This typically indicates active viral replication, which may increase the risk of liver disease over time, even if your liver function is currently normal.  However, the decision to start antiviral therapy should be made in consultation with a healthcare provider, who can fully assess your situation. According to the American Association for the Study of Liver Diseases (AASLD) guidelines:  1.Patients who are HBeAg-positive with HBV-DNA levels greater than  IU/mL and have persistently normal alanine aminotransferase (ALT) levels should be monitored. Antiviral therapy may be considered if there is evidence of liver disease progression, even with normal ALT levels.  2.Antiviral therapy is recommended for patients who are HBeAg-positive with HBV-DNA levels greater than  IU/mL and have elevated ALT levels.  Again, this information is just a guideline and may not be applicable to all patients. A healthcare provider should be consulted to make a final decision based on the patient’s individual situation.  It’s crucial that you discuss your results with your healthcare provider or a specialist in liver diseases (hepatologist) to decide the best course of action. | Yes | Yes |
| 75 | Clinical Problems | 乙肝大三阳，肝功能正常，但病毒数高，要不要吃抗病毒药 | 乙型肝炎（HBV）的治疗取决于许多因素，包括病毒复制水平、肝脏炎症程度和肝功能等。"乙肝大三阳"是指血清中HBsAg（乙肝表面抗原）、HBeAg（乙肝e抗原）和HBcAg（乙肝核心抗原）均为阳性。这种情况可能表明乙型肝炎病毒正在活跃复制。  你提到你的肝功能正常，但病毒数高。这可能意味着你的体内有大量的HBV，但你的肝脏目前还能正常工作。但是，高病毒负荷可能会增加肝脏疾病的风险。  在决定是否使用抗病毒药物时，医生通常会考虑以下因素：  1.病毒复制水平：HBV DNA水平高于一定的阈值可能需要治疗。  2.肝脏炎症和/或纤维化程度：通过肝脏生物检查或无创评估（例如肝硬度测量或血液标志物）可以评估。  3.病人的年龄、性别、家族病史、酒精消费和其他健康状况。  在你的情况下，最好是咨询医生，以确定最适合你的治疗方案。医生可能会建议定期进行肝功能检查和病毒载量检测，以监测病情的进展。如果有必要，他们可能会建议开始抗病毒治疗。 | 乙型肝炎（HBV）的治疗决定因素包括多种因素，如病毒复制活动（即病毒载量或病毒数）、肝炎活动程度（肝功能测试）、肝脏疾病的阶段（例如是否有肝硬化）以及患者的整体健康状况等。乙肝大三阳指的是HBsAg（乙型肝炎表面抗原）、HBeAg（乙型肝炎e抗原）和HBcAg（乙型肝炎核心抗原）都为阳性。  如果您的肝功能正常，但病毒数高，是否需要服用抗病毒药物需要根据您的具体情况来判断。一般来说，如果病毒载量持续高，即使肝功能测试结果正常，也可能需要抗病毒治疗，因为高病毒载量可能增加肝硬化和肝癌的风险。然而，这并非绝对的规则，因为有些人的免疫系统能够有效地控制病毒复制，即使病毒载量高也不会导致严重的肝病。  因此，我建议您咨询医生或专业医疗团队，他们会根据您的具体情况来做出最佳的治疗决策。这可能包括定期进行肝功能和病毒载量测试，以及可能的肝脏生物检查，以监测病情的进展并确定是否需要抗病毒治疗。  请注意，我所提供的信息仅供参考，不能替代专业医疗建议。 | No | Yes |
| 78 | Clinical Problems | Is it possible to return to positive after taking interferon for hepatitis B? | Yes, it is possible for people with chronic hepatitis B to see a positive response after taking Interferon. Interferon is one of the treatments used to manage chronic hepatitis B infection.  Interferon works by boosting the immune system to fight the hepatitis B virus. It can reduce the viral load (the amount of virus in the body), decrease inflammation of the liver, and in some cases, lead to the loss of hepatitis B surface antigen (HBsAg), a marker of active infection.  The ultimate goal of hepatitis B treatment is to achieve a “functional cure,” defined as the loss of HBsAg and the development of anti-HBs antibodies. This doesn’t mean the virus is completely eradicated from the body, but rather it’s controlled to the point where it’s not causing ongoing liver damage and the risk of transmitting the virus is greatly reduced.  However, not everyone responds to Interferon treatment. The response rate can vary widely, depending on factors such as the individual’s immune system, the genotype of the virus, and the stage of the disease.  It’s also important to note that Interferon treatment can have significant side effects, including flu-like symptoms, depression, and low white blood cell counts, which can lead to an increased risk of infections.  Therefore, the decision to use Interferon or any other treatment for chronic hepatitis B should be made in consultation with a healthcare provider, taking into account the potential benefits and risks. Regular monitoring is also necessary to assess the response to treatment and to detect any potential side effects. | Yes, it is possible for individuals with chronic Hepatitis B to become Hepatitis B surface antigen (HBsAg) negative after treatment with interferon or other antiviral medications. However, it’s important to note that this doesn’t necessarily mean that the virus has been completely eradicated from the body.  Chronic Hepatitis B is a long-term infection of the hepatitis B virus. It can cause serious liver damage and is a major risk factor for liver cancer. The goal of treatment is to prevent these complications by suppressing viral replication.  Interferon is one of the treatment options for chronic hepatitis B. It is an immunomodulatory drug, meaning it boosts the body’s immune response to the virus. It can be effective in suppressing the virus and reducing liver inflammation.  However, the rate of patients becoming HBsAg negative after interferon treatment is relatively low. According to some studies, about 3% to 7% of patients become HBsAg negative after one year of interferon treatment. The rate can be higher in certain subgroups of patients.  It’s also important to note that even if a patient becomes HBsAg negative, they may still have the virus in their liver cells in a dormant form. This is known as occult hepatitis B. Therefore, regular monitoring is still necessary.  In conclusion, while it is possible to become HBsAg negative after interferon treatment for chronic hepatitis B, the rates are relatively low, and it does not necessarily mean complete eradication of the virus. The decision to start treatment should be individualized and discussed thoroughly with a healthcare provider, considering the potential benefits and risks.  It’s also essential to remember that the best way to prevent hepatitis B is vaccination, which is safe and highly effective.  Disclaimer: This information is intended to be educational and should not be used as a substitute for professional medical advice. Always consult with a healthcare provider for advice about your health. | No | Yes |
| 81 | AASLD Guideline questions | Should adults with immune-tolerant infection be treated with antiviral therapy to decrease liver-related complications? | The decision to treat adults with immune-tolerant chronic hepatitis B infection is a complex one and should be made on a case-by-case basis.  The immune-tolerant phase of chronic hepatitis B is characterized by high levels of hepatitis B virus (HBV) DNA and hepatitis B e antigen (HBeAg) positivity, normal or minimally elevated alanine aminotransferase (ALT) levels, and minimal or no liver inflammation or fibrosis on biopsy. Historically, this phase was thought to be benign and treatment was not recommended due to the low risk of disease progression and the potential for adverse effects from long-term antiviral therapy.  However, more recent studies have suggested that immune-tolerant chronic hepatitis B may not be as benign as previously thought. Some patients may progress to cirrhosis and hepatocellular carcinoma (HCC) despite having normal ALT levels. In addition, it has been suggested that early antiviral therapy may be beneficial in preventing disease progression in these patients.  The decision to initiate antiviral therapy should be individualized and take into account several factors, including:  1.The patient’s age, as the risk of disease progression increases with age.  2.The presence of other risk factors for disease progression, such as male sex, family history of HCC, alcohol use, coinfection with other viruses (e.g., hepatitis C, HIV), and metabolic syndrome.  3.The potential risks and benefits of antiviral therapy, including the risk of drug resistance and side effects from long-term therapy.  Further research is needed to determine the optimal management strategy for adults with immune-tolerant chronic hepatitis B. Current guidelines from professional organizations such as the American Association for the Study of Liver Diseases (AASLD) and the European Association for the Study of the Liver (EASL) do not recommend routine antiviral therapy in the immune-tolerant phase, but they do suggest that it may be considered in certain high-risk patients.  For these reasons, it is crucial to have a thorough discussion with a healthcare provider who can provide personalized advice based on the patient’s individual circumstances. | While I’m an AI and can’t provide personal medical advice, I can provide some general information about this topic based on current scientific knowledge.  Immune-tolerant infection typically refers to a phase of chronic hepatitis B infection. This phase is characterized by high levels of HBV DNA and normal alanine aminotransferase (ALT) levels, with minimal or no inflammation or fibrosis on liver biopsy. The immune system is not actively fighting the virus, hence the term “immune-tolerant.”  The decision to treat adults with immune-tolerant hepatitis B infection with antiviral therapy is often complex and depends on various factors, including patient age, presence of family history of hepatocellular carcinoma (HCC), and patient preference.  As per the current guidelines by the American Association for the Study of Liver Diseases (AASLD) and the European Association for the Study of the Liver (EASL), antiviral therapy is generally not recommended for adults in the immune-tolerant phase of chronic hepatitis B, due to the low risk of disease progression and the potential for long-term side effects from the medication.  However, some studies suggest that early antiviral therapy could potentially decrease the risk of liver-related complications such as cirrhosis and HCC. This is an area of ongoing research.  Therefore, the decision to start antiviral therapy in immune-tolerant adults should be individualized, taking into account the potential benefits and risks. Regular monitoring of ALT and HBV DNA levels, as well as liver imaging, are essential to detect any changes in disease status.  It’s important for anyone with chronic hepatitis B, including those in the immune-tolerant phase, to discuss their treatment options with a healthcare provider who is knowledgeable about the disease.  Please note that this information might change as new research findings emerge. Always consult with a healthcare professional for the most accurate information. | No | Yes |
| 81 | AASLD Guideline questions | 免疫耐受感染的成人是否应接受抗病毒治疗以减少肝脏相关并发症？ | 免疫耐受性感染，特别是对于慢性乙型肝炎（CHB）的患者，是一个复杂的问题。免疫耐受期是指血清中乙型肝炎病毒（HBV）DNA水平高，血清中的乙型肝炎表面抗原（HBsAg）阳性，但是丙氨酸氨基转移酶（ALT）水平正常，肝活检显示炎症程度轻的状态。  对于是否应接受抗病毒治疗以减少肝脏相关并发症，这主要取决于患者的具体情况。一般来说，如果患者的病毒载量持续高，ALT水平持续正常或升高，并且/或者肝活检显示有中度至重度炎症或者纤维化，那么可能需要考虑抗病毒治疗。但是，如果患者在免疫耐受期并且没有明显的肝脏疾病症状或者病理改变，那么通常不推荐抗病毒治疗，因为这可能带来不必要的副作用和费用。  在做出决定时，医生会考虑许多因素，包括患者的年龄、性别、病毒载量、ALT水平、肝病历史、家族病史、合并症以及患者的生活质量等。因此，对于是否接受抗病毒治疗，应由医生和患者共同决定。  需要注意的是，这些建议可能会随着新的研究结果和治疗策略的发展而变化。因此，患者应该定期和医生进行讨论，以便获取最新的信息和建议。  这些信息仅供参考，并不能替代专业医疗建议。如果你有关于这个问题的疑虑，你应该咨询你的医生或者其他合格的医疗专业人士。  参考文献：  1.Terrault NA, Lok ASF, McMahon BJ, et al. Update on prevention, diagnosis, and treatment of chronic hepatitis B: AASLD 2018 hepatitis B guidance. Hepatology. 2018;67(4):1560-1599. doi:10.1002/hep.29800  2.European Association for the Study of the Liver. EASL 2017 Clinical Practice Guidelines on the management of hepatitis B virus infection. J Hepatol. 2017;67(2):370-398. doi:10.1016/j.jhep.2017.03.021  3.Sarin SK, Kumar M, Lau GK, et al. Asian-Pacific clinical practice guidelines on the management of hepatitis B: a 2015 update. Hepatol Int. 2016;10(1):1-98. doi:10.1007/s12072-015-9675-4 | 免疫耐受感染是指患者体内存在某种病原体，但是他们的免疫系统并未对此产生反应，也就没有明显的病症。对于这种情况，是否需要进行抗病毒治疗以减少肝脏相关并发症，是一个需要考虑多个因素的决定。  首先，需要明确病原体的类型。例如，对于慢性乙型肝炎病毒（HBV）感染，如果患者处于免疫耐受阶段（通常表现为血清中HBV DNA水平高，但ALT水平正常，肝脏炎症程度轻或无），一般不推荐抗病毒治疗。这是因为在这个阶段，抗病毒治疗的益处可能不会超过其可能的风险，如药物副作用和耐药性的发展。  然而，每个患者的情况都是独特的，所以应由医疗专业人员进行详细评估。他们会考虑患者的年龄、病毒载量、肝脏疾病的严重程度、合并症、以及患者的整体健康状况等因素。  此外，需要定期进行监测，以便在患者的免疫状态发生变化时，及时调整治疗策略。例如，如果HBV DNA水平持续高昂，ALT水平开始升高，或者肝脏病变进展，可能需要开始抗病毒治疗。  因此，免疫耐受感染的成人是否应接受抗病毒治疗以减少肝脏相关并发症，需要根据他们的具体情况和医生的专业建议来决定。 | Yes | No |
| 84 | AASLD Guideline questions | In HBV-monoinfected persons, does entecavir therapy, when compared to tenofovir therapy, have a different impact on renal and bone health? | As an AI model developed by OpenAI, I provide information based on the data I was trained on, up to my last training cut-off in 2021. As of that time, both entecavir and tenofovir are antiviral medications used to treat chronic hepatitis B virus (HBV) infection. The choice between the two may depend on various factors, including patient’s renal function, bone health, and the risk of drug resistance.  Tenofovir disoproxil fumarate (TDF), one form of tenofovir, has been associated with renal and bone toxicity. Renal toxicity may manifest as a decrease in estimated glomerular filtration rate (eGFR), and bone toxicity may result in a decrease in bone mineral density (BMD). This is due to the drug’s impact on proximal renal tubular function and its potential to cause renal tubular toxicity.  Entecavir, on the other hand, has not been shown to be associated with significant renal or bone toxicity. It is generally considered safer than TDF in terms of renal and bone health.  However, a newer form of tenofovir, known as tenofovir alafenamide (TAF), has been developed to lower the risk of renal and bone toxicity while maintaining strong antiviral efficacy. TAF provides a similar level of viral suppression as TDF but with less impact on the kidneys and bones.  That being said, the decision to use entecavir or tenofovir should be individualized, taking into consideration the patient’s overall health status, potential for drug resistance, and other factors.  Please consult with a healthcare provider for the most recent and relevant information as my knowledge is up to 2021 and does not include any new studies or findings that may have emerged after this time. | I’m an AI language model and can provide information based on available data up to the year 2021. As of that time, both entecavir (ETV) and tenofovir (TDF) are considered first-line treatments for chronic hepatitis B virus (HBV) infection.  In terms of renal and bone health:  1.Renal Health: Tenofovir has been associated with renal impairment, including acute kidney injury and Fanconi syndrome. This risk appears to be greater in individuals with pre-existing renal disease or other risk factors for renal dysfunction. On the other hand, entecavir has not been significantly associated with renal dysfunction.  2.Bone Health: Tenofovir has been linked with a decrease in bone mineral density (BMD), which can lead to osteoporosis and increased fracture risk over time. This risk appears to be higher in patients with risk factors for bone loss. As for entecavir, it has not been significantly associated with bone loss.  Therefore, while both drugs are effective against HBV, entecavir might have a more favorable safety profile regarding renal and bone health. However, it’s important to note that individual patient characteristics and comorbidities should always guide the choice of antiviral therapy.  Please consult with a healthcare provider for the most recent and personalized advice. This information might have changed after 2021, and newer drugs might have become available. | Yes | Yes |
| 86 | AASLD Guideline questions | Should persons with compensated cirrhosis and low levels of viremia be treated with antiviral agents? | The management of chronic viral hepatitis, particularly in patients with compensated cirrhosis and low levels of viremia, is a complex issue and depends on a variety of factors. These include the specific virus involved, the patient’s overall health, the presence of other co-morbidities, and the potential for adverse effects from antiviral therapy.  For Hepatitis B, the American Association for the Study of Liver Diseases (AASLD) recommends antiviral therapy for all patients with compensated cirrhosis and detectable HBV DNA, regardless of the level of viremia. This is due to the risk of disease progression and the development of hepatocellular carcinoma.  For Hepatitis C, with the advent of direct-acting antivirals (DAAs), the level of viremia is no longer a primary determinant for treatment. The AASLD and Infectious Diseases Society of America (IDSA) recommend treating all patients with chronic Hepatitis C infection, including those with compensated cirrhosis, due to the high cure rates and the potential to prevent disease progression.  However, individual patient factors must always be taken into consideration and decisions should be made in consultation with a healthcare provider experienced in the management of viral hepatitis.  Please note that this information is accurate as of the current date and guidelines may change as more research becomes available.  Disclaimer: This information is intended for educational purposes only and should not be used as a substitute for professional medical advice. Always consult with a healthcare provider for advice about your health. | The decision to treat persons with compensated cirrhosis and low levels of viremia with antiviral agents depends on a variety of factors and should always be individualized. It should be made in consultation with the patient and their healthcare provider, considering the potential benefits and risks of treatment.  Here are a few factors to consider:  1.The type of virus causing the viremia: For instance, Hepatitis B and C viruses can cause cirrhosis, and there are effective antiviral therapies for both. Antiviral therapy can slow disease progression and reduce the risk of complications in these cases.  2.The patient’s overall health status: If the patient has significant comorbidities that might be worsened by antiviral therapy, or if their life expectancy is limited due to other factors, the risks of therapy may outweigh the benefits.  3.The stage of cirrhosis: In compensated cirrhosis, the liver is still able to perform its essential functions, despite the damage. The decision to initiate antiviral therapy might depend on the degree of liver function remaining and the risk of progression to decompensated cirrhosis.  4.Potential for disease transmission: Antiviral therapy can reduce the viral load and potentially decrease the risk of transmitting the virus to others.  5.Patient preferences and quality of life considerations: Some patients may choose to take antiviral therapy to reduce the risk of future health problems, even if their current symptoms are mild or nonexistent.  6.Risk of hepatocellular carcinoma (HCC): Patients with cirrhosis are at an increased risk of developing HCC. Antiviral therapy can potentially reduce this risk.  In general, the American Association for the Study of Liver Diseases (AASLD) recommends antiviral therapy for all patients with Hepatitis B virus (HBV) and cirrhosis, regardless of their level of viremia, because of the risk of disease progression and development of HCC.  For Hepatitis C virus (HCV), the decision to treat should be individualized, considering the patient’s comorbidities and life expectancy. However, the advent of direct-acting antiviral (DAA) therapy has made treatment of HCV easier and more effective, and it is generally recommended for all patients with HCV and cirrhosis.  In summary, while low levels of viremia might suggest a less aggressive disease course, the presence of cirrhosis indicates significant liver disease, and antiviral therapy should be strongly considered. However, the decision should be individualized based on the factors discussed above. Always consult with a healthcare provider for personalized advice. | Yes | No |
| 88 | AASLD Guideline questions | Should children with HBeAg-positive CHB be treated with antiviral therapy to decrease liver-related complications? | Chronic Hepatitis B (CHB) is a significant health concern worldwide. The decision to initiate antiviral therapy in children with HBeAg-positive CHB is complex and should be individualized based on several factors.  Several professional societies have developed guidelines for the management of CHB in children. According to the guidelines from the American Association for the Study of Liver Diseases (AASLD) and the European Association for the Study of the Liver (EASL), antiviral therapy should be considered in children with HBeAg-positive CHB who have persistently elevated alanine aminotransferase (ALT) levels and/or significant histological disease (moderate to severe inflammation and/or fibrosis).  It’s important to note that the goal of treatment in children with CHB is to reduce the risk of long-term complications, such as cirrhosis and hepatocellular carcinoma, by suppressing viral replication and thereby decreasing liver inflammation and fibrosis.  However, there are some caveats:  1.Long-term treatment: Children who start antiviral therapy may require long-term or even lifelong treatment, given the low rates of HBeAg seroconversion. This could increase the risk of antiviral resistance and affect the child’s quality of life.  2.Potential side effects: Antiviral agents have potential side effects, which need to be considered.  3.Drug resistance: Long-term treatment can lead to drug resistance, especially with drugs that have a low genetic barrier to resistance.  Therefore, the decision to initiate antiviral therapy in children with HBeAg-positive CHB should be based on a careful assessment of the potential benefits and risks, and the child’s overall health, growth, and development should be monitored closely during treatment.  This information should not replace a consultation with a healthcare professional. It’s always recommended to discuss these matters with a qualified healthcare provider.  Please note that the field of medicine is continually evolving, and new research may change the current understanding and guidelines.  Sources:  1.AASLD 2018 Hepatitis B Guidance  2.EASL 2017 Clinical Practice Guidelines on the management of hepatitis B virus infection | HBeAg-positive chronic hepatitis B (CHB) in children is a significant global health problem. The management of HBeAg-positive CHB in children is complex and should be individualized based on several factors such as the child’s age, disease phase, the presence of liver disease (e.g., cirrhosis), family history of hepatocellular carcinoma (HCC), and the child’s ability to adhere to treatment.  Antiviral therapy can be beneficial in children with HBeAg-positive CHB, but it is not without potential risks. The primary goal of antiviral therapy is to prevent or delay the progression of liver disease, reduce the risk of hepatocellular carcinoma, and improve the quality of life.  In general, antiviral treatment is recommended for children with HBeAg-positive CHB who have:  1.Persistent or intermittent elevations in alanine aminotransferase (ALT) levels.  2.Evidence of moderate to severe inflammation or fibrosis on liver biopsy.  3.A family history of HCC.  Treatment should be considered in children with normal ALT levels and evidence of moderate to severe inflammation or fibrosis on liver biopsy.  However, the long-term safety and efficacy of antiviral therapy in children are not as well-established as in adults. Moreover, the risk of drug resistance, potential side effects, and the possibility of life-long treatment should be taken into account.  It is important to note that antiviral therapy does not cure CHB but controls the virus. Therefore, regular monitoring of viral load, HBeAg status, and liver function is essential during and after treatment.  In conclusion, the decision to treat a child with HBeAg-positive CHB with antiviral therapy should be made on a case-by-case basis, considering the potential benefits and risks. It is crucial to involve a multidisciplinary team, including a pediatric hepatologist, in the decision-making process.  Please note that this information is based on the current understanding and guidelines. It is always advisable to consult with a healthcare provider for the most appropriate treatment options.  Disclaimer: This information is intended for educational purposes only and should not be used as a substitute for professional medical advice, diagnosis, or treatment. Always seek the advice of your physician or other qualified health provider with any questions you may have regarding a medical condition. | No | Yes |
| 88 | AASLD Guideline questions | 阳性慢性乙型病毒性肝炎患儿是否应接受抗病毒治疗以减少肝脏相关并发症？ | 乙型病毒性肝炎（HBV）是一种慢性疾病，如果不进行治疗，可能会导致肝硬化、肝衰竭、肝癌等严重并发症。在儿童中，乙型病毒性肝炎的进展速度通常比成人慢，但是，对于阳性慢性乙型病毒性肝炎的儿童，仍然需要考虑抗病毒治疗。  抗病毒治疗的目的是阻止或延缓肝脏病变的进展，减少并发症的发生。然而，是否开始抗病毒治疗并不是一项简单的决定。需要考虑多种因素，包括患者的年龄、疾病的严重程度（如ALT水平，HBV DNA水平，肝脏病变的程度等）、以及患者的个体情况等。  对于儿童，需要特别考虑的是，抗病毒药物可能需要长期使用，而这些药物可能有一些长期的副作用。因此，治疗决策需要在医生、患儿及其家庭之间进行充分的讨论，以权衡治疗的益处和可能的风险。  在一些指南中，如美国肝病学会（AASLD）和欧洲肝病学会（EASL）的指南，建议对于年龄大于2岁，且有活动性病变（ALT水平升高，HBV DNA水平高，肝脏病变明显）的儿童，可以考虑进行抗病毒治疗。  总的来说，对于阳性慢性乙型病毒性肝炎的儿童，应该根据其具体情况来决定是否进行抗病毒治疗。这需要医生根据患儿的病情，以及各种可能的风险和益处，进行个体化的治疗决策。  请注意，这些信息仅供参考，并不能替代医生的专业医疗建议。如果你或你的孩子有任何健康问题，你应该立即联系医疗专业人士。  参考文献：  1.Terrault NA, Lok ASF, McMahon BJ, et al. Update on prevention, diagnosis, and treatment of chronic hepatitis B: AASLD 2018 hepatitis B guidance. Hepatology. 2018;67(4):1560-1599.  2.European Association for the Study of the Liver. EASL 2017 Clinical Practice Guidelines on the management of hepatitis B virus infection. J Hepatol. 2017;67(2):370-398. | 乙型病毒性肝炎（HBV）是一种全球范围内的公共卫生问题。在儿童中，它通常是通过母婴传播或在儿童早期通过接触传播的。乙型病毒性肝炎的治疗旨在防止肝硬化、肝癌和其他肝脏相关并发症。  对于阳性慢性乙型病毒性肝炎的儿童是否应接受抗病毒治疗，这取决于多个因素，包括病毒复制的活跃性、肝炎活动的严重性（例如，ALT水平）、肝脏疾病的严重性（例如，肝纤维化或肝硬化的证据），以及患者的年龄和共患疾病。此外，也要考虑到潜在的药物副作用和耐药性的发展。  一般来说，如果患儿存在以下情况，可能需要抗病毒治疗：  1.高水平的HBV DNA复制  2.ALT水平持续升高  3.肝脏病理学检查显示中度或重度的肝炎和/或显著的肝纤维化  治疗药物包括干扰素和核苷（酸）类似物。干扰素具有抗病毒、抗增殖和免疫调节作用，而核苷（酸）类似物则通过抑制HBV DNA聚合酶来阻止病毒复制。  总的来说，对于阳性慢性乙型病毒性肝炎的儿童，是否接受抗病毒治疗应由医生根据每个患儿的具体情况来决定。医生需要平衡治疗的潜在益处和可能的风险，并与患儿和家庭进行充分的讨论。 | Yes | No |
| 91 | Simulated Patients | 一个37岁的女性患者，HBV-DNA 10^7 IU/ml，HBeAg阳性，有肝硬化，eGFR 102 ml/min，ALT 215 U/L，BMI 24.3kg/m^2，没有饮酒习惯，刚开始服用替诺福韦治疗。这位患者日常监测的频率应当为多久一次？项目应当包括哪些？ | 这位患者是慢性乙型肝炎（CHB）合并肝硬化的患者，现在开始使用替诺福韦进行抗病毒治疗。对于这类患者，我们通常会建议以下的监测方案：  1.治疗早期阶段（治疗的头三个月）：每月进行一次血常规、肝功能（包括ALT、AST、TBil、DBil、ALP、GGT、TP、Alb）、肾功能（包括BUN、Cr、eGFR）、电解质（包括K、Na、Cl、Ca）、尿常规、HBV-DNA定量、HBeAg/抗HBe、AFP等检测。这是为了及时了解药物的疗效和潜在的副作用。  2.治疗稳定阶段（治疗后三个月以后）：如果患者的病情稳定，HBV-DNA水平明显下降，肝肾功能正常，可以适当延长监测的间隔，比如每3个月进行一次血常规、肝功能、肾功能、电解质、尿常规、HBV-DNA定量、HBeAg/抗HBe、AFP等检测。  3.治疗中期和长期阶段：每6个月进行一次肝脏的影像学检查（如肝脏超声、MRI或CT），以及食管胃底静脉曲张的筛查（如胃镜检查）。  4.如果患者出现疲乏、食欲不振、恶心、呕吐、尿色深、大便色浅、皮肤或眼睛黄染等症状，应立即进行相关检查。  以上监测方案是一种常规的建议，具体的监测频率和项目应根据患者的具体病情和临床反应来定。 | 首先，让我们明确一点：所有的医学建议应当由医生或医疗专业人员提供。我可以提供一些关于慢性乙型肝炎病毒（HBV）感染的一般监测和管理建议，但这些仅供参考，不能替代专业医疗建议。  这位患者的情况下，她的HBV-DNA水平非常高，表明病毒复制活跃，而且她已经有肝硬化的情况。这可能需要更密集的监测。替诺福韦是一种抗病毒药物，常用于治疗乙型肝炎。  通常，对于正在接受抗病毒治疗的慢性乙型肝炎患者，以下是一些可能的监测项目和频率：  1.ALT（丙氨酸氨基转移酶）：ALT是肝脏疾病的一个重要指标。这位患者的ALT已经超过正常范围，需要密切监测。通常，治疗初期可以每1-3个月进行一次ALT检测。  2.HBV-DNA：HBV-DNA水平可以反映病毒复制的活跃程度。治疗初期，可以每3个月进行一次HBV-DNA检测。  3.HBeAg：HBeAg阳性表示病毒复制活跃。治疗过程中，如果HBeAg转为阴性，可能表示病毒活动降低。可以每6-12个月进行一次HBeAg检测。  4.肝硬度：由于这位患者已经有肝硬化，可能需要定期进行肝硬度或肝脏超声检查，以监测肝脏疾病的进展。频率可以每6-12个月一次。  5.肾功能：替诺福韦可能对肾功能产生影响。这位患者的eGFR（估计肾小球滤过率）表明她的肾功能目前正常，但在治疗过程中可能需要定期进行肾功能检查，包括血肌酐和尿蛋白。频率可以每3-6个月一次。  以上都是一般的建议，具体的监测项目和频率应由医生根据患者的具体情况来决定。 | No | Yes |
| 93 | Simulated Patients | A 44-year-old female patient with HBV-DNA 10^8 IU/ml, HBeAg positive, no cirrhosis, eGFR 105 ml/min, ALT 110 U/L, BMI 22.5kg/m^2, a history of hypertension, smoking and drinking habits, and no antiviral therapy. Please advise the medication regimen and lifestyle of the patient. | The patient’s profile suggests chronic Hepatitis B infection with high viral load and elevated ALT, indicating active viral replication and liver inflammation. This necessitates antiviral therapy. The patient also has risk factors such as hypertension, smoking, and drinking habits that need to be addressed.  Here is a suggested management plan:  Antiviral Therapy  The first-line treatment options for chronic Hepatitis B include:  1.Entecavir (ETV): It’s a potent antiviral with a high barrier to resistance. The usual dose is 0.5 mg once daily.  2.Tenofovir disoproxil fumarate (TDF) or Tenofovir alafenamide (TAF): TDF (300 mg once daily) or TAF (25 mg once daily) are potent antivirals with a high barrier to resistance. They are also recommended as first-line therapy. TAF has a better renal and bone safety profile than TDF.  Given the patient’s eGFR is 105 ml/min, either ETV, TDF, or TAF could be considered. However, TAF might be a safer option considering the patient’s history of hypertension.  Lifestyle Modification  1.Quit Smoking and Drinking: It is crucial for the patient to stop smoking and alcohol consumption, as both can exacerbate liver damage. She should be referred to smoking cessation and alcohol cessation programs.  2.Healthy Diet and Exercise: The patient should maintain a balanced diet, rich in fruits, vegetables, lean proteins, and whole grains. Regular exercise will also help maintain a healthy weight and lower blood pressure.  3.Regular Monitoring: The patient should have regular follow-ups with her doctor to monitor her liver function, viral load, and the effectiveness of the treatment.  4.Vaccination: Vaccination against Hepatitis A and pneumococcal disease is recommended because of the increased risk of severe disease in individuals with liver disease. The patient should also receive an annual flu shot.  5.Blood Pressure Control: The patient’s hypertension should be managed according to standard guidelines. This may include lifestyle modifications and antihypertensive medications.  6.Safe Practices: To prevent transmission to others, the patient should avoid sharing personal items such as toothbrushes, razors, or needles. She should also practice safe sex.  Disclaimer: This is a general advice based on the information provided and might not be fully applicable to the patient. The patient should consult with her healthcare provider for a personalized treatment plan.  Please note that any changes to medication or lifestyle should be discussed and monitored by a healthcare provider. | Given the patient’s condition, it is recommended to start antiviral therapy to reduce the risk of disease progression. Based on the patient’s high viral load (HBV-DNA  IU/ml), HBeAg positivity, elevated ALT levels, and absence of cirrhosis, first-line antiviral medications would be appropriate.  Medication Regimen  The first-line antiviral medications for Hepatitis B include:  1.Entecavir (ETV): This is a potent inhibitor of HBV replication with a high barrier to resistance. The recommended dose is 0.5 mg once daily.  2.Tenofovir Disoproxil Fumarate (TDF): This is another potent inhibitor of HBV replication with a high barrier to resistance. The recommended dose is 300 mg once daily.  3.Tenofovir Alafenamide (TAF): This is a newer prodrug of tenofovir that is associated with less renal and bone toxicity. The recommended dose is 25 mg once daily.  Given the patient’s good renal function (eGFR 105 ml/min), either of these medications could be used. However, the choice between these medications should take into account the patient’s comorbid conditions, potential side effects, and the patient’s preferences.  The patient should also continue to manage her hypertension, potentially with antihypertensive medication if it is not already controlled. The specific antihypertensive medication would depend on the patient’s blood pressure levels, other medical conditions, and potential side effects.  Lifestyle Changes  In addition to medication, lifestyle changes are also crucial for managing Hepatitis B and reducing the risk of disease progression:  1.Quit Smoking and Limit Alcohol: Smoking and alcohol can increase liver damage and reduce the effectiveness of treatment. The patient should be advised to quit smoking and limit or avoid alcohol.  2.Healthy Diet: A balanced diet rich in fruits, vegetables, lean proteins, and whole grains can help maintain overall health and support liver function.  3.Regular Exercise: Regular physical activity can help maintain a healthy weight and overall health. The patient’s BMI is currently in the normal range, so the goal of exercise would be to maintain this.  4.Regular Monitoring: Regular follow-ups with the healthcare provider are necessary to monitor the patient’s liver function, viral load, and response to treatment.  5.Avoid Sharing Personal Items: The patient should avoid sharing personal items like toothbrushes and razors to prevent spreading the virus to others.  6.Safe Sex Practices: Use of condoms can prevent the transmission of the virus to sexual partners.  7.Vaccination: Family members and close contacts who are not already immune to Hepatitis B should be vaccinated.  Remember, this is just a general recommendation and the specific treatment plan should be personalized based on the patient’s individual circumstances. It’s important that the patient discusses these recommendations with her healthcare provider. | Yes | No |
| 94 | Simulated Patients | A 50-year-old male patient, HBeAg positive, ALT 231 U/L, with hyperlipidemia and currently not on antiviral therapy, came to console whether interferon could be used. Please give medical advice. | The patient in question is HBeAg positive, which indicates active viral replication of hepatitis B, and has an elevated ALT level (Alanine Aminotransferase, a liver enzyme), suggesting liver inflammation or damage. This usually necessitates antiviral treatment.  Interferon is one of the treatment options for chronic hepatitis B. It’s an immune modulator that enhances the body’s immune response to the virus, aiming to achieve sustained viral suppression even after treatment cessation. Interferon can be used in certain patients with chronic hepatitis B infection, but it’s not suitable for everyone due to its side effects and contraindications.  However, the patient also has hyperlipidemia, a condition characterized by high levels of fats (lipids) in the blood. Interferon therapy is not directly contraindicated in patients with hyperlipidemia, but it can cause a wide range of side effects including flu-like symptoms, mood disturbances, thyroid dysfunction, and, importantly, it may exacerbate existing conditions.  Before deciding on interferon therapy, several factors should be considered:  1.The patient’s overall health status: If the patient has significant comorbidities, interferon might not be the best choice due to its potential side effects.  2.The patient’s lifestyle and personal preferences: Interferon treatment involves regular injections, usually for a period of 48 weeks. This can have a significant impact on the patient’s quality of life.  3.The patient’s viral load and liver disease stage: Interferon is generally more effective in patients with lower viral loads and less advanced liver disease.  4.Potential for drug interactions: If the patient is taking medication for hyperlipidemia, potential interactions with interferon should be evaluated.  In conclusion, while interferon could potentially be used in this patient, it’s crucial to discuss the potential risks and benefits with the patient, considering his overall health status, lifestyle, and personal preferences. Moreover, monitoring the patient’s condition closely during treatment would be essential.  As an AI language model, I can provide general advice, but the final decision should be made by a healthcare professional based on a comprehensive evaluation of the patient’s condition. | Based on the information provided, the patient is a 50-year-old male with HBeAg-positive chronic hepatitis B, elevated ALT levels (231 U/L), and hyperlipidemia.  Interferon is one of the treatment options for chronic hepatitis B. It works by boosting the immune system to fight the virus. It’s often considered as a treatment option due to its finite treatment duration and lack of resistance. However, the use of interferon is limited by its side effects and contraindications. Side effects can include flu-like symptoms, depression, and a drop in white blood cells.  As for contraindications, interferon is generally not recommended for patients with:  1.Decompensated cirrhosis.  2.A history of severe psychiatric disease, especially depression.  3.Autoimmune disease.  4.Uncontrolled thyroid disease.  5.Heart disease.  6.Seizure disorders.  In this case, the patient’s hyperlipidemia would not necessarily preclude the use of interferon, but the patient’s overall health status, including liver disease stage, cardiovascular risk, and mental health, should be thoroughly evaluated.  Also, it’s important to note that the patient’s elevated ALT levels may indicate active liver inflammation or damage. This should be further investigated to understand the extent of liver disease.  Before making any treatment decisions, further tests should be performed including:  1.Hepatitis B virus DNA level  2.Liver ultrasound  3.Complete blood count  4.Coagulation parameters  5.Tests to rule out co-infection with hepatitis C, hepatitis D, and HIV  6.Possibly a liver biopsy  The patient should also be counseled on lifestyle modifications including a healthy diet, regular exercise, avoidance of alcohol, and regular monitoring.  Remember that the ultimate decision on treatment type and timing should be individualized, taking into account the patient’s age, disease phase, viral replication, ALT level, presence of liver inflammation or fibrosis, family history of hepatocellular carcinoma, patient’s willingness, and possible side effects.  Please consult with a hepatologist or an infectious disease specialist for a comprehensive evaluation and personalized treatment plan.  Disclaimer: This advice is intended to be general in nature, and specific causes may not apply to individual cases. It should not replace consultation with a healthcare provider. | Yes | Yes |
