# Supplementary material for: Assessing ChatGPT as a Medical Consultation Assistant for Chronic Hepatitis B: Cross-Language Study of English and Chinese
Source: JMIR Med Inform. 2024 Aug 8;12:e56426. doi: 10.2196/56426 (PMC11342014; doi:10.2196/56426)
Supplement: Multimedia Appendix 15 [file medinform_v12i1e56426_app15.docx]

**Multimedia Appendix 15.** Results of responses to closed questions (true-or-false) in ChatGPT-3.5 and ChatGPT-4.0.

| Number | Question | Standard Answer | ChatGPT-4.0 | | | ChatGPT-3.5 | | |
| --- | --- | --- | --- | --- | --- | --- | --- | --- |
|  |  |  | Response1 | Response2 | Response3 | Response1 | Response2 | Response3 |
| 1 | Male adults with immune active CHB should be treated with antiviral therapy to decrease liver-related complications. | Yes | Yes | Yes | Yes | Yes | Yes | Yes |
| 1 | 免疫活动性CHB男性成人患者都应接受抗病毒治疗以减少肝脏相关并发症。 | 是 | 是 | 是 | 是 | 是 | 是 | 否 |
| 2 | Antiviral therapy is not recommended for female adults with immune active CHB to decrease adverse drug reaction. | No | No | No | No | No | No | No |
| 2 | 免疫活动性的CHB女性成人患者不推荐进行抗病毒治疗以减少药物不良反应的发生。 | 否 | 否 | 否 | 否 | 是 | 是 | 是 |
| 3 | Adults with immune-tolerant infection should be treated with antiviral therapy to decrease liver-related complications. | No | No | No | No | No | No | No |
| 3 | 免疫耐受感染的成人应接受抗病毒治疗以减少肝脏相关并发症 | 否 | 否 | 否 | 是 | 是 | 是 | 是 |
| 4 | There is no evidence of benefits from ALT tests for adults with immune-tolerant infection of hepatitis B virus. | No | No | No | No | Yes | Yes | No |
| 4 | 确诊免疫耐受感染CHB的成人不再需要检测ALT水平。 | 否 | 否 | 否 | 否 | 是 | 是 | 是 |
| 5 | Moderate-to-severe necroinflammation or fibrosis on a liver biopsy specimen is a reason to stop antiviral therapy to avoid further liver-related complications. | No | No | No | No | No | No | No |
| 5 | 肝活检中出现中度至重度坏死性炎症或纤维化是停止抗病毒治疗以避免更严重的肝脏相关并发症的重要指征。 | 否 | 否 | 否 | 否 | 否 | 是 | 是 |
| 6 | Immune-tolerant status should be defined by albumin levels, utilizing 35 g/L for men and 25 g/L for women as ULN (upper limits of normal). | No | No | No | No | No | Yes | Yes |
| 6 | 白蛋白水平是确诊CHB患者免疫耐受的关键指标，其中以男性35g/L，女性25 g/L作为正常上限值（ULN）。 | 否 | 否 | 否 | 否 | 是 | 是 | 是 |
| 7 | Antiviral therapy should be discontinued in hepatitis B e antigen (HBeAg)-positive persons who have developed HBeAg seroconversion on therapy with persistently normal ALT levels and undetectable serum HBV DNA levels for 6-8 weeks. | No | No | No | No | Yes | No | No |
| 7 | 乙型肝炎e抗原(HBeAg)阳性患者在治疗中发生HBeAg血清学转换后，出现持续正常的ALT水平和无法检测的血清HBV DNA水平6-8周后应当停止抗病毒治疗。 | 否 | 否 | 否 | 否 | 是 | 是 | 否 |
| 8 | Antiviral therapy should be discontinued immediately in persons with HBcAb-negative infection with sustained HBV DNA suppression on therapy. | No | No | No | No | No | No | No |
| 8 | 对于治疗后HBV DNA持续抑制的HBcAb阴性感染者，应立即停止抗病毒治疗。 | 否 | 否 | 否 | 否 | 否 | 否 | 否 |
| 9 | Chronic hepatitis B patients who stop antiviral therapy should be monitored every 1 month for at least 3 years for recurrent viremia, ALT flares, seroreversion, and clinical decompensation. | No | No | No | No | Yes | No | Yes |
| 9 | 停止抗病毒治疗的慢乙肝患者应每1个月监测一次病毒血症复发、ALT爆发、血清学转换和临床代偿失偿，持续至少3年。 | 否 | 是 | 否 | 否 | 是 | 是 | 是 |
| 10 | Antiviral therapy should be discontinued in all HBeAg-positive patients after 3 years of antiviral therapy, regardless of seroreversion. | No | No | No | No | No | No | No |
| 10 | 所有HBeAg阳性患者在接受抗病毒治疗3年后，无论有无发生血清学转换，都应暂停抗病毒治疗。 | 否 | 否 | 否 | 否 | 否 | 否 | 否 |
| 11 | Indefinite antiviral therapy is suggested for HBeAg-positive adults with cirrhosis with CHB who seroconvert to anti-HBe on NA therapy, based on concerns for potential clinical decompensation and death, unless there is a strong competing rationale for treatment discontinuation. | Yes | Yes | Yes | Yes | Yes | Yes | Yes |
| 11 | 合并肝硬化的HBeAg阳性CHB成人患者即使血清转化为抗HBe，也应无限期接受NAs治疗，以消除对潜在临床失代偿和死亡的担忧，除非有强有力的理由要求停止治疗。 | 是 | 是 | 是 | 是 | 是 | 是 | 是 |
| 12 | In persons with HBeAg-negative infection with sustained HBV DNA suppression on therapy, antiviral therapy should be performed immediately once ALT flare is tested. | No | No | No | No | No | No | No |
| 12 | 对于治疗后HBV DNA持续抑制的HBeAg阴性感染者，停药后一旦监测到ALT爆发，就应当立即进行抗病毒干预。 | 否 | 否 | 否 | 否 | 是 | 否 | 否 |
| 13 | In persons with HBsAg-negative infection with sustained HBV DNA suppression on therapy, antiviral therapy should be performed immediately once AST flare is tested. | No | No | No | No | No | No | No |
| 13 | 对于治疗后HBV DNA持续抑制的HBsAg阴性感染者，停药后一旦监测到AST升高，就应当立即进行抗病毒干预。 | 否 | 否 | 否 | 否 | 否 | 否 | 否 |
| 14 | As long as the ALT level is low enough when antiviral therapy is stopped, there is no risk of recurrent toxemia after discontinuation. | No | No | No | No | No | No | No |
| 14 | 只要停止抗病毒治疗时ALT水平足够低，停药后就不会有病毒血症复发的风险。 | 否 | 否 | 否 | 否 | 否 | 否 | 是 |
| 15 | Antiviral therapy is not recommended for persons without cirrhosis who are HBeAg negative with normal ALT activity and low-level viremia. | Yes | Yes | Yes | Yes | Yes | Yes | Yes |
| 15 | 对于不伴有肝硬化的HBeAg阴性、ALT水平正常和低水平病毒血症的患者，不推荐抗病毒治疗。 | 是 | 是 | 是 | 是 | 是 | 是 | 是 |
| 16 | In HBV-monoinfected persons, entecavir therapy has a different impact on renal and bone health when compared to tenofovir therapy. | No | No | No | No | Yes | Yes | Yes |
| 16 | 在HBV单一感染者中，恩替卡韦治疗与替诺福韦治疗对肾脏和骨骼健康的影响不同。 | 否 | 是 | 是 | 是 | 是 | 是 | 是 |
| 17 | In HBV-monoinfected persons, tenofovir alafenamide therapy has a different impact on renal and bone health when compared to tenofovir therapy. | Yes | Yes | Yes | Yes | Yes | Yes | Yes |
| 17 | 在HBV单一感染者中，丙酚替诺福韦治疗与替诺福韦治疗对肾脏和骨骼健康的影响不同。 | 是 | 是 | 是 | 是 | 是 | 是 | 否 |
| 18 | In HBV-monoinfected persons, treated with entecavir, tenofovir should be switched to tenofovir as soon as kidney and bone problems develop. | No | No | No | No | No | Yes | No |
| 18 | 在接受恩替卡韦治疗的HBV单一感染者中，一旦患者出现肾脏和骨骼问题，应当立即换用替诺福韦。 | 否 | 否 | 否 | 是 | 否 | 是 | 是 |
| 19 | Adding a second antiviral agent is benefitial in persons with persistent low levels of viremia while being treated with either tenofovir or entecavir. | No | No | No | No | No | No | No |
| 19 | 在接受替诺福韦或恩替卡韦治疗的持续低水平病毒血症患者中，加用第二种抗病毒药物必然有益。 | 否 | 否 | 否 | 否 | 是 | 是 | 是 |
| 20 | In all persons with persistent low levels of viremia receiving tenofovir or entecavir monotherapy, elevated ALT level activity indicates that a second antiviral agent should be added immediately. | No | No | No | No | No | No | No |
| 20 | 对所有接受替诺福韦或恩替卡韦单药治疗的持续低水平病毒血症患者而言，ALT水平活动性上升说明应当立即加用第二种抗病毒药物。 | 否 | 否 | 否 | 否 | 否 | 是 | 否 |
| 21 | Persons with compensated cirrhosis and low levels of viremia shoul be treated with antiviral agents. | Yes | Yes | Yes | Yes | No | Yes | Yes |
| 21 | 有低病毒血症的代偿期肝硬化患者应当接受抗病毒药物治疗。 | 是 | 是 | 是 | 是 | 是 | 是 | 是 |
| 22 | Antiviral therapy is not recommended for all patients with normal ALT levels to avoid potential kidney and bone health risks. | No | Yes | Yes | Yes | No | No | No |
| 22 | 对于所有ALT水平正常的慢乙肝患者都不建议进行抗病毒治疗以避免出现潜在的肾脏和骨骼健康风险。 | 否 | 否 | 否 | 否 | 否 | 是 | 是 |
| 23 | For CHB patients with compensatory cirrhosis, interferon therapy is safer than NAs. | No | No | No | No | No | No | No |
| 23 | 对于伴有代偿性肝硬化的慢乙肝患者，干扰素治疗比核苷（酸）类药物更安全。 | 否 | 否 | 否 | 否 | 否 | 否 | 否 |
| 24 | Patients with chronic hepatitis B who receive antiviral therapy need not worry about the risk of hepatocellular carcinoma. | No | No | No | No | No | No | No |
| 24 | 接受抗病毒治疗的慢乙肝患者不必担心患肝细胞癌的风险。 | 否 | 否 | 否 | 否 | 是 | 否 | 是 |
| 25 | Pregnant women who are hepatitis B surface antigen (HBsAg) positive with high viral load should receive antiviral treatment in the third trimester to prevent perinatal transmission of HBV. | Yes | Yes | Yes | Yes | Yes | Yes | Yes |
| 25 | 高病毒载量的乙型肝炎表面抗原(hepatitis B surface antigen, HBsAg)阳性孕妇应该在妊娠晚期接受抗病毒治疗以预防HBV的围产期传播。 | 是 | 是 | 是 | 是 | 是 | 是 | 是 |
| 26 | All infants of HBsAg-positive women should be immunized. | Yes | Yes | Yes | Yes | Yes | Yes | Yes |
| 26 | 所有HBsAg阳性妇女的婴儿都应接受免疫预防。 | 是 | 是 | 是 | 是 | 是 | 是 | 是 |
| 27 | Pregnant women with chronic hepatitis B should start antiviral therapy early in pregnancy and stop immediately after delivery. | No | No | No | No | No | No | No |
| 27 | 患有CHB的孕妇应在怀孕早期开始抗病毒治疗，产后应立即停止。 | 否 | 否 | 否 | 否 | 是 | 是 | 否 |
| 28 | Pregnant women with chronic hepatitis B who receive antiviral therapy should continue to monitor ALT levels for 28-32 weeks postpartum. | No | Yes | Yes | Yes | Yes | Yes | Yes |
| 28 | 接受抗病毒治疗的患慢乙肝的孕妇应在产后对ALT水平进行28-32周的持续监测。 | 否 | 否 | 否 | 否 | 是 | 是 | 是 |
| 29 | All children with HBeAg-positive chronic viral hepatitis B (0-18 years of age) should receive antiviral therapy with the goal of achieving sustained HBeAg seroconversion. | No | No | No | No | Yes | No | Yes |
| 29 | 所有HBeAg阳性慢性乙型病毒性肝炎患儿（0-18岁）都应进行抗病毒治疗，目标是实现持续的HBeAg血清转化。 | 否 | 否 | 否 | 否 | 否 | 是 | 否 |
| 30 | Children with chronic hepatitis B who discontinue antiviral therapy (2-18 years) should be monitored for recurrent venereal toxemia, ALT levels, and clinical decompensation at least every month for at least 3 years. | No | No | No | No | No | Yes | No |
| 30 | 停止抗病毒治疗的慢乙肝患儿（2-18岁）应至少每1个月监测一次复发性病毒血症、ALT水平和临床失代偿，持续至少3年。 | 否 | 否 | 否 | 否 | 是 | 是 | 是 |
